# Supplementary material for: Structural insights into tecovirimat antiviral activity and poxvirus resistance
Source: Nat Microbiol. 2025 Feb 12;10(3):734–48. doi: 10.1038/s41564-025-01936-6 (PMC11879855; doi:10.1038/s41564-025-01936-6)
Supplement: Supplementary file 1 — Supplementary Fig. 1 and Tables 1–7. [file 41564_2025_1936_MOESM1_ESM.pdf]

nature microbiology

Supplementary information

https://doi.org/10.1038/s41564-025-01936-6

Structural insights into tecovirimat antiviral activity and poxvirus resistance

In the format provided by the authors and unedited

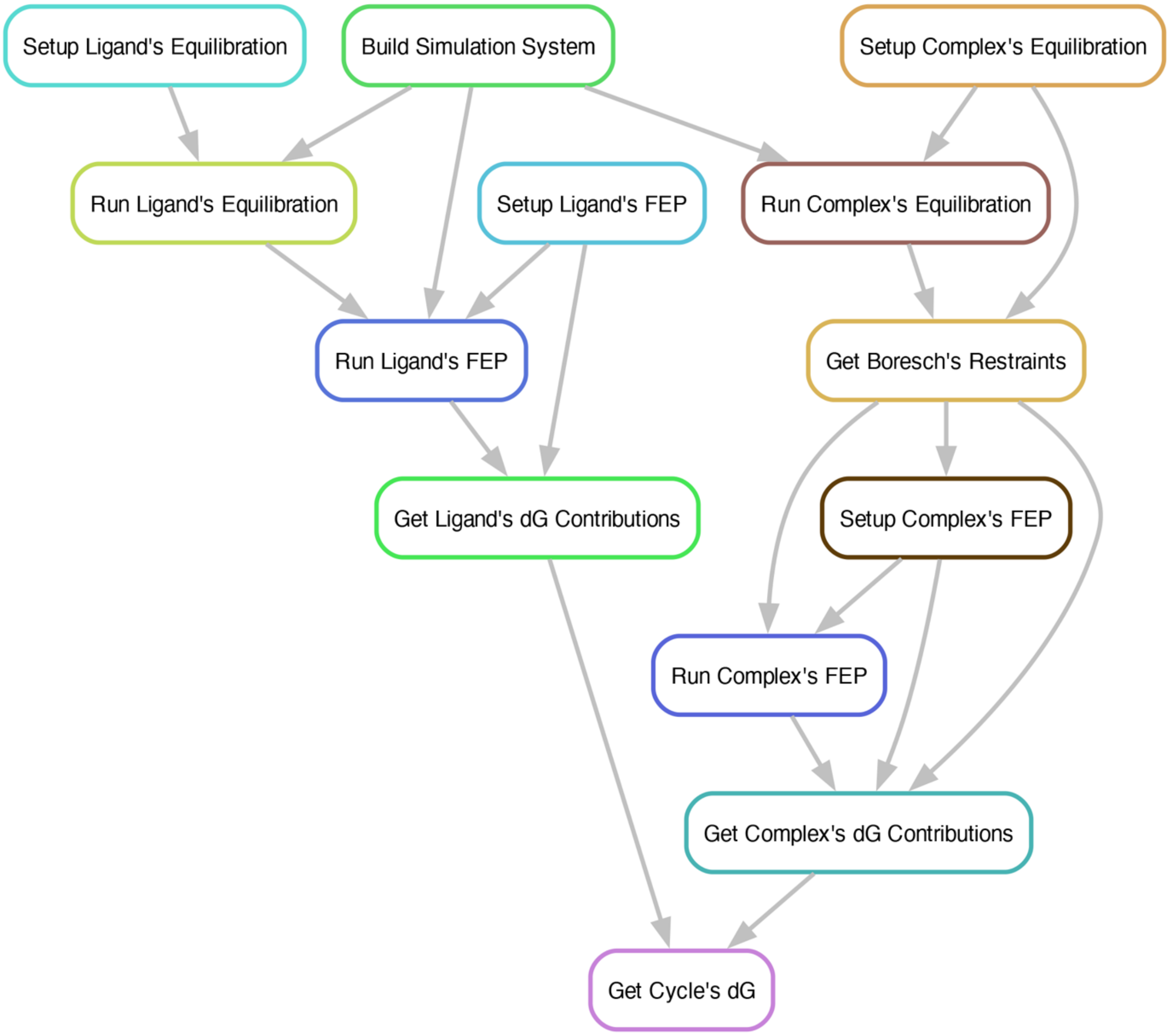

Suplementary Figure 1. Absolute Binding Free Energy (ABFE) calculation pipeline. Each rectangle represents a group of tasks, and the arrows represent the dependencies among these groups. The pipeline starts with the Build Simulation System and Setup steps, where solvated and neutralized systems for the protein-ligand complex and the ligand alone are prepared, and all molecular dynamic parameters (except the complex FEP parameters) are generated. This is followed by the execution of equilibration simulations for both systems. Subsequently, the FEP Setup and Run phase configures and executes free energy perturbation simulations, with Boresch Restraints selected beforehand for the FEP simulations of the complex. The free energy contributions from each perturbed system, calculated at different lambda points, are then computed during the Free Energy Contributions step using MBAR and TI estimators. Finally, results are aggregated in the Cycle ΔG step to calculate the absolute binding free energy (ΔG). Details for each step are provided in the Methods section.

**Table S1. Crystallographic statistics**							
	**F13 WT**				**F13 A295E**		**F13 G277C**
	**APO**	**APO**	**Tecovirimat**	**IMCBH**	**APO**	**Tecovirimat**	**APO**
**PDB code:**	9FHK	9FHS	9HAH	9FJ1	9FIZ	9FJA	9FJ0
**Data collection:**							
Beamline	SOLEIL PX1	SOLEIL PX1	SOLEIL PX1	SOLEIL PX2	SOLEIL PX1	SOLEIL PX1	SOLEIL PX1
Space group	P 1 2_1 1	F 4 3 2	F 4 3 2	F 4 3 2	F 4 3 2	F 4 3 2	F 4 3 2
Unit cell parameters:							
a (Å)	52.35	280.36	281.07	282.80	280.98	281.72	281.05
b (Å)	94.11	280.36	281.07	282.80	280.98	281.72	281.05
c (Å)	73.19	280.36	281.07	282.80	280.98	281.72	281.05
β (°)	98.94						
Resolution (Å)	39.44 – 2.10	49.56 – 2.80	39.36 – 2.60	47.80 – 3.80	49.67 – 2.60	49.80 – 3.50	49.68 – 4.00
Last resolution bin (Å)	2.16 – 2.10	2.95 – 2.80	2.72 – 2.60	4.25 – 3.80	2.72 – 2.60	3.83 – 3.50	4.47 – 4.00
Total observations	287639 (24192)	1272138 (188317)	1255244 (153750)	461213 (131203)	1718736 (212332)	693672 (169435)	657156 (186371)
Unique reflections	40522 (3297)	23822 (3397)	29791 (3565)	10019 (2745)	29770 (3553)	12621 (2936)	8519 (2335)
Completeness (%)	99.1 (99.1)	100 (100)	100 (100)	99.7 (99.0)	100 (100)	100 (100)	100 (100)
Redundancy	7.1 (7.3)	53.4 (55.4)	42.1 (43.1)	46.0 (47.8)	57.7 (59.8)	55.0 (57.7)	77.1 (79.8)
<I/s>	11.3 (1.0)	15.7 (0.3)	19.9 (1.6)	10.1 (1.0)	16.5 (0.2)	8.4 (-0.2)	11.9 (0.2)
R_{sym} (%)	7.3 (168)	23.5 (1438)	20 (336)	56.3 (560)	19.4 (2283)	39.5 (-6275)	27.7 (2510)
$CC_{1/2}$	99.8 (52.2)	99.9 (61.5)	99.9 (51.0)	99.7 (43.0)	99.9 (60.5)	99.9 (22.8)	100 (56.4)
B Wilson (Å^2)	54.21	100.87	65.45	158.11	73.64	117.88	189.54
Resolution with <I/s> = 2 (Å)	2.27	3.06	2.72	4.17	2.85	4.12	4.55
**Refinement:**							
Resolution (Å)	39.44 – 2.10	49.56 – 2.80	39.36 – 2.60	47.80 – 3.80	49.67 – 2.60	49.80 – 3.50	49.68 – 4.00
Last resolution bin (Å)	2.15 – 2.10	2.95 – 2.80	2.68 – 2.60	4.19 – 3.80	2.69 – 2.60	3.85 – 3.50	4.58 – 4.00
Number of reflections	40483 (2693)	22527 (1895)	29784 (2535)	9986 (2264)	28632 (1723)	11873 (2197)	7954 (2096)
Nb of reflections used to calculate Rfree	2009 (166)	1141 (104)	1491 (109)	502 (123)	1396 (102)	589 (137)	401 (102)
B refinement	ISOTROPIC-TLS	ISOTROPIC-TLS	ISOTROPIC-TLS	ISOTROPIC-TLS	ISOTROPIC-TLS	ISOTROPIC-TLS	ISOTROPIC-TLS
Rfactor (%)	21.81 (35.98)	20.34 (78.59)	17.11 (27.38)	20.79 (30.51)	21.59 (83.18)	23.62 (51.20)	23.96 (50.25)
Rfree (%)	24.87 (39.06)	23.19 (85.59)	20.08 (29.11)	24.70 (36.61)	25.86 (73.99)	28.10 (51.87)	27.60 (56.58)
**Number of atoms (Mean B value in A^2):**							
sF13 (A/B)	2764 (65) / 2769 (68)	2884 (94)	2911 (71)	2884 (160)	2888 (88)	2888 (157)	2886 (254)
Citrate	-	13 (112)	13 (92)	13 (213)	13 (125)	-	-
Glycerol	-	72 (120)	54 (100)	12 (159)	78 (116)	18 (169)	6 (218)
Tecovirimat	-	-	27 (75)	-	-	27 (175)	-
IMCBH	-	-	-	20 (156)	-	-	-
Waters	70 (53)	58 (91)	136 (73)	-	59 (88)	14 (93)	-
**Root mean square deviations:**							
Bond lengths (Å)	0.002	0.002	0.018	0.002	0.002	0.044	0.002
Bond angles (°)	0.462	0.463	1.325	0.422	0.484	0.859	0.389
Ramachandran favored/outliers (%)	95.36 / 0.58	94.25 / 0.27	96.44 / 0.55	95.62 / 0.00	95.62 / 0.27	93.42 / 0.55	96.16 / 0.00

Table S2. Lipid composition mimicking the mammalian Golgi membrane.		
**Lipid**	**Structure**	**Conc. (%)**
POPC	16:0 - 18:1	11
PLPC	16:0 - 18:2	14
SAPC	18:0 - 20:4	20
PLPE	16:0 - 18:2	4
PSPE	16:0 - 18:0	8
SAPE	18:0 - 20:4	5
PSPI	16:0 - 18:0	2
POPI	16:0 - 18:1	5
SAPI	18:0 - 20:4	2
POPS	16:0 - 18:1	4
TSM	18:0 - 22:0	12
LPC16	16:0 - 0:0	5
CHOL	Cholesterol	8

Table S3. List of tecovirimat escape mutants.					
	Inhibition assay on MPXV			Mass Photometry assay	
Mutant	IC50 (μM)	Fold change	Reference	EC50 (nM)	Fold change
K174N, N267D	12	720	(1)	-	-
H238Q	0.54–0.6	28–34	(2)	-	-
H238Q, N267D, A295E	24	1,400	(2)	-	-
H238Q, A288P, D294V, I372N	≈5.2	≈290	(1)	-	-
P243S, A288P, A290V	0.56	32	(3)	-	-
T245I, A290V	0.17	10	(1)	-	-
Y258C	18	1,000	(1)	-	-
N267D	10–11	570–630	(2)	-	-
N267D, A288P	1.2–16	71–900	(2, 3)	-	-
N267D, A290V	2.0	110	(1)	-	-
N267D, D294V	12	680	(1)	-	-
N267D, A288P, A290V, D294V	>500	>29,000	(3)	>10,000	>110
N267D, A288P, A290V, A295E,L297ins	>500	>29,000	(3)	-	-
N267D, A288P, A290V, A295E,I372N	>500	>29,000	(1)	-	-
N267del	1.5–4.0	85–230	(4)	>10,000	>110
N267del, A290V	0.13	7.5	(1)	-	-
N267del, N267D, A295E	2.9–18	160–1,000	(1)	-	-
N267del, A288P, A295E	>500	>29,000	(1)	-	-
N267del, T289A, A295E	0.26	15	(1)	-	-
N267del, A290V, I372N	3.1	180	(1)	-	-
N267del, N267D, D294V, A295E	2.5	140	(1)	-	-
G277C	2.3-40	95-800	(5)	334	3.6
D283G	7.1–7.3	404–420	(1)	-	-
Y285H, I372N	0.045	2.6	(1)	-	-
A288P	0.5 to >500	29 to >29,000	(2, 3)	-	-
A288P, I372N	>150	>8,600	(1)	-	-
A288P, A290V, D294V	0.66 to >500	38 to >29,000	(3)	-	-
A288P, A290V, L297ins	>500	>29,000	(3)	-	-
A288P, A290V, I372N	15	880	(1)	-	-
A288P, D294V, A295E	1.4	83	(1)	-	-
A288P, D294V, D301del	>500	>29,000	(3)	-	-
T289A	0.078–0.14	3.7–7.8	(1)	-	-
T289A, R291K	1.7	98	(1)	-	-
A290V	0.17–43	10–2,500	(2, 3)	-	-
A290V, I372N	30–32	1,700–1,800	(2)	-	-
D294V	0.23–1.4	13–78	(2)	-	-
D294V, A295E	1	59	(1)	-	-
A295E	2.0–3.3	110–190	(1)	>10,000	>110
I372N	0.04–>150	2.3 to >8600	(2)	-	-

1. Smith TG, Gigante CM, Wynn NT, Matheny A, Davidson W, Yang Y, et al. Tecovirimat Resistance in Mpox Patients, United States, 2022-2023. Emerg Infect Dis. 2023;29(12):2426-32.
2. Jacob MG, Peera H, Abraar K, Naman KS, Jemma A, Amy NM, et al. Identification of Tecovirimat Resistance-Associated Mutations in Human Monkeypox Virus - Los Angeles County. Antimicrobial Agents and Chemotherapy. 2023;67(7):e00568-23.
3. Alarcón J, Kim M, Terashita D, Davar K, Garrigues JM, Guccione JP, et al. An Mpox-Related Death in the United States. N Engl J Med. 2023;388(13):1246-7.
4. Jacob MG, Peera H, Alex E, Jill KH, Nhien TW, Todd GS, et al. Community spread of a human monkeypox virus variant with a tecovirimat resistance-associated mutation. Antimicrobial Agents and Chemotherapy. 2023;67(11):e00972-23.
5. Duraffour S, Lorenzo MM, Zoller G, Topalis D, Grosenbach D, Hruby DE, et al. ST-246 is a key antiviral to inhibit the viral F13L phospholipase, one of the essential proteins for orthopoxvirus wrapping. J Antimicrob Chemother. 2015;70(5):1367-80.

Table S4. Binding free energy estimation by MBAR and TI with its corresponding energy contributions for all simulations. Units are in kcal/mol.

Pose	Replica	MBAR				TI				Activate Boresch's restraints
		ΔG bind	van der Waals	Coulomb	Release restraint	ΔG bind	van der Waals	Coulomb	Release restraint	
**1**	1	-14,89	-22,542	2,013	-1,276	-14,82	-22,092	1,799	-1,438	6,914
	2	-17,66	-25,127	1,350	-1,116	-17,52	-24,864	1,320	-1,212	7,234
	3	-21,18	-29,620	2,537	-0,848	-21,12	-29,326	2,403	-0,955	6,753
**10**	1	-12,96	-20,602	2,485	-1,432	-12,59	-20,191	2,440	-1,425	6,587
	2	-18,53	-25,882	1,385	-0,742	-18,00	-25,411	1,467	-0,765	6,712
	3	-13,59	-23,426	4,270	-1,202	-12,84	-22,900	4,480	-1,191	6,772
**11**	1	-23,88	-32,042	1,964	-0,455	-22,51	-30,667	2,008	-0,496	6,649
	2	-24,95	-32,246	0,965	-0,474	-24,25	-31,527	0,974	-0,507	6,808
	3	-25,19	-30,307	-0,904	-0,790	-24,79	-30,011	-0,777	-0,815	6,813
**12**	1	-22,45	-28,858	0,795	-1,142	-21,39	-27,893	0,894	-1,148	6,759
	2	-16,54	-25,032	2,554	-0,787	-16,43	-24,869	2,565	-0,847	6,725
	3	-15,09	-23,324	2,349	-0,834	-14,58	-22,804	2,401	-0,897	6,720
**13**	1	-15,12	-24,264	3,304	-1,036	-14,75	-23,782	3,257	-1,100	6,878
	2	-18,25	-26,592	2,926	-1,315	-17,76	-26,234	3,026	-1,286	6,729
	3	-16,75	-25,653	3,478	-1,396	-16,56	-25,390	3,455	-1,444	6,823
**15**	1	-22,60	-29,654	0,971	-0,604	-22,22	-29,257	0,978	-0,633	6,691
	2	-16,34	-22,528	0,185	-0,745	-15,74	-22,052	0,321	-0,761	6,749
	3	-19,25	-24,537	-0,831	-0,642	-18,84	-24,181	-0,759	-0,668	6,764
**16**	1	-21,44	-26,627	-0,533	-0,897	-20,77	-25,962	-0,489	-0,936	6,620
	2	-16,48	-22,323	-0,471	-0,562	-16,18	-22,236	-0,233	-0,592	6,878
	3	-24,54	-30,730	0,055	-0,494	-24,17	-30,363	0,079	-0,520	6,632
**19_3**	1	-16,39	-23,041	0,940	-1,040	-16,24	-22,659	0,976	-1,314	6,753
	2	-14,88	-23,554	3,082	-0,897	-14,65	-23,210	2,990	-0,923	6,489
	3	-16,64	-23,874	1,030	-0,584	-16,38	-23,488	0,927	-0,608	6,793
**20**	1	-12,15	-19,279	1,483	-0,951	-11,96	-19,134	1,511	-0,930	6,594
	2	-19,05	-26,397	1,088	-0,575	-18,74	-26,044	1,069	-0,598	6,835
	3	-18,81	-25,450	0,245	-0,485	-17,94	-24,640	0,412	-0,599	6,885
**20_3**	1	-20,94	-27,894	1,217	-1,007	-20,64	-27,503	1,131	-1,014	6,747
	2	-26,73	-32,534	-0,536	-0,670	-26,24	-32,148	-0,377	-0,721	7,009
	3	-23,19	-29,985	0,341	-0,684	-21,99	-28,745	0,347	-0,731	7,138
**5**	1	-18,24	-25,712	3,056	-2,387	-18,70	-25,397	2,972	-3,081	6,802
	2	-14,86	-23,050	2,557	-0,967	-13,98	-22,260	2,655	-0,974	6,598
	3	-22,11	-29,452	1,610	-1,005	-22,09	-29,313	1,678	-1,192	6,735
**6**	1	-22,74	-28,184	-0,485	-0,663	-22,18	-27,669	-0,414	-0,698	6,596
	2	-18,38	-24,804	0,278	-0,529	-17,95	-24,392	0,332	-0,566	6,677
	3	-25,77	-33,454	1,623	-0,645	-25,53	-33,218	1,647	-0,664	6,700
**6_3**	1	-22,34	-28,062	-0,464	-0,569	-22,34	-28,029	-0,443	-0,625	6,753
	2	-18,73	-28,855	4,516	-1,094	-18,29	-28,489	4,641	-1,152	6,707
	3	-19,70	-27,439	2,493	-1,488	-19,64	-27,113	2,482	-1,744	6,735
**7**	1	-20,93	-27,579	0,259	-0,515	-20,40	-27,329	0,550	-0,520	6,901
	2	-26,94	-33,223	-0,137	-0,520	-26,84	-33,122	-0,106	-0,554	6,944
	3	-19,04	-24,425	0,086	-1,358	-18,18	-23,470	0,117	-1,484	6,656
**9_3**	1	-17,05	-25,236	1,944	-0,568	-16,44	-24,816	2,167	-0,602	6,807
	2	-16,92	-25,526	2,587	-0,713	-16,38	-25,012	2,619	-0,721	6,733
	3	-18,72	-27,425	2,745	-0,768	-18,25	-26,979	2,812	-0,811	6,730

Table S5. Binding free energy estimation by MBAR and TI with its corresponding energy contributions for pose 6-3 on the individual monomers (kcal/mol)										
Pose	Replica	MBAR				TI				Activate Boresch's restraints
		ΔG bind	van der Waal	Coulomb	Release restraint	ΔG bind	van der Waal	Coulomb	Release restraint	
**A**	**1**	-4,36	-11,185	0,936	-0,995	-4,21	-11,064	0,995	-1,027	6,884
	**2**	-5,39	-12,312	1,250	-0,704	-5,12	-11,983	1,236	-0,747	6,373
	**3**	-6,14	-12,200	1,470	-1,853	-6,65	-12,368	1,881	-2,612	6,446
**B**	**1**	-3,62	-8,793	-0,496	-1,047	-2,97	-8,325	-0,305	-1,057	6,719
	**2**	-5,13	-12,671	2,146	-1,323	-5,60	-12,286	2,070	-2,106	6,721
	**3**	-3,15	-8,761	-0,360	-0,726	-3,22	-8,751	-0,356	-0,815	6,697

Table S6. SAXS data collection and scattering derived parameters.	
**Data collection parameters**	
Instrument	Beamline SWING (SOLEIL)
Detector	CCD-based AVIEX
Beam geometry	0.8 mm x 0.15 mm
Wavelength [Å]	1.0
q-range [$Å^{-1}$]	$0.0064 < q < 0.40$
Exposure time [s]	1.5
Temperature [K]	288
**Structural parameters**	
I(0) Guinier [cm^{-1}]	0.039
R_g Guinier [Å][a]	32.1
I(0) *P*(r) [cm^{-1}]	0.039
R_g *P*(r) [Å]	32.9
D_{max} [Å]	108
**Molecular mass determination**	
$MM_{sequence}$[b]	88.6
MM_{SAXS} QR[c]	83.6
MM_{SAXS} QR[d]	76.3

[a] R_g obtained with the Guinier approximation in the range $qR_g < 1.3$; [b] The calculated masses were derived from the sequence ; [c] Molecular mass obtained from the I(q) curve (q_{max} = 0.249 $Å^{-1}$) using the MoW3 program, available at http://saxs.ifsc.usp.br [d] Molecular mass obtained from bayesian interference analysis.

Table S7. SAXS data					
	**R_g** [Å]	***D_{max}*** [Å]	**Chi^2**	**S** [S]	**f/f_0**
**Experimental value**					
sF13 dimer	32.1	108	ND	4.7 ± 0.2	1.45
**Coral models - Calculated values**					
sF13 monomer	21.3	72	234	ND	ND
sF13 dimer	32.3	117	1.58	ND	ND
