# Supplementary material for: Structural insights into tecovirimat antiviral activity and poxvirus resistance
Source: Nat Microbiol. 2025 Feb 12;10(3):734–48. doi: 10.1038/s41564-025-01936-6 (PMC11879855; doi:10.1038/s41564-025-01936-6)
Supplement: Supplementary file 2 — Reporting Summary [file 41564_2025_1936_MOESM2_ESM.pdf]

nature portfolio

Corresponding author(s): Pablo GUARDADO-CALVO

Last updated by author(s): Aug 5, 2024

Reporting Summary

Nature Portfolio wishes to improve the reproducibility of the work that we publish. This form provides structure for consistency and transparency in reporting. For further information on Nature Portfolio policies, see our Editorial Policies and the Editorial Policy Checklist.

Statistics

For all statistical analyses, confirm that the following items are present in the figure legend, table legend, main text, or Methods section.

n/a	Confirmed	
☐	☒	The exact sample size (*n*) for each experimental group/condition, given as a discrete number and unit of measurement
☐	☒	A statement on whether measurements were taken from distinct samples or whether the same sample was measured repeatedly
☐	☒	The statistical test(s) used AND whether they are one- or two-sided<br>*Only common tests should be described solely by name; describe more complex techniques in the Methods section.*
☒	☐	A description of all covariates tested
☐	☒	A description of any assumptions or corrections, such as tests of normality and adjustment for multiple comparisons
☐	☒	A full description of the statistical parameters including central tendency (e.g. means) or other basic estimates (e.g. regression coefficient) AND variation (e.g. standard deviation) or associated estimates of uncertainty (e.g. confidence intervals)
☐	☒	For null hypothesis testing, the test statistic (e.g. *F*, *t*, *r*) with confidence intervals, effect sizes, degrees of freedom and *P* value noted<br>*Give P values as exact values whenever suitable.*
☒	☐	For Bayesian analysis, information on the choice of priors and Markov chain Monte Carlo settings
☒	☐	For hierarchical and complex designs, identification of the appropriate level for tests and full reporting of outcomes
☐	☒	Estimates of effect sizes (e.g. Cohen's *d*, Pearson's *r*), indicating how they were calculated

Our web collection on statistics for biologists contains articles on many of the points above.

Software and code

Policy information about availability of computer code

Data collection	Harmony Software v4.9 (Perkin-Elmer), Acquire MP v2.3 (Refeyn), MXCuBE (version 2). All MD simulations were carried out using publicly available open source code GROMACS, versions 2021 (for dimeric F13 simulation on membrane) and 2022.4 (for F13/tecovirimat simulations). Ligand was fitted into the X-ray density using the open source tools ChimeraX 1.7.1 and Rosetta.
Data analysis	X-ray crystallography: XDS (version January 10, 2022), CCP4 (version 9), PHENIX (version 1.19.2-4158), PYMOL (version 3.0.3) . Mass photometry: DiscoverMP v2.3 (Refeyn). Plotting: Prism v9.0.2 (Graph Pad Software), Adobe Photoshop (v.24.5.0). Analitical ultracentrifugation: SEDNTERP (version 1.9), SEDFIT (version 16.1). Small angle x-ray scattering: ATSAS (version 3.2), GNOM (version 4.6). PLA and IF images: Signals Image Artist v1.3 (Revvity). Sequence analysis: MAFFT (version 7.505), Geneious Prime (version 2024.0.5). MD analysis: were formed using GROMACS 2021 tools and open source python tools such as alchemlyb-2.0.0 and ProLIF. TOFF 0.1.0 (https://zenodo.org/records/8189649). BindFlow has been used for absolute binding free energy calculations available at https://github.com/ale94mleon/BindFlow. A publication describing Bindflow is under preparation, while the code will be released as open source in the next months. Data were plotted using matplotlib.

For manuscripts utilizing custom algorithms or software that are central to the research but not yet described in published literature, software must be made available to editors and reviewers. We strongly encourage code deposition in a community repository (e.g. GitHub). See the Nature Portfolio guidelines for submitting code & software for further information.

Data

Policy information about availability of data

All manuscripts must include a data availability statement. This statement should provide the following information, where applicable:

- Accession codes, unique identifiers, or web links for publicly available datasets
- A description of any restrictions on data availability
- For clinical datasets or third party data, please ensure that the statement adheres to our policy

Atomic coordinates of the reported structures have been deposited in the Protein Data Bank under accession codes 9FHK, 9FHS, 9HAH, 9FJ1, 9FIZ, 9FJA, 9FJ0. All Molecular Dynamic Parameters (MDP), input topologies, coordinates, simulation control files and analysis scripts are provided. Files containing the sampled ΔH and ΔH/Δλ for ABFE are also included. All necessary files to reproduce the molecular dynamic simulations are part of the supporting information and are available at at https://zenodo.org/records/14096216. All the F13 sequences were extracted from the GISAID database at www.gisaid.org.

Research involving human participants, their data, or biological material

Policy information about studies with human participants or human data. See also policy information about sex, gender (identity/presentation), and sexual orientation and race, ethnicity and racism.

Reporting on sex and gender	N/A
Reporting on race, ethnicity, or other socially relevant groupings	N/A
Population characteristics	N/A
Recruitment	N/A
Ethics oversight	N/A

Note that full information on the approval of the study protocol must also be provided in the manuscript.

Field-specific reporting

Please select the one below that is the best fit for your research. If you are not sure, read the appropriate sections before making your selection.

☒ Life sciences ☐ Behavioural & social sciences ☐ Ecological, evolutionary & environmental sciences

For a reference copy of the document with all sections, see nature.com/documents/nr-reporting-summary-flat.pdf

Life sciences study design

All studies must disclose on these points even when the disclosure is negative.

Sample size	MD simulation of membrane patches: Simulation time and replication were large enough to exclude any periodic boundary artifacts or sampling issues or reproducibility issues. PLA: For each condition the experiment was performed twice in triplicates (n=6). Mass photometry: for each condition the experiment was performed in triplicates, each measurements included around 1800 to 4000 particles. Viral inhibition assay: the experiment was performed in quadruplicate. Viral plaque assay: the experiment was perfomed in duplicate and the statistics are based on multiple area measurement within each duplicate.
Data exclusions	No independent experiments were excluded
Replication	All experiments were performed and verified in multiple replicates as indicated in figure legends
Randomization	Sample randomization is not relevant to this study as no populations were investigated. Randomization would not have changed the results. The study is performed under controlled condition, and the reliability stems from the precision and reproducibility of the experiments rather than the use of randomization.
Blinding	Data collection and analysis was not performed blind because it was not needed. The study is performed under controlled condition, using purified molecules, cell lines, and viral strains. The experimental conditions were standardized and reproducible, ensuring that the outcomes were determined by the experimental variables rather than observer input. The data analysis methods, were predefined and computationally driven, eliminating the potential for investigator bias during data interpretation.

Reporting for specific materials, systems and methods

We require information from authors about some types of materials, experimental systems and methods used in many studies. Here, indicate whether each material, system or method listed is relevant to your study. If you are not sure if a list item applies to your research, read the appropriate section before selecting a response.

Materials & experimental systems

n/a	Involved in the study
☐	☒ Antibodies
☐	☒ Eukaryotic cell lines
☒	☐ Palaeontology and archaeology
☒	☐ Animals and other organisms
☒	☐ Clinical data
☒	☐ Dual use research of concern
☒	☐ Plants

Methods

n/a	Involved in the study
☒	☐ ChIP-seq
☒	☐ Flow cytometry
☒	☐ MRI-based neuroimaging

Antibodies

Antibodies used

DuoLink PLA anti-rabbit PLUS probe (cat #DUO92002, Merck) (dil = 1:5)
Duolink PLA anti-mouse MINUS probe (cat #DUO92004, Merck) (dil = 1:5)
Mouse anti-FLAG M2 (cat #F3165, Sigma-Aldrich) (dil = 1:350)
Rabbit anti-FLAG antibody D6W5B (cat #14793, Cell Signaling Technology) (dil = 1:500)
Alexa FluorTM 488 goat anti-mouse antibody (cat #A-11001, Invitrogen) (dil = 1:500)
Alexa FluorTM 488 goat anti-rabbit antibody (cat #A-11008, Invitrogen) (dil = 1:500)

Validation

Validation of all the antibodies has been performed by their providers (Merck, Sigma-Aldrich, Invitrogen and Cell Signaling Technology).
- DuoLink PLA anti-rabbit PLUS probe and Duolink PLA anti-mouse MINUS probe: suitable for Immunofluorescence and proximity ligation assay.
- Mouse anti-FLAG M2: suitable for immunoblotting, immunofluorescence, immunoprecipitation, FACS and ELISA
- Rabbit anti-FLAG antibody D6W5B : Suitable for western blotting, immunoprecipitation, immunoisothcemistry, immunofluorescence, flow cytometry and chropatin immunoprecipitation.
- Alexa FluorTM 488 goat anti-mouse antibody and Alexa FluorTM 488 goat anti-rabbit antibody: suitable for immuno istochemistry, immunocytochemistry, immunofluorescence and flow cytometry.

Eukaryotic cell lines

Policy information about cell lines and Sex and Gender in Research

Cell line source(s)

Hela CCL2 (ATCC), BSC40 (ATCC #CRL-2761) and Vero E6 (ATCC) cells

Authentication

Genotyping (Eurofins)

Mycoplasma contamination

All cell lines were subjected to routine mycoplasma testing (Lonza™ Mycoalert™ Mycoplasma Detection Kit) and were found to be negative

Commonly misidentified lines
(See ICLAC register)

None

Plants

Seed stocks

Report on the source of all seed stocks or other plant material used. If applicable, state the seed stock centre and catalogue number. If plant specimens were collected from the field, describe the collection location, date and sampling procedures.

Novel plant genotypes

Describe the methods by which all novel plant genotypes were produced. This includes those generated by transgenic approaches, gene editing, chemical/radiation-based mutagenesis and hybridization. For transgenic lines, describe the transformation method, the number of independent lines analyzed and the generation upon which experiments were performed. For gene-edited lines, describe the editor used, the endogenous sequence targeted for editing, the targeting guide RNA sequence (if applicable) and how the editor was applied.

Authentication

Describe any authentication procedures for each seed stock used or novel genotype generated. Describe any experiments used to assess the effect of a mutation and, where applicable, how potential secondary effects (e.g. second site T-DNA insertions, mosiacism, off-target gene editing) were examined.
