# Supplementary material for: Structural insights into tecovirimat antiviral activity and poxvirus resistance
Source: Nat Microbiol. 2025 Feb 12;10(3):734–48. doi: 10.1038/s41564-025-01936-6 (PMC11879855; doi:10.1038/s41564-025-01936-6)
Supplement: Supplementary file 4 — GISAID acknowledgment table. [file 41564_2025_1936_MOESM4_ESM.pdf]

We gratefully acknowledge the following Authors from the Originating laboratories responsible for obtaining the specimens, as well as the Submitting laboratories where the genome data were generated and shared via GISAID, on which this research is based.

Ali Submitters of data may be contacted directly via www.gisaid.org

Authors are sorted alphabetically.

Accession ID	Originating Laboratory	Submitting Laboratory	Authors
EPI_ISL_I5969892	Oxford University Clinical Research Unit	Oxford University Clinical Research Unit	Nguyen Thanh Dung, Le Manh Hung, Huynh Thi Thuy Hoa, Tang Chi Thuong, Le Hong Nga, Nguyen Huu Hung, Nghiem My Ngoc, Nguyen Thi Thu Hong, Vo Truong Quy, Vu Thi Kim Thoa, Nguyen Thi Thanh, Phan Vinh Tho, Nguyen Le Nhu Tung, Le Mau Toan, Vo Minh Quang, Dinh Nguyen Huy Man, Nguyên Tân Phât, Trân Th! Lan Phvong, Trân Th! Thanh Tâm, PhQm Th! Ngoc Thoa, Nguyen Hong Tarn, Truong Thi Thanh Lan, Nguyen Thi Han Ny, Tran Tan Thanh, Le Thuy Thuy Khanh, Lam Minh Yen, Guy Thwaites, Louise Thwaites, Nguyen Van Vinh Chau, Nguyen To Anh, Le Van Tan
EPI_ISL_I5972402, EPI_ISL_I5972403, EPI_ISL_I5972404, EPI_ISL_15972406, EPI_ISL_15972407, EPI_ISL_I5972408, EPI_ISL_15972409	Laboratorio Central, Ministerio de Salud Côrdoba	Laboratorio Central, Ministerio de Salud C6rdoba	Castro, G.; Sicilia, P.; Poklepovich, T.; Campos, J.: Barbas, G.
EPI_ISL_I5992095	LESP State of Mexico	Instituto de Diagnostico y Referencia Epidemiologicos (INDRE)	Abril Rodrfguez-Maldonado; Claudia Wong-Arâmbula; Felipe Arguijo-Perez; Helios Cârdenas-Hernândez; Carmen Castro-Méndez: Lidia Garda-Torres; Ruth Madera-Sandoval; América Mandujano-Martfnez: Nancy Martfnez-Velâzquez; Mireya Mederos-Michel; Angélica Pedraza-Meléndez; Joaqufn Quiroz-Mercado; Daniel Regalado-Santiago; Silvia Rivero-Arredondo; Erika Sierra-Atanacio; Fernando Gonzâlez-Domfnguez: Lucia Hernândez-Rivas, Irma L6pez-Martfnez; Ernesto Ramfrez-Gonzâlez; Maribel Gonzâlez-Villa
EPI_ISL_I5992096	LESP Jalisco	Instituto de Diagnostico y Referencia Epidemiologicos (INDRE)	Abril Rodrfguez-Maldonado; Claudia Wong-Arâmbula; Felipe Arguijo-Perez; Helios Cârdenas-Hernândez; Carmen Castro-Méndez; Lidia Garda-Torres; Ruth Madera-Sandoval; América Mandujano-Martfnez; Nancy Martfnez-Velâzquez; Mireya Mederos-Michel; Angélica Pedraza-Meléndez; Joaqufn Quiroz-Mercado; Daniel Regalado-Santiago; Silvia Rivero-Arredondo; Erika Sierra-Atanacio; Fernando Gonzâlez-Domfnguez; Lucia Hernândez-Rivas, Irma L6pez-Martfnez: Ernesto Ramirez-Gonzâlez; Maribel Gonzâlez-Villa
EPI_ISL_I5992097	LESP Morelos	Instituto de Diagnostico y Referencia Epidemiologicos (INDRE)	Abril Rodrfguez-Maldonado: Claudia Wong-Arâmbula; Felipe Arguijo-Perez; Helios Cârdenas-Hernândez; Carmen Castro-Méndez; Lidia Garda-Torres; Ruth Madera-Sandoval; América Mandujano-Martfnez; Nancy Martfnez-Velâzquez; Mireya Mederos-Michel; Angélica Pedraza-Meléndez; Joaqufn Quiroz-Mercado; Daniel Regalado-Santiago; Silvia Rivero-Arredondo; Erika Sierra-Atanacio; Fernando Gonzâlez-Domfnguez; Lucia Hernândez-Rivas, Irma L6pez-Martinez; Ernesto Ramfrez-Gonzâlez; Maribel Gonzâlez-Villa
EPI_ISL_I5992098	LESP Nuevo Lean	Instituto de Diagnostico y Referencia Epidemiologicos (INDRE)	Abril Rodr/guez-Maldonado; Claudia Wong-Arâmbula; Felipe Arguijo-Perez; Helios Cârdenas-Hernândez; Carmen Castro-Méndez; Lidia Garda-Torres; Ruth Madera-Sandoval; América Mandujano-Martfnez; Nancy Martfnez-Velâzquez; Mireya Mederos-Michel; Angélica Pedraza-Meléndez; Joaqufn Quiroz-Mercado; Daniel Regalado-Santiago; Silvia Rivero-Arredondo: Erika Sierra-Atanacio; Fernando Gonzâlez-Domfnguez; Lucia Hernândez-Rivas, Irma L6pez-Martfnez: Ernesto Ramirez-Gonzâlez; Maribel Gonzâlez-Villa
EPI_ISL_I5992099	LESP Hidalgo	Instituto de Diagnostico y Referencia Epidemiologicos (INDRE)	Abril Rodrfguez-Maldonado; Claudia Wong-Arâmbula; Felipe Arguijo-Perez; Helios Cârdenas-Hernândez: Carmen Castro-Méndez; Lidia Garcia-Torres; Ruth Madera-Sandoval; América Mandujano-Martfnez; Nancy Martfnez-Velâzquez; Mireya Mederos-Michel; Angélica Pedraza-Meléndez; Joaqufn Quiroz-Mercado; Daniel Regalado-Santiago: Silvia Rivero-Arredondo: Erika Sierra-Atanacio; Fernando Gonzâlez-Domfnguez; Lucia Hernândez-Rivas. Irma L6pez-Martfnez; Ernesto Ramirez-Gonzâlez; Maribel Gonzâlez-Villa
EPI_ISL_I5992100	LESP Campeche	Instituto de Diagnostico y Referencia Epidemiologicos (INDRE)	Abril Rodrfguez-Maldonado; Claudia Wong-Arâmbula; Felipe Arguijo-Perez; Helios Cârdenas-Hernilndez; Carmen Castro-Méndez; Lidia Garda-Torres; Ruth Madera-Sandoval; América Mandujano-Martfnez; Nancy Martfnez-Velilzquez; Mireya Mederos-Michel; Angélica Pedraza-Meléndez; Joaqufn Quiroz-Mercado; Daniel Regalado-Santiago: Silvia Rivero-Arredondo: Erika Sierra-Atanacio; Fernando Gonzâlez-Domfnguez; Lucia Hernândez-Rivas, Irma L6pez-Martfnez; Ernesto Ramfrez-Gonzâlez; Maribel Gonzâlez-Villa
EPI_ISL_I5992101	LESP Tlaxcala	Instituto de Diagnostico y Referencia Epidemiologicos (INDRE)	Abril Rodrfguez-Maldonado; Claudia Wong-Arâmbula; Felipe Arguijo-Perez; Helios Cârdenas-Hernândez; Carmen Castro-Méndez; Lidia Garda-Torres; Ruth Madera-Sandoval; América Mandujano-Martfnez; Nancy Martfnez-Velâzquez; Mireya Mederos-Michel: Angélica Pedraza-Meléndez; Joaqufn Quiroz-Mercado; Daniel Regalado-Santiago: Silvia Rivero-Arredondo: Erika Sierra-Atanacio; Fernando Gonzâlez-Domfnguez; Lucia Hernândez-Rivas, Irma L6pez-Martfnez; Ernesto Ramfrez-Gonzâlez; Maribel Gonzâlez-Villa
EPI_ISL_I5992102	LESP Aguascalientes	Instituto de Diagnostico y Referencia Epidemiologicos (INDRE)	Abril Rodrfguez-Maldonado; Claudia Wong-Arâmbula; Felipe Arguijo-Perez; Helios Cârdenas-Hernilndez; Carmen Castro-Méndez; Lidia Garda-Torres; Ruth Madera-Sandoval; América Mandujano-Martfnez; Nancy Martfnez-Velilzquez; Mireya Mederos-Michel; Angélica Pedraza-Meléndez; Joaqufn Quiroz-Mercado; Daniel Regalado-Santiago; Silvia Rivero-Arredondo; Erika Sierra-Atanacio; Fernando Gonzâlez-Domfnguez; Lucia Hernândez-Rivas, Irma L6pez-Martfnez; Ernesto Ramfrez-Gonzâlez; Maribel Gonzâlez-Villa
EPI_ISL_15992103, EPI_ISL_15992104	LESP San Luis Potosi	Instituto de Diagnostico y Referencia Epidemiologicos (INDRE)	Abril Rodrfguez-Maldonado; Claudia Wong-Arâmbula; Felipe Arguijo-Perez; Helios Cârdenas-Hernândez; Carmen Castro-Méndez; Lidia Garda-Torres; Ruth Madera-Sandoval; América Mandujano-Martfnez; Nancy Martfnez-Velâzquez; Mireya Mederos-Michel; Angélica Pedraza-Meléndez; Joaqufn Quiroz-Mercado; Daniel Regalado-Santiago; Silvia Rivero-Arredondo; Erika Sierra-Atanacio; Fernando Gonzâlez-Domfnguez; Lucia Hernândez-Rivas, Irma L6pez-Martfnez: Ernesto Ramirez-Gonzâlez; Maribel Gonzâlez-Villa
EPI_ISL_I6006562, EPI_JSL_I6006563	Sequencing/Bioinformatics, Delaware Public Health Lab	Sequencing/Bioinformatics, Delaware Public Health Lab	Bajwa,M.I. and Miller,H.
EPI ISL 16012468, EPI ISL 16012469, EPI ISL 16012470, EPI ISL 16012471, EPI ISL 16012472, EPI ISL 16012473, EPI ISL 16012474, EPI ISL 16012475, EPI ISL 16012476, EPI ISL 16012477, EPI ISL 16012478, EPI ISL 16012479, EPI ISL 16012480, EPI ISL 16012481, EPI ISL 16012482, EPI ISL 16012483, EPI ISL 16012484, EPI ISL 16012485, EPI ISL 16012486, EPI ISL 16012487, EPCISL)6012488. EPI=ISL)6012489, EPÜSL)6012490, EPCISL)6012491, EPI=ISL)6012492, EPCISL)6012493. EPI=ISL)6012494, EPÜSL)6012495. EPCISL)6012496, EPI=ISL)6012497, EPCISL)6012498. EPI=ISL)6012499, EPÜSL)6012500. EPCISL)6012501. EPI=ISL)6012502, EPCISL)6012503. EPI=ISL)6012504, EPÜSL)6012505. EPCISL)6012506, EPI=ISL)6012507, EPI ISL 16012508, EPI ISL 16012509, EPJ ISL 16012510, EPI JSL 16012511, EPI ISL 16012512, EPI ISL 16012513, EPI ISL 16012514, EPI ISL 16012515, EPI ISL 16012516, EPI ISL 16012517, EPI ISL 16012518, EPI ISL 16012519, EPI ISL 16012520, EPI ISL 16012521, EPI ISL 16012522, EPI ISL 16012523, EPI ISL 16012524, EPI ISL 16012525, EPI ISL 16012526, EPI ISL 16012527, EPI=ISL)6012528, EPI=ISL)6012529, EPÜSL)6012530, EPI=ISL)6012531, EPI=ISL)6012532, EPI=ISL)6012533, EPI=ISL)6012534, EPÜSL)6012535, EPI=ISL)6012536			
see above	National Virus Reference Laboratory	National Virus Reference Laboratory	Gabriel Gonzalez, Michael Carr, Brian Keogan, Jose Maria Urtasun Elizari, Jonathan Dean, Daniel Hare, Cillian F De Gascun
EPI_ISL_16031970, EPI_ISL_16031971, EPI_ISL_16031974, EPI_ISL_16031976, EPI_ISL_16031979	Laboratorio de Investigacion Molecular (UNIMOU, Universidad de Cartagena	Laboratorio de Investigacion Molecular (UNIMOU, Universidad de Cartagena	Loyola,S., Fernandez-Ruiz,M., Torres-Pacheco,J., Franco-Munoz,C., Laiton-Donato,K., Ruiz-Moreno,H., Mercado-Reyes,M. and Gomez-Camargo,D.
EPI_ISL_I6074741	Virology Lab, Institute ofTropical Medicine of Sao Paulo, School of Medicine, Universidade de Sao Paulo	Virology Lab	Antonio Charlys da Costa, Maria Cassia Mendes-Correa
EPI_ISL_I6074742	Virology Lab, Institute of Tropical Medicine of Sao Paulo, School of Medicine, Universidade de Sao Paulo	Virology Lab, Institute of Tropical Medicine of Sao Paulo, School of Medicine, Universidade de Sao Paulo	Antonio Charlys da Costa, Maria Cassia Mendes-Correa
EPI_ISL_16080585, EPI_ISL_I6080586, EPI_ISL_16080587, EPI_ISL_16080588, EPI_ISL_I6080589, EPI_ISL_16080590	Institute of Virology, Faculty of Medicine and University Hospital Cologne, University of Cologne, Cologne, Germany	Institute of Virology, Faculty of Medicine and University Hospital Cologne, University of Cologne, Cologne, Germany	Eva Heger, Michael B0hm, Ulrike Wieland, Alexander Kreuter
EPI ISL 16104842, EPI ISL 16104843, EPI ISL 16104844, EPI JSL 16104845, EPI ISL 16104846, EPI ISL 16104847, EPI ISL 16104848, EPI ISL 16104849, EPI ISL 16104850, EPI ISL 16104851. EPI ISL 16104852, EPI ISL 16104853, EPI ISL 16104854, EPI ISL 16104855, EPI ISL 16104856, EPI ISL 16104857, EPI ISL 16104858, EPI ISL 16104859, EPI ISL 16104860, EPI ISL 16104861, EPI-ISL-16104862, EPI-ISL-16104863, EPI-ISL-16104864, EPI-ISL-16104865, EPI-ISL-16104866, EPI-ISL-16104867, EPI-ISL-16104868, EPI-ISL-16104869, EPI-ISL-16104870, EPI-ISL-16104871, EPI-ISL-16104872, EPI-ISL-16104873, EPI-ISL-16104874, EPI-ISL-16104875, EPI-ISL-16104876, EPI-ISL-16104877, EPI-ISL-16104878, EPI-ISL-16104879, EPI-ISL-16104880, EPI-ISL-16104881, EPI-ISL-16104882, EPI-ISL-16104883, EPJ-ISL-16104884, EPI-ISL-16104885, EPI-ISL-16104886, EPI-ISL-16104887, EPI-ISL-16104888, EPI-ISL-16104889, EPI-ISL-16104890, EPI-ISL-16104891, EPI-ISL-16104892, EPI-ISL-16104893, EPI-ISL-16104894, EPI-ISL-16104895, EPI-ISL-16104896, EPI-ISL-16104897, EPI-ISL-16104898, EPI-ISL-16104899, EPI-ISL-16104900, EPI-ISL-16104901, EPI-ISL-16104902, EPI-ISL-16104903, EPJ-ISL-16104904, EPI-ISL-16104905, EPI-ISL-16104906, EPI-ISL-16104907, EPI-ISL-16104908, EPI-ISL-16104909, EPI-ISL-16104910, EPI-ISL-16104911, EPI-ISL-16104912, EPI-ISL-16104913, EPI-ISL-16104914, EPI-ISL-16104915, EPI-ISL-16104916, EPI-ISL-16104917, EPI-ISL-16104918, EPI-ISL-16104919, EPI-ISL-16104920, EPI-ISL-16104921, EPI=ISL)6104922, EPI=ISL)6116727, EPÜSL)6116728, EPI=ISL)6116729, EPI=ISL)6116730, EPI=ISL)6116733, EPI=ISL)6116734, EPÜSL)6116735, EPI=ISL)6116736, EPI=ISL)6116737, EPI=ISL)6116738, EPI=ISL)6116739, EPÜSL)6116740, EPI=ISL)6116741, EPI=ISL)6116742, EPI=ISL)6116743, EPI=ISL)6116744, EPÜSL)6116745, EPI=ISL)6116746, EPI=ISL)6116747, EPI_ISL_I6116748, EPI_ISL_16116749, EPJ_ISL_16116750, EPI_JSL_16116751, EPI_ISL_16116752, EPI_ISL_I6116754, EPI_ISL_16116755, EPI_ISL_16116756, EPI_ISL_16116757, EPI_ISL_16116758, EPI_ISL_I6116759, EPI_ISL_16116760, EPI_ISL_16116761, EPI_ISL_I6116762, EPI_ISL_16116763, EPI_ISL_I6116764, EPI_ISL_16116765			
see above	Laboratorio de Referencia Nacional de Viruas Jnmunoprevenibles. Centro Nacional de Salud Publica. Instiuto Nacional de Salud	Laboratorio de Referencia Nacional de Viruas Inmunoprevenibles. Centro Nacional de Salud Publica. Instiuto Nacional de Salud	Carlos Patricio Padilla Rojas. Carmen Ver6nica Hurtado Vela, Juana Iris Silva Molina. Luis Bârcena Flores, Victor Jiménez Vâsquez, Alicia Elizabeth Nllfiez Llanos. Wendy Lizarraga Olivares, Luren Nieves Sevilla Catafieda. Kelly Vanessa Izarra Rojas, Karla Vasquez Cajachahua, Steve Vladimir Acedo Lazo, Omar Alberto Câceres Rey, Henri Bail6n Calder6n, Priscila Nayu Lope Pari, Nancy Rojas Serrano, Gloria Arotinco Garayar. Equipa de vigilancia genomica del Instituto Nacional de Salud.
EPI ISL 16138916, EPI ISL 16138917, EPI ISL 16138918, EPI ISL 16138919, EPI ISL 16138920, EPI ISL 16138921, EPI ISL 16138922, EPI ISL 16138923, EPI ISL 16138924, EPI ISL 16138925, EPI ISL 16138926, EPI ISL 16138927, EPI ISL 16138928, EPI ISL 16138929, EPI ISL 16138930, EPI ISL 16138933, EPI ISL 16138937, EPI ISL 16138939, EPI ISL 16138940, EPI ISL 16138941, EPCISL)6138944			
see above	California Department of Public Health	California Department of Public Health	Kath, C., Haw, M., Espinosa, A., and Hacker, J.
EPI_ISL_I6183626, EPI_ISL_I6183627	Centers for Disease Control & Prevention (CDC). Division of High Consequence Pathogens and Pathology (DHCPP-PRB)	Centers for Disease Contrai & Prevention (CDC), Division of High Consequence Pathogens and Pathology (DHCPP-PRB)	Gigante,C., Pavlick,J., Zhao,H., Batra,D., Hetrick,E., Howard,□.,Kovar,L., Seabolt.M., Morrison,S, Desch,M., Knipe,K., Weigand,M, Sheth,M., Burgin,A., Burroughs,M., Lee,J., Wilkins,K., McCollum,A., Hutson,C., Davidson,w., Rao,A., Parrott.T. and Li.Y.
EPI_ISL_16183628, EPI_ISL_I6183629, EPI_ISL_16183630	Centers for Disease Control & Prevention (CDC), Division of High Consequence Pathogens and Pathology (DHCPP-PRB)	Centers for Disease Contrai & Prevention (CDC), Division of High Consequence Pathogens and Pathology (DHCPP-PRB)	Gigante,C.. Hottel,W., Zhao,H., Batra,D., Hetrick,E., Howard,D., Kovar,L., Seabolt.M., Morrison,S, Desch,M., Knipe,K., Weigand,M, Sheth,M., Burgin,A., Burroughs,M., Lee,J., Wilkins,K., McCollum,A., Hutson,C., Davidson,W., Rao,A., Nelson,M. and Li,Y.
EPI_ISL_16183631, EPI_ISL_16183632	Centers for Disease Contrai & Prevention (CDC). Division of High Consequence Pathogens and Pathology (DHCPP-PRB)	Centers for Disease Contrai & Prevention (CDC), Division of High Consequence Pathogens and Pathology (DHCPP-PRB)	Gigante,C., Ghinai,I., Zhao,H., Batra,D., Hetrick,E., Howard,D., Kovar,L., Seabolt,M., Morrison,S., Desch,M., Knipe,K., Weigand,M., Sheth,M., Burgin,A., Burroughs,M., Lee.J., Wilkins,K., McCollum.A., Hutson,C., Davidson,W., Rao.A., Kerins,J. and Li,Y.
EPI_ISL_16183633, EPI_ISL_I6183634, EPI_ISL_16183635	Centers for Disease Control & Prevention (CDC), Division of High Consequence Pathogens and Pathology (DHCPP-PRB)	Centers for Disease Contrai & Prevention (CDC), Division of High Consequence Pathogens and Pathology (DHCPP-PRB)	Gigante,C., Pettit,D., Zhao,H., Batra,D., Hetrick,E., Howard,□.,Kovar,L., Seabolt,M., Morrison.S, Desch,M., Knipe,K., Weigand,M., Sheth,M, Burgin,A., Burroughs,M., Lee,J., Wilkins,K., McCollum,A., Hutson,C., Davidson,W., Rao,A., Deutsch-Feldman,M. and Li,Y.
EPI_ISL_I6183636	Centers for Disease Contrai & Prevention (CDC), Division of High Consequence Pathogens and Pathology (DHCPP-PRB)	Centers for Disease Contrai & Prevention (CDC), Division of High Consequence Pathogens and Pathology (DHCPP-PRB)	Gigante,(., Ruiz.V., Zhao,H., Batra,D., Hetrick,E., Howard,D.. Kovar,L., Seabolt.M., Morrison.S, Desch,M., Knipe,K.. Weigand,M, Sheth,M., Burgin,A., Burroughs,M., Lee,J., Wilkins,K., McCollum,A., Hutson,C., Davidson,W., Rao.A., Wang,J. and Li.Y.
EPI_ISL_I6183637	Centers for Disease Contrai & Prevention (CDC), Division of High Consequence Pathogens and Pathology (DHCPP-PRB)	Centers for Disease Contrai & Prevention (CDC), Division of High Consequence Pathogens and Pathology (DHCPP-PRB)	Gigante,C., Cogswell,K., Zhao,H., Batra,D., Hetrick,E., Howard,□.,Kovar,L., Seabolt.M., Morrison,S, Desch,M., Knipe,K., Weigand,M, Sheth,M., Burgin,A., Burroughs,M., Lee,J., Wilkins,K., McCollum,A., Hutson,C., Davidson,W., Rao.A., Grenz,L. and Li,Y.
EPI ISL 16183638, EPI ISL 16183639, EPI ISL 16183640, EPI_ISL_1618364-l, EPI_ISL_1618364-2	Centers for Disease Contrai & Prevention (CDC), Division of High Consequence Pathogens and Pathology (DHCPP-PRB)	Centers for Disease Contrai & Prevention (CDC), Division of High Consequence Pathogens and Pathology (DHCPP-PRB)	Gigante,C., Xia,□.,Zhao,H., Batra,D., Hetrick,E., Howard,D., Kovar,L., Seabolt.M., Morrison,S, Desch,M., Knipe,K.. Weigand,M, Sheth,M.. Burgin,A., Burroughs,M., Lee,J., Wilkins,K., McCollum,A., Hutson,C., Davidson,W., Rao.A., Plipat.N. and li,Y.
EPI_ISL_I6183643	Centers for Disease Contrai & Prevention (CDC). Division of High Consequence Pathogens and	Centers for Disease Contrai & Prevention (CDC), Division of High Consequence Pathogens and Pathology	Gigante,C., Epie,N., Zhao,H., Batra,D., Hetrick,E., Howard,D., Kovar,L., Seabolt,M., Morrison,S., Desch,M., Knipe,K., Weigand,M , Sheth,M., Burgin,A., Burroughs,M., Lee,J., Wilkins,K., McCollum,A., Hutson,C., Davidson,w., Rao.A., Perez,T. and Li,Y.

	Pathology (DHCPP-PRB)	(DHCPP-PRB)	
EPI_ISL_l6183644, EPI_ISL_l6183645	Centers for Disease Contrai & Prevention (CDC), Division of High Consequence Pathogens and Pathology (DHCPP-PRB)	Centers for Disease Contrai & Prevention (CDC), Division of High Consequence Pathogens and Pathology (DHCPP-PRB)	Gigante,C.. Thomas,L., Zhao,H.. Batra,D., Hetrick,E., Howard,D.. Kovar,L.. Seabolt,M., Morrison,S.. Desch,M., Knipe,K., Weigand,M, Sheth,M., Burgin,A.. Burroughs,M., Lee,J., Wilkins,K., McCollum,A., Hutson,C.. Davidson,W., Rao.A., Dunn,J. and Li,Y.
EPI_ISL_l6183646, EPI_ISL_l6183647, EPI_ISL_l6183648	Centers for Disease Contrai & Prevention (CDC), Division of High Consequence Pathogens and Pathology (DHCPP-PRB)	Centers for Disease Contrai & Prevention (CDC), Division of High Consequence Pathogens and Pathology (DHCPP-PRB)	Gigante,C., Kubin,G., Zhao,H., Batra,D., Hetrick,E., Howard,□.,Kovar,L., Seabolt.M., Morrison,S., Desch,M., Knipe,K., Sheth,M.R., Burgin,A., Burraughs,M., Lee,J., Wilkins,K., McCollum,A., Hutson,C., Davidson,W., Rao,A., White,S. and li,Y.
EPI_ISL_l6183649, EPI_ISL_l6183650	Centers for Disease Contrai & Prevention (CDC), Division of High Consequence Pathogens and Pathology (DHCPP-PRB)	Centers for Disease Contrai & Prevention (CDC), Division of High Consequence Pathogens and Pathology (DHCPP-PRB)	Gigante,C.. Mooring,E.. Zhao,H.. Batra,D.. Hetrick,E.. Howard,□.,Kovar,L., Seabolt,M., Morrison,S., Desch,M., Knipe,K., Weigand,M, Sheth,M., Burgin,A.. Burroughs,M.. Lee,J.. Wilkins,K.. McCollum,A.. Hutson,C.. Davidson,W.. Rao.A.. Laurence,J. and Li,Y.
EPI_ISL_l6183651	Centers for Disease Contrai & Prevention (CDC). Division of High Consequence Pathogens and Pathology (DHCPP-PRB)	Centers for Disease Contrai & Prevention (CDC), Division of High Consequence Pathogens and Pathology (DHCPP-PRB)	Gigante,C., Francis,□., Zhao,H.. Batra,D., Hetrick.E., Howard,□.,Kovar.L.. Seabolt.M., Morrison,S., Desch,M.. Knipe,K.. Weigand,M . Sheth.M., Burgin,A., Burroughs,M., Lee.J.. Wilkins,K.. McCollum,A., Hutson,C.. Davidson,W.. Rao.A.. Escobar,). and Li.Y.
EPJ_ISL_16183652, EPI_JSL_16183653	Centers for Disease Contrai & Prevention (CDC), Division of High Consequence Pathogens and Pathology (DHCPP-PRB)	Centers for Disease Contrai & Prevention (CDC), Division of High Consequence Pathogens and Pathology (DHCPP-PRB)	Gigante,C., Ventura,J., Zhao,H., Batra,D., Hetrick,E., Howard,□.,Kovar,L., Seabolt.M., Morrison,S, Desch,M., Knipe,K., Weigand,M, Sheth,M., Burgin,A., Burroughs,M., Lee,J., Wilkins,K., McCollum,A., Hutson,C., Davidson,W., Rao,A., Nash,J. and Li,Y.
EPI_ISL_l6183654, EPI_ISL_l6183655	Centers for Disease Contrai & Prevention (CDC), Division of High Consequence Pathogens and Pathology (DHCPP-PRB)	Centers for Disease Contrai & Prevention (CDC), Division of High Consequence Pathogens and Pathology (DHCPP-PRB)	Gigante,C.. Lee,P.. Zhao,H.. Batra,D.. Hetrick,E., Howard,D.. Kovar,L.. Seabolt,M., Morrison,S.. Desch,M.. Knipe,K.. Weigand,M , Sheth,M.. Burgin,A.. Burraughs,M.. Lee,J.. Wilkins,K.. McCollum,A., Hutson,C.. Davidson,W.. Rao.A.. Stanek,D. and li.Y.
EPI ISL 16190089, EPI ISL 16190090, EPI ISL 16190092, - - EPI_ISL_l619009-4, EPI_ISL_l619oo99	Rush University Medical Center	RIPHL at Rush University Medical Center	Stefan Green, Kevin Kunstman, Hannah Barbian, Felix Araujo Perez, Edith Perez, Sofiya Bobravska, Alyse Kittner, Cecilia Chau, Giancarlo Balangue, Lok Yiu Ashley Wu, Mary Hayden, Joyce Houlihan, Diane Springer, Nicholas Moore
EPI_ISL_l6222568, EPI_ISL_l6222569	Centers for Disease Contrai & Prevention (CDC), Division of High Consequence Pathogens and Pathology (DHCPP-PRB)	Centers for Disease Contrai & Prevention (CDC), Division of High Consequence Pathogens and Pathology (DHCPP-PRB)	Gigante,C., Ruiz.V., Zhao,H., Batra.D.. Hetrick,E., Howard,□.,Kovar,L., Seabolt.M., Morrison,S , Desch,M., Knipe,K., Weigand,M . Sheth,M., Burgin,A.. Burroughs,M., Lee,J., Wilkins.K., McCollum,A., Hutson,C., Davidson,W., Rao.A., Wang,J. and Li,Y.
EPI_ISL_l6222570	Centers for Disease Contrai & Prevention (CDC). Division of High Consequence Pathogens and Pathology (DHCPP-PRB)	Centers for Disease Contrai & Prevention (CDC), Division of High Consequence Pathogens and Pathology (DHCPP-PRB)	Gigante,C., Hauser,)., Zhao,H., Batra,D., Hetrick,E., Howard,□., Kovar,L., Seabolt,M., Morrison,S., Desch,M., Knipe,K., Weigand,M, Sheth,M., Burgin,A., Burroughs,M., Lee,J., Wilkins,K., McCollum,A., Hutson,C., Davidson,W., Rao.A., Mangla,A. and Li,Y.
EPI_ISL_l6222571	Centers for Disease Contrai & Prevention (CDC). Division of High Consequence Pathogens and Pathology (DHCPP-PRB)	Centers for Disease Contrai & Prevention (CDC), Division of High Consequence Pathogens and Pathology (DHCPP-PRB)	Gigante,C., Lee,P.. Zhao.H., Batra,D.. Hetrick,E., Howard.D.. Kovar,L., Seabolt,M., Morrison,S., Desch,M., Knipe,K.. Weigand.M., Sheth,M., Burgin,A., Burraughs,M.. Lee,J., Wilkins,K., McCollum,A., Hutson,C., Davidson.W., Rao.A., Stanek,D. and li.Y.
EPI_ISL_l6222572, EPI_JSL_l6222573	Centers for Disease Contrai & Prevention (CDC). Division of High Consequence Pathogens and Pathology (DHCPP-PRB)	Centers for Disease Contrai & Prevention (CDC), Division of High Consequence Pathogens and Pathology (DHCPP-PRB)	Gigante,C., Ghinai,I., Zhao,H., Batra,D., Hetrick,E., Howard,□., Kovar,L., Seabolt,M., Morrison,S., Desch,M., Knipe,K., Weigand,M , Sheth,M., Burgin,A., Burroughs,M., Lee,J., Wilkins,K., McCollum.A., Hutson,C., Davidson,w., Rao.A., Kerins.J. and Li,Y.
EPI_ISL_l6233781	Complejo Hospitalario Universitario de Pontevedra	Microbiology Department. Complexa Hospitalario Universitario de Vigo	Daviria C, Pizcueta J, Trigo M, Perez-Castro S
EPI ISL 16233782, EPI ISL 16233783, EPI ISL 16233784, EPI=ISL)6233785, EPI=ISL)6233786, EPI=ISL)6233787, EPI_ISL_l6233788	Microbiology Department. Complexa Hospitalario Universitario de Vigo	Microbiology Department. Complexa Hospitalario Universitario de Vigo	Daviria C, Pizcueta J, Perez-Castro S
EPI_ISL_l62603Sl	Centre Médical de l'Institut Pasteur	Cellule d'intervention Biologique d'Urgence, Institut Pasteur	Charlotte Salière. Véronique Hourdel, Aurelia Kwasiborski. Quentin Grassin, Maxence Feher, Damien Hoinard, Jessica Vanhomwegen, Fabien Taieb, Paul-Henri Consigny, Jean-Claude Manuguerra, lndia Leclercq, Christophe Batéjat, Valérie Caro
EPI_ISL_l6260402	Cellule d'intervention Biologique d'Urgence, Institut Pasteur	Cellule d'intervention Biologique d'Urgence, Institut Pasteur	Charlotte Salière, Véronique Hourdel, Aurelia Kwasiborski, Quentin Grassin, Maxence Feher, Damien Hoinard, Jessica Vanhomwegen, Fabien Taieb, Paul-Henri Consigny, Jean-Claude Manuguerra, lndia Leclercq, Christophe Batéjat, Valérie Caro
EPJ_ISL_l6299702, EPI_JSL_l6299718	Azienda Sanitaria dell'Alto Adige - Laboratorio Aziendale di Micrabiologia e Viralogia	Azienda Sanitaria dell'Alto Adige - Laboratorio Aziendale di Micrabiologia e Viralogia	Teresa Fortini, Elisabetta lncrocci, Elisabetta Giacobazzi, Elisa Masi, Irene Bianconi, Elisabetta Pagani
EPI_ISL_l6350819	Laboratorio de Biologia Molecular y Biotecnologia / Facultad de ciencias de la salud, Universidad Tecnologica de Pereira	Laboratorio de Biologia Molecular y Biotecnologia / Facultad de ciencias de la salud, Universidad Tecnologica de Pereira	Orjuela,M., Tabares,F.A., Anacona,J.D., Lopez,P.A., Zuluaga-Velez,A. and Sepulveda-Arias,J.C.
EPI_ISL_l6350820	Laboratorio de Biologia Molecular y Biotecnologia / Facultad de ciencias de la salud, Universidad Tecnologica de Pereira	Laboratorio de Biologia Molecular y Biotecnologia / Facultad de ciencias de la salud, Universidad Tecnologica de Pereira	Tabares,F.A., Anacona.J.D., Lopez.P.A., Orjuela.M., Zuluaga-Velez,A. and Sepulveda-Arias,J.C.
EPI ISL 16360909, EPI ISL 16360911, EPI ISL 16360913, EPI ISL 16360915, EPI ISL 16360917, EPI ISL 16360919, EPI ISL 16360921, EPI ISL 16360922, EPI ISL 16360924, EPI ISL 16360927, EPI ISL 16360929, EPI ISL 16360931, EPI ISL 16360932, EPI ISL 16360935, EPI ISL 16360937, EPI ISL 16360939, EPI ISL 16360941, EPI ISL 16360943, EPI ISL 16360946, EPI ISL 16360948, EPCISL)6360950. EPI=ISL)6360952, EPÜSL)6360954, EPCISL)6360955, EPI=ISL)6360957, EPCISL)6360959. EPI=ISL)6360961, EPÜSL)6360963. EPCISL)636096 5, EPI=ISL)6360967, EPCISL)6360969 - - - - - - - - - - - - - - - - - -			
see above	Centre for Biological Threats, Highly Pathogenic Viruses, Robert Koch Institute	Centre for Biological Threats, Highly Pathogenic Viruses, Robert Koch Institute	Brinkmann,A.. Kohl,C., Pape,K., Schrick,L., Michel,)., Schaade,L. and Nitsche,A.
EPI_ISL_l6467111	IRCCS Sacra Cuore Don Calabria Hospital, Department of Infectious, Tropical Diseases & Micrabiology	IRCCS Sacra Cuore Don Calabria Hospital, Department of Infectious, Tropical Diseases & Microbiology	Michela Deiana. Denise Lavezzari. Silvia Accordini, Concetta Castilletti, Antonio Mari, Elena Pomari, Chiara Piubelli
EPI_ISL_l6505425	Hopital Saint Louis	Hopital Saint Louis	Zeggagh,J., Ferraris,O., Salmona,M., Tarantola,A., Molina,J.M. and Delaugerre,C.
EPI ISL 16510131, EPI ISL 16510132, EPI ISL 16510134, EPI JSL 16510136, EPI ISL 16510138, EPI ISL 16510140, EPI ISL 16510141. EPI ISL 16510143, EPI ISL 16510145, EPI ISL 16510147, EPI ISL 16510148, EPI ISL 16510150, EPI ISL 16510153, EPI ISL 16510154, EPI ISL 16510155, EPI ISL 16510156, EPI ISL 16510157, EPI ISL 16510158, EPI ISL 16510159, EPI ISL 16510162, EPI=ISL)6510163, EPI=ISL)6510164, EPÜSL)6510165, EPI=ISL)6510166, EPI=ISL)6510167, EPI=ISL)6510168, EPI=ISL)6510169, EPÜSL)6510170, EPI=ISL)6510171, EPI=ISL)6510172, EPI=ISL)6510173, EPI=ISL)6510174, EPÜSL)6510175, EPI=ISL)6510177, EPI=ISL)6510178, EPI=ISL)6510179, EPI=ISL)6510180, EPÜSL)6510181, EPI=ISL)6510182, EPI=ISL)6510183, EPI_ISL_l6510184, EPI_ISL_16510185			
see above	National Virus Reference Laboratory	National Virus Reference Laboratory	Gabriel Gonzalez, Michael Carr, Brian Keogan, Jose Maria Urtasun Elizari, Jonathan Dean, Daniel Hare. Cillian F De Gascun
EPI_ISL_16526309	Direccion de Investigacion en Salud Publica, Instituto Nacional de Salud	Direccion de Investigacion en Salud Publica, Instituto Nacional de Salud	Laiton-Donato,K.D., Franco,C.E., Alvarez-Diaz,D.A., Ruiz-Moreno,H.A., Prada,0.A., Rosales,A. and Mercado-Reyes,M.M.
EPI_ISL_16588110, EPI_ISL_16588825, EPI_ISL_16589442	Erasmus Medical Center Department of Viralogy	Erasmus Medical Center Department of Viralogy	Leonard Schuele, Bas Oude Munnink, Marjan Soter, Babette Weller, Babs Verstrepen, Richard Molenkamp, Janette Rahamat-Langendoen, Reina Sikkema, Marion Koopmans
EPI_ISL_l6645184	Microbiology Service of University Hospital of A Coruna (SERGAS)	Microbiology, Instituto de Investigacion Biomedica de A Corufia (INIBIC)	Macaya,P., Rumbo-Feal,S., Poza,M., Canizares,A., Vallejo,J.A. and Bou,G.
EPI_ISL_16645185	Micrabiology Service of University Hospital of A Coruna (SERGAS	Micrabiology, Instituto de Investigacion Biomedica de A Coruna (INIBIC)	Macaya,P.. Rumbo-Feal,S., Poza,M., Canizares,A., Vallejo,J.A. and Bou,G.
EPI_ISL_l6645186, EPI_ISL_16645187, EPJ_ISL_16645188. EPI_JSL_l6645189, EPI_ISL_16645190, EPI_ISL_16645192. EPI_ISL_16645193, EPI_ISL_16645194. EPI_ISL_l6645195, EPI_ISL_l6645196, EPI_ISL_l6645197. EPI_ISL_16645198, EPI_ISL_16645199. EPI_ISL_l6645200, EPI_ISL_16645201, EPI_ISL_l6645202, EPI_ISL_16645203, EPI_ISL_16645204. EPI_ISL_16645205			
see above	Oepartamento de Genetica, Instituto de Biologia, Universidade federal do Rio de Janeiro	Oepartamento de Genetica, Instituto de Biologia, Universidade federal do Rio de Janeiro	Nunes,O.S., Higa,L.M., Oliveira,R.L., Costa,L.C., Bomfim,L.M., Goncalves,C.C.A., Mariane,□.,Hruby,O.E.. Voloch,C.M., Castineiras,T.M.P.P., Tanuri,A. and Oamaso,C.R
EPI_ISL_l6645206	Division of High-risk Pathogens. Korea Disease Contrai and Prevention Agency	Division of High-risk Pathogens, Korea Disease Contrai and Prevention Agency	Rhie.G.-e.
EPI_ISL_l6645207, EPI_ISL_16645208, EPI_ISL_16645209, EPI_ISL_l6645211, EPI_ISL_16645212, EPI_ISL_l6645213, EPI_ISL_16645214, EPI_ISL_16645215, EPI_ISL_l6645216, EPI_ISL_16645218, EPI_ISL_l6645219, EPI_ISL_16645220, EPI_ISL_16645221, EPI_ISL_l6645223, EPI_ISL_16645224, EPI_ISL_l6645226, EPI_ISL_16645227, EPI_ISL_16645228			
see above	Antioquia, Laboratorio Departamental de Salud Publica de Antioquia	Antioquia. laboratorio Departamental de Salud Publica de Antioquia	Betancur.I.I.B., Velarde-Hoyos,C.-A.C.V., Gomez,R.R.G. and Mercado-Reyes,M.M.R.
EPI_ISL_l6645229	Environment and Infectious Risks Unit, Insitut Pasteur	Environment and Infectious Risks Unit, Insitut Pasteur	Baliere,C., Hourdel.V., Kwasiborski,A., Grassin,Q.. Feher,M., Hoinard,D., Vanhomwegen,J., Taieb,F., Consigny,P.-H.. Manuguerra,J.-C., Leclercq,I., Batejat,C. and Caro,V.
EPI ISL 16650246, EPI ISL 16650247, EPI ISL 16650248, EPLISL 16650249, EPI ISL 16650251, EPI ISL 16650260	Laboratorio Central de Saude Publica do Estado de Minas Gerais (Lacen-MG)	Laboratorio Central de Saude Publica do Estado de Minas Gerais {Lacen-MG)	Felipe Campos de Melo lani, Ludmila Oliveira Lamounier, Luiz Marcelo Ribeiro Tomé, Natália Rocha Guimaràes,Talita Emile Ribeiro Adelina.
EPI_ISL_l6650297, EPI_ISL_l6650298, EPI_ISL_l6650299, EPI_ISL_l6650300, EPI_ISL_l6650301, EPI_ISL_l6650302, EPI_ISL_l6650303, EPI_ISL_l6650304, EPI_ISL_l6650305, EPI_ISL_l6650307, EPI_ISL_l6650309, EPI_ISL_l6650311			
see above	Centre for Biological Threats, Highly Pathogenic Viruses, Robert Koch Institute	Centre for Biological Threats, Highly Pathogenic Viruses, Robert Koch Institute	Brinkmann,A., Kohl,C., Pape,K., Schrick,L., Michel,)., Schaade,L. and Nitsche,A.
EPI_ISL_l6679203, EPI_ISL_l6679204, EPI_ISL_l6679206, EPI_ISL_16679207, EPI_ISL_16679208	Los Angeles County Public Health Laboratories	Los Angeles County Public Health Laboratories	P. Hemarajata et al.
EPI_ISL_l6679209, EPI_ISL_l6679210, EPJ_ISL_l6679212, EPI_JSL_l6679213, EPI_ISL_l6679214, EPI_ISL_l6679215, EPI_ISL_l6679216, EPI_ISL_l6679217, EPI_ISL_l6679219, EPI_ISL_l6679220, EPI_ISL_l6679221, EPI_ISL_l6679222, EPI_ISL_l6679223, EPI_ISL_l6679224, EPI_ISL_l6679225, EPI_ISL_l6679226. EPI_ISL_l6679227, EPI_ISL_l6679229, EPI_ISL_l6679230			
see above	Kaiser Permanente Chino Hills Regional Reference Laboratories	Los Angeles County Public Health Laboratories	P. Hemarajata et al.
EPI_ISL_l6679232	Los Angeles County Public Health Laboratories	Los Angeles County Public Health Laboratories	P. Hemarajata et al.
EPI_ISL_16679235	Kaiser Permanente Chino Hills Regional Reference Laboratories	Los Angeles County Public Health Laboratories	P. Hemarajata et al.

EPI ISL 16679236, EPI ISL 16679237, EPI ISL 16679238, EPI ISL 16679240	Los Angeles County Public Health Laboratories	Los Angeles County Public Health Laboratories	P. Hemarajata et al.
EPI ISL 16679242, EPI ISL 16679243, EPI ISL 16679244, EPI ISL 16679245, EPI ISL 16679246, EPI ISL 16679247, EPI ISL 16679248, EPI ISL 16679249, EPI ISL 16679250, EPI ISL 16679251, EPI ISL 16679252, EPI ISL 16679253, EPI ISL 16679254, EPI ISL 16679255, EPI ISL 16679256, EPI ISL 16679257, EPI ISL 16679258, EPI ISL 16679259, EPI ISL 16679260, EPI ISL 16679261, EPI-ISL-16679262, EPI-ISL-16679263, EPJ-ISL-16679264, EPI-JSL-16679265, EPI-ISL-16679266, EPI-ISL-16679267, EPI-ISL-16679268, EPI-ISL-16679269, EPI-ISL-16679270, EPI-ISL-16679271, EPI-ISL-16679272, EPI-ISL-16679273, EPI-ISL-16679274, EPI-ISL-16679275, EPI-ISL-16679276, EPI-ISL-16679277, EPI-ISL-16679278, EPI-ISL-16679279, EPI-ISL-16679280, EPI-ISL-16679281, EPI-ISL-16679282, EPI-ISL-16679283, EPI-ISL-16679284, EPI-ISL-16679285, EPI-ISL-16679286, EPI-ISL-16679287, EPI-ISL-16679288, EPI-ISL-16679289, EPI-ISL-16679290, EPI-ISL-16679291, EPI-ISL-16679292, EPI-ISL-16679293, EPI-ISL-16679294, EPI-ISL-16679295, EPI-ISL-16679296, EPI-ISL-16679297, EPI-ISL-16679298, EPI-ISL-16679299, EPI-ISL-16679300, EPI-ISL-16679301, EPI=ISL)6679302, EPI=ISL)6679303, EPÜSL)6679304			
seeabove	California Department of Public Health	California Department of Public Health	Probert,W., Espinosa,A., Kath,C., Haw,M., O'Neil,R., Bell,]. and Hacker,J.
EPI_ISL_16727186, EPI_ISL_16727549	Erasmus Medical Center, Department of Virology	Erasmus Medical Center Department of Virology	Leonard Schuele, Bas Oude Munnink, Marjan Boter, Babette Weller, Babs Verstrepen, Richard Molenkamp, Janette Rahamat-Langendoen, Reina Sikkema, Marion Koopmans
EPI_ISL_16751080, EPI_ISL_16751081, EPI_ISL_16751082, EPI_ISL_16751083, EPI_ISL_16751084, EPI_ISL_16751085, EPI_ISL_16751086, EPI_ISL_16751087, EPI_ISL_16751088, EPI_ISL_16751089, EPI_ISL_16751090, EPI_ISL_16751091, EPI_ISL_16751092			
see above	Centre for Biological Threats, Highly Pathogenic Viruses, Robert Koch Institute	Centre for Biological Threats, Highly Pathogenic Viruses, Robert Koch Institute	Brinkmann,A., Kohl,C., Pape,K., Schrick,L., Michel,]., Schaade,L. and Nitsche.A.
EPI_ISL_16751093, EPI_ISL_16751094, EPI_ISL_16751095, EPI_ISL_16751096	Centers for Disease Contrai & Prevention (CDC), Division of High Consequence Pathogens and Pathology (DHCPP-PRB)	Centers for Disease Contrai & Prevention (CDC), Division of High Consequence Pathogens and Pathology (DHCPP-PRB)	Gigante,C., Kubin,G., Zhao,H., Batra,D., Hetrick,E., Howard,D., Kovar,L., Seabolt,M., Morrison,S., Desch,M., Knipe,K.. Sheth,M.R., Burgin,A., Burroughs,M., Lee,J, Wilkins,K.. McCollum,A., Hutson,C., Davidson,W.. Rao,A.. White,S. and li,Y.
EPJ_ISL_l6751097, EPI_JSL_16751098	Centers for Disease Contrai & Prevention (CDC), Division of High Consequence Pathogens and Pathology (DHCPP-PRB)	Centers for Disease Contrai & Prevention (CDCJ, Division of High Consequence Pathogens and Pathology (DHCPP-PRB)	Gigante,C., Kubin,G., Zhao,H., Batra,D., Hetrick,E., Howard,D., Kovar,L., Seabolt,M., Morrison,S., Desch,M., Knipe,K., Weigand,M., Sheth,M., Burgin,A., Burroughs,M., Lee,J., Wilkins,K., McCollum,A., Hutson,C., Davidson,w., Rao.A., White,S. and li,Y.
EPI_ISL_16751099, EPI_ISL_16751100	Centers for Disease Contrai & Prevention (CDC), Division of High Consequence Pathogens and Pathology (DHCPP-PRB)	Centers for Disease Contrai & Prevention (CDC), Division of High Consequence Pathogens and Pathology (DHCPP-PRB)	Gigante,C., Murray,]., Zhao,H., Batra,D., Hetrick,E., Howard,D., Kovar,L., Seabolt,M., Morrison,S., Desch,M.. Knipe,K., Weigand,M., Sheth,M., Burroughs,A.B., Lee,J., Wilkins,K., McCollum,A., Hutson,C., Davidson,W., Rao.A., Atkinson,A. and Li.Y.
EPI ISL 16751101, EPI ISL 16751102, EPI ISL 16751103, EPI=ISL)6751104, EPI=ISL)6751105, EPCISL)6751106, EPI_ISL_16751107	Centers for Disease Contrai & Prevention (CDC), Division of High Consequence Pathogens and Pathology (DHCPP-PRB)	Centers for Disease Contrai & Prevention (CDC), Division of High Consequence Pathogens and Pathology (DHCPP-PRB)	Gigante,C., Hauser,]., Zhao,H., Batra,D, Hetrick,E, Howard,D, Kovar,L., Seabolt,M, Morrison,S., Desch,M., Knipe,K., Weigand,M., Sheth,M, Burgin,A., Burroughs,M., Lee,J., Wilkins,K., McCollum,A, Hutson,C., Davidson,W., Rao.A., Mangla,A. and Li.Y.
EPI ISL 16751108, EPI ISL 16751109, EPI ISL 16751110, EPI ISL l6751111	Centers for Disease Contrai & Prevention (CDC), Division of High Consequence Pathogens and Pathology (DHCPP-PRB)	Centers for Disease Contrai & Prevention (CDC), Division of High Consequence Pathogens and Pathology (DHCPP-PRB)	Gigante,(., Bradley.A., Zhao,H., Batra,D., Hetrick,E, Howard,D, Kovar,L., Seabolt.M, Morrison,S.. Desch,M., Knipe,K.. Sheth,M.R., Burgin,A., Burroughs,M.. Lee,J.. Wilkins,K.. McCollum,A,. Hutson,C, Davidson,W, Rao.A.. Anderson,], and Li.Y.
EPI_ISL_l6751112	Centers for Disease Contrai & Prevention (CDC), Division of High Consequence Pathogens and Pathology (DHCPP-PRB)	Centers for Disease Contrai & Prevention (CDC), Division of High Consequence Pathogens and Pathology (DHCPP-PRB)	Gigante,C., Johnson,S., Zhao,H., Batra,D., Hetrick,E., Howard,D., Kovar,L., Seabolt,M., Morrison,S., Weigand,M., Knipe,K., Sheth,M, Burgin,A, Burraughs,M., Lee,J, Wilkins,K., McCollum,A., Hutson,C., Davidson,W., Rao.A., Riner,D. and Li.Y.
EPI_ISL_16751113	Centers for Disease Contrai & Prevention (CDC), Division of High Consequence Pathogens and Pathology (DHCPP-PRB)	Centers for Disease Contrai & Prevention (CDCJ, Division of High Consequence Pathogens and Pathology (DHCPP-PRB)	Gigante,C., Cleavinger,K. Zhao,H, Batra,D., Hetrick,E., Howard,D, Kovar,L., Seabolt.M., Morrison,S., Desch,M, Knipe,K., Burroughs,M.R., Lee,J., Wilkins,K., McCollum,A., Hutson,C, Davidson,W., Rao.A., Sinn,M, and Li,Y.
EP1_ISl_16751114, EPI_ISL_16751115	Centers for Disease Contrai & Prevention (CDC), Division of High Consequence Pathogens and Pathology (DHCPP-PRB)	Centers for Disease Contrai & Prevention (CDC), Division of High Consequence Pathogens and Pathology (DHCPP-PRB)	Gigante,C., Mozer,M., Zhao,H., Batra,D., Hetrick,E., Howard,D., Kovar,L., Seabolt,M., Morrison,S., Desch,M., Knipe,K., Weigand,M., Sheth,M., Burgin,A.. Burroughs,M., Lee,J., Wilkins,K., McCollum,A., Hutson,C., Davidson,W., Rao.A., Hopkins,B. and Li,Y.
EPI_ISL_l6751116	Centers for Disease Contrai & Prevention (CDC), Division of High Consequence Pathogens and Pathology (DHCPP-PRB)	Centers for Disease Contrai & Prevention (CDC), Division of High Consequence Pathogens and Pathology (DHCPP-PRB)	Gigante,C., Buttery,E., Zhao,H, Batra,D., Hetrick,E., Howard,D., Kovar,L., Seabolt,M., Morrison,S., Desch,M., Knipe,K., Weigand,M., Sheth,M., Burroughs,A.B., Lee,J., Wilkins,K., McCollum,A., Hutson,C., Davidson,w., Rao.A., Raman,D and Li,Y.
EPI ISL 16751117, EPI ISL 16751118, EPI ISL 16751119, EPC_ISL l6751120, EPI ISL l6751121, EPI ISL l6751122	Centers for Disease Contrai & Prevention (CDC), Division of High Consequence Pathogens and Pathology (DHCPP-PRB)	Centers for Disease Contrai & Prevention (CDC), Division of High Consequence Pathogens and Pathology (DHCPP-PRB)	Gigante,C., Ruiz,V., Zhao,H., Batra,D., Hetrick,E., Howard,D., Kovar,L., Seabolt.M., Morrison,S., Desch,M., Knipe,K., Weigand,M., Sheth,M., Burgin,A., Burroughs,M., Lee,J., Wilkins,K., McCollum,A., Hutson,C., Davidson,W., Rao,A., Wang,]. and Li.Y.
EPI_ISL_16751123	Centers for Disease Contrai & Prevention (CDC), Division of High Consequence Pathogens and Pathology (DHCPP-PRB)	Centers for Disease Contrai & Prevention (CDC), Division of High Consequence Pathogens and Pathology (DHCPP-PRB)	Gigante,(, Thomas,L., Zhao,H., Batra,D., Hetrick,E., Howard,D., Kovar,L., Seabolt,M., Morrison,S., Desch,M., Knipe,K., Weigand,M., Sheth,M., Burgin,A., Burraughs,M., Lee,J., Wilkins,K., McCollum,A., Hutson,C., Davidson,W., Rao,A., DunnJ andli.Y.
EPI_ISL_16751124	Centers for Disease Contrai & Prevention (CDC), Division of High Consequence Pathogens and Pathology (DHCPP-PRB)	Centers for Disease Contrai & Prevention (CDC), Division of High Consequence Pathogens and Pathology (DHCPP-PRB)	Gigante,C, Kubin,G., Zhao,H., Batra,D., Hetrick,E., Howard,D., Kovar,L., Seabolt.M., Morrison,S., Desch,M., Knipe,K., Sheth,M.R., Burgin,A., Burroughs,M., Lee,J., Wilkins,K., McCollum,A., Hutson,C., Davidson,W., Rao.A., White,S. and Li,Y.
EPI_ISL_16751125, EPI_ISL_16751126	Centers for Disease Contrai & Prevention (CDC), Division of High Consequence Pathogens and Pathology (DHCPP-PRB)	Centers for Disease Contrai & Prevention (CDC), Division of High Consequence Pathogens and Pathology (DHCPP-PRB)	Gigante,C., Pettit,D., Zhao,H., Batra,D., Hetrick,E., Howard,D., Kovar,L., Seabolt,M., Morrison,S., Desch,M., Knipe,K., Weigand,M., Sheth,M., Burgin,A., Burraughs,M., Lee,J., Wilkins,K., McCollum,A., Hutson,C., Davidson,W., Rao,A., Deutsch-Feldman,M. and Li.Y.
EPI_ISL_16751127, EPI_ISL_16751128, EPI_ISL_16751129	Centers for Disease Contrai & Prevention (CDC), Division of High Consequence Pathogens and Pathology (DHCPP-PRB)	Centers for Disease Contrai & Prevention (CDCJ, Division of High Consequence Pathogens and Pathology (DHCPP-PRB)	Gigante,C., Thomas,L., Zhao,H., Batra,D., Hetrick,E., Howard,D., Kovar,L., Seabolt.M., Morrison,S., Desch,M., Knipe,K., Weigand,M., Burroughs,M.S., Lee,J., Wilkins,K., McCollum,A., Hutson,C., Davidson,W., Rao,A., Dunn,J. and Li,Y.
EPI_ISL_16751130	Centers for Disease Contrai & Prevention (CDC), Division of High Consequence Pathogens and Pathology (DHCPP-PRB)	Centers for Disease Contrai & Prevention (CDC), Division of High Consequence Pathogens and Pathology (DHCPP-PRB)	Gigante,C., Kubin,G., Zhao,H., Batra,D., Hetrick,E., Howard,D., Kovar,L., Seabolt.M.. Morrison,S., Desch,M., Knipe,K.. Burraughs,M.R., Lee.J., Wilkins,K.. McCollum,A., Hutson,C., Davidson,W., Rao.A., White.S. and Li.Y.
EPI_ISL_l6751131	Centers for Disease Contrai & Prevention (CDC), Division of High Consequence Pathogens and Pathology (DHCPP-PRB)	Centers for Disease Contrai & Prevention (CDC), Division of High Consequence Pathogens and Pathology (DHCPP-PRB)	Gigante,C., Goldoft,M., Zhao,H., Batra,D., Hetrick,E., Howard,D., Kovar,L., Seabolt,M., Morrison,S., Desch,M., Knipe,K., Weigand,M., Burroughs,M.S., Lee,J., Wilkins,K., McCollum,A., Hutson,C., Davidson,w., Rao.A., Holshue,M. and Li,Y.
EPI_ISL_16751132	Centers for Disease Contrai & Prevention (CDC), Division of High Consequence Pathogens and Pathology (DHCPP-PRB)	Centers for Disease Contrai & Prevention (CDC), Division of High Consequence Pathogens and Pathology (DHCPP-PRB)	Gigante,C., Francis,D., Zhao,H., Batra,D.. Hetrick,E., Howard,D., Kovar,L., Seabolt,M., Morrison,5., Desch,M.. Knipe,K., Weigand,M., Burroughs,M.S., Lee,J., Wilkins,K., McCollum,A., Hutson,C.. Davidson,W., Rao.A.. Escobar,]. and Li.Y.
EPI_ISL_16751133	Centers for Disease Contrai & Prevention (CDC), Division of High Consequence Pathogens and Pathology (DHCPP-PRB)	Centers for Disease Contrai & Prevention (CDC), Division of High Consequence Pathogens and Pathology (DHCPP-PRB)	Gigante,C., Ventura,J., Zhao,H., Batra,D., Hetrick,E., Howard,D., Kovar,L., Seabolt,M., Morrison,S, Desch,M., Knipe,K., Weigand,M, Sheth,M., Burgin,A., Burraughs,M., Lee,J., Wilkins,K., McCollum,A., Hutson,C., Davidson,W., Rao,A., Nash,]. and li.Y.
EPI_ISL_16751134	Centers for Disease Contrai & Prevention (CDC), Division of High Consequence Pathogens and Pathology (DHCPP-PRB)	Centers for Disease Contrai & Prevention (CDC), Division of High Consequence Pathogens and Pathology (DHCPP-PRB)	Gigante,C., Hauser,J., Zhao,H., Batra,D., Hetrick,E., Howard,D., Kovar,L., Seabolt,M., Knipe,K., Burroughs,M.S., Lee,J., Wilkins,K., McCollum,A., Hutson,C., Davidson,W., Rao.A., Mangla,A. and li.Y.
EPI_ISL_16751135	Centers for Disease Contrai & Prevention (CDC), Division of High Consequence Pathogens and Pathology (DHCPP-PRB)	Centers for Disease Contrai & Prevention (CDC), Division of High Consequence Pathogens and Pathology (DHCPP-PRB)	Gigante,C., Lee,P., Zhao,H., Batra,D., Hetrick,E., Howard,D., Kovar,L., Seabolt,M., Morrison,S., Desch,M., Knipe,K., Weigand,M., Burraughs,M.5., Lee,J., Wilkins,K., McCollum,A., Hutson,C., Davidson,W., Rao,A., Stanek,D. and Li.Y.
EPI ISL 16751136, EPI ISL 16751137, EPI ISL 16751138, EPI ISL 16751139	Centers for Disease Contrai & Prevention (CDC), Division of High Consequence Pathogens and Pathology (DHCPP-PRB)	Centers for Disease Contrai & Prevention (CDCJ, Division of High Consequence Pathogens and Pathology (DHCPP-PRB)	Gigante,C., Pavlick,J., Zhao,H., Batra,D., Hetrick,E., Howard,D., Kovar,L., Seabolt.M., Morrison,S , Desch,M., Knipe,K., Weigand,M , Sheth,M., Burgin,A., Burroughs,M., Lee,J., Wilkins,K., McCollum,A., Hutson,C., Davidson,W., Rao,A., Parratt,T. and Li.Y.
EPI_ISL_16751140	Centers for Disease Contrai & Prevention (CDC), Division of High Consequence Pathogens and Pathology (DHCPP-PRB)	Centers for Disease Contrai & Prevention (CDC), Division of High Consequence Pathogens and Pathology (DHCPP-PRB)	Gigante,C., Culbertson,M., Zhao,H.. Batra.D., Hetrick,E., Howard,D., Kovar,L., Seabolt,M., Weigand,M., Burraughs,M.S., Lee,]., Wilkins,K., McCollum,A., Hutson,C., Davidson.W., Rao.A., Pope,B. and Li.Y.
EPI_ISL_16751141	Centers for Disease Contrai & Prevention (CDC), Division of High Consequence Pathogens and Pathology (DHCPP-PRB)	Centers for Disease Contrai & Prevention (CDC), Division of High Consequence Pathogens and Pathology (DHCPP-PRB)	Gigante,C., Haydel,D., Zhao,H., Batra,D., Hetrick,E., Howard,D., Kovar,L., Seabolt.M., Weigand,M., Burroughs,M.S., Lee,J., Wilkins,K., McCollum,A., Hutson,C., Davidson,w., Rao.A., Salinas.A. and Li.Y.
EPI_ISL_16751142, EPI_ISL_16751143, EPI_ISL_16751144	Centers for Disease Contrai & Prevention (CDC), Division of High Consequence Pathogens and Pathology (DHCPP-PRB)	Centers for Disease Contrai & Prevention (CDC), Division of High Consequence Pathogens and Pathology (DHCPP-PRB)	Gigante,C., Ostadkar,R., Zhao,H.. Batra,D.. Hetrick,E., Howard,D., Kovar,L., Seabolt,M., Weigand,M.. Knipe,K., Burroughs,M.S.. Lee,).. Wilkins,K., McCollum,A.. Hutson,C., Davidson,W., Rao,A., Wang.X. and Li,Y.
EPI_ISL_16751145	Centers for Disease Contrai & Prevention (CDC), Division of High Consequence Pathogens and Pathology (DHCPP-PRB)	Centers for Disease Contrai & Prevention (CDC), Division of High Consequence Pathogens and Pathology (DHCPP-PRB)	Gigante,C., Kubin,G., Zhao,H, Batra,D., Hetrick,E., Howard,D., Kovar,L., Seabolt,M., Morrison,S.. Desch,M., Knipe,K., Burraughs,M.R., Lee,J., Wilkins,K., McCollum,A., Hutson,C.. Davidson,W., Rao.A., White,S. and li.Y.
EPI_ISL_16758555, EPI_ISL_16758556, EPI_ISL_l6758557, EPI_ISL_16758558, EPI_ISL_16758559, EPI_ISL_16758560, EPI_ISL_16758561, EPI_ISL_l6758562, EPI_ISL_16758563, EPI_ISL_16758564, EPI_ISL_16758565			
seeabove	Centre for Biological Threats, Highly Pathogenic Viruses, Robert Koch Institute	Centre for Biological Threats, Highly Pathogenic Viruses, Robert Koch Institute	Brinkmann,A., Kohl,C., Pape,K., Schrick,L., Michel,]., Schaade,L. and Nitsche,A.
EPI ISL 16847486, EPI ISL 16847487	Institute for Medical Virology, University Hospital, Goethe University	Institute for Medical Virology, University Hospital, Goethe University	Denisa Bojkova, Julia Schneider, Victor M. Carman, Martin Michaelis, Jindrich Cinatl jr.
EPI_ISL_l6871158, EPI_ISL_l6871159, EPI_ISL_16871160, EPI_ISL_16871161, EPI_ISL_16871162, EPI_ISL_16871163	Laboratorio de Enterovirus, Instituto Oswaldo Cruz, Fiocruz	Instituto Oswaldo Cruz FIOCRUZ - Laboratory of Respiratory Viruses and Measles (LVRS)	Paola Resende, Elisa Cavalcante Pereira, Bruna Mendonça da Silva, Jéssica Graça Macedo de Carvalho, Larissa Macedo Pinto, Victor Guimaraes, Marilda Siqueira, Renan da Silva Faustino, Marilia Santini, Edson Elias da Silva on behalf of the Fiocruz Genomic Surveillance Network
EPI_ISL_l6905442, EPI_ISL_l6905443, EPI_ISL_l6905444	Tokyo Metropolitan Institute of Public Health,	Tokyo Metropolitan Institute of Public Health,	Kasuya,F., Negishi,A., Kumagai,R., Hasegawa,M., fujiwara,T., Miyake,H., Nagashima,M. and Sadamasu,K.

	Department of Microbiology	Department of Microbiology	
EPI_ISL_l6926988, EPI_ISL_16926991, EPI_ISL_16926994, EPI_ISL_l6926997, EPI_ISL_16927000, EPI_ISL_l6927003, EPI_ISL_16927007, EPI_ISL_16927010, EPI_ISL_l6927013, EPI_ISL_16927016, EPI_ISL_16927018, EPI_ISL_16927021			
see above	Naval Infectious Diseases Diagnostic Laboratory	Naval Medical Research Center Biological Defense Research Directorate	Logan J. Voegtly, Gregory K. Rice. Adrian Pakey, Andrea E. Luquette, Maren C. Fitzpatrick. Hannah M. Drumm, Victor Sugiharto, Hua-Wei Chen, Francisco Malagon, Regina Z. Cer, Kimberly A. Bishop-Lilly
EPI_ISL_l6930148, EPI_ISL_l6930151, EPI_ISL_16930154, EPI_ISL_l6930157, EPI_ISL_16930160, EPI_ISL_l6930165, EPI_ISL_l6930168, EPI_ISL_16930171, EPI_ISL_l6930174, EPI_ISL_16930177, EPI_ISL_16930180, EPI_ISL_16930183			
see above	California Department of Public Health	California Department of Public Health	Probert,W., Espinosa,A., Kath,C., Haw,M., D'Neil,R., Bell,J. and Hacker,J.
EPI_ISL_l6946400	Division de Microbiologia, Hospital Nacional de Nirios Carlos Saenz Herrera	Instituto Costarricense de Investigacion y Enserianza en Nutricion y Salud, Inciensa	Diana Cantillo, Hillary Serrano, Ana Isela Ruiz, Gustavo Vega, Claudio Soto-Garita, Adriana Godinez, Estela Cordera, Melany Calderon, Francisco Quarte
EPI_ISL_l6955153, EPI_ISL_16955154, EPI_ISL_16955155, EPI ISL 16955156, EPI ISL 16955157, EPI ISL 16955158, EPI_ISL_l695-5159, EPI_JSL_l69551fo	CT Department of Public Health	CT Department of Public Health	Claire Pearson, Tu N. Nguyen, Kutluhan Incekara, Neranjan V. Perera
EPI_ISL_16955204, EPI_ISL_16955205, EPJ_ISL_16955206, EPI_JSL_16955207, EPI_ISL_16955208, EPI_ISL_16955209, EPI_ISL_16955210, EPI_ISL_16955211, EPI_ISL_16955212, EPI_ISL_16955213, EPI_ISL_16955214, EPI_ISL_16955215, EPI_ISL_16955216, EPI_ISL_16955217, EPI_ISL_16955218, EPI_ISL_16955219, EPI_ISL_16955220, EPI_ISL_16955221, EPI_ISL_16955222			
see above	Public Health Laboratory, NYC Department of Health and Mental Hygiene	Public Health Laboratory, NYC Department of Health and Mental Hygiene	Wang,J.C., Amin,H.S., Clabby,T.T., Taki,F., Su,M., Rahat.A., De La Cruz,N., Dlsen,A., Thi,C., Silver,S., Akther,S., Chowdhury,M., Omoregie,E. and Hughes,S.
EPI ISL 16955223, EPI ISL 16955224, EPI ISL 16955225, EPI IS(16955226	Centre for Biological Threats, Highly Pathogenic Viruses, Robert Koch Institute	Centre for Biological Threats, Highly Pathogenic Viruses, Robert Koch Institute	Brinkmann,A., Kohl,C., Pape,K., Schrick,L., Michel,J., Schaade,L. and Nitsche,A.
EPI ISL 16955227, EPI ISL 16955228, EPI ISL 16955229, EPI ISL l6955230	Public Health Laboratory, NYC Department of Health and Mental Hygiene	Public Health Laboratory, NYC Department of Health and Mental Hygiene	Wang,J.C., Amin,H.5., Clabby,T.T., Taki,F., Su,M., Rahat,A., De La Cruz,N., Dlsen,A., Thi,C., Silver,S., Akther,S., Chowdhury,M., Dmoregie,E. and Hughes,$.
EPI_ISL_16955231, EPI_ISL_16955232, EPI_ISL_16955233, EPI_ISL_16955234, EPI_ISL_16955235, EPI_ISL_l6955237, EPI_ISL_l6955238	Centre for Biological Threats, Highly Pathogenic Viruses, Robert Koch Institute	Centre for Biological Threats, Highly Pathogenic Viruses, Robert Koch Institute	Brinkmann,A., Kohl,C., Pape,K., Schrick,L., Michel,J., Schaade,L. and Nitsche,A.
EPI ISL 16955239, EPI ISL 16955240, EPI ISL 16955241. EPI ISL 16955242, EPI ISL 16955243, EPI ISL 16955244, EPI ISL 16955245, EPI ISL 16955246, EPI ISL 16955247, EPI ISL 16955248, EPI ISL 16955249, EPI ISL 16955250, EPI ISL 16955251, EPI ISL 16955252, EPI ISL 16955253, EPI ISL 16955254, EPI ISL 16955255, EPI ISL 16955256, EPI ISL 16955257, EPI ISL 16955258, EPI-ISL-16955259, EPI-ISL-16955260, EPJ-ISL-16955261, EPI-JSL-16955262, EPI-ISL-16955263, EPI-ISL-16955264, EPI-ISL-16955265, EPI-ISL-16955266, EPI-ISL-16955267, EPI-ISL-16955268, EPI-ISL-16955269, EPI-ISL-16955270, EPI-ISL-16955271, EPI-ISL-16955272, EPI-ISL-16955273, EPI-ISL-16955274, EPI-ISL-16955275, EPI-ISL-16955276, EPI-ISL-16955277, EPI-ISL-16955278, EPCISL)6955279. EPI=ISL)6955283, EPÜSL)6955284, EPCISL)6955285, EPI=ISL)6955286, EPCISL)6955287. EPI=ISL)6955288, EPÜSL)6955289, EPCISL)6955290			
see above	Public Health Laboratory, NYC Department of Health and Mental Hygiene	Public Health Laboratory, NYC Department of Health and Mental Hygiene	Wang,J.C., Amin,H.S., Clabby,T.T., Taki,F., Su,M., Rahat,A., De La Cruz,N., Dlsen,A., Thi,C., Silver,S., Akther,S., Chowdhury,M., Dmoregie,E. and Hughes,S.
EPI_ISL_l6955293	Laboratory of Virology, University Hospitals of Geneva	Laboratory of Virology, University Hospitals of Geneva	Laubscher,F., Chudzinsk,V., Schibler,M., Kaiser,L. and Renzoni,A.
EPI ISL 16955294, EPI ISL 16955295, EPI ISL 16955296, EPI ISL 16955297, EPI ISL 16955298, EPI ISL 16955299, EPI ISL 16955300, EPI ISL 16955302, EPI ISL 16955303, EPI ISL 16955304, EPI ISL 16955305, EPI ISL 16955306, EPI ISL 16955307, EPI ISL 16955308, EPI ISL 16955309, EPI ISL 16955310, EPI ISL 16955311, EPI ISL 16955312, EPI ISL 16955313, EPI ISL 16955314, EPI=ISL)6955315, EPI=ISL)6955316			
see above	Public Health Laboratory, NYC Department of Health and Mental Hygiene	Public Health Laboratory, NYC Department of Health and Mental Hygiene	Wang,J.C., Amin,H.S., Clabby,T.T., Taki,F., Su,M., Rahat,A., De La Cruz,N., Dlsen,A., Thi,C., Silver,S., Akther,S., Chowdhury,M., Omoregie,E. and Hughes,S.
EPI ISL 16985950, EPI ISL 16985951, EPJ ISL 16985952, EPI JSL 16985954, EPI ISL 16985955, EPI ISL 16985956, EPI ISL 16985957, EPI ISL 16985958, EPI ISL 16985959, EPI ISL 16985960, EPI ISL 16985961, EPI ISL 16985962, EPI ISL 16985963, EPI ISL 16985964, EPI ISL 16985965, EPI ISL 16985966, EPI ISL 16985967, EPI ISL 16985968, EPI ISL 16985969, EPI ISL 16985970, EPCISL)6985971. EPI=ISL)6985972			
see above	National Virus Reference Laboratory	National Virus Reference Laboratory	Gabriel Gonzalez, Michael Carr, Emer D'Byrne, Weronika Banka, Brian Keogan, Jose Maria Urtasun Elizari, Jonathan Dean, Daniel Hare, Cillian f De Gascun
EPI_ISL_16987277	Centers for Disease Contrai & Prevention (CDC), Division of High Consequence Pathogens and Pathology (DHCPP-PRB)	Centers for Disease Contrai & Prevention (CDC), Division of High Consequence Pathogens and Pathology (DHCPP-PRB)	Gigante,C., Buttery,E., Zhao,H., Batra,D., Hetrick,E., Howard,D., Kovar,L., Seabolt,M., Knipe,K., Burroughs,M.S., Lee,J., Wilkins,K., McCollum,A., Hutson,C., Davidson,w., Rao.A., Raman,D. and Li,Y.
EPI ISL 16987278, EPI ISL 16987279, EPI ISL 16987280, EPC_ISL 16987281, EPI ISL 16987282, EPI ISL 16987283	Centers for Disease Contrai & Prevention (CDC), Division of High Consequence Pathogens and Pathology (DHCPP-PRB)	Centers for Disease Contrai & Prevention (CDC), Division of High Consequence Pathogens and Pathology (DHCPP-PRB)	Gigante,C.. Ruiz,V., Zhao,H.. Batra,D., Hetrick,E., Howard,D., Kovar,L., Seabolt.M., Desch,M., Morrison,S., Desch,M., Knipe,K., Weigand,M., Sheth,M., Burgin,A.. Burroughs,M., Lee,J., Wilkins,K., McCollum,A., Hutson,C., Davidson,W., Rao,A., Wang,J. and Li,Y.
EPI_ISL_16987284	Centers for Disease Contrai & Prevention (CDC), Division of High Consequence Pathogens and Pathology (DHCPP-PRB)	Centers for Disease Contrai & Prevention (CDC), Division of High Consequence Pathogens and Pathology (DHCPP-PRB)	Gigante,C., Lee,B, Zhao,H., Batra,D., Hetrick,E., Howard,D., Kovar,L., Seabolt,M., Weigand,M., Knipe,K., Burraughs,M.S., Lee,J., Wilkins,K., McCollum,A., Hutson,C., Davidson,W., Rao,A., Salehi,E. and Li,Y.
EPJ_ISL_16987285, EPI_JSL_l6987286	Centers for Disease Contrai & Prevention (CDC), Division of High Consequence Pathogens and Pathology (DHCPP-PRB)	Centers for Disease Contrai & Prevention (CDC), Division of High Consequence Pathogens and Pathology (DHCPP-PRB)	Gigante,(, Cogswell,K., Zhao,H, Batra,D., Hetrick,E., Howard,D., Kovar,L., Seabolt.M., Morrison.S, Desch,M., Knipe,K., Weigand,M, Sheth,M., Burgin,A., Burroughs,M., Lee,J., Wilkins,K., McCollum,A., Hutson,C., Davidson,W, Rao,A., Grenz,L. and Li.Y.
EPI_ISL_16987287	Centers for Disease Contrai & Prevention (CDC), Division of High Consequence Pathogens and Pathology (DHCPP-PRB)	Centers for Disease Contrai & Prevention (CDC), Division of High Consequence Pathogens and Pathology (DHCPP-PRB)	Gigante,C., Xia,□., Zhao,H., Batra,D., Hetrick,E., Howard,D., Kovar,L., Seabolt.M., Morrison,S, Desch,M., Knipe,K., Weigand,M, Sheth,M., Burgin,A., Burroughs,M., Lee,J., Wilkins,K., McCollum,A., Hutson,C., Davidson,w., Rao,A., Plipat,N. and Li.Y.
EPI_ISL_l6987288	Centers for Disease Contrai & Prevention (CDC), Division of High Consequence Pathogens and Pathology (DHCPP-PRB)	Centers for Disease Contrai & Prevention (CDC), Division of High Consequence Pathogens and Pathology (DHCPP-PRB)	Gigante,(, Thomas,L., Zhao,H., Batra,D., Hetrick,E., Howard,D., Kovar,L., Seabolt,M., Morrison,S., Desch,M., Knipe,K., Weigand,M., Sheth,M., Burgin,A., Burroughs,M., Lee,J., Wilkins,K., McCollum,A., Hutson,C., Davidson,W., Rao,A., Dunn,J. and Li,Y.
EPI_ISL_16987289, EPI_ISL_l6987290, EPI_ISL_16987291, EPI_ISL_l6987292	Centers for Disease Contrai & Prevention (CDC), Division of High Consequence Pathogens and Pathology (DHCPP-PRB)	Centers for Disease Contrai & Prevention (CDC), Division of High Consequence Pathogens and Pathology (DHCPP-PRB)	Gigante,C Kubin,G., Zhao,H., Batra,D., Hetrick,E., Howard,D., Kovar,L., Seabolt,M., Morrison,S., Desch,M., Knipe,K., Sheth,M.R., Burgin,A., Burroughs,M., Lee,J., Wilkins,K., McCollurn,A., Hutson,C., Davidson,W., Rao,A, White,S. and Li.Y.
EPJ_ISL_16987293, EPI_JSL_16987294	Centers for Disease Contrai & Prevention (CDC), Division of High Consequence Pathogens and Pathology (DHCPP-PRB)	Centers for Disease Contrai & Prevention (CDC), Division of High Consequence Pathogens and Pathology (DHCPP-PRB)	Gigante,C., Murray,J., Zhao,H., Batra,D., Hetrick,E., Howard,D., Kovar,L., Seabolt.M., Knipe,K., Burroughs,M.S., Lee,J., Wilkins,K., McCollum,A., Hutson,C., Davidson,W., Rao.A., Atkinson,A. and li.Y
EPI_ISL_16987295	Centers for Disease Contrai & Prevention (CDC), Division of High Consequence Pathogens and Pathology (DHCPP-PRB)	Centers for Disease Contrai & Prevention (CDC), Division of High Consequence Pathogens and Pathology (DHCPP-PRB)	Gigante,C., Goldoft,M.. Zhao.H., Batra,D.. Hetrick,E.. Howard.D., Kovar.L., Seabolt,M.. Morrison,S., Desch.M., Knipe.K., Weigand,M., Burraughs,M.S.. Lee.J.. Wilkins,K.. McCollum,A., Hutson,C.. Davidson.W.. Rao.A.. Holshue,M. and Li.Y.
EPI ISL 16987296, EPI ISL 16987297, EPI ISL 16987298, EPI ISL 16987299	Centers for Disease Contrai & Prevention (CDC), Division of High Consequence Pathogens and Pathology (DHCPP-PRB)	Centers for Disease Contrai & Prevention (CDC), Division of High Consequence Pathogens and Pathology (DHCPP-PRB)	Gigante,C., Ventura,J., Zhao,H., Batra,D., Hetrick,E., Howard,D., Kovar,L., Seabolt,M., Morrison,S, Desch,M., Knipe,K., Weigand,M, Sheth,M., Burgin,A., Burroughs,M., Lee,J., Wilkins,K., McCollum,A., Hutson,C., Davidson,W., Rao,A., Nash,J. and Li,Y.
EPJ_ISL_16987300. EPI_JSL_l6987301	Centers for Disease Contrai & Prevention (CDC), Division of High Consequence Pathogens and Pathology (DHCPP-PRB)	Centers for Disease Contrai & Prevention (CDC), Division of High Consequence Pathogens and Pathology (DHCPP-PRB)	Gigante.C.. Steidley,B., Seabolt,M.. Zhao.H., Wilkins,K.. McCollum,A.. Hutson,C., Davidson,W.. Rao.A.. Davizon,E. and Li.Y.
EPI ISL 16987302, EPI ISL 16987303, EPI ISL 16987380, EPI ISL l6987381	Centers for Disease Contrai & Prevention (CDC), Division of High Consequence Pathogens and Pathology (DHCPP-PRB)	Centers for Disease Contrai & Prevention (CDC), Division of High Consequence Pathogens and Pathology (DHCPP-PRB)	Gigante,C., Hauser,J., Zhao,H., Batra,D., Hetrick,E., Howard,D., Kovar,L., Seabolt,M., Morrison,S., Desch,M., Knipe,K., Weigand,M., Sheth,M., Burgin,A., Burroughs,M., Lee,J., Wilkins,K., McCollum,A., Hutson,C., Davidson,w., Rao.A., Mangla.A. and Li,Y.
EPI ISL 16987382, EPI ISL 16987383, EPI ISL 16987384, EPI ISL 16987385	Centers for Disease Contrai & Prevention (CDC), Division of High Consequence Pathogens and Pathology (DHCPP-PRB)	Centers for Disease Contrai & Prevention (CDC), Division of High Consequence Pathogens and Pathology (DHCPP-PRB)	Gigante,C., Lee,P., Zhao,H., Batra,D., Hetrick,E., Howard,D., Kovar,L., Seabolt,M., Morrison,S., Desch,M., Knipe,K., Weigand,M., Sheth,M., Burgin,A., Burroughs,M., Lee,J., Wilkins,K., McCollum,A., Hutson,C., Davidson,W., Rao,A., Stanek,D. and Li,Y.
EPI_ISL_16987386	Centers for Disease Contrai & Prevention (CDC), Division of High Consequence Pathogens and Pathology (DHCPP-PRB)	Centers for Disease Contrai & Prevention (CDC), Division of High Consequence Pathogens and Pathology (DHCPP-PRB)	Gigante,C., Pavlick,J., Zhao,H., Batra,D., Hetrick,E., Howard,D., Kovar,L., Seabolt,M., Morrison,S., Desch,M., Knipe,K., Weigand,M., Sheth,M., Burgin.A., Burroughs,M., Lee,J., Wilkins,K., McCollum,A., Hutson,C., Davidson,W., Rao,A., Parrott.T. and Li.Y.
EPI_ISL_16987387	Centers for Disease Contrai & Prevention (CDC), Division of High Consequence Pathogens and Pathology (DHCPP-PRB)	Centers for Disease Contrai & Prevention (CDC), Division of High Consequence Pathogens and Pathology (DHCPP-PRB)	Gigante,C., Ceniseros,A., Zhao,H., Batra,D., Hetrick,E., Howard,D., Kovar,L., Seabolt,M., Morrison,S., Desch,M., Knipe,K., Weigand,M., Burroughs,M.S., Lee,J., Wilkins,K., McCollum,A., Hutson,C., Davidson,W., Rao,A., Cahill,M. and li.Y.
EPJ_ISL_16987388. EPI_JSL_16987389	Centers for Disease Contrai & Prevention (CDC), Division of High Consequence Pathogens and Pathology (DHCPP-PRB)	Centers for Disease Contrai & Prevention (CDC), Division of High Consequence Pathogens and Pathology (DHCPP-PRB)	Gigante,C.. Ghinai,I.. Zhao,H., Batra,D., Hetrick.E.. Howard.D.. Kovar,L., Seabolt,M.. Morrison,S.. Desch,M., Knipe.K.. Weigand,M.. Sheth,M . Burgin,A.. Burroughs.M., Lee.J., Wilkins.K., McCollum.A., Hutson.C., Davidson,W., Rao,A., Kerins,J. and li,Y.
EPI_ISL_l6987390	Centers for Disease Contrai & Prevention (CDC), Division of High Consequence Pathogens and Pathology (DHCPP-PRB)	Centers for Disease Contrai & Prevention (CDC), Division of High Consequence Pathogens and Pathology (DHCPP-PRB)	Gigante,C., Salinas.A., Zhao,H., Batra,D., Hetrick,E., Howard,D., Kovar,L., Seabolt.M., Morrison,S., Desch,M., Knipe,K., Burraughs,M.R., Lee,J., Wilkins,K., McCollum,A., Hutson,C, Davidson,W, Rao.A., Haydel,D. and Li,Y.
EPI_ISL_16987391, EPI_ISL_l6987392, EPI_ISL_16987393	Centers for Disease Contrai & Prevention (CDC), Division of High Consequence Pathogens and Pathology (DHCPP-PRB)	Centers for Disease Contrai & Prevention (CDC), Division of High Consequence Pathogens and Pathology (DHCPP-PRB)	Gigante,C.. Johnson.S.. Zhao,H.. Batra,D., Hetrick,E., Howard,D.. Kovar,L., Seabolt,M., Weigand,M., Knipe,K., Burroughs,M.S.. Lee,J., Wilkins,K., McCollum,A.. Hutson,C.. Davidson,W.. Rao.A.. Riner.D. and Li,Y.
EPI_ISL_l6997389, EPI_ISL_l6997390, EPJ_ISL_l6997391, EPI_JSL_l6997392, EPI_ISL_l6997393, EPI_ISL_l6997394, EPI_ISL_l6997395, EPI_ISL_l6997396, EPI_ISL_l6997397, EPI_ISL_l6997398, EPI_ISL_l6997399, EPI_ISL_l6997400, EPI_ISL_l6997401, EPI_ISL_l6997402, EPI_ISL_l6997403			
see above	California Department of Public Health	California Department of Public Health	Probert,W., Espinosa,A., Kath,C., Haw,M., D'Neil,R., Bell,J. and Hacker,J.
EPI_ISL_16997404, EPI_ISL_l6997405, EPI_ISL_16997406	Quest Diagnostics Nichais Institute	Los Angeles County Public Health Laboratories	P. Hemarajata et al.

EPI_ISL_16997407, EPI_ISL_l6997411	Los Angeles County Public Health Laboratories	Los Angeles County Public Health Laboratories	P. Hemarajata et al.
EPI ISL 16997413, EPI ISL 16997415, EPI ISL 16997417, EPI ISL 16997418, EPI ISL 16997419, EPI ISL 16997421. EPI ISL 16997422, EPI ISL 16997423, EPI ISL 16997424, EPI ISL 16997425, EPI ISL 16997426, EPI ISL 16997427, EPI ISL 16997432, EPI ISL 16997433, EPI ISL 16997434, EPI ISL 16997435, EPI ISL 16997437, EPI ISL 16997439, EPI ISL 16997440, EPI ISL 16997441. EPI=ISL)6997442, EPI=ISL)6997443, EPÜSL)6997444, EPI=ISL)6997445, EPI=ISL)6997446			
see above	Kaiser Permanente Chine Hills Regional Reference Laboratories	Los Angeles County Public Health Laboratories	P. Hemarajata et al.
EPI ISL 16997447, EPI ISL 16997449, EPI ISL 16997450, EPLISL l6997451, EPI ISL l6997452. EPI ISL l6997453	Laboratory Corporation of America	Los Angeles County Public Health Laboratories	P. Hemarajata et al.
EPI_ISL_l6997455, EPI_ISL_l6997456, EPI_ISL_l6997457, EPI_ISL_l6997458	Los Angeles County Public Health Laboratories	Los Angeles County Public Health Laboratories	P. Hemarajata et al.
EPI_ISL_l6997460, EPI_ISL_l6997461, EPI_ISL_l6997462. EPI ISL 16997463, EPI ISL 16997464, EPI ISL 16997465, EPI=ISL=l6997466, EPI=ISL=l6997467, EPI=ISL=l6997468, EPI_ISL_l6997469	Quest Diagnostics Nichais Institute	Los Angeles County Public Health Laboratories	P. Hemarajata et al.
EPI_ISL_l6997470	UCLA Clinical Micro Lab	Los Angeles County Public Health Laboratories	P. Hemarajata et al.
EPI_ISL_l6999059, EPI_ISL_l6999060, EPI_ISL_l6999062, EPI_JSL_l6999064, EPI_ISL_l6999065, EPI_ISL_16999066, EPI_ISL_l6999067, EPI_ISL_l6999068, EPI_ISL_l6999069, EPI_ISL_l6999070, EPI_ISL_16999071, EPI_ISL_l6999073, EPI_ISL_l6999074, EPI_ISL_l6999075, EPI_ISL_l6999076, EPI_ISL_l6999077, EPI_ISL_l6999078, EPI_ISL_l6999079, EPI_ISL_l6999081, EPI_ISL_l6999082, EPI ISL 16999084, EPI ISL 16999085, EPI ISL 16999086, EPI ISL 16999087, EPI ISL 16999090, EPI ISL 16999091. EPI ISL 16999093, EPI ISL 16999095, EPI ISL 16999098, EPI ISL 16999099, EPI ISL 16999100, EPI ISL 16999101. EPI ISL 16999102, EPI ISL 16999104, EPI ISL 16999105, EPI ISL 16999108, EPI ISL 16999110, EPI ISL 16999111, EPI ISL 16999113, EPI ISL 16999114, EPCISL)6999115, EPI=ISL)6999116, EPÜSL)6999117, EPI=ISL)6999120, EPI=ISL)6999121, EPI=ISL)6999123, EPI=ISL)6999124, EPÜSL)6999125, EPI=ISL)6999126, EPI=ISL)6999127, EPI=ISL)6999128, EPI=ISL)6999129, EPÜSL)6999131, EPI=ISL)6999132, EPI=ISL)6999133, EPI=ISL)6999134, EPI=ISL)6999137, EPÜSL)6999138, EPI=ISL)6999139			
see above	Laboratorio de Referencia Nacional de Virus Inmunoprevenibles. Centre Nacional de Salud Publica. Instiuto Nacional de Salud	Equipa de Vigilancia Genomica. Area de Innovaci6n y Desarrollo. Centra Nacional de Salud Publica. Instiuto Nacional de Salud	Carlos Patricio Padilla Rojas, Carmen Ver6nica Hurtado Vela, Juana Iris Silva Molina, Luis Bârcena Flores, Victor Jiménez Vâsquez, Alicia Elizabeth Nûliez llanos, Wendy lizarraga Olivares, Luren Nieves Sevilla Catalieda, Kelly Vanessa Izarra Rojas, Karla vasquez Cajachahua, Steve Vladimir Acedo Lazo, Omar Alberto caceres Rey, Henri Bail6n Calder6n, Priscila Nayu Lope Pari, Nancy Rojas Serrano, Gloria Arotinco Garayar. Equipa de Vigilancia Genomica del Instituto Nacional de Salud.
EPI_ISL_l 7008293. EPI_ISL_l 7008294, EPI_ISL_17008295. EPI_ISL_l 7008296	Tokyo Metropolitan Institute of Public Health, Department of Microbiology	Tokyo Metropolitan Institute of Public Health. Department of Microbiology	Kasuya,f., Negishi,A., Kumagai,R., Hasegawa,M., fujiwara,T., Miyake,H., Nagashima.M. and Sadamasu.K.
EPI_ISL_l7008374. EPI_ISL_l7008375, EPI_ISL_l7008376, EPI_ISL_l7008377, EPI_ISL_l7008378, EPI_ISL_l7008379. EPI_ISL_l7008380, EPI_ISL_l7008381, EPI_ISL_l7008382, EPI_ISL_l7008383, EPI_ISL_l7008384			
see above	Centre for Biological Threats, Highly Pathogenic Viruses, Robert Koch Institute	Centre for Biological Threats, Highly Pathogenic Viruses, Robert Koch Institute	Brinkmann,A., Kohl,C., Pape,K., Schrick,L., Michel,]., Schaade,L. and Nitsche,A.
EPI_ISL_l7012023. EPI_ISL_l7012024, EPI_ISL_l7012025, EPI_ISL_l7012026, EPI_ISL_l7012027, EPI_ISL_l7012028. EPI_ISL_l7012029, EPI_ISL_l7012031, EPI_ISL_l7012032, EPI_ISL_l7012033, EPI_ISL_l7012036. EPI_ISL_l7012037, EPI_ISL_l7012038, EPI_ISL_17012039, EPI_ISL_l7012040, EPI_ISL_l7012041. EPI_ISL_l7012042, EPI_ISL_l7012043, EPI_ISL_17012044, EPI_ISL_l7012047, EPI_ISL_l 7012049, EPI_ISL_l 70120SO, EPI_ISL_l 7012051, EPI_ISL_l 7012052, EPI_ISL_l 7012053, EPI_ISL_l 7012054, EPI_ISL_l 70120S5, EPI_ISL_l 7012056, EPI_ISL_l 7012057, EPI_ISL_l 7012059, EPI_ISL_l 7012060, EPI_ISL_l 7012061, EPI_ISL_l 7012062, EPI_ISL_17012063, EPI_ISL_l 7012064, EPI_ISL_l 7012067, EPI_ISL_l 7012068, EPI_ISL_l 7012069, EPI_ISL_17012071			
see above	Laboratorio de Virus Exantematicos, Gastroentéricos y Otros Transmitidos por Vectores	Centre de Referencia Nacional de Genomica, Secuenciacion y Bioinformatica GENS8I0, INSPI-CZ9	Andrés Carrazco, Silvia Salgado, Diana Gutiérrez, Damaris Alarc6n, Andrés Herrera, Andrés Tinizaray, Martha Silnchez, Johanna Parra les, Diego Morales, Jorge Bejarano, Leandro Patilio.
EPI_ISL_l7012073	Laboratorio de Virus Exantematicos, Gastroentéricos y Otros Transmitidos por Vectores	Centre de Referencia Nacional de Genomica, Secuenciacion y Bioinformatica GENSBIO, INSPI-CZ12	Andrés Carrazco, Silvia Salgado, Diana Gutiérrez, Damaris Alarc6n, Andrés Herrera, Andrés Tinizaray, Martha Sanchez, Johanna Parrales, Diego Morales, Jorge Bejarano, Leandro Patirio.
EPI_ISL_l7012074	Laboratorio de Virus Exantematicos, Gastroentéricos y Otros Transmitidos por Vectores	Centre de Referencia Nacional de Genomica, Secuenciacion y 8ioinformatica GENSBIO, INSPI-CZ14	Andrés Carrazco, Silvia Salgado, Diana Gutiérrez, Damaris Alarc6n, Andrés Herrera, Andrés Tinizaray, Martha Sânchez, Johanna Parrales, Diego Morales, Jorge Bejarano, Leandro Patilio.
EPI_ISL_l 701207S	Laboratorio de Virus Exantematicos. Gastroentéricos y Otros Transmitidos por Vectores	Centre de Referencia Nacional de Genomica, Secuenciacion y Bioinformatica GENSBIO, INSPI-CZ15	Andrés Carrazco, Silvia Salgado, Diana Gutiérrez, Damaris Alarc6n, Andrés Herrera, Andrés Tinizaray, Martha Sanchez, Johanna Parrales, Diego Morales, Jorge Bejarano, Leandro Patirio.
EPI_ISL_l 7012076	Laboratorio de Virus Exantematicos, Gastroentéricos y Otros Transmitidos por Vectores	Centre de Referencia Nacional de Genomica, Secuenciacion y Bioinformatica GENSBIO. INSPI-CZ16	Andrés Carrazco, Silvia Salgado, Diana Gutiérrez, Damaris Alarc6n, Andrés Herrera, Andrés Tinizaray, Martha Sânchez, Johanna Parrales, Diego Morales, Jorge Bejarano, Leandro Patirio.
EPI_ISL_l7012077	Laboratorio de Virus Exantematicos, Gastroentéricos y Otros Transmitidos por Vectores	Centre de Referencia Nacional de Genomica, Secuenciacion y Bioinformatica GENSBIO, INSPI-CZ18	Andrés Carrazco, Silvia Salgado, Diana Gutiérrez, Damaris Alarc6n. Andrés Herrera, Andrés Tinizaray, Martha Sanchez, Johanna Parrales, Diego Morales, Jorge Bejarano, Leandro Patilio.
EPI ISL 17012079, EPI ISL 17012080, EPI ISL 17012082, EPI ISL 17012084, EPI ISL 17012088, EPI ISL 17012089, EPI ISL 17012090, EPI ISL 17012091, EPI ISL 17012092, EPI ISL 17012094, EPI ISL 17012096, EPI ISL 17012097, EPI ISL 17012100, EPI ISL 17012101, EPI ISL 17012102, EPI ISL 17012104, EPI ISL 17012107, EPI ISL 17012108, EPI ISL 17012109, EPI ISL 17012110, EPCISL)7012111. EPI=ISL)7012115			
see above	Laboratorio de Virus Exantematicos, Gastroentéricos y Otros Transmitidos por Vectores	Centre de Referencia Nacional de Genomica, Secuenciacion y Bioinformatica GENSBIO, INSPI-CZ9	Andrés Carrazco-Motalvo, Silvia Salgado, Diana Gutiérrez, Damaris Alarc6n, Andrés Herrera, Andrés Tinizaray, Ruth G6mez, Martha Sânchez, Johanna Parrales, Diego Morales, Jorge Bejarano, Leandro Patilio.
EPI ISL 17018429, EPI ISL 17018430, EPI ISL 17018431, EPI=ISL)7018433, EPI=ISL)7018434, EPI=ISL)7018435, EPI ISL 17018436, EPI ISL 17018437, EPI ISL 17018438, EPI ISL I7018439	National Institute for Infectious Diseases "Matei Bals"	National Institute for Infectious Diseases "Matei Bals"	Robert Hohan, Marius Surleac, Leontina Banica, Andreea Tudor, Simona Paraschiv
EPI ISL 17019459, EPI ISL 17019461. EPI ISL 17019462, - - EPI_ISL_I7019464, EPI_ISL_I 701946-7	Parkland Health and Hospital System	Dallas County Health & Human Services Public Health Laboratory	Kabir, Farruk; Plaisance, Erin; Stringer, Joey; Short, Luke.
EPI_ISL_l 7019470	MD Progressive Care	Dallas County Health & Human Services Public Health Laboratory	Kabir, Farruk; Plaisance, Erin; Stringer, Joey: Short, Luke.
EPI_ISL_l7019472	White Rock Medical Center	Dallas County Health & Human Services Public Health Laboratory	Kabir, Farruk; Plaisance, Erin; Stringer, Joey; Short, Luke.
EPI_ISL_l 7019473	Children's Health Dallas Texas	Dallas County Health & Human Services Public Health Laboratory	Kabir. Farruk; Plaisance, Erin; Stringer. Joey; Short. Luke.
EPI ISL 17019476	Dallas County Jail	Dallas County Health & Human Services Public Health laboratory	Kabir. Farruk; Plaisance, Erin; Stringer. Joey: Short. Luke.
EPI ISL 17048204, EPI ISL 17048205, EPI ISL 17048206, EPI ISL I7048207	laboratorio Central de Saude Publica do Estado da Bahia (LACEN/BA)	laboratory of Respiratory Viruses and Measles, Oswaldo Cruz Institute, FIOCRUZ	Paola Resende, Fernando Motta, Elisa Cavalcante Pereira, Bruna Mendonça da Silva, Jéssica Graça Macedo de Carvalho, Larissa Macedo Pinto, Victor Guimaraes, Felicidade Pereira, Marilda Siqueira, Renan da Silva Faustino, Marilia Santini, Edson Elias da Silva on behalf of the Fiocruz COVID-19 Genomic Surveillance Network
EPI_ISL_l7048208	Laboratorio de Enterovirus, Instituto Oswaldo Cruz, Fiocruz	Laboratory of Respiratory Viruses and Measles, Oswaldo Cruz Institute, FIOCRUZ	Paola Resende, Elisa Cavalcante Pereira, Bruna Mendonça da Silva, Jéssica Graça Macedo de Carvalho, Larissa Macedo Pinto, Victor Guimaraes, Marilda Siqueira, Renan da Silva Faustino, Marilia Santini, Edson Elias da Silva on behalf of the Fiocruz Genomic Surveillance Network
EPI ISL 17085647, EPI ISL 17085649, EPI ISL 17085650, EPI ISL 17085652, EPI ISL 17085653, EPI ISL 17085655, EPI ISL 17085656, EPI ISL 17085658, EPI ISL 17085659, EPI ISL 17085661, EPI ISL 17085662, EPI ISL 17085664, EPI ISL 17085665, EPI ISL 17085667, EPI ISL 17085668, EPI ISL 17085670, EPI ISL 17085671, EPI ISL 17085673, EPI ISL 17085674, EPI ISL 17085676, EP(ISL)7085678. EPI=ISL) 7085681, EPÜSL)7085682. EP(JSL) 7085684. EPI=ISL) 7085685, EP(ISL)7085687. EPI=ISL) 7085688, EPÜSL)7085690. EP(ISL) 7085692. EPI=ISL) 7085693, EP(ISL)7085695. EPI=ISL) 7085696, EPÜSL)7085698. EPI=ISL)7085700. EPI=ISL) 7085701, EP(ISL) 7085703. EPI=ISL) 7085704, EPÜSL)7085706. EP(ISL)7085708, EPI=ISL) 7085709, EPI_ISL_I7085712			
see above	Public Health Laboratory, NYC Department of Health and Mental Hygiene	Public Health Laboratory, NYC Department of Health and Mental Hygiene	Wang,J.C., Amin,H.S., Clabby,T.T., Taki,F., Su,M., Rahat,A., De La Cruz,N., Olsen,A., Thi,C., Silver,S., Akther,S., Chowdhury,M., Omoregie,E. and Hughes,S.
EPI ISL 17085714, EPI ISL 17085715, EPI ISL 17085717, EPI ISL 17085719, EPI ISL 17085720, EPI ISL 17085722, EPI ISL 17085723, EPI ISL 17085725, EPI ISL 17085726, EPI ISL 17085728, EPI ISL 17085730, EPI ISL 17085733, EPI ISL 17085734, EPI ISL 17085736, EPI ISL 17085737, EPI ISL 17085739, EPI ISL 17085740, EPI ISL 17085742, EPI ISL 17085743, EPI ISL 17085745, EPI=ISL)7085746, EPI=ISL)7085748, EPÜSL)7085750, EP1=15L)7085752, EPI=ISL)7085753, EPI=ISL)7085755, EPI=ISL)7085756, EPÜSL)7085758, EP1=15L)7085760, EPI=ISL)7085761, EPI=ISL)7085762, EP1=15L)7085764, EPÜSL)7085765, EP1=15L)7085767, EPI=ISL)7085769, EPI=ISL)7085770. EPI=ISL)7085771, EPÜSL)7085773, EP1=15L)7085774, EPI=ISL)7085776, EPI ISL 17085777, EPI ISL 17085779, EPJ ISL 17085781, EPI JSL 17085782, EPI ISL 17085784, EPI ISL 17085785, EPI ISL 17085787, EPI ISL 17085788, EPI ISL 17085790, EPI ISL 17085792, EPI ISL 17085793, EPI ISL 17085794, EPI ISL 17085795, EPI ISL 17085796, EPI ISL 17085797, EPI ISL 17085798, EPI ISL 17085920, EPI ISL 17085921, EPI ISL 17085922, EPI ISL 17085923, EPI-ISL-17085924, EPI-ISL-17085925, EPI-ISL-17085926, EPI-ISL-17085927, EPI-ISL-17085928, EPI-ISL-17085929, EPI-ISL-17085930, EPI-ISL-17085931, EPI-ISL-17085932, EPI-ISL-17085933, EPI-ISL-17085934, EPI-ISL-17085935, EPI-ISL-17085936, EPI-ISL-17085937, EPI-ISL-17085938, EPI-ISL-17085939, EPI-ISL-17085940, EPI-ISL-17085941, EPI-ISL-17085942, EPI-ISL-17085943, EP(ISL)7085944. EPCtsL)7085945, EPÜSL)7085946. EP(JSL)7085947, EPI=ISL)7085948, EP(ISL)7085949. EPI=ISL)7085950, EPÜSL)7085951. EP(ISL)7085952, EPI=ISL)7085953, EP(ISL)7085954. EPI=ISL)7085955, EPÜSL)7085956. EPI=ISL)7085957, EPI=ISL)7085958, EP(ISL)7085959. EPI=ISL)7085960. EPÜSL)7085961			
see above	Public Health laboratory, NYC Department of Health and Mental Hygiene	Public Health Laboratory, NYC Department of Health and Mental Hygiene	Clabby,T.T., Amin,H.S., Wang,J.C., Taki,F., Su,M., Rahat,A., De La Cruz,N., Olsen,A., Thi,C., Silver,S., Akther,S., Chowdhury,M., Omoregie,E. and Hughes,S.
EPI_ISL_l 7085962, EPI_ISL_l 7085963, EPJ_ISL_l 7085964. EPI_JSL_l 7085965, EPI_ISL_l 7085966, EPI_ISL_l 7085967. EPI_ISL_l 7085968, EPI_ISL_l 7085969. EPI_ISL_l 7085970. EPI_ISL_l 7085971, EPI_ISL_l 7085972, EPI_ISL_l 7085973, EPI_ISL_l 7085974. EPI_ISL_17085975. EPI_ISL_l 7085976, EPI_ISL_l 7085977. EPI_ISL_l 7085978. EPI_ISL_l 7085979. EPI_ISL_17085980, EPI_ISL_l 7085981, EPI ISL 17085983, EPI ISL 17085984, EPI ISL 17085985, EPI ISL 17085986, EPI ISL 17085987, EPI ISL 17085988, EPI ISL 17085989, EPI ISL 17085990, EPI ISL 17085991, EPI ISL 17085992, EPI ISL 17085993, EPI ISL 17085994, EPI ISL 17085995, EPI ISL 17085996, EPI ISL 17085997, EPI ISL 17085998, EPI ISL 17085999, EPI ISL 17086000, EPI ISL 17086001, EPI ISL 17086002, EPI-ISL-17086003, EPI-ISL-17086004, EPJ-ISL-17086005, EPI-ISL-17086006, EPI-ISL-17086007, EPI-ISL-17086008, EPI-ISL-17086009, EPI-ISL-17086010, EPI-ISL-17086011, EPI-ISL-17086012, EPI-ISL-17086013, EPI-ISL-17086014, EPI-ISL-17086015, EPI-ISL-17086016, EPI-ISL-17086017, EPI-ISL-17086018. EPI-ISL-17086019, EPI-ISL-17086020, EPI-ISL-17086021, EPI-ISL-17086022, EPI-ISL-17086023, EPI-ISL-17086024, EPJ-ISL-17086025, EPI-JSL-17086026, EPI-ISL-17086027, EPI-ISL-17086028. EPI-ISL-17086029, EPI-ISL-17086030, EPI-ISL-17086031, EPI-ISL-17086032, EPI-ISL-17086033, EPI-ISL-17086034, EPI-ISL-17086035. EPI-ISL-17086036, EPI-ISL-17086037, EPI-ISL-17086038, EPI-ISL-17086039, EPI-ISL-17086040, EPI-ISL-17086042. EPI-ISL-17086043, EPI=ISL)7086044, EPI=ISL)7086045, EPÜSL)7086046, EPI=ISL)7086048, EPI=ISL)7086049, EPI=ISL)7086050, EPI=ISL)7086051, EPÜSL)7086052, EPI=ISL)7086053, EPI=ISL)7086054, EPI=ISL)7086055, EPI=ISL)7086056, EPÜSL)7086057, EPI=ISL)7086058, EPI=ISL)7086059, EPI=ISL)7086060, EPI=ISL)7086061			
see above	Public Health Laboratory, NYC Department of Health and Mental Hygiene	Public Health Laboratory, NYC Department of Health and Mental Hygiene	Amin,H.S., Clabby,T.T., Wang,J.C., Taki,F., Su,M., Rahat.A., De La Cruz,N., Olsen,A., Thi,C., Silver,S., Akther,S., Chowdhury,M., Omoregie,E. and Hughes,S.
EPI ISL 17086062, EPI ISL 17086063, EPI ISL 17086064, EPI ISL 17086065, EPI ISL 17086066, EPI ISL 17086067, EPI ISL 17086068, EPI ISL 17086069, EPI ISL 17086070, EPI ISL 17086071, EPI ISL 17086072, EPI ISL 17086073, EPI ISL 17086074, EPI ISL 17086075, EPI ISL 17086076, EPI ISL 17086077, EPI ISL 17086078, EPI ISL 17086079, EPI ISL 17086080, EPI ISL 17086081, EPI=ISL) 7086082, EPI=ISL) 7086083, EPÜSL)7086084, EPI=JSL)7086086, EPI=ISL) 7086087, EPI=ISL) 7086088, EPI=ISL) 7086089, EPÜSL)7086090, EP(ISL) 7086091, EPI=ISL) 7086093, EPI=ISL) 7086095. EP(ISL)7086096, EPÜSL)7086097, EPI=ISL)7086098, EPI=ISL) 7086101, EPI=ISL) 7086102. EPI=ISL) 7086103, EPÜSL)7086104, EPI=ISL)7086105, EPI=ISL) 7086106, EPI ISL 17086107, EPI ISL 17086108, EPI ISL 17086109, EPI ISL 17086110, EPI ISL 17086111. EPI ISL 17086112, EPI ISL 17086113, EPI ISL 17086114, EPI ISL 17086115, EPI ISL 17086116, EPI ISL 17086117, EPI ISL 17086118, EPI ISL 17086119, EPI ISL 17086120, EPI ISL 17086121, EPI ISL 17086122, EPI ISL 17086123, EPI ISL 17086124, EPI ISL 17086125, EPI ISL 17086126, EPI-ISL-17086127, EPI-ISL-17086128, EPJ-ISL-17086129, EPI-ISL-17086130, EPI-ISL-17086131, EPI-ISL-17086132, EPI-ISL-17086133, EPI-ISL-17086134, EPI-ISL-17086135, EPI-ISL-17086136, EPI-ISL-17086137, EPI-ISL-17086138, EPI-ISL-17086139, EPI-ISL-17086140, EPI-ISL-17086141, EPI-ISL-17086142, EPI-ISL-17086143, EPI-ISL-17086144, EPI-ISL-17086145, EPI-ISL-17086146, EPI-ISL-17086147, EPI-ISL-17086148, EPJ-ISL-17086149, EPI-JSL-17086150, EPI-ISL-17086151, EPI-ISL-17086152, EPI-ISL-17086153, EPI-ISL-17086154, EPI-ISL-17086155, EPI-ISL-17086156, EPI-ISL-17086157, EPI-ISL-17086158, EPI-ISL-17086159, EPI-ISL-17086160, EPI-ISL-17086161, EPI-ISL-17086162, EPI-ISL-17086163, EPI-ISL-17086164, EPI-ISL-17086165, EPI-ISL-17086166, EPI-ISL-17086167, EPI-ISL-17086168, EPI-ISL-17086169, EPI-ISL-17086170, EPI-ISL-17086171, EPI-ISL-17086172, EPI-ISL-17086173, EPI-ISL-17086174, EPI-ISL-17086175, EPI-ISL-17086176, EPI-ISL-17086177, EPI-ISL-17086178, EPI-ISL-17086179, EPI-ISL-17086180, EPI-ISL-17086181, EPI-ISL-17086182, EPI-ISL-17086183, EPI-ISL-17086184, EPI-ISL-17086185, EPI-ISL-17086186, EPI=ISL) 7086187, EPI=ISL) 7086188, EPÜSL)7086189, EP(JSL) 7086190, EPI=ISL) 7086191, EPI=ISL) 7086192. EPI=ISL) 7086193, EPÜSL)7086194, EPI=ISL) 7086195, EPI=ISL) 7086196, EPI=ISL) 7086197. EP(ISL)7086198, EPÜSL)7086199, EP(ISL)7086200, EPI=ISL) 7086201, EPI=ISL) 7086202. EPI=ISL) 7086203, EPÜSL)7086204, EPI=ISL) 7086205, EPI=ISL) 7086206, EPI_ISL_l 7086207, EPI_ISL_l 7086208, EPI_ISL_l 7086209, EPI_ISL_l 7086210, EPI_ISL_l 7086211. EPI_ISL_l 7086212, EPI_ISL_l 7086213, EPI_ISL_l 7086214, EPI_ISL_l 7086215, EPI_ISL_l 7086216, EPI_ISL_l 7086217, EPI_ISL_l 7086218, EPI_ISL_l 7086219, EPI_ISL_17086232, EPI_ISL_l 7086233, EPI_ISL_l 7086234, EPI_ISL_l 7086235, EPI_ISL_l 7086236, EPI_ISL_17086237, EPI_ISL_l 7086238,			

EPI ISL 17086239, EPI ISL 17086240, EPI ISL 17086241, EPI ISL 17086242, EPI ISL 17086243, EPI ISL 17086244, EPI ISL 17086245, EPI ISL 17086246, EPI ISL 17086247, EPI ISL 17086248, EPI ISL 17086249, EPI ISL 17086250, EPI ISL 17086251, EPI ISL 17086252, EPI ISL 17086253, EPI ISL 17086254, EPI ISL 17086255, EPI ISL 17086256, EPI ISL 17086257, EPI ISL 17086258, EPI=ISL)7086259, EPI=ISL)7086260, EPÜSL)7086261, EPI=JSL)7086262, EPI=ISL)7086263, EPI=ISL)7086264, EPI=ISL)7086265, EPÜSL)7086266, EPI=ISL)7086267, EPI=ISL)7086268, EPI=ISL)7086269, EPI=ISL)7086270, EPÜSL)7086271, EPI=ISL)7086272 - - - - - - - - - - - -			
see above	Public Health Laboratory, NYC Department of Health and Mental Hygiene	Public Health Laboratory, NYC Department of Health and Mental Hygiene	Wang,J.C., Amin,H.S., Clabby,T.T., Taki,F., Su,M., Rahat,A., De La Cruz,N., Olsen,A., Thi,C., Silver,S., Akther,S., Chowdhury,M., Omoregie,E. and Hughes,S.
EPI ISL 17086274, EPI ISL 17086275, EPJ ISL 17086276, EPI JSL 17086277, EPI ISL 17086278, EPI ISL 17086279, EPI ISL 17086280, EPI ISL 17086281, EPI ISL 17086282, EPI ISL 17086283, EPI ISL 17086284, EPI ISL 17086285, EPI ISL 17086286, EPI ISL 17086287, EPI ISL 17086288, EPI ISL 17086289, EPI ISL 17086290, EPI ISL 17086291, EPI ISL 17086292, EPI ISL 17086293, EPI-ISL-17086294, EPI-ISL-17086295, EPI-ISL-17086296, EPI-ISL-17086297, EPI-ISL-17086298, EPI-ISL-17086299, EPI-ISL-17086300, EPI-ISL-17086301, EPI-ISL-17086302, EPI-ISL-17086304, EPI-ISL-17086305, EPI-ISL-17086306, EPI-ISL-17086307, EPI-ISL-17086308, EPI-ISL-17086309, EPI-ISL-17086310, EPI-ISL-17086311, EPI-ISL-17086312, EPI-ISL-17086313, EPI-ISL-17086314, EPI-ISL-17086315, EPI-ISL-17086316, EPI-ISL-17086317, EPI-ISL-17086318, EPI-ISL-17086319, EPI-ISL-17086320, EPI-ISL-17086321, EPI-ISL-17086322, EPI-ISL-17086323, EPI-ISL-17086324, EPI-ISL-17086325, EPI-ISL-17086326, EPI-ISL-17086327, EPI-ISL-17086328, EPI-ISL-17086329, EPI-ISL-17086330, EPI-ISL-17086331, EPI-ISL-17086332, EPI-ISL-17104181, EPI-ISL-17104182, EPCISL)7104183. EPI=ISL) 7104184, EPÜSL)7104185, EPCISL)7104186, EPI=ISL) 7104188, EPCISL)7104189. EPI=ISL) 7104190, EPÜSL)7104191, EPCISL) 7104192, EPI=ISL) 7104193, EPCISL)7104194. EPI=ISL) 7104195, EPÜSL)7104196, EPCISL)7104197, EPI=ISL) 7104198, EPCISL)7104200. EPI=ISL) 7104201, EPÜSL)7104203, EPCISL)7104204, EPI=ISL) 7104205, EPI ISL 17104206, EPI ISL 17104207, EPI ISL 17104208, EPI ISL 17104209, EPI ISL 17104210, EPI ISL 17104212, EPI ISL 17104213, EPI ISL 17104214, EPI ISL 17104215, EPI ISL 17104216, EPI ISL 17104217, EPI ISL 17104218, EPI ISL 17104219, EPI ISL 17104220, EPI ISL 17104221. EPI ISL 17104222, EPI ISL 17104224, EPI ISL 17104225, EPI ISL 17104226, EPI ISL 17104227, EPI-ISL-17104228, EPI-ISL-17104229, EPJ-ISL-17104230, EPI-JSL-17104231, EPI-ISL-17104232, EPI-ISL-17104233, EPI-ISL-17104236, EPI-ISL-17104237, EPI-ISL-17104238, EPI-ISL-17104239, EPI-ISL-17104240, EPI-ISL-17104241, EPI-ISL-17104242, EPI-ISL-17104243, EPI-ISL-17104244, EPI-ISL-17104245, EPI-ISL-17104247, EPI-ISL-17104248, EPI-ISL-17104249, EPI-ISL-17104250, EPI-ISL-17104251. EPI-ISL-17104252, EPI-ISL-17104253, EPI-ISL-17104254, EPI-ISL-17104255, EPI-ISL-17104256, EPI-ISL-17104257, EPI-ISL-17104259, EPI-ISL-17104260, EPI-ISL-17104261. EPI-ISL-17104262, EPI-ISL-17104263, EPI-ISL-17104264, EPI-ISL-17104265, EPI-ISL-17104266, EPI-ISL-17104267, EPI-ISL-17104268, EPI-ISL-17104269, EPI-ISL-17104271, EPI-ISL-17104272, EPI-ISL-17104273, EPI-ISL-17104274, EPI-ISL-17104275, EPI-ISL-17104276, EPI-ISL-17104277, EPI-ISL-17104278, EPI-ISL-17104279, EPI-ISL-17104280, EPI-ISL-17104281, EPI-ISL-17104282, EPI-ISL-17104284, EPI-ISL-17104285, EPI-ISL-17104286, EPI-ISL-17104287, EPI-ISL-17104288, EPI-ISL-17104350, EPI-ISL-17104352, EPI-ISL-17104353, EPI-ISL-17104354, EPI-ISL-17104355, EPI-ISL-17104356. EPI-ISL-17104357, EPJ-ISL-17104358, EPI-ISL-17104359, EPI-ISL-17104360, EPI-ISL-17104361. EPI-ISL-17104362, EPI-ISL-17104364, EPI-ISL-17104365, EPI-ISL-17104366, EPI-ISL-17104368. EPI-ISL-17104369, EPI-ISL-17104370, EPI-ISL-17104371, EPI-ISL-17104372, EPI-ISL-17104373. EPI-ISL-17104374, EPI-ISL-17104375, EPI-ISL-17104377, EPI-ISL-17104378, EPI-ISL-17104379, EPI-ISL-17104380, EPI-ISL-17104381, EPI-ISL-17104382, EPI-ISL-17104383, EPI-ISL-17104384, EPI-ISL-17104385, EPI-ISL-17104386, EPI-ISL-17104387, EPI-ISL-17104389, EPI-ISL-17104390, EPI-ISL-17104391, EPI-ISL-17104392, EPI-ISL-17104393, EPI-ISL-17104394, EPI-ISL-17104395, EPI-ISL-17104396, EPI-ISL-17104397, EPI-ISL-17104398, EPI-ISL-17104399, EPI=ISL)7104401, EPI=ISL)7104402, EPÜSL)7104403, EPI=JSL)7104404, EPI=ISL)7104405, EPI=ISL)7104406, EPI=ISL)7104407, EPÜSL) 7104409, EPI=ISL) 7104410, EPI=ISL)7104412, EPI=ISL)7104413, EPI=ISL)7104414, EPÜSL) 7104415, EPI=ISL)7104416, EPI=ISL)7104417, EPI=ISL)7104418, EPI=ISL)7104419, EPÜSL) 7104421, EPI=ISL)7104422, EPI=ISL)7104423, EPI ISL 17104425, EPI ISL 17104426, EPI ISL 17104427, EPI ISL 17104428, EPI ISL 17104429, EPI ISL 17104430, EPI ISL 17104431. EPI ISL 17104432, EPI ISL 17104433, EPI ISL 17104434, EPI ISL 17104435, EPI ISL 17104437, EPI ISL 17104438, EPI ISL 17104439, EPI ISL 17104440, EPI ISL 17104441. EPI ISL 17104442, EPI ISL 17104443, EPI ISL 17104444, EPI ISL 17104445, EPI-ISL-17104446, EPI-ISL-17104447, EPI-ISL-17104448, EPI-ISL-17104450, EPI-ISL-17104451, EPI-ISL-17104452, EPI-ISL-17104453, EPI-ISL-17104454, EPI-ISL-17104455, EPI-ISL-17104456, EPI-ISL-17104457, EPI-ISL-17104458, EPI-ISL-17104490, EPI-ISL-17104491, EPI-ISL-17104492, EPI-ISL-17104493, EPI-ISL-17104494, EPI-ISL-17104496, EPI-ISL-17104497, EPI-ISL-17104498, EPI-ISL-17104499. EPI-ISL-17104500, EPJ-ISL-17104501, EPI-ISL-17104502, EPI-ISL-17104503, EPI-ISL-17104504. EPI-ISL-17104506, EPI-ISL-17104507, EPI-ISL-17104508, EPI-ISL-17104509, EPI-ISL-17104510. EPI-ISL-17104511, EPI-ISL-17104512, EPI-ISL-17104513, EPI-ISL-17104514, EPI-ISL-17104515. EPI-ISL-17104516, EPI-ISL-17104517, EPI-ISL-17104518, EPI-ISL-17104521, EPI-ISL-17104522, EPI-ISL-17104523, EPI-ISL-17104524, EPI-ISL-17104525, EPI-ISL-17104526, EPI-ISL-17104527, EPI-ISL-17104528, EPI-ISL-17104529, EPI-ISL-17104530, EPI-ISL-17104531. EPI-ISL-17104533, EPI-ISL-17104534, EPI-ISL-17104535, EPI-ISL-17104536, EPI-ISL-17104537, EPI-ISL-17104538, EPI-ISL-17104539, EPI-ISL-17104540, EPI-ISL-17104541, EPI-ISL-17104542, EPI-ISL-17104543, EPI-ISL-17104545, EPJ-ISL-17104546, EPI-JSL-17104547, EPI-ISL-17104548, EPI-ISL-17104549, EPI-ISL-17104551, EPI-ISL-17104552, EPI-ISL-17104553, EPI-ISL-17104554, EPI-ISL-17104555, EPI-ISL-17104556, EPI-ISL-17104557, EPI-ISL-17104559, EPI-ISL-17104560, EPI-ISL-17104561, EPI-ISL-17104562, EPI-ISL-17104563, EPI-ISL-17104564, EPI-ISL-17104565, EPI-ISL-17104566, EPI-ISL-17104567, EPI-ISL-17104568, EPI-ISL-17104569, EPI-ISL-17104570, EPI-ISL-17104572, EPI-ISL-17104573, EPI-ISL-17104574, EPI-ISL-17104575, EPI-ISL-17104576, EPI-ISL-17104577, EPI-ISL-17104578, EPI-ISL-17104579, EPI-ISL-17104580, EPI-ISL-17104581, EPI-ISL-17104582, EPI-ISL-17104583, EPI-ISL-17104585, EPI-ISL-17104586, EPI-ISL-17104587, EPI-ISL-17104588, EPI-ISL-17104589, EPI-ISL-17104590, EPI-ISL-17104591, EPI-ISL-17104592, EPI-ISL-17104593, EPI-ISL-17104594, EPI-ISL-17104595, EPI-ISL-17104597, EPI-ISL-17104598, EPI-ISL-17104599, EPI-ISL-17104613, EPI-ISL-17104614, EPI-ISL-17104615, EPI-ISL-17104616, EPI-ISL-17104617, EPI-ISL-17104618, EPI-ISL-17104619, EPI-ISL-17104620, EPI-ISL-17104621, EPI=ISL)7104622. EPI=ISL)7104623, EPÜSL)7104625, EPI=ISL)7104626, EPI=ISL) 7104627, EPI=ISL) 7104628. EPI=ISL) 7104629, EPÜSL)7104630, EPI=ISL) 7104631, EPI=ISL)7104632, EPI=ISL) 7104633. EPI=ISL) 7104634, EPÜSL) 7104635, EPI=ISL)7104637, EPI=ISL) 7104638, EPI=ISL) 7104639. EPI=ISL) 7104641, EPÜSL) 7104642, EPI=ISL) 7104643, EPI=ISL) 7104644, EPI_ISL_I 7104645, EPI_ISL_l7104646, EPI_ISL_l7104647, EPI_ISL_l7104648, EPI_ISL_l7104649, EPI_ISL_l7104650, EPI_ISL_l7104651, EPI_ISL_I 7104652, EPI_ISL_l7104653, EPI_ISL_I 7104655, EPI_ISL_l7104656, EPI_ISL_I 7104657			
see above	Public Health Laboratory. NYC Department of Health and Mental Hygiene	Public Health Laboratory, NYC Department of Health and Mental Hygiene	Clabby,T.T.. Amin,H.S.. Wang,J.C., Taki,F., Su,M., Rahat,A., De la Cruz,N., Olsen,A., Thi,C., Silver,S., Akther,S., Chowdhury,M., Omoregie,E. and Hughes,S.
EPI ISL 17118598, EPI ISL 17118599, EPI ISL 17118601, EPI ISL 17118602, EPI ISL 17118603, EPI ISL 17118604, EPI ISL 17118605, EPI ISL 17118606, EPI ISL 17118607, EPI ISL 17118608, EPI ISL 17118609, EPI ISL 17118610, EPI ISL 17118611, EPI ISL 17118612, EPI ISL 17118613, EPI ISL 17118614, EPI ISL 17118615, EPI ISL 17118616, EPI ISL 17118617, EPI ISL 17118618, EPI-ISL-17118619, EPI-ISL-17118620, EPJ-ISL-17118621, EPI-JSL-17118622, EPI-ISL-17118623, EPI-ISL-17118624, EPI-ISL-17118625, EPI-ISL-17118626, EPI-ISL-17118627, EPI-ISL-17118628, EPI-ISL-17118629, EPI-ISL-17118630, EPI-ISL-17118631, EPI-ISL-17118632, EPI-ISL-17118633, EPI-ISL-17118634, EPI-ISL-17118635, EPI-ISL-17118636, EPI-ISL-17118637, EPI-ISL-17118638, EPCISL)7118639. EPI=ISL)7118640, EPÜSL)7118641, EPCISL)7118642, EPI=ISL)7118643, EPCISL)7118644. EPI=ISL)7118645, EPÜSL)7118646, EPCISL)7118647, EPI=ISL)7118648, EPCISL)7118649. EPI=ISL)7118650, EPÜSL)7118651, EPCISL)7118652, EPI=ISL)7118653, EPCISL)7118654. EPI=ISL)7118655, EPÜSL)7118656, EPCISL)7118657, EPI=ISL)7118659, EPI ISL 17118660, EPI ISL 17118661, EPI ISL 17118662, EPI ISL 17118663, EPI ISL 17118664, EPI ISL 17118665, EPI ISL 17118666, EPI ISL 17118667, EPI ISL 17118668, EPI ISL 17118669, EPI ISL 17118670, EPI ISL 17118671, EPI ISL 17118672, EPI ISL 17118673, EPI ISL 17118674, EPI ISL 17118675, EPI ISL 17118676, EPI ISL 17118677, EPI ISL 17118678, EPI ISL 17118679, EPI=ISL)7118680, EPI=ISL)7118681, EPÜSL)7118682, EPI=ISL)7118683, EPI=ISL)7118684, EPI=ISL)7118685, EPI=ISL)7118686, EPÜSL)7118687, EPI=ISL)7118688, EPI=ISL)7118689, EPI=ISL)7118690, EPI=ISL)7118691, EPÜSL)7118692, EPI=ISL)7118693, EPI=ISL)7118694, EPI=ISL)7118695, EPI=ISL)7118696, EPÜSL)7118697 - - - -			
see above	Public Health Laboratory, NYC Department of Health and Mental Hygiene	Public Health Laboratory, NYC Department of Health and Mental Hygiene	Wang,J.C., Amin,H.S., Clabby,T.T., Taki,F., Su,M., Rahat,A., De La Cruz,N., Olsen,A., Thi,C., Silver,S., Akther,S., Chowdhury,M., Omoregie,E. and Hughes,S.
EPI_ISL_l7118698, EPI_ISL_l7118699, EPI_ISL_l7118700, EPI_ISL_l7118701, EPI_ISL_l7118702, EPI_ISL_l7118703, EPI_ISL_l7118704, EPI_ISL_I 7118705, EPI_ISL_I 7118706, EPI_ISL_I 7118707, EPI_ISL_l7118708, EPI_ISL_l7118709, EPI_ISL_I 7118710, EPI_ISL_l7118711, EPI_ISL_l7118712, EPI_ISL_l7118713, EPI_ISL_l7118714, EPI_ISL_I 7118715, EPI_ISL_l7118716, EPI_ISL_l7118717, EPI_ISL_I 7118718, EPI_ISL_I 7118719, EPI_ISL_I 7118720, EPI_ISL_I 7118721, EPI_ISL_I 7118722, EPI_ISL_I 7118723, EPI_ISL_I 7118724, EPI_ISL_I 7118725, EPI_ISL_I 7118726, EPI_ISL_I 7118727, EPI_ISL_I 7118728, EPI_ISL_I 7118729, EPI_ISL_I 7118730, EPI_ISL_l7118731, EPI_ISL_I 7118732, EPI_ISL_I 7118733			
see above	Laboratory Medicine, UW Virology	Laboratory Medicine, UW Virology	Sereewit,J., Xie,H.. Roychoudhury.P. and Greninger.A.L.
EPI_ISL_l7118734, EPI_ISL_I 7118735, EPI_ISL_l7118736, EPI_ISL_I 7118737	Environmental, Agricultural, and Occupational Health, University of Nebraska Medical Center	Environmental, Agricultural, and Occupational Health, University of Nebraska Medical Center	Gigante,C., Wang,Y., Zhao,H., Batra,O., Hetrick,E., Howard,☐., Kovar,L., Seabolt,M., Knipe,K., Burroughs,M.S., Lee,J., Wilkins,K., McCollum,A., Hutson,C., Oavidson,W., Rao,A., Stone,). and Li,Y.
EPI_ISL_I 7118738, EPI_ISL_I 7118739	Environmental, Agricultural, and Occupational Health, University of Nebraska Medical Center	Environmental, Agricultural. and Occupational Health, University of Nebraska Medical Center	Pentella,M., Chapman,R.C.. Stapleton,J., Meier.)., Xiang.j., Li,M., Reeb.V., Benfer,J.. Eveland,K., Wiley,M.R., Hottel,W. and Cross,S.T.
EPI_ISL_l7152764	Centers for Disease Contrai, R.O.C. (Taiwan)	Centers for Disease Contrai, R.O.C. (Taiwan)	Lin JH, Chiu SC, Huang Hl. Huang WL, Li TY, Fann WB, Hsieh PY, Yang JY
EPI_ISL_l 7152842	Centers for Oisease Contrai, R.O.C. (Taiwan)	Centers for Oisease Contrai, R.O.C. (Taiwan)	Lin JH, Chiu SC, Huang Hl, Huang LW, Li TY, Fann WB, Hsieh PY, Yang JY
EPI_ISL_l 7152843	Centers for Oisease Contrai, R.O.C. (Taiwan)	Centers for Oisease Contrai, R.O.C. (Taiwan)	Lin JH, Chiu SC, Huang Hl, Huang WL, Li TY, Fann WB, Hsieh PY, Yang JY
EPI ISL 17156262, EPI ISL 17156263, EPJ ISL 17156264, EPI JSL 17156265, EPI ISL 17156266, EPI ISL 17156267, EPI ISL 17156268, EPI ISL 17156269, EPI ISL 17156270, EPI ISL 17156271, EPI ISL 17156272, EPI ISL 17156273, EPI ISL 17156274, EPI ISL 17156275, EPI ISL 17156276, EPI ISL 17156277, EPI ISL 17156278, EPI ISL 17156279, EPI ISL 17156280, EPI ISL 17156281, EPI=ISL)7156282. EPI=ISL)7156283, EPÜSL)7156284, EPI=ISL)7156285, EPI=ISL)7156286, EPI=ISL)7156287. EPI=ISL)7156288, EPÜSL)7156289, EPI=ISL)7156290, EPI=ISL)7156291 - - - - - - - - - - - - - - - - - -			
see above	Centre for Biological Threats, Highly Pathogenic Viruses, Robert Koch Institute	Centre for Biological Threats, Highly Pathogenic Viruses, Robert Koch Institute	Brinkmann,A., Kohl,C., Pape,K., Schrick,L., Michel,)., Schaade,L. and Nitsche,A.
EPI_ISL_l7165661	Division of High Consequence Pathogens and Pathology (DHCPP) - Poxvirus and Rabies Branch (PRB), CDC	Division of High Consequence Pathogens and Pathology (DHCPP) - Poxvirus and Rabies Branch (PRB), COC	Gigante,C., Kubin,G., Zhao,H., Batra,O., Hetrick,E., Howard,☐.,Kovar,L., Seabolt,M., Morrison,S., Oesch,M., Knipe,K., Weigand,M., Cintron,R., Sheth,M., Burgin,A., Burroughs,M., Lee,J., Wilkins,K., McCollum,A., Hutson,C., Oavidson,W., Rao.A., White,S. and Li,Y.
EPI_ISL_l7165662	Division of High Consequence Pathogens and Pathology (OHCPP) - Poxvirus and Rabies Branch (PRB), CDC	Division of High Consequence Pathogens and Pathology (OHCPP) - Poxvirus and Rabies Branch (PRB), COC	Gigante,C. Ventura,J., Zhao,H., Batra,O., Hetrick,E., Howard,☐.,Kovar,L., Seabolt,M., Morrison,S., Desch,M., Knipe,K., Weigand,M, Cintron,R., Sheth,M., Burgin,A., Burroughs,M., Lee,J., Wilkins,K., McCollum,A., Hutson,C., Davidson,W., Rao.A., Nash,J. and Li,Y.
EPI_ISL_l7165663	Division of High Consequence Pathogens and Pathology (DHCPP) - Poxvirus and Rabies Branch (PRB), CDC	Division of High Consequence Pathogens and Pathology (DHCPP) - Poxvirus and Rabies Branch (PRB), COC	Gigante,C., Steidley,B., Zhao,H., Batra,D., Hetrick,E., Howard,☐., Kovar,L., Seabolt,M., Morrison,S., Desch,M., Knipe,K., Weigand,M , Cintron,R., Sheth,M., Burgin,A., Burroughs,M., Lee,J., Wilkins,K., McCollum,A., Hutson,C., Oavidson,W., Rao.A., Oavizon,E. and li.Y.
EPJ_ISL_l 7165664, EPI_JSL_l 7165665	Division of High Consequence Pathogens and Pathology (DHCPP) Poxvirus and Rabies Branch (PRB). CDC	Division of High Consequence Pathogens and Pathology (DHCPPJ - Poxvirus and Rabies Branch (PRB), *CDC*	Gigante,C., Alexander,S., Zhao,H., Batra,O., Hetrick,E., Howard,☐.,Kovar,L., Seabolt.M , Morrison,5., Desch,M., Knipe,K., Weigand,M , Cintron,R., Sheth,M., Burgin,A., Burroughs,M., Lee,J., Wilkins,K., McCollum,A., Hutson,C., Davidson,w., Rao,A., Pinnick,D. and Li,Y.
EPI_ISL_l7165666, EPI_ISL_l 7165667	Division of High Consequence Pathogens and Pathology (DHCPP) - Poxvirus and Rabies Branch (PRB), CDC	Division of High Consequence Pathogens and Pathology (DHCPP) - Poxvirus and Rabies Branch (PRB), COC	Gigante,C.M., Benton,C., Zhao,H., Batra,O., Hetrick,E.E., Howard,O.T., Kovar,L., Seabolt,M.H., Morrison,S.S., Oesch,M., Knipe,K., Weigand,M.R., Cintron,R., Sheth,M., Burgin,A., Burroughs,M., Lee,J., Wilkins,K., McCollum,A., Hutson,C., Oavidson,W., Rao.A., Bonner,K.O. and Li,Y.
EPI ISL 17165668, EPI ISL 17165669, EPI ISL 17165670 - - - - - -	Division of High Consequence Pathogens and Pathology (OHCPP) - Poxvirus and Rabies Branch (PRB). CDC	Division of High Consequence Pathogens and Pathology (OHCPP) - Poxvirus and Rabies Branch (PRB), COC	Gigante,C., Ruiz.V., Zhao,H., Batra,D., Hetrick,E., Howard,☐.,Kovar,L., Seabolt,M., Morrison,S., Oesch,M, Knipe,K., Weigand,M, Cintron,R., Burgin,A., Burroughs,M., Lee,J., Wilkins,K., McCollum,A., Hutson,C., Davidson,W., Rao.A., Wang.J. and li,Y.
EPI_ISL_l7165671, EPI_ISL_17165672, EPI_ISL_l7165673, EPI ISL 17165674, EPI ISL 17165675, EPI ISL 17165676, - - EPI_ISL_l716567-7, EPI_ISL_l716567-8	Division of High Consequence Pathogens and Pathology (DHCPP) - Poxvirus and Rabies Branch (PRB), CDC	Division of High Consequence Pathogens and Pathology (DHCPP) - Poxvirus and Rabies Branch (PRB), COC	Gigante,C., Kubin,G., Zhao,H., Batra,O., Hetrick,E., Howard,☐.,Kovar,L., Seabolt,M., Morrison,S., Oesch,M., Knipe,K., Weigand,M., Cintron,R., Sheth,M., Burgin,A., Burroughs,M., Lee,J., Wilkins,K., McCollum,A., Hutson,C., Oavidson,W., Rao.A., White,S. and li.Y.
EPI ISL 17165679, EPI ISL 17165680, EPI ISL 17165681, EPI ISL 17165683, EPI ISL 17165684, EPI ISL 17165685, EPI ISL 17165686, EPI ISL 17165687, EPI ISL 17165688, EPI ISL 17165689, EPI ISL 17165690, EPI ISL 17165691, EPI ISL 17165692, EPI ISL 17165693, EPI ISL 17165694, EPI ISL 17165695, EPI ISL 17165696, EPI ISL 17165697, EPI ISL 17165698, EPI ISL 17165699, EPI=ISL)7165700, EPI=ISL)7165701, EPÜSL)7165702, EPI=ISL)7165703, EPI=ISL)7165704, EPI=ISL)7165705, EPI=ISL)7165707, EPÜSL)7165708, EPI=ISL)7165709, EPI=ISL)7165710, EPI=ISL)7165711, EPI=ISL)7165712, EPÜSL)7165713, EPI=ISL)7165714, EPI=ISL)7165715, EPI=ISL)7165716, EPI=ISL)7165717, EPÜSL)7165718, EPI=ISL)7165719, EPI=ISL)7165720, EPI ISL 17165721, EPI ISL 17165722, EPJ ISL 17165723, EPI JSL 17165724, EPI ISL 17165725, EPI ISL 17165726, EPI ISL 17165727, EPI ISL 17165728, EPI ISL 17165729, EPI ISL 17165730, EPI ISL 17165731, EPI ISL 17165732, EPI ISL 17165733, EPI ISL 17165734, EPI ISL 17165735, EPI ISL 17165736, EPI ISL 17165737, EPI ISL 17165738, EPI ISL 17165739, EPI ISL 17165740, EPI-ISL-17165741, EPI-ISL-17165742, EPI-ISL-17165743, EPI-ISL-17165744, EPI-ISL-17165745, EPI-ISL-17165746, EPI-ISL-17165747, EPI-ISL-17165748, EPI-ISL-17165749, EPI-ISL-17165750, EPI-ISL-17165751, EPI-ISL-17165752, EPI-ISL-17165753, EPI-ISL-17165754, EPI-ISL-17165755, EPI-ISL-17165756, EPI-ISL-17165757, EPI-ISL-17165758, EPI-ISL-17165759, EPI-ISL-17165760, EPC1sL)7165761. EP1=1sL)7165762, EPÜSL)7165763. EPCJsL)7165764, EP1=1sL)7165765, EPC1sL)7165766. EP1=1sL)7165767, EPÜSL)7165768. EPC1sL)7165769, EP1=1sL)7165770, EPC1sL)7165771. EP1=1sL)7165772, EPÜSL)7165773. EP1=1sL)7165774, EP1=1sL)7165775, EPC1sL) 7165776. EP1=1sL)7165777, EPÜSL)716577B - - - -			
see above	Public Health Laboratory, NYC Oepartment of Health and Mental Hygiene (DOHMH)	Public Health Laboratory, NYC Oepartment of Health and Mental Hygiene (DOHMH)	Clabby,T.T., Amin,H.S., Wang,J.C., Taki,F., Su,M., Rahat,A., De La Cruz,N., Olsen,A., Thi,C., Silver,S., Akther,S., Chowdhury,M., Omoregie,E. and Hughes,S.
EPI_ISL_l7170656. EPI_ISL_l7170657, EPJ_ISL_l 7170658. EPI_JSL_I 7170659, EPI_ISL_l7170660, EPI_ISL_l7170661. EPI_ISL_l7170662, EPI_ISL_l7170663. EPI_ISL_l7170664, EPI_ISL_l7170665, EPI_ISL_l7170666. EPI_ISL_l7170667, EPI_ISL_I 7170668. EPI_ISL_17170669, EPI_ISL_l7170670, EPI_ISL_l 7170671			
see above	California Oepartment of Public Health	California Oepartment of Public Health	Haw, M., Kath, C., Espinosa, A., O'Neil, R., and Hacker, J.
EPI_ISL_17179627, EPI_ISL_17179628	St Jame·s Hospital, Virology Department	National Virus Reference Laboratory	Patrice Keane, Yvonne Lynagh, Brendan Crowley, Gabriel Gonzalez, Michael Carr, Emer O'Byrne, Weronika Banka, Brian Keogan, Jose Maria Urtasun Elizari, Jonathan Dean, Daniel Hare, Cillian F De Gascun
EPI ISL 17179629, EPI ISL 17179630, EPI ISL 17179631, - - EP1-=_1sL-=.l7179632 - -	National Virus Reference Laboratory	National Virus Reference Laboratory	Gabriel Gonzalez, Michael Carr, Emer O'Byrne, Weronika Banka, Brian Keogan, Jose Maria Urtasun Elizari, Jonathan Dean, Daniel Hare, Cillian F De Gascun
EPI_ISL_l 7179633	St Jame·s Hospital, Virology Department	National Virus Reference Laboratory	Patrice Keane, Yvonne Lynagh, Brendan Crowley, Gabriel Gonzalez, Michael Carr, Emer O'Byrne, Weronika Banka, Brian Keogan, Jose Maria Urtasun Elizari, Jonathan Dean, Daniel Hare, Cillian F De Gascun
EPI ISL 17179634, EPI ISL 17179635, EPI ISL 17179636, - - EP1-=_1sL-=.17179637 - -	National Virus Reference Laboratory	National Virus Reference Laboratory	Gabriel Gonzalez, Michael Carr, Emer O'Byrne, Weronika Banka, Brian Keogan, Jose Maria Urtasun Elizari, Jonathan Dean, Daniel Hare, Cillian F De Gascun
EPI ISL 17179638, EPI ISL 17179639, EPI ISL 17179640, EPl_1sL-=.17179641, EP1-=.1sL-=.17179642, EP1-=_1sL-=.17179643	St Jame's Hospital, Virology Department	National Virus Reference Laboratory	Patrice Keane, Yvonne Lynagh, Brendan Crawley, Gabriel Gonzalez, Michael Carr, Emer O'Byrne, Weronika Banka, Brian Keogan, Jose Maria Urtasun Elizari, Jonathan Dean, Daniel Hare, Cillian F De Gascun
EPJ_ISL_l 7187497, EPI_ISL_l 7187498	Vajira Hospital	Thai Red Cross Emerging Infectious Diseases Clinical Center and Faculty of Medicine, Chulalongkorn University	Suppasit srisaeng, Praepoly Ruekmuang, Kusuma Swangpun, Arriya Panchaiyaphum, Pakita Salaeh, Natpusda Kongmaung. Pornsiri limwattanawong, Noree Pholprasert, Montriya Unteamsorn, Kanjana Jeknok, Withak Withaksabut, Sunisa Nilda. Artorn Niakul, Sopon lamsirithaworn, Thitipong Yingyong, Rossaporn Kittiyaowamarn, Rome Buathong, Ratanaporn Tangwangvivat, Supaporn Wacharapluesadee, Sininat Petcharat. Ananporn Supataragul, Stefan Fernandez, Achawin Rojanaviwat. Chonticha Klungthong, Pilailuk Okada, Khajohn Joonlasak, Chakkarat Pitayawonganon, Opass Putcharoen
EPI_ISL_l 7187499	Department of Disease Contrai, Ministry of Public Health	Thai Red Cross Emerging Infectious Diseases Clinical Center and Faculty of Medicine, Chulalongkorn University	Suppasit srisaeng, Praepoly Ruekmuang, Kusuma Swangpun, Arriya Panchaiyaphum, Pakita Salaeh, Natpusda Kongmaung, Pornsiri limwattanawong, Noree Pholprasert, Montriya Unteamsorn, Kanjana Jeknok, Withak Withaksabut, Sunisa Nilda, Artorn Niakul, Sopon larnsirithaworn, Thitipong Yingyong, Rossaporn Kittiyaowarnarn, Rome Buathong, Ratanaporn Tangwangvivat, Supaporn wacharapluesadee. Sininat Petcharat, Ananporn Supataragul, Stefan Fernandez, Achawin Rojanaviwat. Chonticha Klungthong, Pilailuk Okada, Khajohn Joonlasak, Chakkarat Pitayawonganon, Opass Putcharoen
EPJ_ISL_l 7187500, EPI_JSL_l7187501	Bangkok Hospital Phuket	Thai Red Cross Emerging Infectious Oiseases Clinical Center and Faculty of Medicine, Chulalongkorn University	Nungrathai Srisong, Praepoly Ruekrnuang, Kusurna Swangpun, Arriya Panchaiyaphurn, Pakita Salaeh, Natpusda Kongrnaung, Pornsiri limwattanawong, Noree Pholprasert, Montriya Unteamsorn, Kanjana Jeknok, Withak Withaksabut, Sunisa Nilda, Artorn Niakul, Sopon lamsirithaworn, Thitipong Yingyong, Rossaporn Kittiyaowamarn, Rome Buathong, Ratanaporn Tangwangvivat, Supaporn Wacharapluesadee, Sininat Petcharat, Ananporn Supataragul, Stefan Fernandez, Achawin Rojanaviwat. Chonticha Klungthong, Pilailuk Okada, Khajohn Joonlasak, Chakkarat Pitayawonganon, Opass Putcharoen
EPI_ISL_l7187502	Department of Disease Contrai, Ministry of Public	Thai Red Cross Emerging Infectious Diseases Clinical	Supanut Chotichavalrattanakul, , Praepoly Ruekmuang, Kusuma Swangpun, Arriya Panchaiyaphum, Pakita Salaeh, Natpusda Kongmaung, Pornsiri Limwattanawong, Noree Pholprasert, Montriya Unteamsorn, Kanjana Jeknok, Withak

	Health	Center and Faculty of Medicine, Chulalongkorn University	Withaksabut, Sunisa Nilda, Artorn Niakul, Sopon lamsirithaworn, Thitipong Yingyong, Rossaporn Kittiyaowamarn, Rome Buathong, Ratanaporn Tangwangvivat. Supaporn Wacharapluesadee, Sininat Petcharat. Ananporn Supataragul, Stefan Fernandez, Achawin Rojanaviwat, Chonticha Klungthong, Pilailuk Okada. Khajohn Joonlasak, Chakkarat Pitayawonganon, Opass Putcharoen
EPJ_ISL_l7187503, EPI_ISL_l 7187504	Suvarnabhumi Airport	Thai Red Cross Emerging Infectious Diseases Clinical Center and Faculty of Medicine, Chulalongkorn University	Phawinee Montri, Praepoly Ruekmuang, Kusuma Swangpun, Arriya Panchaiyaphum, Pakita Salaeh, Natpusda Kongmaung, Pornsiri Limwattanawong, Noree Pholprasert, Montriya Unteamsom, Kanjana Jeknok, Withak Withaksabut, Sunisa Nilda, Artorn Niakul, Sopon lamsirithaworn, Thitipong Yingyong, Rossaporn Kittiyaowamarn, Rome Buathong, Ratanaporn Tangwangvivat, Supaporn Wacharapluesadee. Sininat Petcharat. Ananporn Supataragul, Stefan Fernandez, Achawin Rojanaviwat, Chonticha Klungthong, Pilailuk Okada, Khajohn Joonlasak, Chakkarat Pitayawonganon, Opass Putcharoen
EPI_ISL_l7187505	Phuket Provincial Public Health Office	Thai Red Cross Emerging Infectious Diseases Clinical Center and Faculty of Medicine, Chulalongkorn University	Nungrathai Srisong, Praepoly Ruekmuang, Kusuma Swangpun, Arriya Panchaiyaphum, Pakita Salaeh, Natpusda Kongmaung, Pornsiri Limwattanawong, Noree Pholprasert, Montriya Unteamsom, Kanjana Jeknok, Withak Withaksabut. Sunisa Nilda, Artorn Niakul, Sopon lamsirithaworn, Thitipong Yingyong, Rossaporn Kittiyaowamarn, Rome Buathong, Ratanaporn Tangwangvivat, Supaporn Wacharapluesadee. Sininat Petcharat, Ananporn Supataragul, Stefan Fernandez, Achawin Rojanaviwat. Chonticha Klungthong, Pilailuk Okada, Khajohn Joonlasak. Chakkarat Pitayawonganon. Opass Putcharoen
EPI_ISL_l 7201439, EPI_ISL_l7201440, EPI_ISL_l7201441	Genomics Division, Instituto Tecnologico y de Energias Renovables (ITER)	Genomics Division, Instituto Tecnologico y de Energias Renovables (ITER)	Munoz-Barrera,A., Ciuffreda,L. Alcoba-Florez,J, Rubio-Rodriguez,L.A., Rodriguez-Perez,H., Gil-Campesino,H., Garcia-Martinez de Artola,D., Salas-Hernandez,J, Rodriguez-Nunez,J, Inigo-Campos,A. Garcia-Olivares.V., Diez-Gil,O., Gonzalez-Montelongo,R, Valenzuela-Fernandez,A., Lorenzo-Salazar,J.M. and Flores,C.
EPI_ISL_l 7201443	Genomics Division. Instituto Tecnologico y de Energias Renovables (ITER)	Genomics Division, Instituto Tecnologico y de Energias Renovables (ITER)	Munoz Barrera.A., Ciuffreda,L. Alcoba-Florez,J , Rubio-Rodriguez,L.A., Rodriguez-Perez.H., Gil-Campesino,H.. Garcia-Martinez de Artola,D.. Salas-Hernandez,J . Rodriguez-Nunez,J , Inigo-Campos,A. Garcia-Olivares.V.. Diez-Gil,O.. Gonzalez-Montelongo,R., Valenzuela-Fernandez,A., Lorenzo-Salazar,J.M. and Flores,C.
EPI_ISL_l 7201444	Genomics Division, Instituto Tecnologico y de Energias Renovables (ITER)	Genomics Division, Instituto Tecnologico y de Energias Renovables (ITER)	Munoz-Barrera,A. Ciuffreda,L. Alcoba-Florez,J, Rubio-Rodriguez,L.A., Rodriguez-Perez,H.. Gil-Campesino,H.. Garcia-Martinez de Artola,D.. Salas-Hernandez,J, Rodriguez-Nunez,J, Inigo-Campos,A, Garcia-Olivares.V.. Diez-Gil.O.. Gonzalez-Montelongo,R., Valenzuela-Fernandez,A., Lorenzo-Salazar,J.M. and Flores,C.
EPI_ISL_l7206607, EPI_ISL_l7206608, EPI_ISL_l7206609, EPI_ISL_l7206610, EPI_ISL_l7206611. EPI_ISL_l7206612, EPI_ISL_l7206613, EPI_ISL_l 7206614, EPI_ISL_l7206615, EPI_ISL_l7206616, EPI_ISL_l7206617, EPI_ISL_l7206618, EPI_ISL_l 7206619, EPI_ISL_l7206620			
see above	California Department of Public Health	California Department of Public Health	Haw,M., Kath,C.. Espinosa,A.. O'Neil.R.. and Hacker,J.
EPI_ISL_l 7206621, EPI_ISL_l 7206622	California Department of Public Health	California Department of Public Health	Kath, C , Haw, M.. Espinosa, A.. and Hacker, J.
EPI_ISL_l7211323	Los Angeles County Public Health Laboratories	Los Angeles County Public Health Laboratories	P. Hemarajata et al.
EPI ISL 17211324, EPI ISL 17211325, EPI ISL 17211326, EPI=ISL)7211327. EPI=ISL)7211328, EPI=ISL)7211329, EPI_ISL_l 7211330	Kaiser Permanente Chino Hills Regional Reference Laboratories	Los Angeles County Public Health Laboratories	P. Hemarajata et al.
EPI_ISL_l 7211331	Los Angeles County Public Health Laboratories	Los Angeles County Public Health Laboratories	P. Hemarajata et al.
EPI_ISL_l 7211332	Ouest Diagnostics Nichols Institute	Los Angeles County Public Health Laboratories	P. Hemarajata et al.
EPI_ISL_l7211333	Los Angeles County Public Health Laboratories	Los Angeles County Public Health Laboratories	P. Hemarajata et al.
EPI_ISL_l 7211335	Laboratory Corporation of America	Los Angeles County Public Health Laboratories	P. Hemarajata et al.
EPI_ISL_l 7222811, EPI_ISL_l7222812, EPI_ISL_l7222813, EPI_ISL_l7222814, EPI_ISL_l7222816, EPI_ISL_l7222817, EPI_ISL_l7222818, EPI_ISL_l7222819, EPI_ISL_l 7222820, EPI_ISL_l7222822, EPI_ISL_l7222823, EPI_ISL_l7222824, EPI_ISL_l 7222825, EPI_ISL_l7222827, EPI_ISL_l7222828, EPI_ISL_l7222829			
see above	Viral and Rickettsial Disease Laboratory (VRDL) California Department of Public Health (CDPH)	Viral and Rickettsial Disease Laboratory (VRDL) California Department of Public Health (CDPH)	Haw,M., Kath,C., Espinosa,A.. O'Neil,R. and Hacker.J.
EPI ISL 17246657, EPI ISL 17246659	Fumi Kasuya Tokyo Metropolitan Institute of Public Health, Department of Microbiology	Fumi Kasuya Tokyo Metropolitan Institute of Public Health, Department of Microbiology	Kasuya,F., Negishi,A., Kumagai,R.. Hasegawa,M.. Fujiwara,T.. Miyake,H., Nagashima,M. and Sadamasu,K.
EPI ISL 17269833, EPI ISL 17269834, EPI ISL 17269835, EPI=ISL) 7269836, EPI=ISL) 7269837, EPI=ISL)7269838, EPI_ISL_l 7269839	Environmental, Agricultural, and Occupational Health, University of Nebraska Medical Center	Environmental, Agricultural, and Occupational Health, University of Nebraska Medical Center	Tegomoh,B., Cross,S.T., Chapman,R.C., Bernhard,K., McCutchen,E.L., Fauver,J.R., Pratt,C.8., Warden,D.E., Iwen,P.C., Donahue,M. and Wiley,M.R.
EPI_ISL_17271956, EPI_ISL_172719S7	Rhode Island State Health Laboratory	Rhode Island State Health Laboratory	Kristin Carpenter-Azevedo, Sean Sierra-Patev, Richard C. Huard
EPI ISL 17319546, EPI ISL 17319547, EPI ISL 17319548, EPCISL)7319549, EPI=ISL)7319550, EPI=ISL)7319551. EPI_ISL_l7319554, EPI_ISL_l7319555	Department of Clinical Sciences, Institute of Tropical Medicine	Department of Clinical Sciences, Institute of Tropical Medicine	Mertes,H., Rezende,A.M., Naesens,R.. de Block,T., Michiels,J., Coppens,)., Van Dijck,C., Bomans,P.. Arien,K., Bottieau,E.. Van Esbroeck,M., Liesenborghs,L. and Vercauteren,K.
EPI ISL 17319556	Center of Diagnostics and Vaccine Development. Centers for Disease Control	Center of Diagnostics and Vaccine Development. Centers for Disease Control	Lin,J.-H., Chiu,S.-C., Huang,H.-1., Huang,W.-l.. Li,T.-Y., Fann,W.-B.. Hsieh,P.-Y. and Yang,J.-Y.
EPI_ISL_l7383630, EPI_ISL_l7383632, EPI_ISL_l7383634, EPI_ISL_l7383635, EPI_ISL_l7383636, EPI_ISL_17383637, EPI_ISL_l7383639, EPI_ISL_l 7383641, EPI_ISL_l7383645	Laboratorio Departamental de Salud Publica de Antioquia	Laboratorio Departamental de Salud Publica de Antioquia	Betancur,I.I.B., Velarde Hoyos,C.A.C.V., Gomez,R.R.G. and Mercado-Reyes,M.M.R.
EPI ISL 17390796, EPI ISL 17390797, EPI ISL 17390799. EPC_ISL l 7390801, EPI ISL l7390803, EPI ISL l7390804	Antioquia, Laboratorio Departamental de Salud Publica de Antioquia	Antioquia. Laboratorio Departamental de Salud Publica de Antioquia	Betancur.1.1.B.. Velarde Hoyos,C.A.C.V., Gomez,R.R.G. and Mercado-Reyes,M.M.R.
EPJ_ISL_l7394091. EPI_JSL_l 7394092	Division of High-Consequence Pathogens and Pathology - Poxvirus and Rabies Branch (DHCPP-PRB). Centers for Disease Control and Prevention (CDC)	Division of High-Consequence Pathogens and Pathology - Poxvirus and Rabies Branch (DHCPP-PRB), Centers for Disease Contrai and Prevention (CDC)	Gigante,(, Ostadkar,R., Zhao,H., Batra,D., Hetrick,E., Howard,□., Kovar,L., Seabolt,M., Morrison,S., Desch,M., Knipe,K., Weigand,M, Cintron,R., Burgin,A., Burroughs,M., Lee,J., Wilkins,K., McCollum,A., Hutson,C., Davidson,W., Rao.A., Wang.X. and Li.Y.
EPI_ISL_l 7394093	Division of High-Consequence Pathogens and Pathology - Poxvirus and Rabies Branch (DHCPP-PRB). Centers for Disease Control and Prevention (CDC)	Division of High-Consequence Pathogens and Pathology Poxvirus and Rabies Branch (DHCPP-PRB), Centers for Disease Control and Prevention (CDC)	Gigante,C., Wang.Y., Zhao,H., Batra,D., Hetrick,E., Howard,□.,Kovar,L., Seabolt,M., Morrison,S., Desch,M., Knipe,K., Weigand,M, Cintron,R., Burgin,A., Burroughs,M., Lee,J., Wilkins,K., McCollum,A., Hutson,C., Davidson,W., Rao.A., O'Dell,J. and Li,Y.
EPI_ISL_l 7394094	Division of High-Consequence Pathogens and Pathology - Poxvirus and Rabies Branch (DHCPP-PRB), Centers for Disease Control and Prevention (CDC)	Division of High-Consequence Pathogens and Pathology - Poxvirus and Rabies Branch (DHCPP-PRB), Centers for Disease Control and Prevention (CDC)	Gigante,C., Cogswell.K.. Zhao.H.. Batra,D., Hetrick,E., Howard.D., Kovar,L., Seabolt,M.. Morrison.S., Desch,M.. Knipe,K.. Weigand,M Cintron.R., Burgin,A., Burroughs,M.. Lee.J., Wilkins.K., McCollum,A.. Hutson,C., Davidson,W., Rao.A., Grenz,L. and Li.Y.
EPI_ISL_l 7394095	Division of High-Consequence Pathogens and Pathology - Poxvirus and Rabies Branch (DHCPP-PRB), Centers for Disease Control and Prevention (CDC)	Division of High-Consequence Pathogens and Pathology - Poxvirus and Rabies Branch (DHCPP-PRB), Centers for Disease Control and Prevention (CDC)	Gigante,C., Xia.D., Zhao,H.. Batra.D., Hetrick,E.. Howard,D.. Kovar.L.. Seabolt,M., Morrison.S.. Desch,M , Knipe,K., Weigand,M , Cintron,R.. Burgin,A., Burroughs,M.. Lee.J., Wilkins.K.. McCollum.A., Hutson,C., Davidson,W., Rao.A., Plipat,N. and Li,Y.
EPI_ISL_l7394096, EPI_ISL_l7394097, EPI_ISL_l7394098	Division of High-Consequence Pathogens and Pathology - Poxvirus and Rabies Branch (DHCPP-PRB), Centers for Disease Control and Prevention (CDC)	Division of High-Consequence Pathogens and Pathology - Poxvirus and Rabies Branch (DHCPP-PRB), Centers for Disease Control and Prevention (CDC)	Gigante,C., Berns,A.. Zhao,H.. Batra.D., Hetrick,E., Howard,D., Kovar,L., Seabolt,M.. Morrison.S., Desch,M.. Knipe,K.. Weigand,M.. Cintron.R. Burgin,A.. Burroughs,M.. Lee.J., Wilkins.K., McCollum,A., Hutson,C., Davidson,W., Rao.A., Carpenter-Azevedo,K. and Li.Y.
EPI_ISL_l7394099, EPI_ISL_l 7394100	Division of High-Consequence Pathogens and Pathology - Poxvirus and Rabies Branch (DHCPP-PR8), Centers for Disease Contrai and Prevention (CDC)	Division of High-Consequence Pathogens and Pathology - Poxvirus and Rabies Branch (DHCPP-PRB), Centers for Disease Contrai and Prevention (CDC)	Gigante,C., Kubin,G., Zhao,H., Batra,D., Hetrick,E., Howard,□.,Kovar,L., Seabolt,M, Morrison,S., Desch,M., Knipe,K., Weigand,M. Cintron,R., Burgin,A.. Burroughs,M., Lee,J., Wilkins,K., McCollum,A., Hutson,C., Davidson,W., Rao.A., White,S. and Li,Y.
EPI_ISL_17394101, EPI_ISL_17394102, EPI_ISL_17394103, EPI_ISL_l 7394104, EPI_ISL_l 7394105	Division of High-Consequence Pathogens and Pathology - Poxvirus and Rabies Branch (DHCPP-PR8), Centers for Disease Contrai and Prevention (CDC)	Division of High-Consequence Pathogens and Pathology - Poxvirus and Rabies Branch (DHCPP-PRB), Centers for Disease Contrai and Prevention (CDC)	Gigante,C., Haydel,D., Zhao,H., Batra,D., Hetrick,E.. Howard,D., Kovar,L., Seabolt,M.. Morrison,S, Desch,M., Knipe,K., Weigand,M, Cintron,R., Burgin,A.. Burroughs,M.. Lee.J., Wilkins,K.. McCollum,A., Hutson,C., Davidson,W., Rao.A., Salinas.A. and Li.Y.
EPI_ISL_l7394106, EPI_ISL_l 7394107	Division of High-Consequence Pathogens and Pathology - Poxvirus and Rabies Branch (DHCPP-PRB), Centers for Disease Contrai and Prevention (CDC)	Division of High-Consequence Pathogens and Pathology - Poxvirus and Rabies Branch (DHCPP-PRB), Centers for Disease Contrai and Prevention (CDC)	Gigante,C. Ostadkar,R., Zhao,H., Batra,D., Hetrick,E., Howard,D.. Kovar,I., Seabolt,M., Morrison,S., Desch,M., Knipe,K., Weigand,M, Cintron,R.. Burgin,A., Burroughs,M., Lee,J., Wilkins,K., McCollum,A., Hutson,C., Davidson,W., Rao,A., Wang.X. and Li.Y.
EPI_ISL_l7406093, EPI_ISL_l7406094, EPJ_ISL_l 7406095, EPI_ISL_l7406096, EPI_ISL_l7406097, EPI_ISL_l7406098, EPI_ISL_l7406099, EPI_ISL_l7406100, EPI_ISL_l 7406101, EPI_ISL_l7406102, EPI_ISL_l7406103, EPI_ISL_l7406104, EPI_ISL_l 7406105, EPI_ISL_17406106, EPI_ISL_l7406107, EPI_ISL_l7406108, EPI_ISL_l7406109, EPI_ISL_l7406110, EPI_ISL_17406111, EPI_ISL_l7406112, EPI_ISL_l 7406113, EPI_ISL_l7406114, EPI_ISL_l7406115, EPI_ISL_l7406116, EPI_ISL_l7406117, EPI_ISL_l7406118, EPI_ISL_l7406119, EPI_ISL_l7406120, EPI_ISL_l7406121. EPI_ISL_l 7406122, EPI_ISL_l7406123, EPI_ISL_l7406124			
see above	CDCT/CEVS/SES-RS	CDCT/CEVS/SES-RS	Richard Steiner Salvato. Fernanda Marques Godinho, Regina Bones Barcellos, Patricia Sesterheim, Amanda Pellenz Ruivo, Viviane Horn de Melo. JUiio Augusto Schroder
EPI_ISL_l 7424657, EPI_ISL_l 7424658, EPJ_ISL_l 7424659, EPI_JSL_l 7424660, EPI_ISL_l 7424661, EPI_ISL_l 7424662, EPI_ISL_l 7424663, EPI_ISL_l 7424664, EPI_ISL_l 7424665, EPI_ISL_l 7424666, EPI_ISL_l 7424667, EPI_ISL_l 7424668, EPI_ISL_l 7424669, EPI_ISL_17424670, EPI_ISL_l 7424671, EPI_ISL_l 7424673, EPI_ISL_l 7424674, EPI_ISL_l 7424675, EPI_ISL_17424676, EPI_ISL_l 7424677, EPI_ISL_l 7424678			
see above	Molecular Microbiology Laboratory, Department of Pathology, Molecular and Cell-Based Medicine, Icahn School of Medicine at Mount Sinai,	Molecular Microbiology Laboratory, Department of Pathology, Molecular and Cell-Based Medicine, Icahn School of Medicine at Mount Sinai,	Luz H. Patiiio, Susana Guerra, Marina Muiioz, Nicolas Luna, Keith Farrugia, Adriana van de Guchte. Zain Khalil, Ana Silvia Gonzalez-Reiche, Matthew M. Hernandez ,Radhika Banu, Paras Shrestha, Bernadette Liggayu, Adolfo Firpo Betancourt, David Reich, Carlos Cordon-Cardo, Randy Albrechtf, Rebecca Pearlf, Viviana Simona, Aria Rookera, Emilia Mia Sordillo, Harm van Bakeld, Adolfo Garcfa-Sastre, Dusan Bogunovic, Gustavo Palacios, Alberto Paniz Mondolfi, Juan David Ramirez
EPI ISL 17428282, EPI ISL 17428283, EPI ISL 17428284, EPI IS(.17428286	(enter of Diagnostics and Vaccine Development. Centers for Disease Control	Center of Diagnostics and Vaccine Development. Centers for Disease Control	Lin,J.-H., Chiu,5.-C., Huang,H.-1., Huang,W.-l., Li,T.-Y., Fann,W.-B., Hsieh,P.-Y. and Yang,J.-Y.
EPI_ISL_l7428287	Centers for Disease Control and Prevention DHCPP-PRB, CDC	Centers for Disease Control and Prevention DHCPP-PRB, CDC	Gigante,C., Francis,□., Zhao,H., Batra,D.. Hetrick,E., Howard,□.,Kovar,L., Seabolt,M., Morrison,5.. Desch,M.. Knipe,K., Weigand,M.. Cintron,R.. Burgin,A.. Burroughs,M., Lee,J.. Wilkins,K., McCollum.A., Hutson,C.. Davidson,w.. Rao.A., Escobar,). and Li,Y.
EPI_ISL_l 7428289	Centers for Disease Control and Prevention DHCPP-PRB, CDC	Centers for Disease Control and Prevention DHCPP-PRB, CDC	Gigante,C. Vang,K., Zhao,H., Batra,D, Hetrick,E, Howard.D.. Kovar,I., Seabolt,M, Morrison,S., Desch.M., Knipe.K., Weigand,M., Cintron,R. Burgin,A., Burroughs,M., Lee,J., Wilkins,K., McCollum,A.. Hutson,C., Davidson,W., Rao.A., Seely,K. and li.Y.
EPI_ISL_l7428290, EPI_ISL_l7428291	Centers for Disease Control and Prevention DHCPP-PRB, CDC	Centers for Disease Control and Prevention DHCPP-PRB, CDC	Gigante,C.. Ghinai,I.. Zhao,H.. Batra,D., Hetrick,E, Howard,□,Kovar,L., Seabolt.M, Morrison,5, Desch,M., Knipe,K.. Weigand,M., Cintron,R., Burgin,A., Burroughs,M.. Lee,J., Wilkins,K, McCollum,A.. Hutson,C., Davidson,W.. Rao.A., Kerins,J. and Li,Y.

EPI_ISL_I7428292, EPI_ISL_I7428293	Centers for Disease Control and Prevention DHCPP-PRB, CDC	Centers for Disease Control and Prevention DHCPP-PRB, CDC	Gi9ante,C., Bradley.A. Zhao,H, Batra,D., Hetrick,E., Howard,D., Kovar,I. Seabolt.M., Morrison,S., Desch,M., Knipe,K., Weigand,M., Cintron,R, Burgin,A., Burroughs,M., Lee,J, Wilkins,K., McCollum,A., Hutson,C., Davidson,W., Rao.A., Anderson,J. and li,Y.
EPJ_ISL_I7428294, EPI_ISL_I 7428295	Centers for Disease Control and Prevention DHCPP-PRB, CDC	Centers for Disease Contrai and Prevention DHCPP-PRB, CDC	Gigante,C., Johnson,$., Zhao,H, Batra,D., Hetrick,E., Howard,D., Kovar,L., Seabolt,M, Morrison,S, Desch,M., Knipe,K., Weigand,M., Cintron,R., Burgin,A., Burroughs,M., Lee,J, Wilkins,K., McCollum,A., Hutson,C., Davidson,W., Rao.A., Riner.D. and li,Y.
EPI_ISL_I7428297	Centers for Disease Control and Prevention DHCPP-PRB, CDC	Centers for Disease Control and Prevention DHCPP-PRB, CDC	Gigante,C., Ostadkar,R., Zhao,H., Batra,D., Hetrick,E., Howard,D., Kovar,I., Seabolt,M., Morrison,S., Desch,M., Knipe,K., Weigand,M., Cintron,R., Burgin,A., Burroughs,M., Lee,J., Wilkins,K., McCollurn,A., Hutson,C., Davidson,W., Rao,A., Wang.X. and Li.Y.
EPI_ISL_I7428298, EPI_ISL_I 7428300, EPI_ISL_I7428302	Centers for Disease Control and Prevention DHCPP-PRB, CDC	Centers for Disease Control and Prevention DHCPP-PRB, CDC	Gigante,C., Cleavinger,K., Zhao,H., Batra,D., Hetrick,E.. Howard.D., Kovar,I., Seabolt,M., Morrison,S., Desch.M., Knipe.K., Weigand,M., Cintron,R., Burgin,A., Burroughs,M., Lee,J., Wilkins,K.. McCollurn,A., Hutson,C., Davidson,W.. Rao.A., Sinn,M. and li,Y.
EPI_ISL_I 7428303, EPI_ISL_I 7428305	Centers for Disease Control and Prevention DHCPP-PRB, CDC	Centers for Disease Control and Prevention DHCPP-PRB, CDC	Gigante,(., Fisher.S., Zhao,H., Batra,D., Hetrick,E., Howard,D., Kovar,l., Seabolt,M., Morrison.S., Desch,M., Knipe,K., Weigand,M., Cintron,R., Burgin,A., Burroughs,M., Lee,J., Wilkins,K., McCollum,A., Hutson,C., Davidson,W., Rao.A., Siebert,M. and li,Y.
EPI_ISL_I 7428306, EPI_ISL_I 7428307	Centers for Disease Control and Prevention DHCPP-PRB, CDC	Centers for Disease Control and Prevention DHCPP-PRB, CDC	Gigante,C., Ruiz.V., Zhao,H, Batra,D., Hetrick,E, Howard,D., Kovar,I., Seabolt,M., Morrison,S., Desch,M., Knipe,K., Weigand,M., Cintron,R., Burgin,A., Burroughs,M., Lee,J., Wilkins,K., McCollum,A., Hutson,C., Davidson,W., Rao.A., Wang,). and Li,Y.
EPI_ISL_I7428308, EPI_ISL_I7428309, EPI_ISL_I7428311	Centers for Disease Control and Prevention DHCPP-PRB, CDC	Centers for Disease Contrai and Prevention DHCPP-PRB, CDC	Gi9ante,C., Lee,B., Zhao,H., Batra,D., Hetrick,E, Howard,D., Kovar,I., Seabolt.M, Morrison,S., Desch,M., Knipe,K., Weigand,M., Cintron,R., Bur9in,A., Burroughs,M., Lee,J., Wilkins,K., McCollum,A., Hutson,C., Davidson,W., Rao.A., Salehi,E. and Li.Y.
EPI_ISL_I7428313, EPI_ISL_I7428315, EPI_ISL_I7428316	Centers for Disease Control and Prevention DHCPP-PRB, CDC	Centers for Disease Control and Prevention DHCPP-PRB, CDC	Gigante,C., Epie,N., Zhao,H., Batra,D., Hetrick,E., Howard,D., Kovar,I., Seabolt,M., Morrison,S., Desch,M., Knipe,K., Weigand,M., Cintron,R., Burgin,A., Burroughs,M., Lee,J., Wilkins,K., McCollum,A., Hutson,C., Davidson,W., Rao.A., Perez,T. and Li.Y.
EPI_ISL_I 7428317, EPI_ISL_I 7428319. EPI_ISL_I 7428321	Centers for Disease Control and Prevention DHCPP-PRB, CDC	Centers for Disease Control and Prevention DHCPP-PRB, CDC	Gigante,C., Thomas,L., Zhao.H., Batra,D.. Hetrick,E.. Howard.D., Kovar,l., Seabolt,M., Morrison,S., Desch.M., Knipe.K., Weigand.M., Cintron,R., Burgin.A., Burroughs,M., Lee,J.. Wilkins,K.. McCollum,A., Hutson,C.. Davidson,W.. Rao.A.. Dunn,J. and li,Y.
EPI_ISL_I7428323	Centers for Disease Control and Prevention DHCPP-PRB, CDC	Centers for Disease Control and Prevention DHCPP-PRB, CDC	Gigante,C., Kubin,G., Zhao,H., Batra,D., Hetrick,E., Howard,D., Kovar,L., Seabolt.M., Morrison,S., Desch,M., Knipe,K.. Weigand,M., Cintron,R., Burgin,A., Burroughs,M., lee,J., Wilkins,K., McCollum,A., Hutson,C., Davidson,W., Rao.A., White,S. and Li,Y.
EPI_ISL_I7428325, EPI_ISL_I 7428326, EPI_ISL_I7428328	Centers for Disease Control and Prevention DHCPP-PRB, CDC	Centers for Disease Control and Prevention DHCPP-PRB, CDC	Gigante,C., Segaloff,H.. Zhao,H., Batra,D.. Hetrick,E.. Howard,D.. Kovar,L., Seabolt.M.. Morrison.S., Desch,M., Knipe,K.. Weigand,M , Cintron,R., Burgin,A., Burroughs,M.. Lee,J.. Wilkins,K.. McCollum,A.. Hutson,C.. Davidson,W.. Rao.A.. Florek,K. and Li,Y.
EPI ISL 17445514, EPI ISL 17445515, EPI ISL 17445516, EPLISL I7445517, EPI ISL I7445518, EPI ISL I7445519	Tokyo Metropolitan Institute of Public Health	Tokyo Metropolitan Institute of Public Health	Fumi Kasuya, Wakaba Okada, Ryota Kuma9ai, Sachiko Harada, Arisa Amano, Michiya Hasegawa, Mami Nagashima, Kenji Sadamasu
EPI_ISL_I7471100, EPI_ISL_I7471101, EPJ_ISL_I 7471102, EPI_JSL_I 7471103, EPI_ISL_I7471104, EPI_ISL_I7471105, EPI_ISL_I7471106, EPI_ISL_I7471107, EPI_ISL_I7471108, EPI_ISL_I7471109, EPI_ISL_I7471110			
see above	Laboratorio de Enterovirus, Instituto Oswaldo Cruz, Fiocruz	Laboratory of Respiratory Viruses and Measles, Oswaldo Cruz Institute, FIOCRUZ	Paola Resende, Elisa Cavalcante Pereira, Bruna Mendonça da Silva, Jéssica Graça Macedo de Carvalho, Larissa Macedo Pinto, Victor Guimaraes, Marilda Siqueira, Renan da Silva Faustino, Marilia Santini, Beatriz Grinsztejn, Mayara Secco Torres da Silva, Edson Elias da Silva on behalf of the Fiocruz Genomic Surveillance Network
EPI_ISL_I7472037	Institut National de Recherche Biomedicale	Division of Infectious Disease Vaccine Research, Korea National Institute of Health	Lee,T.Y., Hwang,Y.-H., Yun,M.-R., Kim,Y.-J. and Kim,D.
EPI_ISL_I7472041, EPI_ISL_I7472042, EPI_ISL_I7472043, EPI ISL 17472044, EPI ISL 17472045, EPI ISL 17472046, EPI ISL I7472047	DPH, Massachusetts State Public Health laboratory	DPH, Massachusetts State Public Health Laboratory	Doucette,M., Gagne,L. and Smole,S.
EPI ISL 17485343	Laboratorio de Enterovirus, Instituto Oswaldo Cruz, Fiocruz	Instituto Oswaldo Cruz FIOCRUZ - Laboratory of Respiratory Viruses and Measles (LVRS)	Paola Resende, Elisa Cavalcante Pereira, Bruna Mendonça da Silva, Jéssica Graça Macedo de Carvalho, Larissa Macedo Pinto, Victor Guimaraes, Marilda Siqueira, Renan da Silva Faustino, Marilia Santini, Beatriz Grinsztejn, Mayara Secco Torres da Silva, Edson Elias da Silva on behalf of the Fiocruz Genomic Surveillance Network
EPI ISL 17485440, EPI ISL 17485441, EPJ ISL 17485442, EPI JSL 17485443, EPI ISL 17485444, EPI ISL 17485445, EPI ISL 17485446, EPI ISL 17485447, EPI ISL 17485448, EPI ISL 17485449, EPI ISL 17485450, EPI ISL 17485451, EPI ISL 17485452, EPI ISL 17485453, EPI ISL 17485454, EPI ISL 17485455, EPI ISL 17485456, EPI ISL 17485457, EPI ISL 17485458, EPI ISL 17485459, EPCISL=I7485460, EPI=ISL) 7485461, EPÜSL)7485462, EPCISL)7485463, EPI=ISL) 7485464, EPCISL=I7485465, EPI=ISL) 7485466, EPÜSL)7485467, EPCISL)7485468, EPI=ISL) 7485469, EPCISL=I7485470, EPI=ISL) 7485471, EPÜSL)7485472, EPCISL)7485473, EPI=ISL) 7485474, EPCISL=I 7485475, EPI=ISL) 7485476, EPÜSL)7485477, EPCISL)7485478, EPI=ISL) 7485479, EPI ISL 17485480, EPI ISL 17485481. EPI ISL 17485482, EPI ISL 17485483, EPI ISL 17485484, EPI ISL 17485485, EPI ISL 17485486, EPI ISL 17485487, EPI ISL 17485488, EPI ISL 17485489, EPI ISL 17485490, EPI ISL 17485491. EPI ISL 17485492, EPI ISL 17485493, EPI ISL 17485494, EPI ISL 17485495, EPI ISL 17485496, EPI ISL 17485497, EPI ISL 17485498, EPI ISL 17485499, EPI-ISL-17485500, EPI-ISL-17485501, EPI-ISL-17485502, EPI-ISL-17485503, EPI-ISL-17485504, EPI-ISL-17485505, EPI-ISL-17485506, EPI-ISL-17485507, EPI-ISL-17485508, EPI-ISL-17485509, EPI-ISL-17485510, EPI-ISL-17485511, EPI-ISL-17485512, EPI-ISL-17485513, EPI-ISL-17485514, EPI-ISL-17485515, EPI-ISL-17485516, EPI-ISL-17485517, EPI-ISL-17485518, EPI-ISL-17485519, EPI-ISL-17485520, EPI-ISL-17485521, EPI-ISL-17485522, EPI-ISL-17485523, EPI-ISL-17485524, EPI-ISL-17485525, EPI-ISL-17485526, EPI-ISL-17485527, EPI-ISL-17485528, EPI-ISL-17485529, EPI-ISL-17485530, EPI-ISL-17485531, EPI-ISL-17485532, EPI-ISL-17485533, EPI-ISL-17485534, EPI-ISL-17485535, EPI-ISL-17485536, EPI-ISL-17485537, EPI-ISL-17485538, EPI-ISL-17485539, EPI=ISL)7485540, EPI=ISL)7485541, EPÜSL)7485542			
seeabove	Centre for Biological Threats, Highly Pathogenic Viruses, Robert Koch Institute	Centre for Biological Threats, Highly Pathogenic Viruses, Robert Koch Institute	Brinkmann.A., Kohl,C., Schrick,l.. Michel,)., Schaade,l. and Nitsche,A.
EPI_ISL_I 7502583	Public Health Laboratory, Public Health Service Amsterdam, The Netherlands	Department of Medical Microbiolo9y & Infection prevention, Amsterdam University Medical Centers location AMC	Matthijs Welkers,)elle Koopsen, Robin van Houdt, Marcel Jonges, Sebastien Matamoros, Sjoerd Rebers, Fokla Zorgdrager, Sylvia Bruisten, Akke Cornelissen, Janke Schinkel, Ewout Fanoy, Roisin Bavalia, Menno de Jong and Mariken van der lubben on behalf of the Amsterdam Regional Genomic epidemiology and Outbreak Surveillance (ARGOS) consortium
EPI_ISL_I 7518107	Virology Section, Division of Microbiology,Osaka Institute of Public Health	Virology Section, Division of Microbiology,Osaka Institute of Public Health	Daiki Kanbayashi, Takako Kurata, Takuya Kawahata, Fumiya Bannno. Minami Hama, Kazushi Motomuta
EPI_ISL_I 7525484	Division de Microbiologia, Hospital Nacional de Nifios Carlos Saenz Herrera	Inciensa, Investigación y Enser'lanza en Nutrición y Salud Centra Nacional de Referencia de Virologfa	Cristian Perez Corrales, Christopher Mairena Acuna. Diana Cantillo, Hillary Serrano, Ana Isela Ruiz, Gustavo Vega, Claudio Soto-Garita, Adriana Godinez, Estela Cordero, Melany Calderon, Francisco Duarte
EPI_ISL_I7529368	laboratorio de Virus Exantematicos, Gastroentéricos y Otros Transmitidos por Vectores	Centro de Referencia Nacional de Genomica, Secuenciacion y Bioinformatica GENSBIO, INSPI-CZ9	Andrés Carrazco*, Slivia Salgado, Diana Gutiérrez, Damaris Alarc6n, Andrés Tinizaray, Ruth G6mez, Martha Sânchez, Johanna Parra les, Eva Nicola, Jorge Bejarano, Leandro Patifio.
EPI_ISL_I7536780	Department of Virology, National Institute of Health, Islamabad, Pakistan	Department of Virology. National Institute of Health. Islamabad, Pakistan	Massab Umair, Muhammad Ammar, Syed Adnan Haider. Rabia Hakim. Qasim Malik. Muhammad Salman, Ghazala Parveen. and Naseem Akhtar
EPI ISL 17536782, EPI ISL 17536783, EPI ISL 17536784, EPI ISL I7536785	laboratorio de Enterovirus, Instituto Oswaldo Cruz, Fiocruz	Instituto Oswaldo Cruz FIOCRUZ - Laboratory of Respiratory Viruses and Measles (LVRS)	Paola Resende, Elisa Cavalcante Pereira, Bruna Mendonça da Silva, Jéssica Graça Macedo de Carvalho, Larissa Macedo Pinto, Victor Guimaraes, Marilda Siqueira, Renan da Silva Faustino, Marilia Santini, Edson Elias da Silva on behalf of the Fiocruz Genomic Surveillance Network
EPI_ISL_I7582853	Ouest Diagnostics Nichols Institute	los Angeles County Public Health laboratories	P. Hemarajata et al.
EPI_ISL_I7584292	Centro Medico ABC	Instituto Nacional de Medicina Genomica	Cedro Tanda Alberto, Roxana Trejo Gonzillez, Laura Gomez-Romero, Alfredo Mendoza-Vargas, Dora Garnica-lopez, Alfredo Hidalgo-Miranda, luis A Herrera.
EPI_ISL_I 7592665, EPI_ISL_I 7592666, EPI_ISL_I7592667, EPI_ISL_I7592668, EPI_ISL_I7592669, EPI_ISL_I7592670	Tokyo Metropolitan Institute of Public Health	Tokyo Metropolitan Institute of Public Health	Fumi Kasuya. Wakaba Okada. Ryota Kumagai, Sachiko Harada, Arisa Amano. Michiya Hasegawa, Mami Nagashima, Kenji Sadamasu
EPJ_ISL_I 7595302, EPI_ISL_I7595303	Ouest Diagnostics Nichols Institute	los Angeles County Public Health laboratories	P. Hemarajata et al.
EPI_ISL_I7595304, EPI_ISL_I7595305, EPI_ISL_I7595306, EPJ_ISL_I7595307, EPI_JSL_I7595308	Kaiser Permanente China Hills Regional Reference Laboratories	Los Angeles County Public Health Laboratories	P. Hemarajata et al.
EPI_ISL_I 7614017. EPI_ISL_I7614018, EPJ_ISL_I 7614019. EPI_JSL_I 7614020. EPI_ISL_I7614021, EPI_ISL_I7614022. EPI_ISL_I7614023, EPI_ISL_I7614024. EPI_ISL_I7614025. EPI_ISL_I7614026, EPI_ISL_I7614027. EPI_ISL_I7614028, EPI_ISL_I7614029. EPI_ISL_17614030. EPI_ISL_I7614031, EPI_ISL_I7614032. EPI_ISL_I7614033, EPI_ISL_I7614034. EPI_ISL_17614035, EPI_ISL_I7614036, EPI_ISL_I7614037, EPI_ISL_I7614038, EPI_ISL_I7614039, EPI_ISL_I7614040, EPI_ISL_I7614041, EPI_ISL_I7614042, EPI_ISL_I7614043, EPI_ISL_I7614044, EPI_ISL_I7614045, EPI_ISL_I7614046, EPI_ISL_I7614047, EPI_ISL_I7614048, EPI_ISL_I 7614049			
see above	Laboratorio de Enterovirus, Instituto Oswaldo Cruz, Fiocruz	Instituto Oswaldo Cruz FIOCRUZ - Laboratory of Respiratory Viruses and Measles (LVRS)	Paola Resende, Elisa Cavalcante Pereira, Bruna Mendonça da Silva, Jéssica Graça Macedo de Carvalho, Larissa Macedo Pinto, Victor Guimaraes, Marilda Siqueira, Renan da Silva Faustino, Marilia Santini, Edson Elias da Silva on behalf of the Fiocruz Genornic Surveillance Network
EPI ISL 17665624, EPI ISL 17665625, EPI ISL 17665626, EPI ISL I7665627	Tokyo Metropolitan Institute of Public Health	Tokyo Metropolitan Institute of Public Health	Fumi Kasuya, Wakaba Okada, Ryota Kumagai, Sachiko Harada, Arisa Amano, Michiya Hasegawa, Mami Nagashima, Kenji Sadamasu
EPI_ISL_I 7672206	LESP State of Mexico	Instituto de Diagnostico y Referencia Epidemiologicos (INDRE)	Abril Rodriguez-Maldonado; Claudia Wong-Arâmbula; Silvia Rivero-Arredondo; Ruth Madera-Sandoval; Joaqufn Quiroz-Mercado; Fernando Gonzâlez-Domfnguez; Lucia Hernândez-Rivas, Irma L6pez-Martinez; Ernesto Ramirez-Gonzâlez: Maribel Gonzâlez-Villa
EPI_ISL_I 7672207	LESP Jalisco	Instituto de Diagnostico y Referencia Epidemiologicos (INDRE)	Abril Rodrfguez-Maldonado: Claudia Wong-Arâmbula; Silvia Rivero-Arredondo; Ruth Madera-Sandoval; Joaqufn Quiroz-Mercado; Fernando Gonzâlez-Domînguez; Lucia Hernândez-Rivas, Irma Lôpez-Martfnez: Ernesto Ramfrez-Gonzâlez; Maribel Gonzâlez-Villa
EPI_ISL_I7672208	LESP Queretaro	Instituto de Diagnostico y Referencia Epidemiologicos (INDRE)	Abril Rodrfguez-Maldonado; Claudia Wong-Arâmbula; Silvia Rivero-Arredondo; Ruth Madera-Sandoval; Joaqufn Quiroz-Mercado; Fernando Gonzâlez-Domfnguez; Lucia Hernândez-Rivas, Irma L6pez-Martfnez; Ernesto Ramfrez-Gonzâlez; Maribel Gonzâlez-Villa
EPI_ISL_I 7672209	LESP Yucatan	Instituto de Diagnostico y Referencia Epidemiologicos (INDRE)	Abril Rodrfguez-Maldonado; Claudia Wong-Arâmbula; Silvia Rivero-Arredondo; Ruth Madera-Sandoval; Joaqufn Quiroz-Mercado; Fernando Gonzâlez-Domfnguez; Lucia Hernândez-Rivas, Irma L6pez-Mart1nez; Ernesto Ramlrez-Gonzâlez; Maribel Gonzâlez-Villa
EPI_ISL_I7672210	LESP Quintana Roo	Instituto de Diagnostico y Referencia Epiderniologicos (INDRE)	Abril Rodriguez-Maldonado; Claudia Wong-Arâmbula; Silvia Rivero-Arredondo; Ruth Madera-Sandoval; Joaqufn Quiroz-Mercado; Fernando Gonzâlez-Domfnguez; Lucia Hernândez-Rivas, Irma L6pez-Mart/nez; Ernesto Ram/rez-Gonziilez: Maribel Gonzâlez-Villa
EPI_ISL_I 7672211	LESP Mexico City	Instituto de Diagnostico y Referencia Epidemiologicos (INDRE)	Abril Rodrfguez-Maldonado; Claudia Wong-Arâmbula; Silvia Rivero-Arredondo; Ruth Madera-Sandoval; Joaqufn Quiroz-Mercado; Fernando Gonzâlez-Domfnguez: Lucia Hernândez-Rivas, Irma Lôpez-Martfnez; Ernesto Ramfrez-Gonzâlez; Maribel Gonzâlez-Villa
EPI_ISL_I 7672212	LESP Tamaulipas	Instituto de Diagnostico y Referencia Epidemiologicos (INDRE)	Abril Rodrfguez-Maldonado: Claudia Wong-Arâmbula; Silvia Rivero-Arredondo; Ruth Madera-Sandoval; Joaqufn Quiroz-Mercado; Fernando Gonzâlez-Domfnguez; Lucia Hernândez-Rivas. Irma Lôpez-Martfnez; Ernesto Ramfrez-Gonzâlez; Maribel Gonzâlez-Villa
EPI_ISL_I 7672213	LESP Puebla	Instituto de Diagnostico y Referencia Epidemiologicos (INDRE)	Abril Rodrfguez-Maldonado; Claudia Wong-Arâmbula; Silvia Rivero-Arredondo; Ruth Madera-Sandoval; Joaqufn Ouiroz-Mercado; Fernando Gonzâlez-Domfnguez; Lucia Hernândez-Rivas, Irma L6pez-Martfnez; Ernesto Ramfrez-Gonzâlez; Maribel Gonzâlez-Villa
EPI_ISL_I 7672214	LESP Guerrero	Instituto de Diagnostico y Referencia Epidemiologicos (INDRE)	Abril Rodrfguez-Maldonado; Claudia Wong-Arâmbula; Silvia Rivero-Arredondo; Ruth Madera-Sandoval; Joaqufn Quiroz-Mercado; Fernando Gonzâlez-Domfnguez; Lucia Hernândez-Rivas, Irma L6pez-Martfnez; Ernesto Ramfrez-Gonzâlez; Maribel Gonzâlez-Villa
EPI_ISL_I7672215	LESP Oaxaca	Instituto de Diagnostico y Referencia Epiderniologicos (INDRE)	Abril Rodriguez-Maldonado; Claudia Wong-Arâmbula; Silvia Rivero-Arredondo; Ruth Madera-Sandoval; Joaqufn Quiroz-Mercado; Fernando Gonzâlez-Domfnguez; Lucia Hernândez-Rivas, Irma L6pez-Martinez: Ernesto Ramirez-Gonzâlez: Maribel Gonzâlez-Villa
EPI_ISL_I 7672216	LESP Hidalgo	Instituto de Diagnostico y Referencia Epidemiologicos (INDRE)	Abril Rodrfguez-Maldonado; Claudia Wong-Arâmbula; Silvia Rivero-Arredondo; Ruth Madera-Sandoval; Joaqufn Quiroz-Mercado; Fernando Gonzâlez-Domfnguez; Lucia Hernândez-Rivas, Irma L6pez-Martfnez; Ernesto Ramfrez-Gonzâlez: Maribel Gonzâlez-Villa

EPI_ISL_I 7672217	LESP Zacatecas	Instituto de Diagnostico y Referencia Epidemiologicos (INDRE)	Abril Rodrfguez-Maldonado; Claudia Wong-Arémbula; Silvia Rivero-Arredondo; Ruth Madera-Sandoval; Joaqufn Quir□z•Mercado; Fernando Gonzélez-Domfnguez: Lucia Hernéndez•Rivas, Irma L6pez-Martfnez; Ernesto Ramfrez-González: Maribel González-Villa
EPI_ISL_I7672218	LESP Tabasco	Institut□ de Diagnostic□y Referencia Epidemiologicos (INDRE)	Abril Rodriguez-Maldonado; Claudia Wong-Arâmbula; Silvia Rivero-Arredondo; Ruth Madera-Sandoval; Joaqufn Quiroz-Mercado; Fernando González-Domfnguez: Lucia Hernândez-Rivas, Irma L6pez-Martinez: Ernesto Ramirez-González: Maribel González-Villa
EPI_ISL_I7672219	LESP Aguascalientes	Institut□ de Diagnostic□y Referencia Epidemiologicos (INDRE)	Abril Rodrfguez-Maldonado: Claudia Wong-Arâmbula; Silvia Rivero-Arredondo; Ruth Madera-Sandoval; Joaqufn Quiroz-Mercado; Fernando González-Domfnguez: Lucia Hernândez-Rivas, Irma L6pez-Martfnez; Ernesto Ramfrez-Gonzàlez: Maribel Gonzàlez-Villa
EPI_ISL_I7672220	LESP Tlaxcala	Institut□ de Diagnostic□y Referencia Epidemiologicos (INDRE)	Abril Rodrfguez-Maldonado; Claudia Wong-Arâmbula; Silvia Rivero-Arredondo; Ruth Madera-Sandoval; Joaqufn Quiroz-Mercado; Fernando González-Domfnguez: Lucia Hernândez-Rivas, Irma L6pez-Martfnez: Ernesto Ramfrez-González: Maribel González-Villa
EPI_ISL_I 7672221	LESP Chihuahua	Institut□ de Diagnostic□y Referencia Epidemiologicos (INDRE)	Abril Rodrfguez-Maldonado; Claudia Wong-Arâmbula; Silvia Rivero-Arredondo; Ruth Madera-Sandoval; Joaqufn Quiroz-Mercado; Fernando González-Domfnguez: Lucia Hernàndez-Rivas, Irma Lôpez-Martfnez: Ernesto Ramfrez-Gonzélez: Maribel Gonzélez-Villa
EPI_ISL_I7672222	LESP Veracruz	Instituto de Diagnostico y Referencia Epidemiologicos (INDRE)	Abril Rodrfguez-Maldonado: Claudia Wong-Arémbula; Silvia Rivero-Arredondo; Ruth Madera-Sandoval; Joaqufn Quiroz-Mercado; Fernando Gonzélez-Domfnguez: Lucia Hernàndez-Rivas, Irma L6pez-Martfnez: Ernesto Ramfrez-González: Maribel González-Villa
EPI_ISL_I7672223	LESP Baja California	Institut□ de Diagnostic□y Referencia Epidemiologicos (INDRE)	Abril Rodrfguez-Maldonado; Claudia Wong-Arâmbula; Silvia Rivero-Arredondo; Ruth Madera-Sandoval; Joaqufn Quiroz-Mercado; Fernando González-Domfnguez: Lucia Hernândez-Rivas, Irma L6pez-Martfnez; Ernesto Ramfrez-González: Maribel González-Villa
EPI_ISL_I 7672224	LESP Tamaulipas	Institut□ de Diagnostic□y Referencia Epidemiologicos (INDRE)	Abril Rodriguez-Maldonado; Claudia Wong-Arâmbula; Silvia Rivero-Arredondo; Ruth Madera-Sandoval; Joaqufn Quiroz-Mercado; Fernando González-Domfnguez; Lucia Hernândez-Rivas, Irma L6pez-Martinez; Ernesto Ramirez-González: Maribel González-Villa
EPI_ISL_I7672225	LESP Campeche	Institut□ de Diagnostic□y Referencia Epidemiologicos (INDRE)	Abril Rodrfguez-Maldonado: Claudia Wong-Arâmbula; Silvia Rivero-Arredondo; Ruth Madera-Sandoval; Joaqufn Quiroz-Mercado; Fernando González-Domfnguez; Lucia Hernândez-Rivas, Irma L6pez-Martfnez: Ernesto Ramfrez-Gonzàlez; Maribel Gonzàlez-Villa
EPI_ISL_I7672226	LESP Hidalgo	Institut□ de Diagnostic□y Referencia Epidemiologicos (INDRE)	Abril Rodrfguez-Maldonado; Claudia Wong-Arâmbula; Silvia Rivero-Arredondo; Ruth Madera-Sandoval; Joaqufn Quiroz-Mercado; Fernando González-Domfnguez; Lucia Hernândez-Rivas, Irma L6pez-Martfnez: Ernesto Ramfrez-Gonzàlez; Maribel Gonzàlez-Villa
EPI_ISL_I 7672227	LESP Mexico City	Institut□ de Diagnostic□y Referencia Epidemiologicos (INDRE)	Abril Rodrfguez-Maldonado: Claudia Wong-Arâmbula; Silvia Rivero-Arredondo; Ruth Madera-Sandoval; Joaqufn Quiroz-Mercado; Fernando González-Domfnguez: Lucia Hernândez-Rivas, Irma Lôpez-Martfnez; Ernesto Ramfrez-González; Maribel González-Villa
EPI_ISL_I7672228	LESP Guerrero	Instituto de Diagnostico y Referencia Epidemiologicos (INDRE)	Abril Rodrfguez-Maldonado: Claudia Wong-Arémbula; Silvia Rivero-Arredondo; Ruth Madera-Sandoval; Joaqufn Quiroz-Mercado; Fernando Gonzélez-Domfnguez: Lucia Hernéndez-Rivas, Irma L6pez-Martinez; Ernesto Ramirez-González; Maribel González-Villa
EPI_ISL_I7672229	LESP Coahuila	Institut□ de Diagnostic□y Referencia Epidemiologicos (INDRE)	Abril Rodrfguez-Maldonado; Claudia Wong-Arâmbula; Silvia Rivero-Arredondo; Ruth Madera-Sandoval; Joaqufn Quiroz-Mercado; Fernando González-Domfnguez: Lucia Hernândez-Rivas, Irma L6pez-Martinez; Ernesto Ramirez-Gonzâlez: Maribel González-Villa
EPI_ISL_I7672230	LESP Tabasco	Institut□ de Diagnostic□y Referencia Epidemiologicos (INDRE)	Abril Rodrfguez-Maldonado; Claudia Wong-Arâmbula; Silvia Rivero-Arredondo; Ruth Madera-Sandoval; Joaqufn Quiroz-Mercado; Fernando Gonzâlez-Domfnguez; Lucia Hernández-Rivas, Irma L6pez-Martfnez; Ernesto Ramfrez-González: Maribel González-Villa
EPI_ISL_I 7672231	LESP Chiapas	Institut□ de Diagnostic□y Referencia Epidemiologicos (INDRE)	Abril Rodrfguez-Maldonado; Claudia Wong-Arâmbula; Silvia Rivero-Arredondo; Ruth Madera-Sandoval; Joaqufn Quiroz-Mercado; Fernando González-Domfnguez; Lucia Hernândez-Rivas, Irma L6pez-Martfnez: Ernesto Ramfrez-González; Maribel González-Villa
EPI_ISL_I7672232	LESP Yucatan	Institut□ de Diagnostic□y Referencia Epidemiologicos (INDRE)	Abril Rodrfguez-Maldonado; Claudia Wong-Arâmbula; Silvia Rivero-Arredondo; Ruth Madera-Sandoval; Joaqufn Quiroz-Mercado; Fernando González-Domfnguez: Lucia Hernândez-Rivas, Irma Lôpez-Martfnez; Ernesto Ramfrez-Gonzélez; Maribel Gonzélez-Villa
EPI_ISL_I 7672233	LESP Quintana Roo	Instituto de Diagnostico y Referencia Epidemiologicos (INDRE)	Abril Rodrfguez-Maldonado: Claudia Wong-Arâmbula; Silvia Rivero-Arredondo; Ruth Madera-Sandoval; Joaqufn Quiroz-Mercado; Fernando Gonztilez-Domfnguez: Lucia Herntindez-Rivas, Irma L6pez-Martfnez; Ernesto Ramfrez-González; Maribel González-Villa
EPI_ISL_I7672234	LESP Jalisco	Institut□ de Diagnostic□y Referencia Epidemiologicos (INDRE)	Abril Rodrfguez-Maldonado; Claudia Wong-Arâmbula; Silvia Rivero-Arredondo; Ruth Madera-Sandoval; Joaqufn Quiroz-Mercado; Fernando González-Domfnguez; Lucia Hernândez-Rivas, Irma L6pez-Martfnez; Ernesto Ramfrez-González; Maribel González-Villa
EPI_ISL_I 7672235	LESP State of Mexico	Institut□ de Diagnostic□y Referencia Epidemiologicos (INDRE)	Abril Rodrfguez-Maldonado; Claudia Wong-Arâmbula; Silvia Rivero-Arredondo; Ruth Madera-Sandoval; Joaqufn Quiroz-Mercado; Fernando González-Domfnguez: Lucia Hernândez-Rivas, Irma L6pez-Martfnez; Ernesto Ramfrez-González: Maribel González-Villa
EPI_ISL_I7672236	Laboratorio Estatal de Salud PIJblica Puebla	Institut□ de Diagnostic□y Referencia Epidemiologicos (INDRE)	Abril Rodrfguez-Maldonado; Claudia Wong-Arâmbula; Silvia Rivero-Arredondo; Ruth Madera-Sandoval; Joaqufn Quiroz-Mercado; Fernando Gonztilez-Domfnguez; Lucia Herntindez-Rivas, Irma L6pez-Martfnez; Ernesto Ramfrez-González; Maribel González-Villa
EPI_ISL_I7672237	LESP Campeche	Institut□ de Diagnostic□y Referencia Epidemiologicos (INDRE)	Abril Rodrfguez-Maldonado; Claudia Wong-Artlmbula; Silvia Rivero-Arredondo; Ruth Madera-Sandoval; Joaqufn Quiroz-Mercado; Fernando Gonztilez-Domfnguez; Lucia Hernândez-Rivas, Irma L6pez-Martfnez: Ernesto Ramfrez-González; Maribel González-Villa
EPI_ISL_I7672238	LESP State of Mexico	Instituto de Diagnostic□y Referencia Epidemiologicos (INDRE)	Abril Rodrfguez-Maldonado: Claudia Wong-Artimbula; Silvia Rivero-Arredondo; Ruth Madera-Sandoval; Joaqufn Quiroz-Mercado; Fernando Gonztilez-Domfnguez; Lucia Hernândez-Rivas, Irma L6pez-Martfnez; Ernesto Ramfrez-González; Maribel González-Villa
EPI_ISL_I7672239	LESP Chiapas	Instituto de Diagnostico y Referencia Epidemiologicos (INDRE)	Abril Rodriguez-Maldonado; Claudia Wong-Arâmbula; Silvia Rivero-Arredondo; Ruth Madera-Sandoval; Joaqufn Quiroz-Mercado; Fernando Gonzâlez-Domfnguez; Lucia Hernândez-Rivas, Irma L6pez-Martinez; Ernesto Ramirez-González; Maribel González-Villa
EPI_ISL_I7672240	LESP Coahuila	Institut□ de Diagnostic□y Referencia Epidemiologicos (INDRE)	Abril Rodrfguez-Maldonado; Claudia Wong-Arâmbula; Silvia Rivero-Arredondo; Ruth Madera-Sandoval; Joaqufn Quiroz-Mercado; Fernando González-Domfnguez; Lucia Hernândez-Rivas, Irma L6pez-Martfnez; Ernesto Ramfrez-González: Maribel González-Villa
EPI_ISL_I7672241	LESP Jalisco	Institut□ de Diagnostic□y Referencia Epidemiologicos (INDRE)	Abril Rodrfguez-Maldonado; Claudia Wong-Arâmbula; Silvia Rivero-Arredondo; Ruth Madera-Sandoval; Joaqufn Quiroz-Mercado; Fernando González-Domfnguez; Lucia Hernândez-Rivas, Irma L6pez-Martfnez; Ernesto Ramfrez-Gonzàlez: Maribel Gonzàlez-Villa
EPI_ISL_I 7672242	LESP Nuevo Lean	Institut□ de Diagnostic□y Referencia Epidemiologicos (INDRE)	Abril Rodrfguez-Maldonado; Claudia Wong-Arâmbula; Silvia Rivero-Arredondo; Ruth Madera-Sandoval; Joaqufn Quiroz-Mercado; Fernando González-Domfnguez; Lucia Hernândez-Rivas, Irma L6pez-Martfnez; Ernesto Ramfrez-González: Maribel González-Villa
EPI_ISL_I7672243	LESP Queretaro	Institut□ de Diagnostic□y Referencia Epidemiologicos (INDRE)	Abril Rodrfguez-Maldonado; Claudia Wong-Arâmbula; Silvia Rivero-Arredondo; Ruth Madera-Sandoval; Joaqufn Quiroz-Mercado; Fernando González-Domfnguez; Lucia Hernândez-Rivas, Irma L6pez-Martfnez; Ernesto Ramfrez-González; Maribel González-Villa
EPI_ISL_I 7672244	LESP Hidalgo	Instituto de Diagnostico y Referencia Epidemiologicos (INDRE)	Abril Rodrfguez-Maldonado: Claudia Wong-Arâmbula; Silvia Rivero-Arredondo; Ruth Madera-Sandoval; Joaqufn Quiroz-Mercado; Fernando González-Domfnguez; Lucia Hernândez-Rivas, Irma L6pez-Martfnez; Ernesto Ramfrez-González; Maribel González-Villa
EPI_ISL_I767224S	LESP Tamaulipas	Institut□ de Diagnostic□y Referencia Epidemiologicos (INDRE)	Abril Rodrfguez-Maldonado; Claudia Wong-Arâmbula; Silvia Rivero-Arredondo; Ruth Madera-Sandoval; Joaqufn Quiroz-Mercado; Fernando González-Domfnguez; Lucia Hernândez-Rivas, Irma L6pez-Martfnez; Ernesto Ramirez-Gonzâlez: Maribel Gonzâlez-Villa
EPI_ISL_I 7672246	LESP Veracruz	Institut□ de Diagnostic□y Referencia Epidemiologicos (INDRE)	Abril Rodrfguez-Maldonado; Claudia Wong-Arâmbula; Silvia Rivero-Arredondo; Ruth Madera-Sandoval; Joaqufn Quiroz-Mercado; Fernando González-Domfnguez; Lucia Hernândez-Rivas, Irma L6pez-Martfnez; Ernesto Ramfrez-Gonzàlez: Maribel Gonzàlez-Villa
EPI_ISL_I7672247	LESP Quintana Rao	Institut□ de Diagnostic□y Referencia Epidemiologicos (INDRE)	Abril Rodrfguez-Maldonado: Claudia Wong-Arâmbula; Silvia Rivero-Arredondo; Ruth Madera-Sandoval; Joaqufn Quiroz-Mercado; Fernando Gonzâlez-Domfnguez; Lucia Hernández-Rivas, Irma L6pez-Martfnez; Ernesto Ramfrez-González; Maribel Gonzàlez-Villa
EPI_ISL_I7672248	LESP Queretaro	Institut□ de Diagnostic□y Referencia Epidemiologicos (INDRE)	Abril Rodrfguez-Maldonado; Claudia Wong-Arémbula; Silvia Rivero-Arredondo; Ruth Madera-Sandoval; Joaqufn Quiroz-Mercado; Fernando González-Domfnguez; Lucia Hernândez-Rivas, Irma L6pez-Martfnez; Ernesto Ramfrez-Gonzàlez; Maribel Gonzàlez-Villa
EPI_ISL_I7672249	LESP Oaxaca	Instituto de Diagnostic□y Referencia Epidemiologicos (INDRE)	Abril Rodrfguez-Maldonado: Claudia Wong-Artimbula; Silvia Rivero-Arredondo; Ruth Madera-Sandoval; Joaqufn Quiroz-Mercado; Fernando Gonztilez-Domfnguez; Lucia Hernândez-Rivas, Irma L6pez-Martfnez; Ernesto Ramfrez-González: Maribel González-Villa
EPI_ISL_I 7672250	LESP Yucatan	Instituto de Diagnostico y Referencia Epidemiologicos (INDRE)	Abril Rodriguez-Maldonado; Claudia Wong-Arâmbula; Silvia Rivero-Arredondo; Ruth Madera-Sandoval; Joaqufn Quiroz-Mercado; Fernando Gonzâlez-Domfnguez; Lucia Hernândez-Rivas, Irma L6pez-Martinez; Ernesto Ramirez-González; Maribel González-Villa
EPI_ISL_I7672251	LESP Guanajuato	Institut□ de Diagnostic□y Referencia Epidemiologicos (INDRE)	Abril Rodr/guez-Maldonado; Claudia Wong-Arâmbula; Silvia Rivero-Arredondo; Ruth Madera-Sandoval; Joaqufn Quiroz-Mercado; Fernando González-Domfnguez; Lucia Hernândez-Rivas, Irma L6pez-Martfnez; Ernesto Ramfrez-González: Maribel González-Villa
EPI_ISL_I7672252	LESP Veracruz	Institut□ de Diagnostic□y Referencia Epidemiologicos (INDRE)	Abril Rodrfguez-Maldonado; Claudia Wong-Arâmbula; Silvia Rivero-Arredondo; Ruth Madera-Sandoval; Joaqufn Quiroz-Mercado; Fernando González-Domfnguez; Lucia Hernândez-Rivas, Irma L6pez-Martfnez; Ernesto Ramfrez-Gonzàlez; Maribel Gonzàlez-Villa
EPI_ISL_I7672253	LESP Mexico City	Institut□ de Diagnostic□y Referencia Epidemiologicos (INDRE)	Abril Rodr/guez-Maldonado; Claudia Wong-Arâmbula; Silvia Rivero-Arredondo; Ruth Madera-Sandoval; Joaqufn Quiroz-Mercado; Fernando González-Domfnguez; Lucia Hernândez-Rivas, Irma L6pez-Martfnez; Ernesto Ramfrez-González: Maribel González-Villa
EPI_ISL_I 7672254	LESP Durango	Institut□ de Diagnostic□y Referencia Epidemiologicos (INDRE)	Abril Rodrfguez-Maldonado; Claudia Wong-Arâmbula; Silvia Rivero-Arredondo; Ruth Madera-Sandoval; Joaqufn Quiroz-Mercado; Fernando González-Domfnguez; Lucia Hernândez-Rivas, Irma L6pez-Martfnez; Ernesto Ramfrez-González: Maribel González-Villa
EPI_ISL_I7672255	LESP San Luis Potosi	Institut□ de Diagnostic□y Referencia Epidemiologicos (INDRE)	Abril Rodrfguez-Maldonado: Claudia Wong-Arâmbula; Silvia Rivero-Arredondo; Ruth Madera-Sandoval; Joaqufn Quiroz-Mercado; Fernando González-Domînguez; Lucia Hernândez-Rivas, Irma Lôpez-Martfnez; Ernesto Ramfrez-González; Maribel González-Villa
EPI_ISL_I7672256	LESP Nuevo Lean	Institut□ de Diagnostic□y Referencia Epidemiologicos (INDRE)	Abril Rodrfguez-Maldonado: Claudia Wong-Arémbula; Silvia Rivero-Arredondo; Ruth Madera-Sandoval; Joaqufn Quiroz-Mercado; Fernando González-Domfnguez; Lucia Hernândez-Rivas, Irma L6pez-Martfnez; Ernesto Ramfrez-González; Maribel González-Villa
EPI_ISL_I7672257, EPI_ISL_I7672258	LESP Quintana Rao	Instituto de Diagnostic□y Referencia Epidemiologicos (INDRE)	Abril Rodrfguez-Maldonado: Claudia Wong-Artimbula; Silvia Rivero-Arredondo; Ruth Madera-Sandoval; Joaqufn Quiroz-Mercado; Fernando Gonztilez-Domfnguez: Lucia Hernândez-Rivas, Irma L6pez-Martfnez; Ernesto Ramfrez-González; Maribel González-Villa
EPI_ISL_I 76722S9	LESP Mexico City	Instituto de Diagnostic□y Referencia Epidemiologicos (INDRE)	Abril Rodriguez-Maldonado; Claudia Wong-Arâmbula; Silvia Rivero-Arredondo; Ruth Madera-Sandoval; Joaqufn Quiroz-Mercado; Fernando González-Domfnguez: Lucia Hernândez-Rivas, Irma L6pez-Martinez; Ernesto Ramirez-González; Maribel González-Villa
EPI_ISL_I7672260	LESP Veracruz	Institut□ de Diagnostic□y Referencia Epidemiologicos (INDRE)	Abril Rodrfguez-Maldonado; Claudia Wong-Arâmbula; Silvia Rivero-Arredondo; Ruth Madera-Sandoval; Joaqufn Quiroz-Mercado; Fernando González-Domfnguez; Lucia Hernândez-Rivas, Irma L6pez-Martfnez; Ernesto Ramfrez-González: Maribel González-Villa
EPI_ISL_I7692268, EPI_ISL_I7692269	Tokyo Metropolitan Institute of Public Health	Tokyo Metropolitan Institute of Public Health	Fumi Kasuya, Wakaba Okada, Ryota Kumagai, Sachiko Harada, Arisa Amano, Michiya Hasegawa, Mami Nagashima, Kenji Sadamasu
EPI_ISL_I7703564, EPI_ISL_I7703565	LESP State of Mexico	Institut□ de Diagnostic□y Referencia Epidemiologicos (INDRE)	Abril Rodr/guez-Maldonado; Claudia Wong-Arâmbula; Felipe Arguijo-Perez; Helios Cérdenas-Hernéndez; Carmen Castro-Méndez; Lidia Garda-Torres; Ruth Madera-Sandoval; América Mandujano-Mart/nez; Nancy Martfnez-Velézquez; Mireya Mederos-Michel; Angélica Pedraza-Meléndez; Joaquîn Quiroz-Mercado; Daniel Regalado-Santiago; Silvia Rivero-Arredondo; Erika Sierra-Atanacio; Fernando Gonzélez-Domfnguez; Lucia Hernández-Rivas, Irma L6pez-Martfnez:

			Ernesto Ramfrez-Gonzâlez; Maribel Gonzâlez-Villa
EPI ISL 17703566	LESP Jalisco	Instituto de Diagnostico y Referencia Epidemiologicos (INDRE)	Abril Rodrfguez-Maldonado; Claudia Wong-Arâmbula; Felipe Arguijo-Perez; Helios Câcdenas-Hernândez; Carmen Castro-Méndez; Lidia Garda-Torres; Ruth Madera-Sandoval; América Mandujano-Martfnez; Nancy Martfnez-Velâzquez; Mireya Mederos-Michel; Angélica Pedraza-Meléndez; Joaqufn Quiroz-Mercado; Daniel Regalado-Santiago; Silvia Rivero-Arredondo; Erika Sierra-Atanacio; Fernando Gonzâlez-Domfnguez; Lucia Hernândez-Rivas, Irma L6pez-Martfnez; Ernesto Ramfrez-Gonzâlez; Maribel Gonzâlez-Villa
EPI_ISL_I7703567	LESP State of Mexico	Instituto de Diagnostico y Referencia Epidemiologicos (INDRE)	Abril Rodriguez-Maldonado; Claudia Wong-Arâmbula; Felipe Arguijo-Perez; Helios Cârdenas-Hernândez; Carmen Castro-Méndez; Lidia Garda-Torres; Ruth Madera-Sandoval; América Mandujano-Martinez; Nancy Martfnez-Velàzquez; Mireya Mederos-Michel; Angélica Pedraza-Meléndez; Joaqufn Quiroz-Mercado; Daniel Regalado-Santiago; Silvia Rivero-Arredondo; Erika Sierra-Atanacio; Fernando Gonzâlez-Domfnguez; Lucia Hernândez-Rivas, Irma L6pez-Martfnez; Ernesto Ramfrez-Gonzâlez; Maribel Gonzâlez-Villa
EPI ISL 17703568	LESP Quintana Roo	Instituto de Diagnostico y Referencia Epidemiologicos (INDRE)	Abril Rodrfguez-Maldonado; Claudia Wong-Arâmbula; Felipe Arguijo-Perez; Helios Cârdenas-Hernândez; Carmen Castro-Méndez; Lidia Garda-Torres; Ruth Madera-Sandoval; América Mandujano-Martfnez; Nancy Martfnez-Velâzquez; Mireya Mederos-Michel; Angélica Pedraza-Meléndez; Joaqufn Quiroz-Mercado; Daniel Regalado-Santiago; Silvia Rivero-Arredondo; Erika Sierra-Atanacio; Fernando Gonzâlez-Domfnguez; Lucia Hernândez-Rivas, Irma L6pez-Martinez; Ernesto Ramfrez-Gonzâlez; Maribel Gonzâlez-Villa
EPI ISL 17703569, EPI ISL 17703570	LESP Mexico City	Instituto de Diagnostico y Referencia Epidemiologicos (INDRE)	Abril Rodr/guez-Maldonado; Claudia Wong-Arâmbula; Felipe Arguijo-Perez; Helios Cârdenas-Hernândez; Carmen Castro-Méndez; Lidia Garda-Torres; Ruth Madera-Sandoval; América Mandujano-Martfnez; Nancy Martfnez-Velâzquez; Mireya Mederos-Michel; Angélica Pedraza-Meléndez; Joaqufn Quiroz-Mercado; Daniel Regalado-Santiago; Silvia Rivero-Arredondo; Erika Sierra-Atanacio; Fernando Gonzâlez-Domfnguez; Lucia Hernândez-Rivas, Irma L6pez-Martfnez; Ernesto Ramîrez-Gonzâlez; Maribel Gonzâlez-Villa
EPI ISL 17703571	LESP Sinaloa	Instituto de Diagnostico y Referencia Epidemiologicos (INDRE)	Abril Rodrfguez-Maldonado; Claudia Wong-Arémbula; Felipe Arguijo-Perez; Helios Cârdenas-Hernàndez; Carmen Castro-Méndez; Lidia Garda-Torres; Ruth Madera-Sandoval; América Mandujano-Martfnez; Nancy Martfnez-Velâzquez; Mireya Mederos-Michel; Angélica Pedraza-Meléndez; Joaqufn Quiroz-Mercado; Daniel Regalado-Santiago; Silvia Rivero-Arredondo; Erika Sierra-Atanacio; Fernando Gonzâlez-Domfnguez; Lucia Hernândez-Rivas, Irma L6pez-Martinez; Ernesto Ramfrez-Gonzâlez: Maribel Gonzâlez-Villa
EPI ISL 17703572	LESP State of Mexico	Instituto de Diagnostico y Referencia Epidemiologicos (INDRE)	Abril Rodrfguez-Maldonado; Claudia Wong-Arâmbula; Felipe Arguijo-Perez; Helios Cârdenas-Hernilndez; Carmen Castro-Méndez; Lidia Garda-Torres; Ruth Madera-Sandoval; América Mandujano-Martfnez; Nancy Martfnez-Velilzquez; Mireya Mederos-Michel; Angélica Pedraza-Meléndez; Joaqufn Quiroz-Mercado; Daniel Regalado-Santiago; Silvia Rivero-Arredondo; Erika Sierra-Atanacio; Fernando Gonzélez-Domfnguez; Lucia Hernândez-Rivas, Irma L6pez-Martfnez; Ernesto Ramirez-Gonzâlez; Maribel Gonzâlez-Villa
EPI_ISL_I 7703573	LESP Mexico City	Instituto de Diagnostico y Referencia Epidemiologicos (INDRE)	Abril Rodrfguez-Maldonado; Claudia Wong-Arémbula; Felipe Arguijo-Perez; Helios Cârdenas-Hernândez; Carmen Castro-Méndez; Lidia Garda-Torres; Ruth Madera-Sandoval; América Mandujano-Martfnez; Nancy Martfnez-Velâzquez; Mireya Mederos-Michel; Angélica Pedraza-Meléndez; Joaqufn Quiroz-Mercado; Daniel Regalado-Santiago: Silvia Rivero-Arredondo: Erika Sierra-Atanacio; Fernando Gonzâlez-Domfnguez; Lucia Hernândez-Rivas, Irma L6pez-Martfnez; Ernesto Ramfrez-Gonzâlez; Maribel Gonzâlez-Villa
EPI ISL 17703574	LESP State of Mexico	Instituto de Diagnostico y Referencia Epidemiologicos (INDRE)	Abril Rodrfguez-Maldonado; Claudia Wong-Arâmbula; Felipe Arguijo-Perez; Helios Cârdenas-Hernândez; Carmen Castro-Méndez; Lidia Garda-Torres; Ruth Madera-Sandoval; América Mandujano-Martfnez; Nancy Martfnez-Velâzquez; Mireya Mederos-Michel; Angélica Pedraza-Meléndez; Joaqufn Ouiroz-Mercado; Daniel Regalado-Santiago; Silvia Rivero-Arredondo; Erika Sierra-Atanacio; Fernando Gonzélez-Domfnguez; Lucia Hernéndez-Rivas, Irma L6pez-Martfnez; Ernesto Ramfrez-Gonzâlez; Maribel Gonzâlez-Villa
EPI_ISL_I 770357S	LESP Mexico City	Instituto de Diagnostico y Referencia Epidemiologicos (INDRE)	Abril Rodrfguez-Maldonado; Claudia Wong-Arâmbula; Felipe Arguijo-Perez; Helios Cârdenas-Hernândez; Carmen Castro-Méndez; Lidia Garda-Torres; Ruth Madera-Sandoval; América Mandujano-Martfnez; Nancy Martfnez-Velàzquez; Mireya Mederos-Michel; Angélica Pedraza-Meléndez; Joaqufn Quiroz-Mercado; Daniel Regalado-Santiago: Silvia Rivero-Arredondo: Erika Sierra-Atanacio; Fernando Gonzâlez-Domfnguez; Lucia Hernândez-Rivas, Irma L6pez-Martfnez; Ernesto Ramirez-Gonzâlez; Maribel Gonzâlez-Villa
EPI ISL 17703576	LESP Quintana Roo	Instituto de Diagnostico y Referencia Epidemiologicos (INDRE)	Abril Rodrfguez-Maldonado: Claudia Wong-Arâmbula; Felipe Arguijo-Perez; Helios Cârdenas-Hernilndez: Carmen Castro-Méndez: Lidia Garda-Torres; Ruth Madera-Sandoval; América Mandujano-Martfnez: Nancy Martfnez-Velilzquez; Mireya Mederos-Michel; Angélica Pedraza-Meléndez; Joaqufn Quiroz-Mercado; Daniel Regalado-Santiago; Silvia Rivero-Arredondo; Erika Sierra-Atanacio; Fernando Gonzâlez-Domfnguez; Lucia Hernândez-Rivas, Irma L6pez-Martfnez; Ernesto Ramfrez-Gonzâlez; Maribel Gonzâlez-Villa
EPI ISL 17703577	LESP Mexico City	Instituto de Diagnostico y Referencia Epidemiologicos (INDRE)	Abril Rodr/guez-Maldonado; Claudia Wong-Arâmbula; Felipe Arguijo-Perez; Helios Cârdenas-Hernândez; Carmen Castro-Méndez; Lidia Garda-Torres; Ruth Madera-Sandoval; América Mandujano-Martfnez; Nancy Martfnez-Velâzquez; Mireya Mederos-Michel; Angélica Pedraza-Meléndez; Joaqufn Quiroz-Mercado; Daniel Regalado-Santiago: Silvia Rivero-Arredondo: Erika Sierra-Atanacio; Fernando Gonzâlez-Domfnguez: Lucia Hernândez-Rivas, Irma L6pez-Martfnez: Ernesto Ramfrez-Gonzâlez; Maribel Gonzâlez-Villa
EPI_ISL_I 7703578	LESP Yucatan	Instituto de Diagnostico y Referencia Epidemiologicos (INDRE)	Abril Rodrfguez-Maldonado; Claudia Wong-Artimbula; Felipe Arguijo-Perez; Helios Cârdenas-Hernândez; Carmen Castro-Méndez; Lidia Garda-Torres; Ruth Madera-Sandoval; América Mandujano-Martfnez; Nancy Martfnez-Velâzquez; Mireya Mederos-Michel; Angélica Pedraza-Meléndez; Joaqufn Quiroz-Mercado; Daniel Regalado-Santiago; Silvia Rivero-Arredondo; Erika Sierra-Atanacio; Fernando Gonzâlez-Domfnguez; Lucia Hernândez-Rivas, Irma L6pez-Martfnez; Ernesto Ramfrez-Gonzâlez: Maribel Gonzâlez-Villa
EPI ISL 17703579	LESP Quintana Roo	Instituto de Diagnostico y Referencia Epidemiologicos (INDRE)	Abril Rodrfguez-Maldonado; Claudia Wong-Arâmbula; Felipe Arguijo-Perez; Helios Cârdenas-Hernilndez; Carmen Castro-Méndez; Lidia Garda-Torres; Ruth Madera-Sandoval; América Mandujano-Martfnez; Nancy Martfnez-Velilzquez; Mireya Mederos-Michel; Angélica Pedraza-Meléndez; Joaqufn Quiroz-Mercado; Daniel Regalado-Santiago; Silvia Rivero-Arredondo; Erika Sierra-Atanacio; Fernando Gonztilez-Domfnguez; Lucia Herntindez-Rivas, Irma L6pez-Martfnez; Ernesto Ramfrez-Gonzâlez; Maribel Gonzâlez-Villa
EPI_ISL_I 7703580	LESP Hidalgo	Instituto de Diagnostico y Referencia Epidemiologicos (INDRE)	Abril Rodrfguez-Maldonado; Claudia Wong-Arâmbula; Felipe Arguijo-Perez; Helios Cérdenas-Hernândez; Carmen Castro-Méndez; Lidia Garda-Torres; Ruth Madera-Sandoval; América Mandujano-Martfnez; Nancy Martfnez-Velâzquez; Mireya Mederos-Michel; Angélica Pedraza-Meléndez; Joaqufn Quiroz-Mercado; Daniel Regalado-Santiago: Silvia Rivero-Arredondo: Erika Sierra-Atanacio; Fernando Gonzâlez-Domfnguez; Lucia Hernândez-Rivas, Irma L6pez-Martfnez; Ernesto Ramfrez-Gonzâlez; Maribel Gonzâlez-Villa
EPI ISL 17703581	LESP Yucatan	Instituto de Diagnostico y Referencia Epidemiologicos (INDRE)	Abril Rodrfguez-Maldonado; Claudia Wong-Arémbula; Felipe Arguijo-Perez; Helios Cârdenas-Hernilndez; Carmen Castro-Méndez: Lidia Garda-Torres; Ruth Madera-Sandoval; América Mandujano-Martfnez; Nancy Martfnez-Velâzquez; Mireya Mederos-Michel; Angélica Pedraza-Meléndez; Joaqufn Ouiroz-Mercado; Daniel Regalado-Santiago; Silvia Rivero-Arredondo; Erika Sierra-Atanacio; Fernando Gonzâlez-Domfnguez; Lucia Hernândez-Rivas, Irma L6pez-Martfnez; Ernesto Ramfrez-Gonzâlez; Maribel Gonzâlez-Villa
EPI_ISL_I7703582	LESP Nuevo Leon	Instituto de Diagnostico y Referencia Epidemiologicos (INDRE)	Abril Rodrfguez-Maldonado; Claudia Wong-Arâmbula; Felipe Arguijo-Perez; Helios Cârdenas-Hernândez; Carmen Castro-Méndez; Lidia Garda-Torres; Ruth Madera-Sandoval; América Mandujano-Martfnez; Nancy Martfnez-Velâzquez; Mireya Mederos-Michel; Angélica Pedraza-Meléndez; Joaqufn Quiroz-Mercado; Daniel Regalado-Santiago: Silvia Rivero-Arredondo: Erika Sierra-Atanacio; Fernando Gonzélez-Domfnguez; Lucia Hernândez-Rivas, Irma L6pez-Martfnez; Ernesto Ramirez-Gonzâlez; Maribel Gonzâlez-Villa
EPI_ISL_I7703583	LESP Quintana Rao	Instituto de Diagnostic□y Referencia Epidemiologicos (INDRE)	Abril Rodrfguez-Maldonado: Claudia Wong-Artimbula; Felipe Arguijo-Perez; Helios Cârdenas-Hernândez: Carmen Castro-Méndez: Lidia Garda-Torres; Ruth Madera-Sandoval; América Mandujano-Martfnez: Nancy Martfnez-Velâzquez; Mireya Mederos-Michel; Angélica Pedraza-Meléndez; Joaqufn Quiroz-Mercado; Daniel Regalado-Santiago; Silvia Rivero-Arredondo; Erika Sierra-Atanacio; Fernando Gonzâlez-Domfnguez; Lucia Hernândez-Rivas, Irma L6pez-Martfnez; Ernesto Ramfrez-Gonzâlez: Maribel Gonzâlez-Villa
EPI ISL 17703584	LESP Chihuahua	Instituto de Diagnostico y Referencia Epidemiologicos (INDRE)	Abril Rodrfguez-Maldonado; Claudia Wong-Arâmbula; Felipe Arguijo-Perez; Helios Cârdenas-Hernândez; Carmen Castro-Méndez; Lidia Garda-Torres; Ruth Madera-Sandoval; América Mandujano-Martfnez; Nancy Martfnez-Velâzquez; Mireya Mederos-Michel; Angélica Pedraza-Meléndez; Joaqufn Quiroz-Mercado; Daniel Regalado-Santiago: Silvia Rivero-Arredondo: Erika Sierra-Atanacio; Fernando Gonztilez-Domfnguez; Lucia Herntindez-Rivas, Irma L6pez-Martfnez: Ernesto Ramfrez-Gonzâlez; Maribel Gonzâlez-Villa
EPI_ISL_I 7703585	LESP Yucatan	Instituto de Diagnostico y Referencia Epidemiologicos (INDRE)	Abril Rodrfguez-Maldonado; Claudia Wong-Arâmbula; Felipe Arguijo-Perez; Helios Ctirdenas-Hernândez; Carmen Castro-Méndez; Lidia Garda-Torres; Ruth Madera-Sandoval; América Mandujano-Martfnez; Nancy Martfnez-Velâzquez; Mireya Mederos-Michel; Angélica Pedraza-Meléndez; Joaqufn Quiroz-Mercado; Daniel Regalado-Santiago; Silvia Rivero-Arredondo; Erika Sierra-Atanacio; Fernando Gonzâlez-Domfnguez; Lucia Hernândez-Rivas, Irma L6pez-Martfnez; Ernesto Ramfrez-Gonzâlez; Maribel Gonzâlez-Villa
EPI ISL 17703586, EPI ISL 17703587	LESP Nuevo Leon	Instituto de Diagnostico y Referencia Epidemiologicos (INDRE)	Abril Rodrfguez-Maldonado: Claudia Wong-Arâmbula; Felipe Arguijo-Perez; Helios Cârdenas-Hernândez; Carmen Castro-Méndez; Lidia Garda-Torres; Ruth Madera-Sandoval; América Mandujano-Martfnez: Nancy Martfnez-Velâzquez; Mireya Mederos-Michel; Angélica Pedraza-Meléndez; Joaqufn Quiroz-Mercado; Daniel Regalado-Santiago; Silvia Rivero-Arredondo; Erika Sierra-Atanacio; Fernando Gonztilez-Domfnguez; Lucia Hertindez-Rivas, Irma L6pez-Martfnez: Ernesto Ramirez-Gonzâlez; Maribel Gonzâlez-Villa
EPI_ISL_I 7703588, EPI_ISL_I 7703589	LESP Quintana Rao	Instituto de Diagnostic□y Referencia Epidemiologicos (INDRE)	Abril Rodrfguez-Maldonado; Claudia Wong-Arâmbula; Felipe Arguijo-Perez; Helios Cârdenas-Hernândez; Carmen Castro-Méndez; Lidia Garda-Torres; Ruth Madera-Sandoval; América Mandujano-Martfnez; Nancy Martfnez-Velâzquez; Mireya Mederos-Michel; Angélica Pedraza-Meléndez; Joaqufn Quiroz-Mercado; Daniel Regalado-Santiago; Silvia Rivero-Arredondo; Erika Sierra-Atanacio; Fernando Gonzâlez-Domfnguez; Lucia Hernândez-Rivas, Irma L6pez-Martfnez; Ernesto Ramfrez-Gonzâlez: Maribel Gonzâlez-Villa
EPI ISL 17703590	LESP State of Mexico	Instituto de Diagnostico y Referencia Epidemiologicos (INDRE)	Abril Rodrfguez-Maldonado: Claudia Wong-Arâmbula; Felipe Arguijo-Perez; Helios Cârdenas-Hernândez; Carmen Castro-Méndez; Lidia Garda-Torres; Ruth Madera-Sandoval; América Mandujano-Martfnez: Nancy Martfnez-Velâzquez; Mireya Mederos-Michel; Angélica Pedraza-Meléndez; Joaqufn Ouiroz-Mercado; Daniel Regalado-Santiago; Silvia Rivero-Arredondo; Erika Sierra-Atanacio; Fernando Gonztilez-Domfnguez; Lucia Hernândez-Rivas, Irma L6pez-Martfnez: Ernesto Ramfrez-Gonzâlez: Maribel Gonzâlez-Villa
EPI_ISL_I7703591	LESP Campeche	Instituto de Diagnostico y Referencia Epidemiologicos (INDRE)	Abril Rodrfguez-Maldonado: Claudia Wong-Arémbula; Felipe Arguijo-Perez; Helios Cârdenas-Hernândez: Carmen Castro-Méndez: Lidia Garda-Torres; Ruth Madera-Sandoval; América Mandujano-Martinez: Nancy Martfnez-Velâzquez; Mireya Mederos-Michel; Angélica Pedraza-Meléndez; Joaqufn Quiroz-Mercado; Daniel Regalado-Santiago; Silvia Rivero-Arredondo; Erika Sierra-Atanacio; Fernando Gonzâlez-Domfnguez; Lucia Hernândez-Rivas, Irma L6pez-Martfnez; Ernesto Ramfrez-Gonzâlez; Maribel Gonzâlez-Villa
EPI ISL 17703592	LESP Nuevo Leon	Instituto de Diagnostico y Referencia Epidemiologicos (INDRE)	Abril Rodrfguez-Maldonado; Claudia Wong-Arâmbula; Felipe Arguijo-Perez; Helios Cârdenas-Hernilndez; Carmen Castro-Méndez: Lidia Garda-Torres; Ruth Madera-Sandoval; América Mandujano-Martfnez: Nancy Martfnez-Velilzquez; Mireya Mederos-Michel; Angélica Pedraza-Meléndez; Joaqufn Quiroz-Mercado; Daniel Regalado-Santiago; Silvia Rivero-Arredondo; Erika Sierra-Atanacio; Fernando Gonzélez-Domfnguez; Lucia Hernéndez-Rivas, Irma L6pez-Martinez: Ernesto Ramfrez-Gonzâlez; Maribel Gonzâlez-Villa
EPI_ISL_I 7703593, EPI_ISL_I 7703594	LESP Jalisco	Instituto de Diagnostico y Referencia Epidemiologicos (INDRE)	Abril Rodrfguez-Maldonado: Claudia Wong-Arâmbula; Felipe Arguijo-Perez; Helios Cârdenas-Hernândez; Carmen Castro-Méndez: Lidia Garda-Torres; Ruth Madera-Sandoval; América Mandujano-Mart/nez: Nancy Martfnez-Velâzquez; Mireya Mederos-Michel: Angélica Pedraza-Meléndez; Joaqufn Quiroz-Mercado; Daniel Regalado-Santiago: Silvia Rivero-Arredondo: Erika Sierra-Atanacio; Fernando Gonzâlez-Domfnguez; Lucia Hernândez-Rivas, Irma L6pez-Martfnez; Ernesto Ramfrez-Gonzâlez: Maribel Gonzâlez-Villa
EPI ISL 17703595, EPI ISL 17703596	LESP Nuevo Lean	Instituto de Diagnostic□y Referencia Epidemiologicos (INDRE)	Abril Rodrfguez-Maldonado; Claudia Wong-Arâmbula; Felipe Arguijo-Perez; Helios Cârdenas-Hernândez; Carmen Castro-Méndez; Lidia Garda-Torres; Ruth Madera-Sandoval; América Mandujano-Martfnez; Nancy Martfnez-Velâzquez; Mireya Mederos-Michel; Angélica Pedraza-Meléndez; Joaqufn Quiroz-Mercado; Daniel Regalado-Santiago; Silvia Rivero-Arredondo; Erika Sierra-Atanacio; Fernando Gonzâlez-Domfnguez: Lucia Hernândez-Rivas, Irma L6pez-Mart/nez: Ernesto Ramfrez-Gonzâlez: Maribel Gonzâlez-Villa
EPI ISL 17703597	Laboratorio Estatal de Salud PLIblica Nuevo Leon	Instituto de Diagnostico y Referencia Epidemiologicos (INDRE)	Abril Rodrfguez-Maldonado: Claudia Wong-Arâmbula; Felipe Arguijo-Perez; Helios Cârdenas-Hernândez; Carmen Castro-Méndez; Lidia Garda-Torres; Ruth Madera-Sandoval; América Mandujano-Martfnez; Nancy Martfnez-Velâzquez; Mireya Mederos-Michel; Angélica Pedraza-Meléndez; Joaqufn Quiroz-Mercado; Daniel Regalado-Santiago; Silvia Rivero-Arredondo; Erika Sierra-Atanacio; Fernando Gonztilez-Domfnguez; Lucia Hernândez-Rivas, Irma L6pez-Martfnez; Ernesto Ramirez-Gonzâlez: Maribel Gonzâlez-Villa
EPI_ISL_I7703598	LESP Quintana Rao	Instituto de Diagnostic□y Referencia Epidemiologicos (INDRE)	Abril Rodrfguez-Maldonado: Claudia Wong-Arâmbula; Felipe Arguijo-Perez; Helios Cârdenas-Hernândez; Carmen Castro-Méndez; Lidia Garda-Torres; Ruth Madera-Sandoval; América Mandujano-Martfnez: Nancy Martfnez-Velâzquez; Mireya Mederos-Michel; Angélica Pedraza-Meléndez; Joaqufn Quiroz-Mercado; Daniel Regalado-Santiago: Silvia Rivero-Arredondo: Erika Sierra-Atanacio; Fernando Gonzâlez-Domfnguez; Lucia Hernândez-Rivas, Irma L6pez-Martfnez; Ernesto Ramfrez-Gonzâlez; Maribel Gonzâlez-Villa
EPI ISL 17703599, EPI ISL 17703600, EPI ISL 17703601, EPC_ISL I7703602, EPI ISL I7703603, EPI ISL I7703604	LESP Nuevo Lean	Instituto de Diagnostico y Referencia Epidemiologicos (INDRE)	Abril Rodrfguez-Maldonado; Claudia Wong-Arâmbula; Felipe Arguijo-Perez; Helios Cârdenas-Hernândez; Carmen Castro-Méndez; Lidia Garda-Torres; Ruth Madera-Sandoval; América Mandujano-Martfnez; Nancy Martfnez-Velâzquez; Mireya Mederos-Michel; Angélica Pedraza-Meléndez; Joaqufn Quiroz-Mercado; Daniel Regalado-Santiago; Silvia Rivero-Arredondo; Erika Sierra-Atanacio; Fernando Gonztilez-Domfnguez; Lucia Herntindez-Rivas, Irma L6pez-Martfnez; Ernesto Ramfrez-Gonzâlez; Maribel Gonzâlez-Villa
EPI_ISL_I 7703605	LESP Chihuahua	Instituto de Diagnostico y Referencia Epidemiologicos (INDRE)	Abril Rodrfguez-Maldonado: Claudia Wong-Arâmbula; Felipe Arguijo-Perez; Helios Cârdenas-Hernândez: Carmen Castro-Méndez: Lidia Garda-Torres; Ruth Madera-Sandoval; América Mandujano-Martinez: Nancy Martfnez-Velàzquez; Mireya Mederos-Michel; Angélica Pedraza-Meléndez; Joaqufn Quiroz-Mercado; Daniel Regalado-Santiago: Silvia Rivero-Arredondo: Erika Sierra-Atanacio; Fernando Gonzâlez-Domfnguez; Lucia Hernândez-Rivas, Irma L6pez-Martfnez; Ernesto Ramfrez-Gonzâlez; Maribel Gonzâlez-Villa
EPI ISL 17703606	LESP Nuevo Lean	Instituto de Diagnostico y Referencia Epidemiologicos (INDRE)	Abril Rodrfguez-Maldonado; Claudia Wong-Arâmbula; Felipe Arguijo-Perez; Helios Cârdenas-Hernilndez; Carmen Castro-Méndez: Lidia Garda-Torres; Ruth Madera-Sandoval; América Mandujano-Martfnez; Nancy Martfnez-Velilzquez; Mireya Mederos-Michel; Angélica Pedraza-Meléndez; Joaqufn Quiroz-Mercado; Daniel Regalado-Santiago; Silvia Rivero-Arredondo; Erika Sierra-Atanacio; Fernando Gonzâlez-Domfnguez; Lucia Hernândez-Rivas, Irma L6pez-Martfnez; Ernesto Ramfrez-Gonzâlez; Maribel Gonzâlez-Villa
EPI_ISL_I7703607, EPI_ISL_I7703608, EPI_ISL_17703609,	LESP Mexico City	Instituto de Diagnostico y Referencia Epidemiologicos	Abril Rodr/guez-Maldonado: Claudia Wong-Arâmbula; Felipe Arguijo-Perez; Helios Cilrdenas-Hernândez; Carmen Castro-Méndez; Lidia Garda-Torres; Ruth Madera-Sandoval; América Mandujano-Mart/nez: Nancy Martfnez-Velâzquez;

EPI_ISL_l7703610		(INORE)	Mireya Mederos-Michel; Angélica Pedraza-Meléndez; Joaqufn Quiroz-Mercado; Daniel Regalado-Santiago; Silvia Rivero-Arredondo; Erika Sierra-Atanacio; Fernando Gonzâlez-Oomfnguez; Lucia Hernândez-Rivas, Irma L6pez-Martfnez; Ernesto Ramirez-Gonzâlez; Maribel Gonzâlez-Villa
EPI_ISL_l7703611	LESP Tlaxcala	Instituto de Diagnostico y Referencia Epidemiologicos (INORE)	Abril Rodrfguez-Maldonado; Claudia Wong-Ar.3mbula; Felipe Arguijo-Perez; Helios C.3rdenas-Hernândez: Carmen Castro-Méndez: Lidia Garcia-Torres; Ruth Madera-Sandoval; América Mandujano-Martfnez: Nancy Martfnez-Velâzquez; Mireya Mederos-Michel; Angélica Pedraza-Meléndez; Joaqufn Quiroz-Mercado; Daniel Regalado-Santiago; Silvia Rivero-Arredondo; Erika Sierra-Atanacio; Fernando Gonzâlez-Oomfnguez; Lucia Hernândez-Rivas, Irma L6pez-Mart/nez; Ernesto Ramfrez-Gonzâlez: Maribel Gonzâlez-Villa
EPI_ISL_l7703612	LESP Nuevo Lean	Instituto de Oiagnostico y Referencia Epidemiologicos (INDRE)	Abril Rodrfguez-Maldonado; Claudia Wong-Ar.3mbula; Felipe Arguijo-Perez; Helios Cârdenas-Hernândez: Carmen Castro-Méndez: Lidia Garcia-Torres; Ruth Madera-Sandoval; América Mandujano-Martfnez: Nancy Martfnez-Velâzquez; Mireya Mederos-Michel; Angélica Pedraza-Meléndez; Joaqufn Quiroz-Mercado; Daniel Regalado-Santiago; Silvia Rivero-Arredondo; Erika Sierra-Atanacio; Fernando Gonz.3lez-Domfnguez; Lucia Hern.3ndez-Rivas, Irma L6pez-Martfnez: Ernesto Ramfrez-Gonztilez; Maribel Gonztilez-Villa
EPI_ISL_l7703743, EPI_JSL_l7703744	Parasitology Laboratory, Institute of Tropical Medicine of Sac Paulo, School of Medicine, University of Sao Paulo	Parasitology Laboratory	Raissa Heloisa de Araujo Eliodoro, Ingra Clara Morales, Ester Cerdeira Sabino
EPI_ISL_l7703745	Parasitology Laboratory, Institute of Tropical Medicine of Sac Paulo, Scheel of Medicine, University of Sao Paulo	Parasitology Laboratory	Raissa Heloisa de Araujo Eliodoro, Ingra Morales Clara, Ester Cerdeira Sabino
EPI_ISL_l 7703746	Parasitology Laboratory, 1nstitute of Tropical Medicine of Sao Paulo, School of Medicine, University of Sao Paulo	Parasitology Laboratory	Raissa Heloisa de Araujo Eliodoro, Ingra Clara Morales, Ester Cerdeira Sabino
EPI_ISL_l7718458	Department of Virology, National Institute of Health, Islamabad, Pakistan	Department of Virology, National Institute of Health, Islamabad, Pakistan	Massab Umair, Muhammad Ammar, Syed Adnan Haider, Rabia Hakim, Qasim Malik, Muhammad Salman, Ghazala Parveen, and Naseem Akhtar
EPI_ISL_l 7722468	Tokyo Metropolitan Institute of Public Health	Tokyo Metropolitan Institute of Public Health	Fumi Kasuya, Wakaba Okada, Ryota Kumagai, Sachiko Harada, Arisa Amano, Michiya Hasegawa, Mami Nagashima, Kenji Sadamasu
EPI ISL 17736865, EPI ISL 17736866, EPJ ISL 17736867, EPI JSL 17736868, EPI ISL 17736869, EPI ISL 17736870, EPI ISL 17736871, EPI ISL 17736872, EPI ISL 17736873, EPI ISL 17736874, EPI ISL 17736875, EPI ISL 17736876, EPI ISL 17736877, EPI ISL 17736878, EPI ISL 17736879, EPI ISL 17736880, EPI ISL 17736881, EPI ISL 17736882, EPI ISL 17736883, EPI ISL 17736884, EPI=ISL)7736885, EPI=ISL)7736886, EPÜSL)7736887, EPI=ISL)7736888, EPI=ISL)7736889, EPI=ISL)7736890, EPI=ISL)7736891			
see above	Charité Universitatsmedizin Berlin, Institute for Virology/Laboratory Berlin	Charité Universitatsmedizin Berlin, Institute for Virology	Terry C. Jones, Julia Melchert, Barbara Mühlemann, Talitha Veith, Ji:irn Beheim-Schwarzbach, Julia Tesch, Marie Luisa Schmidt, Felix Walper, Tobias Bleicker, Caroline Isner, Frieder pfafflin, Ricardo Niklas Werner, Victor M. Carman, Christian Drosten
EPI_ISL_l7737466, EPI_ISL_l7737467, EPJ_ISL_l 7737468, EPI_ISL_l7737469, EPI_ISL_l7737470, EPI_ISL_l7737471, EPI_ISL_l7737472, EPI_ISL_l7737473, EPI_ISL_l 7737474, EPI_ISL_l 7737475, EPI_ISL_l7737476, EPI_ISL_l7737477, EPI_ISL_l 7737478, EPI_ISL_l7737479, EPI_ISL_l7737480, EPI_ISL_l7737481, EPI_ISL_l7737482, EPI_ISL_l 7737483, EPI_ISL_l7737484, EPI_ISL_l7737485, EPI ISL 17737486, EPI ISL 17737488, EPI ISL 17737489, EPI ISL 17737490, EPI ISL 17737491. EPI ISL 17737492, EPI ISL 17737493, EPI ISL 17737495, EPI ISL 17737496, EPI ISL 17737497, EPI ISL 17737498, EPI ISL 17737499, EPI ISL 17737500, EPI ISL 17737501, EPI ISL 17737502, EPI ISL 17737503, EPI ISL 17737504, EPI ISL 17737505, EPI ISL 17737506, EPI ISL 17737507, EPI-ISL-17737508, EPI-ISL-17737509, EPJ-ISL-17737510, EPI-JSL-17737511, EPI-ISL-17737512, EPI-ISL-17737513, EPI-ISL-17737514, EPI-ISL-17737515, EPI-ISL-17737516, EPI-ISL-17737517, EPI-ISL-17737518, EPI-ISL-17737519, EPI-ISL-17737520, EPI-ISL-17737521, EPI-ISL-17737522, EPI-ISL-17737523, EPI-ISL-17737524, EPI-ISL-17737525, EPI-ISL-17737526, EPI-ISL-17737527, EPCISL)7737528. EPI=I5L)7737529, EPÜSL)7737530, EPCI5L) 7737531, EPI=ISL) 7737532, EPCISL)7737533. EPI=I5L)7737534, EPÜSL)7737535, EPCI5L) 7737536, EPI=ISL) 7737537, EPCISL)7737538. EPI=I5L)7737539, EPÜSL)7737540, EPCI5L)7737541, EPI=ISL) 7737542, EPCISL)7737543. EPI=I5L)7737544, EPÜSL)7737545, EPCI5L)7737546, EPI=ISL) 7737547, EPI_ISL_l7737548, EPI_ISL_l7737549, EPJ_ISL_l7737550, EPI_ISL_l7737551, EPI_ISL_l7737553, EPI_ISL_l7737555, EPI_ISL_l7737556			
see above	Public Health Ontario	Public Health Ontario	Isabel 5, Eshaghi A, Ouvvuri VR, Gubbay JB, Cronin K, Ii A, Hasso M, Clark ST, Hopkins JP, Patel SN, Braukmann TWA
EPI_ISL_l7762484, EPI_I5L_l 7762485	Tokyo Metropolitan Institute of Public Health	Tokyo Metropolitan Institute of Public Health	Fumi Kasuya, Wakaba Okada. Ryota Kumagai, Sachiko Harada, Arisa Amano, Michiya Hasegawa, Mami Nagashima, Kenji 5adamasu
EPI_I5L_l7779992	Servicio de Microbiologia Hospital Ramon y Cajal	Servicio de Microbiologia Hospital Ramon y Cajal	Ponce-Alonso M, Martinez-Garda L, Olavarrieta L, Galan JC
EPI_ISL_l7789831	Delaware Public Health Lab	Delaware Public Health Lab	Moneeb Bajwa & Holly Miller
EPI_ISL_l7793219	Microbiology, Immunology and Transplantation, KU Leuven	Microbiology, Immunology and Transplantation, KU Leuven	Wawina-Bokalanga,T., Vanmechelen,B., Logist.A.-5., Bloemen,M. and Maes,P.
EPI_ISL_l7793220	Microbiology, Immunology and Transplantation, KU Leuven	Microbiology, Immunology and Transplantation, KU Leuven	Vanmechelen,B., Wawina-Bokalanga,T., Logist.A.-5., Bloemen,M. and Maes,P.
EPI_ISL_l7793221, EPI_ISL_l 7793222	Microbiology, Immunology and Transplantation, KU Leuven	Microbiology, Immunology and Transplantation, KU Leuven	Wawina-Bokalanga,T., Vanmechelen,B., Logist,A.-5., Bloemen,M. and Maes,P.
EPI_ISL_l 7793223, EPI_ISL_l 7793224. EPI_ISL_l 7793225	Microbiology, Immunology and Transplantation, KU Leuven	Microbiology, Immunology and Transplantation, KU Leuven	Vanmechelen,B., Wawina-Bokalanga,T., Logist,A.-5., Bloemen,M. and Maes,P.
EPI_ISL_l7793226	Microbiology, Immunology and Transplantation, KU Leuven	Microbiology, Immunology and Transplantation, KU Leuven	Wawina-Bokalanga,T., Vanmechelen,B., Logist,A.-5., Bloemen,M. and Maes,P.
EPI_ISL_l7793227	Microbiology, Immunology and Transplantation, KU Leuven	Microbiology, Immunology and Transplantation, KU Leuven	Vanmechelen,B., Wawina-Bokalanga,T., Logist.A.-5., Bloemen,M. and Maes,P.
EPJ_ISL_l7793229, EPI_JSL_l7793230	Microbiology, Immunology and Transplantation, KU Leuven	Microbiology, Immunology and Transplantation, KU Leuven	Wawina-Bokalanga,T., Vanmechelen,B., Logist,A.-5., Sinnesael,R., Ysebaert,L., Verlinden,J., Van Holm,B., Bloemen,M. and Maes,P.
EPI_ISL_l7793231	Microbiology, Immunology and Transplantation, KU Leuven	Microbiology, Immunology and Transplantation, KU Leuven	Vanmechelen,B., Wawina-Bokalanga,T., Logist,A.-5., Bloemen,M. and Maes,P.
EPI_ISL_l7793232	Microbiology, Immunology and Transplantation, KU Leuven	Microbiology, Immunology and Transplantation, KU Leuven	Wawina-Bokalanga,T., Vanmechelen,B., Logist,A.-5., Bloemen,M. and Maes,P.
EPI_ISL_l7793233, EPI_ISL_l 7793234	Microbiology, Immunology and Transplantation, KU Leuven	Microbiology, Immunology and Transplantation, KU Leuven	Vanmechelen,B., Wawina-Bokalanga,T., Logist,A.-5., Bloemen,M. and Maes,P.
EPI_ISL_l 7793235	Microbiology, Immunology and Transplantation, KU Leuven	Microbiology, Immunology and Transplantation, KU Leuven	Wawina-Bokalanga,T., Vanmechelen,B., Logist.A.-5., Bloemen,M. and Maes,P.
EPI_ISL_l7793236, EPI_ISL_l7793238, EPI_ISL_l7793239	Microbiology, Immunology and Transplantation, KU Leuven	Microbiology, Immunology and Transplantation, KU Leuven	Vanmechelen,B., Wawina-Bokalanga,T., Logist.A.-5., Bloemen,M. and Maes,P.
EPI_ISL_l7793240	Microbiology, Immunology and Transplantation, KU Leuven	Microbiology, Immunology and Transplantation, KU Leuven	Vanmechelen,B., Wawina-Bokalanga,T., Logist.A.-5., Sinnesael,R., Ysebaert,L., Verlinden,J., Van Holm,B., Bloemen,M. and Maes,P.
EPI_ISL_l7793241	Microbiology, Immunology and Transplantation, KU Leuven	Microbiology, Immunology and Transplantation, KU Leuven	Wawina-Bokalanga,T., Vanmechelen,B., Logist,A.-5., Bloemen,M. and Maes,P.
EPI_ISL_l7793242. EPI_ISL_l 7793243	Microbiology. Immunology and Transplantation, KU Leuven	Microbiology, Immunology and Transplantation, KU Leuven	Vanmechelen.B., Wawina-Bokalanga,T., Logist,A.-5., Bloemen,M. and Maes,P.
EPJ_ISL_l7793244. EPI_ISL_l7793245	Microbiology. Immunology and Transplantation, KU Leuven	Microbiology. Immunology and Transplantation, KU Leuven	Wawina-Bokalanga,T., Vanmechelen,B., Logist,A.-5., Bloemen,M. and Maes,P
EPI_ISL_l7793246	Microbiology, Immunology and Transplantation, KU Leuven	Microbiology, Immunology and Transplantation, KU Leuven	Wawina-Bokalanga,T., Vanmechelen,B., Logist,A.-5., Horemans,M., Ysebaert,L., Verlinden,J., Van Holm,B., Bloemen,M. and Maes,P
EPI_ISL_l7793247, EPI_ISL_l 7793248, EPI_ISL_l7793250	Microbiology, Immunology and Transplantation, KU Leuven	Microbiology, Immunology and Transplantation, KU Leuven	Vanmechelen,B., Wawina-Bokalanga,T., Logist,A.-5., Bloemen,M. and Maes,P.
EPI_ISL_l7793251	Microbiology, Immunology and Transplantation, KU Leuven	Microbiology, Immunology and Transplantation, KU Leuven	Wawina-Bokalanga,T., Vanmechelen,B., Logist,A.-5., Bloemen,M. and Maes,P.
EPI_ISL_l7793252, EPI_ISL_l 7793253	Microbiology, Immunology and Transplantation, KU Leuven	Microbiology, Immunology and Transplantation, KU Leuven	Vanmechelen,B., Wawina-Bokalanga,T., Logist,A.-5., Bloemen,M. and Maes,P.
EPI_ISL_l 7793254	Microbiology. Immunology and Transplantation, KU Leuven	Microbiology, Immunology and Transplantation, KU Leuven	Wawina-Bokalanga,T., Vanmechelen.B.. logist.A.-5.. Ysebaert,L., Horemans,M., Verlinden,J.. VanHolm,B., Bloemen,M. and Maes,P.
EPI_ISL_l7793255	Microbiology, Immunology and Transplantation, KU Leuven	Microbiology, Immunology and Transplantation, KU Leuven	Wawina-Bokalanga,T., Vanmechelen,B., Logist.A.-5.. Sinnesael,R., Ysebaert,L.. Verlinden,J., Van Holm,B., Bloemen,M. and Maes,P.
EPI_ISL_l7793256	Microbiology, Immunology and Transplantation, KU Leuven	Microbiology, Immunology and Transplantation, KU Leuven	Vanmechelen,B., Wawina-Bokalanga,T., Logist,A.-5., Bloemen,M. and Maes,P.
EPI_ISL_l7793257	Microbiology, Immunology and Transplantation, KU Leuven	Microbiology, Immunology and Transplantation, KU Leuven	Wawina-Bokalanga,T., Vanmechelen,B., Logist,A.-5., Bloemen,M. and Maes,P.
EPI_ISL_l 7793258	Microbiology, Immunology and Transplantation, KU Leuven	Microbiology, Immunology and Transplantation, KU Leuven	Vanmechelen,B., Wawina-Bokalanga,T., Logist,A.-5., Bloemen,M. and Maes,P.
EPI_ISL_l7793259, EPI_ISL_l7793260, EPI_I5L_l7793261	Microbiology, Immunology and Transplantation, KU Leuven	Microbiology, Immunology and Transplantation, KU Leuven	Wawina-Bokalanga,T., Vanmechelen,B., Logist,A.-5., Bloemen,M. and Maes,P.
EPI_ISL_l7793262, EPI_ISL_l7793263, EPI_ISL_l7793264, EPI_ISL_l 7793265, EPI_ISL_l7793266, EPI_ISL_l 7793267	Microbiology, Immunology and Transplantation, KU Leuven	Microbiology, Immunology and Transplantation. KU Leuven	Vanmechelen,B., Wawina-Bokalanga,T., Logist.A.-5., Bloemen,M. and Maes,P.
EPI_ISL_l7793268	Microbiology, Immunology and Transplantation, KU Leuven	Microbiology, Immunology and Transplantation, KU Leuven	Wawina-Bokalanga,T., Vanmechelen,B., Logist,A.-5., Ysebaert,L., Verlinden,J., Sinnesael,R., Van Holm,B., Bloemen,M. and Maes,P
EPI_ISL_l7793269	Microbiology, Immunology and Transplantation, KU Leuven	Microbiology, Immunology and Transplantation, KU Leuven	Wawina-Bokalanga,T., Vanmechelen,B., Logist,A.-5., Bloemen,M. and Maes,P.

EPI_ISL_l7793270	Microbiology, Immunology and Transplantation, KU Leuven	Microbiology, Immunology and Transplantation, KU Leuven	Vanmechelen,B., Wawina-Bokalanga,T., Logist.A.-5., Bloemen,M. and Maes,P.
EPI_ISL_l 7793271	Microbiology, Immunology and Transplantation, KU Leuven	Microbiology, Immunology and Transplantation, KU Leuven	Wawina-Bokalanga,T., Vanmechelen,B., Logist.A.-5., Bloemen,M., Van Holm,B. and Maes,P.
EPI_ISL_l7793272, EPI_JSL_l7793273	Microbiology, Immunology and Transplantation, KU Leuven	Microbiology, Immunology and Transplantation, KU Leuven	Wawina-Bokalanga,T., vanmechelen,B., Logist.A.-5., Bloemen,M. and Maes,P.
EPI_ISL_l7793274	Microbiology, Immunology and Transplantation, KU Leuven	Microbiology, Immunology and Transplantation, KU Leuven	Vanmechelen,B., Wawina-Bokalanga,T., Logist,A.-5., Bloemen,M. and Maes,P.
EPI_ISL_l 7793275	Microbiology, Immunology and Transplantation, KU Leuven	Microbiology, Immunology and Transplantation, KU Leuven	Wawina-Bokalanga,T., Vanmechelen,B., Logist.A.-5., Bloemen,M. and Maes,P.
EPI_ISL_l 7793276, EPI_ISL_l 7793277	Microbiology, Immunology and Transplantation, KU Leuven	Microbiology, Immunology and Transplantation, KU Leuven	Vanmechelen,B., Wawina-Bokalanga,T., Logist.A.-5., Bloemen,M. and Maes,P.
EPI_ISL_l7793278	Microbiology, Immunology and Transplantation, KU Leuven	Microbiology, Immunology and Transplantation, KU Leuven	Wawina-Bokalanga,T., Mechelen,B., Logist.A.-5., Bloemen,M. and Maes,P.
EPI_ISL_l7793279	Microbiology, Immunology and Transplantation, KU Leuven	Microbiology, Immunology and Transplantation, KU Leuven	Wawina-Bokalanga,T., Vanmechelen,B., Logist.A.-5., Bloemen,M. and Maes,P.
EPJ_ISL_l 7793280, EPI_ISL_l 7793281	Microbiology, Immunology and Transplantation, KU Leuven	Microbiology, Immunology and Transplantation, KU Leuven	Vanmechelen,B., Wawina-Bokalanga,T., Logist,A.-5., Bloemen,M. and Maes,P.
EPI_ISL_l7793282	Microbiology, Immunology and Transplantation, KU Leuven	Microbiology, Immunology and Transplantation, KU Leuven	Vanmechelen,B., Wawina-Bokalanga,T., Logist,A.-5., Van Holm,B., Bloemen,M. and Maes,P.
EPI_ISL_l7793283	Microbiology, Immunology and Transplantation, KU Leuven	Microbiology, Immunology and Transplantation, KU Leuven	Wawina-Bokalanga,T.. Vanmechelen,B., Logist.A.-5., Bloemen,M. and Maes,P.
EPI_ISL_l7793284	Microbiology, Immunology and Transplantation, KU Leuven	Microbiology, Immunology and Transplantation, KU Leuven	Vanmechelen,B., Wawina-Bokalanga,T., Logist.A.-5., Bloemen,M. and Maes,P.
EPI_ISL_l7793285	Microbiology, Immunology and Transplantation, KU Leuven	Microbiology, Immunology and Transplantation, KU Leuven	Wawina-Bokalanga,T., Vanmechelen,B., Logist.A.-5., Bloemen,M. and Maes,P.
EPI_ISL_l7793286	Microbiology, Immunology and Transplantation, KU Leuven	Microbiology, Immunology and Transplantation, KU Leuven	Vanmechelen,B., Wawina-Bokalanga,T., Logist.A.-5., Bloemen,M. and Maes,P.
EPI_ISL_l7793287	Microbiology, Immunology and Transplantation, KU Leuven	Microbiology, Immunology and Transplantation, KU Leuven	Vanmechelen,B.. Wawona-Bokalanga,T., Logist,A.-5., Bloemen,M. and Maes,P.
EPI_ISL_l 7793288, EPI_ISL_l 7793289	Microbiology, Immunology and Transplantation, KU Leuven	Microbiology, Immunology and Transplantation, KU Leuven	Wawina-Bokalanga,T.. Vanmechelen,B., Logist.A.-5., Bloemen,M. and Maes,P.
EPJ_ISL_l7793290, EPI_JSL_l 7793291	Microbiology, Immunology and Transplantation, KU Leuven	Microbiology, Immunology and Transplantation, KU Leuven	Vanmechelen,B., Wawina-Bokalanga,T., Logist.A.-5., Bloemen,M. and Maes,P.
EPI_ISL_l 7793292, EPI_ISL_l 7793293, EPI_ISL_l7793294, EPI_ISL_l 7793295	Microbiology, Immunology and Transplantation, KU Leuven	Microbiology, Immunology and Transplantation, KU Leuven	Wawina-Bokalanga,T., Vanmechelen,B., Logist.A.-5., Bloemen,M. and Maes,P.
EPI_ISL_l7797712	Microbiology, Immunology and Transplantation, KU Leuven	Microbiology, Immunology and Transplantation, KU Leuven	Vanmechelen,B., Wawina-Bokalanga,T., Logis,A.-S., Bloemen,M., Van Holm,B. and Maes,P.
EPI_ISL_l 7797713	Microbiology, Immunology and Transplantation, KU Leuven	Microbiology, Immunology and Transplantation, KU Leuven	Vanmechelen,B., Wawina-Bokalanga,T., Logist,A.-5., Van Holm,B.. Bloemen,M. and Maes,P.
EPI_ISL_l7797714	Microbiology, Immunology and Transplantation, KU Leuven	Microbiology, Immunology and Transplantation, KU Leuven	Wawina-Bokalanga.T.. Vanmechelen,B., Logist,A.-5., Van Holm,B., Bloemen,M. and Maes,P.
EPI_ISL_l7797715	Microbiology, Immunology and Transplantation, KU Leuven	Microbiology, Immunology and Transplantation, KU Leuven	Vanmechelen,B., Wawina-Bokalanga,T., Logist.A.-5., Bloemen,M. and Maes,P.
EPI_ISL_l7797716	Microbiology, Immunology and Transplantation, KU Leuven	Microbiology, Immunology and Transplantation, KU Leuven	Wawina-Bokalanga,T., Vanmechelen,B., Logist.A.-5., Bloemen,M. and Maes,P.
EPI_ISL_l7797717	Microbiology, Immunology and Transplantation, KU Leuven	Microbiology, Immunology and Transplantation, KU Leuven	Vanmechelen,B., Wawina-Bokalanga,T., Logist.A.-5.. Van Holm,B., Bloemen,M. and Maes,P.
EPI_ISL_l7797718	Microbiology, Immunology and Transplantation, KU Leuven	Microbiology, Immunology and Transplantation, KU Leuven	Wawina-Bokalanga,T., Vanmechelen,B., Logist,A.-5., Bloemen,M. and Maes,P.
EPI_ISL_l7797719	Microbiology, Immunology and Transplantation, KU Leuven	Microbiology, Immunology and Transplantation, KU Leuven	Vanmechelen,B.. Wawina-Bokalanga,T.. Logist,A.-5., Van Holm,B.. Bloemen,M. and Maes,P.
EPJ_ISL_l 7797720, EPI_JSL_l 7797721	Microbiology, Immunology and Transplantation, KU Leuven	Microbiology, Immunology and Transplantation, KU Leuven	Wawina-Bokalanga,T.. Vanmechelen,B.. Logist.A.-5., Bloemen,M. and Maes,P.
EPI_ISL_l7797722	Microbiology, Immunology and Transplantation, KU Leuven	Microbiology, Immunology and Transplantation, KU Leuven	Vanmechelen,B., Wawina-Bokalanga,T., Logist.A.-5., Bloemen,M., Van Holm,B. and Maes,P.
EPI_ISL_l 7797723	Microbiology, Immunology and Transplantation, KU Leuven	Microbiology, Immunology and Transplantation, KU Leuven	Vanmechelen,B., Wawina-Bokalanga,T., Logist.A.-5., Bloemen,M. and Maes,P.
EPI_ISL_l7797724, EPI_ISL_l7797725	Microbiology, Immunology and Transplantation, KU Leuven	Microbiology, Immunology and Transplantation, KU Leuven	Wawina-Bokalanga,T., Vanmechelen,B., Logist,A.-5., Bloemen,M. and Maes,P.
EPI_ISL_l 7797726, EPI_ISL_l 7797727	Microbiology, Immunology and Transplantation, KU Leuven	Microbiology, Immunology and Transplantation, KU Leuven	Wawina-Bokalanga.T.. Vanmechelen,B.. Logist,A.-5., Van Holm,B.. Bloemen,M. and Maes,P.
EPI_ISL_l7797728	Microbiology, Immunology and Transplantation, KU Leuven	Microbiology, Immunology and Transplantation, KU Leuven	Wawina-Bokalanga,T., Vanmechelen,B., Logist.A.-s., Van Holm,B., Bloemen,M. and Maes,P.
EPI_ISL_l7797729, EPI_ISL_l7797730, EPI_ISL_l7797731	Microbiology, Immunology and Transplantation, KU Leuven	Microbiology, Immunology and Transplantation, KU Leuven	Wawina-Bokalanga,T., Vanmechelen,B., Logist.A.-5., Bloemen,M. and Maes,P.
EPI_ISL_l7797732	Microbiology, Immunology and Transplantation, KU Leuven	Microbiology, Immunology and Transplantation, KU Leuven	Vanmechelen,B.. Wawina-Bokalanga,T.. Logist,A.-5., Van Holm,B., Bloemen,M. and Maes,P.
EPI_ISL_l7797733	Microbiology, Immunology and Transplantation, KU Leuven	Microbiology, Immunology and Transplantation, KU Leuven	Wawina-Bokalanga.T.. Vanmechelen,B., Logist,A.-5., Van Holm,B.. Bloemen,M. and Maes,P.
EPI_ISL_l 7797734, EPI_ISL_l 7797735, EPI_ISL_l 7797736	Microbiology, Immunology and Transplantation, KU Leuven	Microbiology, Immunology and Transplantation, KU Leuven	Wawina-Bokalanga,T., Vanmechelen,B., Logist.A.-5., Bloemen,M. and Maes,P.
EPI_ISL_l7797737, EPI_JSL_l7797738	Microbiology, Immunology and Transplantation, KU Leuven	Microbiology, Immunology and Transplantation, KU Leuven	Wawina-Bokalanga,T., Vanmechelen,B., Logist,A.-5., Van Holm,B., Bloemen,M. and Maes,P.
EPI_ISL_l 7797739	Microbiology, Immunology and Transplantation, KU Leuven	Microbiology, Immunology and Transplantation, KU Leuven	Vanmechelen,B., Wawina-Bokalanga,T., Logist.A.-5., Van Holm,B., Bloemen,M. and Maes,P.
EPI_ISL_l 7797740	Microbiology, Immunology and Transplantation, KU Leuven	Microbiology, Immunology and Transplantation, KU Leuven	Vanmechelen,B., Wawina-Bokalanga.T., Logist,A.-5., Bloemen,M. and Maes,P.
EPI_ISL_l 7797741	Microbiology, Immunology and Transplantation, KU Leuven	Microbiology, Immunology and Transplantation, KU Leuven	Vanmechelen,B.. Wawina-Bokalanga,T.. Logist,A.-5.. van Holm,B., Bloemen,M. and Maes,P.
EPI_ISL_l7797742	Microbiology, Immunology and Transplantation, KU Leuven	Microbiology, Immunology and Transplantation. KU Leuven	Wawina-Bokalanga,T.. Vanmechelen,B., Logist.A.-5., Bloemen,M. and Maes,P.
EPI_ISL_l7797743	Microbiology, Immunology and Transplantation, KU Leuven	Microbiology, Immunology and Transplantation, KU Leuven	Vanmechelen,B., Wawina-Bokalanga,T., Logist.A.-5., Van Holm,B., Bloemen,M. and Maes,P.
EPI_ISL_l 7797744	Microbiology, Immunology and Transplantation, KU Leuven	Microbiology, Immunology and Transplantation, KU Leuven	Vanmechelen,B., Wawina-Bokalanga,T., Logist.A.-5., Bloemen,M., Van Holm,B. and Maes,P.
EPJ_ISL_l7797745, EPI_ISL_l7797746	Microbiology, Immunology and Transplantation, KU Leuven	Microbiology, Immunology and Transplantation, KU Leuven	Wawina-Bokalanga,T., vanmechelen,B., Logist.A.-5., Bloemen,M. and Maes,P.
EPI_ISL_l7797747	Microbiology, Immunology and Transplantation, KU Leuven	Microbiology, Immunology and Transplantation, KU Leuven	Wawina-Bokalanga,T.. Vanmechelen,B.. Logist,A.-5., van Holm,B.. Bloemen,M. and Maes,P.

EPI_ISL_I 7797748	Microbiology, Immunology and Transplantation, KU Leuven	Microbiolo9y, Immunology and Transplantation, KU Leuven	Vanmechelen,B., Wawina-Bokalanga,T., Logist,A.-5., Bloemen,M., Van Holm,B. and Maes,P.
EPI_ISL_I7797749	Microbiology, Immunology and Transplantation, KU Leuven	Microbiology, Imrnunology and Transplantation, KU Leuven	Vanmechelen,B., Wawina-Bokalanga,T., Logist,A.-S., Bloemen,M. and Maes,P.
EPI_ISL_I7797750	Microbiology, Immunology and Transplantation, KU Leuven	Microbiology, Imrnunology and Transplantation, KU Leuven	Vanmechelen,B., Wawina-Bokalanga,T., Logist,A.-S., Van Holm,B.. Bloemen,M. and Maes,P.
EPI_I5I_I7797751	Microbiology, Immunology and Transplantation, KU Leuven	Microbiology, Imrnunology and Transplantation, KU Leuven	Wawina-Bokalanga,T., Vanmechelen,B .. Logist.A.-S.. Van Holm,B., Bloemen,M. and Maes,P.
EPI_ISL_I 7797752	Microbiology, Immunology and Transplantation, KU Leuven	Microbiology, Immunology and Transplantation, KU Leuven	Vanmechelen,B .. Wawina-Bokalanga,T.. Logist,A.-S.. Van Holm,B., Bloemen,M. and Maes,P.
EPI ISL 17797753 - -	Microbiology, Immunology and Transplantation, KU Leuven	Microbiology, Immunology and Transplantation, KU Leuven	Wawina-Bokalanga,T., Vanmechelen,B., Logist,A.-5., Van Holm,B., Bloemen,M. and Maes,P.
EPI_ISL_I7797754	Microbiology, Immunology and Transplantation, KU Leuven	Microbiology, Immunology and Transplantation, KU Leuven	Wawina-Bokalanga,T., Vanmechelen,B., Logist,A.-5., Bloemen,M. and Maes,P.
EPI_ISL_I 7804477	Center for Virology, Medical University of Vienna	Center for Virology, Medical University of Vienna	Camp,J.V., Redlberger-Fritz,M. and Aberle,S.W.
EPI_ISL_I7809521	Hangzhou Center for Disease Contrai and Prevention	Hangzhou Center for Disease Contrai and Prevention	Lijiao Ao , Jun Li , Yue Yu
EPI_ISL_I 7817239, EPI_ISL_I 7817240, EPI_ISL_I 7817241	Tokyo Metropolitan Institute of Public Health	Tokyo Metropolitan Institute of Public Health	Fumi Kasuya, Wakaba Okada, Ryota Kumagai, Sachiko Harada, Arisa Amano, Michiya Hasegawa, Mami Nagashima, Kenji Sadamasu
EPI ISL 17821080, EPI ISL 17821081, EPI ISL 17821082, EPI=I5L)7821083, EPI=ISL)7821084, EPCISL)7821085, EPI_ISL_I7821086, EPI_ISL_I 7821087, EPI_ISL_I7821088	National Institute for Infectious Diseases "Matei Bals"	National Institute for Infectious Diseases "Matei Bals"	Robert Hohan, Ovidiu Vlaicu, Marius Surleac, Leontina Banica, Andreea Tudor, Simona Paraschiv
EPI_ISL_I7821096, EPI_ISL_I7821097, EPI_ISL_I7821098	ACL Laboratories	RIPHL at Rush University Medical Center	Stefan Green, Kevin Kunstman, Hannah Barbian, Felix Araujo Perez, Edith Perez, Sofiya Bobrovska, Alyse Kittner, Cecilia Chau, Giancarlo Balangue, Lok Yiu Ashley Wu
EPI_ISL_I 7821099, EPI_ISL_I7821100, EPI_I5L_I7821101	Quest Diagnostics	RIPHL at Rush University Medical Center	Stefan Green, Kevin Kunstman, Hannah Barbian. Felix Araujo Perez, Edith Perez, Sofiya Bobrovska, Alyse Kittner, Cecilia Chau, Giancarlo Balangue, Lok Yiu Ashley Wu
EPI_ISL_I7831608	Delaware Public Health Lab	Delaware Public Health Lab	Miller,H. and Bajwa,M.
EPI_ISL_I7834476	California Department of Public Health	California Department of Public Health	Kath, C., Haw, M., Espinosa, A., and Hacker, J.
EPI ISL 17837266, EPI ISL 17837267, EPI ISL 17837268, EPLISL I7959214, EPI ISL I7959215, EPI ISL I7959216	Tokyo Metropolitan Institute of Public Health	Tokyo Metropolitan Institute of Public Health	Fumi Kasuya, Wakaba Okada, Ryota Kumagai, Sachiko Harada, Arisa Amano, Michiya Hasegawa, Mami Nagashima, Kenji Sadamasu
EPI ISL 17960863, EPI ISL 17960864, EPI ISL 17960865, - - EPI ISL I7960866 - -	Laboratorio Nacional de Salud Pûblica Dr. Defill6	Laboratorio Nacional de Salud Pûblica Dr. Defill6	Isaac Miguel Sanchez, Carlos Vergara Castillo, Edwin Féliz, Anny Perla, Pedro Martinez, Yeny E. Lara Perez, Robinson Agramonte
EPI_ISL_I7972012, EPI_ISL_I7972014, EPI_ISL_I7972015	California Department of Public Health (CDPH)	California Department of Public Health (CDPH)	Kath,C., Haw,M., Espinosa,A. and Hacker,J.
EPI ISL 17972019, EPI ISL 17972020, EPI ISL 17972021 - - - - - -	Centers for Disease Contrai and Prevention DHCPP-PRB, CDC	Centers for Disease Contrai and Prevention DHCPP-PRB, CDC	Li,C.M.
EPI_ISL_I7972022	Centers for Disease Contrai and Prevention DHCPP-PRB, COC	Centers for Oisease Contrai and Prevention OHCPP-PRB, CDC	Gigante,C., Johnson,$., Zhao,H., Batra,O., Hetrick,E., Howard,D., Kovar,L., Seabolt,M., Morrison,S, Desch,M., Knipe,K., Weigand,M, Cintran,R., Burgin,A., Burraughs,M., Lee,J., Wilkins,K., McCollum,A., Hutson,C., Oavidson,W., Rao.A., Riner,D. and li.Y.
EPI_ISL_I 7972023. EPI_ISL_I 7972024, EPI_ISL_17972025. EPJ_ISL_I7972026, EPI_JSL_I 7972027	Centers for Disease Contrai and Prevention DHCPP-PRB, CDC	Centers for Oisease Contrai and Prevention OHCPP-PRB, CDC	Li,C.M.
EPI_ISL_I7972028	Centers for Disease Contrai and Prevention DHCPP-PRB, CDC	Centers for Disease Contrai and Prevention DHCPP-PRB, CDC	Gigante,C., Thomas,I., Zhao,H., Batra,D.. Hetrick,E.. Howard,☐., Kovar,I., Seabolt,M, Morrison,S., Desch,M., Knipe,K., Weigand,M, Cintron,R., Burgin,A.. Burroughs,M., Lee,J., Wilkins,K., McCollum,A., Hutson,C., Davidson,W., Rao,A., Dunn,J. and Li,Y.
EPJ_ISL_I7972029, EPI_JSL_I7972030	Centers for Oisease Contrai and Prevention OHCPP-PRB, CDC	Centers for Oisease Contrai and Prevention OHCPP-PRB, COC	Li,C.M.
EPI_ISL_I 7972031	Centers for Disease Contrai and Prevention DHCPP-PRB, CDC	Centers for Disease Contrai and Prevention DHCPP-PRB, coc	Gigante,C., Kubin,G., Zhao,H., Batra,D., Hetrick,E., Howard,☐., Kovar,I., Seabolt,M., Morrison,S., Desch,M., Knipe,K., Weigand,M, Cintron,R., Burgin,A., Burroughs,M., Lee,J., Wilkins,K., McCollum,A., Hutson,C., Davidson,W., Rao.A., White,$. and Li,Y.
EPI_ISL_I7972032	Centers for Disease Contrai and Prevention DHCPP-PRB. COC	Centers for Disease Contrai and Prevention DHCPP-PRB, COC	Li,C.M.
EPI_ISL_I7972033	Centers for Disease Contrai and Prevention OHCPP-PRB, COC	Centers for Disease Contrai and Prevention DHCPP-PRB, COC	Gigante,C., Ostadkar,R., Zhao,H., Batra,O., Hetrick,E., Howard,D., Kovar,L., Seabolt,M., Morrison,S., Oesch,M., Knipe,K., Weigand,M, Cintran,R., Burgin,A., Burraughs,M., Lee,J., Wilkins,K., McCollurn,A., Hutson,C., Davidson,W., Rao,A., Wang.X. and Li.Y.
EPI_ISL_I7972034	Centers for Disease Contrai and Prevention OHCPP-PRB, COC	Centers for Disease Contrai and Prevention DHCPP-PRB, CDC	Gigante.C., Johnson,S.. Zhao,H., Batra.D., Hetrick,E., Howard,☐..Kovar.I., Seabolt,M.. Morrison,S.. Desch,M., Knipe,K., Weigand,M., Cintran,R.. Burgin,A.. Burroughs.M., Lee,J., Wilkins,K., McCollum.A., Hutson.C., Davidson.W., Rao.A., Riner,O. and Li.Y.
EPI_ISL_I 7972035	Centers for Oisease Contrai and Prevention DHCPP-PRB, CDC	Centers for Oisease Contrai and Prevention OHCPP-PRB, coc	Gigante,C, Ostadkar,R., Zhao,H., Batra,O., Hetrick,E., Howard,☐.,Kovar,I., Seabolt.M., Morrison,S., Oesch,M., Knipe,K., Weigand,M, Cintron,R., Burgin,A., Burroughs,M., Lee,J., Wilkins,K.. McCollum,A., Hutson,C., Oavidson,W., Rao,A., Wang.X. and Li,Y.
EPI_ISL_I7972036, EPI_ISL_I7972037, EPI_ISL_I7972038	Centers for Oisease Contrai and Prevention DHCPP-PRB, CDC	Centers for Oisease Contrai and Prevention OHCPP-PRB, CDC	Li,C.M.
EPI_ISL_I 7972039	Centers for Disease Contrai and Prevention DHCPP-PRB. COC	Centers for Disease Contrai and Prevention DHCPP-PRB, COC	Gigante,C., Kubin,G., Zhao,H., Batra,D., Hetrick,E., Howard,D., Kovar,I., Seabolt,M., Morrison,S., Desch,M., Knipe,K., Weigand,M, Cintron,R., Burgin,A., Burroughs,M., Lee,J., Wilkins,K., McCollum,A., Hutson,C., Davidson,w., Rao.A., White,S. and Li,Y.
EPI_ISL_I7972040	Centers for Disease Contrai and Prevention DHCPP-PRB, CDC	Centers for Disease Contrai and Prevention DHCPP-PRB, COC	Li,C.M.
EPI_ISL_I 7977751	Centra Medico ABC	Instituto Nacional de Medicina Genomica	Cedro Tanda Alberto, Roxana Trejo Gonzalez. Laura Gomez-Romero, Alfredo Mendoza-Vargas, Dora Garnica-Lopez, Alfredo Hidalgo-Miranda.
EPI_ISL_I7979000, EPI_ISL_I7979001, EPI_ISL_I7979002	Ouest Diagnostics	Regional Innovative Public Health Laboratory at Rush University Medical Center	Stefan Green, Kevin Kunstman, Hannah Barbian, Sofiya Bobravska, Felix Araujo Perez, Edith Perez, Cecilia Chau, Giancarlo Balangue, Lok Yiu Ashley Wu, Trisha Jean, Marisol Dominguez, Latifah Boyd
EPI_ISL_I7980808	Hospital Ramon y Cajal	Hospital Ramon y Cajal	Ponce-Alonso,M., Martinez-Garcia,I., Olavarrieta,L. and Galan,J.C.
EPI ISL 17988349, EPI ISL 17988350, EPI ISL 17988351, EPI ISL 17988352, EPI ISL 17988353, EPI ISL 17988354, EPI ISL 17988355, EPI ISL 17988356, EPI ISL 17988357, EPI ISL 17988358, EPI ISL 17988359, EPI ISL 17988360, EPI ISL 17988361, EPI ISL 17988362, EPI ISL 17988363, EPI ISL 17988364, EPI ISL 17988365, EPI ISL 17988366, EPI ISL 17988367, EPI ISL 17988368, EPC1sL) 7988369. EP1=1sL) 7988370, EPÜSL)7988371. EPC1sL) 7988372. EP1=1sL) 7988373, EPC1sL) 7988374. EP1=1sL) 7988375, EPÜSL)7988376. EPC1sL) 7988377. EP1=1sL) 7988378 -			
see above	Laboratorio Central de Salud Publica	Laboratorio Central de Salud Publica	Cynthia Vazquez, Vagner Fonseca, Andrea Gomez de la Fuente, Sandra Gonzalez, Fatima Fleitas, Mauricio Lima, Natalia R. Guimaraes, Felipe C. M. Iani, Analia Rojas, Tania Alfonso, Cesar Cantera, Julio Barrios, Shirley Villalba, Maria Jose Ortega, Juan Torales, Maria Liz Gamarra, Carolina Aquino, Jaira Mendez Rico, Luiz Carlos Junior Alcantara, Marta Giovanetti
EPI_ISL_18044981. EPI_ISL_18044982, EPI_ISL_18044983, EPI_ISL_18044984, EPI_ISL_18044987	Center for Vectors and Infectious Diseases Research (CEVOI). National Health Institute Doutor Ricardo Jorge, IP (INSA),	Center for Vectors and Infectious Diseases Research (CEVOI). National Health Institute Ooutor Ricardo Jorge, IP (INSA),	Isidro.)., Borges.V., Pinto,M., Sabrai.☐.,Santos,)., Nunes,A., Mixao,V., Ferreira,R., Santos,☐..Duarte,S., Vieira,L., Borrego,M.J., Nuncio.S., Lapes de Carvalho,I.. Pelerito,A., Cordeiro.R. and Gomes,J.P.
EPI_ISL_I8055899, EPI_ISL_I8055900	Tokyo Metropolitan Institute of Public Health	Tokyo Metropolitan Institute of Public Health	Fumi Kasuya, Wakaba Okada, Ryota Kumagai, Sachiko Harada, Arisa Amano, Michiya Hasegawa, Mami Nagashima, Kenji Sadamasu
EPI_ISL_18059182, EPI_ISL_18059183, EPI_ISL_18059184	Oepartment of Acute Infectious Oiseases Contrai and Prevention, Yunnan Center for Disease Contrai and Prevention	Oepartment of Acute Infectious Oiseases Contrai and Prevention, Yunnan Center for Disease Contrai and Prevention	Meiling Zhang, Ruize Ni, Xiaoqing Fu
EPI_(SL_18064640. EPI_ISL_18064641. EPI_ISL_18064642, EPI ISL 18064643, EPI ISL 18064644, EPI ISL 18064645, - - EPI ISL 18064648 - -	Laboratory of Microbiology and Viralogy, Ospedale Amedeo di Savoia, ASL "Città di Torino"	Laboratory of Microbiology and Viralogy, Ospedale Amedeo di Savoia, ASL "Città di Torino"	Francesco Cerutti. Tiziano Allice, Maria Grazia Milia, Gabriella Gregori. Elisa Burdino, Sara Monteleone, Marisa Cazzadore, Valeria Ghisetti
EPI_ISL_I8075506, EPI_ISL_I8075507, EPI_ISL_I8075508	Tokyo Metropolitan Institute of Public Health	Tokyo Metropolitan Institute of Public Health	Fumi Kasuya, Wakaba Okada, Ryota Kumagai, Sachiko Harada, Arisa Amano, Michiya Hasegawa, Mami Nagashima, Kenji Sadamasu
EPI ISL 18076378, EPI ISL 18076379, EPI ISL 18076380, EPI=ISL=18076382, EPI=ISL=18076383, EPI=ISL=18076384, EPI ISL 18076385, EPI ISL 18076386, EPI ISL 18076388, - - EPI ISL I8076389 - -	Center for Vectors and Infectious Oiseases Research (CEVOJ). National Health Institute Doutor Ricardo Jorge, IP (INSA)	Center for Vectors and Infectious Oiseases Research (CEVDI), National Health Institute Doutor Ricardo Jorge, IP (INSA)	Isidro,)., Borges,V., Pinto,M, Sobral,O., Santos,)., Nunes,A., Mixao,V., Ferreira,R., Santos,☐.,Ouarte,S., Vieira,L., Borrego,M.J., Nuncio,S., Lapes de Carvalho,I., Pelerito,A. Cordeiro,R. and Gomes,J.P.
EPI_ISL_18097375	Tokyo Metropolitan Institute of Public Health	Tokyo Metropolitan Institute of Public Health	Fumi Kasuya, Wakaba Okada, Ryota Kumagai, Sachiko Harada, Arisa Amano, Michiya Hasegawa, Mami Nagashima, Kenji Sadamasu
EPI_ISL_18125028, EPI_ISL_18125029, EPI_ISL_18125030, EPI_(SL_18125031. EPI_ISL_18125033, EPI_ISL_18125034. EPI_ISL_18125035, EPI_ISL_18125036, EPI_(SL_18125037, EPI_ISL_18125038, EPI_ISL_18125039, EPI_ISL_18125040, EPI_ISL_18125041, EPI_ISL_18125042. EPI_ISL_18125043, EPI_ISL_18125044. EPI_ISL_18125045, EPI_ISL_18125046			
see above	University of Washington, Departrnent of Laboratory Medicine	University of Washington, Oepartment of Laboratory Medicine	Sereewit,J., Xie,H., Roychoudhury,P. and Greninger,A.l.
EPI_ISL_18128768	Bichat-Claude Bernard Hospital, Paris France	Institut Pasteur	Aurelia Kwasiborski, Véronique Hourdel, Charlotte Salière, Damien Hoinard, Quentin Grassin, Maxence Feher, Clémentine De La Porte Des Vaux, Mélanie Cresta, Jessica Vanhomwegen, Jean-Claude Manuguerra, Christophe Batéjat, Valérie Caro
EPI ISL 18131371, EPI ISL 18131372, EPI ISL 18131373, EPI ISL 18131374, EPI ISL 18131375, EPI ISL 18131376, EPI ISL 18131377, EPI ISL 18131378, EPI ISL 18131379, EPI ISL 18131380, EPI ISL 18131381, EPI ISL 18131382, EPI ISL 18131383, EPI ISL 18131384, EPI ISL 18131385, EPI ISL 18131386, EPI ISL 18131387, EPI ISL 18131388, EPI ISL 18131389, EPI ISL 18131391. EPI-ISL-18131392, EPI-ISL-18131393, EPJ-ISL-18131395, EPI-JSL-18131396, EPI-ISL-18131397, EPI-ISL-18131398, EPI-ISL-18131400, EPI-ISL-18131401, EPI-ISL-18131402, EPI-ISL-18131403, EPI-ISL-18131404, EPI-ISL-18131405, EPI-ISL-18131406, EPI-ISL-18131407, EPI-ISL-18131408, EPI-ISL-18131409, EPI-ISL-18131410, EPI-ISL-18131411, EPI-ISL-18131412, EPI-ISL-18131413, EP1=1sL)8131414. EPCtsL)813141S, EPÜSL)8131416, EPC1sL)8131417, EPC1sL)8131418, EP1=1sL)8131419. EPC1sL)8131420, EPÜSL)8131421. EPC1sL)8131422, EPC1sL)8131423, EP1=1sL)8131424. EPC1sL)8131426, EPÜSL)8131427. EPC1sL)8131428, EP1=1sL)8131429, EP1=1sL)8131430, EPC1sL)8131431. EPÜSL)8131432, EPC1sL)8131433, EPC1sL)8131434, EPI ISL 18131435, EPI ISL 18131436, EPI ISL 18131437, EPI ISL 18131438, EPI ISL 18131439, EPI ISL 18131440, EPI ISL 18131441, EPI ISL 18131442, EPI ISL 18131443, EPI ISL 18131444, EPI ISL 18131445, EPI ISL 18131446, EPI ISL 18131447, EPI ISL 18131448, EPI ISL 18131449, EPI ISL 18131450, EPI ISL 18131451, EPI ISL 18131452, EPI ISL 18131453, EPI ISL 18131454, EPC1sL)8131455, EP1=1sL)8131456, EPÜSL)8131457, EPCJsL)8131458, EPI=ISL)8131459, EPC1sL)8131460. EP1=1sL)8131461, EPÜSL)8131462, EPC1sL)8131463, EP1=1sL)8131464, EP1=1sL)8131465. EPC1sL)8131466, EPÜSL)8131467, EPC1sL)8131468, EP1=1sL)8131469, EP1=1sL)8131470. EP1=1sL)8131471, EPÜSL)8131472, EPC1sL)8131473, EP1=1sL)8131474,			

EPI_ISL_l8131475, EPI_ISL_l8131476, EPI_ISL_18131477, EPI_ISL_l8131478, EPI_ISL_18131479, EPI_ISL_l8131480, EPI_ISL_l8131481, EPI_ISL_18131482, EPI_ISL_l8131483, EPI_ISL_18131484, EPI_ISL_l8131485, EPI_ISL_l8131486, EPI_ISL_18131487, EPI_ISL_l8131488, EPI_ISL_18131489, EPI_ISL_l8131490, EPI_ISL_l8131491			
see above	Robert Koch Institute	Robert Koch Institute	Brinkmann,A., Pape,K.. Kohl.C., Schrick,L.. Michel,)., Schaade,L. and Nitsche,A.
EPI_ISL_l8137801, EPI_ISL_l8137802. EPI_ISL_l8137803	Northwestern Medicine	RIPHL at Rush University Medical Center	Stefan Green, Kevin Kunstman, Hannah Barbian, Sofiya Bobrovska, Felix Araujo Perez, Edith Perez, Cecilia Chau, Giancarlo Balangue, Lok Yiu Ashley Wu. Trisha Jean, Marisol Dominguez, Latifah Boyd, Lacy Simons
EPI_ISL_l8137804	Ouest Diagnostics	RIPHL at Rush University Medical Center	Stefan Green, Kevin Kunstman, Hannah Barbian, Sofiya Bobravska, Felix Araujo Perez, Edith Perez, Cecilia Chau, Giancarlo Balangue, Lok Yiu Ashley Wu, Trisha Jean, Marisol Dominguez, Latifah Boyd
EPI_ISL_l8137805	ACL Laboratories	RIPHL at Rush University Medical Center	Stefan Green, Kevin Kunstman, Hannah Barbian, Sofiya Bobrovska, Felix Araujo Perez, Edith Perez, Cecilia Chau, Giancarlo Balangue, Lok Yiu Ashley Wu, Trisha Jean, Marisol Dominguez, Latifah Boyd
EPI_ISL_l8137806	Ouest Diagnostics	RIPHL at Rush University Medical Center	Stefan Green, Kevin Kunstman, Hannah Barbian, Sofiya Bobrovska, Felix Araujo Perez, Edith Perez, Cecilia Chau, Giancarlo Balangue, Lok Yiu Ashley Wu, Trisha Jean, Marisol Dominguez, Latifah Boyd
EPI_ISL_l8137807, EPI_ISL_l8137808	Northwestern Medicine	RIPHL at Rush University Medical Center	Stefan Green, Kevin Kunstman, Hannah Barbian, Sofiya Bobravska, Felix Araujo Perez, Edith Perez, Cecilia Chau, Giancarlo Balangue, Lok Yiu Ashley Wu, Trisha Jean, Marisol Dominguez, Latifah Boyd, Lacy Simons
EPI_ISL_l8137809, EPI_ISL_l8137810, EPI_ISL_l8137811, EPI_ISL_l8137812, EPI_ISL_l8137813	Ouest Diagnostics	RIPHL at Rush University Medical Center	Stefan Green, Kevin Kunstman, Hannah Barbian, Sofiya Bobrovska, Felix Araujo Perez, Edith Perez, Cecilia Chau, Giancarlo Balangue, Lok Yiu Ashley Wu. Trisha Jean, Marisol Dominguez, Latifah Boyd
EPI ISL 18137814, EPI ISL 18137815, EPI ISL 18137816, EPI-ISL-18137817, EPI-ISL-18137818, EPI-ISL-18137819, - - EPI IS(_18137820 - -	Northwestern Medicine	RIPHL at Rush University Medical Center	Stefan Green, Kevin Kunstman, Hannah Barbian, Sofiya Bobrovska, Felix Araujo Perez, Edith Perez, Cecilia Chau, Giancarlo Balangue, Lok Yiu Ashley Wu, Trisha Jean, Marisol Dominguez, Latifah Boyd, Lacy Simons
EPI_ISL_l8137821, EPI_ISL_l8137822, EPI_ISL_l8137823, EPI ISL 18137824, EPI ISL 18137825, EPI ISL 18137826, EPC_ISL l8137827, EPI ISL l8137828, EPI ISL l8137829	Universidad de Chile	Universidad de Chile	Ampuera,M.M.A.
EPI_ISL_l8137830	University of Washington, Department of Laboratory Medicine	University of Washington, Department of Laboratory Medicine	Sereewit.J., Xie,H., Roychoudhury,P. and Greninger,A.L.
EPI ISL 18147334, EPI ISL 18147335, EPJ ISL 18147336, EPI JSL 18147337, EPI ISL 18147338, EPI ISL 18147339, EPI ISL 18147340, EPI ISL 18147341, EPI ISL 18147342, EPI ISL 18147343, EPI ISL 18147344, EPI ISL 18147345, EPI ISL 18147346, EPI ISL 18147347, EPI ISL 18147348, EPI ISL 18147349, EPI ISL 18147350, EPI ISL 18147351, EPI ISL 18147352, EPI ISL 18147353, EPI=ISL)8147354. EPI=ISL)8147355, EPÜSL)8147356, EPI=ISL)8147357, EPI=ISL)8147358, EPI=ISL)8147359. EPI=ISL)8147360, EPÜSL)8147361, EPI=ISL)8147362, EPI=ISL)8147363 -			
see above	Korea Disease Contrai and Prevention Agency	Korea Disease Contrai and Prevention Agency	Chung,Y.-5., Yi,H., Choi,M.-M., Kim,J.-W., Lee,M., Lee,S., Sim,G., Lee,J.H., Shin,H. and Choi,C.
EPI ISL 18147364, EPI ISL 18147365, EPJ ISL 18147366, EPI JSL 18147367, EPI ISL 18147368, EPI ISL 18147369, EPI ISL 18147370, EPI ISL 18147371, EPI ISL 18147372, EPI ISL 18147373, EPI ISL 18147374, EPI ISL 18147375, EPI ISL 18147376, EPI ISL 18147377, EPI ISL 18147378, EPI ISL 18147379, EPI ISL 18147380, EPI ISL 18147381, EPI ISL 18147382, EPI ISL 18147383, EPI-ISL-18147384, EPI-ISL-18147385, EPI-ISL-18147386, EPI-ISL-18147387, EPI-ISL-18147388, EPI-ISL-18147389, EPI-ISL-18147390, EPI-ISL-18147391, EPI-ISL-18147392, EPI-ISL-18147393, EPI-ISL-18147394, EPI-ISL-18147395, EPI-ISL-18147396, EPI-ISL-18147397, EPI-ISL-18147398, EPI-ISL-18147399, EPI-ISL-18147400, EPI-ISL-18147401, EPI-ISL-18147402, EPI-ISL-18147403, EPI=ISL)8147404, EPCISL)8147405, EPÜSL)8147406, EPCISL)8147407, EPCISL)8147408, EPI=ISL)8147409, EPCISL)8147410, EPÜSL)8147411, EPCISL)8147412, EPCISL)8147413, EPI=ISL)8147414, EPCISL)8147415, EPÜSL)8147416, EPCISL)8147417, EPCISL)8147418, EPI=ISL)8147419, EPCISL)8147420 - - - - - - - -			
see above	Centre for Biological Threats. Highly Pathogenic Viruses, Robert Koch Institute	Centre for Biological Threats, Highly Pathogenic Viruses, Robert Koch Institute	Brinkmann,A., Pape,K., Kohl,C., Schrick,L., Michel,)., Schaade,L. and Nitsche,A.
EPI_ISL_l8147421	Division of High-Consequence Pathogens & Pathology (DHCPP) Poxvirus and Rabies Branch (PRB) - Centers for Disease Contrai and Prevention (CDC)	Division of High-Consequence Pathogens & Pathology (DHCPP) - Poxvirus and Rabies Branch (PRB) - Centers for Disease Contrai and Prevention (CDC)	Gigante,C.. Kubin,G., Zhao,H.. Batra,D.. Hetrick,E., Howard,D., Kovar,L., Seabolt,M , Morrison,S., Desch,M., Knipe,K., Weigand,M.. Mcgrath,D , Takakuwa,J.. Burgin,A., Burroughs,M., Lee,J., Wilkins,K., Mccollum,A.. Hutson,C.. Davidson,w., Rao.A., White,S. and Li,Y.
EPI_ISL_l8147422	Division of High-Consequence Pathogens & Pathology (DHCPP) Poxvirus and Rabies Branch (PRB) - Centers for Disease Contrai and Prevention (CDC)	Division of High-Consequence Pathogens & Pathology (DHCPP) - Poxvirus and Rabies Branch (PRB) - Centers for Disease Contrai and Prevention (CDC)	Gigante,C., Smole.S.. Zhao,H.. Batra,D.. Hetrick,E.. Howard,D., Kovar,L., Seabolt,M , Morrison,S., Desch,M., Knipe,K.. Weigand,M.. Mcgrath,D , Takakuwa,J.. Burgin,A., Burroughs,M., Lee,J., Wilkins,K., Mccollum,A., Hutson,C.. Davidson,w., Rao.A., Brown,C. and Li.Y.
EPI_ISL_l8147423	Division of High-Consequence Pathogens & Pathology (DHCPP) Poxvirus and Rabies Branch (PRB) - Centers for Disease Contrai and Prevention (CDC)	Division of High-Consequence Pathogens & Pathology (DHCPP) - Poxvirus and Rabies Branch (PRB) - Centers for Disease Contrai and Prevention (CDC)	Gigante,C.. Ruiz.V.. Zhao,H., Batra,D.. Hetrick,E.. Howard,D., Kovar,L., Seabolt.M , Morrison.S., Desch,M., Knipe,K., Weigand,M.. Mcgrath,D. Takakuwa,J.. Burgin,A.. Burraughs,M., Lee,J.. Wilkins,K.. Mccollum,A.. Hutson,C.. Davidson,w., Rao.A., Wang,). and Li,Y.
EPI_ISL_l8147424	Division of High-Consequence Pathogens & Pathology (DHCPP) Poxvirus and Rabies Branch (PRB) - Centers for Disease Contrai and Prevention (CDC)	Division of High-Consequence Pathogens & Pathology (DHCPP) - Poxvirus and Rabies Branch (PRB) - Centers for Disease Contrai and Prevention (CDC)	Gigante,C., Johnson,S.. Zhao,H., Batra,D., Hetrick,E., Howard,D., Kovar,L., Seabolt.M , Morrison,S.. Desch,M., Knipe,K.. Weigand,M., Mcgrath,D , Takakuwa,J., Burgin,A.. Burroughs,M., Lee,J., Wilkins,K.. Mccollum,A.. Hutson,C., Davidson,W., Rao.A., Riner,D. and Li,Y.
EPI_ISL_l8147425	Division of High-Consequence Pathogens & Pathology (DHCPP) - Poxvirus and Rabies Branch (PRB) - Centers for Disease Contrai and Prevention (CDC)	Division of High-Consequence Pathogens & Pathology (DHCPP) - Poxvirus and Rabies Branch (PRB) - Centers for Disease Contrai and Prevention (CDC)	Gigante,C.. Kubin,G., Zhao.H., Batra,D.. Hetrick,E., Howard.D., Kovar,L., Seabolt.M, Morrison,S., Desch.M., Knipe.K., Weigand.M., Mcgrath,D , Takakuwa.J., Burgin,A., Burroughs,M., Lee.J., Wilkins.K., Mccollum,A.. Hutson,C., Davidson,W., Rao.A., White,$. and Li,Y
EPI_ISL_l8147426	Division of High-Consequence Pathogens & Pathology (DHCPP) - Poxvirus and Rabies Branch (PRB) - Centers for Disease Contrai and Prevention (CDC)	Division of High-Consequence Pathogens & Pathology (DHCPP) - Poxvirus and Rabies Branch (PRB) - Centers for Disease Contrai and Prevention (CDC)	Gigante,C., Hauser.)., Zhao,H., Batra,D., Hetrick,E.. Howard,D., Kovar,L., Seabolt,M , Morrison,S., Desch,M., Knipe.K.. Weigand.M.. Mcgrath,D . Takakuwa,J.. Burgin,A.. Burroughs,M., Lee.J., Wilkins.K., Mccollum,A.. Hutson,C., Davidson,W., Rao,A., Mangla,A. and Li,Y.
EPI_ISL_l8147427	Division of High-Consequence Pathogens & Pathology (DHCPP) - Poxvirus and Rabies Branch (PRB) - Centers for Disease Contrai and Prevention (CDC)	Division of High-Consequence Pathogens & Pathology (DHCPP) - Poxvirus and Rabies Branch (PRB) - Centers for Disease Contrai and Prevention (CDC)	Gigante,C., Ventura.J., Zhao,H.. Batra.D., Hetrick,E., Howard,D.. Kovar,L., Seabolt,M, Morrison,S.. Desch,M., Knipe,K., Weigand,M., Mcgrath.D. Takakuwa,J., Burgin,A., Burroughs.M.. Lee.)., Wilkins.K., Mccollum,A., Hutson,C.. Davidson,W., Rao.A., Nash,). and Li.Y.
EPI_ISL_l8147428. EPI_ISL_l8147429, EPI_ISL_l8147430. EPI_ISL_l8147431	Division of High-Consequence Pathogens & Pathology (DHCPP) - Poxvirus and Rabies Branch (PRB) - Centers for Disease Contrai and Prevention (CDC)	Division of High-Consequence Pathogens & Pathology (DHCPP) - Poxvirus and Rabies Branch (PRB) - Centers for Disease Contrai and Prevention (CDC)	Gigante,C., Ruiz.V., Zhao,H., Batra.D., Hetrick.E., Howard,D.. Kovar.L., Seabolt,M . Morrison,S.. Desch,M., Knipe,K.. Weigand,M., Mcgrath.D. Takakuwa.J., Burgin.A., Burraughs,M.. Lee,J.. Wilkins,K., Mccollum.A., Hutson,C., Davidson,W., Rao.A., Wang,). and li.Y.
EPI_ISL_l8147432	Division of High-Consequence Pathogens & Pathology (DHCPP) - Poxvirus and Rabies Branch (PRB) - Centers for Disease Contrai and Prevention (CDC)	Division of High-Consequence Pathogens & Pathology (DHCPP) - Poxvirus and Rabies Branch (PRB) - Centers for Disease Contrai and Prevention (CDC)	Gigante.C., Pettit,D., Zhao,H.. Batra.D., Hetrick,E., Howard,D.. Kovar,L., Seabolt,M, Morrison.S.. Desch,M, Knipe,K., Weigand,M., Mcgrath.D. Takakuwa.J., Burgin,A., Burroughs.M.. Lee,)., Wilkins,K., Mccollum,A., Hutson,C.. Davidson,W., Rao.A., Deutsch-Feldman,M. and li.Y.
EPI_ISL_l8147433	Division of High-Consequence Pathogens & Pathology (DHCPP) - Poxvirus and Rabies Branch (PRB) - Centers for Disease Contrai and Prevention (CDC)	Division of High-Consequence Pathogens & Pathology (DHCPP) - Poxvirus and Rabies Branch (PRB) - Centers for Disease Contrai and Prevention (CDC)	Gigante,C.. Kubin.G., Zhao.H., Batra,D.. Hetrick,E.. Howard.D., Kovar,L., Seabolt,M, Morrison,S., Desch.M. Knipe.K., Weigand,M., Mcgrath,D . Takakuwa,J., Burgin,A.. Burroughs,M., Lee.J., Wilkins,K., Mccollum,A.. Hutson.C., Davidson,W., Rao.A., White,S. and Li.Y
EPI_ISL_18147434	Division of High-Consequence Pathogens & Pathology (DHCPP) - Poxvirus and Rabies Branch (PRB) - Centers for Disease Contrai and Prevention (CDC)	Division of High-Consequence Pathogens & Pathology (DHCPP) - Poxvirus and Rabies Branch (PRB) - Centers for Disease Contrai and Prevention (CDC)	Gigante,C., Thomas,L., Zhao,H., Batra,D., Hetrick,E., Howard,D., Kovar,L., Seabolt,M , Morrison,S., Desch,M., Knipe,K., Weigand,M., Mcgrath,D. Takakuwa,J., Burgin,A., Burraughs,M., Lee.J., Wilkins,K., Mccollum,A., Hutson,C., Oavidson,W., Rao.A., Ounn,J. and li.Y.
EPI_ISL_18147435	Division of High-Consequence Pathogens & Pathology (DHCPP) - Poxvirus and Rabies Branch (PRB) - Centers for Disease Contrai and Prevention (CDC)	Division of High-Consequence Pathogens & Pathology (DHCPP) - Poxvirus and Rabies Branch (PRB) - Centers for Disease Contrai and Prevention (CDC)	Gigante,C., Ventura.J., Zhao,H., Batra,D., Hetrick,E., Howard,D., Kovar,L., Seabolt,M, Morrison,S., Oesch,M., Knipe,K., Weigand,M., Mcgrath,D., Takakuwa,J., Burgin,A., Burraughs,M., Lee,J., Wilkins,K., Mccollum,A., Hutson,C., Davidson,W., Rao.A., Nash,). and li.Y.
EPI_ISL_l8147436, EPI_ISL_18147437	Division of High-Consequence Pathogens & Pathology (DHCPP) - Poxvirus and Rabies Branch (PRB) - Centers for Disease Contrai and Prevention (CDC)	Division of High-Consequence Pathogens & Pathology (DHCPP) - Poxvirus and Rabies Branch (PRB) - Centers for Disease Contrai and Prevention (CDC)	Gigante,C., Ruiz,V., Zhao,H., Batra,D., Hetrick,E., Howard,O., Kovar,L., Seabolt,M , Morrison,S., Oesch,M., Knipe,K., Weigand,M., Mcgrath,D. Takakuwa.J., Burgin,A., Burraughs,M., Lee,J., Wilkins,K., Mccollum,A., Hutson,C., Davidson,W., Rao.A., Wang,). and li.Y.
EPI_ISL_l8147438	Division of High-Consequence Pathogens & Pathology (DHCPP) - Poxvirus and Rabies Branch (PRB) - Centers for Disease Contrai and Prevention (CDC)	Division of High-Consequence Pathogens & Pathology (DHCPP) - Poxvirus and Rabies Branch (PRB) - Centers for Disease Contrai and Prevention (CDC)	Gigante,C., Johnson,S., Zhao,H., Batra,D., Hetrick,E., Howard,O., Kovar,L., Seabolt,M , Morrison,S., Desch,M., Knipe,K., Weigand,M., Mcgrath,D , Takakuwa,J., Burgin,A., Burroughs,M., Lee,J., Wilkins,K., Mccollum,A., Hutson,C., Davidson,W.. Rao.A.. Riner,O. and Li.Y.
EPI_ISL_l8147439	Division of High-Consequence Pathogens & Pathology (DHCPP) - Poxvirus and Rabies Branch (PRB) - Centers for Disease Contrai and Prevention (CDC)	Division of High-Consequence Pathogens & Pathology (DHCPP) - Poxvirus and Rabies Branch (PRBJ - Centers for Disease Contrai and Prevention (CDC)	Gigante,C., Pettit,D., Zhao,H., Batra,O., Hetrick,E., Howard,D., Kovar,L., Seabolt,M., Morrison,S., Oesch,M., Knipe,K., Weigand,M., Mcgrath,O., Takakuwa,J, Burgin,A., Burraughs,M., Lee,J., Wilkins,K., Mccollum,A., Hutson,C.. Oavidson,W., Rao.A.. Oeutsch-Feldman,M. and Li,Y.
EPI_ISL_l8147440, EPI_ISL_l8147441	Division of High-Consequence Pathogens & Pathology (DHCPP) - Poxvirus and Rabies Branch (PRB) - Centers for Disease Contrai and Prevention (CDC)	Division of High-Consequence Pathogens & Pathology (DHCPP) - Poxvirus and Rabies Branch (PRB) - Centers for Disease Contrai and Prevention (CDC)	Gigante,C., Ruiz.V., Zhao,H., Batra,D., Hetrick,E., Howard,O., Kovar,L., Seabolt,M , Morrison,S., Oesch,M., Knipe,K., Weigand,M., Mcgrath,D. Takakuwa.J., Burgin,A., Burroughs,M., Lee,J., Wilkins,K., Mccollum,A., Hutson,C., Davidson,W., Rao,A., Wang,). and Li.Y.
EPI_ISL_l8147442	Division of High-Consequence Pathogens & Pathology (DHCPP) - Poxvirus and Rabies Branch (PRB) - Centers for Disease Contrai and Prevention (CDC)	Division of High-Consequence Pathogens & Pathology (DHCPP) - Poxvirus and Rabies Branch (PRB) - Centers for Disease Contrai and Prevention (CDC)	Gigante,C., Bradley.A., Zhao,H., Batra,D., Hetrick,E., Howard,□.,Kovar,L., Seabolt.M , Morrison,S., oesch,M., Knipe,K., Weigand,M., Mcgrath,D., Takakuwa,J, Burgin,A., Burroughs,M., Lee,J., Wilkins,K., Mccollum,A., Hutson,C.. Davidson,W., Rao,A., Anderson,). and li.Y.
EPI_ISL_l8147443	Division of High-Consequence Pathogens & Pathology (DHCPP) - Poxvirus and Rabies Branch (PRB) - Centers for Disease Contrai and	Division of High-Consequence Pathogens & Pathology (DHCPP) - Poxvirus and Rabies Branch (PRB) - Centers for Disease Contrai and Prevention (CDC)	Gigante,C., Ruiz.V., Zhao,H., Batra,D., Hetrick,E., Howard,D., Kovar,L., Seabolt,M , Morrison,S., Desch,M., Knipe,K., Weigand,M., Mcgrath,D., Takakuwa,J., Burgin,A., Burroughs,M., Lee,J., Wilkins,K., Mccollum,A., Hutson,C., Davidson,W., Rao,A., Wang,). and Li.Y.

	Prevention (CDC)		
EPI_ISL_l8147444	Division of High-Consequence Pathogens & Pathology (DHCPP) Poxvirus and Rabies Branch (PRB) - Centers for Disease Control and Prevention (CDC)	Division of High-Consequence Pathogens & Pathology (DHCPP) • Poxvirus and Rabies Branch (PRB) - Centers for Disease Contrai and Prevention (CDC)	Gigante,C., Lee,B.. Zhao,H.. Batra,D., Hetrick,E., Howard,D.. Kovar,L., Seabolt.M.. Morrison.S.. Desch,M.. Knipe,K.. Weigand,M.. Mcgrath,D. Takakuwa,J.. Burgin,A.. Burroughs,M.. Lee,J.. Wilkins,K.. Mccollum,A.. Hutson,C., Davidson,w., Rao.A., Salehi,E. and Li,Y.
EPI_ISL_l8147445	Division of High-Consequence Pathogens & Pathology (DHCPP) • Poxvirus and Rabies Branch (PRB) - Centers for Disease Control and Prevention (CDC)	Division of High-Consequence Pathogens & Pathology (DHCPP). Poxvirus and Rabies Branch (PRB) - Centers for Disease Contrai and Prevention (CDC)	Gigante,(.. Kubin,G., Zhao,H.. Batra,D.. Hetrick,E., Howard,D.. Kovar,L., Seabolt.M , Morrison,S., Desch,M.. Knipe,K.. Weigand,M.. Mcgrath,D , Takakuwa,J.. Burgin,A., Burroughs,M.. Lee,J.. Wilkins,K.. Mccollum,A.. Hutson,C.. Davidson,w., Rao.A., White,S. and li.Y.
EPI_ISL_l8147446	Division of High-Consequence Pathogens & Pathology (DHCPP) • Poxvirus and Rabies Branch (PRB) - Centers for Disease Control and Prevention (CDC)	Division of High-Consequence Pathogens & Pathology (DHCPP). Poxvirus and Rabies Branch (PRB) - Centers for Disease Contrai and Prevention (CDC)	Gigante,(.. Ruiz,V.. Zhao,H.. Batra,D.. Hetrick,E.. Howard,D.. Kovar,L., Seabolt.M.. Morrison,S.. Desch,M., Knipe,K.. Weigand,M.. Mcgrath,D., Takakuwa,J, Burgin,A.. Burroughs,M.. Lee,J.. Wilkins,K., Mccollum,A.. Hutson,C., Davidson,W.. Rao.A.. Wang,J. and Li,Y.
EPI_ISL_l8147447	Division of High-Consequence Pathogens & Pathology (DHCPP) • Poxvirus and Rabies Branch (PRB) - Centers for Disease Control and Prevention (CDC)	Division of High-Consequence Pathogens & Pathology (DHCPP). Poxvirus and Rabies Branch (PRB) - Centers for Disease Contrai and Prevention (CDC)	Gigante,C.. Xia,D.. Zhao,H., Batra,D.. Hetrick,E., Howard,D.. Kovar,L., Seabolt.M , Morrison,S.. Desch,M.. Knipe,K.. Weigand,M., Mcgrath,D. Takakuwa,J., Burgin,A.. Burroughs,M.. Lee,J.. Wilkins,K.. Mccollum,A.. Hutson,C., Davidson,W., Rao.A., Plipat,N. and Li,Y.
EPI_ISL_l8147448	Division of High-Consequence Pathogens & Pathology (DHCPP) Poxvirus and Rabies Branch (PRB) - Centers for Disease Control and Prevention (CDC)	Division of High-Consequence Pathogens & Pathology (DHCPP). Poxvirus and Rabies Branch (PRB) - Centers for Disease Control and Prevention (CDC)	Gigante,C.. Goldoft,M.. Zhao,H.. Batra,D., Hetrick,E.. Howard,☐.. Kovar,L., Seabolt.M , Morrison.S.. Desch,M., Knipe,K.. Weigand,M., Mcgrath,D. Takakuwa,J.. Burgin,A.. Burroughs,M.. Lee,J.. Wilkins,K.. Mccollum,A.. Hutson,C., Davidson,W., Rao,A., Holshue,M. and Li,Y.
EPI_ISL_l8147449	Division of High-Consequence Pathogens & Pathology (DHCPP) • Poxvirus and Rabies Branch (PRB) - Centers for Disease Control and Prevention (CDC)	Division of High-Consequence Pathogens & Pathology (DHCPP) • Poxvirus and Rabies Branch (PRB) - Centers for Disease Control and Prevention (CDC)	Gigante,C., Kubin,G., Zhao,H., Batra,D., Hetrick,E., Howard,D., Kovar,L., Seabolt,M , Morrison,S., Desch,M., Knipe,K., Weigand,M., Mcgrath,D , Takakuwa,J., Burgin,A., Burroughs,M., Lee,J., Wilkins,K., Mccollum,A.. Hutson,C., Davidson,W., Rao.A., White,S. and U,Y.
EPI_ISL_l8161269, EPI_ISL_l8161270, EPI_ISL_l8161271. EPI ISL 18161272, EPI ISL 18161273, EPI ISL 18161274, EPI=ISL)8161275, EPI=ISL)8161276, EPI=ISL)8161277, EPI_ISL_l8161279	Quest Diagnostics Nichols Institute	Los Angeles County Public Health Laboratories	J. Garrigues et al.
EPI_ISL_l8161280	UCLA Clinical Micro Lab	Los Angeles County Public Health Laboratories	J. Garrigues et al.
EPI_ISL_l8161281, EPI_ISL_l8161282, EPI_ISL_l8161284, EPI_ISL_l8161285, EPI_ISL_l8161286	Quest Diagnostics Nichols Institute	Los Angeles County Public Health Laboratories	J. Garrigues et al.
EPI_ISL_18161289, EPI_ISL_l8161290, EPI_ISL_l81612gl	Los Angeles County Public Health Laboratories	Los Angeles County Public Health Laboratories	J. Garrigues et al.
EPI_ISL_l8161292	Quest Diagnostics Nichols Institute	Los Angeles County Public Health Laboratories	J. Garrigues et al.
EPI ISL 18161293, EPI ISL 18161294, EPI ISL 18161295, - - EPI IS(.18161296 - -	Laboratory Corporation of America	Los Angeles County Public Health Laboratories	J. Garrigues et al.
EPI ISL 18161297, EPI ISL 18161298, EPI ISL 18161299, EPI-ISL-18161300, EPI-ISL-18161301, EPI-ISL-18161302, EPC_ISL 18161303, EPI ISL l8161304, EPI ISL l8161305	Quest Diagnostics Nichols Institute	Los Angeles County Public Health Laboratories	J. Garrigues et al.
EPI_ISL_l8161306	Los Angeles County Public Health Laboratories	Los Angeles County Public Health Laboratories	J. Garrigues et al.
EPI ISL 18161307, EPI ISL 18161308, EPI ISL 18161309. EPC_ISL 18161310, EPI ISL l8161312, EPI ISL 18161314	Quest Diagnostics Nichols Institute	Los Angeles County Public Health Laboratories	J. Garrigues et al.
EPI_ISL_l8161315	ARUP Laboratories	Los Angeles County Public Health Laboratories	J. Garrigues et al.
EPI ISL 18161316, EPI ISL 18161317, EPI ISL 18161318, EPI-ISL-18161319, EPI-ISL-18161320, EPI-ISL-18161321, - - EPI_ISL_1816132-2, EPI_ISL_181613f4	Laboratory Corporation of America	Los Angeles County Public Health Laboratories	J. Garrigues et al.
EPI_ISL_l8161325	Los Angeles County Public Health Laboratories	Los Angeles County Public Health Laboratories	J. Garrigues et al.
EPI_ISL_l8161326	Quest Diagnostics Nichols Institute	Los Angeles County Public Health Laboratories	J. Garrigues et al.
EPI_ISL_18168621, EPI_ISL_18168622. EPI_ISL_18168623	Quest Diagnostics	RIPHL at Rush University Medical Center	Stefan Green, Kevin Kunstman. Hannah Barbian. Sofiya Bobrovska, Felix Araujo Perez. Edith Perez, Cecilia Chau. Giancarlo Balangue. Lok Yiu Ashley Wu. Trisha Jean, Marisol Dominguez. Latifah Boyd
EPI_ISL_18168624	ACL Laboratories	RIPHL at Rush University Medical Center	Stefan Green, Kevin Kunstman, Hannah Barbian, Sofiya Bobrovska, Felix Araujo Perez, Edith Perez, Cecilia Chau, Giancarlo Balangue, Lok Yiu Ashley Wu, Trisha Jean, Marisol Dominguez, Latifah Boyd
EPI ISL 18213374, EPI ISL 18213375	Jnstitute for Hepatology,Shenzhen Third People's Hospital	Institute for Hepatology,Shenzhen Third People's Hospital	Lin Cheng,Zheng Zhang
EPI_ISL_l8228619, EPI_ISL_18228621, EPJ_ISL_18228622, EPI_ISL_18228624, EPI_ISL_l8228625, EPI_ISL_l8228626, EPI_ISL_l8228629, EPI_ISL_l8228630, EPI_ISL_18228631, EPI_ISL_18228632, EPI_ISL_18228633, EPI_ISL_18228634, EPI_ISL_l8228637, EPI_ISL_l8228638, EPI_ISL_18228640, EPI_ISL_18228641, EPI_ISL_18228643, EPI_ISL_18228645			
see above	Center for Vectors and Infectious Diseases Research (CEVDI). National Health Institute Doutor Ricardo Jorge, IP (INSA)	Center for Vectors and Infectious Diseases Research (CEVDI), National Health Institute Doutor Ricardo Jorge, IP (INSA)	Isidro,]., Borges.V., Pinto,M., Sobral,D., Santos,]., Nunes,A., Mixao,v., Ferreira,R., Santos,☐.,Duarte,S., Vieira,L., Borrego,M.J., Nuncio,S., Lapes de Carvalho,I., Pelerito,A., Cordeiro,R. and Gomes,J.P.
EPI_ISL_l8238278, EPI_ISL_1823827g, EPJ_ISL_18238280, EPI_JSL_18238281, EPI_ISL_18238282, EPI_ISL_l8238283, EPI_ISL_18238284, EPI_ISL_18238285, EPI_ISL_18238286, EPI_ISL_18238287, EPI_ISL_l8238291, EPI_ISL_18238292, EPI_ISL_18238293, EPI_ISL_l8238294, EPI_ISL_182382g5, EPI_ISL_l8238296, EPI_ISL_18238297, EPI_ISL_18238298, EPI_ISL_l823829g, EPI_ISL_18238300, EPI_ISL_l8238301			
see above	Centre for Biological Threats, Highly Pathogenic Viruses, Robert Koch Institute	Centre for Biological Threats, Highly Pathogenic Viruses, Robert Koch Institute	Brinkmann,A., Kohl,C., Pape,K., Schrick,I., Michel,]., Schaade,L. and Nitsche,A.
EPI ISL 18238302, EPI ISL 18238303, EPI ISL 18238304, EPI ISL 18238305, EPI ISL 18238306, EPI ISL 18238307, EPI ISL 18238308, EPI ISL 18238309, EPI ISL 18238310, EPI ISL 18238312, EPI ISL 18238313, EPI ISL 18238314, EPI ISL 18238315, EPI ISL 18238316, EPI ISL 18238317, EPI ISL 18238319, EPI ISL 18238320, EPI ISL 18238321, EPI ISL 18238322, EPI ISL 18238323, EPI=ISL)8238324. EPI=ISL)8238325, EPÜSL)8238326. EPI=JSL)8238327			
see above	DPH, Massachusetts State Public Health Laboratory	DPH, Massachusetts State Public Health Laboratory	Doucette,M., Gagne,L. and Smole,S.C.
EPI_ISL_18241786, EPI_ISL_18241787, EPI_ISL_18241788, EPI ISL 18241789, EPI ISL 18241790, EPI ISL 18241791, - - EPI ISL l8241792 - -	Unidade de Genômica • UFRJ	Unidade de Genômica - UFRJ	Carolina Moreira Voloch, Filipe Romero Rebella Moreira, Diana Mariani, Rafael Mello Galliez, Debora Souza Faffe, Terezinha Marta Pereira Pinto Castii'ieiras, Clarissa Damaso, Amflcar Tanuri.
EPI ISL 18245407, EPI ISL 18245408, EPI ISL 18245409, EPI=ISL)8245410, EPI=ISL)8245411, EPI=ISL)8245412, EPI_ISL_l8245413	Indian Council of Medical Research-National Institute of Virology	Indian Council of Medical Research-National Institute of Virology	Pragya Yadav, Rima Sahay, Anita Aich Shete, Sreelekshmy Mohandas
EPI_ISL_l8257122	Haidian CDC	Haidian District Center for Disease Control and Prevention Microbiological Laboratory	Fangyao Liu, Lifei Shi,Feng Liu. Heng Zhang
EPI_ISL_l8285959	UCLA Clinical Micro Lab	Los Angeles County Public Health Laboratories	J. Garrigues et al.
EPI ISL 18285960, EPI ISL 18285961, EPI ISL 18285962, EPI=ISL=18285963. EPI=ISL)8285964, EP(ISL)8285965. EPI_ISL_18285966, EPI_ISL_l8285967, EPI_ISL_18285968	Quest Diagnostics Nichols Institute	Los Angeles County Public Health Laboratories	J. Garrigues et al.
EPI_ISL_l8285969	Laboratory Corporation of America	Los Angeles County Public Health Laboratories	J. Garrigues et al.
EPI_ISL_18285970	Quest Diagnostics Nichols Institute	Los Angeles County Public Health Laboratories	J. Garrigues et al.
EPI_ISL_l8285971, EPI_ISL_l8285972	Cedars-Sinai Medical Center	Los Angeles County Public Health Laboratories	J. Garrigues et al.
EPI_ISL_l8285973, EPI_ISL_l8285974, EPI_ISL_l8285975, EPI_ISL_l8285976, EPI_ISL_l8285977, EPI_ISL_l8285978, EPI_ISL_l8285979, EPI_ISL_l8285980, EPI_ISL_l8285981, EPI_ISL_l8285982, EPI_ISL_l8285983, EPI_ISL_l8285984, EPI_ISL_l8285985, EPI_ISL_l8285986, EPI_ISL_l8285987			
see above	Quest Diagnostics	RIPHL at Rush University Medical Center	Stefan Green, Kevin Kunstman, Hannah Barbian, Sofiya Bobrovska, Felix Araujo Perez, Edith Perez, Cecilia Chau, Giancarlo Balangue, Lok Yiu Ashley Wu, Trisha Jean, Marisol Dominguez, Latifah Boyd
EPI ISL 18299473, EPI JSL 18299474	Centre for Biological Threats, Highly Pathogenic Viruses, Robert Koch Institute	Centre for Biological Threats, Highly Pathogenic Viruses, Robert Koch Institute	Brinkmann,A., Kohl,C., Schrick,L., Michel,]., Schaade,L. and Nitsche,A.
EPI ISL 18308395, EPI ISL 18308396, EPI ISL 18308397, - - EPJ_ISL_l830839-8, EPI_ISL_l83Û83f9	National Virus Reference Laboratory	National Virus Reference Laboratory	Gabriel Gonzalez, Michael Carr, Emer O'Byrne, Weronika Banka, Brian Keogan, Jonathan Dean, Daniel Hare, Cillian F De Gascun
EPI ISL 18323779, EPI ISL 18323780, EPI ISL 18323781, EPI ISL 18323782, EPI ISL 18323783, EPI ISL 18323784, EPI ISL 18323785, EPI ISL 18323786, EPI ISL 18323787, EPI ISL 18323788, EPI ISL 18323789, EPI ISL 18323790, EPI ISL 18323791, EPI ISL 18323792, EPI ISL 18323793, EPI ISL 18323794, EPI ISL 18324980, EPI ISL 18324981, EPI ISL 18324982, EPI ISL 18324983, EPI=ISL=18324984, EPI=ISL)8324985, EPÜSL)8324986, EPI=JSL=18324987, EPI=ISL)8324988, EPI=ISL=18324989, EPI=ISL)8324990, EPÜSL)8324991, EP(ISL=18324992, EPI=ISL)8324993, EPI=ISL=18324994. EP(ISL)8324995, EPÜSL)8324996, EPI=ISL=18324997, EPI=ISL)8324998, EPI=ISL=18324999. EPI=ISL)8325000, EPÜSL)8325001, EPI=ISL)8325002, EPI=ISL)8325003, EPI_ISL_l8325004, EPI_ISL_18325005, EPJ_ISL_18325006, EPI_JSL_18325007, EPI_ISL_18325008, EPI_ISL_l8325010, EPI_ISL_1832501l			
see above	California Department of Public Health	California Department of Public Health	Kath, c.. Haw, M.. Espinosa, A., and Hacker, J.
EPI_ISL_18352302, EPI_ISL_18352303, EPI_ISL_18352304, EPJ_ISL_18352305, EPI_ISL_18352306	Tokyo Metropolitan Institute of Public Health	Tokyo Metropolitan Institute of Public Health	Fumi Kasuya, Wakaba Okada. Ryota Kumagai, Sachiko Harada, Arisa Amano. Michiya Hasegawa, Mami Nagashima, Kenji Sadamasu

EPI_ISL_l8354483	Shenzhen Key Laboratory of Pathogen and Immunity	Shenzhen Key Laboratory of Pathogen and Immunity	Yang Yang, Shengjie Zhang, Yun Peng, Fuxiang Wang, Yingxia Liu, Hangzhou Lu
EPI_ISL_l8360394	Haidian District Center for Disease Contrai and Prevention Microbiological Laboratory	Haidian District Center for Disease Contrai and Prevention Microbiological Laboratory	fangyao Liu, Lifei Shi,Feng Liu, Heng Zhang
EPI_ISL_l8361186	University of Washington. Department of Laboratory Medicine	University of Washington, Department of Laboratory Medicine	Sereewit,J., Xie,H.. Roychoudhury,P. and Greninger.A.L.
EPI_ISL_l8386999, EPI_ISL_18387000, EPI_ISL_18387001. EPI_ISL_18387002, EPI_ISL_18387003, EPI_ISL_l8387004, EPI_ISL_l8387005, EPI_ISL_l8387006, EPI_ISL_l8387008, EPI_ISL_l8387009, EPI_ISL_l8387010, EPI_ISL_18387011, EPI_ISL_18387012, EPI_ISL_l8387013			
see above	NC - Los Angeles County Public Health Laboratories	NC - Los Angeles County Public Health Laboratories	Garrigues,J.M. and Green,N.M.
EPI_ISL_l8387015	Institut Pasteur de Dakar, Virology Unit	Institut Pasteur de Dakar, Virology Unit	Martin,F., Anges,Y., Benjamin,H., Amadou.S.A. and Ousmane,F.
EPI_ISL_18399134, EPI_ISL_18399135, EPI_ISL_18399136, EPI_ISL_18399137, EPI_ISL_18399138, EPI_ISL_l8399139, EPI_ISL_18399140, EPI_ISL_18399141, EPI_ISL_18399142, EPI_ISL_18399143, EPI_ISL_l8399144. EPI_ISL_18399145, EPI_ISL_18399146, EPI_ISL_l8399147			
see above	California Department of Public Health	California Department of Public Health	Kath, C., Haw, M., Espinosa, A., and Hacker, J.
EPI ISL 18414668, EPI ISL 18414669, EPI ISL 18414671, EPI IS(.18414672	Laboratory Medicine, UW Virology	Laboratory Medicine, UW Virology	Sereewit,J., Xie,H., Roychoudhury,P. and Greninger,A.L.
EPI_ISL_l8427686, EPI_ISL_l8427687, EPI_ISL_l8427688, EPI_ISL_18427689, EPI_ISL_l8427690, EPI_ISL_18427691	Ouest Diagnostics	RIPHL at Rush University Medical Center	Stefan Green, Kevin Kunstman, Hannah Barbian, Sofiya Bobravska, Felix Araujo Perez, Edith Perez, Cecilia Chau, Giancarlo Balangue, Lok Yiu Ashley Wu, Trisha Jean, Marisol Dominguez, Latifah Boyd
EPI_ISL_l8427692	ACL Laboratories	RIPHL at Rush University Medical Center	Stefan Green, Kevin Kunstman, Hannah Barbian, Sofiya Bobrovska, Felix Araujo Perez, Edith Perez, Cecilia Chau, Giancarlo Balangue, Lok Yiu Ashley Wu, Trisha Jean, Marisol Dominguez, Latifah Boyd
EPI_ISL_18436040	PKC Mampang Prapatan	National Institute of Health Research and Development	Fajar Nur Sulistyahadi, Arie Ardiansyah Nugraha, Hana Apsari Pawestri, Kartika Dewi Puspa, Herna, Subangkit. IGM Wirabrata
EPI_ISL_18436041	PKC Jatinegara	National Institute of Health Research and Development	Fajar Nur Sulistyahadi, Arie Ardiansyah Nugraha, Hana Apsari Pawestri, Kartika Dewi Puspa, Herna, Subangkit, IGM Wirabrata
EPI ISL 18443030, EPI ISL 18443031, EPI ISL 18443032, EPI ISL 18443033, EPI ISL 18443034, EPI ISL 18443035, EPI ISL 18443036, EPI ISL 18443037, EPI ISL 18443038, EPI ISL 18443039, EPI ISL 18443040, EPI ISL 18443041, EPI ISL 18443042, EPI ISL 18443043, EPI ISL 18443044, EPI ISL 18443045, EPI ISL 18452332, EPI ISL 18452334, EPI ISL 18452335, EPI ISL 18452336, EPI-ISL-18452337, EPI-ISL-18452338, EPJ-ISL-18452339, EPI-JSL-18452340, EPI-ISL-18452341, EPI-ISL-18452342, EPI-ISL-18452343, EPt-ISL-18452344, EPI-ISL-18452345, EPI-ISL-18452346, EPI-ISL-18452347, EPI-ISL-18458948, EPI-ISL-18458949, EPI-ISL-18458950, EPI-ISL-18458951, EPI-ISL-18458952, EPI-ISL-18458953, EPI-ISL-18458954, EPI-ISL-18458955, EPI-ISL-18458956, EPCISL)8458957, EPI=ISL)8458958, EPÜSL)8458959, EPCISL)8458960, EPI=ISL)8458961, EPCISL)8458962, EPI=ISL)8458963, EPÜSL)8458964, EPCISL)8458965, EPI=ISL)8460494, EPCISL)8460495, EPI=ISL)8460496, EPÜSL)8460497, EPCISL)8460498, EPI=ISL)8460499, EPCISL)8460500, EPI=ISL)8460501, EPÜSL)8460502, EPCISL)8460503, EPI=ISL)8460504, EPI_ISL_l8460505			
see above	California Department of Public Health	California Department of Public Health	Kath, C.. Haw, M., Espinosa, A., and Hacker, J.
EPI_ISL_l8463158	PKM Kembangan	National Institute of Health Research and Development	Hana Apsari Pawestri, Arie Ardiansyah Nugraha, Fajar Nur Sulistyahadi, Hartanti Dian Ikawati, Kartika Dewi Puspa, Markus Evan Anggia, Subangkit, Nelis Imaningsih, IGM Wirabrata
EPI_ISL_l8463159	PKC Setiabudi	National Institute of Health Research and Development	Hana Apsari Pawestri, Arie Ardiansyah Nugraha, Fajar Nur Sulistyahadi, Hartanti Dian Ikawati, Kartika Dewi Puspa, Markus Evan Anggia, Subangkit. Nelis Imaningsih, IGM Wirabrata
EPI_ISL_l8463160	RSUPN Dr Cipto Mangunkusumo	National Institute of Health Research and Development	Hana Apsari Pawestri, Arie Ardiansyah Nugraha, Fajar Nur Sulistyahadi, Hartanti Dian Ikawati, Kartika Dewi Puspa, Markus Evan Anggia, Subangkit, Nelis Imaningsih, IGM Wirabrata
EPI_ISL_l8463161	RSUD Kembangan	National Institute of Health Research and Development	Hana Apsari Pawestri, Arie Ardiansyah Nugraha, Fajar Nur Sulistyahadi, Hartanti Dian Ikawati, Kartika Dewi Puspa, Markus Evan Anggia, Subangkit. Nelis Imaningsih, IGM Wirabrata
EPI_ISL_l8467794	Eka Hospital BSD	National Institute of Health Research and Development	Fajar Nur Sulistyahadi, Hana Apsari Pawestri, Arie Ardiansyah Nugraha, Hartanti Dian Ikawati, Kartika Dewi Puspa, Subangkit, IGM Wirabrata
EP1_ISl_18467795, EPl_I5L_18467796	PKM Kembangan	National Institute of Health Research and Development	Hana Apsari Pawestri, Arie Ardiansyah Nugraha, fajar Nur Sulistyahadi, Hartanti Dian Ikawati, Kartika Dewi Puspa, Subangkit, IGM Wirabrata
EPI_ISL_l8467797	PKC Cengkareng	National Institute of Health Research and Development	Arie Ardiansyah Nugraha, fajar Nur Sulistyahadi, Hartanti Dian Ikawati, Kartika Dewi Puspa, Hana Apsari Pawestri, Subangkit, IGM Wirabrata
EPI_ISL_18467798	PKC Grogol Petamburan	National Institute of Health Research and Development	Fajar Nur Sulistyahadi, Hana Apsari Pawestri, Arie Ardiansyah Nugraha, Hartanti Dian Ikawati, Kartika Dewi Puspa, Subangkit, IGM Wirabrata
EPI_ISL_l8467799	PKC Setiabudi	National Institute of Health Research and Development	Hana Apsari Pawestri, Arie Ardiansyah Nugraha, Fajar Nur Sulistyahadi, Hartanti Dian Ikawati, Kartika Dewi Puspa, Subangkit, IGM Wirabrata
EPI_ISL_l8467800	PKC Pancoran	National Institute of Health Research and Development	Arie Ardiansyah Nugraha, Fajar Nur Sulistyahadi, Hartanti Dian Ikawati, Kartika Dewi Puspa, Hana Apsari Pawestri, Subangkit, IGM Wirabrata
EPI_ISL_l8467801	PKM Mampang Prapatan	National Institute of Health Research and Development	Arie Ardiansyah Nugraha, fajar Nur Sulistyahadi, Hartanti Dian Ikawati, Kartika Dewi Puspa, Hana Apsari Pawestri, Subangkit, IGM Wirabrata
EPI_ISL_18467803	RS Brawijaya Saharjo	National Institute of Health Research and Development	Fajar Nur Sulistyahadi, Hana Apsari Pawestri, Arie Ardiansyah Nugraha, Hartanti Dian Ikawati, Kartika Dewi Puspa, Subangkit, IGM Wirabrata
EPI_ISL_l8467805	PKC Kramat Jati	National Institute of Health Research and Development	Arie Ardiansyah Nugraha, Fajar Nur Sulistyahadi, Hartanti Dian Ikawati, Kartika Dewi Puspa, Hana Apsari Pawestri, Subangkit, IGM Wirabrata
EPI_ISL_l8467806	PKM Tanjung Priuk	National Institute of Health Research and Development	Fajar Nur Sulistyahadi, Hana Apsari Pawestri, Arie Ardiansyah Nugraha, Hartanti Dian Ikawati, Kartika Dewi Puspa, Subangkit, IGM Wirabrata
EPI_ISL_l8467807	PKC Kelapa Gading	National Institute of Health Research and Development	Fajar Nur Sulistyahadi, Hana Apsari Pawestri. Arie Ardiansyah Nugraha, Hartanti Dian Ikawati, Kartika Dewi Puspa, Subangkit, IGM Wirabrata
EPI_ISL_l8467808	RSUP Dr Hasan Sadikin	National Institute of Health Research and Development	Arie Ardiansyah Nugraha, fajar NurSulistyahadi, Hartanti Dian Ikawati, Kartika Dewi Puspa, Hana Apsari Pawestri, Subangkit, IGM Wirabrata
EPI ISL 18486349, EPI ISL 18486350, EPI ISL 18486351, EPI IS(.18486352	U.0. Microbiologia Laboratorio Unico Centra Servizi - Azienda Unità Sanitarie Locali della Romagna	U.0. Microbiologia Laboratorio Unico Centra Servizi - Azienda Unità Sanitarie Locali della Romagna - DIMEC - Università di Balogna	Alessandra Scagliarini, Vittorio Sambri, Maria Elena Turba, Fabio Gentilini, Francesca Taddei, Giorgio Dirani, Silvia zannoli, Giulia Gatti, Martina Brandolini, Alessandra Mistral De Pascali, Monica Cricca
EPI_ISL_l8486433, EPI_ISL_l8486434, EPJ_ISL_l8486435, EPI_JSL_18486436, EPI_ISL_l8486437, EPI_ISL_l8486438, EPI_ISL_l8486439, EPI_ISL_l8486440, EPI_ISL_18538995, EPI_ISL_l8538996, EPI_ISL_l8538997, EPI_ISL_l8538998, EPI_ISL_l8538999, EPI_ISL_l8539000, EPI_ISL_l8539001, EPI_ISL_l8539002, EPI_ISL_l8539003, EPI_ISL_l8539004, EPI_ISL_l8539005, EPI_ISL_l8539006			
see above	Centre for Biological Threats. Highly Pathogenic Viruses, Robert Koch Institute	Centre for Biological Threats, Highly Pathogenic Viruses, Robert Koch Institute	Brinkmann.A., Kohl,C.. Schrick,L., Michel.].. Schaade,L. and Nitsche.A.
EPI_ISL_l8539007, EPI_ISL_l8539008, EPJ_ISL_l8539009, EPI_JSL_18539010, EPI_ISL_l8539011, EPI_ISL_l8539012, EPI_ISL_l8539013, EPI_ISL_l8539014, EPI_ISL_18539015, EPI_ISL_l8539016, EPI_ISL_l8539017			
see above	University of Washington, Department of Laboratory Medicine	University of Washington, Department of Laboratory Medicine	Sereewit,J., Nunley,E.B., Xie,H.. Roychoudhury,P. and Greninger,A.L.
EPI_ISL_l8539018	Microbiology and Laboratory Science, Public Health Ontario	Microbiology and Laboratory Science, Public Health Ontario	Isabel.$., Eshaghi,A.. Duvvuri.V.R.. Gubbay,J.B.. Cronin,K.. Li.A.. Hasso,M., Clark.S.T.. Hopkins,J.P., Patel.S.N. and Braukmann,T.W.A.
EPI_ISL_l8553811. EPI_ISL_l8553812, EPI_ISL_l8553813. EPI_ISL_18553815, EPI_ISL_l8553816, EPI_ISL_l8553817. EPI_ISL_l8553818, EPI_ISL_l8553820. EPI_ISL_18553823, EPI_ISL_l8553824, EPI_ISL_l8553826. EPI_ISL_l8553827, EPI_ISL_l8553829. EPI_ISL_18553830, EPI_ISL_l8553831, EPI_ISL_18553832, EPI_ISL_l8553833, EPI_ISL_l8553835, EPI_ISL_18553836, EPI_ISL_l8553837, EPI_ISL_l8553838, EPI_ISL_l8553839, EPJ_ISL_l8553843, EPI_JSL_l8553844, EPI_ISL_l8553845, EPI_ISL_l8553846, EPI_ISL_l8553847, EPI_ISL_18553848, EPI_ISL_l8553849, EPI_ISL_l8553850			
see above	Center for Vectors and Infectious Diseases Research (CEVDI), National Health Institute Doutor Ricardo Jorge, IP (INSA)	Center for Vectors and tnfectious Diseases Research (CEVOI). National Health Institute Doutor Ricardo Jorge, IP (INSA)	Isidro,]., Borges.V., Pinto,M., Sabrai,☐.,Santos,]., Nunes,A., Mixao,V., Ferreira,R., Santos,☐.,Duarte,S., Vieira,L., Borrego,M.J., Nuncio,S., Lapes de Carvalho,I.. Pelerito,A., Cordeiro,R. and Gomes,J.P.
EPI_ISL_18557816, EPI_ISL_18557817, EPI_ISL_18557818	Tokyo Metrapolitan Institute of Public Health	Tokyo Metrapolitan Institute of Public Health	Fumi Kasuya, Wakaba Okada, Ryota Kumagai, Sachiko Harada, Arisa Amano, Michiya Hasegawa, Mami Nagashima, Kenji Sadamasu
EPI_ISL_18560904, EPI_ISL_18560905	Department of Pediatrics, Faculty of Medicine, Chulalongkorn University	Department of Pediatrics, Faculty of Medicine, Chulalongkorn University	Puenpa,J., Vongpunsawad,S., Intharasongkroh,D., Chaiwanichsiri,D. and Poovorawan,Y.
EPI_I5L_l8567805, EPl_I5L_18567806, EP1_15L_l8567807	Southern Nevada Public Health Laboratory	Southern Nevada Public Health Laboratory	Horng-Yuan Kan
EPI_ISL_l8627266	Division of Infectious Diseases and Tropical Medicine, University Hospital, Ludwig-Maximilians-Universitaet (LMU) Munich, Munich, Germany	Bundeswehr Institute of Microbiology	MH Antwerpen, D Lang, S Zange, R Woelfel
EPI_ISL_18634755, EPI_ISL_18634756	National Virus Reference Laboratory	National Virus Reference Laboratory	Gabriel Gonzalez, Michael Carr, Emer O'Byrne, Weronika Banka, Brian Keogan, Jose Maria Urtasun Elizari, Jonathan Dean, Daniel Hare, Cillian F De Gascun
EPI_ISL_l8642356	PKC Senen	National Institute of Health Research and Development	Hana Apsari Pawestri, Arie Ardiansyah Nugraha, Fajar Nur Sulistyahadi, Markus Evan Anggia, Subangkit, Herna, IGM Wirabrata
EPI_ISL_l8642357	PKC Cakung	National Institute of Health Research and Development	Hana Apsari Pawestri, Arie Ardiansyah Nugraha, Fajar Nur Sulistyahadi, Markus Evan Anggia, Subangkit, Herna, IGM Wirabrata
EPI_ISL_l8642358	RS Mitra Keluarga Gading	National Institute of Health Research and Development	Hana Apsari Pawestri, Arie Ardiansyah Nugraha, Fajar Nur Sulistyahadi, Markus Evan Anggia, Subangkit, Herna, IGM Wirabrata
EPI_I5L_l8642359	RSUP Persahabatan	National Institute of Health Research and Development	Hana Apsari Pawestri, Arie Ardiansyah Nugraha, Fajar Nur Sulistyahadi, Markus Evan Anggia, Subangkit. Herna, IGM Wirabrata
EPI_ISL_l8642360	PKC Cilandak	National Institute of Health Research and Development	Hana Apsari Pawestri, Arie Ardiansyah Nugraha, Fajar Nur Sulistyahadi, Markus Evan Anggia, Subangkit. Herna, IGM Wirabrata
EPI_ISL_l8642361	PKC Mampang Prapatan	National Institute of Health Research and Development	Hana Apsari Pawestri, Arie Ardiansyah Nugraha, Fajar Nur Sulistyahadi, Markus Evan Anggia, Subangkit, Herna, IGM Wirabrata
EPI_ISL_l8642362	Dinkes Kabupaten Cirebon	National Institute of Health Research and Development	Hana Apsari Pawestri, Arie Ardiansyah Nugraha, Fajar Nur Sulistyahadi, Markus Evan Anggia, Subangkit, Herna, IGM Wirabrata
EPI_ISL_l8642363	PKC Setiabudi	National Institute of Health Research and Development	Hana Apsari Pawestri, Arie Ardiansyah Nugraha, Fajar Nur Sulistyahadi, Markus Evan Anggia, Subangkit, Herna, IGM Wirabrata
EPI_ISL_l8642364	PKM Pancoran	National Institute of Health Research and Development	Hana Apsari Pawestri, Arie Ardiansyah Nugraha, Fajar Nur Sulistyahadi, Markus Evan Anggia, Subangkit. Herna, IGM Wirabrata
EPI_ISL_l8642365	Puskesmas Bambu Apus	National Institute of Health Research and Development	Hana Apsari Pawestri, Arie Ardiansyah Nugraha, Fajar Nur Sulistyahadi, Markus Evan Anggia, Subangkit. Herna, IGM Wirabrata
EPI_ISL_l8659828	Erasmus Medical Center Department of Virology	Erasmus Medical Center Department of Virology	Leonard Schuele, Bas Oude Munnink, Marjan Boter, Babette Weller, Babs Verstrepen, Richard Molenkamp, Reina Sikkema, Marion Koopmans
EPJ_ISL_18659829, EPI_JSL_18659846	Erasmus Medical Center Department of Virology	Erasmus Medical Center Department of Virology	Leonard Schuele, Marjan Soter, Hayley Cassidy, Babette Weller, Babs Verstrepen, Richard Molenkamp, Marion Koopmans, Bas Oude Munnink
EPI_I5L_l8668236, EPI_ISL_l8668237, EPI_ISL_l8668238, EPI_ISL_18668239. EPI_ISL_18668240, EPI_ISL_l8668241, EPI_ISL_18689510	University of Washington, Department of Laboratory Medicine	University of Washington, Department of Laboratory Medicine	Sereewit,J., Nunley,E.B., Xie,H., Roychoudhury,P. and Greninger,A.L.
EPI_ISL_l8689511	Bundeswehr Institute of Microbiology	Bundeswehr Institute of Microbiology	Antwerpen,M.H., Lang,☐.,Sabine,$. and Woelfel,R.
EPI_ISL_l8689512	NC - Division of High-Consequence Pathogens and Pathology (DHCPP)- Poxvirus and Rabies Branch (PRB)	NC - Division of High-Consequence Pathogens and Pathology (OHCPP)- Poxvirus and Rabies Branch (PRB)	Brien,S.C.. LeBreton,M., Doty,J.B., Mauldin,M.R.. Morgan,C.N.. Pieracci,E.G.. Ritter,J.M., Matheny,A.. Wilkins,K., Tafon,B.G., Tamoufe,U, Missoup,A.D, Nwobegahay,J., Takuo,J.M.. Nkom,F.. Mouiche,M.M., Feussom,J.M.. Wade,A. and McCollum,A.M.

EPI_ISL_l8697752	National Institute of Public Health	Institut Pasteur du Cambodge, Virology Unit	Janin Nouhin, Leakhena Pum, Jurre Y Siegers, Chin Savuth, Chau Darapheak, Veasna Duong, Erik A Karlsson
EPI ISL 18702208, EPI ISL 18702209, EPI ISL 18702212, EPI ISL 18702213, EPI ISL 18702216, EPI ISL 18702219. EPI ISL 18702220, EPI ISL 18702221, EPI ISL 18702222, EPI ISL 18702223, EPI ISL 18702224. EPI ISL 18702225, EPI ISL 18702226, EPI ISL 18702227, EPI ISL 18702228, EPI ISL 18702229. EPI ISL 18702230, EPI ISL 18702231, EPI ISL 18702234, EPI ISL 18702235, EPI=ISL)8702236, EPI=ISL)8702237, EPÜSL)8702238, EPCISL)8702239, EPI=ISL)8702240, EPI=ISL)8702241, EPI=ISL)8702243, EPÜSL)8702247, EPCISL)8702248, EPI=ISL)8702249, EPI=ISL)8702250, EPCISL)8702251, EPÜSL)8702252, EPCISL)8702253, EPI=ISL)8702254, EPI=ISL)8702255, EPCISL)8702256, EPÜSL)8702257 - - - -			
see above	Center for Vectors and Infectious Diseases Research (CEVDI). National Health Institute Doutor Ricardo Jorge, IP (INSA)	Center for Vectors and Infectious Diseases Research (CEVDI). National Health Institute Doutor Ricardo Jorge, IP (INSA)	Isidro,)., Borges.V .. Pinto,M.. Sobral,D., Santos,)., Nunes,A., Mixao,V., Ferreira,R., Santos,☐.. Duarte,S., Vieira,L, Borrego,M.. Nuncio.S.. lapes de Carvalho,I., Pelerito,A., Cordeiro,R. and Gomes,J.P.
EPI_ISL_l8719998, EPI_ISL_l8719999	CT Department of Public Health	CT Department of Public Health	Claire Pearson, Tu N. Nguyen, Kutluhan Incekara, Neranjan V. Perera
EPI_ISL_18722047	Dallas Regional Medical Center	Dallas County Health and Human Services Public Health Laboratory	Kabir, Farruk; Plaisance, Erin; Stringer, Joey: Short, Luke.
EPI_ISL_l8722048	DCHHS Sexual Health Clinic	Dallas County Health and Human Services Public Health laboratory	Kabir, Farruk; Plaisance, Erin; Stringer, Joey: Short, Luke.
EPI_ISL_18722049, EPI_ISL_18722050, EPI_ISL_l8722051, EPI_ISL_l8722052, EPI_ISL_18722053	Parkland Health and Hospital System	Dallas County Health and Human Services Public Health laboratory	Kabir, Farruk; Plaisance, Erin; Stringer, Joey; Short, Luke.
EPI_ISL_l8723946	Dallas Regional Medical Center	Dallas County Health & Human Services Public Health Laboratory	Kabir, Farruk; Plaisance, Erin; Stringer, Joey; Short, Luke.
EPI_ISL_l8723947, EPI_ISL_18723948	DCHHS Sexual Health Clinic	Dallas County Health & Human Services Public Health laboratory	Kabir, Farruk; Plaisance, Erin; Stringer, Joey; Short, Luke.
EPI_ISL_18723949	Texas Health Presbyterian at Dallas	Dallas County Health & Human Services Public Health Laboratory	Kabir, Farruk; Plaisance, Erin; Stringer, Joey: Short, Luke.
EPI_ISL_l8723950	DCHHS Sexual Health Clinic	Dallas County Health & Human Services Public Health Laboratory	Kabir, Farruk; Plaisance, Erin; Stringer, Joey: Short, Luke.
EPI_ISL_l8723952, EPI_ISL_l8723953	Parkland Health and Hospital System	Dallas County Health & Human Services Public Health laboratory	Kabir, Farruk; Plaisance, Erin; Stringer, Joey; Short, Luke.
EPI_ISL_l8723954, EPI_ISL_18723955	DCHHS Sexual Health Clinic	Dallas County Health & Human Services Public Health laboratory	Kabir, Farruk; Plaisance, Erin; Stringer. Joey; Short, Luke.
EPI_ISL_l8723956	MD Progressive Care	Dallas County Health & Human Services Public Health Laboratory	Kabir, Farruk; Plaisance, Erin; Stringer, Joey; Short, Luke.
EPI_ISL_l8723957	Collin County Health Care Services	Dallas County Health & Human Services Public Health Laboratory	Kabir, Farruk; Plaisance, Erin; Stringer, Joey; Short, Luke.
EPI_ISL_l8723958	Baylor University Medical Center Dallas	Dallas County Health & Human Services Public Health Laboratory	Kabir, Farruk; Plaisance, Erin; Stringer, Joey: Short, Luke.
EPI_ISL_l8723959, EPI_ISL_l8723960, EPI_ISL_l8723961, EPI_ISL_l8723962	Parkland Health and Hospital System	Dallas County Health & Human Services Public Health Laboratory	Kabir, Farruk; Plaisance, Erin; Stringer, Joey; Short, Luke.
EPI_ISL_l8723963	DCHHS Sexual Health Clinic	Dallas County Health & Human Services Public Health laboratory	Kabir, Farruk; Plaisance, Erin; Stringer, Joey; Short, Luke.
EPI ISL 18723965, EPI ISL 18723966, EPI ISL 18723967, - - EPI-=-ISL-=-18723968 - -	Parkland Health and Hospital System	Dallas County Health & Human Services Public Health Laboratory	Kabir, Farruk; Plaisance, Erin; Stringer, Joey; Short, Luke.
EPI_ISL_l8723969	DCHHS Sexual Health Clinic	Dallas County Health & Human Services Public Health Laboratory	Kabir, Farruk; Plaisance, Erin; Stringer, Joey; Short, Luke.
EPI_ISL_l8723970	Texas Health Presbyterian at Dallas	Dallas County Health & Human Services Public Health Laboratory	Kabir, Farruk; Plaisance, Erin; Stringer, Joey: Short, Luke.
EPI_ISL_18723971	White Rock Medical Center	Dallas County Health & Human Services Public Health Laboratory	Kabir, Farruk; Plaisance, Erin; Stringer, Joey; Short, Luke.
EPI_ISL_l8723972	Dallas Regional Medical Center	Dallas County Health & Human Services Public Health laboratory	Kabir, Farruk; Plaisance, Erin; Stringer, Joey; Short, Luke.
EPI_ISL_l8723973	MD Progressive Care	Dallas County Health & Human Services Public Health Laboratory	Kabir. Farruk; Plaisance, Erin; Stringer. Joey; Short, Luke.
EPI ISL 18723974, EPI ISL 18723975, EPI ISL 18723976, EPC_1sL-=_1a723977, EP1-=_1sL-=_1a723978, EP1-=_1sL-=_1a723980	Parkland Health and Hospital System	Dallas County Health & Human Services Public Health Laboratory	Kabir, Farruk; Plaisance, Erin; Stringer, Joey; Short, Luke.
EPI_ISL_18723981	MD Progressive Care	Dallas County Health & Human Services Public Health Laboratory	Kabir, Farruk; Plaisance, Erin; Stringer, Joey; Short, Luke.
EPI_ISL_l8723982	Dallas Regional Medical Center	Dallas County Health & Human Services Public Health Laboratory	Kabir, Farruk; Plaisance, Erin; Stringer, Joey; Short, Luke.
EPI_ISL_l8723983	Parkland Health and Hospital System	Dallas County Health & Human Services Public Health laboratory	Kabir, Farruk; Plaisance, Erin; Stringer, Joey; Short, Luke.
EPI_ISL_l8723984	Dallas County Health and Human Services Sexual Health Clinic	Dallas County Health & Human Services Public Health laboratory	Kabir, Farruk; Plaisance, Erin; Stringer, Joey; Short, Luke.
EPI_ISL_l8723985	Dallas Regional Medical Center	Dallas County Health & Human Services Public Health Laboratory	Kabir, Farruk; Plaisance, Erin; Stringer, Joey; Short, Luke.
EPJ_ISL_l8723986, EPI_JSL_18723987	Parkland Health and Hospital System	Dallas County Health & Human Services Public Health Laboratory	Kabir, Farruk; Plaisance, Erin; Stringer, Joey; Short, Luke.
EPI_ISL_l8723988	Children's Health Dallas Texas	Dallas County Health & Human Services Public Health Laboratory	Kabir, Farruk; Plaisance, Erin; Stringer, Joey: Short, Luke.
EPI_ISL_l8723989	Dallas County Health and Human Services Sexual Health Clinic	Dallas County Health & Human Services Public Health laboratory	Kabir, Farruk; Plaisance, Erin; Stringer, Joey; Short, Luke.
EPI_ISL_l8723990, EPI_ISL_18737443	Parkland Health and Hospital System	Dallas County Health & Human Services Public Health laboratory	Kabir, Farruk; Plaisance, Erin; Stringer, Joey; Short, Luke.
EPI_ISL_l8737444	Dallas Veteran Affairs Hospital	Dallas County Health & Human Services Public Health laboratory	Kabir, Farruk; Plaisance, Erin; Stringer. Joey; Short, Luke.
EPI ISL 18737445, EPI ISL 18737446, EPI ISL 18737447, EPI-ISL-18737448, EPI-ISL-18737449, EPI-ISL-18737450, - - EPI_ISL_l873745-l, EPI_ISL_187375fo	Parkland Health and Hospital System	Dallas County Health & Human Services Public Health Laboratory	Kabir, Farruk; Plaisance, Erin; Stringer, Joey; Short, Luke.
EPI_ISL_l8737522	Dallas Veteran Affairs Hospital	Dallas County Health & Human Services Public Health Laboratory	Kabir, Farruk; Plaisance, Erin; Stringer, Joey; Short, Luke.
EPI_ISL_l8737523, EPI_ISL_18737524, EPI_ISL_l8737526. EPI_ISL_18737527, EPI_ISL_18737528, EPI_ISL_l8737529. EPI_ISL_18737530, EPI_ISL_l8737544, EPI_ISL_18737545, EPI_ISL_l8737546, EPI_ISL_l8737547, EPI_ISL_18737548			
see above	Parkland Health and Hospital System	Dallas County Health & Human Services Public Health Laboratory	Kabir, Farruk; Plaisance, Erin; Stringer, Joey; Short, Luke.
EPJ_ISL_l8737549, EPI_JSL_18737550	MD Progressive Care	Dallas County Health & Human Services Public Health Laboratory	Kabir, Farruk; Plaisance, Erin; Stringer, Joey; Short, Luke.
EPI ISL 18737551, EPI ISL 18737552, EPI ISL 18737553, - - EPI_ISL_l8737554, EPI_ISL_l873755-5	Parkland Health and Hospital System	Dallas County Health & Human Services Public Health Laboratory	Kabir, Farruk; Plaisance, Erin; Stringer, Joey; Short, Luke.
EPI ISL 18737556 - -	Dallas County Health & Human Services Sexual Health Clinic	Dallas County Health & Human Services Public Health Laboratory	Kabir, Farruk; Plaisance, Erin; Stringer, Joey: Short, Luke.
EPI_ISL_l8737557	Parkland Health and Hospital System	Dallas County Health & Human Services Public Health Laboratory	Kabir, Farruk; Plaisance, Erin; Stringer, Joey: Short, Luke.
EPI_ISL_l8739594, EPI_ISL_l8739595	Tokyo Metropolitan Institute of Public Health	Tokyo Metropolitan Institute of Public Health	Fumi Kasuya, Wakaba Okada, Ryota Kumagai, Sachiko Harada, Arisa Amano, Michiya Hasegawa, Mami Nagashima, Kenji Sadamasu
EPI_ISL_18744048, EPI_ISL_l8744049, EPI_ISL_l8744050	National Medical Center	National Medical Center	Jun-sun Park, Hongsoon Yim, Jihye Um, Hyang Su Kim, BumSik Chin, Jaehyun Jeon, Yeonjae Kim, Min-Kyung Kim
EPJ_ISL_l8746884, EPI_ISL_l8746885	Erasmus Medical (enter Department of Virology	Erasmus Medical Center Department of Virology	Leonard Schuele, Marjan Soter, Babs Verstrepen, Richard Molenkamp, Marion Koopmans, Bas Oude Munnink
EPI_ISL_l8746957, EPI_ISL_18746958, EPI_ISL_l8746959, EPI_ISL_l8746960, EPI_ISL_18746961, EPI_ISL_l8746962, EPI_ISL_18746963, EPI_ISL_l8746964, EPI_ISL_l8746965, EPI_ISL_l8746966, EPI_ISL_l8746967			

see above	Parkland Health and Hospital System	Dallas County Health & Human Services Public Health Laboratory	Kabir, Farruk; Plaisance, Erin; Stringer, Joey: Short, Luke.
EPI_ISL_l8746968	DCHHS Sexual Health Clinic	Dallas County Health & Human Services Public Health Laboratory	Kabir, Farruk; Plaisance, Erin; Stringer, Joey: Short, Luke.
EPI_ISL_l8746969	Parkland Health and Hospital System	Dallas County Health & Human Services Public Health Laboratory	Kabir, Farruk; Plaisance, Erin; Stringer, Joey: Short, Luke.
EPI_ISL_l8746970	DCHHS Sexual Health Clinic	Dallas County Health & Human Services Public Health Laboratory	Kabir, Farruk; Plaisance, Erin; Stringer. Joey; Short, Luke.
EPI ISL 18746971. EPI ISL 18746972, EPI ISL 18746973, EPI ISL 18746974, EPI ISL 18746975, EPI ISL 18746976, EPI ISL 18746977, EPI ISL 18746978, EPI ISL 18746979, EPI ISL 18746980, EPI ISL 18746981. EPI ISL 18746982, EPI ISL 18746983, EPI ISL 18746984, EPI ISL 18746985, EPI ISL 18746987, EPI ISL 18746988, EPI ISL 18746989, EPI ISL 18746990, EPI ISL 18746991. EPI=ISL)8746992, EPI=ISL)8746993, EPÜSL)8746994, EPI=ISL)8746995, EPI=ISL)8746997			
see above	Parkland Health and Hospital System	Dallas County Health & Human Services Public Health Laboratory	Kabir, Farruk; Plaisance, Erin; Stringer, Joey; Short, Luke.
EPI_ISL_18746998	White Rock Medical Center	Dallas County Health & Human Services Public Health Laboratory	Kabir, Farruk; Plaisance, Erin; Stringer, Joey: Short, Luke.
EPI_ISL_l8746999	DCHHS Sexual Health Clinic	Dallas County Health & Human Services Public Health Laboratory	Kabir, Farruk; Plaisance, Erin; Stringer, Joey: Short, Luke.
EPI_ISL_l8747000. EPI_ISL_l8747001, EPI_ISL_l8747002, EPI_ISL_l8747003, EPI_ISL_l8747004, EPI_ISL_l8747005. EPI_ISL_l8747006, EPI_ISL_l8747007, EPI_ISL_l8747008, EPI_ISL_l8747009, EPI_ISL_l8747010. EPI_ISL_l8747011			
see above	Parkland Health and Hospital System	Dallas County Health & Human Services Public Health Laboratory	Kabir, Farruk; Plaisance, Erin; Stringer, Joey: Short, Luke.
EPI_ISL_l8747012	DCHHS Sexual Health Clinic	Dallas County Health & Human Services Public Health Laboratory	Kabir, Farruk; Plaisance, Erin; Stringer. Joey; Short, Luke.
EPI_ISL_l8747013. EPI_ISL_l8747014, EPI_ISL_l8747015, EP1_15L_l8747016, EPI_ISL_l8747017, EPI_ISL_l8747018. EPI_ISL_l8747019, EPI_ISL_l8747020, EPI_ISL_l8747021, EPI_ISL_l8747022, EPI_ISL_l8747023. EPI_ISL_l8747024, EPI_ISL_l8747025, EP1_15L_l8747027, EPI_ISL_l8747028			
see above	Parkland Health and Hospital System	Dallas County Health & Human Services Public Health Laboratory	Kabir, Farruk; Plaisance, Erin; Stringer, Joey; Short, Luke.
EPI ISL 18747029	DCHHS Sexual Health Clinic	Dallas County Health & Human Services Public Health Laboratory	Kabir, Farruk; Plaisance, Erin; Stringer. Joey; Short, Luke.
EPI ISL 18755970, EPI ISL 18755971. EPI ISL 18755972, EPI-ISL-18755973, EPI-ISL-18755974, EPI-ISL-18755975, EPI=ISL=l8755976, EPI=ISL)8755977, EPCISL)8755978. EPI_ISL_l8755979	Erasmus Medical Center Department of Virology	Erasmus Medical Center Department of Virology	Leonard Schuele, Marjan Soter, Babs Verstrepen, Richard Molenkamp, Marion Koopmans, Bas Oude Munnink
EPI ISL 18773078, EPI ISL 18773079, EPI ISL 18773080, EPI ISL 18773081, EPI ISL 18773082, EPI ISL 18773083, EPI ISL 18773084, EPI ISL 18773085, EPI ISL 18773086, EPI ISL 18773087, EPI ISL 18773088, EPI ISL 18773089, EPI ISL 18773090, EPI ISL 18773091, EPI ISL 18773092, EPI ISL 18773093, EPI ISL 18773094, EPI ISL 18773095, EPI ISL 18773096, EPI ISL 18773097, EPI=ISL)8773098, EPI=ISL)8773099, EPÜSL)8773100, EPI=ISL)8773101, EPI=ISL)8773102, EPI=ISL)8773103, EPI=ISL)8773104, EPÜSL)8773105, EPI=ISL)8773106, EPI=ISL)8773107, EPI=ISL)8773108			
see above	Centre for Biological Threats - Highly Pathogenic Viruses, Robert Koch Institute	Centre for Biological Threats - Highly Pathogenic Viruses, Robert Koch Institute	Brinkmann,A., Kohl,C., Schrick,l., Michel,)., Schaade,L. and Nitsche,A.
EPI ISL 18773109, EPI ISL 18773110, EPI ISL 18773111, EPI ISL 18773112, EPI ISL 18773113, EPI ISL 18773114, EPI ISL 18773115, EPI ISL 18773116, EPI ISL 18773117, EPI ISL 18773118, EPI ISL 18773119, EPI ISL 18773120, EPI ISL 18773121, EPI ISL 18773122, EPI ISL 18773123, EPI ISL 18773124, EPI ISL 18773125, EPI ISL 18773126, EPI ISL 18773127, EPI ISL 18773128, EPCISL)8773129. EPI=ISL)8773130, EPÜSL)8773131, EPCISL)8773132, EPI=ISL)8773133, EPCISL)8773134. EPI=ISL)8773135, EPÜSL)8773136, EPCISL)8773137, EPI=ISL)8773138, EPCISL)8773139. EPI=ISL)8773140, EPÜSL)8773141, EPCISL)8773142, EPI=ISL)8773143, EPCISL)8773144. EPI=ISL)8773145, EPÜSL)8773146, EPCISL)8773147, EPI=ISL)8773148, EPI_ISL_l8773149, EPI_ISL_l8773150			
see above	Animal Health - Istituto Zooprofilattico Sperimentale del Mezzogiorno	Animal Health - Istituto Zooprofilattico Sperimentale del Mezzogiorno	Viscardi,M., Cozzolino,L., Rinaldi,A., De Martinis,C., Cardillo,L., Tiberio,C., Falco,R., Guarino,V., D'Auria,G., Nappo,F., Atripaldi,L., Coppola,M.G. and Fusco,G.
EPI ISL 18781624, EPI ISL 18781626, EPI ISL 18781627, EPI JSL 18781628, EPI ISL 18781629, EPI ISL 18781630, EPI ISL 18781631. EPI ISL 18781632, EPI ISL 18781633, EPI ISL 18781634, EPI ISL 18781635, EPI ISL 18781636, EPI ISL 18781637, EPI ISL 18781786, EPI ISL 18781787, EPI ISL 18781788, EPI ISL 18781790, EPI ISL 18781791, EPI ISL 18781792, EPI ISL 18781793, EPI-ISL-18781795, EPI-ISL-18781796, EPI-ISL-18781797, EPI-ISL-18781798, EPI-ISL-18781799, EPI-ISL-18781806, EPI-ISL-18781807, EPI-ISL-18781808, EPI-ISL-18781809, EPI-ISL-18781810, EPI-ISL-18781811, EPI-ISL-18781812, EPI-ISL-18781813, EPI-ISL-18781815, EPI-ISL-18781816, EPI-ISL-18781817, EPI-ISL-18781818, EPI-ISL-18781819, EPI-ISL-18781820, EPI-ISL-18781833, EPI-ISL-18781834. EPI-ISL-18781835, EPI-ISL-18781836, EPI-ISL-18781837, EPI-ISL-18781839, EPI-ISL-18781840. EPI-ISL-18781841, EPI-ISL-18781842, EPI-ISL-18781843, EPI-ISL-18781844, EPI-ISL-18781845. EPI-ISL-18781846, EPI-ISL-18781847, EPI-ISL-18781848, EPI-ISL-18781849, EPI-ISL-18781913. EPI-ISL-18781915, EPI-ISL-18781916. EPI-ISL-18781917, EPI-ISL-18781918, EPI-ISL-18781919, EPI-ISL-18781920, EPI-ISL-18781921, EPI-ISL-18781922, EPI-ISL-18781923, EPI-ISL-18781924, EPI-ISL-18781925, EPI-ISL-18781926, EPI-ISL-18781928, EPI-ISL-18781929, EPI-ISL-18786340, EPI-ISL-18786341, EPI-ISL-18786342, EPI-ISL-18786343, EPI-ISL-18786346, EPI-ISL-18786347, EPI-ISL-18786348, EPI-ISL-18786349, EPI-ISL-18786350, EPI-ISL-18786351, EPI=ISL)8786352, EPI=ISL)8786354, EPÜSL)8786356, EPI=ISL)8786357, EPI=ISL)8786358, EPI=ISL)8786359			
see above	California Department of Public Health	California Department of Public Health	Kath, C., Haw, M., Espinosa, A., and Hacker, J.
EPI_ISL_l8798834	PKM Mampang Prapatan	National Institute of Health Research and Development	Hana Apsari Pawestri, Arie Ardiansyah Nugraha, Fajar Nur Sulistyahadi, Markus Evan Anggia, Subangkit
EPI_ISL_l8798835	PKC Kebayoran Lama	National Institute of Health Research and Development	Hana Apsari Pawestri, Arie Ardiansyah Nugraha, Fajar Nur Sulistyahadi, Markus Evan Anggia, Subangkit
EPI_ISL_l8798836	RS Grha Kedoya Jakarta	National Institute of Health Research and Development	Hana Apsari Pawestri, Arie Ardiansyah Nugraha, Fajar Nur Sulistyahadi, Markus Evan Anggia, Subangkit
EPI_ISL_l8798837	PKC Kebayoran Saru	National Institute of Health Research and Development	Hana Apsari Pawestri, Arie Ardiansyah Nugraha, Fajar Nur Sulistyahadi, Markus Evan Anggia, Subangkit
EPI_ISL_l8798838	PKC Tanah Abang	National Institute of Health Research and Development	Hana Apsari Pawestri, Arie Ardiansyah Nugraha, Fajar Nur Sulistyahadi, Markus Evan Anggia, Subangkit
EPI_ISL_l8798839	PKM Bogor Timur	National Institute of Health Research and Development	Hana Apsari Pawestri, Arie Ardiansyah Nugraha, Fajar Nur Sulistyahadi, Markus Evan Anggia, Subangkit
EPI_ISL_l8798840	PKM Warung Jambu	National Institute of Health Research and Development	Hana Apsari Pawestri, Arie Ardiansyah Nugraha, Fajar Nur Sulistyahadi, Markus Evan Anggia, Subangkit
EPI_ISL_l8798841	PKC Pademangan	National Institute of Health Research and Development	Hana Apsari Pawestri, Arie Ardiansyah Nugraha, Fajar Nur Sulistyahadi. Markus Evan Anggia, Subangkit
EPI_ISL_l8798842	PKC Kebayoran Saru	National Institute of Health Research and Development	Hana Apsari Pawestri, Arie Ardiansyah Nugraha, Fajar Nur Sulistyahadi, Markus Evan Anggia, Subangkit
EPI_ISL_l8809376	California Department of Public Health	California Department of Public Health	Kath, C., Haw, M., Espinosa, A., and Hacker, J.
EPI_ISL_l8822108, EPI_ISL_l8822109	Ouest Diagnostics Nichols Institute	Los Angeles County Public Health Laboratories	S. McCann et al
EPI ISL 18846272, EPI ISL 18846273, EPI ISL 18846274, EPI-ISL-18846275, EPI-ISL-18846276, EPI-ISL-18846277, EPC,ISL-=.18846278	Centre for Biological Threats, Highly Pathogenic Viruses, Robert Koch Institute	Centre for Biological Threats, Highly Pathogenic Viruses, Robert Koch Institute	Brinkmann,A., Kohl,C., Schrick,L., Michel,)., Schaade,L. and Nitsche,A.
EPI_ISL_18846279, EPI_ISL_18846280, EPI_ISL_18846284, EPI_ISL_18846285, EPI_ISL_l8846287, EPI_ISL_18846288, EPI_ISL_18846289, EPI_ISL_18846290, EPI_ISL_18846291, EPI_ISL_18846292, EPI_ISL_18846293, EPI_ISL_18846294, EPI_ISL_18846295, EPI_ISL_18846296, EPI_ISL_18846297, EPI_ISL_18846298, EPI_ISL_18846299, EPI_ISL_18846301, EPI_ISL_18846302, EPI_ISL_l8846303, EPI_ISL_l8846304. EPI_ISL_18846305, EPJ_ISL_18846306. EPI_ISL_l8846307, EPI_ISL_18846309, EPI_ISL_l8846310. EPI_ISL_18846311, EPI_ISL_18846312. EPI_ISL_l8846313, EPI_ISL_18846314, EPI_ISL_l8846315. EPI_ISL_18846316, EPI_ISL_18846318. EPI_ISL_l8846319			
see above	Animal Health, Istituto Zooprofilattico Sperimentale del Mezzogiorno	Hanimal Health, Istituto Zooprofilattico Sperimentale del Mezzogiorno	Viscardi,M., Cozzolino,L., Rinaldi,A., De Martinis,C., Cardillo,L., Tiberio,C., Falco,R., Guarino,V., D'Auria,G., Nappo,F. Atripaldi,L., Coppola,M.G. and Fusco,G.
EPI_ISL_18846321, EPI_ISL_l8846322. EPI_ISL_18846323	University of Washington. Department of Laboratory Medicine	University of Washington, Department of Laboratory Medicine	Sereewit,J., Nunley.E.B., Xie,H., Roychoudhury,P. and Greninger,A.L.
EPI_lSL_18857033, EPI_ISL_l8857034	Pathogen Genomics Lab, National Institute for Biomedical Research (INRB)	Pathogen Genomics Lab, National Institute for Biomedical Research (INRBJ	Placide Mbala Kingebeni, Gradi Luakanda-Ndelemo, Steven Lakin, Eddy Kinganda-Lusamaki, Amuri Aziza, Francisca Muyembe, Thierry Kalonji, Emile Malembi, Jean Paul Matela, Nicolas Fernandez Nuríez, Toutou Ukafi, Emmanuel Hasivirwe Vakaniaki, Emmanuel Lokilo Lofiko, Jean Claude Makangara, Nicole A. Hoff, Joelle Kabamba, Tine Cooreman, Béatrice Nguete, MD, Danae Witte, Ahidjo Ayouba, Stijn Roge, Martine Peeters, Sydney Merritt,Elisabeth Pukuta, Joachim Mariën. Eugene Bangwen, Charles Lewis, Jeffrey B. Doty, Laurens Liesenborghs, Didine Kaba, Andrea McCollum, Lisa E. Hensley. Robert Shongo, Steve Ahuka-Mundeke, Eric Delaporte, Anne W. Rimoin, Jean Jacques Muyembe-Tamfum
EPI_ISL_l8879931	Central Public Health Laboratories, Ministry of Health Egypt	Center of Scientific Excellence for Influenza Viruses, National Research Centre (NRC), Egypt.	Wael H. Roshdy, Rabeh El-Shesheny, Yassmin Moatasim, Mina N. Kamel, Shaymaa Shawky, Mokhtar Gomaa, Galal Mahmoud, Amer Sayed, Amel Naguib, Nancy El Guindy, Ahmed Kandeil, Mohamed A. Ali, Amr Kandeel
EPI_ISL_18886301	Reseau lab Bukavu-Kamituga DPS/ Sud -Kivu	Reseau lab Bukavu-Kamituga DPS/ Sud-Kivu	Leandre Murhula Masirika, Jean Claude Udahemuka, Leonard Schuele, Pacifique Ndishimye, Saria Otani ,Justin Bengehya Mbiribindi, Jean M. Marekani, Léandre Mutimbwa Mambo, Marjan Soter, David F. Nieuwenhuijse,Ernest Balyahamwabo Kalalizi,Trudie Lang, Jean Pierre Musabyimana, Frank M. Aarestrup, Marion Koopmans, Bas B. Oude Munnink. Freddy Belesi Siangoli
EPI_ISL_l8886639	Reseau Lab Bukavu-Kamituga DPS/ SUD-Kivu	Reseau Lab Bukavu-Kamituga DPS/ Sud-Kivu	Leandre Murhula Masirika, Jean Claude Udahemuka, Leonard Schuele, Pacifique Ndishimye, Saria Otani ,Justin Bengehya Mbiribindi, Jean M. Marekani, Léandre Mutimbwa Mambo, Marjan Boter, David F. Nieuwenhuijse,Ernest Balyahamwabo Kalalizi,Trudie Lang, Jean Pierre Musabyimana. Frank M. Aarestrup, Marion Koopmans. Bas B. Oude Munnink. Freddy Belesi Siangoli
EPI_ISL_l8899228	OUEST DIAGNOSTICS NICHOLS INSTITUTE	Los Angeles County Public Health Laboratories	S. McCann et. al.
EPI_ISL_l8899231	LOS ANGELES COUNTY PUBLIC HEALTH LABORATORY	Los Angeles County Public Health Laboratories	S. McCann et. al.
EPI_ISL_l8899234, EPI_ISL_18899235	LABCORP	Los Angeles County Public Health Laboratories	S. McCann et. al.
EPI_ISL_l8899237, EPI_ISL_18899239	LOS ANGELES COUNTY PUBLIC HEALTH LABORATORY	Los Angeles County Public Health Laboratories	S. McCann et. al.
EPI_ISL_l8899240, EPI_ISL_l8899241, EPI_ISL_l8899242	OUEST DIAGNOSTICS WEST HILLS	Los Angeles County Public Health Laboratories	S. McCann et. al.
EPI_ISL_l8899244	ARUP LABORATORIES	Los Angeles County Public Health Laboratories	S. McCann et. al.
EPI_ISL_18952716, EPI_ISL_l8952717	Ouest Diagnostics Nichols Institute	Los Angeles County Public Health Laboratory	S. McCann et. al.
EPI_ISL_l8952718	Los Angeles County Public Health Laboratory	Los Angeles County Public Health Laboratory	S. McCann et. al.
EPI_ISL_18952719	Laboratory Corporation of America	Los Angeles County Public Health Laboratory	S. McCann et. al.
EPI_ISL_l8959309, EPI_ISL_l8959310, EPJ_ISL_l8959311, EPI_JSL_l8959312, EPI_ISL_l8959313, EPI_ISL_l8959314, EPI_ISL_l8959315, EPI_ISL_l8959316, EPI_ISL_l8959317, EPI_ISL_l8959318, EPI_ISL_l8959319, EPI_ISL_l8959320, EPI_ISL_l8959321, EPI_ISL_l8959322, EPI_ISL_l8959323, EPI_ISL_l8959324			
see above	California Department of Public Health	California Department of Public Health	Kath, C., Haw, M., Espinosa, A., and Hacker, J.

EPI_ISL_18971016, EPI_ISL_l8971017	Central Public Health Laboratory, State Health Surveillance Center of the Rio Grande do Sul State Health Department (LACEN/CEVS/SES-RS)	Center for Scientific and Technological Development, State Center for Health Surveillance of the Secretary of Health of the State of Rio Grande do Sul (CDCT/CEVS/SES-RS)	Fernanda Godinho, Richard Steiner Salvato
EPI_ISL_l8993169, EPI_ISL_18993171, EPI_ISL_18993173	HCMC Hospital of Dermato Venereology	STls Lab, Pasteur Institute in Ho Chi Minh City	Yen Nhi Nguyen, Tam-Duong Le-Ha, Lien Le, Hanh Lan Nguyen Thi, Thang Minh Cao, Thinh Viet Nguyen, Quang Duy Pham, Quang Luang Chan, Thuong Vu Nguyen, Trung Vu Nguyen
EPI_ISL_18993174	Lam Dong 2 Hospital	STls Lab, Pasteur Institute in Ho Chi Minh City	Yen Nhi Nguyen, Tam-Duong le-Ha, lien Le, Hanh Lan Nguyen Thi, Thang Minh Cao, Thinh Viet Nguyen, Quang Duy Pham, Quang Luang Chan, Thuong Vu Nguyen, Trung Vu Nguyen
EPI_ISL_18993183	Quest Diagnostics Nichols Institute	Los Angeles County Public Health Laboratories	J. Garrigues et. al.
EPI_ISL_l899318S	Ucla Healthcare Clinical Laboratory - Brentwood	Los Angeles County Public Health Laboratories	J. Garrigues et. al.
EPI ISL 18993186, EPI ISL 18993187, EPI ISL 18993188, - - EPI_ISL_1899318-9, EPI_ISL_1899319-1	Ouest Diagnostics Nichais Institute	Los Angeles County Public Health Laboratories	J. Garrigues et. al.
EPI_ISL_l8993192, EPI_ISL_l8993194, EPI_ISL_l8993195	Laboratory Corporation Of America	Los Angeles County Public Health Laboratories	J. Garrigues et. al.
EPI_ISL_18993197, EPI_ISL_18993198	Los Angeles County Public Health Laboratory	Los Angeles County Public Health Laboratories	J. Garrigues et. al.
EPI_ISL_l8993199, EPI_ISL_18993200	Quest Diagnostics Nichais Institute	Los Angeles County Public Health laboratories	J. Garrigues et. al.
EPI_ISL_l8993201	Los Angeles County Public Health Laboratory	Los Angeles County Public Health Laboratories	J. Garrigues et. al.
EPI_ISL_l8993203	Quest Diagnostics Nichais Institute	los Angeles County Public Health laboratories	J. Garrigues et. al.
EPI_ISL_l8993204	Laboratory Corporation Of America	Los Angeles County Public Health Laboratories	J. Garrigues et. al.
EPI_ISL_18993205	Quest Diagnostics Nichais Institute	Los Angeles County Public Health Laboratories	J. Garrigues et. al.
EPI_ISL_l8993206	Quest Diagnostics West Hills	Los Angeles County Public Health laboratories	J. Garrigues et. al.
EPI_ISL_l8993207	Laboratory Corporation Of America	Los Angeles County Public Health Laboratories	J. Garrigues et. al.
EPI_ISL_18993208, EPI_ISL_l8993209	Quest Diagnostics Nichais Institute	Los Angeles County Public Health Laboratories	J. Garrigues et. al.
EPI_ISL_18993210	Arup Laboratories	Los Angeles County Public Health Laboratories	J. Garrigues et. al.
EPI_ISL_18993211, EPI_ISL_l8993212, EPI_ISL_18993213	Laboratory Corporation Of America	Los Angeles County Public Health Laboratories	J. Garrigues et. al.
EPI_ISL_l8993214	Quest Diagnostics Nichais Institute	Los Angeles County Public Health laboratories	J. Garrigues et. al.
EPI_ISL_l8993954. EPI_ISL_189939S5, EPI_ISL_18993956, EPI_ISL_18993958, EPI_ISL_18993959, EPI_ISL_l8993960. EPI_ISL_18993961, EPI_ISL_18993962, EPI_ISL_18993963, EPI_ISL_18993964, EPI_ISL_l8993965. EPI_ISL_18993966, EPI_ISL_18993967, EPI_ISL_l8993968, EPI_ISL_18993969, EPI_ISL_l8993970			
see above	Center for Vectors and Infectious Diseases Research (CEVDI). National Health Institute Doutor Ricardo Jorge, IP (INSA)	Center for Vectors and Infectious Diseases Research (CEVDI), National Health Institute Doutor Ricardo Jorge, IP (INSA)	Isidro,J., Borges,V., Pinto,M, Sobral,D., Santos,J., Nunes,A., Mixao,V., Ferreira,R., Santos,☐.,Ouarte,S., Vieira,L., Borrego,M.J., Nuncio,S., Lapes de Carvalho,I., Pelerito,A., Cordeiro,R. and Gomes,J.P.
EPI_ISL_l9001887	Tokyo Metropolitan Institute of Public Health	Tokyo Metropolitan Institute of Public Health	Fumi Kasuya, Wakaba Okada, Ryota Kumagai, Sachiko Harada, Arisa Amano, Michiya Hasegawa, Mami Nagashima, Kenji Sadamasu
EPI_ISL_l9002300	Erasmus Medical Center Department of Virology	Erasmus Medical Center Department of Virology	Leonard Schuele, Marjan Boter, Babs Verstrepen, Richard Molenkamp, Marion Koopmans, Bas Oude Munnink
EPI_ISL_l9004044	Centre de Recherche en Sciences Naturelles de Lwiro	Centre de Recherche en Sciences Naturelles de Iwiro	leandre Murhula Masirika, Jean Claude Udahemuka, Pacifique Ndishimye, Gustavo Sganzerla Martinez, Patricia Kelvin, Maliyamungu Bubala Nadine, Bilembo Kitwanda Steeven, Franklin Kumbana Mweshi, Léandre Mutimbwa Mambo, Bas B. Oude Munnink, Justin Bengehya Mbiribindi, Freddy Belesi Siangoli, Trudie Lang, Jean M. Malekani, Frank M. Aarestrup, Marion Koopmans, Leonard Schuele, Jean Pierre Musabyimana, Brigitte Umutoni, Ali Toloue, Benjamin Hewins, Mansi Dutt, Anuj Kumar, Alyson A. Kelvin, Jean-Paul Kabemba lukusa, Christian Gortazar, David J Kelvin, Luis Flores
EPI_ISL_19004045, EPI_ISL_19004046	Centre de Recherche en Sciences Naturelles de Iwiro	Centre de Recherche en Sciences Naturelles de Lwiro	Leandre Murhula Masirika , Jean Claude Udahemuka, Pacifique Ndishimye, Gustavo Sganzerla Martinez, Patricia Kelvin, Maliyamungu Bubala Nadine, Bilembo Kitwanda Steeven, Franklin Kumbana Mweshi, Léandre Mutimbwa Mambo, Bas B. Oude Munnink, Justin Bengehya Mbiribindi, Freddy Belesi Siangoli, Trudie Lang, Jean M. Malekani, Frank M. Aarestrup, Marion Koopmans, Leonard Schuele, Jean Pierre Musabyimana. Brigitte Umutoni, Ali Toloue, Benjamin Hewins, Mansi Dutt, Anuj Kumar, Alyson A. Kelvin, Jean-Paul Kabemba Lukusa, Christian Gortazar, David J Kelvin, Luis Flores
EPI_ISL_19012435, EPI_ISL_19012436	Laboratorio de Enterovirus, Instituto Oswaldo Cruz, Fiocruz	Oswaldo Cruz Foundation Laboratory of Respiratory Virus and Measles	Paola Resende, Elisa Cavalcante Pereira, Bruna Mendonça da Silva, Jéssica Graça Macedo de Carvalho, Larissa Macedo Pinto, Victor Guimaraes, Marilda Siqueira, Renan da Silva Faustino, Marilia Santini, Edson Elias da Silva on behalf of the Fiocruz Genomic Surveillance Network
EPI ISL 19016746, EPI ISL 19016747, EPI ISL 19016748, EPI ISL 19016749, EPI ISL 19016763, EPI ISL 19016764, EPI ISL 19016765, EPI ISL 19016766, EPI ISL 19016767, EPI ISL 19016768, EPI ISL 19016769, EPI ISL 19016772, EPI ISL 19016773, EPI ISL 19016774, EPI ISL 19016775, EPI ISL 19016776, EPI ISL 19016778, EPI ISL 19016779, EPI ISL 19016780, EPI ISL 19016781. EPI=ISL)9016782, EPI=ISL)9016783, EPÜSL)901678S, EPI=ISL)9016786, EPI=ISL)9016787			
see above	Ouest Diagnostics	RIPHL at Rush University Medical (enter	Stefan Green, Kevin Kunstman, Hannah Barbian, Sofiya Bobrovska, Felix Araujo Perez, Erin Newcomer
EPI ISL 19016818, EPI ISL 19016819	Ouest Diagnostics	Regional Innovative Public Health Laboratory (RIPHL) at Rush University Medical Center	Stefan Green, Kevin Kunstman, Hannah Barbian, Sofiya Bobrovska, Felix Araujo Perez, Erin Newcomer
EPI_ISL_l9022858, EPI_ISL_l9022859, EPI_ISL_19022860, EPI_ISL_19022861, EPI_ISL_19022862	Osaka Metropolitan University, Graduate School of Medicine, Oepartment of Virology and Parasitology	Osaka Metropolitan University, Graduate School of Medicine, Department of Virology and Parasitology	Evariste Tshibangu-Kabamba, Natsuko Kaku, Eisuke Adachi, Mayo Yasugi, Takuya Yamamoto, Takuto Nogimori, Yoshiyuki Wakabayashi, Yasutoshi Kido
EPI_ISL_l9027495. EPI_ISL_19027496, EPI_ISL_19027497. EPI_ISL_19027498, EPI_ISL_19027499, EPI_ISL_l9027500. EPI_ISL_19027501, EPI_ISL_19027502. EPI_ISL_19027503, EPI_ISL_19027504, EPI_ISL_19027505. EPI_ISL_19027506, EPI_ISL_19027507. EPI_ISL_19027508, EPI_ISL_19027509, EPI_ISL_19027511, EPI_ISL_19027512, EPI_ISL_19027513. EPI_ISL_19027514, EPI_ISL_19027515, EPI ISL 19027516, EPI ISL 19027517, EPI ISL 19027518, EPI ISL 19027519, EPI ISL 19027520, EPI ISL 19027521, EPI ISL 19027522, EPI ISL 19027523, EPI ISL 19027524, EPI ISL 19027525, EPI ISL 19027526, EPI ISL 19027527, EPI ISL 19027528, EPI ISL 19027529, EPI ISL 19027530, EPI ISL 19027531, EPI ISL 19027532, EPI ISL 19027533, EPI ISL 19027534, EPI ISL 19027535, EPI=ISL=l9027536, EPI=ISL)9027S37, EPÜSL=19027538, EPI=ISL=19027539, EPI=ISL)9027540, EPI=ISL=l9027541, EPI=ISL)9027543, EPÜSL=19027544, EPI=ISL)9027545, EPI=ISL)9027546, EPI=ISL=l9027547, EPI=ISL)9027548, EPÜSL=19027549, EPI=ISL)9027551, EPI=ISL)9027552, EPI=ISL=19027554, EPI=ISL)9027555, EPÜSL=19027556, EPI=ISL)9027557, EPI=ISL)9027558, EPI_ISL_l9027559, EPI_ISL_19027560, EPI_ISL_19027561, EPI_ISL_19027562, EPI_ISL_19027563, EPI_ISL_l9027564, EPI_ISL_19027565, EPI_ISL_l9027566, EPI_ISL_19027568, EPI_ISL_19027569, EPI_ISL_l9027570, EPI_ISL_19027571, EPI_ISL_19027572, EPI_ISL_l9027573, EPI_ISL_19027574, EPI_ISL_l9027575, EPI_ISL_19027576			
see above	NYC Department of Health and Mental Hygiene, Public Health Laboratory	NYC Department of Health and Mental Hygiene. Public Health Laboratory	Clabby,T.T., WangJC., Amin,H.S., Taki,F., Su,M., De la Cruz,N.. Olsen.A.. Thi,C., Silver,S., Akther.S., Chowdhury,M.. Omoregie,E. and Siemetzki-Kapoor,U
EPI ISL 19028679, EPI ISL 19028680, EPI ISL 19028681, EPI ISL 19028682, EPI ISL 19028683, EPI ISL 19028684, EPI ISL 19028685, EPI ISL 19028686, EPI ISL 19028687, EPI ISL 19028688, EPI ISL 19028689, EPI ISL 19028690, EPI ISL 19028691, EPI ISL 19028692, EPI ISL 19028693, EPI ISL 19028694, EPI ISL 19028695, EPI ISL 19028696, EPI ISL 19028697, EPI ISL 19028698, EPI-ISL-19028699, EPI-ISL-19028700, EPI-ISL-19028701, EPI-ISL-19028702, EPI-ISL-19028703, EPI-ISL-19028704, EPI-ISL-19028705, EPI-ISL-19028706, EPI-ISL-19028707, EPI-ISL-19028708, EPI-ISL-19028709, EPI-ISL-19028710, EPI-ISL-19028711, EPI-ISL-19028712, EPI-ISL-19028713, EPI-ISL-19028714, EPI-ISL-19028715, EPI-ISL-19028716, EPI-ISL-19028717, EPI-ISL-19028718, EPCISL)9028719. EPI=ISL)9028720, EPÜSL)9028721. EPCISL=19028722, EPI=ISL)9028723, EPCISL)9028724. EPI=ISL)9028725, EPÜSL)9028726. EPCISL=19028727, EPI=ISL)9028728, EPCISL=l9028729. EPI=ISL)9028730, EPÜSL)9028731. EPCISL=19028732, EPI=ISL)9028733, EPCISL=19028734. EPI=ISL=19028735, EPÜSL)9028736. EPCISL)9028737, EPI=ISL)9028738, EPI ISL 19028739, EPI ISL 19028740, EPI ISL 19028741, EPI ISL 19028742, EPI ISL 19028743, EPI ISL 19028744, EPI ISL 19028745, EPI ISL 19028746, EPI ISL 19028747, EPI ISL 19028748, EPI ISL 19028749, EPI ISL 19028750, EPI ISL 19028751, EPI ISL 19028752, EPI ISL 19028753, EPI ISL 19028754, EPI ISL 19028755, EPI ISL 19028756, EPI ISL 19028757, EPI ISL 19028758, EPI-ISL-19028759, EPI-ISL-19028760, EPI-ISL-19028761, EPI-ISL-19028762, EPI-ISL-19028763, EPI-ISL-19028764, EPI-ISL-19028765, EPI-ISL-19028766, EPI-ISL-19028767, EPI-ISL-19028768, EPI-ISL-19028769, EPI-ISL-19028770, EPI-ISL-19028771, EPI-ISL-19028772, EPI-ISL-19028773, EPI-ISL-19028774, EPI-ISL-19028775, EPI-ISL-19028776, EPI-ISL-19028777, EPI-ISL-19028778, EPI-ISL-19028779, EPI-ISL-19028780, EPI-ISL-19028781, EPI-ISL-19028782, EPI-ISL-19028783, EPI-ISL-19028784, EPI-ISL-19028785, EPI-ISL-19028786, EPI-ISL-19028787, EPI-ISL-19028788, EPI-ISL-19028789, EPI-ISL-19028790, EPI-ISL-19028791, EPI-ISL-19028792, EPI-ISL-19028793, EPI-ISL-19028794, EPI-ISL-19028795, EPI-ISL-19028796, EPI-ISL-19028797, EPI-ISL-19028798, EPI=ISL=19028799, EPI=ISL=l9028800, EPÜSL=l9028801, EPI=ISL=l9028802, EPI=ISL=l9028803, EPI=ISL=19028804, EPI=ISL=l9028805, EPÜSL=l9028806, EPI=ISL=l9028807, EPI=ISL=l9028808, EPI=ISL=19028809, EPI=ISL=l9028810, EPÜSL=l9028811, EPI=ISL=19028812, EPI=ISL=l9028813, EPI=ISL=19028814, EPI=ISL=l9028815, EPÜSL=l9028816, EPI=ISL=19028817, EPI=ISL=l9028818			
see above	British Columbia Centre For Oisease Control	BCCDC Public Health Laboratory	Prystajecky,Natalie; Tyson,John; Jassem,Agatha; Lee.Tracy; Azana,Rob; Fung,Janet; Chan.Michael; Cheung,Branco; Tsang,Frankie; Newman.Tara; Yang.Kevin; Russell.Shannon; Zlosnik,James; Hoang,linda
EPI ISL 19031631, EPI ISL 19031632. EPI ISL 19031633. EPI-ISL-19031634, EPI-ISL-19031635, EPI-ISL-19031636, - - EPI_ISL_19031637, EPI_ISL_19031638	Ouest Diagnostics	RIPHL at Rush University Medical Center	Stefan Green, Kevin Kunstman, Hannah Barbian. Sofiya Bobrovska. Felix Araujo Perez, Erin Newcomer, Alyse Kitttner
EPI_ISL_l9032701	Institute for Hepatology, Shenzhen Third People's Hospital	Institute for Hepatology, Shenzhen Third People's Hospital	Lin Cheng,Zheng Zhang
EPI_ISL_l9053766	Laboratory Medicine and Pathology, University of Washington	Laboratory Medicine and Pathology, University of Washington	Roychoudhury,P., Xie.X., Sereewit,J., Ellis,S. and Greninger,A.
EPI ISL 19058864, EPI ISL 19058865, EPI ISL 19058866, EPI=ISL)9058867, EPI=ISL)9058868, EPI=ISL)9058869, EPI_ISL_19058870, EPI_ISL_19058871, EPI_ISL_19058872, EPI_ISL_l9058873	Guangdong Provincial Center for Disease Contrai and Prevention, Institute of Pathogenic Microbiology	Guangdong Provincial Center for Disease Contrai and Prevention, Institute of Pathogenic Microbiology	li,B., Zhao.W. and Shen,C.
EPI ISL 19058874. EPI ISL 19058875	Massachusetts State Public Health laboratory, Department of Public Health	Massachusetts State Public Health laboratory, Department of Public Health	Doucette,M.. Fortes,E.. Bhattacharyya,S. and Epie,N.
EPI_ISL_19061081. EPI_ISL_l9061082, EPI_ISL_19061083	Rhode Island State Health Laboratory	Rhode Island State Health Laboratory	Richard C. Huard, Kristin Carpenter-Azevedo, Sean Sierra-Patev, Geoffrey Gasselin, Courtney Sowa
EPI_ISL_l9064115	Ouest Diagnostic Nichais Institute	los Angeles County Public Health laboratories	N. Heibeck et. al.
EPI_ISL_19064116	UCLA Healthcare Clinical Lab, Brentwood	Los Angeles County Public Health Laboratories	N. Heibeck et. al.
EPI_ISL_l9073514, EPI_ISL_l9073515, EPI_ISL_l9073516, EPI_ISL_19073517, EPI_ISL_l9073518. EPI_ISL_19073519	Ouest Diagnostics	RIPHL at Rush University Medical Center	Stefan Green, Kevin Kunstman, Hannah Barbian, Sofiya Bobrovska, Felix Araujo Perez, Erin Newcomer, Alyse Kitttner
EPI_ISL_19079342, EPI_ISL_19079343, EPI_ISL_19079344	Centre de Recherche en Sciences Naturelles de Lwiro (CRSN Lwiro)	Centre de Recherche en Sciences Naturelles de Lwiro (CRSN Lwiro)	Leandre M Masirika, Anuj Kumar, Mansi Dutt, Ali Toloue Ostadgavahi, Benjamin Hewins, Maliyamungu B Nadine, Bilembo K Steeven, Franklin K Mweshi, léandre M Mambo, Justin B Mbiribindi, Freddy B Siangoli, Alyson A Kelvin, Jean Claude Udahemuka, Patricia Kelvin, Luis Flores, David J Kelvin, Gustavo Sganzerla Martinez
EPI_ISL_l9093415	ARUP Laboratories	Los Angeles County Public Health Laboratories	N. Heibeck et al.
EPI ISL 19093789, EPI ISL 19093791, EPI ISL 19093793, EPI ISL 19093796, EPI ISL 19093798, EPI ISL 19093801, EPI ISL 19093802, EPI ISL 19093803, EPI ISL 19093804, EPI ISL 19093806, EPI ISL 19093807, EPI ISL 19093808, EPI ISL 19093809, EPI ISL 19093810, EPI ISL 19093811, EPI ISL 19093812. EPI ISL 19093813, EPI ISL 19093815, EPI ISL 19093817, EPI ISL 19093818, eP1=1sL)9093Bl9, EPC1sL)9093820, EPÜSL)9093822. EPCisL)9093823, EPC1sL)9093825, eP1=1sL)9093826, EPC1sL)9093827, EPÜSL)9093828. EPC1sL)9093829, EPC1sL)9093830, EP1=1sL)9093831, EPC1sL)9093832, EPÜSL)9093833. EPC1sL)909383S			
see above	Pathogen Genomic Laboratory, Institut National de Recherche Biomedicale	Pathogen Genomic Laboratory, Institut National de Recherche Biomedicale	Vakaniaki,E.H., Kaciat,C, Kinganda - Lusamaki,E., O'Toole,A., Wawina -Bokalanga,T.. Mukadi - Bamuleka,D.. Amuri,A.A., Parker,E., Muswamba-Kayembe,P.-C., Makangara - Cigolo,J.-C., Mulopo - Mukanya,N., Pukuta - Simbu,E., Mujula,Y., Nundu,S.S., Akil Bandali,P., Kavunga,H., Lushima,R.S., Vercauteren,K., Sam-Agudu,N.A., Mills,E.J., Tshiani - Mbaya,O., Hoff,N., Rimoin,A.W., Hensley,L.E., Kundrachuk,J., Ayouba,A., Peeters,M., Delaporte,E., Nachega,J.B., Ahuka - Mundeke,S., Muyembe - Tamfum,J.-J., Rambaut,A., liesenborghs,L. and Mbala - Kingebeni,P.
EPI_ISL_19107667	CT Department of Public Health	CT Department of Public Health	Claire Pearson, Tu N. Nguyen, Kutluhan Incekara, Neranjan V. Perera
EPI_ISL_l9108154, EPI_ISL_l9108156, EPI_ISL_l9108157, EPI_ISL_19108158, EPI_ISL_19108159, EPI_ISL_l9108160, EPI_ISL_l9108161. EPI_ISL_l9108165, EPI_ISL_19108167, EPI_ISL_l9108169, EPI_ISL_l9108170, EPI_ISL_19108171			
see above	Oxford University Clinical Research Unt	Oxford University Clinical Research Unt	Huynh Thi Thuy Hoa, Nguyen Thanh Dung, Le Manh Hung, Nguyen Thi Thu Hong, Vo Truong Quy, Hoang Truong, Nguyen Trang Duy, Tran Minh Hoang, Nguyen Thi Thanh, Mai Hong Phuoc, Nguyen Nhut Thong, Nguyen Duc Huy, Vu Thi Kim Thoa, Nghiem My Ngoc, Vo Trang Vuong, Ngo Tan Tai, Huynh Kim Nhung, Dao Phuong linh, Pham Thi Ngoc Thoa, Lam Minh Yen, Nguyen Thi Thao, Tran Ba Thien, Truong Hoang Chau Truc, Le Kim Thanh, Vo Tan Hoang,

			Nguyen Thanh Ngoc, Tran Tan Thanh, Louise Thwaites, Nguyen Van Vinh Chau, Guy Thwaites, Nguyen To Anh, Le Van Tan
EPI_ISL_l9109197	Laboratory Medicine and Pathology, University of Washington	Laboratory Medicine and Pathology, University of Washington	Roychoudhury,P., Xie,X., Sereewit,J., Ellis.S. and Greninger,A.
EPI ISL 19116565	Department of Public Health, Massachusetts State Public Health Laboratory	Department of Public Health, Massachusetts State Public Health Laboratory	Doucette,M., Fortes,E., Bhattacharyya,S. and Epie,N.
EPI ISL 19131254, EPI ISL 19131255, EPJ ISL 19131256, EPI JSL 19131257, EPI ISL 19131258, EPI ISL 19131260, EPI ISL 19131262, EPI ISL 19131263, EPI ISL 19131264, EPI ISL 19131265, EPI ISL 19131266, EPI ISL 19131267, EPI ISL 19131268, EPI ISL 19131269, EPI ISL 19131270, EPI ISL 19131271, EPI ISL 19131272, EPI ISL 19131273, EPI ISL 19131274, EPI ISL 19131275, EPCISL)9131276. EPI=ISL)9131277			
see above	NYC Department of Health and Mental Hygiene, Public Health Laboratory	NYC Department of Health and Mental Hygiene, Public Health Laboratory	Clabby,T., Amin,H.S., Taki,F., Su,M., Wang,J.C., De La Cruz,N., Olsen,A., Thi,C., Akther,S., Chowdhury,M., Omoregie,E., Polanco,M. and Chen,X.
EPI_ISL_19131278, EPI_ISL_19131279	Laboratory Medicine and Pathology, University of Washington	Laboratory Medicine and Pathology, University of Washington	Roychoudhury,P.. Xie.X., Sereewit,J., Ellis,S. and Greninger,A.
EPI_ISL_19131334, EPI_ISL_19131335, EPI_ISL_19131336, EPI_ISL_19131337, EPI_ISL_19131338, EPI_ISL_19131339, EPI_ISL_19131340, EPI_ISL_19131341, EPI_ISL_19131342, EPI_ISL_19131343, EPI_ISL_19131344, EPI_ISL_19131345			
see above	Indian Council of Medical Research-National Institute of Virology, Microbial Containment Complex	Indian Council of Medical Research-National Institute of Virology, Microbial Containment Complex	Pragya D. Yadav
EPJ_ISL_19136974, EPI_JSL_19136975	CT Department of Public Health	CT Department of Public Health	Claire Pearson, Tu N. Nguyen, Kutluhan Incekara, Neranjan V. Perera
EPI_ISL_l9139151	OUEST DIAGNOSTICS WEST HILLS	Los Angeles County Public Health Laboratories	N. Heibeck et al.
EPI_ISL_19139937, EPI_ISL_19139938, EPI_ISL_19139939, EPI_ISL_l9139940, EPI_ISL_19139941, EPI_ISL_19139942, EPI_ISL_19139943, EPI_ISL_19139944, EPI_ISL_19139945, EPI_ISL_19139946, EPI_ISL_19139947			
see above	Centre for Biological Threats. Highly Pathogenic Viruses, Robert Koch Institute	Centre for Biological Threats, Highly Pathogenic Viruses, Robert Koch Institute	Brinkmann,A., Kohl,C., Schrick,L., Michel,]., Schaade,L. and Nitsche,A.
EPI_ISL_19141102	Changchun Veterinary Research Institute, Chinese Academy of Agricultural Sciences	Changchun Veterinary Research Institute. Chinese Academy of Agricultural Sciences	Chen,M., Shi,S., Shan9,C. and Jiang.O.
EPI_ISL_19143445. EPI_ISL_19143446, EPI_ISL_19143447, EPI_ISL_l9143448, EPI_ISL_19143449, EPI_ISL_19143450. EPI_ISL_191434Sl, EPI_ISL_19143452, EPI_ISL_l9143453, EPI_ISL_19143454, EPI_ISL_19143455. EPI_ISL_19143458, EPI_ISL_19143459, EPI_ISL_l9143460, EPI_ISL_19143461, EPI_ISL_l9143462. EPI_ISL_19143464, EPI_ISL_19143467, EPI_ISL_l9143468, EPI_ISL_19143469, EPI_ISL_19143470, EPI_ISL_19143471, EPI_ISL_19143472, EPI_ISL_19143473, EPI_ISL_19143475, EPI_ISL_l9143476			
see above	California Department of Public Health	California Department of Public Health	Kath, C., Haw, M., Espinosa, A., and Hacker, J.
EPI_ISL_19151800, EPI_ISL_19151801	Tokyo Metropolitan Institute of Public Health	Tokyo Metropolitan Institute of Public Health	Wakaba Okada, Ryota Kumagai, Sachiko Harada, Yu Yaoita, Arisa Amano, Kumiko Takahashi, Mami Nagashima, Kenji Sadamasu
EPI_ISL_l91SS828	OUEST DIAGNOSTIC N1CHOLS INSTITUTE	Los Angeles County Public Health Laboratories	S. McCann et. al.
EPI_ISL_l9155829	ARUP	Los Angeles County Public Health Laboratories	S. McCann et. al.
EPI_ISL_l9155830	Laboratory Corporation of America	Los Angeles County Public Health Laboratories	S. McCann et. al.
EPI ISL 19157973, EPI ISL 19157974, EPI ISL 19157975, EPI-ISL-19157976, EPI-ISL-19157977, EPI-ISL-19157978, EP1_1SL_1915-79i9, EPI_ISL_191579fO	Centre Pasteur du Cameroun, Virology	Centre Pasteur du Cameroun, Virology	Djuicy,D.D., Sadeuh-Mba,S.A., Bilounga,C.N., Yonga,M.G., Tchatchueng-Mbougua,J.B., Essima,G.D, Esso,L., Nguidjol,I.M.E., Metomb,S.F, Chebo,C., Agwe,S.M., Ankone,P.A., Ngonla,F.N.N., Mossi,H.M., Etoundi,A.G.M., Eyangoh,5.1., Kazanji,M. and Njouom,R.
EPI_ISL_l9158911, EPI_ISL_l9158913	HCMC Hospital of Dermato Venereology	STIs Lab, Pasteur Institute in Ho Chi Minh City	Yen Nhi Nguyen, Tam-Duong Le-Ha, Lien Le, Hanh Lan Nguyen Thi, Thang Minh Cao, Thinh Viet Nguyen, Ouang Duy Pham, Ouang Luang Chan, Thuong Vu Nguyen, Trung Vu Nguyen
EPI_ISL_l9158914	Thu Duc Medical Center	STIS Lab, Pasteur Institute in Ho Chi Minh City	Yen Nhi Nguyen, Tam-Duong Le-Ha, Lien Le, Hanh Lan Nguyen Thi, Thang Minh Cao, Thinh Viet Nguyen, Ouang Duy Pham, Ouang Luang Chan, Thuong Vu Nguyen, Trung Vu Nguyen
EPI ISL 19158915, EPI ISL 19158916, EPI ISL 19158917, EPI-=-ISL-=-19158919	HCMC Hospital of Dermato Venereology	STIs Lab, Pasteur Institute in Ho Chi Minh City	Yen Nhi Nguyen, Tam-Duong Le-Ha, Lien Le, Hanh Lan Nguyen Thi, Thang Minh Cao, Thinh Viet Nguyen, Ouang Duy Pham, Ouang Luang Chan, Thuong Vu Nguyen, Trung Vu Nguyen
EPI_ISL_l9158924	Can Tho Hospital of Dermato Venereology	STIs Lab, Pasteur Institute in Ho Chi Minh City	Yen Nhi Nguyen, Tam-Duong Le-Ha, Lien Le, Hanh Lan Nguyen Thi, Thang Minh Cao, Thinh Viet Nguyen, Ouang Duy Pham, Ouang Luang Chan, Thuong Vu Nguyen, Trung Vu Nguyen
EPI ISL 19158928, EPI I5L 19158931. EPI ISL 19158934, EPI_ISL_l915894Û, EPI_ISL_l915894-l	HCMC Hospital of Dermato Venereology	STIs Lab, Pasteur Institute in Ho Chi Minh City	Yen Nhi Nguyen, Tam-Duong Le-Ha, Lien Le, Hanh Lan Nguyen Thi, Thang Minh Cao, Thinh Viet Nguyen, Ouang Duy Pham, Ouang Luang Chan, Thuong Vu Nguyen, Trung Vu Nguyen
EPI ISL 19158945, EPI ISL 19158946, EPI ISL 19158947, EPI=ISL)9158948, EPI=ISL)9158949, EPI=ISL=19158950, EPI_ISL_l9158951	Northwestern Medicine	RIPHL at Rush University Medical Center	Stefan Green, Kevin Kunstman, Hannah Barbian, Sofiya Bobrovska, Felix Araujo Perez, Erin Newcomer, Alyse Kitttner
EPI_ISL_l9158952	Ouest Diagnostics	RIPHL at Rush University Medical Center	Stefan Green, Kevin Kunstman, Hannah Barbian. Sofiya Bobrovska. Felix Araujo Perez, Erin Newcomer, Alyse Kitttner
EPI_ISL_19159108	RSPI Sulianti Saroso	Balai 8esar Laboratorium Biologi Kesehatan	Hana Apsari Pawestri, Arie Ardiansyah Nugraha, Fajar Nur Sulistyahadi, Markus Evan Anggia, Subangkit
EPI_ISL_l9159109	RSUP Dr Hasan Sadikin	Balai Besar Laboratorium Biologi Kesehatan	Hana Apsari Pawestri, Arie Ardiansyah Nugraha, Fajar Nur Sulistyahadi, Markus Evan Anggia, Subangkit
EPI_ISL_19159110	Puskesmas Cilodong	Balai Besar Laboratorium Biologi Kesehatan	Hana Apsari Pawestri, Arie Ardiansyah Nugraha, Fajar Nur Sulistyahadi, Markus Evan Anggia, Subangkit
EPI_ISL_l9159111	RSUD Cengkareng	Balai Besar Laboratorium Biologi Kesehatan	Hana Apsari Pawestri, Arie Ardiansyah Nugraha, Fajar Nur Sulistyahadi, Markus Evan Anggia, Subangkit
EPI_ISL_19159112	Eka Hospital BSD	Balai Besar Laboratorium Biologi Kesehatan	Hana Apsari Pawestri, Arie Ardiansyah Nugraha, Fajar Nur Sulistyahadi. Markus Evan Anggia, Subangkit
EPI_ISL_19159113	PKC Tebet	Balai Besar Laboratorium Biologi Kesehatan	Hana Apsari Pawestri, Arie Ardiansyah Nugraha, Fajar Nur Sulistyahadi, Markus Evan Anggia, Subangkit
EPI_ISL_l9159114	PKC Menteng	Balai Besar Laboratorium Biologi Kesehatan	Hana Apsari Pawestri, Arie Ardiansyah Nugraha, Fajar Nur Sulistyahadi, Markus Evan Anggia, Subangkit
EPJ_ISL_l9159115, EPI_JSL_l9159116	PKC Tanjung Priok	Balai Besar Laboratorium Biologi Kesehatan	Hana Apsari Pawestri, Arie Ardiansyah Nugraha, Fajar Nur Sulistyahadi, Markus Evan Anggia, Subangkit
EPI_ISL_l9159119	RSPI Sulianti Saroso	Balai Besar Laboratorium Biologi Kesehatan	Hana Apsari Pawestri, Arie Ardiansyah Nugraha, Fajar Nur Sulistyahadi, Markus Evan Anggia, Subangkit
EPI_ISL_19159120	RSUP Fatmawati	Balai Besar Laboratorium Biologi Kesehatan	Hana Apsari Pawestri, Arie Ardiansyah Nugraha, Fajar Nur Sulistyahadi. Markus Evan Anggia, Subangkit
EPI ISL 19170459, EPI ISL 19170460, EPI ISL 19170461, EPI ISL 19170462, EPI ISL 19170465, EPI ISL 19170466, EPI ISL 19170467, EPI ISL 19170468, EPI ISL 19170469, EPI ISL 19170470, EPI ISL 19170471, EPI ISL 19170472, EPI ISL 19170473, EPI ISL 19170474, EPI ISL 19170475, EPI ISL 19170476, EPI ISL 19170477, EPI ISL 19170478, EPI ISL 19170479, EPI ISL 19170480, EPCISL)9170481. EPI=ISL)9170482, EPÜSL)9170483, EPCISL)9170486, EPI=ISL)9170487, EPCISL)9170488, EPI=ISL)9170489, EPÜSL)9170490			
see above	California Department of Public Health	California Department of Public Health	Kath, C.. Haw, M.. Espinosa. A., and Hacker. J.
EPI_ISL_l9179078	CT Department of Public Health	CT Department of Public Health	Claire Pearson, Tu N. Nguyen, Kutluhan Incekara, Neranjan V. Perera
EPI_ISL_l9185424, EPI_ISL_l9185425	Rhode Island State Health Laboratory	Rhode Island State Health Laboratory	Richard C. Huard, Kristin Carpenter-Azevedo, Sean Sierra-Patev, Geoffrey Gasselin, Courtney Sowa
EPI ISL 19193026, EPI JSL 19193027	Massachusetts State Public Health Laboratory, Department of Public Health	Massachusetts State Public Health Laboratory, Department of Public Health	Ooucette,M., Fortes,E., Bhattacharyya,S. and Epie,N.
EPI_ISL_l9193028	California Department of Public Health, Viral and Rickettsial Disease Laboratory	California Department of Public Health, Viral and Rickettsial Disease Laboratory	Kath,C., Haw,M., Espinosa,A. and Hacker,J.
EPI_ISL_l9196357	Laboratory Corporation of America	Los Angeles County Public Health Laboratories	S. McCann et. al.
EPI_ISL_19196358	Ouest Diagnostics West Hills	Los Angeles County Public Health Laboratories	S. McCann et. al.
EPI_ISL_l9196359	Ouest Diagnostic Nichais Institute	Los Angeles County Public Health Laboratories	S. McCann et. al.
EPJ_ISL_19204032. EPI_ISL_l9204033	Laboratory Corporation of America	Los Angeles County Public Health Laboratories	S. McCann et. al.
EPI_ISL_19204034	OUEST DIAGNOSTIC NICHOLS INSTITUTE	Los Angeles County Public Health Laboratories	S. McCann et. al.
EPI_ISL_l9204035	OUEST DIAGNOSTICS WEST HILLS	Los Angeles County Public Health Laboratories	S. McCann et. al.
EPI ISL 19205399, EPI ISL 19205401, EPI ISL 19205402, EPI=ISL)9205403, EPI=ISL)9205404, EPI=ISL)920S40S, EPI_ISL_19205406, EPI_ISL_l9205407	Laboratorio de Enterovirus, Instituto Oswaldo Cruz, Fiocruz	Instituto Oswaldo Cruz FIOCRUZ - Laboratory of Respiratory Viruses and Measles (LVRS)	Paola Resende, Elisa Cavalcante Pereira, Bruna Mendonça da Silva, Jéssica Graça Macedo de Carvalho, Larissa Macedo Pinto, Victor Guimaraes, Luciana Appolinario, Alice Sampaio, Marilda Siqueira, Renan da Silva Faustino, Marilia Santini, Edson Elias da Silva on behalf of the Fiocruz Genomic Surveillance Network
EPI_ISL_19226017	Rhode Island State Health Laboratory	Rhode Island State Health Laboratory	Richard C. Huard, Kristin Carpenter-Azevedo. Sean Sierra-Patev, Geoffrey Gasselin, Courtney Sowa
EPI_ISL_19230662, EPI_ISL_19230663, EPI_ISL_19230664, EPI_ISL_19230665, EPI_ISL_l9230666	Charite - Universitatsmedizin Berlin	Charite - Universitatsmedizin Berlin	Obermeier,P.E, Plinke,C.F., Brinkmann,A., Lachmann,R., Melchert,J., Corman,V.M., Nitsche,A., Marcus,U., Schmidt,A.J., Jansen,K. and Buder,S.C.
EPI_ISL_19230668	Virologia, INEI- ANUS Dr. Carlos G. Malbran	Virologia. INEI- ANUS Dr. Carlos G. Malbran	Lewis.A., Josiowicz.A.. Poklepovich,T.. Mallou.F.. Cuba,F.. Haim.M. and Cisterna,D.

EPI_ISL_13052263	Micrabiol Genomics and Bioinformatics, Bundeswehr Institute of Microbiology	Microbiol Genomics and Bioinformatics, Bundeswehr Institute of Microbiology	Antwerpen,M.H., Lang,D., Zange,S., Walter,M.C. and Woelfel,R.
EPI ISL 13052266, EPI ISL 13052267, EPI-ISL-13052268, EPI-ISL-13052269, EPI-ISL-13052270, EPI-ISL-13052272, - - EPI_ISL_130522f3	Instituto Nacional de Saude Doutor Ricardo Jorge (INSA)	Instituto Nacional de Saude Doutor Ricardo Jorge (INSA)	Joana Isidro, Vftor Borges, Miguel Pinto, Daniel Sobral, Joiio Dourado Santos, Alexandra Nunes, Ver6nica Mixiio, Rita Ferreira, Daniela Santos, Silvia Duarte, Lufs Vieira, Maria José Borrego, Sofia NCincio, Isabel Lapes de Carvalho, Ana Pelerito, Rita Cordeiro, Joiio Paulo Gomes
EPI_ISL_l3052274	Laboratory of Virology, University Hospitals of Geneva	Laboratory of Virology, University Hospitals of Geneva	Laubscher,F., Chudzinski,V., Schibler,M., Kaiser,L. and Renzoni,A.
EPI_ISL_l3052275	IHAP, VIRAL, Universite de Toulouse, INRAE, ENVT	IHAP, VIRAL, Universite de Toulouse, INRAE, ENVT	Croville,G., Walch,M., Guerin,J.-L., Mansuy,J.-M., Pasquier,C. and Izopet,J.
EPI_ISL_l3052276	Laboratory of Virology, INMI Lazzara Spallanzani IRCCS	Laboratory of Virology, INMI Lazzaro Spallanzani IRCCS	Gruber,C.E.M., Rueca,M., rGramigna,G., Car-letti,F., Butera,O., Fabeni,L., Specchiarello,E., Meschi,S., Colavita,F., Minosse,C., Francalancia,M., Lapa,□., Garbuglia,A.R. and Giombini,E.
EPI_ISL_l3052277	Public Health Virology, Erasmus Medical Centre	Public Health Virology, Erasmus Medical Centre	Oude Munnink,B.B., Boter,M., Wellers,B., Molenkamp,R., Sikkema,R.S. and Koopmans,M.
EPI_ISL_l3052278	Research and Evaluation, UKHSA	Research and Evaluation, UKHSA	Osman,K.L., Lewandowski,K.S., Pullan,S.T., Carter,D.P., Crook,J.M., Vipond,R. and Chand,M.
EPI_ISL_l3052279, EPI_ISL_l3052280, EPI_ISL_l3052281	Research and Evaluation, UKHSA	Research and Evaluation, UKHSA	Osman,K.L., Lewandowski,K.S., Carter,D.P., Craok,J.M., Pullan,S.T., Vipond,R. and Chand,M.
EPI_ISL_l3052282	Microbiology, Immunology and iTransplantation, KU Leuven, Rega Institute	Microbiology, Immunology and Transplantation, KU Leuven, Rega Institute	Vanmechelen,B., Wawina-Bokalanga,T., Logist,A.-S., Sinnesael,R., Ysebaert,L., VerlindenJ., Bloemen,M. and Maes,P.
EPI_ISL_l3052283	Microbiology, Immunology and iTransplantation, KU Leuven, Rega Institute	Microbiology, Immunology and Transplantation, KU Leuven, Rega Institute	Wawina-Bokalanga,T., Vanmechelen,B., Logist,A.-S., Sinnesael,R., Ysebaert,L., Verlinden,J., Bloemen,M. and Maes,P.
EPI_ISL_l3052284	Micrabiology, Hospital Universitari Germans Trias i Pujol	Micrabiology, Hospital Universitari Germans Trias i Pujol	Martinez-Puchol,S., Coello,A., Bordoy,A.E., Soler,L., Panisello,D., Gonzalez-Gomez,S., Clara,G., Paris de Leon,A., Not,A., Hernandez,A., Bofill-Mas,S., Saludes,V., Blanco,I., Martro,E. and Cardona,P.-J.
EPI_ISL_l3052285	Laboratory of Virology, University Hospitals of Geneva	Laboratory of Virology, University Hospitals of Geneva	Laubscher,F., Schibler,M., Kaiser,L. and Renzoni,A.
EPI_ISL_l3052287	Virology, GENomique EPIdemiologique des maladies Infectieuses	Virology, GENomique E.PIdemiologique des maladies Infectieuses	unknown
EPI_ISL_l3052288	Department of Health, Utah Public Health Laboratory	Department of Health, Utah Public Health Laboratory	Young,E.L., Hergert,J. and Oakeson,K.F.
EPI_ISL_l3052289	Centers for Disease Contrai & Prevention (CDC), Division of High Consequence Pathogens and Pathology (DHCPP-PRB)	Centers for Disease Contrai & Prevention (CDC), Division of High Consequence Pathogens and Pathology (DHCPP-PRB)	Gigante,C.M., Smole,S., Seabolt,M.H., Wilkins,K., McCollum,A., Hutson,C., Davidson,W., Rao,A., Brown,C. and Li,Y.
EPI_ISL_l3052290	Laboratory for Diagnostics of Zoonoses and WHO Centre, Institute of Microbiology and Immunology, Faculty of Medicine, University of Ljubljana	Laboratory for Diagnostics of Zoonoses and WHO Centre, Institute of Micrabiology and Immunology, Faculty of Medicine, University of Ljubljana	Zakotnik,S., Vlaj,D., Suljic,A., Zorec,T.M., Korva,M., Poljak,M. and Avsic Zupanc,T.
EPI_ISL_l3052291	Laboratory for Diagnostics of Zoonoses and WHO Centre, Institute of Micrabiology and Immunology, Faculty of Medicine, University of Ljubljana	Laboratory for Diagnostics of Zoonoses and WHO Centre, Institute of Micrabiology and Immunology, Faculty of Medicine, University of Ljubljana	Zakotnik,S., Vlaj,D., Suljic,A., Zorec,T.M., Skubic,C., Rozman,D., Korva,M., Poljak,M. and Avsic Zupanc,T.
EPI_ISL_l3052292	Victorian Infectious Diseases Reference Laboratory, Doherty Institute	Victorian Infectious Diseases Reference Laboratory, Doherty Institute	Hammerschlag,Y., MacLeod,G., Papadakis,G., Adan-Sanchez.A., Druce,J.D., Williamson,D.A., Cheng,A.C. and McMahon,J.H.
EPI_ISL_l3052293, EPI_ISL_l3052294	Centre for Biological Threats, Highly Pathogenic Viruses, Robert Koch Institute	Centre for Biological Threats, Highly Pathogenic Viruses, Robert Koch Institute	Brinkmann,A., Kohl,C., Uddin,S., Pape,K., Schrick,L., Michel,J., Schaade,L. and Nitsche,A.
EPI_ISL_l3052295	SC (UCO) Igiene e Sanità Pubblica, ASUGI, Trieste	Genomics and Epigenomics, AREA Science Park	Licastra,D., DeGasperi,M., Negri,C., Piscianz,E., Koncan,R., Dai Monego,S., Segat,L. and D'Agaro,P.
EPI ISL 13056271, EPI ISL 13056272, EPI=-ISL-=-13056273, EPC_ISL-=-13056274	Centers for Disease Contrai and Prevention	Centers f o rDisease Contrai and Prevention	Mauldin,M.R., McCollum,A.M., Nakazawa,Y.J., Mandra,A., Whitehouse,E.R., Davidson,W., Zhao,H., Gao,J., Li,Y., Doty,J., Yinka-Ogunleye,A., Akinpelu,A., Aruna,O., Naidoo,D., Lewandowski,K., Afrough,B., Graham.V., Aarans,E., Hewson,R., Vipond,R., Dunning,J., Chand,M., Brown,C., Cohen-Gihon,I., Erez,N., Shifman,0., Israeli,0., Sharon,M., Schwartz,E., Beth-Din,A., Zvi,A., Mak,T.M., Ng,Y.K., Cui,L., Lin,R.T.P., Olson,V.A., Brooks,T., Paran,N., Ihekweazu,C. and Reynolds,M.G.
EPI ISL 13056275, EPI ISL 13056276, EPI=ISL=13056277, EPI=ISL=l3056278, EPI ISL 13056279, EPI ISL 13056280, - - EPI_ISL_1305628-l	Centers for Disease Contrai and Prevention	Centers for Disease Contrai and Prevention	Yinka-Ogunleye,A., Aruna,O., Dalhat,M., Ogoina,D., McCollum,A., Disu,Y., Mamadu,I., Akinpelu,A., Ahmad,A., Burga,J., Ndoreraho,A., Nkunzimana,E., Manneh,L., Mohammed,A., Adeoye,O., Tom-Aba,D., Silenou,B., Ipadeola,O., Saleh,M., Adeyemo,A., Nwadiutor,I., Aworabhi,N., Uke,P., John,D., Wakama,P., Reynolds,M., Mauldin,M., Doty,J., Wilkins,K., Musa,J., Khalakdina,A., Adedeji,A., Mba,N., Ojo,O., Krause,G. and Ihekweazu,C.
EPI ISL 13056282, EPI ISL 13056283, EPI-ISL-13056284, EPI-ISL-13056285, - - EPI_ISL_l30562f6	Centers for Disease Contrai and Prevention	Centers for Disease Contrai and Prevention	Mauldin,M.R., McCollum,A.M., Nakazawa,Y.J., Mandra,A., Whitehouse,E.R., Davidson,W., Zhao,H., Gao,J., Li,Y., Doty,J., Yinka-Ogunleye,A., Akinpelu,A., Aruna,O., Naidoo,D., Lewandowski,K., Afrough,B., Graham.V., Aarans,E., Hewson,R., Vipond,R., Dunning,J., Chand,M., Brown,C., Cohen-Gihon,I., Erez,N., Shifman,O., Israeli,O., Sharon,M., Schwartz,E., Beth-Din,A., Zvi,A., Mak,T.M., Ng,Y.K., Cui,L., Lin,R.T.P., Olson,V.A., Brooks,T., Paran,N., Ihekweazu,C. and Reynolds,M.G.
EPI_ISL_13056289	Biochemistry and Molecular Biology, Israel Institute for Biological Research	Biochemistry and Molecular Biology, Israel Institute for Biological Research	Cohen Gihon,I., Israeli,0., Shifman,O., Erez,N., Melamed,S., Paran,N., Beth-Din,A. and Zvi,A.
EPI_ISL_l3056891	Centre for Clinical Infection & Diagnostics Research, King's College London, St Thomas Hospital	Centre for Clinical Infection & Diagnostics Research	Alcolea-Medina,A., Charalampous,T., Snell,L.B., Batra,R. and Edgeworth,J.D.
EPI_ISL_l3056892, EPI_ISL_l3056893, EPI_ISL_l3056894, EPI_ISL_l3056895, EPI_ISL_l3056896, EPI_ISL_l3056897, EPI_ISL_l3056899, EPI_ISL_l3056900, EPI_ISL_l3056901, EPI_ISL_l3056902, EPI_ISL_l3056903, EPI_ISL_l3056904, EPI_ISL_l3056905, EPI_ISL_l3056906, EPI_ISL_l3056907, EPI_ISL_l3056908, EPI_ISL_l3056909			
see above	Instituto Nacional de Saude Doutor Ricardo Jorge (INSA)	Instituto Nacional de Saude Douter Ricardo Jorge (INSA)	Joana Isidro, Vftor Borges, Miguel Pinto, Daniel Sabrai, Joiio Dourado Santos, Aléxandra Nunes, Ver6nica Mixiio, Rita Ferreira, Daniela Santos, Silvia Duarte, Lufs Vieira, Maria José Borrego, Sofia NCincio, Isabel Lapes de Carvalho, Ana Pelerito, Rita Cordeiro, Joiio Paulo Gomes
EPI_ISL_l3056910	Biochemistry and Molecular Genetics, Israel Institute for Biological Research	Biochemistry and Molecular Genetics, Israel Institute for Biological Research	Israeli,O., Guedj-Dana,Y., Lazar,S., Shifman,O., Erez,N., Weiss,S., Paran,N., Israely,T., Schuster,O., Zvi,A., Beth-Din,A. and Cohen Gihon,I.
EPI_ISL_l3058462, EPI_ISL_l3058463	Center for Genome Sciences, USAMRIID	Center f o rGenome Sciences, USAMRIID	Faye,O., Pratt,C.B., Faye,M., Fall,G., Chitty,J.A., Diagne,M.M., Wiley,M.R., Yinka-Ogunleye,A.F., Aruna,S., Etebu,E.N., Aworabhi,N., Ogoina,D., Numbere,W., Mba,N., Palacios,G., Sall,A.A. and Ihekweazu,C.
EPI ISL 13058465, EPI ISL 13058466, EPI=ISL)3058467, EPI=ISL)3058468, EPI ISL 13058469, EPI ISL 13058470, EPI-ISL-13058471, EPI-ISL-13058472, EP1-=_1SL-=_13058473, EPCisL-=_13058474	Virology, Centre International de Recherches Medicales de Franceville	Virology, Centre International de Recherches Medicales de Franceville	Selekon,B., Labouba,I,L., Gonofio,E.C., Sem Ouilibona,R., Sima Tchetgna,H., Besombes,C., Feher,M., Fontanet,A., Kazanji,M., Manugerra,J.-C., Gessain,A., Nakoune,E. and Berthet,N.
EPI_ISL_l3058475	National Public Health Laboratory, National Centre for Infectious Diseases	National Public Health Laboratory, National Centre for Infectious Diseases	Yong,S.E.F., Ng,O.T., Ho,Z.J.M., Mak,T.M., Marimuthu,K., Vasoo,S., Yeo,T.W., Ng,Y.K., Cui,L., Ferdous,Z., Chia,P.Y., Aw,B.J.W., Manauis,C.M., Low,C.K.K., Chan,G., Peh,X., Lim,P.L., Chow,L.P.A., Chan,M., Lee,V.J.M., Lin,R.T.P., Heng,M.K.D. and Leo,Y.S.
EPI_ISL_l3069002	Erasmus Medical Center Department of Virology	Erasmus Medical Center Department of Virology	Bas Oude Munnink, Marjan Boter, Babette Weller, Richard Molenkamp, Janette Rahamat-Langendoen, Reina Sikkema, Marion Koopmans
EPI_ISL_l3089461	Hospital General Universitario Gregorio Maran6n	Hospital General Universitario Gregorio Marai'i6n	Sergio Buenestado Serrano, Rosalia Palomino Cabrera, Daniel Pei'ias Utrilla, Jorge Rodrfguez-Grande, Laura Pérez-Lago, Cristina Rodriguez-Grande, Marta Herranz Martin, Julia Suérez, Pilar Catalén, Patricia Muriez, Darfo Garda de Viedma
EPI_ISL_l3090993	Virology, Instituto Nacional de Enfermedades Infecciosas	Virology, Instituto Nacional de Enfermedades Infecciosas	Lewis,A., Josiowicz,A., Bonaventura,R., Basiletti,J., Hirmas Riade,S.M., Tous,M. and Cisterna,D.M.
EPI_ISL_l3094227	Centers for Disease Contrai & Prevention (CDC), Division of High Consequence Pathogens and Pathology (DHCPP-PRB)	Centers for Disease Contrai & Prevention (CDC), Division of High Consequence Pathogens and Pathology (DHCPP-PRB)	Gigante,C.M., Lee,P., Seabolt,M.H., Wilkins,K., McCollum,A., Hutson,C., Davidson,W., Rao,A., Mendoza,R. and Li,Y.
EPI_ISL_13096615	Centers for Disease Contrai & Prevention (CDC), Division of High Consequence Pathogens and Pathology (DHCPP-PRB)	Centers for Disease Contrai & Prevention (CDC), Division of High Consequence Pathogens and Pathology (DHCPP-PRB)	Gigante,C.M., GriffinThomas,L.A., Seabolt,M.H., Wilkins,K., McCollum,A., Hutson,C., Davidson,w., Rao,A., Crain,J. and Li,Y.
EPI_ISL_l3100618	Centers for Disease Contrai & Prevention (CDC), Division of High Consequence Pathogens and Pathology (DHCPP-PRB)	Centers for Disease Contrai & Prevention (CDC), Division of High Consequence Pathogens and Pathology (DHCPP-PRB)	Gigante,C.M., Ventura,J., Seabolt,M.H., Wilkins,K., McCollum,A., Hutson,C., Davidson,W., Rao,A., Nash,J. and Li,Y.
EPI_ISL_l3100619	Centers for Disease Contrai & Prevention (CDC), Division of High Consequence Pathogens and Pathology (DHCPP-PRB)	Centers for Disease Contrai & Prevention (CDC), Division of High Consequence Pathogens and Pathology (DHCPP-PRB)	Gigante,C.M., Lee,P., Seabolt,M.H., Wilkins,K., McCollum,A., Hutson,C., Davidson,W., Rao,A., Mendoza,R. and Li.Y.
EPI_ISL_l3100620	Centers for Disease Contrai & Prevention (CDC), Division of High Consequence Pathogens and	Centers for Disease Contrai & Prevention (CDC), Division of High Consequence Pathogens and	Gigante,C.M., Atkinson,A., Seabolt,M.H., Wilkins,K., McCollum,A., Hutson,C., Davidson,W., Rao,A., Murray,]. and Li,Y.

	Pathology (DHCPP-PRB)	Pathology (DHCPP-PRB)	
EPI_ISL_l3100621	Centers for Disease Contrai & Prevention (CDC), Division of High Consequence Pathogens and Pathology (DHCPP-PRB)	Centers for Disease Contrai & Prevention {CDC). Division of High Consequence Pathogens and Pathology (DHCPP-PRB)	Gigante,C.M., Stringer,J., Seabolt,M.H., Wilkins,K., McCollum,A.. Hutson,C., Davidson,W., Rao.A., Schulte,J. and Li,Y.
EPI_ISL_l3100622	Centers for Disease Contrai & Prevention (CDC), Division of High Consequence Pathogens and Pathology (DHCPP-PRB)	Centers for Disease Contrai & Prevention {CDC). Division of High Consequence Pathogens and Pathology (DHCPP-PRB)	Gigante,C.M., Myers,R., Seabolt,M.H., Wilkins,K., McCollum,A., Hutson,C., Davidson,W., Rao,A., Blythe,D. and Li,Y.
EPI_ISL_l3100719	Centers for Disease Contrai & Prevention (CDC), Division of High Consequence Pathogens and Pathology (DHCPP-PRB)	Centers for Disease Contrai & Prevention {CDC). Division of High Consequence Pathogens and Pathology (DHCPP-PRB)	Gigante,C.M.. Ventura,J.. Seabolt,M.H.. Wilkins,K.. McCollum,A.. Hutson,C.. Davidson,W.. Rao.A.. Nash,J. and Li.Y.
EPI_ISL_l3106454	Hospital General Universitario Gregorio Marafi6n	Hospital General Universitario Gregorio Marai'i6n	Sergio Buenestado Serrano, Rosalfa Palomino Cabrera, Daniel Pefias Utrilla. Jorge Rodrfguez-Grande, Pedro Sola Campoy, Laura Pérez-Lago, Cristina Rodriguez-Grande, Marta Herranz Martin, Julia Suârez, Pilar Catalân, Patricia Mufioz, Darfo Garda de Viedma
EPI_ISL_l3117291	Centre for Biological Threats, Highly Pathogenic Viruses, Robert Koch Institute	Centre for Biological Threats, Highly Pathogenic Viruses, Robert Koch Institute	Brinkmann,A.. Kohl,C.. Uddin,S., Pape,K.. Schrick,l., Michel,J.. Jessen,H., Schaade,L. and Michel,A.
EPI_ISL_l3117292, EPI_ISL_l3117293	Centre for Biological Threats, Highly Pathogenic Viruses, Robert Koch Institute	Centre for Biological Threats, Highly Pathogenic Viruses, Robert Koch Institute	Brinkmann,A.. Kohl,C., Uddin,S., Pape,K.. Schrick,L., Michel,]., Stocker,H., Schaade,L. and Nitsche,A.
EPJ ISL 13117294, EPI JSL 13117295, EPÜSL)3117296, EPCISL)3117297, EPI_ISL_l3117298	Centre for Biological Threats, Highly Pathogenic Viruses, Robert Koch Institute	Centre for Biological Threats, Highly Pathogenic Viruses, Robert Koch Institute	Brinkmann,A., Kohl,C., Uddin,S., Pape,K., Schrick,L., Michel,J., Schaade,I. and Nitsche.A.
EPI_ISL_l3148263, EPI_ISL_l3148264, EPI_ISL_l3148265	Centre for Biological Threats, Highly Pathogenic Viruses, Robert Koch Institute	Centre for Biological Threats, Highly Pathogenic Viruses, Robert Koch Institute	Brinkmann,A., Kohl,C., Uddin,S., Pape,K., Schrick,L., Michel,J., Jessen,H., Schaade,I. and Nitsche,A.
EPI ISL 13148266, EPI ISL 13148267, EPI-=-JSL-=-13148268, EPC_ISL-=-13148269	Centre for Biological Threats, Highly Pathogenic Viruses, Robert Koch Institute	Centre for Biological Threats, Highly Pathogenic Viruses, Robert Koch Institute	Brinkmann,A., Kohl,C., Uddin,S,, Pape,K.. Schrick.L,, Michel,]., Stocker,H,, Schaade,I. and Nitsche,A.
EPJ ISL 13148270, EPI JSL 13148271, EPI=ISL)3148272, EPI=ISL)3148273, EPI ISL 13148274, EPI ISL 13148275, - - EPI_ISL_l314827-6	Centre for Biological Threats, Highly Pathogenic Viruses, Robert Koch Institute	Centre for Biological Threats, Highly Pathogenic Viruses, Robert Koch Institute	Brinkmann,A., Kohl,C., Uddin,S., Pape,K., Schrick,L., Michel,]., Schaade,I. and Nitsche,A.
EPI_ISL_l3157812	Laboratorio di Microbiologia e Virologia, Università Vita-Salute San Raffaele, Milano	Laboratorio di Microbiologia e Virologia, Università Vita-Salute San Raffaele, Milano	Benedetta Giuliani, Sofia Sisti, Michela Sampaolo, Elena Criscuolo, Matteo Castelli, Roberto Ferrarese, Roberta Antonia Oiotti, Massimo Locatelli, Massimo Clementi, Nicasio Mancini, Nicola Clementi
EPI_ISL_l3158444	Laboratorio di Microbiologia e Viralogia, Università Vita-Salute San Raffaele, Milano	Laboratorio di Microbiologia e Virologia, Università Vita-Salute San Raffaele, Milano	Sofia Sisti, Michela Sampaolo, Elena Criscuolo, Benedetta Giuliani, Matteo Castelli, Roberto Ferrarese, Martina Libera, Massimo Locatelli, Massimo Clementi, Nicasio Mancini, Nicola Clementi
EPI_ISL_l3159759	Laboratorio di Microbiologia e Viralogia, Università Vita-Salute San Raffaele, Milano	Laboratorio di Micrabiologia e Virologia, Università Vita-Salute San Raffaele, Milano	Roberto ferrarese, Benedetta Giuliani, Elena Criscuolo, Sofia Sisti, Michela Sampaolo, Matteo Castelli, Roberta Antonia Oiotti, Massimo Locatelli, Massimo Clementi, Nicasio Mancini, Nicola Clementi
EPI_ISL_l3191424	Virology, INEI-ANLIS Dr. Carlos G. Malbran,	Virology, INEI-ANLIS Or. Carlos G. Malbran,	Lewis,A., Josiowicz,A., Hirmas Riade,S.M., Tous,M. and Cisterna,D.M
EPI_ISL_l3191438	Instituto de Infectologia Emilio Ribas	Instituto Adolfo Lutz Strategic Laboratory	Claudio Tavares Sacchi, Karaline Rodrigues Campos, Marion Benedito Nascimento Santos, Alex Oomingos Reis, Ariadne Ferreira Amarante, Adriano Abbud, Adriana Bugna, Walkiria Oelnora Almeida Prado, Regiane Cardoso de Paula
EPI_ISL_l3194516	Alberta Precision Laboratories	Alberta Precision Laboratories	Matthew Croxen, Ashwin Deo, Paul Dieu, Xiaoli Dong, Kara Gill, David Granger, Christina Ferrato, Vanipriyadarsini Ikkurti, Jamil Kanji, Petya Koleva, Vincent Li, Colin Lloyd, Tarah Lynch, Raymond Ma, Kanti Pabbaraju, Silas Rotich, Hilary Sergeant, Steven Shideler, Todd Skitsko, Sandy Shokopoles, Graham Tipples, Johanna Thayer, Anita Wang
EPI_ISL_l3234112	Laborat6rio Central de SaUde Publica do Estado do Rio Grande do Sul	Instituto Adolfo Lutz Strategic Laboratory	Claudio Tavares Sacchi, Karaline Rodrigues Campos, Adriano Abbud, Adriana Bugna
EPI_ISL_l3242738	Hospital General Universitario Gregorio Marafi6n	Hospital General Universitario Gregorio Marai'i6n	Sergio Buenestado Serrano, Rosalfa Palomino Cabrera, Daniel Pefias Utrilla, Jorge Rodriguez-Grande, Pedro Sola Campoy, Laura Pérez-Lago, Cristina Rodriguez-Grande, Marta Herranz Martin, Julia Suârez, Pilar Catalân, Patricia Mufioz. Daria Garda de Viedma
EPI_ISL_l3244349, EPI_ISL_l3244610	Erasmus Medical Center Department of Viralogy	Erasmus Medical Center Oepartment of Virology	Bas Oude Munnink. Marjan Soter. Babette Weller, Richard Molenkamp, Janette Rahamat-Langendoen, Reina Sikkema. Marion Koopmans
EPI_ISL_l3251120	Laboratory of Viralogy, INMI Lazzara Spallanzani IRCCS	Laboratory of Viralogy, INMI Lazzara Spallanzani IRCCS	Giombini,E., Gruber,C.E.M., Rueca,M., Gramigna,G., Vlta,S., Carletti,F., D'Abramo,A., Lapa,□.,Pura,V., Fabeni,l., 8utera,O., Colavita,F., Meschi,S., Matusali,G., Specchiarello,E., Vaira,f., Vaia,F., Garbuglia,A.R., Nicastri,E., Antinori,A., Glrardi,E. and Maggi,F.
EPI_ISL_l3251157	checkin Zollhaus	Institute of Medical Virology, University of Zurich	Verena Kufner. Gabriela Ziltener, Maryam Zaheri, Stefan Schmutz, Annette Audigé, Odette Bernasconi. Kevin Steiner, Jan Huder, Cyril Shah, Riccarda Capaul, Guido Bloemberg, Jürg Bëni, Michael Huber, Alexandra Trkola
EPI_ISL_l3251584	Division of Infectious Diseases, University Hospital Zürich	Institute of Medical Virology, University of Zurich	Verena Kufner, Gabriela Ziltener, Maryam Zaheri, Stefan Schmutz, Annette Audigé, Odette 8ernasconi, Kevin Steiner, Jon Huder, Cyril Shah, Riccarda Capaul, Guido Bloemberg, Jürg Bëni, Michael Huber, Alexandra Trkola
EPI_ISL_l3251723	checkin Zollhaus	Institute of Medical Virology, University of Zurich	Verena Kufner. Gabriela Ziltener, Maryam Zaheri, Stefan Schmutz, Annette Audigé, Odette Bernasconi. Kevin Steiner, Jan Huder, Cyril Shah, Riccarda Capaul, Guido Bloemberg, Jürg Bëni, Michael Huber, Alexandra Trkola
EPI_ISL_l3269478	Alberta Precision Laboratories	Alberta Precision Laboratories	Matthew Craxen, Ashwin Deo, Paul Dieu, Xiaoli Dong, Kara Gill, David Granger, Christina Ferrato, Vanipriyadarsini Ikkurti, Jamil Kanji, Petya Koleva, Vincent Li, Colin Lloyd, Tarah Lynch, Raymond Ma, Kanti Pabbaraju, Silas Rotich, Hilary Sergeant, Steven Shideler, Todd Skitsko, Sandy Shokopoles, Graham Tipples, Johanna Thayer, Anita Wang
EPI_ISL_l3270980	Institut□ de Infectologia Emilio Ribas	Imperia! College London, School of Public Health	Claro,I.M.. de Lima,E.L., Romano,C.M., Candido.D.S., Lindoso,J.A.L., Barra,I.A.C., Borges.l.M.S.. Medeiros.L.A., Tomishige,M.Y.S., Ramundo,M.S., Moutinho,T., da Silva,A.J.O.. Rodrigues,C.C.M., de Azevedo.L.C.F.. Villas-Boas.L.S.. da Silva,C.A.M., Coletti,T.M., O'Toole,A., Quick,J., Loman,N., Rambaut.A., Faria,N.R., figueiredo-Mello,C. and Sabino,E.C.
EPI_ISL_l3302316	Laboratory of Clinical Microbiology, Virology and Bioemergencies. ASST-Fatebenefratelli-Sacco, L.Sacco University Hospital	Army Medical and Veterinary Research Center	Silvia Fillo, Riccardo De Sanctis, Giovanni Faggioni, Andrea Ciammaruconi, Anna Anselmo, Vanessa Vera Fain, Simone Di Sabatino, Francesco Giordani. Antonella Fortunato, Rossella Brandi. Giulia Campoli, Marzia Cavalli, Anella Monte, Martina Lipari, Maria Di Spirite, Giorgia Grilli, Silvia Chimienti, Giandomenico Cerreto, Filippo Molinari, Giancarlo Petralito, Davide Mileto, Valeria Micheli, Maria Rita Gismondo, Florigio Lista
EPI_ISL_l3304977	National Public Health Center, National Biosafety Laboratory	National Public Health Center, National Biosafety Laboratory	Judit Henczk6, Dâniel Oéri, Lili Jârmi, Bernadett Pâlyi, Zoltân Kis,
EPI_ISL_l3308117, EPI_ISL_l3308118, EPI_ISL_l3308119, EPI_ISL_l3308121, EPI_ISL_l3308122, EPI_ISL_l3308124, EPI_ISL_l3308125, EPI_ISL_l3308127, EPI_ISL_l3308129, EPI_ISL_l3308131, EPI_ISL_l3308133, EPI_ISL_l3308135, EPI_ISL_l3308137, EPI_ISL_l3308139, EPI_ISL_l3308140, EPI_ISL_l3308142, EPI_ISL_l3308144, EPI_ISL_l3308145, EPI_ISL_l3308146, EPI_ISL_l3308147, EPI_ISL_l3308148, EPI_ISL_l3308150, EPJ_ISL_l3308151, EPI_JSL_l3308153, EPI_ISL_l3308155, EPI_ISL_l3308157			
see above	Centre for Biological Threats, Highly Pathogenic Viruses, Robert Koch Institute	Centre for Biological Threats, Highly Pathogenic Viruses, Robert Koch Institute	Brinkmann,A.. Kohl.C., Uddin,S., Pape,K., Schrick,L., Michel,J., Schaade,I. and Nitsche,A.
EPI_JSL_l3308158, EPI_ISL_l3308160	IRBA Research Institute Biomédicale Des Armées	IRBA Research Institute Biomédicale Des Armées	Jarjaval,F., Nolent,F., Criqui,A., Chapus,C., Lamer,O., Ferraris,O. and Gorge,O.
EPJ ISL 13308162. EPI JSL 13308163, EPI-=-ISL-=-13308165, EPC_ISL-=-13308167	Laboratory for Diagnostics of Zoonoses and WHO Centre, Institute of Microbiology and Immunology, Faculty of Medicine, University of Ljubljana	Laboratory for Diagnostics of Zoonoses and WHO Centre, Institute of Microbiology and Immunology, faculty of Medicine, University of Ljubljana	Zakotnik,S., Vlaj,O.. Suljic.A., Zorec.T.M., Korva.M.. Poljak,M. and Avsic Zupanc,T
EPI_ISL_l3314740	Laboratorio de Vigilancia em Saude de Vinhedo	Instituto Adolfo Lutz Strategic Laboratory	Claudio Tavares Sacchi, Karoline Rodrigues Campos, Adriano Abbud, Adriana Bugna
EPI_ISL_l3331598	Department for Virology, Molecular Biology and Genome Research, R. G. Lugar Center for Public Health Research, National Center for Disease Contrai and Public Health (NCDC) of Georgia	Oepartment for Virology, Molecular Biology and Genome Research, R. G. Lugar Center for Public Health Research, National Center for Disease Contrai and Public Health (NCDC) of Georgia	Giorgi Tomashvili, Salome Javashvili, Meri Pantsulaia, Gvantsa Brachveli, Ana Papkiauri, Giorgi Gogoladze, Gvantsa Chanturia, Adam Kotorashvili, Maia Alkhazashvili, Khatuna Zakhashvili, Paata Imnadze, Amiran Gamkrelidze.
EPI_ISL_l3331712	Laboratory of Virology, INMI Lazzaro Spallanzani IRCCS	Laboratory of Virology, INMI Lazzaro Spallanzani IRCCS	Rueca,M., Giombini,E., Gruber,C.E.M., Gramigna,G., Mazzotta,V. Carletti,F , lapa,□.,Pittalis,S., Puro,V., Fabeni,L., Butera,O., Colavita,F., Meschi,S., Matusali,G , Specchiarello,E., Vairo,F., Vaia,F., Nicastri,E., Antinori,A., Girardi,E. and Maggi,F.
EPI_ISL_l3331713	Laboratory of Virology, INMI Lazzaro Spallanzani IRCCS	Laboratory of Virology, INMI Lazzaro Spallanzani IRCCS	Gramigna,G., Giombini,E., Gruber,C.E.M., Rueca,M., Carletti,F. Cicalini,S., Lapa,□., Pure.V., Marani,A., Fabeni,l., Butera,O., Colavita,F., Meschi,S., Matusali,G., Rivano Capparuccia,M., Specchiarello,E., Vairo,F., Vaia,F., Nicastri,E., Antinori,A.. Girardi.E. and Maggi,F.
EPI_ISL_l3331714	Department of Virology, Faculty of Medicine, University of Helsinki, Hartmaninkatu 3	Department of Viralogy, Faculty of Medicine, University of Helsinki, Hartmaninkatu 3	Kant,R., Smura,T., Vauhkonen,H. and Vapalahti,O.
EPI_ISL_l3331715	Department of Virology, Faculty of Medicine, University of Helsinki, Hartmaninkatu 3	Department of Virology, Faculty of Medicine, University of Helsinki, Hartmaninkatu 3	Kant,R.. Smura,T.. Vauhkonen,H., Vapalahti,O. and Siranen,T.
EPI_ISL_l3331716	Genomics Division, Instituto Tecnologico y de Energias Renovables (ITER). Poligono Industrial de Granadilla	Genomics Division, Instituto Tecnologico y de Energias Renovables (ITER), Poligono Industrial de Granadilla	Alcoba-Florez,J., Munoz-Barrera,A.. Ciuffreda,L., Rodriguez-Perez,H , Rubio-Rodriguez,L.A., Gil-Campesino,H., Garcia-Martinez de Artola,D., Inigo-Campos,A., Diez-Gil,O. Gonzalez-Montelongo,R., Valenzuela-Fernandez,A., Lorenzo-Salazar,J.M. and Flores,C.
EPI_ISL_l3331717	Genomics Division, Instituto Tecnologico y de Energias Renovables (ITER), Poligono Industrial de Granadilla	Genomics Division, Instituto Tecnologico y de Energias Renovables (ITER), Poligono Industrial de Granadilla,	Alcoba-Florez,J., Munoz-Barrera,A., Ciuffreda,L., Rodriguez-Perez,H , Rubio-Rodriguez,L.A., Gil-Campesino,H., Garcia-Martinez de Artola,D., Inigo-Campos,A., Diez-Gil,O. Gonzalez-Montelongo,R., Valenzuela-Fernandez,A., Lorenzo-Salazar,J.M. and Flores,C.
EPI_ISL_l3338028	Clinical Virology Unit, Department of Clinical Sciences, Institute of Tropical Medicine of Antwerp	Clinical Virology Unit. Oepartment of Clinical Sciences, Institute of Tropical Medicine of Antwerp	Antonio Mauro Rezende*, Tessa de Black*, Sandra Coppens, Eric florence, Maartje van Frankenhuijsen, Stefanie Bracke, Isabel Brosius, Laurens Liesenborghs, Patrick Soentjens, Kevin Ariën, Marjan Van Esbroeck, Philippe Selhorst , Koen Vercauteren* *equal contribution
EPI_ISL_l3339105	Microbiology Service, Hospital Universitario Clinico San Cecilia, Granada	Microbiology Service, Hospital Universitario Clinico San Cecilia, Granada	Chueca N, de Salazar A, Viriuela L, Fuentes A, Casimiro-Soriguer CS, Perez-Florido J. Dopazo J, Garcia F
EPI_ISL_l3342823	Clinical Viralogy Unit, Department of Clinical Sciences, Institute of Tropical Medicine of Antwerp	Clinical Virology Unit, Oepartment of Clinical Sciences, Institute of Tropical Medicine of Antwerp	Philippe Selhorst, Antonio Mauro Rezende, Tessa de Black, Sandra Coppens, Eric Florence, Isabel Brosius, Laurens Liesenborghs, Kevin Ariën, Marjan Van Esbroeck, Chris Kenyan, Koen Vercauteren
EPI_ISL_l3343634	Instituto de Infectologia Emilio Ribas	Instituto Adolfo Lutz Strategic Laboratory	Claudio Tavares Sacchi, Karoline Rodrigues Campos, Adriano Abbud, Adriana Bugna
EPI_ISL_l3343697	Fleury Medicina Oign6stica	Instituto Adolfo Lutz Strategic Laboratory	Claudio Tavares Sacchi, Karoline Rodrigues Campos, Adriano Abbud, Adriana Bugna

EPI_ISL_l3343718	Hospital Santa Ignes	Instituto Adolfo Lutz Strategic Laboratory	Claudio Tavares Sacchi, Karoline Rodrigues Campos, Adriano Abbud, Adriana Bugno
EPI_ISL_l3351002	B.C. Centre for Disease Contrai Public Health Laboratory	B.C. Centre for Disease Contrai Public Health Laboratory	John Tyson, Tracy Lee, Anthea Lam, Josh Quick, Agatha Jassem, Natalie Prystajecky, Linda Hoang, Inna Sekirov, Catherine Hogn, Frankie Tsang, Mel Krajden
EPI_ISL_l3362760, EPI_ISL_13362764	Laboratorio di Epidemiologia Molecolare e Sanità Pubblica-Policlinico Bari	Istituto Zooprofilattico Sperimentale della Puglia e della Basilicata	Parisi A, Simone D, Capozzi L. Del Sambro I, Bianco A, Chironna M, Loconsole D, Sallustio F, Galante D, Pace L. Manzulli V, Fasanella A.
EPI_ISL_l3363142	Hospital Universitari Vall d'Hebron	Hospital Universitari Vall d'Hebron	Maria Pir'iana, Cristina Andrés, Alejandra Gonztilez-Stinchez, Damir Garcia-Cehic, Ariadna Rando, Juliana Esperalba, Maria Gema Codina, Maria Carmen Martin, Carla Castillo, Karen Garcfa, Rodrigo Vtisquez, Maria Piquer, Tomàs Pumarola, Josep Quer, Andrés Ant6n
EPI_ISL_l3374487	National Public Health Center, National Biosafety Laboratory	National Public Health Center, National Biosafety Laboratory	Judit Henczk6, Dâniel Déri, Fruzsina Petrovay, Lili Jârmi, Bernadett Pâlyi, Eszter Balla, Zoltân Kis
EPI_ISL_13408797	Virology, Instituto Nacional de Enfermedades Infecciosas	Virology. Instituto Nacional de Enfermedades Infecciosas	Lewis,A., Josiowicz.A., Hirmas Riade,S.M., Tous,M. and Cisterna,D.M
EPI ISL 13408799, EPI ISL 13408801, - - EPI_ISL_l34Ü880-3	Public Health Agency of Canada, National Microbiology Laboratory	Public Health Agency of Canada, National Microbiology Laboratory	Knox,N.. Hole,D.. Duggan,A., Yadav,C.. Haidl,E., Chapel,M., Graham,M.. Domselaar,G.V., Jolly,G., Audet,J.. Fernando,L.. Antonation,K., Hagan,M., Griffiths,E., Leung,A.. Safronetz,D.. Eshaghi,A.. Gubbay,J.B., Hasso,M.. Marchand-Austin.A., Olsha,R. and Patel,5.N.
EPI_ISL_l3408805, EPI_ISL_l3408807, EPI_ISL_l3408809, EPI_ISL_l3408811, EPI_ISL_l3408813, EPI_ISL_l3408815, EPI_ISL_l3408817, EPI_ISL_l3408819, EPI_ISL_l3408821, EPI_ISL_l3408823, EPI_ISL_l3408825, EPI_ISL_l3408827, EPI_ISL_l3408829, EPI_ISL_l3408831, EPI_ISL_l3408833, EPI_ISL_l3408835			
see above	Public Health Agency of Canada, National Microbiology Laboratory	Public Health Agency of Canada, National Microbiology Laboratory	ncknox
EPI_ISL_l3408837, EPI_ISL_13408839, EPI_ISL_l3408841, EPI_ISL_13408843, EPI_ISL_l3408845, EPI_ISL_l3408847, EPI_ISL_l3408849, EPI_ISL_l3408851, EPI_ISL_13408853, EPI_ISL_13408855, EPI_ISL_l3408857, EPI_ISL_l3408859, EPI_ISL_13408861			
see above	Public Health Agency of Canada, National Microbiology Laboratory	Public Health Agency of Canada, National Microbiology Laboratory	Knox,N., Duggan,A., Yadav,C., Hole,D., Haidl,E., Chapel,M., Jolly,G., Domselaar,G.V., Antonation,K., Leung,A., Fernando,L, Audet,J., Hagan,M., Graham,M., Griffiths,E., Safronetz,D., Charest,H., Levade,I. and Fafard,J.
EPI ISL 13409177, EPI ISL 13409178, EPJ-ISL-13409179, EPI-JSL-13409180, - - EPI_ISL_l34Ü918-1	Viral Genomics and Bioinformatics, MRC University of Glasgow Centre for Virus Research	Viral Genomics and Bioinformatics, MRC University of Glasgow Centre for Virus Research	Filipe,A.. Tong,L.. Vattipally.S.B., Maclean,A., Gunson,R.. Holden,M.T.G.. Barr,D., Ho.A., Palmarini,M., Rambaut.A.. Robertson,D.L and Thomson,E.C.
EPI_ISL_l3411153, EPI_JSL_l3411154, EPI ISL 13411155, EPI ISL 13411156, EPI JSL l3411157, EPC_ISL l3411158	Centre for Biological Threats, Highly Pathogenic Viruses, Robert Koch Institute	Centre for Biological Threats, Highly Pathogenic Viruses, Robert Koch Institute	Brinkmann,A., Kohl,C., Uddin,S., Pape,K., Schrick,L., Michel,]., Schaade,L. and Nitsche,A.
EPJ ISL 13411159, EPI ISL 13411160, EPI JSL l3411161, EPC_ISL l3411162	Centre for Biological Threats, Highly Pathogenic Viruses, Robert Koch Institute	Centre for Biological Threats, Highly Pathogenic Viruses, Robert Koch Institute	Brinkmann,A., Kohl,C., Uddin,S., Pape,K., Schrick,L., Michel,]., Stocker,H., Schaade,L. and Nitsche,A.
EPJ ISL 13411163, EPI JSL 13411164, - - EPI_ISL_134l116-5	Centre for Biological Threats, Highly Pathogenic Viruses, Robert Koch Institute	Centre for Biological Threats, Highly Pathogenic Viruses, Robert Koch Institute	Brinkmann,A., Kohl,C.. Uddin,S., Pape,K., Schrick,L., Michel,]., Schaade,L. and Nitsche,A.
EPI_JSL_l3411166, EPI_ISL_l3411167	Centre for Biological Threats, Highly Pathogenic Viruses, Robert Koch Institute	Centre for Biological Threats, Highly Pathogenic Viruses, Robert Koch Institute	Brinkmann,A., Kohl,C., Uddin,S., Pape,K., Schrick,L., Michel,]., Jessen,H., Schaade,L. and Nitsche,A.
EPI_ISL_l3411168	Centre for Biological Threats, Highly Pathogenic Viruses, Robert Koch Institute	Centre for Biological Threats. Highly Pathogenic Viruses, Robert Koch Institute	Brinkmann,A.. Kohl,C.. Uddin,S., Pape,K.. Schrick,L., Michel,]., Pfaefflin,F.. Schaade,L. and Nitsche,A
EPI_ISL_13436658	Coordenadoria de Vigilancia em Saude - Sao Paulo	Instituto Adolfo Lutz Strategic Laboratory	Claudio Tavares Sacchi. Karoline Rodrigues Campos, Ariadne Fereira Amarante, Adriano Abbud, Adriana Bugno
EPI_ISL_13436792	Hospital Santa Ignes	Instituto Adolfo Lutz Strategic Laboratory	Claudio Tavares Sacchi, Karoline Rodrigues Campos, Adriano Abbud. Adriana Bugno
EPI_ISL_l3437056	Hosp. Alemao Oswaldo Cruz	Instituto Adolfo Lutz Strategic Laboratory	Claudio Tavares Sacchi, Karoline Rodrigues Campos, Ariadne Ferreira Amarante, Adriano Abbud, Adriana Bugno
EPI_ISL_13445553	Laboratory for Diagnostics of zoonoses and WHO Centre, Institute of Microbiology and Immunology, Faculty of Medicine, University of Ljubljana	Laboratory for Diagnostics of zoonoses and WHO Centre, Institute of Microbiology and Immunology. Faculty of Medicine, University of Ljubljana	Zakotnik,S., Vlaj.D., Suljic,A., zorec,T.M., Korva,M., Poljak,M. and Avsic Zupanc,T.
EPI_ISL_l3449965, EPI_ISL_l3449966	Hospital Universitario La Paz, Microbiology	Hospital Universitario La Paz, Microbiology	de la Hoz-Sanchez,B., Lopez-Ortiz,M., Gutierrez-Arroyo,A., Roces-Alvarez,P., Lazaro-Peona,F., Dahdouh,E., Bloise,I., Garcia-Rodriguez,J. and Mingorance,J.
EPI_ISL_13459346	CRT-DST-AIDS	Instituto Adolfo Lutz Strategic Laboratory	Claudio Tavares Sacchi, Karoline Rodrigues Campos, Ariadne Ferreira Amarante, Adriano Abbud, Adriana Bugno
EPI ISL 13459347, EPI ISL 13459482, - - EPI_ISL_l345948-3	Instituto de Infectologia Emilio Ribas	Instituto Adolfo Lutz Strategic Laboratory	Claudio Tavares Sacchi, Karoline Rodrigues Campos, Ariadne Ferreira Amarante, Adriano Abbud, Adriana Bugno
EPI_ISL_l3466447, EPI_ISL_l3466448, EPI_ISL_l3466449, EPI_ISL_l3466450, EPI_ISL_l3466451, EPI_ISL_l3466452, EPI_ISL_l3466453, EPI_ISL_l3466455, EPI_ISL_l3466456, EPI_ISL_l3466457, EPI_ISL_l3466458, EPI_ISL_13466459, EPI_ISL_l3466460, EPI_ISL_l3466461, EPI_ISL_l3466462, EPI_ISL_l3466463, EPI_ISL_l3466464, EPI_ISL_l3466465			
see above	Department of Infectious Diseases, National Institute of Health Doutor Ricardo Jorge, Portugal (INSA)	Department of Infectious Diseases, National Institute of Health Doutor Ricardo Jorge. Portugal (INSA)	Isidro,).. Borges.V., Pinto,M., Sabrai,□., Santos,)., Nunes,A., Mixao,v., Ferreira,R.. Santos,D., Duarte,S., Vieira,L., Borrego,M.J., Nuncio,S., Lopes de Carvalho,I., Pelerito,A., Cordeiro,R., Gomes,J.P.
EPI_ISL_l3472080	National Institute of Public Health NIH - NRI	National Institute of Public Health NIH - NRI	Wo/kowicz Tomasz, Zacharczuk Katarzyna, Gierczyr'lski Ratai
EPI_ISL_l3472250	Medical University of Vienna Center for Virology	Medical University of Vienna Center for Virology	Jeremy V. Camp, Monika Redlberger-Fritz, Stephan W. Aberle
EPI ISL 13483155, EPI ISL 13483157, EPÜSL)3483159, EPI=ISL)3483161, EPJ_ISL_13483162, EPI_ISL_13483163, EPI_ISL_13483164	Centre for Biological Threats, Highly Pathogenic Viruses, Robert Koch Institute	Centre for Biological Threats, Highly Pathogenic Viruses, Robert Koch Institute	Brinkmann,A., Kohl,C., Uddin,S., Pape,K., Schrick,I., Michel,)., Jessen,H., Schaade,L. and Nitsche,A.
EPI ISL 13483165, EPI ISL 13483167, EPJ ISL 13483168, EPI JSL 13483170, EPI ISL 13483171, EPI ISL 13483173, EPI ISL 13483175, EPI ISL 13483177, EPI ISL 13483178, EPI ISL 13483180, EPI ISL 13483182, EPI ISL 13483183, EPI ISL 13483185, EPI ISL 13483187, EPI ISL 13483188, EPI ISL 13483190, EPI ISL 13483191, EPI ISL 13483193, EPI ISL 13483195, EPI ISL 13483196, EPI=ISL)3483198, EPI=ISL)3483200, EPÜSL)3483201, EPI=ISL)3483203, EPI=ISL)3483205, EPI=ISL)3483206, EPI=ISL)3483208			
see above	Centre for Biological Threats, Highly Pathogenic Viruses. Robert Koch Institute	Centre for Biological Threats, Highly Pathogenic Viruses. Robert Koch Institute	Brinkmann,A., Kohl,C., Uddin,S., Pape,K., Schrick,L., Michel,]., Schaade,L. and Nitsche.A.
EPI_ISL_13484458	Laboratorio de Enterovirus, Instituto Oswaldo Cruz, Fiocruz	Instituto Oswaldo Cruz FIOCRUZ - Laboratory of Respiratory Viruses and Measles (LVRS)	Paola Resende, Elisa Cavalcante Pereira, Bruna Mendonça da Silva, Jéssica Graça Macedo de Carvalho, Larissa Macedo Pinto, Victor Guimaraes, Marilda Siqueira, Renan da Silva Faustino, Marilia Santini, Edson Elias da Silva on behalf of the Fiocruz Genomic Surveillance Network
EPI_ISL_l3498265	National Institute for Communicable Diseases of the National Health Laboratory Service	National Institute for Communicable Diseases of the National Health Laboratory Service	Chan WY, Mtshali PS, Grobbelaar A. Moolla N. Mohale T, Du Plessis MG. Ismail A. Weyer J
EPI_ISL_l3502582	Laboratory of Microbiology and Virology, Ospedale Amedeo di Savoia, ASL "Città di Torino"	Laboratory of Microbiology and Virology, Ospedale Amedeo di Savoia, ASL "Città di Torino"	Francesco Cerutti. Antonella Bottoni. Marisa Cazzadore. Tiziano Allice. Maria Grazia Milia. Gabriella Gregori, Elisa Burdino, Valeria Ghisetti
EPI_ISL_13508393	Hosp. Itacolomy Butanta	Instituto Adolfo Lutz Strategic Laboratory	Claudio Tavares Sacchi, Karoline Rodrigues Campos, Ariadne Ferreira Amarante, Adriano Abbud, Adriana Bugno
EPI_ISL_l3508471	Instituto de Infectologia Emilio Ribas	Instituto Adolfo Lutz Strategic Laboratory	Claudio Tavares Sacchi. Karoline Rodrigues Campos. Ariadne Ferreira Amarante, Adriano Abbud, Adriana Bugno
EPI_ISL_l3511312	Laboratorio de Salud Pl.Iblica de Antioquia	Instituto Nacional de Salud- Direcci6n de Investigaci6n en Salud Pl.Iblica	Katherine Laiton-Donato, Diego A. Alvarez-Dfaz, Carlos Franco-Mur'ioz, Héctor A. Ruiz-Moreno, Paola Rojas-Estevez, Andres Prada, Alicia Rosales, Marcela Mercado-Reyes
EPI_ISL_l3530881	Laboratorio de Referencia Nacional de Virus Respiratorios. Centra Nacional de Salud Publica. Instituto Nacional de Salud Peru.	Laboratorio de Referencia Nacional de Virus Respiratorios. Centra Nacional de Salud Publica. Instituto Nacional de Salud Peru.	Carlos Padilla Rojas. Veronica Hurtado Vela, Iris Silva Molina. Luren Sevilla Castaiieda, Victor Jimenez Vasquez, Orson Mestanza Millones, Luis Barcena Flores, Wendy Lizarraga Olivares. Alicia Nuiiez llanos, Steve Acedo Lazo, Francisco Ascue Orosco, Kelly Izarra Rojas, Princesa Medrano Alhuay, Karla Vasquez Cajachahua, Estela Huaman Angeles, Jorge Giralda Chavez, Lilian Huarca Balbin, Lisbet Roxana Inga Angulo, Maria Sandra Villar Saavedra, Henri Bailon Calderon, Lely Solari Zerpa, Gloria Arotinco Garayar. Equipa de vigilancia genomica del Instituto Nacional de Salud.
EPI_ISL_l3537922	Instituto de Medicina Tropical de Sao Paulo (IMT-USP)	School of Public Health, Imperial College London	Coletti,T.M., Ghilardi,F., khan,M.J., Claro,I.M., Valenca.LN., Faria,N.R. and Sabino,E.C.
EPI_ISL_l3537923	Microbiology, Immunology and Transplantation, KU Leuven, Rega Institute	Microbiology, Immunology and Transplantation, KU Leuven, Rega Institute	Wawina-Bokalanga,T., Vanmechelen,B., Logist,A.-5., Sinnesael,R., Ysebaert,I., Bloemen,M. and Maes,P.
EPI ISL 13537924, EPI ISL 13537925, - - EPI_ISL_l35379f6	Microbiology, Immunology and Transplantation, KU Leuven. Rega Institute	Microbiology, Immunology and Transplantation, KU Leuven, Rega Institute	Vanmechelen,B., Wawina-Bokalanga,T., Logist.A.-S., Sinnesael,R., Ysebaert,L., Verlinden,J., Van Holm,B., Bloemen,M. and Maes,P.
EPI_ISL_l3544223, EPI_ISL_l3544224, EPJ_ISL_13544225, EPI_ISL_l3544226, EPI_ISL_l3544227, EPI_ISL_l3544228, EPI_ISL_l3544229, EPI_ISL_13544230, EPI_ISL_l3544231, EPI_ISL_l3544232, EPI_ISL_l3544233, EPI_ISL_l3544234, EPI_ISL_13544235			
see above	Public Health Agency of Canada, National Microbiology Laboratory	Public Health Agency of Canada, National Microbiology Laboratory	Duggan,A., Hole,D., Knox,N., Yadav,C., Haidl,E., Chapel,M., Domselaar,G.V. Jolly,G., Audet,J., Fernando,L., Antonation,K., Safronetz,D., Hagan,M., Griffiths,E., Leung,A., Graham,M., Peters,G., Go.A., Laminman,v., Kaplen,B., Eshaghi,A., Gubbay,J.B., Hasso.M., Marchand-Austin.A., Olsha.R. and Patel.S.N.
EPI_ISL_l3544237, EPI_ISL_13544238, EPJ_ISL_13544239, EPI_JSL_13544240, EPI_ISL_13544241, EPI_ISL_13544243, EPI_ISL_13544244, EPI_ISL_13544245, EPI_ISL_13544246, EPI_ISL_13544247, EPI_ISL_13544248, EPI_ISL_13544249, EPI_ISL_13544250, EPI_ISL_l3544251, EPI_ISL_13544252, EPI_ISL_13544253. EPI_ISL_13544254, EPI_ISL_13544255, EPI_ISL_l3544256, EPI_ISL_13544257, EPI_ISL_l3544258. EPI_ISL_13544259, EPJ_ISL_13544260, EPI_ISL_13544261. EPI_ISL_13544262, EPI_ISL_13544263. EPI_ISL_13544264, EPI_ISL_13544265, EPI_ISL_13544266. EPI_ISL_13544267			
see above	Public Health Agency of Canada, National Microbiology Laboratory	Public Health Agency of Canada, National Microbiology Laboratory	Duggan,A., Hole,D., Knox,N., Yadav,C., Haidl,E., Chapel,M., Domselaar.G.V., Fernando,L., Graham,M., Antonation,K., Audet.J., Hagan,M., Safronetz,D., Leung,A., Peters,G., Go,A., Laminman,V., Kaplen,B., Jolly,G., Charest,H., Levade,I. and Fafard,J.
EPI_ISL_l3573943	Center for Virology, Medical University of Vienna	Medical University of Vienna Center for Virology	Jeremy V. Camp, Monika Redlberger-Fritz, Stephan W. Aberle
EPI_JSL_l3584854, EPI_ISL_13586184	Institute for Virology, Philipps-University Marburg	Institute for Virology, Philipps-University Marburg	Eickmann, M., Lier, C., Kowalski, K., Kraft, F., Becker, S.
EPI_ISL_l3607904	Servicio de Infectologia, Hospital Universitario Dr. José Eleuterio Gonzalez, Universidad Autonoma de Nuevo Leon	Centra de Investigacion e Innovacion en Virologia Medica, Departamento de Bioquimica y Medicina Molecular, Facultad de Medicina, Universidad Autonoma de Nuevo Leon	Kame A. Galan-Huerta, Manuel Paz Infanzon, Ali f. Ruiz Higareda, Laura Nuzzolo-Shihadeh, Adrian Camacho-Ortiz. Paola Bocanegra-Ibarias. Ana M. Rivas-Estilla. Daniel Zacarias-Villarreal. Luis A. Yamallel-Ortega, Maria D. Guerrero-Putz, Jorge Ocampo-Candiani
EPI_ISL_l3624509	Instituto de Diagn6stico y Referencia Epidemiol6gicos/Jurisdicci6n Sanitaria	Instituto de Diagn6stico y Referencia Epidemiol6gicos/Instituto de Biotecnologfa UNAM	Adnan Araiza-Rodriguez, Adriana Salvador-Patii'io, Alejandro Sanchez-Flores, América del Pilar Mandujano-Martfnez, Blanca Taboada, Carlos Eduardo Hernândez-Sânchez, Carlos f. Arias, Claudia Elena Wong-Arâmbula, Daniel José Regalado-Santiago, David Esal.l fragoso-Fonseca, Elizabeth Andrade-Montiel, Fabiola Garcés-Ayala, Fernando Gonztilez-Dominguez, Gabriel Garcfa-Rodrfguez, Gloria Vtizquez-Castro, Hugo L6pez Gatell Ramfrez, Irma L6pez-Martfnez,

	Cuauhtémoc/Hospital Ângeles Roma		Jerome Verleyen, Jesûs Trujillo, Jorge Ochoa, José Ernesto Ramfrez-Gonzâlez, Karel Estrada-Guerra, Luda Hernândez-Rivas, Magaly Guadalupe Landa-Flores, Maribel Gonzâlez-Villa, Mireya Mederos-Michel, Nancy Martfnez-Velâzquez, Noé Escobar-Escamilla, Oliva L6pez, Ricardo Cortés-Alcalâ, Ricardo Grande, Ver6nica Jiménez-Jacinto
EPI_ISL_l3632071	(enter of Diagnostics and Vaccine Development. Centers for Disease Control, Taiwan	(enter of Diagnostics and Vaccine Development, Centers for Disease Control, Taiwan	Jih-Hui Lin, Shu-Chun Chiu, Hsin-I, Huang, Wei-Lun Huang, Wen-Bin, Fann, Pei-Yu, Hsieh, Jyh-Yuan Yang
EPI_ISL_l3632288	National Institute for Communicable Diseases of the National Health Laboratory Service	National Institute for Communicable Diseases of the National Health Laboratory Service	Chan WY, Mtshali PS, Grabbelaar A, Moolla N, Mohale T, Lowe M, Du Plessis MG, Ismail A, Weyer J
EPI_ISL_l3651348, EPI_ISL_l3651349, EPI_ISL_l3651350	Laboratorio de Referencia Nacional de Virus Respiratorio. Centro Nacional de Salud Publica. Instituto Nacional de Salud.	Laboratorio de Referencia Nacional de Virus Respiratorio. Centra Nacional de Salud Publica. Instituto Nacional de Salud.	Carlos Padilla Rojas, Veronica Hurtado Vela, Iris Silva Molina, Luren Sevilla Castar'ieda, Victor Jimenez Vasquez, Orson Mestanza Millones, Luis Barcena Flores, Wendy Lizarraga Olivares, Alicia Nur'iez Llanos, Steve Acedo Lazo, Francisco Ascue Orosco, Kelly Izarra Rojas, Princesa Medrano Alhuay, Karla Vasquez Cajachahua, Estela Huaman Angeles, Jorge Giraldo Chavez, Lilian Huarca Balbin, Lisbet Roxana Inga Angulo, Maria Sandra Villar Saavedra, Henri Bailon Calderon, Lely Solari Zerpa, Gloria Arotinco Garayar. Equipo de vigilancia genomica del Instituto Nacional de Salud.
EPI_JSL_13658019, EPI_ISL_13658021	Erasmus Medical Center Department of Virology	Erasmus Medical Center Department of Virology	Bas Oude Munnink, Marjan Boter, Babette Weller, Richard Molenkamp, Janette Rahamat-Langendoen, Reina Sikkema, Marion Koopmans
EPI_ISL_l3660191	Hospital Center Luxembourg	Laboratoire National de Santé Microbiologie	Eric Hugoson, Ines Kozar, Sibel Berger, Anke Wienecke-Baldacchino, Bas Oude Munnink, Michel Kohnen, Jean-Hugues Francois, Tamir Abdelrahman
EPI_ISL_l3705358	Hosp. Alemao Dswaldo Cruz	Instituto Adolfo Lutz Strategic Laboratory	Claudio Tavares Sacchi, Karoline Rodrigues Campos, Ariadne Ferreira Amarante, Marion Benedito Nascimento Santos, Alex Domingos Reis, Adriano Abbud, Adriana Bugno
EPI_ISL_l3705407	Hosp. Sirio-Libanes	Instituto Adolfo Lutz Strategic Laboratory	Claudio Tavares Sacchi, Karoline Rodrigues Campos, Ariadne Ferreira Amarante, Marion Benedito Nascimento Santos, Alex Domingos Reis, Adriano Abbud, Adriana Bugno
EPI_ISL_l3717674	Hospital Center Luxembourg	Laboratoire National de Santé Microbiologie	Eric Hugoson, Ines Kozar, Sibel Berger, Anke Wienecke-Baldacchino, Bas Oude Munnink, Michel Kohnen, Jean-Hugues Francois, Tamir Abdelrahman
EPI_ISL_l3728303	Department of Medical Microbiology & Infection prevention, Amsterdam University Medical Centers location AMC	Department of Medical Microbiology & Infection prevention, Amsterdam University Medical Centers location AMC	Matthijs Welkers, Jelle Koopsen, Robin van Houdt, Marcel Jonges, Sebastien Matamoros, Sjoerd Rebers, Fokla zorgdrager, Sylvia Bruisten, Judith den Uil, Akke Cornelissen, Janke Schinkel, Menno de Jong, Gini van Rijckevorsel and Mariken van der Lubben on behalf of the Amsterdam Regional Genomic epidemiology and Outbreak Surveillance (ARGOS) consortium
EPI_ISL_l3732932	Hosp. Sao Joaquim - Beneficiencia Portuguesa	Instituto Adolfo Lutz Strategic Laboratory	Claudio Tavares Sacchi, Karoline Rodrigues Campos, Ariadne Ferreira Amarante, Marion Benedito Nascimento Santos, Alex Domingos Reis, Adriano Abbud, Adriana Bugno
EPI_ISL_l3734230, EPI_ISL_l3734231, EPI_ISL_l3734232	UK Health Security Agency	UK Health Security Agency	Atkinson,B., Pottage,T., Ngabo,D., Crook,A., Pitman,J., Summers,S., Pullan,S., Lewandowski,K., Furneaux,J., Davies,K. and Brooks,T.
EPI_ISL_l3734233	Microbial Genomics, Hospital General Universitario Gregorio Maranon	Microbial Genomics. Hospital General Universitario Gregorio Maranon	Palomino-Cabrera,R., Penas-Utrilla,D., Buenestado-Serrano,S., Perez-Lago,L, Herranz Martin,M., Veintimilla,C, Catalan,P., Munoz,P. and Garcia de Viedma.D.
EPI ISL 13734237, EPI ISL 13734236, EPI ISL 13734239, EPI ISL 13734240, EPI ISL 13734241. EPI ISL 13734242, EPI ISL 13734243, EPI ISL 13734244, EPI ISL 13734245, EPI ISL 13734246, EPI ISL 13734247, EPI ISL 13734248, EPI ISL 13734249, EPI ISL 13734250, EPI ISL 13734251. EPI ISL 13734252, EPI ISL 13734253, EPI ISL 13734254, EPI ISL 13734255, EPI ISL 13734256, EPI=ISL)3734257, EPI=ISL)3734258, EPÜSL)3734259, EPI=ISL)3734260, EPI=ISL)3734261, EPI=ISL)3734262, EPI=ISL)3734263, EPÜSL)3734264, EPI=ISL)3734265, EPI=ISL)3734266, EPI=ISL)3734267, EPI=ISL)3734268			
see above	Centre for Biological Threats, Highly Pathogenic Viruses, Robert Koch Institute	Centre for Biological Threats, Highly Pathogenic Viruses, Robert Koch Institute	Brinkmann,A., Kohl,C, Pape,K., Uddin,S., Schrick,L., Michel,]., Schaade,L. and Nitsche,A.
EPI_ISL_l3734269	Department of Clinical Sciences, Institute of Tropica Medicine	Department of Clinical Sciences, Institute ofTropica Medicine	De Baetselier,I., Van Dijck,C, Kenyon,C, Coppens,)., Smet,H., de Block,T., Coppens,S., Vanroye,F., Bugert,J., Girl,P., Liesenborghs,L., Selhorst,P., Arien,K., Van den Bossche,D., Florence,E., Rezende,A.M, Vercauteren,K. and Van Esbroeck,M.
EPI_ISL_l3734270	Centers for Disease Contrai & Prevention (CDC), Division of High Consequence Pathogens and Pathology (DHCPP-PRB)	Centers for Disease Contrai & Prevention {CDC). Division of High Consequence Pathogens and Pathology (DHCPP-PRB)	Gigante,C.M., Ventura,J., Seabolt,M.H, Zhao,H., Wilkins,K., Respress,J., Howard,□.,Batra,D., McCollum,A., Hutson,C, Davidson,W., Rao.A., Nash,J. and Li,Y.
EPI_ISL_l3744896	Centers for Disease Control & Prevention (CDC), Division of High Consequence Pathogens and Pathology (DHCPP-PRB)	Centers for Disease Contrai & Prevention {CDC). Division of High Consequence Pathogens and Pathology (DHCPP-PRB)	Gigante.CM., Ghinai,I., Seabolt.M.H., Zhao,H., Wilkins,K., Respress,J., Howard,□.,Batra,D., McCollum,A., Hutson,C, Davidson,W., Rao.A., Kerins,J. and Li.Y.
EPI_ISL_l3744897, EPI_ISL_l3744898	Centers for Disease Control & Prevention (CDC), Division of High Consequence Pathogens and Pathology (DHCPP-PRB)	Centers for Disease Contrai & Prevention {CDC). Division of High Consequence Pathogens and Pathology (DHCPP-PRB)	Gigante.CM., Hughes,S., Seabolt,M.H., Zhao,H., Wilkins,K., Respress.J., Howard,□.,Batra,D.. McCollum,A., Hutson,C, Davidson,W.. Rao.A.. Baumgartner,J. and Li,Y.
EPI_ISL_l3744899	Centers for Disease Contrai & Prevention (CDC), Division of High Consequence Pathogens and Pathology (DHCPP-PRB)	Centers for Disease Contrai & Prevention {CDC). Division of High Consequence Pathogens and Pathology (DHCPP-PRB)	Gigante.CM., Ghinai,I., Seabolt.M.H., Zhao,H., Wilkins,K., Respress,J., Howard,□., Batra,D., McCollum,A., Hutson,C, Davidson,W., Rao.A., Kerins,J. and Li,Y.
EPI_ISL_l3744900, EPI_ISL_l3744901	Centers for Disease Control & Prevention (CDC), Division of High Consequence Pathogens and Pathology (DHCPP-PRB)	Centers for Disease Contrai & Prevention {CDC). Division of High Consequence Pathogens and Pathology (DHCPP-PRB)	Gigante.CM., Hughes,S., Seabolt.M.H.. Zhao,H., Wilkins,K., Respress.J,, Howard,□.. Batra,D.. McCollum,A., Hutson,C, Davidson,W.. Rao.A.. Baumgartner,J. and Li,Y.
EPI_ISL_l3744902	Department of Virology, Faculty of Medicine, University of Helsinki	Department of Virology, Faculty of Medicine, University of Helsinki	Kant,R., Smura,T., Vauhkonen,H. and Vapalahti,O.
EPI_ISL_l3744903, EPI_ISL_l3744904, EPI_ISL_l3744905	Centre for Biological Threats, Highly Pathogenic Viruses, Robert Koch Institute	Centre for Biological Threats, Highly Pathogenic Viruses, Robert Koch Institute	Brinkmann,A., Kohl,C, Pape,K., Uddin,S., Schrick,L., Michel,]., Jessen,H., Schaade,L. and Nitsche,A.
EPI_ISL_l3744906, EPI_ISL_l3744907, EPI_ISL_l3744908, EPI_ISL_l3744909, EPI_ISL_l3744910, EPI_ISL_l3744911. EPI_ISL_l3744912, EPI_ISL_l3744913, EPI_ISL_l3744914. EPI_ISL_l3744915, EPI_ISL_l3744916. EPI_ISL_l3744917, EPI_ISL_l3744918. EPI_ISL_l3744919. EPI_ISL_l3744920, EPI_ISL_l3744921. EPI_ISL_l3744922, EPI_ISL_l3744923. EPI_ISL_l3744924, EPI_ISL_l3744925, EPI_ISL_l3744926, EPI_ISL_l3744927, EPI_ISL_l3744928, EPI_ISL_l3744929, EPI_ISL_l3744930, EPI_ISL_l3744931			
see above	Centre for Biological Threats, Highly Pathogenic Viruses, Robert Koch Institute	Centre for Biological Threats, Highly Pathogenic Viruses, Robert Koch Institute	Brinkmann,A., Kohl,C, Pape,K., Uddin,S., Schrick,L., Michel,]., Schaade,L. and Nitsche,A.
EPI_ISL_l3817808	New York University Langone Health	New York University Langone Health	Adriana Heguy, Dacia Dimartino, Emily Guzman, Christian Marier, Peter Meyn, Sitharam Ramaswami, Gael Westby, Paul Zappile, Yutong Zhang, Guiqing Wang
EPI ISL 13822667, EPI ISL 13822668, EP1-=.1sL-=.13822669, EPlisL-=.13822718	Erasmus Medical Center Department of Virology	Erasmus Medical Center Department of Virology	Bas Oude Munnink, Marjan Soter, Babette Weller, Richard Molenkamp, Janette Rahamat-Langendoen, Reina Sikkema, Marion Koopmans
EPI ISL 13827273, EPI ISL 13827274, EPÜSL)382727S, EPCISL)3827277, EPI ISL 13827278, EPI ISL 13827279, EPI-ISL-13827280, EPI-ISL-13827281, - - EPI_ISL_l38f728-2	Public Health Agency of Canada, National Microbiology Laboratory	Public Health Agency of Canada, National Microbiology Laboratory	Duggan.A., Hole,D., Yadav,C, Knox,N., Haidl,E., Chapel,M., Domselaar,G.V., Fernando,L., Graham,M., Antonation,K., Audet.J., Hagan,M., Safronetz,D., Leung,A., Peters,G., Go.A., Laminman,v., Kaplen,B., Jolly,G., Marchand-Austin,A., EshaghiA, Patel,S.N.. Hasso,M.. Gubbay,J.B. and Dlsha,R.
EPI_ISL_l3827283	Military Heatlh Institute in Prague, Military Heatlh Institute	Military Heatlh Institute in Prague. Military Heatlh Institute	Chmel,M., Pajer,P.. Nagy.A., Zlamal.M.. Jirincova,H.. Dresler,J. and Bartos,O.
EPJ ISL 13833194, EPI JSL 13833195, eP1-=.1sL-=.Bs33196, EPlisL-=.13833197	Laboratorio de Referencia Nacional de Virus Respiratorio. Centro Nacional de Salud Publica. Instituto Nacional de Salud	Laboratorio de Referencia Nacional de Virus Respiratorio. Centro Nacional de Salud Publica. Instituto Nacional de Salud.	Carlos Padilla Rojas, Veronica Hurtado Vela. Iris Silva Molina. Luren Sevilla Castarïeda, Victor Jimenez Vasquez, Orson Mestanza Millones, Luis Barcena Flores, Wendy lizarraga Olivares. Alicia Nurïez Llanos, Steve Acedo Lazo, Francisco Ascue Orosco, Kelly Izarra Rojas, Princesa Medrano Alhuay, Karla Vasquez Cajachahua, Estela Huaman Angeles, Jorge Giraldo Chavez, Lilian Huarca Balbin, Lisbet Roxana Inga Angulo, Maria Sandra Villar Saavedra, Henri Bailon Calderon, Lely Solari Zerpa, Gloria Arotinco Garayar. Equipa de vigilancia genomica del Instituto Nacional de Salud.
EPI_ISL_l3842269, EPI_ISL_l3842548	Center for Virology, Medical University of Vienna	Medical University of Vienna Center for Virology	Jeremy V. Camp, Monika Redlberger-Fritz, Stephan W. Aberle
EPI ISL 13889435, EPI ISL 13889436, EPI ISL 13889437, EPI ISL 13889438, EPI ISL 13889439, EPI ISL 13889440, EPI ISL 13889441, EPI ISL 13889442, EPI ISL 13889443, EPI ISL 13889444, EPI ISL 13889445, EPI ISL 13889446, EPI ISL 13889447, EPI ISL 13889448, EPI ISL 13889449, EPI ISL 13889450, EPI ISL 13889515, EPI ISL 13889590, EPI ISL 13889660, EPI ISL 13889729, EPI=ISL)3889796, EPI=ISL)3889908, EPÜSL)3889977, EPI=JSL)3890048, EPI=ISL)389013S, EPI=ISL)3890204, EPI=ISL)3890273, EPÜSL)3890338, EPI=ISL)3890408, EPI=ISL)3890464, EPI=ISL)3890465, EPI=ISL)3890466, EPÜSL)3890467, EPI=ISL)3890468, EPI=ISL)3890469, EPI=ISL)3890470, EPI=ISL)3890471, EPÜSL)3890472, EPI=ISL)3890473, EPI=ISL)3890474, EPI_ISL_l3890475, EPI_ISL_l3890476, EPI_ISL_l3890477, EPI_ISL_l3890478, EPI_ISL_l3890479, EPI_ISL_l3890480, EPI_ISL_l3890481, EPI_ISL_l3890482			
see above	Charité Universitatsmedizin Berlin, Institut für Virologie/Laber Berlin	Charité Universitatsmedizin Berlin. Institut für Virologie	Terry C Jones. Julia Schneider. Barbara Mühlemann, Talitha Veith, Jt:irn Beheim-Schwarzbach, Julia Tesch. Marie Luisa Schmidt, Felix Walper. Tobias Bleicker. Caroline Isner. Frieder Pfi:ifflin, Ricardo Niklas Werner, Victor M. Corman. Christian Drosten
EPI_ISL_l3891126	Ministry of Health Turkey	Ministry of Health Turkey	Fatma Bayrakdar, Suleyman Yalcin, Gulay Korukluoglu
EPI_ISL_l3908328	Center of Diagnostics and Vaccine Development. Centers for Disease Control	Center of Diagnostics and Vaccine Development, Centers for Disease Contrai	lin.J.-H., Chiu.5.-C, Huang,H.-1., Huang,W.-L. Fann,W.-B., Hsieh,P.-Y., Hsu.5.-C, Liu.P.-C., Chang,T.-Y. and Yang,J.-Y.
EPI_ISL_l3908329, EPI_ISL_l3908332, EPI_ISL_l3908333, EPI_ISL_l3908334, EPI_ISL_l3908335, EPI_ISL_l3908336, EPI_ISL_l3908337, EPI_ISL_l3908338, EPI_ISL_l3908339, EPI_ISL_l3908340, EPI_ISL_l3908341. EPI_ISL_l3908342, EPI_ISL_l3908343, EPI_ISL_l3908345			
see above	Public Health Agency of Canada, National Microbiology Laboratory	Public Health Agency of Canada, National Microbiolo9y Laboratory	Duggan,A., Hole,D., Yadav,C.. Knox,N., Chapel,M., Tyler.A.. Haidl,E., Domselaar.G.V.. Antonation.K., Audet.J., Fernando,L., Ha9an,M., Safronetz,D . Graham.M.. Peters.G., Go.A., Laminman,V., Kaplen,B.. Leung,A., Jolly,G.. Fafard,J., Charest,H. and Levade,I.
EPI ISL 13908346, EPI ISL 13908347, eP1-=.1sL-=.1390S348, EPl1sL-=.139os349	Centre for Biological Threats, Highly Pathogenic Viruses, Robert Koch Institute	Centre for Biological Threats, Highly Pathogenic Viruses, Robert Koch Institute	Brinkmann,A., Kohl,C, Pape,K., Uddin,S.. Schrick,L., Michel,].. Jessen,H., Schaade,L. and Nitsche,A.
EPI_ISL_l3908350, EPI_ISL_l3908351, EPI_ISL_l3908352, EPI_ISL_l3908353, EPI_ISL_l3908354, EPI_ISL_l3908355, EPI_ISL_l3908356, EPI_ISL_l3908357, EPI_ISL_l3908358, EPI_ISL_l3908359, EPI_ISL_l3908360, EPI_ISL_l3908361, EPI_ISL_l3908362, EPI_ISL_l3908363, EPI_ISL_l3908364, EPI_ISL_l3908365			
see above	Centre for Biological Threats, Highly Pathogenic Viruses, Robert Koch Institute	Centre for Biological Threats. Highly Pathogenic Viruses, Robert Koch Institute	Brinkmann,A.. Kohl.C., Pape,K., Uddin.S., Schrick.L.. Michel.].. Schaade,L. and Nitsche,A.
EPI_ISL_l3953610, EPI_ISL_l3953611	Indian Council of Medical Research-National Institute of Virology	Indian Council of Medical Research-National Institute of Virology	Pragya Yadav, Rima Sahay, Anita Aich Shete, Sreelekshmy Mohandas, Priya Abraham
EPI_ISL_l3955501	Public Health Authority of the Slovak Republic	Laboratory of Genomics and Bioinformatics, Comenius University Science Park	Tomas Szemes, Edita Staroi'lové, Elena Tiché, Lucia SevCiková, Terézia Vrabrova, Tatiana Sedléckové, Miroslav Bôhmer, Jaroslav BudiS, Pavai MiSenko
EPI_ISL_l3958697	Research and Evaluation, UKHSA	Research and Evaluation, UKHSA	Groves,N., Osman,K.L., Lewandowski,K.S., Carter,D.P., Pullan,S.T., Myers,R., Vipond,R. and Chand,M.
EPI_ISL_13983354, EPI_ISL_13983355	Instituto de Infectologia Emilio Ribas	Instituto Adolfo Lutz Strategic Laboratory	Claudio Tavares Sacchi, Karoline Rodrigues Campos, Ariadne Ferreira Amarante, Marion Benedito Nascimento Santos, Alex Domingos Reis, Adriano Abbud, Adriana Bugna
EPI_ISL_l3983356	INSPI-Centro de Referencia Nacional de Virus Exantemâticos, Gastroentéricos y Transmitido por Vectores.	INSPI-Direcci6n Técnica de Investigaci6n, Desarrollo e Innovaci6n INSPI-Centro de Referencia Nacional de Gen6mica, Secuenciaci6n y Bioinformîltica	Andrés Carrazco-Montalvo, Diana Gutiérrez, Naomi Mora, Silvia Salgado-Cisneros, Johana Parrales-Valdiviezo, Martha Sânchez-Domenech, Diego Morales, Gulnara Borja-Cabrera, Leandro Patifio*

EPI_ISL_13983888	Bangkok Hospital Phuket	National Institute of Health, Department of Medical Sciences, Ministry of Public Health, Thailand	Pilailuk Okada: Siripaporn Phuygun; Nuttida Thon9pramul; Thanutsapa Thanadachakul: Kazuhisa Okada; Archawin Rojanawiwat; Chakkarat Pitayawon9anon; Supakit Sirilak
EPJ ISL 13993734, EPI ISL 13993735, EPJ-ISL-13993737, EPI-JSL-13993738, - - EPI_ISL_139g"373-9	California Department of Public Health	California Department of Public Health	Viral and Rickettsial Disease Laboratory
EPI_ISL_14003930	University of Rochester Medical Center	University of Rochester Medical Center	Andrew Cameron, Mondraya Howard, Sara Connelly, Dwight Hardy, Kelly Delary
EPI_ISL_14011193	Bangkok Hospital Phuket	Thai Red Cross Emerging Infectious Diseases Clinical Center and Faculty of Medicine, Chulalongkorn University	Kusak Kukiattikoon, Waritta Dararattanaroj. Rome Buathong, Supaporn Wacharapluesadee. Sininat Petcharat, Ananporn Supataragul, Stefan Fernandez, Achawin Rojanaviwat. Chonticha Klungthong, Pilailuk Okada, Khajohn Joonlasak, Chakkarat Pitayawonganon, Opass Putcharoen
EPI_ISL_14021725	Hosp. Municipal Enf. Antonio Policarpo de Oliveira	Instituto Adolfo Lutz Strategic Laboratory	Claudio Tavares Sacchi, Karoline Rodrigues Campos, Ariadne Ferreira Amarante, Marion Benedito Nascimento Santos, Alex Domin9os Reis, Adriano Abbud, Adriana Bugna
EPI_ISL_14033203	Animal Health, Istituto Zooprofilattico Sperimentale del Mezzogiorno	Animal Health, Istituto Zooprofilattico Sperimentale del Mezzogiorno	Viscardi,M., Cardillo,L., De Martinis,C., Cozzolino,L. and Fusco,G.
EPJ ISL 14033204, EPI JSL 14033205, EPÜSL)4033206, EPCISL)4033207, EPI ISL 14033208, EPI ISL 14033209, EPI=ISL=14033210, EPI=JSL)4033211, EPI_ISL_14033212, EPI_ISL_14033213	Laboratory Medicine, UW Virolo9y	Laboratory Medicine, UW Virology	Sereewit,J., Xie,H., Pavitra,R. and Greninger,A.
EPI_ISL_14049244, EPI_ISL_14049245	Indian Council of Medical Research-National 1nstitute of Virology	Indian Council of Medical Research-National Institute of Virology	Pragya Yadav, Rima Sahay, Anita Aich Shete, Sreelekshmy Mohandas, Priya Abraham
EPJ ISL 14050451, EPI JSL 14050453, EPJ-ISL-14050454, EPI-ISL-14050455, EP1-=_1sL-=_14oso456, EPIisL-=_14050453	Public Health Agency of Canada, National Microbiology Laboratory	Public Health Agency of Canada, National Microbiology Laboratory	Duggan,A., Hole,D., Yadav,C., Knox,N., Tyler.A., Haidl,E., Chapel,M., Domselaar,G.V., Graham,M , Audet,J., Fernando,L., Hagan,M., Safronetz,D., Leung,A., Peters,G., Go.A., Laminman,V., Kaplen,B., Antonation,K., Jolly,G., Griffiths,E., Charest,H., Levade,I. and Fafard,J.
EPI_ISL_14056410	Azienda Sanitaria dell'Alto Adige - Laboratorio Aziendale di Microbiologia e Virologia	Azienda Sanitaria dell'Alto Adige - Laboratorio Aziendale di Microbiologia e Virologia	Teresa Fortini, Elisabetta Incrocci, Elisabetta Giacobazzi, Elisa Masi, Irene Bianconi, Elisabetta Pagani
EPI_ISL_l4070493, EPI_JSL_l4070852, EPI_ISL_14070854, EPI_ISL_14070855	Instituto de Infectologia Emilio Ribas	Instituto Adolfo Lutz Strategic Laboratory	Claudio Tavares Sacchi, Karoline Rodrigues Campos, Ariadne Ferreira Amarante, Marion Benedito Nascimento Santos, Alex Domingos Reis, Adriano Abbud, Adriana Bugna
EPI_ISL_14089382	Pathogen Genomics Lab, National Institute for Biomedical Research (INRB)	Pathogen Genomics Lab. National Institute for Biomedical Research (INRB)	Placide Mbala-Kingebeni, Eddy Kinganda-Lusamaki, Adrienne Amuri-Aziza, Elisabeth Pukuta, Catherine Pratt, Nicolas Fernandez, Emmanuel Lokilo Lofiko, Gradi Luakanda Ndelemo, Francisca Muyembe Mawete, Jean Claude Makangara Cigolo, Elisabeth Muyamuna, Raphaël Lumembe Numbi, Gabriel Kabamba Lungenyi, Prince Akil Sandali, Pauline Musuamba Kayembe, Rilia Ola Mpumbe, Emile Malembi, Emmanuel Hasivirwe Vakaniaki, Andrew Rambaut, Nick Loman, Kristian Andersen, Michael Wiley, Ahidjo Ayouba, Steve Ahuka-Mundeke, Martine Peeters, Eric Delaporte, Jean-Jacques Muyembe Tamfum
EPI_ISL_14153982	Vajira Hospital	National Institute of Health, Department of Medical Sciences, Ministry of Public Health, Thailand	Pilailuk Okada: Siripaporn Phuygun; Nuttida Thongpramul; Thanutsapa Thanadachakul: Kazuhisa Okada; Archawin Rojanawiwat; Chakkarat Pitayawonganon; Supakit Sirilak
EPI_ISL_l4166709	Medical University of Vienna Center for Virology	Medical University of Vienna Center for Virology	Jeremy V Camp, Monika Redlberger-Fritz, Stephan W. Aberle
EPJ ISL 14167248, EPI JSL 14167573, EPC.1sL-=_14151s14, EPC.1sL-=_14151s1s	Medical University of Vienna Center for Virology	Medical University of Vienna Center for Virology	Jeremy V. Camp, Monika Redlberger-Fritz, Stephan W. Aberle
EPI_ISL_l4170200, EPI_ISL_l4170201, EPI_ISL_14170203, EPI_ISL_14170204	Erasmus Medical Center Department of Virology	Erasmus Medical Center Oepartment of Virology	Bas Oude Munnink, Marjan Boter, Babette Weller, Babs Verstrepen, Richard Molenkamp, Janette Rahamat-Langendoen, Reina Sikkema, Marion Koopmans
EPI_ISL_l4181948. EPI_ISL_l4181949, EPJ_ISL_14181950, EPI_ISL_l4181951, EPI_ISL_l4181952, EPI_ISL_l4181953. EPI_ISL_l4181954, EPI_ISL_l4181955, EPI_ISL_l4181956, EPI_ISL_l4181957, EPI_ISL_l4181958. EPI_ISL_l4181959, EPI_ISL_l4181960			
see above	Institute of Health Carlos m, Bioinformatics Unit	Institute of Health Carlos 111, Bioinformatics Unit	Cuesta,I.
EPJ ISL 14189012, EPI JSL 14189013, EPÜSL)4189014, EPI=ISL)4189015, EPI ISL 14189016, EPI ISL 14189017, EP1-=_1sL-=_14189018, EPC.1sL-=_14139019	Los Angeles County Public Health Laboratories	Los Angeles County Public Health Laboratories	P. Hemarajata et al.
EPJ ISL 14201640, EPI JSL 14201641, EPÜSL=l4201642, EPI=ISL)4201643, EPI_JSL_14201644, EPI_ISL_14201645	Pathogen Genomics Lab, National Institute for Biomedical Research (INRB)	Pathogen Genomics Lab, National Institute for Biomedical Research (INRB)	Placide Mbala-Kingebeni, Eddy Kinganda-Lusamaki, Adrienne Amuri-Aziza, Elisabeth Pukuta, Catherine Pratt, Nicolas Fernandez, Emmanuel Lokilo Lofiko, Gradi Luakanda Ndelemo, Francisca Muyembe Mawete, Jean Claude Makangara Cigolo, Elisabeth Muyamuna, Raphaël Lumembe Numbi, Gabriel Kabamba Lungenyi, Prince Akil Sandali, Pauline Musuamba Kayembe, Rilia Ola Mpumbe, Emile Malembi, Emmanuel Hasivirwe Vakaniaki, Andrew Rambaut, Nick Leman, Kristian Andersen, Michael Wiley, Ahidjo Ayouba, Steve Ahuka-Mundeke, Martine Peeters, Eric Delaporte, Jean-Jacques Muyembe Tamfum
EPI_ISL_l4207724. EPI_ISL_14207725, EPI_ISL_l4207726, EPI_ISL_14207727, EPI_ISL_14207728, EPI_ISL_l4207729. EPI_ISL_14207730, EPI_ISL_l4207731. EPI_ISL_14207732. EPI_ISL_l4207733, EPI_ISL_l4207734. EPI_ISL_14207735, EPI_ISL_l4207736, EPI_ISL_l4207737. EPI_ISL_14207738, EPI_ISL_l4207739. EPI_ISL_14207740, EPI_ISL_l4207741			
see above	Laboratorio de Referencia Nacional de Virus Respiratorio. Centro Nacional de Salud Publica. Instituto Nacional de Salud.	Laboratorio de Referencia Nacional de Virus Respiratorio. Centra Nacional de Salud Publica Instituto Nacional de Salud.	Carlos Padilla Rojas, Veronica Hurtado Vela, Iris Silva Molina, Luren Sevilla Castai'ieda, Victor Jimenez Vasquez, Orsan Mestanza Millones, Luis Barcena Flores, Wendy Lizarraga Olivares, Alicia Nui'iez Llanos, Steve Acedo Lazo, Francisco Ascue Orosco, Kelly lzarra Rojas, Princesa Medrano Alhuay, Karla Vasquez Cajachahua, Estela Huaman Angeles, Jorge Giralda Chavez, Lilian Huarca Balbin, Lisbet Roxana Inga Angulo, Maria Sandra Villar Saavedra, Henri Bailon Calderon, Lely Solari zerpa, Gloria Arotinco Garayar. Equipe de vigilancia genomica del Instituto Nacional de Salud.
EPI_JSL_14211644, EPI_ISL_14211645	Public Health Authority of the Slovak Republic	Laboratory of Genomics and Bioinformatics, Comenius University Science Park	Tomâs Szemes, Edita Staroflovâ, Elena Tichâ, Lucia Sevcfkovâ, Terézia Vrabrovâ, Tatiana Sedlâckovâ, Miroslav Bôhmer, Jaroslav BudiS, Pavol MiSenko
EPI_ISL_l4216746, EPI_ISL_14216748, EPJ_ISL_l4216750, EPI_ISL_14216752, EPI_ISL_l4216753, EPI_ISL_14216754, EPI_ISL_14216755, EPI_ISL_l4216756, EPI_ISL_14216757, EPI_ISL_l4216758, EPI_ISL_14216759, EPI_ISL_14216761, EPI_ISL_14216762, EPI_ISL_14216764, EPI_ISL_l4216767, EPI_ISL_14216769			
see above	Laboratory Medicine, UW Virology	Laboratory Medicine, UW Virology	Sereewit,J., Xie,H., Roychoudhury,P. and Greninger,A.
EPI_ISL_l4224334	Genetica Molecular and Subdepartamento de Virologia ISP (hile	Instituto de Salud Publica de Chile	Paulo C. Covarrubias, Andrés E. Castillo, Constanza Campana, Mariela Guajardo, Bilrbara Parra, Rodrigo Fasce Pineda, Jorge Fernilndez
EPI_JSL_14241409, EPI_ISL_l4241410	Department of Clinical Sciences, Institute of Tropica Medicine	Department of Clinical Sciences, Institute ofTropica Medicine	De Baetselier,I., Van DiJck,C., Kenyon,C. Coppens,)., Smet,H., de Block,T., Coppens,S., Vanroye,F., Bugert,J., Girl,P., Liesenborghs,L., Selhorst,P., Arien,K., den Bossche,D.V., Florence,E., Rezende,A.M , Vercauteren,K. and Esbroeck,M.V.
EPI_ISL_14241411	Department of Clinical Sciences, Institute of Tropica Medicine	Department of Clinical Sciences, Institute ofTropica Medicine	Rezende,A.M., de Block,T., Coppens,S., Florence,E., Bracke,S., Brosius,I., Liesenborghs,L., Soentjens,P., Arien,K., Esbroeck,M.V., Selhorst,P. and Vercauteren,K.
EPJ ISL 14241412, EPI ISL 14241413, EPLJsL-=_14241414, EPC.1sL-=_1424141s	Department of Clinical Sciences, Institute of Tropica Medicine	Department of Clinical Sciences, Institute ofTropica Medicine	De Baetselier,I., Van Dijck,C., Kenyon,C., Coppens,)., Smet,H., de Block,T., Coppens,S., Vanroye,F., Bugert,J., Girl,P., Liesenborghs,L., Selhorst,P., Arien,K., den Bossche,D.V., Florence,E., Rezende,A.M , Vercauteren,K. and Esbroeck,M.V.
EPI_ISL_14244555	Centers for Disease Contrai & Prevention (CDC). Division of High Consequence Pathogens and Pathology (DHCPP-PRB)	Centers for Disease Contrai & Prevention (CDC). Division of High Consequence Pathogens and Pathology (DHCPP-PRB)	Gigante.C.. Ventura.J.. Seabolt,M.H., Zhao,H.. Wilkins.K., McCollum,A., Hutson.C.. Davidson.W.. Rao.A.. Nash.J. and Li,Y.
EPI_ISL_14244556	Centers for Disease Contrai & Prevention (CDC), Division of High Consequence Pathogens and Pathology (DHCPP-PRB)	Centers for Disease Contrai & Prevention {CDC). Division of High Consequence Pathogens and Pathology (DHCPP-PRB)	Gigante,C., Ventura,J., Seabolt,M.H., Zhao,H., Wilkins,K., McCollum,A., Hutson,C., Davidson,W., Rao.A., Nash,J., Sheth,M. and Li,Y.
EPI_ISL_14244557	Centers for Disease Contrai & Prevention (CDC), Division of High Consequence Pathogens and Pathology (DHCPP-PRB)	Centers for Disease Contrai & Prevention {CDC). Division of High Consequence Pathogens and Pathology (DHCPP-PRB)	Gigante,C., Xia,D., Seabolt,M., Zhao,H., Wilkins.K., McCollum,A., Hutson,C., Davidson,W., Rao.A., Plipat,N. and Li.Y.
EPI_ISL_14244558	Centers for Disease Contrai & Prevention (CDC), Division of High Consequence Pathogens and Pathology (DHCPP-PRB)	Centers for Disease Contrai & Prevention {CDC). Division of High Consequence Pathogens and Pathology (DHCPP-PRB)	Gigante,C., Goldoft,M., Seabolt,M., Zhao,H., Wilkins,K., McCollum,A., Hutson,C., Davidson,W., Rao.A., Holshue.M. and Li,Y.
EPI_ISL_14244559	Centers for Disease Contrai & Prevention (CDC), Division of High Consequence Pathogens and Pathology (DHCPP-PRB)	Centers for Disease Contrai & Prevention (CDC). Division of High Consequence Pathogens and Pathology (DHCPP-PRB)	Gigante,C., Pavlick,J., Seabolt,M., Zhao,H., Wilkins,K., McCollum,A., Hutson,C., Davidson,W., Rao.A., Parrott,T. and Li,Y.
EPI_ISL_14251112	University of Rochester Medical Center	University of Rochester Medical Center	Andrew Cameron, Mondraya Howard. Joel Maki, Sara Connelly, Kelly Delary. Dwight Hardy
EPI_ISL_l4254435, EPI_ISL_14254436, EPI_ISL_14254437, EPI_ISL_l4254438	Erasmus Medical Center Department of Virology	Erasmus Medical Center Department of Virology	Bas Oude Munnink, Marjan Soter, Babette Weller, Babs Verstrepen, Richard Molenkamp, Janette Rahamat-Langendoen. Reina Sikkema, Marion Koopmans
EPI_ISL_l4259830	Virology Unit, Azienda Ospedaliero-Universitaria Pisana	Virology Unit. Azienda Ospedaliero-Universitaria Pisana	Vatteroni.M. and Frateschi,S.
EPI_ISL_l4295679	Bangkok Hospital Phuket	National Institute of Health, Department of Medical Sciences, Ministry of Public Health, Thailand	Pilailuk Okada: Siripaporn Phuygun; Nuttida Thongpramul; Thanutsapa Thanadachakul; Kazuhisa Okada; Archawin Rojanawiwat; Chakkarat Pitayawonganon; Supakit Sirilak
EPI_ISL_14315314, EPI_ISL_14315315, EPJ_ISL_l4315316, EPI_JSL_14315317, EPI_ISL_l4315318, EPI_ISL_14315319, EPI_ISL_l4315320, EPI_ISL_l4315321, EPI_ISL_14315322, EPI_ISL_14315323, EPI_ISL_14315324			
see above	Laboratory Medicine, UW Virology	Laboratory Medicine, UW Virology	Sereewit.J., Xie,H., Roychoudhury,P. and Greninger,A.L.
EPJ_ISL_l4326638, EPI_ISL_14326639, EPI ISL 14326640, EPI JSL 14326641, EP1-=_1sL-=_14326642, EPIisL-=_14326643	Environmental, Agricultural, and Occupational Health, University of Nebraska Medical Center, 984388 Nebraska Medical Center	Enviranmental, Agricultural, and Occupational Health, University of Nebraska Medical Center, 984388 Nebraska Medical Center	Tegomoh.B., Cross,S.T.. Chapman,R.C., Bernhard.K., McCutchen,E.L., Fauver,J.R., Pratt,C.B., Warden,D.E, Iwen,P.C., Oonahue,M. and Wiley.M.R.
EPI_ISL_14326644	Environmental, Agricultural, and Occupational Health, University of Nebraska Medical Center, 984388 Nebraska Medical Center	Environmental, Agricultural, and Occupational Health, University of Nebraska Medical Center, 984388 Nebraska Medical Center	Tegomoh,B., Cross,S.T., Chapman,R.C., Bernhard,K., McCutchen,E.L., Fauver,J.R., Pratt.C.B., Warden,D.E., Iwen,P.C., Donahue,M. and Wiley,M.R

EPI ISL 14355206, EPI ISL 14355207, EPI=ISL=I4355208, EPI=JSL=I4355209, EPI_ISL_I4355210, EPI_ISL_I4355211	Laboratory Medicine, UW Virology	Laboratory Medicine, UW Virology	Sereewit,J., Xie,H., Roychoudhury,P. and Greninger,A.L.
EPI_ISL_I4362272	Centers for Disease Contrai & Prevention (CDC), Division of High Consequence Pathogens and Pathology (DHCPP-PRB)	Centers for Disease Contrai & Prevention {CDC). Division of High Consequence Pathogens and Pathology (DHCPP-PRB)	Gigante,C.M., Kubin,G., Seabolt,M.H., Zhao,H., Wilkins,K., McCollum,A., Hutson,C.. Davidson,W., Rao.A., White,S.L. and Li,Y.
EPI_JSL_14362274, EPI_ISL_I4362276	Centers for Disease Contrai & Prevention (CDC), Division of High Consequence Pathogens and Pathology (DHCPP-PRB)	Centers for Disease Control & Prevention {CDC). Division of High Consequence Pathogens and Pathology (DHCPP-PRB)	Gigante,C.M., Hughes,S., Seabolt.M.H., Zhao,H., Wilkins,K., McCollum,A., Hutson,C., Davidson,W., Rao.A., Baumgartner,J. and Li,Y.
EPI_ISL_I4362278	Centers for Disease Contrai & Prevention (CDC), Division of High Consequence Pathogens and Pathology (DHCPP-PRB)	Centers for Disease Contrai & Prevention {CDC). Division of High Consequence Pathogens and Pathology (DHCPP-PRB)	Gigante,C.M., Ghinai,L, Seabolt.M.H., Zhao,H.. Wilkins,K.. McCollum,A.. Hutson,C.. Davidson,W.. Rao.A.. Kerins,J. and Li,Y.
EPI_ISL_I4362280	Centers for Disease Contrai & Prevention (CDC), Division of High Consequence Pathogens and Pathology (DHCPP-PRB)	Centers for Disease Contrai & Prevention {CDC). Division of High Consequence Pathogens and Pathology (DHCPP-PRB)	Gigante,C.M., Lee,P., Seabolt,M.H., Zhao,H., Wilkins,K., McCollum,A., Hutson,C., Davidson,W., Rao.A., Mendoza,R. and Li,Y.
EPI_ISL_I4362282, EPI_ISL_I4362285	Centers for Disease Contrai & Prevention (CDC), Division of High Consequence Pathogens and Pathology (DHCPP-PRB)	Centers for Disease Contrai & Prevention {CDC). Division of High Consequence Pathogens and Pathology (DHCPP-PRB)	Gigante,C.M.. Steidley,B., Seabolt,M.H., Zhao,H., Wilkins,K.. McCollum,A., Hutson,C.. Davidson,W.. Rao.A.. Davizon,E.S. and Li,Y.
EPI_ISL_I4362287	Centers for Disease Contrai & Prevention (CDC), Division of High Consequence Pathogens and Pathology (DHCPP-PRB)	Centers for Disease Contrai & Prevention {CDC). Division of High Consequence Pathogens and Pathology (DHCPP-PRB)	Gigante.CM., Ventura,J., Seabolt,M.H., Zhao,H., Wilkins,K., McCollum,A., Hutson,C.. Davidson,W., Rao.A., Nash.J. and Li,Y.
EPI_ISL_I4362289	Centers for Disease Contrai & Prevention (CDC), Division of High Consequence Pathogens and Pathology (DHCPP-PRB)	Centers for Disease Contrai & Prevention {CDC). Division of High Consequence Pathogens and Pathology (DHCPP-PRB)	Gigante,C.M., Francis,D., Seabolt,M.H., Zhao,H., Wilkins,K., McCollum,A., Hutson,C., Davidson,W., Rao.A., Escobar,J. and li.Y.
EPI_ISL_I4394060	Ryota Kumagai Tokyo Metropolitan Institute of Public Health, Department of Microbiology	Ryota Kumagai Tokyo Metrapolitan Institute of Public Health, Department of Microbiology	Kasuya,F., Negishi,A., Kumagai.R., Hasegawa,M.. Fujiwara,T.,Miyake,H., Nagashima,M. and Sadamasu,K
EPI_ISL_I4414948	UMS Parque Industrial Curitiba	Instituto Adolfo Lutz Strategic Laboratory	Claudio Tavares Sacchi, Karoline Rodrigues Campos, Ariadne Ferreira Amarante, Marion Benedito Nascimento Santos, Alex Domingos Reis, Adriano Abbud, Adriana Bugna
EPI_ISL_I4415810	CTA Sao Miguel	Instituto Adolfo Lutz Strategic Laboratory	Claudio Tavares Sacchi. Karoline Rodrigues Campos, Ariadne Ferreira Amarante. Marion Benedito Nascimento Santos, Alex Domingos Reis. Adriano Abbud. Adriana Bugna
EPI_ISL_I4438678, EPI_ISL_I4438679, EPI_ISL_I4438682, EPI_ISL_I4438683, EPI_ISL_I4438684, EPI_ISL_I4438685, EPI_ISL_I4438686, EPI_ISL_I4438687, EPI_ISL_I4438688, EPI_ISL_I4438689, EPI_ISL_I4438690, EPI_ISL_I4438691, EPI_ISL_I4438692, EPI_ISL_I4438693, EPI_ISL_I4438696, EPI_ISL_I4438697, EPI_ISL_I4438698			
see above	Rhode Island State Health Laboratory	Rhode Island State Health Laboratory	Kristin Carpenter-Azevedo, Sean Sierra-Patev, Richard C. Huard
EPI ISL 14439712, EPI ISL 14439713, EPJ ISL 14439714, EPI JSL 14439715, EPI ISL 14439716, EPI ISL 14439717, EPI ISL 14439718, EPI ISL 14439719, EPI ISL 14439720, EPI ISL 14439721. EPI ISL 14439722, EPI ISL 14439723, EPI ISL 14439724, EPI ISL 14439725, EPI ISL 14439726, EPI ISL 14439727, EPI ISL 14439728, EPI ISL 14439729, EPI ISL 14439730, EPI ISL 14439731. EPI=ISL=I4439732, EPI=ISL)4439733, EPÜSL=I4439734, EPI=ISL=I4439735, EPI=ISL=I4439736, EPI=ISL=I4439737, EPI=ISL)4439738, EPÜSL=I4439739, EPI=ISL)4439740, EPI=ISL=I4439741, EPI=ISL=I4439742, EPI=ISL)4439743, EPÜSL=I4439745, EPI=ISL)4439746, EPI=ISL=I4439747, EPI=ISL=I4439748, EPI=ISL)4439749, EPÜSL=I4439750, EPI=ISL)4439751, EPI=ISL=I4439752, EPI ISL 14439753, EPI ISL 14439754, EPI ISL 14439755, EPI ISL 14439756, EPI ISL 14439757, EPI ISL 14439758, EPI ISL 14439759, EPI ISL 14439760, EPI ISL 14439761, EPI ISL 14439762, EPI ISL 14439763, EPI ISL 14439764, EPI ISL 14439765, EPI ISL 14439766, EPI ISL 14439767, EPI ISL 14439768, EPI ISL 14439769, EPI ISL 14439770, EPI ISL 14439771, EPI ISL 14439772, EPI=ISL)4439773, EPI=ISL)4439774, EPÜSL)4439775, EPI=JSL)4439776, EPI=ISL)4439777, EPI=ISL)4439778, EPI=ISL)4439779, EPÜSL)4439780, EPI=ISL)4439781, EPI=ISL)4439782, EPI=ISL)4439783, EPI=ISL)4439784, EPÜSL)4439785 - - - - - - - - - - - - - -			
see above	Research and Evaluation, UKHSA	Research and Evaluation, UKHSA	Groves,N., Osman,K.L., Lewandowski,K.S., Carter,D.P., Pullan,S.T., Myers,R., Vipond,R. and Chand,M.
EPI_ISL_I4441870, EPI_ISL_I4441871, EPI_ISL_14441872, EPI_ISL_I4441873, EPI_ISL_I4441874, EPI_ISL_I4441875, EPI_ISL_I4441876, EPI_ISL_14441877, EPI_ISL_14441878, EPI_ISL_14441879, EPI_ISL_I4441880, EPI_ISL_14441881, EPI_ISL_14441882, EPI_ISL_14441883, EPI_ISL_14441884, EPI_ISL_14441885, EPI_ISL_14441886, EPI_ISL_14441887			
see above	Rhode Island State Health Laboratory	Rhode Island State Health Laboratory	Kristin Carpenter-Azevedo, Sean Sierra-Patev. Richard C. Huard
EPI ISL 14445098, EPI ISL 14445100, EPI ISL 14445101, EPI ISL 14445102, EPI ISL 14445103, EPI ISL 14445104, EPI ISL 14445107, EPI ISL 14445109, EPI ISL 14445111, EPI ISL 14445113, EPI ISL 14445114, EPI ISL 14445115, EPI ISL 14445116, EPI ISL 14445117, EPI ISL 14445118, EPI ISL 14445119, EPI ISL 14445120, EPI ISL 14445121, EPI ISL 14445122, EPI ISL 14445123, EPI-ISL-14445124, EPI-ISL-14445125, EPI-ISL-14445126, EPI-ISL-14445127, EPI-ISL-14445128, EPI-ISL-14445129, EPI-ISL-14445130, EPI-ISL-14445131, EPI-ISL-14445132, EPI-ISL-14445133, EPI-ISL-14445134, EPI-ISL-14445135, EPI-ISL-14445136, EPI-ISL-14445137, EPI-ISL-14445138, EPI-ISL-14445139, EPI-ISL-14445140, EPI-ISL-14445141, EPI-ISL-14445142, EPI-ISL-14445143, EPI=ISL)4445144, EPI=ISL)4445145, EPÜSL)4445146, EPI=JSL)4445147, EPI=ISL)4445148, EPI=ISL)4445149, EPI=ISL)4445150, EPÜSL)4445151, EPI=ISL)4445152, EPI=ISL)4445153 -			
see above	Laboratorio de Referencia Nacional de Virus Respiratorio. Centra Nacional de Salud Publica. Instituto Nacional de Salud	Laboratorio de Referencia Nacional de Virus Respiratorio. Centra Nacional de Salud Publica. Instituto Nacional de Salud.	Carlos Padilla Rojas, Veronica Hurtado Vela, Iris Silva Molina, Luren Sevilla Castar'ieda, Victor Jimenez Vasquez, Orsan Mestanza Millones, Luis Barcena Flores, Wendy Lizarraga Olivares, Alicia Nur'iez Llanos, Steve Acedo Lazo, Francisco Ascue Orasco, Kelly Izarra Rojas, Princesa Medrano Alhuay, Karla Vasquez Cajachahua, Estela Huaman Angeles, Jorge Giralda Chavez, Lilian Huarca Balbin, Lisbet Roxana Inga Angulo, Maria Sandra Villar Saavedra, Henri Bailon Calderon. Lely Solari Zerpa, Gloria Aratinco Garayar. Equipa de vigilancia genomica del Instituto Nacional de Salud.
EPI_ISL_I4445154, EPI_ISL_I4445155, EPI_ISL_I4445156	Centre for Biological Threats, Highly Pathogenic Viruses, Robert Koch Institute	Centre for Biological Threats, Highly Pathogenic Viruses, Robert Koch Institute	Brinkmann,A., Kohl,C., Pape,K., Uddin,S., Schrick,L., Michel,)., Stocker,H., Schaade,L. and Nitsche,A.
EPI_ISL_14445157, EPI_ISL_14445158, EPJ ISL 14445159, EPI JSL 14445160, EPÜSL)4445161, EPI=ISL)4445162, EPJ ISL 14445163, EPI ISL 14445164, - - EPI_ISL_I444516-5	Centre for Biological Threats. Highly Pathogenic Viruses, Robert Koch Institute	Centre for Biological Threats. Highly Pathogenic Viruses, Robert Koch Institute	Brinkmann.A., Kohl,C., Pape.K., Uddin.S.. Schrick.L.. Michel,).. Jessen,H., Schaade,L. and Nitsche.A.
EPI_ISL_14465517	Centro de Desenvolvimento Cientffico e Tecnol6gico (CDCT), Centra Estadual de Vigilância em Sallde (CEVS) da Secretaria Estadual da Sallde (SES-RS)	Centra de Desenvolvimento Cientffico e Tecnol6gico (CDCT), Centra Estadual de Vigilância em SaUde (CEVS) da Secretaria Estadual da Sallde (SES-RS)	Richard Steiner Salvato, Regina Bones Barcellos, Fernanda Marques Godinho
EPI_ISL_I4467428, EPI_ISL_14467429	Laborat6rio Central de Sallde Pllblica do Amazonas - LACEN-AM	Laborat6rio de Ecologia de Doenças Transmissfveis na Amazônia, Instituto Leônidas e Maria Deane - Fiocruz Amazônia	Victor Souza. Fernanda Nascimento, Matilde Mejia, Dejanane Silva, Luciana Gonçalves. Tatyana Costa Amorim Ramas, Ana Ruth Lima Arcanjo, Valdinete Nascimento. Felipe Naveca on behalf of the Fiocruz COVID-19 Genomic Surveillance Network
EPI_ISL_I4486937, EPI_ISL_I4487241	Instituto Nacional de Salud	Instituto Nacional de Salud- Direcci6n de Investigaci6n en Salud Pllblica	Katherine Laiton-Donato, Diego A. Alvarez-Diaz, Carlos Franco-Mufloz, Héctor A. Ruiz-Moreno, Paola Rojas-Estevez, Alicia Resales, Daniel Martinez, Sergio G6mez, Astrid Carolina Flores, Franklin Prieto, Diana Walteros, Marcela Mercado-Reyes
EPI_ISL_I4487651	Centers for Disease Contrai & Prevention (CDC), Division of High Consequence Pathogens and Pathology (DHCPP-PRB)	Centers for Disease Contrai & Prevention {CDC). Division of High Consequence Pathogens and Pathology (DHCPP-PRB)	Gigante,C.M., Hughes,S., Seabolt,M.H., Zhao,H., Wilkins,K., McCollum,A., Hutson,C., Davidson,W., Rao.A., Baumgartner,J. and Li,Y.
EPI_ISL_I4487652	Centers for Disease Contrai & Prevention (CDC), Division of High Consequence Pathogens and Pathology (DHCPP-PRB)	Centers for Disease Contrai & Prevention {CDC). Division of High Consequence Pathogens and Pathology (DHCPP-PRB)	Gigante,C.M., Griffin-Thomas,L., Seabolt,M.H., Zhao,H., Wilkins,K., McCollum,A., Hutson,C., Davidson,W., Rao.A., Crain.). and li.Y.
EPI_ISL_I4487653	Centers for Disease Contrai & Prevention (CDC), Division of High Consequence Pathogens and Pathology (DHCPP-PRB)	Centers for Disease Contrai & Prevention {CDC). Division of High Consequence Pathogens and Pathology (DHCPP-PRB)	Gigante,C.M., Ghinai,I., Seabolt,M.H., Zhao,H., Wilkins,K., McCollum,A., Hutson,C., Davidson,W., Rao.A., Kerins,J. and Li,Y.
EPI_ISL_I4487654	Centers for Disease Contrai & Prevention (CDC), Division of High Consequence Pathogens and Pathology (DHCPP-PRB)	Centers for Disease Contrai & Prevention {CDC). Division of High Consequence Pathogens and Pathology (DHCPP-PRB)	Gigante,C.M., Steidley,B., Seabolt,M.H., Zhao,H., Wilkins,K., McCollum,A., Hutson,C., Davidson,W., Rao.A., Davizon,E. and Li,Y.
EPI_ISL_I4487655	Centers for Disease Contrai & Prevention (CDC). Division of High Consequence Pathogens and Pathology (DHCPP-PRB)	Centers for Disease Contrai & Prevention (CDC). Division of High Consequence Pathogens and Pathology (DHCPP-PRB)	Gigante,C.M., Ghinai.I., Seabolt.M.H., Zhao.H., Wilkins,K.. McCollum,A.. Hutson.C., Davidson.W., Rao.A., Kerins.J. and Li,Y.
EPI_ISL_I4487656	Centers for Disease Contrai & Prevention (CDC), Division of High Consequence Pathogens and Pathology (DHCPP-PRB)	Centers for Disease Control & Prevention {CDC). Division of High Consequence Pathogens and Pathology (DHCPP-PRB)	Gigante,C.M., Lee,P., Seabolt,M.H., Zhao,H., Wilkins,K., McCollum,A., Hutson,C., Davidson,w., Rao.A., Mendoza,R. and Li,Y.
EPI_ISL_14487657	Centers for Disease Contrai & Prevention (CDC), Division of High Consequence Pathogens and Pathology (DHCPP-PRB)	Centers for Disease Contrai & Prevention {CDC). Division of High Consequence Pathogens and Pathology (DHCPP-PRB)	Gigante,C.M., Ghinai,I., Seabolt,M.H., Zhao,H.. Wilkins,K.. McCollum,A.. Hutson,C.. Davidson,W.. Rao.A.. Kerins,J. and Li.Y.
EPI_ISL_I4487658	Centers for Disease Contrai & Prevention (CDC), Division of High Consequence Pathogens and Pathology (DHCPP-PRB)	Centers for Disease Contrai & Prevention {CDC). Division of High Consequence Pathogens and Pathology (DHCPP-PRB)	Gigante,C.M., Lee,P., Seabolt,M.H., Zhao,H., Wilkins,K., McCollum,A., Hutson,C., Davidson,W., Rao,A., Mendoza,R. and U,Y.
EPI_ISL_I4487659	Centers for Disease Contrai & Prevention (CDC), Division of High Consequence Pathogens and Pathology (DHCPP-PRB)	Centers for Disease Contrai & Prevention (CDC). Division of High Consequence Pathogens and Pathology (DHCPP-PRB)	Gigante,C.M., Hauser.J.R., Seabolt,M.H., Zhao.H., Wilkins.K.. McCollum,A., Hutson.C.. Davidson.W.. Rao.A.. Mangla.A. and Li.Y.
EPI_ISL_I4487660	Centers for Disease Contrai & Prevention (CDC), Division of High Consequence Pathogens and Pathology (DHCPP-PRB)	Centers for Disease Contrai & Prevention {CDC). Division of High Consequence Pathogens and Pathology (DHCPP-PRB)	Gigante,C.M., Ghinai,I., Seabolt,M.H., Zhao,H., Wilkins,K., McCollum,A., Hutson,C., Davidson,W., Rao.A., Kerins,J. and Li,Y.
EPI_ISL_14494949	Division of High-risk Pathogens, Korea Disease Contrai and Prevention Agency	Division of High-risk Pathogens, Korea Disease Contrai and Prevention Agency	Rhie,G.-E.
EPI_ISL_I4515100, EPI_ISL_I4515101, EPI_ISL_I4515102, EPI_ISL_14515103, EPI_ISL_14515104, EPI_ISL_I4515105, EPI_ISL_I4515106, EPI_ISL_I4515107, EPI_ISL_14515108, EPI_ISL_14515109, EPI_ISL_I4515110, EPI_ISL_14515111. EPI_ISL_I4515112, EPI_ISL_14515113			
see above	Centre for Biological Threats, Highly Pathogenic Viruses, Robert Koch Institute	Centre for Biological Threats, Highly Pathogenic Viruses, Robert Koch Institute	Brinkmann,A., Kohl,C., Pape,K., Uddin,S., Schrick,L., Michel,)., Jessen,H., Schaade,L. and Nitsche,A.
EPI_ISL_I4515114, EPI_ISL_I4515115, EPI_ISL_I4515116, EPI_ISL_14515117, EPI_ISL_I4515118, EPI_ISL_I4515119, EPI_ISL_I4515120, EPI_ISL_I4515121, EPI_ISL_14515122, EPI_ISL_I4515123, EPI_ISL_I4515124, EPI_ISL_14515125, EPI_ISL_I4515126, EPI_ISL_14515127, EPI_ISL_I4515128, EPI_ISL_I4515129, EPI_ISL_14515130, EPI_ISL_I4515131, EPI_ISL_14515132, EPI_ISL_I4515133,			

EPI_ISL_l4515134, EPI_ISL_14515135, EPI_ISL_14515136, EPI_ISL_l4515137, EPI_ISL_14515138, EPI_ISL_l4515139, EPI_ISL_l4515140, EPI_ISL_14515141, EPI_ISL_l4515142, EPI_ISL_14515143, EPI_ISL_l4515144, EPI_ISL_l4515145, EPI_ISL_14515146, EPI_ISL_l4515147, EPI_ISL_14515148, EPI_ISL_l4515149, EPI_ISL_l4515150, EPI_ISL_14515151, EPI_ISL_l4515152			
see above	Centre for Biological Threats, Highly Pathogenic Viruses, Robert Koch Institute	Centre for Biological Threats, Highly Pathogenic Viruses, Robert Koch Institute	Brinkmann,A.. Kohl.C., Pape,K.. Uddin,S., Schrick,l., Michel,].. Schaade,L. and Nitsche,A.
EPI ISL 14515153, EPI ISL 14515154, EPI JSL l4515155, EPLISL l4515156	Centre for Biological Threats, Highly Pathogenic Viruses, Robert Koch Institute	Centre for Biological Threats, Highly Pathogenic Viruses, Robert Koch Institute	Brinkmann,A., Kohl,C., Pape,K., Uddin,S., Schrick,L., Michel,]., Jessen,H., Schaade,L. and Nitsche,A.
EPI_ISL_14515157, EPI_ISL_l4515158, EPJ_ISL_l4515159, EPI_JSL_l4515160, EPI_ISL_l4515161, EPI_ISL_14515162, EPI_ISL_l4515163, EPI_ISL_14515164, EPI_ISL_l4515165, EPI_ISL_l4515166, EPI_ISL_14515167, EPI_ISL_l4515168, EPI_ISL_14515169, EPI_ISL_l4515170, EPI_ISL_l4515171, EPI_ISL_l4515172			
see above	Centre for Biological Threats, Highly Pathogenic Viruses, Robert Koch Institute	Centre for Biological Threats, Highly Pathogenic Viruses, Robert Koch Institute	Brinkmann,A., Kohl,C., Pape,K., Uddin,S., Schrick,L., Michel,]., Schaade,L. and Nitsche,A.
EPI ISL 14515173, EPI ISL 14515174, EPJ ISL 14515175, EPI JSL 14515176, EPI ISL 14515178, EPI ISL 14515180, EPI ISL 14515181, EPI ISL 14515182, EPI ISL 14515183, EPI ISL 14515184, EPI ISL 14515185, EPI ISL 14515186, EPI ISL 14515187, EPI ISL 14515188, EPI ISL 14515189, EPI ISL 14515190, EPI ISL 14515191, EPI ISL 14515192, EPI ISL 14515193, EPI ISL 14515194, EPCISL)4515195, EPI=ISL)4515196, EPÜSL)4515197, EPCISL)4515198, EPI=ISL)4515199, EPCISL)4515200, EPI=ISL)4515201, EPÜSL)4515203, EPCISL)4515204, EPI=ISL)4515206, EPCISL)4515208, EPI=ISL)4515209, EPÜSL)4515210, EPCISL)4515211, EPI=ISL)4515212, EPCISL)4515215, EPI=ISL)4515216, EPÜSL)4515217, EPCISL)4515218, EPI=ISL)4515219, EPI_ISL_l4515220, EPI_ISL_14515221. EPI_ISL_l4515222, EPI_ISL_l4515224, EPI_ISL_14515225, EPI_ISL_l4515226, EPI_ISL_l4515227, EPI_ISL_l4515228			
see above	Department of Infectious Diseases, National Institute of Health Doutor Ricardo Jorge, Portugal (INSA)	Department of Infectious Diseases, National Institute of Health Douter Ricardo Jorge, Portugal (INSAJ	Isidro,]., Borges.V., Pinto,M., Sobral,D., Santos,]., Nunes,A., Mixao,V., Ferreira,R., Santos,☐.,Ouarte,S., Vieira,L., Borrego,M.J., Nuncio,S., Lapes de Carvalho,I., Pelerito,A., Cordeiro,R. and Gomes,J.P.
EPI_ISL_l4526939, EPI_ISL_l4526940, EPI_ISL_l4526941, EPI_ISL_l4526942, EPI_ISL_l4526943, EPI_ISL_l4526944, EPI_ISL_l4526945, EPI_ISL_l4526947, EPI_ISL_l4526948, EPI_ISL_l4526949, EPI_ISL_l4526950, EPI_ISL_l4526951, EPI_ISL_l4526952, EPI_ISL_l4526953, EPI_ISL_l4526954, EPI_ISL_l4526955, EPI_ISL_l4526956			
see above	Connecticut Department of Public Health	Grubaugh Lab - Yale School of Public Health	Nicholas F. G. Chen, Chrispin Chaguza, Kien Pham, Nathan D. Grubaugh, Christina Nishimura, Claire Pearson, Kutluhan Incekara. Jian Ping Huang, Emily Gagnon, Ethan Reever, Jafar Razeq, Anthony Muyombwe, Chantal B.F. Vogels
EPJ_ISL_l4541645, EPI_ISL_l4S41647, EPI ISL 14541649, EPI ISL 14541652, - - EPI_ISL_l454165-4	Public Health Authority of the Slovak Republic	Laboratory of Genomics and Bioinformatics, Comenius University Science Park	Tomâs Szemes, Edita Staroi'lovâ, Elena Tichâ, Lucia evéikovâ, Terézia Vrabrovâ, Tatiana Sedlâèkovâ, Miroslav Bôhmer, Jaroslav BudiS, Pavai MiSenko
EPI ISL 14553812 - -	Hospital CIMA San Jose	Inciensa, Instituto de Investigaci6n y Ensefianza en Nutrici6n y Salud	Francisco Duarte, Ana lsela Ruiz-Gonzalez, Hillary Serrano, Diana Cantillo, Claudio Soto-Garita, Gustavo Vega, Estela Cordera, Adriana Godinez & Melany Calderon
EPI_ISL_l4561914, EPI_ISL_l4561915, EPJ_ISL_l4561916, EPI_JSL_l4561917, EPI_ISL_l4561918, EPI_ISL_l4561919, EPI_ISL_l4561920, EPI_ISL_14561921, EPI_ISL_l4561922, EPI_ISL_14561923, EPI_ISL_l4561924, EPI_ISL_l4561925, EPI_ISL_14561926, EPI_ISL_l4561927, EPI_ISL_14561928, EPI_ISL_l4561929, EPI_ISL_l4561930, EPI_ISL_14561931, EPI_ISL_l4561932			
see above	Los Angeles County Public Health Laboratories	Los Angeles County Public Health Laboratories	P. Hemarajata et al.
EPI ISL 14562478. EPI ISL 14562479, EPI ISL 14562480, EPI ISL 14562481. EPI ISL 14562482, EPI ISL 14562483, EPI ISL 14562484, EPI ISL 14562485, EPI ISL 14562486, EPI ISL 14562487, EPI ISL 14562488, EPI ISL 14562489, EPI ISL 14562490, EPI ISL 14562491. EPI ISL 14562492, EPI ISL 14562493, EPI ISL 14562494, EPI ISL 14562495, EPI ISL 14562496, EPI ISL 14562497, EPI=ISL)4562498, EPI=ISL)4562499, EPÜSL)4562500, EPI=JSL)4562501, EPI=ISL)4562502			
see above	Laboratory Medicine, UW Virology	Laboratory Medicine, UW Virology	Sereewit,J., Xie,H., Roychoudhury,P. and Greninger,A.L.
EPI ISL 14562503, EPI ISL 14562504, EPJ-ISL-14562505, EPI-JSL-14562506, EPÜSL)4562507, EPCISL)4562508, EPJ ISL 14562509, EPI JSL 14562510, EPI JSL l4562511, EPLISL l4562512	Centre for Biological Threats, Highly Pathogenic Viruses, Robert Koch Institute	Centre for Biological Threats. Highly Pathogenic Viruses, Robert Koch Institute	Brinkmann,A., Kohl,C.. Uddin,S.. Pape,K., Schrick,l., Michel,]., Schaade,L. and Nitsche,A.
EPI_ISL_l4571429	Hosp. Municipal Dr. Jose de Carvalho Florence	Instituto Adolfo Lutz Strategic Laboratory	Claudio Tavares Sacchi, Karoline Rodrigues Campos, Ariadne Ferreira Amarante, Marion Benedito Nascimento Santos, Alex oomingos Reis, Adriano Abbud, Adriana Bugna
EPI_ISL_l4571433	Casa de Saude Stella Maris	Instituto Adolfo Lutz Strategic Laboratory	Claudio Tavares Sacchi, Karoline Rodrigues Campos, Ariadne Ferreira Amarante, Marion Benedito Nascimento Santos, Alex Oomingos Reis, Adriano Abbud, Adriana Bugna
EPI_ISL_l4571435	Secretaria Municipal de Saude de Sertaozinho	Instituto Adolfo Lutz Strategic Laboratory	Claudio Tavares Sacchi, Karoline Rodrigues Campos, Ariadne Ferreira Amarante, Marion Benedito Nascimento Santos, Alex Domingos Reis, Adriano Abbud, Adriana Bugno
EPI_ISL_l4571439	Secretaria Municipal de Saude de Sata Barbara D Oeste	Instituto Adolfo Lutz Strategic Laboratory	Claudio Tavares Sacchi, Karaline Rodrigues Campos, Ariadne Ferreira Amarante, Marion Benedito Nascimento Santos, Alex Oomingos Reis, Adriano Abbud, Adriana Bugno
EPI_ISL_l4571441	Hosp. Municipal Or. Waldemar Tebaldi	Instituto Adolfo Lutz Strategic Laboratory	Claudio Tavares Sacchi, Karaline Rodrigues Campos, Ariadne Ferreira Amarante, Marion Benedito Nascimento Santos, Alex Oomingos Reis, Adriano Abbud, Adriana Bugno
EPI_ISL_l4571442	Instituto de Infectologia Emilio Ribas II Baixada Santista	Instituto Adolfo Lutz Strategic Laboratory	Claudio Tavares Sacchi, Karaline Rodrigues Campos, Ariadne Ferreira Amarante, Marion Benedito Nascimento Santos, Alex Domingos Reis, Adriano Abbud, Adriana Bugno
EPI_ISL_l4571444	UBDS DR. Italo Baruffi Castelo Branco	Instituto Adolfo Lutz Strategic Laboratory	Claudio Tavares Sacchi, Karoline Rodrigues Campos, Ariadne Ferreira Amarante, Marion Benedito Nascimento Santos, Alex Oomingos Reis, Adriano Abbud, Adriana Bugno
EPI_ISL_14583298, EPI_ISL_14583299, EPJ_ISL_14583300, EPI_ISL_14583301, EPI_ISL_14583302, EPI_ISL_14583303, EPI_ISL_14583305, EPI_ISL_14583306, EPI_ISL_14583307, EPI_ISL_14583308, EPI_ISL_14583309			
see above	Rhode Island State Health Laboratory	Rhode Island State Health Laboratory	Kristin Carpenter-Azevedo, Sean Sierra-Patev, Richard C. Huard
EPI ISL 14584274, EPI ISL 14584275, EPJ ISL 14584276, EPI JSL 14584277, EPI ISL 14584278, EPI ISL 14584279, EPI ISL 14584281. EPI ISL 14584282, EPI ISL 14584283, EPI ISL 14584284, EPI ISL 14584286, EPI ISL 14584287, EPI ISL 14584289, EPI ISL 14584290, EPI ISL 14584291. EPI ISL 14584292, EPI ISL 14584293, EPI ISL 14584294, EPI ISL 14584295, EPI ISL 14584296, EPI=ISL)4584297, EPI=ISL)4584298, EPÜSL)4584299, EPI=ISL)4584300, EPI=ISL)4584301, EPI=ISL)4584302, EPI=ISL)4584303, EPÜSL)4584304, EPI=ISL)4584305, EPI=ISL)4584306, EPI=ISL)4584307, EPI=ISL)4584308, EPÜSL)4584309, EPI=ISL)4584310, EPI=ISL)4584311			
see above	Laboratorio de Referencia Nacional de Virus Respiratorio. Centra Nacional de Salud Publica. Instituto Nacional de Salud	Laboratorio de Referencia Nacional de Virus Respiratorio. Centra Nacional de Salud Publica. Instituto Nacional de Salud.	Carlos Padilla Rojas, Veronica Hurtado Vela, Iris Silva Molina, Luren Sevilla Castafieda, Victor Jimenez Vasquez, Orsan Mestanza Millones, Luis Barcena Flores, Wendy Lizarraga Olivares, Alicia Nufiez Llanos, Steve Acedo Lazo, Francisco Ascue Orasco, Kelly Izarra Rojas, Princesa Medrano Alhuay, Karla Vasquez Cajachahua, Estela Huaman Angeles, Jorge Giraldo Chavez, Lilian Huarca Balbin, Lisbet Roxana Inga Angulo, Maria Sandra Villar Saavedra, Henri Bailon Calderon, Lely Solari Zerpa, Gloria Arotinco Garayar. Equipa de vigilancia genomica del Instituto Nacional de Salud.
EPI_ISL_14586688	Public Health Authority of the Slovak Republic	Laboratory of Genomics and Bioinformatics, Comenius University Science Park	TomâS Szemes, Edita Starariovâ, Elena Tichâ, Lucia evèfkovâ, Terézia Vrabrovâ. Tatiana Sedlâèkovâ. Miroslav Bôhmer, Jaroslav BudiS. Pavol MiSenko
EPJ ISL 14587544, EPI JSL 14587545, EPJ-ISL-14587546, EPI-ISL-14587548, EPÜSL)4587549, EPCISL=l4587550, EPI_ISL_l4587551	Public Health Agency of Canada, National Microbiology Laboratory	Public Health Agency of Canada, National Microbiology Laboratory	Duggan,A., Hole,D., Yadav,C., Knox,N., Tyler.A., Haidl,E., Chapel,M., Domselaar,G.V., Graham,M . Audet.J., Fernando,L., Hagan,M., Safronetz,D., Leung,A., Peters,G., Go.A., Laminman,V., Kaplen,8., Antonation,K., Griffiths,E., Jolly,G., Charest,H., Levade,I. and Fafard,J.
EPJ ISL 14587552, EPI JSL 14587553, EPJ-ISL-14587554, EPI-ISL-14587555, EPÜSL)4587556, EPCISL=l4587557, EPI_ISL_l4587558	Centre for Biological Threats, Highly Pathogenic Viruses, Robert Koch Institute	Centre for Biological Threats. Highly Pathogenic Viruses, Robert Koch Institute	Brinkmann,A.. Kohl.C., Pape,K.. Uddin,S.. Schrick,l., Michel.].. Friesen,].. Schaade,L. and Nitsche,A.
EPI_ISL_l4594041, EPI_ISL_14594042, EPJ_ISL_14594043, EPI_JSL_l4594047, EPI_ISL_14594049, EPI_ISL_l4594050, EPI_ISL_l4594051, EPI_ISL_l4594052, EPI_ISL_l4594053, EPI_ISL_l4594054, EPI_ISL_l4594055, EPI_ISL_14594056			
see above	Public Health Agency of Canada, National Microbiology Laboratory	Public Health Agency of Canada, National Microbiology Laboratory	Duggan,A., Hole,D., Yadav,C.. Knox,N., Tyler.A., Haidl.E., Chapel,M, Domselaar,G.V., Graham,M., Audet.J., Fernando,L., Antonation,K., Safronetz.D., Hagan,M., Peters,G., Go.A., Laminman,V., Kaplen,8., Leung,A., Griffiths,E., Jolly,G., Eshaghi,A., Gubbay,J.B., Hasso,M., Marchand-Austin.A., Olsha,R. and Patel,S.N.
EPI_ISL_14615579	RSUPN dr. Cipto Mangunkusumo	National Institute of Health Research and Development	Hana Apsari Pawestri, Arie Ardiansyah Nugraha, Fajar Nur Sulistiyohadi, Subangkit, Krisna NA Pangesti, Tze Minn Mak, 1 Gede Made Wirabrata
EPI_JSL_l4621525, EPI_ISL_14621526	Virology, APHP Pitie Salpetriere SU	Virology, APHP Pitie Salpetriere SU	Seang,S., Burrel,S., Todesco,E., Leducq,V., Monsel,G., Le Pluart,D., Cordevant.C., Pourcher,V. and Palich,R.
EPI_ISL_14622055	Instituto de Infectologia Emilio Ribas	Instituto Adolfo Lutz Strategic Laboratory	Claudio Tavares Sacchi, Karaline Rodrigues Campos. Ariadne Ferreira Amarante. Marion Benedito Nascimento Santos, Alex Domingos Reis. Adriano Abbud, Adriana Bugno
EPI_ISL_14622520	UBSJovaia	Instituto Adolfo Lutz Strategic Laboratory	Claudio Tavares Sacchi, Karaline Rodrigues Campos, Ariadne Ferreira Amarante, Marion Benedito Nascimento Santos, Alex Domingos Reis, Adriano Abbud, Adriana Bugno
EPI_ISL_l4622705	UBS Jardim Santista	Instituto Adolfo Lutz Strategic Laboratory	Claudio Tavares Sacchi, Karoline Rodrigues Campos, Ariadne Ferreira Amarante, Marion Benedito Nascimento Santos, Alex oomingos Reis, Adriano Abbud, Adriana Bugno
EPI_ISL_14622706	Centra de Referencia Modulo I SAE II Bauru	Instituto Adolfo Lutz Strategic Laboratory	Claudio Tavares Sacchi, Karoline Rodrigues Campos, Ariadne Ferreira Amarante, Marion Benedito Nascimento Santos, Alex Oomingos Reis, Adriano Abbud, Adriana Bugno
EPI_ISL_l4622707	USF Boicucanga I Sao Sebastiao	Instituto Adolfo Lutz Strategic Laboratory	Claudio Tavares Sacchi, Karoline Rodrigues Campos, Ariadne Ferreira Amarante, Marion Benedito Nascimento Santos, Alex Domingos Reis, Adriano Abbud, Adriana Bugna
EPI_ISL_l4622913	Secretaria Municipal de Saude de Caxias do Sul	Instituto Adolfo Lutz Strategic Laboratory	Claudio Tavares Sacchi, Karaline Rodrigues Campos. Ariadne Ferreira Amarante, Marion Benedito Nascimento Santos, Alex Domingos Reis, Adriano Abbud, Adriana Bugno
EPI_ISL_14622953	Sistema de Vigilancia em Saude Viamao	Instituto Adolfo Lutz Strategic Laboratory	Claudio Tavares Sacchi, Karaline Rodrigues Campos, Ariadne Ferreira Amarante, Marion Benedito Nascimento Santos, Alex Oomingos Reis, Adriano Abbud, Adriana Bugno
EPI_ISL_l4622960	Vigilancia Epidemiologica Municipal	Instituto Adolfo Lutz Strategic Laboratory	Claudio Tavares Sacchi, Karaline Rodrigues Campos, Ariadne Ferreira Amarante, Marion Benedito Nascimento Santos, Alex Domingos Reis, Adriano Abbud, Adriana Bugno
EPI_ISL_l4623175	Centra de Referencia em Especialidades Central Rib Preto	Instituto Adolfo Lutz Strategic Laboratory	Claudio Tavares Sacchi, Karoline Rodrigues Campos, Ariadne Ferreira Amarante, Marion Benedito Nascimento Santos, Alex Domingos Reis, Adriano Abbud, Adriana Bugna
EPI_ISL_l4623523	Laboratorio Municipal de Piracicaba	Instituto Adolfo Lutz Strategic Laboratory	Claudio Tavares Sacchi, Karoline Rodrigues Campos, Ariadne Ferreira Amarante, Marion Benedito Nascimento Santos, Alex Domingos Reis, Adriano Abbud, Adriana Bugno
EPI_ISL_l4623704	Unidade Basica de Saude Esplanada	Instituto Adolfo Lutz Strategic Laboratory	Claudio Tavares Sacchi, Karoline Rodrigues Campos, Ariadne Ferreira Amarante, Marion Benedito Nascimento Santos, Alex Domingos Reis, Adriano Abbud, Adriana Bugna
EPI_ISL_l4624411	Hospital Albert Sabin Atibaia	Instituto Adolfo Lutz Strategic Laboratory	Claudio Tavares Sacchi, Karaline Rodrigues Campos, Ariadne Ferreira Amarante, Marion Benedito Nascimento Santos, Alex Domingos Reis, Adriano Abbud, Adriana Bugno
EPI_ISL_14624610	USAFA Forte	Instituto Adolfo Lutz Strategic Laboratory	Claudio Tavares Sacchi, Karoline Rodrigues Campos, Ariadne Ferreira Amarante, Marion Benedito Nascimento Santos, Alex Oomingos Reis, Adriano Abbud, Adriana Bugno
EPI_ISL_14624698	Centra de Referencia em AIDS SECRAIDS	Instituto Adolfo Lutz Strategic Laboratory	Claudio Tavares Sacchi, Karoline Rodrigues Campos, Ariadne Ferreira Amarante, Marion Benedito Nascimento Santos, Alex Oomingos Reis, Adriano Abbud, Adriana Bugno
EPI_ISL_l4624832	Servico de Vigilancia Epidemiologica e de Zoonoses do Guaruja	Instituto Adolfo Lutz Strategic Laboratory	Claudio Tavares Sacchi, Karoline Rodrigues Campos, Ariadne Ferreira Amarante, Marion Benedito Nascimento Santos, Alex Domingos Reis, Adriano Abbud, Adriana Bugna
EPI_ISL_l4624915	SMSAruja	Instituto Adolfo Lutz Strategic Laboratory	Claudio Tavares Sacchi, Karaline Rodrigues Campos, Ariadne Ferreira Amarante. Marion Benedito Nascimento Santos, Alex oomingos Reis. Adriano Abbud, Adriana Bugno
EPI_ISL_l4625156	Secretaria Municipal de Saude de Suzano	Instituto Adolfo Lutz Strategic Laboratory	Claudio Tavares Sacchi, Karoline Rodrigues Campos, Ariadne Ferreira Amarante, Marion Benedito Nascimento Santos, Alex Domingos Reis, Adriano Abbud, Adriana Bugna
EPI_ISL_l4625157	PSF Vila Nossa Senhora de Fatima Fartura	Instituto Adolfo Lutz Strategic Laboratory	Claudio Tavares Sacchi, Karoline Rodrigues Campos, Ariadne Ferreira Amarante, Marion Benedito Nascimento Santos, Alex Domingos Reis, Adriano Abbud, Adriana Bugna
EPI_ISL_l4625190	Ambulatorio de Atendimentode DST de Guariba	Instituto Adolfo Lutz Strategic Laboratory	Claudio Tavares Sacchi, Karaline Rodrigues Cajachahua Campos, Ariadne Ferreira Amarante, Marion Benedito Nascimento Santos, Alex Oomingos Reis, Adriano Abbud, Adriana Bugno
EPI_ISL_14625230	UBS Centra Clair Aparecida Pavan	Instituto Adolfo Lutz Strategic Laboratory	Claudio Tavares Sacchi, Karaline Rodrigues Campos, Ariadne Ferreira Amarante, Marion Benedito Nascimento Santos, Alex Domingos Reis, Adriano Abbud, Adriana Bugno
EPI_ISL_l4625256	UMS Campina do Siqueira	Instituto Adolfo Lutz Strategic Laboratory	Claudio Tavares Sacchi, Karaline Rodrigues Campos, Ariadne Ferreira Amarante, Marion Benedito Nascimento Santos, Alex oomingos Reis, Adriano Abbud, Adriana Bugno
EPI_ISL_l4625282	Hospital Edmundo Vasconcelos	Instituto Adolfo Lutz Strategic Laboratory	Claudio Tavares Sacchi, Karoline Rodrigues Campos, Ariadne Ferreira Amarante, Marion Benedito Nascimento Santos, Alex Domingos Reis, Adriano Abbud, Adriana Bugna

EPI ISL 14664595, EPI ISL 14665380, EPI=ISL=l4665384, EPI=JSL=l4665389, EPI ISL 14665390, EPI ISL 14665391. - - EPI_ISL_l466539-3	CT Department of Public Health	CT Department of Public Health	Claire Pearson, Tu N. Nguyen. Kutluhan Incekara
EPI_ISL_l4666780	Public Health Authority of the Slovak Republic	Laboratory of Genomics and Bioinformatics, Comenius University Science Park	Tomas Szemes. Edita Staror'iovâ, Elena Tichâ. Lucia $evcfkovâ. Terézia Vrabrovâ, Tatiana SedlâCkovâ, Miroslav Bôhmer, Jaroslav BudiS, Pavol MiSenko
EPI_ISL_l4676265	Centro de Desenvolvimento Cientffico e Tecnol6gico (CDCT)/CEVS/SES-RS	Centro de Desenvolvimento Cientffico e Tecnol6gico (CDCT)/CEVS/SES-RS	Richard Steiner Salvato, Regina Bones Barcellos, Fernanda Marques Godinho
EPI_ISL_l4699907, EPI_JSL_l4699908, EPI_ISL_l4699909, EPI_ISL_l4699910	Centers for Disease Control & Prevention (CDC), Division of High Consequence Pathogens and Pathology (DHCPP-PRB)	Centers for Disease Control & Prevention {CDC). Division of High Consequence Pathogens and Pathology (DHCPP-PRB)	Gigante,C.M., Lee,P., Zhao,H., Batra,D., Hetrick,E.E., Howard,D.T., Kovar,L., Seabolt,M.H., Weigand,M.R., Burroughs,M.S., Lee,J., Wilkins,K., McCollum,A., Hutson,C., Davidson,W., Rao.A., Mendoza,R. and Li,Y.
EPI ISL 14699911, EPI ISL 14699912, EPI=ISL=l4699913, EPI=JSL)4699914, EPI_ISL_l4699915	Centers for Disease Contrai & Prevention (CDC), Division of High Consequence Pathogens and Pathology (DHCPP-PRB)	Centers for Disease Control & Prevention {CDC). Division of High Consequence Pathogens and Pathology (DHCPP-PRB)	Gigante,C.M., Ghinai,I., Zhao,H., Batra,D., Hetrick,E.E., Howard,D.T., Kovar,L., Seabolt.M.H.. Weigand,M.R., Burroughs,M.5., Lee,J., Wilkins,K., McCollum,A.. Hutson,C., Davidson,W., Rao.A., Kerins,J. and Li.Y.
EPI_ISL_l4699916	Centers for Disease Control & Prevention (CDC), Division of High Consequence Pathogens and Pathology (DHCPP-PRB)	Centers for Disease Control & Prevention {CDC). Division of High Consequence Pathogens and Pathology (DHCPP-PRB)	Gigante,C.M., Kubin,G., Zhao,H., Batra,D., Hetrick.E.E., Howard,D.T., Kovar,L., Seabolt,M.H., Weigand.M.R., Burroughs,M.S., Lee.].. Wilkins,K., McCollum,A., Hutson,C.. Davidson,W.. Rao,A.. White,S.L. and Li,Y.
EPI_ISL_l4699917	Centers for Disease Control & Prevention (CDC), Division of High Consequence Pathogens and Pathology (DHCPP-PRB)	Centers for Disease Control & Prevention (CDC). Division of High Consequence Pathogens and Pathology (DHCPP-PRB)	Gigante,C.M., Winter,K., Zhao,H., Batra,D., Hetrick,E.E., Howard,D.T., Kovar,L., Seabolt,M.H., Weigand,M.R., Burroughs,M.S., Lee,J., Wilkins,K., McCollum,A., Hutson,C., Davidson,w., Rao.A., Arora,v. and Li,Y.
EPI_ISL_l4699918	Centers for Disease Control & Prevention (CDC), Division of High Consequence Pathogens and Pathology (DHCPP-PRB)	Centers for Disease Control & Prevention {CDC). Division of High Consequence Pathogens and Pathology (DHCPP-PRB)	Gigante,C.M., Hughes,S.. Zhao,H., Batra,D., Hetrick,E.E., Howard,D.T., Kovar,L., Seabolt,M.H., Weigand,M.R., Burroughs,M.S., Lee,J., Wilkins,K., McCollum,A., Hutson,C., Davidson,W., Rao,A., Baumgartner,J. and Li,Y.
EPI_ISL_l4699919	Centers for Disease Control & Prevention (CDC), Division of High Consequence Pathogens and Pathology (DHCPP-PRB)	Centers for Disease Control & Prevention (CDC). Division of High Consequence Pathogens and Pathology (DHCPP-PRB)	Gigante,C.M., Ghinai,I., Zhao,H., Batra,D., Hetrick,E.E., Howard,D.T., Kovar,L., Seabolt,M.H., Weigand,M.R., Burroughs,M.S., Lee,J., Wilkins,K., McCollum,A., Hutson,C., Davidson,w., Rao.A., Kerins,J. and Li,Y.
EPI_ISL_l4699920, EPI_ISL_l4699921	Centers for Disease Contrai & Prevention (CDC), Division of High Consequence Pathogens and Pathology (DHCPP-PRB)	Centers for Disease Control & Prevention {CDC). Division of High Consequence Pathogens and Pathology (DHCPP-PRB)	Gigante,C.M.. lwen,P.C.. Zhao,H., Batra,D.. Hetrick,E.E.. Howard,D.T.. Kovar,L.. Seabolt,M.H., Weigand,M.R.. Burroughs,M.S., Lee,J.. Wilkins,K., McCollum,A.. Hutson,C., Davidson,W., Rao,A., Donahue,M. and Li.Y.
EPI_JSL_l4699922, EPI_ISL_l4699923	Centers for Disease Control & Prevention (CDC), Division of High Consequence Pathogens and Pathology (DHCPP-PRB)	Centers for Disease Control & Prevention {CDC). Division of High Consequence Pathogens and Pathology (DHCPP-PRB)	Gigante,C.M., Hughes,S., Zhao,H., Batra,D., Hetrick,E.E., Howard,D.T., Kovar,L., Seabolt,M.H., Weigand,M.R., Burroughs,M.S., Lee,J., Wilkins,K., McCollum,A., Hutson,C., Davidson,W., Rao.A., Baumgartner,J. and Li,Y.
EPI_ISL_l4699924	Centers for Disease Contrai & Prevention (CDC), Division of High Consequence Pathogens and Pathology (DHCPP-PRB)	Centers for Disease Control & Prevention {CDC). Division of High Consequence Pathogens and Pathology (DHCPP-PRB)	Gigante,C.M., Lee,B., Zhao,H., Batra,D., Hetrick,E.E., Howard,D.T., Kovar,L., Seabolt,M.H., Weigand,M.R., Burroughs,M.S., Lee,J., Wilkins,K., McCollum,A., Hutson,C., Davidson,W., Rao.A., Salehi,E. and Li,Y.
EPI_ISL_l4699925	Centers for Disease Control & Prevention (CDC). Division of High Consequence Pathogens and Pathology (DHCPP-PRB)	Centers for Disease Control & Prevention {CDC). Division of High Consequence Pathogens and Pathology (DHCPP-PRB)	Gigante,C.M., Hughes,S.. Zhao,H., Batra,D., Hetrick.E.E., Howard,D.T., Kovar,L., Seabolt,M.H., Weigand,M.R.. Burroughs,M.S., Lee.J., Wilkins.K., McCollum,A., Hutson,C., Davidson,W., Rao,A., Baumgartner,J. and Li,Y
EPI_ISL_14699926	Centers for Disease Control & Prevention (CDC). Division of High Consequence Pathogens and Pathology (DHCPP-PRB)	Centers for Disease Control & Prevention (CDC). Division of High Consequence Pathogens and Pathology (DHCPP-PRB)	Gigante,C.M., Griffin-Thomas,L., Zhao,H., Batra,D., Hetrick,E.E., Howard,D.T., Kovar,L., Seabolt,M.H., Weigand,M.R., Burroughs,M.S., Lee,J., Wilkins,K., McCollum,A., Hutson,C., Davidson,w., Rao.A., Crain,J. and Li,Y.
EPI ISL 14699927, EPI ISL 14699928, EPI ISL 14699929, EPI ISL 14699930, EPI ISL 14699931, EPI ISL 14699932, EPI ISL 14699933, EPI ISL 14699934, EPI ISL 14699935, EPI ISL 14699936, EPI ISL 14699937, EPI ISL 14699938, EPI ISL 14699939, EPI ISL 14699940, EPI ISL 14699941, EPI ISL 14699942, EPI ISL 14699943, EPI ISL 14699944, EPI ISL 14699945, EPI ISL 14699946, EPCISL)4699947, EPI=ISL)4699948, EPÜSL)4699949, EPI=JSL)4699950, EPI=ISL)4699951, EPI=ISL)4699952, EPI=ISL)4699953, EPÜSL)4699954, EPI=ISL)4699955, EPI=ISL)4699956, EPI=ISL)4699957, EPI=ISL)4699958			
see above	Laboratory Medicine, UW Virology	Laboratory Medicine, UW Virology	Sereewit.J.. Xie,H., Roychoudhury,P. and Greninger,A.L.
EPI_ISL_14707250	Charité Universitatsmedizin Berlin, Institut für Virologie	Charité Universitatsmedizin Berlin, Institut für Virologie	Julia Schneider, Victor M Carman. Terry C Jones, Christian Drosten
EPI ISL 14721255, EPI ISL 14721256, EPI-ISL-14721259, EPI-ISL-14721262, EPÜSL)4721263, EPCISL)4721264, EPI_ISL_14721265	National Public Health Laboratory, National Centre for Infectious Diseases	National Public Health Laboratory. National Centre for Infectious Diseases	Yichen Ding, Benny Yeo, Daniel Lim, Zhenyang Zhou, Royce Ang, Samuel Loo, Lin Cui, Raymond Tzer Pin Lin
EPI ISL 14736400, EPI ISL 14736401, EPI-ISL-14736402, EPI-ISL-14736403, EPÜSL)4736404, EPCISL=14736405, EPI ISL 14736406, EPI ISL 14736408, - - EPI_ISL_l473640-9	California Department of Public Health	California Department of Public Health	Viral and Rickettsial Disease Laboratory
EPI_ISL_l4752090, EPI_ISL_14752091, EPI ISL 14752093, EPI ISL 14752094, - - EPI_ISL_l475209-6	Environmental. Agricultural. and Occupational Health. University of Nebraska Medical Center	Environmental. Agricultural, and Occupational Health. University of Nebraska Medical Center	Tegomoh.B., Cross.S.T.. Chapman.R.C., Bernhard.K., McCutchen,E.L., Fauver,J.R., Pratt,C.B., Warden,D.E.. lwen,P.C.. Donahue,M. and Wiley.M.R
EPI ISL 14752098, EPI ISL 14752100, EPI ISL 14752102, EPI ISL 14752104, EPI ISL 14752106, EPI ISL 14752108, EPI ISL 14752109, EPI ISL 14752111, EPI ISL 14752115, EPI ISL 14752117, EPI ISL 14752119, EPI ISL 14752120, EPI ISL 14752122, EPI ISL 14752124, EPI ISL 14752126, EPI ISL 14752127, EPI ISL 14752128, EPI ISL 14752129, EPI ISL 14752130, EPI ISL 14752131, EPI=ISL)4752132. EPI=ISL)4752133, EPÜSL)4752135. EPI=ISL)4752137, EPI=ISL)4752139, EPI=ISL)4752141. EPI=ISL)4752143, EPÜSL)4752145. EPI=ISL)4752146, EPI=ISL)4752148, EPI=ISL)4752150. EPI=ISL)4752152, EPÜSL)4752154. EPI=ISL)4752156. EPI=ISL)4752157, EPI=ISL)4752159. EPI=ISL)4752161, EPÜSL)4752163. EPI=ISL)4752165, EPI=ISL)4752167, EPI ISL 14752169, EPI ISL 14752171. EPI ISL 14752173, EPI ISL 14752175, EPI ISL 14752177, EPI ISL 14752178, EPI ISL 14752180, EPI ISL 14752182, EPI ISL 14752184, EPI ISL 14752186, EPI ISL 14752188, EPI ISL 14752189, EPI ISL 14752191, EPI ISL 14752193, EPI ISL 14752195, EPI ISL 14752199, EPI ISL 14752202, EPI ISL 14752204, EPI ISL 14752206, EPI ISL 14752208, EPI-ISL-14752211, EPI-ISL-14752212, EPI-ISL-14752213, EPI-ISL-14752214, EPI-ISL-14752215, EPI-ISL-14752216, EPI-ISL-14752217, EPI-ISL-14752218, EPI-ISL-14752219, EPI-ISL-14752221, EPI-ISL-14752222, EPI-ISL-14752224, EPI-ISL-147S222S, EPI-ISL-147S2229, EPI-ISL-14752231, EPI-ISL-147S2233, EPI-ISL-14752236, EPI-ISL-14752242, EPI-ISL-14752246, EPI-ISL-14752248, EP(ISL)4752249, EPI=ISL)4752251, EPÜSL)4752253			
see above	Department of Infectious Diseases, National Institute of Health Doutor Ricardo Jorge (INSA)	Department of Infectious Diseases, National Institute of Health Doutor Ricardo Jorge (INSA)	Isidro.)., Borges,V.. Pinto,M.. Sobral.D., Santos.).. Nunes,A., Mixao.V.. Ferreira,R.. Santos,□.. Duarte,S., Vieira,L., Borrego.M.J., Nuncio.S., Lopes de Carvalho,I.. Pelerito,A.. Cordeiro.R. and Gomes,J.P.
EPI ISL 14752257. EPI ISL 14752259, EPJ-ISL-14752260, EPI-ISL-14752261, EPI=.JsL=.14752262, EPI:.ISL-=-14752263	Laboratory Medicine, UW Virology	Laboratory Medicine. UW Virology	Sereewit.J., Xie.H.. Roychoudhury.P. and Greninger.A.L
EPI_ISL_l4752264, EPI_ISL_l4752265, EPI_ISL_l4752267, EPI_ISL_l4752269, EPI_ISL_l4752270, EPI_ISL_l4752272, EPI_ISL_l4752274, EPI_ISL_l4752276, EPI_ISL_l4752278, EPI_ISL_l4752280, EPI_ISL_l4752282			
see above	Centre for Biological Threats, Highly Pathogenic Viruses, Robert Koch Institute	Centre for Biological Threats, Highly Pathogenic Viruses, Robert Koch Institute	Brinkmann,A., Kohl,C., Uddin,S., Pape,K., Schrick,L., Michel,)., Schaade,L. and Nitsche,A.
EPI_ISL_l4752284	Research and Evaluation, UKHSA	Research and Evaluation, UKHSA	Grove,N., Osman,K.L., Lewandowski,K.S., Carter,D.P., Pullan,S.T., Myers,R., Vipond,R. and Chand,M.
EPI ISL 14752286, EPI ISL 14752288, EP1=_1sL-=.14752290, EPi:.ISL-=-14752291	Research and Evaluation, UKHSA	Research and Evaluation, UKHSA	Groves,N., Osman,K.L., Lewandowski,K.S., Carter,D.P., Pullan,S.T., Myers,R., Vipond,R. and Chand,M.
EPI_ISL_l4752293	Medical Microbiology & Infection Prevention, Amsterdam Medical Centres location AMC	Medical Microbiology & Infection Prevention, Amsterdam Medical Centres location AMC	Welkers,M., Jonges,M., de Regt,M., Ooijevaar,R. and Wagemakers,A.
EPI_ISL_14772317	Policlinica Jacare Wilson Federzoni (abreuva	Instituto Adolfo Lutz Strategic Laboratory	Claudio Tavares Sacchi, Karoline Rodrigues Campos, Ariadne Ferreira Amarante, Marion Benedito Nascimento Santos, Alex Domingos Reis, Adriano Abbud. Adriana Bugno
EPI_ISL_l4772318	Secretaria Municipal de Saude de Sertaozinho	Instituto Adolfo Lutz Strategic Laboratory	Claudio Tavares Sacchi, Karoline Rodrigues Campos, Ariadne Ferreira Amarante, Marion 8enedito Nascimento Santos, Alex Domingos Reis, Adriano Abbud, Adriana Bugno
EPI_ISL_l4772912	USF Jardim Oratorio	Instituto Adolfo Lutz Strategic Laboratory	Claudio Tavares Sacchi, Karoline Rodrigues Campos, Ariadne Ferreira Amarante, Marion Benedito Nascimento Santos, Alex Domingos Reis, Adriano Abbud, Adriana Bugno
EPI_ISL_14772913	Vigilancia Epidemiologica Jardinopolis - SP	Instituto Adolfo Lutz Strategic Laboratory	Claudio Tavares Sacchi, Karoline Rodrigues Campos, Ariadne Ferreira Amarante, Marion Benedito Nascimento Santos, Alex Domingos Reis, Adriano Abbud, Adriana Bugno
EPI_ISL_14772914	Pronto Atendimento Infantil e entrai de Quimioterapia Sjrpreto	Instituto Adolfo Lutz Strategic Laboratory	Claudio Tavares Sacchi, Karoline Rodrigues Campos. Ariadne Ferreira Amarante, Marion Benedito Nascimento Santos, Alex Domingos Reis. Adriano Abbud. Adriana Bugno
EPI_ISL_14773001	CEDIC CTA	Instituto Adolfo Lutz Strategic Laboratory	Claudio Tavares Sacchi, Karoline Rodrigues Campos, Ariadne Ferreira Amarante, Marion Benedito Nascimento Santos, Alex Domingos Reis. Adriano Abbud, Adriana Bugno
EPI_ISL_14783237	Sicilian Regional Laboratory - AOUP "P. Giaccone" - University of Palermo	Sicilian Regional Laboratory - AOUP "P. Giaccone" - University of Palermo	Fabio Tramuto, Carmelo Massimo Maida, Giulia Randazzo, Valeria Guzzetta, Walter Mazzucco, Giorgio Graziano, Vincenzo Restivo, Claudio Costantino, Francesco Vitale
EPI_ISL_14786290	IRCCS Sacro Cuore Don Calabria Hospital, Department of Infectious, Tropical Diseases & Microbiology	Department of Infectious. Tropical Diseases & Microbiology,IRCCS Sacra Cuore Don Calabria Hospital	Michela Deiana, Antonio Mori, Concetta Castilletti. Chiara Piubelli, Denise Lavezzari, Elena Pomari
EPI_ISL_l4786346	IRCCS Sacro Cuore Don Calabria Hospital, Department of Infectious, Tropical Diseases & Microbiology	IRCCS Sacro Cuore Oon Calabria Hospital, Department of Infectious, Tropical Diseases & Microbiology	Michela Deiana, Antonio Mori, Concetta Castilletti, Chiara Piubelli, Denise Lavezzari, Elena Pomari
EPI ISL 14793992, EPI ISL 14795058, EPÜSL)4795084, EPI=ISL)4795085, EPI_ISL_l4795259	Erasmus Medical Center Department of Virology	Erasmus Medical Center Department of Virology	Bas Oude Munnink, Leonard Schuele, Marjan Boter, Babette Weller, Babs Verstrepen, Richard Molenkamp, Janette Rahamat-Langendoen, Reina Sikkema, Marion Koopmans

EPI ISL 14804616, EPI ISL 14804617, EPI ISL 14804618, EPI ISL 14804619, EPI ISL 14804620, EPI ISL 14804621. EPI ISL 14804622, EPI ISL 14804623, EPI ISL 14804624, EPI ISL 14804625, EPI ISL 14804626, EPI ISL 14804627, EPI ISL 14804628, EPI ISL 14804629, EPI ISL 14804630, EPI ISL 14804631. EPI ISL 14804632, EPI ISL 14804633, EPI ISL 14804634, EPI ISL 14804635, EPI=ISL)4804636			
see above	Centre for Biological Threats, Highly Pathogenic Viruses, Robert Koch Institute	Centre for Biological Threats, Highly Pathogenic Viruses, Robert Koch Institute	Brinkmann,A., Kohl,C., Pape,K., Uddin,S., Schrick,L., Michel,)., Schaade,L., Nitsche,A.
EPI_ISL_l4804638, EPI_JSL_l4804639, EPI ISL 14804640, EPI ISL 14804641, EPI-ISL-14804642, EPI-ISL-14804643, EPÜSL)4804644, EPCISL)4804645, EPI_ISL_l4804646, EPI_ISL_l4804647	Nebraska Public Health Laboratory	University of Nebraska Medical Center, Oklahoma Pathogen Genomics Consortium	Chapman,R.C., Bernhard,K., McCutchen,E.L., Fauver,J.R., O'Dell,J.X., Mannell,M., Wiley,M.R., Cross,S.T.
EPI_ISL_l4809096	AMA Capao Redonda	Instituto Adolfo Lutz Strategic Laboratory	Claudio Tavares Sacchi, Karoline Rodrigues Campos, Ariadne Ferreira Amarante, Marion Benedito Nascimento Santos, Alex Oomingos Reis, Adriano Abbud, Adriana Bugna
EPI_ISL_l4809097	Pronto Socorro Municipal de Taubate	Instituto Adolfo Lutz Strategic Laboratory	Claudio Tavares Sacchi, Karoline Rodrigues Campos, Ariadne Ferreira Amarante. Marion Benedito Nascimento Santos, Alex Domingos Reis. Adriano Abbud. Adriana Bugna
EPI_ISL_l4809098	Laboratorio Municipal de Piracicaba	Instituto Adolfo Lutz Strategic Laboratory	Claudio Tavares Sacchi, Karoline Rodrigues Campos, Ariadne Ferreira Amarante, Marion Benedito Nascimento Santos, Alex Domingos Reis, Adriano Abbud, Adriana Bugna
EPI_ISL_14809099	Centra de Saude Gabriel de Lara	Instituto Adolfo Lutz Strategic Laboratory	Claudio Tavares Sacchi, Karoline Rodrigues Campos, Ariadne Ferreira Amarante, Marion Benedito Nascimento Santos, Alex Domingos Reis, Adriano Abbud, Adriana Bugna
EPI_ISL_l4809100	Secretaria Municipal da Saude de Joanopolis	Instituto Adolfo Lutz Strategic Laboratory	Claudio Tavares Sacchi, Karoline Rodrigues Campos, Ariadne Ferreira Amarante, Marion Benedito Nascimento Santos, Alex Oomingos Reis, Adriano Abbud, Adriana Bugna
EPI ISL 14810370, EPI ISL 14810404, EPI=ISL=l4810405, EPI=JSL)4810406, EPI_ISL_l4810407	Erasmus Medical Center Department of Virology	Erasmus Medical Center Department of Virology	Leonard Schuele, Bas Oude Munnink, Marjan Boter, Babette Weller, Babs Verstrepen, Richard Molenkamp, Janette Rahamat-Langendoen, Reina Sikkema, Marion Koopmans
EPI_ISL_l4810460	Rapid Respose Lab, Pasteur Institute of Iran	Rapid Respose Lab, Pasteur Institute of Iran	Mahsa Tavakoli. Zahra Fereydouni, Zahra Ahmadi. Amir Hesam Nemati, Setareh Kashanian, Zahra Hosseini, Laya Farhan Asadi, Jahangir Rezaie, Parastoo Yektay Sanati, Farideh Niknarn Oskouei, Tahrnineh Jalali, Mohamrnad Hassan Pouriayevali, Keyhan Azadmanesh, Arash Arashkia, Ahmad Adeli, Ali Maleki, Mahdi Rohani, Mostafa Salehi-Vaziri
EPI ISL 14818584, EPI ISL 14818585, EPI ISL 14818586, EPI ISL 14818587, EPI ISL 14818588, EPI ISL 14818589, EPI ISL 14818590, EPI ISL 14818591, EPI ISL 14818592, EPI ISL 14818593, EPI ISL 14818594, EPI ISL 14818595, EPI ISL 14818596, EPI ISL 14818597, EPI ISL 14818598, EPI ISL 14818599, EPI ISL 14818600, EPI ISL 14818601, EPI ISL 14818602, EPI ISL 14818603, EPI=ISL)4818605, EPI=ISL)4818606, EPÜSL)4818607, EPCISL)4818608, EPI=ISL)4818609, EPI=ISL)4818610, EPI=ISL)4818611, EPÜSL)4818614, EPI=ISL)4818615, EPI=ISL)4818616, EPI=ISL)4818617			
see above	Los Angeles County Public Health Laboratories	Los Angeles County Public Health Laboratories	P. Hemarajata et al.
EPI ISL 14818783, EPI ISL 14818784, EPI ISL 14818785, EPI ISL 14818786, EPI ISL 14818787, EPI ISL 14818788, EPI ISL 14818789, EPI ISL 14818790, EPI ISL 14818791, EPI ISL 14818792, EPI ISL 14818793, EPI ISL 14818794, EPI ISL 14818795, EPI ISL 14818796, EPI ISL 14818797, EPI ISL 14818798, EPI ISL 14818799, EPI ISL 14818800, EPI ISL 14818801, EPI ISL 14818802, EPI=ISL)4818803, EPI=ISL)4818804, EPÜSL)4818805, EPI=ISL)4818806, EPI=ISL)4818807, EPI=ISL)4818808, EPI=ISL)4818809, EPÜSL)4818810, EPI=ISL)4818811, EPI=ISL)4818812, EPI=ISL)4818813, EPI=ISL)4818814, EPÜSL)4818815, EPI=ISL)4818817, EPI=ISL)4818818, EPI=ISL)4818819, EPI=ISL)4818820, EPÜSL)4818821, EPI=ISL)4818822			
see above	Laboratorio de Referencia Nacional de Virus Inmunoprevenibles. Centra Nacional de Salud Publica. Instituto Nacional de Salud	Laboratorio de Referencia Nacional de Virus Inmunoprevenibles. Centra Nacional de Salud Publica. Instituto Nacional de Salud.	Carlos Padilla Rojas, Veronica Hurtado Vela, Iris Silva Molina, Luren Sevilla Castarieda, Victor Jimenez Vasquez, Luis Barcena Flores, Alicia Nuriez Llanos, Kelly Izarra Rojas, Karla Vasquez Cajachahua, Estela Huaman Angeles, Jorge Giralda Chavez, Lilian Huarca Balbin, Maria Sandra Villar Saavedra, Henri Bailon Calderon, Lely Solari zerpa, Gloria Arotinco Garayar. Equipa de vigilancia genomica del Instituto Nacional de Salud.
EPI_ISL_l4835894, EPI_JSL_l4835895, EPI_ISL_l4835897	Ouest Diagnostics Nichais Institute	Los Angeles County Public Health Laboratories	P. Hernarajata et al.
EPI_ISL_l4838586, EPI_ISL_l4838587, EPI_JSL_l4838588, EPI_ISL_l4838589	Pathogen Genomics Lab, National Institute for Biomedical Research (INRB)	Pathogen Genomics Lab. National Institute for Biomedical Research (INRB)	Placide Mbala-Kingebeni, Eddy Kinganda-Lusamaki, Adrienne Amuri-Aziza, Elisabeth Pukuta, Catherine Pratt, Nicolas Fernandez, Emmanuel Lokilo Lofiko, Gradi Luakanda Ndelemo, Francisca Muyernbe Mawete, Jean Claude Makangara Cigolo, Elisabeth Muyamuna, Raphaël Lumembe Numbi, Gabriel Kabamba Lungenyi, Prince Akil Sandali, Pauline Musuamba Kayembe, Rilia Ola Mpumbe, Emile Malembi, Emmanuel Hasivirwe Vakaniaki, Andrew Rambaut, Nick Loman, Kristian Andersen, Michael Wiley, Ahidjo Ayouba, Steve Ahuka-Mundeke, Martine Peeters, Eric Delaporte, Jean-Jacques Muyernbe Tamfurn
EPI_ISL_l4842151	Division of High Consequence Pathogens and Pathology (DHCPP)-PRB, CDC	Division of High Consequence Pathogens and Pathology {DHCPP)-PRB. CDC	Gigante,C.M., Hauser,J.R., Zhao,H., Batra,o., Hetrick,E.E., Howard,D.T., Kovar,L., Seabolt,M.H., Weigand,M.R., Burroughs,M., Lee,J., Wilkins,K., McCollum,A., Hutson,C., Davidson,w., Rao.A., Mangla,A. and Li,Y.
EPI_ISL_l4842152	Division of High Consequence Pathogens and Pathology (DHCPP)-PRB, CDC	Division of High Consequence Pathogens and Pathology {DHCPP)-PRB. CDC	Gigante,C.M., Pavlick,J., Zhao,H., Batra,D., Hetrick,E.E., Howard,D.T., Kovar,L., Seabolt,M.H., Weigand,M.R., Burroughs,M., Lee,J., Wilkins,K., McCollurn,A., Hutson,C., Davidson,W., Rao.A., Parrott,T. and Li,Y.
EPI_ISL_l4842153, EPI_ISL_l4842154	Division of High Consequence Pathogens and Pathology (DHCPP)-PRB, CDC	Division of High Consequence Pathogens and Pathology {DHCPP)-PRB, CDC	Gigante,C.M., Hauser.J.R., Zhao,H.. Batra.D., Hetrick,E.E., Howard,D.T., Kovar,L., Seabolt,M.H., Weigand.M.R., Burroughs,M.. lee,J.. Wilkins,K.. McCollurn,A.. Hutson,C.. Davidson,W., Rao.A., Mangla,A. and Li.Y.
EPI ISL 14842155, EPI ISL 14842156, EPI_ISL_l48i215-7	Division of High Consequence Pathogens and Pathology (DHCPP)-PRB, CDC	Division of High Consequence Pathogens and Pathology (DHCPP)-PRB, CDC	Gigante,C.M., Pavlick,J., Zhao,H., Batra,D., Hetrick,E.E.. Howard,D.T., Kovar,L., Seabolt,M.H., Weigand,M.R., Burroughs,M., Lee,)., Wilkins,K., McCollum,A., Hutson,C., Davidson,W., Rao,A., Parrott,T. and Li,Y.
EPI_ISL_l4842158	Division of High Consequence Pathogens and Pathology (DHCPP)-PRB, CDC	Division of High Consequence Pathogens and Pathology (DHCPP)-PRB, CDC	Gigante,C.M., Hughes,S., Zhao,H., Batra,O., Hetrick,E.E., Howard,D.T., Kovar,L., Seabolt,M.H., Weigand,M.R., Burroughs,M., Lee,J., Wilkins,K., McCollum,A., Hutson,C., Davidson,W., Rao.A., Baumgartner,J. and Li,Y
EPI_ISL_l4842159	Division of High Consequence Pathogens and Pathology (DHCPP)-PRB, CDC	Division of High Consequence Pathogens and Pathology {DHCPP)-PRB. CDC	Gigante,C.M., Johnson,S., Zhao,H., Batra,D., Hetrick,E.E., Howard,D.T., Kovar,L., Seabolt,M.H., Weigand,M.R., Burroughs,M., Lee,J., Wilkins,K., McCollum,A., Hutson,C., Davidson,W., Rao.A., Riner,D. and Li,Y.
EPI_ISL_l4842160	Division of High Consequence Pathogens and Pathology (DHCPP)-PRB, CDC	Division of High Consequence Pathogens and Pathology {DHCPP)-PRB. CDC	Gigante,C.M., Manuzak,A., Zhao,H., Batra,D., Hetrick,E.E., Howard,D.T., Kovar,L., Seabolt.M.H., Weigand,M.R., Burroughs,M., Lee,J., Wilkins,K., McCollum,A., Hutson,C., Davidson,w., Rao.A., Gose,R. and Li,Y.
EPI_ISL_l4842161, EPI_ISL_l4842162	Division of High Consequence Pathogens and Pathology (DHCPP)-PRB, CDC	Division of High Consequence Pathogens and Pathology {DHCPP)-PRB, CDC	Gigante,C.M., Kubin,G., Zhao,H., Batra,D., Hetrick,E.E., Howard,D.T., Kovar,L., Seabolt,M.H., Weigand,M.R., Burroughs,M., Lee,J., Wilkins,K., McCollum,A., Hutson,C., Davidson,W., Rao,A., White,S.L. and Li,Y.
EPI_ISL_l4842163	Division of High Consequence Pathogens and Pathology (DHCPP)-PRB, CDC	Division of High Consequence Pathogens and Pathology {DHCPP)-PRB, CDC	Gigante,C.M., Ghinai.l., Zhao.H., Batra,D.. Hetrick,E.E.. Howard,D.T.. Kovar.l.. Seabolt.M.H., Weigand,M.R., Burroughs.M.. Lee.J.. Wilkins,K., McCollum.A., Hutson.C., Davidson.W., Rao.A., Kerins.J. and LLY
EPI_JSL_l4842164, EPI_ISL_l4842165	Division of High Consequence Pathogens and Pathology (DHCPP)-PRB, CDC	Division of High Consequence Pathogens and Pathology (DHCPP)-PRB, CDC	Gigante,C.M., Griffin-Thomas,L., Zhao,H., Batra,O., Hetrick,E.E., Howard,D.T., Kovar,L., Seabolt,M.H., Weigand,M.R., Burroughs,M., Lee,J., Wilkins,K., McCollum,A., Hutson,C., Davidson,W., Rao.A., Crain,). and Li,Y.
EPI_ISL_l4842166	Division of High Consequence Pathogens and Pathology (DHCPP)-PRB, CDC	Division of High Consequence Pathogens and Pathology (DHCPP)-PRB, CDC	Gigante,C.M., Xia,D., Zhao,H., Batra,O., Hetrick,E.E., Howard,D.T., Kovar,L., Seabolt.M.H., Weigand,M.R., Burroughs,M., Lee,J., Wilkins,K., McCollum,A., Hutson,C., Davidson,W., Rao.A., Plipat,N. and Li,Y.
EPI_ISL_l4842167, EPI_ISL_l4842168	Division of High Consequence Pathogens and Pathology (DHCPP)-PRB, CDC	Division of High Consequence Pathogens and Pathology {DHCPP)-PRB. CDC	Gigante,C.M., Griffin-Thornas,L., Zhao,H., Batra,o., Hetrick,E.E., Howard,D.T., Kovar,L., Seabolt,M.H., Weigand,M.R., Burroughs,M., Lee,J., Wilkins,K., McCollum,A., Hutson,C., Davidson,w., Rao.A., Crain,). and Li,Y.
EPI ISL 14863040, EPI ISL 14863041, EPJ-ISL-14863042, EPI-JSL-14863043, EPI-ISL-14863044, EPI-ISL-14863045, EP1-=_1sL-=_14863046, EPI_isL-=_14863047	Molecular Epiderniology, Idaho Bureau of Laboratories	Molecular Epidemiology, Idaho Bureau of Laboratories	Ceniseros,A.
EPI_ISL_14863048	MEPHI, IHU - Mediterranee Infection	MEPHI, IHU - Mediterranee Infection	Colson,P.
EPI_ISL_l4863049	Molecular Epidemiology, Idaho Bureau of Laboratories	Molecular Epidemiology, Idaho Bureau of Laboratories	Ceniseros,A.
EPI_ISL_l4863050, EPI_ISL_l4863051. EPJ_ISL_14863052, EPI_JSL_l4863053, EPI_ISL_14863054, EPI_ISL_l4863055, EPI_ISL_l4863057, EPI_ISL_14863058, EPI_ISL_l4863059, EPI_ISL_14863060, EPI_ISL_l4863061. EPI_ISL_l4863062, EPI_ISL_14863064, EPI_ISL_l4863065			
see above	MEPHJ, IHU - Mediterranee Infection	MEPHI, IHU - Mediterranee Infection	Colson.P.
EPI_ISL_14865785	UBSJ COPA	Instituto Adolfo Lutz Strategic Laboratory	Claudio Tavares Sacchi, Karoline Rodrigues Campos, Ariadne Ferreira Amarante, Marion Benedito Nascimento Santos, Alex Domingos Reis, Adriano Abbud, Adriana Bugna
EPI_ISL_l4866481	PR S da Familia Unidade de Saude Adalberto Rocha	Instituto Adolfo Lutz Strategic Laboratory	Claudio Tavares Sacchi, Karoline Rodrigues Campos, Ariadne Ferreira Amarante, Marion Benedito Nascimento Santos, Alex Domingos Reis, Adriano Abbud, Adriana Bugna
EPI_ISL_14866751	Pronto Socorro da Vila Oirce	Instituto Adolfo Lutz Strategic Laboratory	Claudio Tavares Sacchi, Karoline Rodrigues Campos, Ariadne Ferreira Amarante, Marion Benedito Nascimento Santos, Alex Domingos Reis, Adriano Abbud, Adriana Bugna
EPI_I5L_l4866752	Secretaria Municipal de Saude Sao Carlos	Instituto Adolfo Lutz Strategic Laboratory	Claudio Tavares Sacchi, Karoline Rodrigues Campos, Ariadne Ferreira Amarante, Marion Benedito Nascimento Santos, Alex Oomingos Reis, Adriano Abbud, Adriana Bugna
EPI_ISL_l4887952, EPI_ISL_14887953, EPJ_ISL_14887954. EPI_JSL_l4887957, EPI_ISL_14887958, EPI_ISL_l4887959. EPI_ISL_14887960, EPI_ISL_14887961. EPI_ISL_l4887962. EPI_ISL_14887963, EPI_ISL_l4887964. EPI_ISL_14887965, EPI_ISL_14887966. EPI_ISL_l4887967. EPI_ISL_14887968, EPI_ISL_l4887970. EPI_ISL_14887971. EPI_ISL_14887972, EPI_ISL_l4887974, EPI_ISL_14887975, EPI_ISL_l4887976, EPI_ISL_l4887977, EPI_ISL_14887978, EPI_ISL_l4887979, EPI_ISL_14887980, EPI_ISL_l4887981, EPI_ISL_l4887982, EPI_ISL_14887983, EPI_ISL_l4887984, EPI_ISL_14887985, EPI_ISL_l4887986, EPI_ISL_l4887987, EPI_ISL_14887988, EPI_ISL_l4887989, EPI_ISL_14887990			
see above	Viral Genotyping Reference Laboratory, Royal Infirmary of Edinburgh	Viral Genotyping Reference Laboratory, Royal Infirrnary of Edinburgh	McHugh,M.P., Maloney,D., Parker.A., Mathers,K., Dewar,R., Kenicer,J., Cotton,S., Wild,J. and Templeton,K.E.
EPI ISL 14910863, EPI ISL 14910864, EPI ISL 14910865, EPI ISL 14910866, EPI ISL 14910867, EPI ISL 14910868, EPI ISL 14910869, EPI ISL 14910870, EPI ISL 14910871, EPI ISL 14910872, EPI ISL 14910873, EPI ISL 14910874, EPI ISL 14910875, EPI ISL 14910876, EPI ISL 14910877, EPI ISL 14910878, EPI ISL 14910879, EPI ISL 14910880, EPI ISL 14910881, EPI ISL 14910882, EPI=ISL)4910883, EPI=ISL)4910884, EPÜSL)4910885			
seeabove	Laboratory Medicine, UW Virology	Laboratory Medicine, UW Virology	Sereewit,J., Xie,H., Roychoudhury,P. and Greninger,A.L.
EPI_ISL_l4910886	Research and Evaluation, UKHSA	Research and Evaluation, UKHSA	Burton,)., Easterbrook,l., Orinkwater,E, Groves,N., Osman,K.L., Lewandowski,K.S., Carter,☐., Pullan,S.T., Myers,R., Vipond,R. and Chand,M.
EPI_ISL_l4917557, EPI_ISL_14917558, EPJ_ISL_14917559, EPI_JSL_l4917560, EPI_ISL_14917561, EPI_ISL_l4917562, EPI_ISL_l4917563, EPI_ISL_14917564, EPI_ISL_l4917565, EPI_ISL_14917566, EPI_ISL_l4917567, EPI_ISL_14917568, EPI_ISL_14917569, EPI_ISL_l4917570, EPI_ISL_14917571, EPI_ISL_l4917572, EPI_ISL_14917573, EPI_ISL_14917574			
see above	Los Angeles County Public Health Laboratories	Los Angeles County Public Health Laboratories	P. Hernarajata et al.
EPI_ISL_14917575	Ouest Diagnostics Nichais Institute	Los Angeles County Public Health Laboratories	P. Hernarajata et al.
EPI_ISL_l4917576	Los Angeles County Public Health Laboratories	Los Angeles County Public Health Laboratories	P. Hemarajata et al.
EPI_ISL_l4917577, EPI_ISL_14917578	Ouest Diagnostics Nichais Institute	Los Angeles County Public Health Laboratories	P. Hemarajata et al.
EPI_JSL_l4917580, EPI_ISL_14917581	Los Angeles County Public Health Laboratories	Los Angeles County Public Health Laboratories	P. Hernarajata et al.
EPI_ISL_14917582	Ouest Diagnostics Nichais Institute	Los Angeles County Public Health laboratories	P. Hernarajata et al.
EPJ_ISL_l4917583, EPI_ISL_l4917585, EPJ_ISL_14917586, EPI_ISL_l4917587, EPI_ISL_14917588	Los Angeles County Public Health Laboratories	Los Angeles County Public Health Laboratories	P. Hernarajata et al.
EPI ISL 14923900, EPI ISL 14923901, EPÜSL)4923902, EPI=ISL)4923903,	Research and Evaluation, UKHSA	Research and Evaluation, UKHSA	Groves,N., Osman,K.L., Lewandowski,K.S., Carter,O.P., Pullan,S.T., Myers,R., Vipond,R. and Chand,M.

EPI_ISL_l4923904, EPI_ISL_14923905			
EPI_ISL_l4934116	Medical Center of Vienna Center for Virology	Medical University of Vienna Center for Virology	Jeremy V. Camp, Monika Redlberger-Fritz, Stephan W. Aberle
EPI_ISL_l4934140	Center for Viralogy Medical Unviersity of Vienna	Medical University of Vienna Center for Viralogy	Jeremy V. Camp, Monika Redlberger-Fritz, Stephan W. Aberle
EPI_ISL_l4934382	Medical University of Vienna Center for Viralogy	Medical University of Vienna Center for Viralogy	Jeremy V. Camp, Monika Redlberger-Fritz, Stephan W. Aberle
EPI_ISL_l4934478	Medical University of Vienna Center for Virology	Medical University of Vienna Center for Virology	Jeremy V. Camp, Monika Redlberg-Fritz, Stephan W. Aberle
EPI_ISL_l4934480, EPI_ISL_l4934481, EPI_ISL_l4934482, EPI_ISL_l4934483, EPI_ISL_l4934484, EPI_ISL_l4934485, EPI_ISL_l4934486, EPI_ISL_l4934487, EPI_ISL_l4934488, EPI_ISL_l4934489, EPI_ISL_l4934490, EPI_ISL_l4934491, EPI_ISL_l4934492, EPI_ISL_l4934493, EPI_ISL_l4934494, EPI_ISL_l4934495			
see above	Research and Evaluation, UKHSA	Research and Evaluation, UKHSA	Groves,N., Osman,K.L, Lewandowski,K.S.. Carter,D.P.. Pullan.S.T., Myers,R.. Vipond,R. and Chand,M.
EPI_ISL_l4934496, EPI_ISL_l4934497, EPI_ISL_l4934498, EPI_ISL_l4934499, EPI_ISL_l4934500, EPI_ISL_l4934501. EPI_ISL_l4934502, EPI_ISL_l4934503, EPI_ISL_l4934505, EPI_ISL_l4934506, EPI_ISL_l4934507, EPI_ISL_l4934510, EPI_ISL_l4934511, EPI_ISL_l4934512, EPI_ISL_l4934513, EPI_ISL_l4934514. EPI_ISL_l4934515, EPI_ISL_l4934517, EPI_ISL_l4934518, EPI_ISL_l4934519, EPI ISL 14934520, EPI ISL 14934521. EPI ISL 14934522, EPI ISL 14934523, EPI ISL 14934524, EPI ISL 14934525, EPI ISL 14934526, EPI ISL 14934527, EPI ISL 14934528, EPI ISL 14934529, EPI ISL 14934530, EPI ISL 14934531. EPI ISL 14934534, EPI ISL 14934535, EPI ISL 14934536, EPI ISL 14934537, EPI ISL 14934538, EPI ISL 14934539, EPI ISL 14934540, EPI ISL 14934541. EPI-ISL-14934543, EPI-ISL-14934544, EPI-ISL-14934545, EPI-ISL-14934546, EPI-ISL-14934547, EPI-ISL-14934548, EPI-ISL-14934549, EPI-ISL-14934550, EPI-ISL-14934551, EPI-ISL-14934554, EPI-ISL-14934555, EPI-ISL-14934556, EPI-ISL-14934558, EPI-ISL-14934559, EPI-ISL-14934560, EPI-ISL-14934561, EPI-ISL-14934562, EPI-ISL-14934563, EPI-ISL-14934564, EPI-ISL-14934565, EPI-ISL-14934566, EPI-ISL-14934568, EPI-ISL-14934569, EPI-ISL-14934570, EPI-ISL-14934572, EPI-ISL-14934573, EPI-ISL-14934574, EPI-ISL-14934575, EPI-ISL-14934576, EPI-ISL-14934577, EPI-ISL-14934578, EPI-ISL-14934579, EPI-ISL-14934580, EPI-ISL-14934581, EPI-ISL-14934582, EPI-ISL-14934583, EPI-ISL-14934584, EPI-ISL-14934585, EPI-ISL-14934586, EPI-ISL-14934587, EPI=ISL)4934588, EPI=ISL)4934589, EPÜSL)4934608, EPI=ISL)4934609, EPI=ISL)4934611, EPI=ISL)4934612, EPI=ISL)4934613, EPÜSL)4934614, EPI=ISL)4934615, EPI=ISL)4934616, EPI=ISL)4934619 -			
see above	Department of lnfectious Diseases, National Institute of Health Douter Ricardo Jorge, Portugal (INSA)	Department of lnfectious Diseases, National Institute of Health Doutor Ricardo Jorge, Portugal (INSA)	Isidro,)., Borges,V., Pinto,M., Sobral,D.. Santos,)., Nunes,A.. Mixao,V., Ferreira,R., Santos,D., Duarte,S., Vieira,L.. Borrego,M.J.. Nuncio,S.. Lapes de Carvalho,I.. Pelerito,A., Cordeiro,R. and Gomes,J.P.
EPI ISL 14934620, EPI ISL 14934621, EPJ ISL 14934622, EPI JSL 14934623, EPI ISL 14934624, EPI ISL 14934626, EPI ISL 14934627, EPI ISL 14934628, EPI ISL 14934629, EPI ISL 14934630, EPI ISL 14934631, EPI ISL 14934632, EPI ISL 14934633, EPI ISL 14934634, EPI ISL 14934635, EPI ISL 14934636, EPI ISL 14934637, EPI ISL 14934638, EPI ISL 14934639, EPI ISL 14934640, EPI-ISL-14934641. EPI-ISL-14934642, EPI-ISL-14934643, EPI-ISL-14934644, EPI-ISL-14934645, EPI-ISL-14934646, EPI-ISL-14934647, EPI-ISL-14934648, EPI-ISL-14934649, EPI-ISL-14934650, EPI-ISL-14934651. EPI-ISL-14934652, EPI-ISL-14934653, EPI-ISL-14934654, EPI-ISL-14934655, EPI-ISL-14934656, EPI-ISL-14934657, EPI-ISL-14934658, EPI-ISL-14934659, EPI-ISL-14934661, EPI-ISL-14934662, EPI-ISL-14934663, EPI-ISL-14934664, EPI-ISL-14934665, EPI-ISL-14934666, EPI-ISL-14934667, EPI-ISL-14934668, EPI-ISL-14934669, EPI-ISL-14934670, EPI-ISL-14934671, EPI-ISL-14934672, EPI-ISL-14934673, EPI-ISL-14934674, EPI-ISL-14934675, EPI-ISL-14934677, EPI-ISL-14934678, EPI-ISL-14934679, EPI-ISL-14934680, EPI-ISL-14934681, EPI-ISL-14934682, EPI-ISL-14934683, EPI-ISL-14934684, EPI-ISL-1493468S, EPI-ISL-14934686, EPI-ISL-14934687, EPI-ISL-14934688, EPI-ISL-14934689, EPI-ISL-14934690, EPI-ISL-14934691, EPI-ISL-14934692, EPI-ISL-14934693, EPI-ISL-14934694, EPI-ISL-1493469S, EPI-ISL-14934696, EPI-ISL-14934697, EPI-ISL-14934698, EPI-ISL-14934699, EPI-ISL-14934700, EPI-ISL-14934701, EPI-ISL-14934702, EP1=1sL)4934703, EPC1sL)4934704, EPÜSL)4934705, EPC1sL)4934706, EP1=1sL)4934707 -			
see above	Research and Evaluation, UKHSA	Research and Evaluation, UKHSA	Groves,N., Osman,K.L. Lewandowski,K.S., Carter,O.P., Pullan,S.T., Myers,R., Vipond,R. and Chand,M.
EPI_ISL_14944276, EPI_ISL_14944277, EPI_ISL_14944278, EPI_ISL_14944279, EPI_ISL_14944280, EPI_ISL_14944282, EPI_ISL_14944283, EPI_ISL_14944284, EPI_ISL_14944285, EPI_ISL_14944288, EPI_ISL_14944289, EPI_ISL_14944291, EPI_ISL_14944292, EPI_ISL_14944293, EPI_ISL_14944294			
see above	Rhode Island State Health Laboratory	Rhode Island State Health Laboratory	Kristin Carpenter-Azevedo, Sean Sierra-Patev, Richard C. Huard
EPI_ISL_l4945299	Department of Microbiology, The University of Hong Kong	Department of Microbiology, The University of Hong Kong	Kelvin K.W. To, Kwok-Yung Yuen
EPI_ISL_14952916	Indian Council of Medical Research-National Institute of Viralogy	Indian Council of Medical Research-National Institute of Viralogy	Pragya Yadav, Rima Sahay, Anita Aich Shete, Sreelekshmy Mohandas, Priya Abraham
EPI_ISL_l4961089, EPI_ISL_l4961090	Public Health Authority of the Slovak Republic	Laboratory of Genomics and Bioinformatics, Comenius University Science Park	Tomas Szemes, Edita Staroi'iova, Elena Ticha, Lucia Sevëikova, Terézia Vrabrova, Tatiana SedlèCkova, Miroslav Bôhmer, Jaroslav BudiS, Pavai MiSenko
EPI_ISL_l4962734	Laboratory of Viralogy, University Hospitals of Geneva	Laboratory of Viralogy, University Hospitals of Geneva	Laubscher,F., Chudzinsk,V., Cordey,S., Schibler,M., Kaiser,L. and Renzoni,A.
EPI ISL 14977306, EPI ISL 14977307, EPÜSL)4977308, EPCISL=l4977309, EPI_ISL_l4977310	Environmental, Agricultural, and Occupational Health, University of Nebraska Medical Center	Environmental, Agricultural, and Occupational Health, University of Nebraska Medical Center	Tegomoh,B., Cross,S.T. Chapman,R.C., Bernhard,K., McCutchen,E.L., Fauver,J.R., Pratt,C.B., Warden,D.E., Iwen,P.C, Donahue,M. and Wiley,M.R.
EPI_ISL_l4980972, EPI_ISL_l4981151	Kingston Health Sciences Centre	Kingston Health Sciences Centre	Calvin Sjaarda, Henry Wong, Nick Buchner, Drew Roberts, Phung Ta, Jacob Whalen, Sheri Levesque, Prameet Sheth
EPI_ISL_l4994740	UBS Vila California Zeilival Bruscagin	Instituto Adolfo Lutz Strategic Laboratory	Claudio Tavares Sacchi, Karaline Rodrigues Campos, Ariadne Ferreira Amarante, Marion Benedito Nascimento Santos, Alex Domingos Reis, Adriano Abbud, Adriana Bugna
EPI_ISL_l4995206	Pranto Socorra Municiplal de Cravinhos	Instituto Adolfo Lutz Strategic Laboratory	Claudio Tavares Sacchi, Karaline Rodrigues Campos, Ariadne Ferreira Amarante, Marion Benedito Nascimento Santos, Alex Oomingos Reis, Adriano Abbud, Adriana Bugna
EPI_ISL_l4995578	Hosp. Municipal de Ilhabela Gov. Mario Cavas Jr	Instituto Adolfo Lutz Strategic Laboratory	Claudio Tavares Sacchi, Karoline Rodrigues Campos, Ariadne Ferreira Amarante, Marion Benedito Nascimento Santos, Alex Domingos Reis, Adriano Abbud, Adriana Bugna
EPI_ISL_l4995579	Secretaria Municipal de Saude de Feira de Santana	Instituto Adolfo Lutz Strategic Laboratory	Claudio Tavares Sacchi, Karoline Rodrigues Campos, Ariadne Ferreira Amarante, Marion Benedito Nascimento Santos, Alex Domingos Reis, Adriano Abbud, Adriana Bugna
EPI_ISL_l4995580	UBS Alexander Fleming Simioni	Instituto Adolfo Lutz Strategic Laboratory	Claudio Tavares Sacchi, Karaline Rodrigues Campos, Ariadne Ferreira Amarante, Marion Benedito Nascimento Santos, Alex Domingos Reis, Adriano Abbud, Adriana Bugna
EPI_ISL_l4995581	Secretaria Municipal de Saude Sorocaba	Instituto Adolfo Lutz Strategic Laboratory	Claudio Tavares Sacchi, Karoline Rodrigues Campos, Ariadne Ferreira Amarante, Marion Benedito Nascimento Santos, Alex Oomingos Reis, Adriano Abbud, Adriana Bugna
EPI_ISL_l4995S82	Hosp. Municipla. Dr. Jose de Carvalho Florence	Instituto Adolfo Lutz Strategic Laboratory	Claudio Tavares Sacchi, Karaline Rodrigues Campos, Ariadne Ferreira Amarante, Marion Benedito Nascimento Santos, Alex Domingos Reis, Adriano Abbud, Adriana Bugna
EPI_ISL_l4995583	UBS Agua Rasa	Instituto Adolfo Lutz Strategic Laboratory	Claudio Tavares Sacchi, Karoline Rodrigues Campos, Ariadne Ferreira Amarante, Marion Benedito Nascimento Santos, Alex Domingos Reis, Adriano Abbud, Adriana Bugna
EPI_ISL_l4995585	Pronto Socorro Municipal do Promorar	Instituto Adolfo Lutz Strategic Laboratory	Claudio Tavares Sacchi, Karoline Rodrigues Campos, Ariadne Ferreira Amarante, Marion Benedito Nascimento Santos, Alex Domingos Reis, Adriano Abbud, Adriana Bugna
EPI_ISL_l4995586	UPA Centra	Instituto Adolfo Lutz Strategic Laboratory	Claudio Tavares Sacchi, Karaline Rodrigues Campos. Ariadne Ferreira Amarante. Marion Benedito Nascimento Santos, Alex Domingos Reis. Adriano Abbud, Adriana Bugna
EPI_ISL_l4995587, EPI_ISL_l4995588	Centra de Saude 24 haras	Instituto Adolfo Lutz Strategic Laboratory	Claudio Tavares Sacchi, Karoline Rodrigues Campos, Ariadne Ferreira Amarante, Marion Benedito Nascimento Santos, Alex Oomingos Reis, Adriano Abbud, Adriana Bugno
EPI_ISL_l4995589	Cresser Centra de Referencia da Saûde Sexual e Repradutiva	Instituto Adolfo Lutz Strategic Laboratory	Claudio Tavares Sacchi, Karoline Rodrigues Campos, Ariadne Ferreira Amarante, Marion Benedito Nascimento Santos, Alex Domingos Reis, Adriano Abbud, Adriana Bugno
EPI_ISL_l4995590, EPI_ISL_l4995591	Instituto de Infectologia Emilio Ribas	Instituto Adolfo Lutz Strategic Laboratory	Claudio Tavares Sacchi, Karaline Rodrigues Campos, Ariadne Ferreira Amarante, Marion Benedito Nascimento Santos, Alex Domingos Reis, Adriano Abbud, Adriana Bugno
EPI_ISL_l4995592	Unidade de Pronto Atendimento Cipo	Instituto Adolfo Lutz Strategic Laboratory	Claudio Tavares Sacchi, Karoline Rodrigues Campos, Ariadne Ferreira Amarante, Marion Benedito Nascimento Santos, Alex Domingos Reis, Adriano Abbud, Adriana Bugno
EPI_ISL_l4995593	SAE DST/ Aids Ipiranga Jose Francisco Araujo	Instituto Adolfo Lutz Strategic Laboratory	Claudio Tavares Sacchi, Karoline Rodrigues Campos, Ariadne Ferreira Amarante, Marion Benedito Nascimento Santos, Alex Domingos Reis, Adriano Abbud, Adriana Bugno
EPI_ISL_l4995611	UBS Horta Florestal	Instituto Adolfo Lutz Strategic Laboratory	Claudio Tavares Sacchi, Karaline Rodrigues Campos, Ariadne Ferreira Amarante, Marion Benedito Nascimento Santos, Alex Domingos Reis, Adriano Abbud, Adriana Bugno
EPI_ISL_l4995612	Secretaria Minicipal de Saude de IRECE	Instituto Adolfo Lutz Strategic Laboratory	Claudio Tavares Sacchi, Karaline Rodrigues Campos, Ariadne Ferreira Amarante, Marion Benedito Nascimento Santos, Alex Domingos Reis, Adriano Abbud, Adriana Bugno
EPI_ISL_l4995619	Hosp. Tereza de Lisieux	Instituto Adolfo Lutz Strategic Laboratory	Claudio Tavares Sacchi, Karaline Rodrigues Campos, Ariadne Ferreira Amarante, Marion Benedito Nascimento Santos, Alex Oomingos Reis, Adriano Abbud, Adriana Bugno
EPI_ISL_l4995622	UBS Parque Meia Lua	Instituto Adolfo Lutz Strategic Laboratory	Claudio Tavares Sacchi, Karoline Rodrigues Campos, Ariadne Ferreira Amarante, Marion Benedito Nascimento Santos, Alex Domingos Reis, Adriano Abbud, Adriana Bugno
EPI_ISL_l4995631	UPA Novo Horizonte	Instituto Adolfo Lutz Strategic Laboratory	Claudio Tavares Sacchi, Karaline Rodrigues Campos, Ariadne Ferreira Amarante, Marion Benedito Nascimento Santos, Alex Domingos Reis, Adriano Abbud, Adriana Bugno
EPI_ISL_l4995649	Instituto de Infectologia Emilio Ribas	Instituto Adolfo Lutz Strategic Laboratory	Claudio Tavares Sacchi, Karaline Rodrigues Campos, Ariadne Ferreira Amarante, Marion Benedito Nascimento Santos, Alex Domingos Reis, Adriano Abbud, Adriana Bugno
EPI_ISL_l4995652	Hosp. Or. Osiris Florindo Coelho Ferraz de vasconcelos	Instituto Adolfo Lutz Strategic Laboratory	Claudio Tavares Sacchi, Karoline Rodrigues Campos, Ariadne Ferreira Amarante, Marion Benedito Nascimento Santos, Alex Domingos Reis, Adriano Abbud, Adriana Bugno
EPI_ISL_l4995653	Unidade Basica de Saude Vila Cristina	Instituto Adolfo Lutz Strategic Laboratory	Claudio Tavares Sacchi, Karaline Rodrigues Campos, Ariadne Ferreira Amarante, Marion Benedito Nascimento Santos, Alex Domingos Reis, Adriano Abbud, Adriana Bugno
EPI_ISL_l4995723	Unidade Mista de Atendimento Infantil Carapicuiba	Instituto Adolfo Lutz Strategic Laboratory	Claudio Tavares Sacchi, Karoline Rodrigues Campos, Ariadne Ferreira Amarante, Marion Benedito Nascimento Santos, Alex Domingos Reis, Adriano Abbud, Adriana Bugna
EPI_ISL_l4995724	Hosp. Carlos Chagas	Instituto Adolfo Lutz Strategic Laboratory	Claudio Tavares Sacchi, Karoline Rodrigues Campos, Ariadne Ferreira Amarante, Marion Benedito Nascimento Santos, Alex Domingos Reis, Adriano Abbud, Adriana Bugno
EPI_ISL_l4997063, EPI_ISL_l4997064, EPJ_ISL_l4997065, EPI_JSL_l4997066, EPI_ISL_l4997067, EPI_ISL_l4997068, EPI_ISL_l4997069, EPI_ISL_l4997070, EPI_ISL_l4997071, EPI_ISL_l4997072, EPI_ISL_l4997073, EPI_ISL_l4997074			
see above	Laboratory Medicine, UW Viralogy	Laboratory Medicine, UW Viralogy	Sereewit,J., Xie,H., Roychoudhury,P. and Greninger,A.L.
EPI_ISL_15003284, EPI_ISL_15003285, EPI_ISL_15003286, EPI_ISL_15003287, EPI_ISL_15003288, EPI_ISL_15003289, EPI_ISL_15003291, EPI_ISL_15003293, EPI_ISL_15003294, EPI_ISL_15003295, EPI_ISL_15003296, EPI_ISL_15003297			
see above	Rhode Island State Health Laboratory	Rhode Island State Health Laboratory	Kristin Carpenter-Azevedo, Sean Sierra-Patev, Richard C. Huard
EPI_I5L_l5005641	Chongqing Municipal Center for Disease Contrai and Prevention	Chongqing Municipal Center for Disease Contrai and Prevention	Sheng Ye, Yun Tang, Shuang Chen, Mingyue Wang, Zhangping Tan, Zhen Yu
EPI ISL 15008574, EPI ISL 15008575, EP1-=.1sL-=.15008576, EPl,1sL-=.1s008577	Indian Council of Medical Research-National Institute of Viralogy	Indian Council of Medical Research-National Institute of Viralogy	Pragya Yadav, Rima Sahay, Anita Aich Shete, Sreelekshmy Mohandas, Priya Abraham
EPI_ISL_l5016104, EPI_ISL_l5016105	Centers for Disease Contrai & Prevention (CDC), Division of High Consequence Pathogens and Pathology (DHCPP-PRB)	Centers for Disease Contrai & Prevention {CDC). Division of High Consequence Pathogens and Pathology (DHCPP-PRB)	Gigante.CM., Kubin,G., Zhao,H., Batra,D., Hetrick,E.E., Howard,D.T., Kovar,L., Seabolt.M.H., Weigand,M.R., Burroughs,M., Lee,J., Wilkins,K., McCollum,A., Hutson,C., Davidson,w., Rao.A., White,S.L. and Li,Y.
EPI_ISL_15016106	Centers for Disease Contrai & Prevention (CDC), Division of High Consequence Pathogens and Pathology (DHCPP-PRB)	Centers for Disease Contrai & Prevention {CDC). Division of High Consequence Pathogens and Pathology (DHCPP-PRB)	Gigante,C.M., Goldoft,M., Zhao,H., Batra,D., Hetrick,E.E., Howard,D.T., Kovar,L., Seabolt,M.H., Weigand,M.R., Burroughs,M., Lee,)., Wilkins,K.. McCollum,A., Hutson,C., Davidson,W., Rao.A., Holshue,M. and Li.Y.
EPI_ISL_15016107	Centers for Disease Contrai & Prevention (CDC), Division of High Consequence Pathogens and Pathology (DHCPP-PRB)	Centers for Disease Contrai & Prevention {CDC). Division of High Consequence Pathogens and Pathology (DHCPP-PRB)	Gigante,C.M., Thomas,L., Zhao,H., Batra,D., Hetrick,E.E., Howard,D.T., Kovar,L., Seabolt,M.H., Weigand,M.R., Burraughs,M., Lee,J., Wilkins.K., McCollum,A., Hutson,C., Davidson,W., Rao.A., Dunn,J. and Li,Y.
EPI_ISL_l5016108, EPI_ISL_l5016109	Centers for Disease Contrai & Prevention (CDC), Division of High Consequence Pathogens and Pathology (DHCPP-PRB)	Centers for Disease Contrai & Prevention (CDC). Division of High Consequence Pathogens and Pathology (DHCPP-PRB)	Gigante,C.M., Buttery,E., Zhao,H., Batra,D., Hetrick,E.E., Howard,D.T., Kovar,L., Seabolt,M.H., Weigand,M.R., Burroughs,M., Lee,J., Wilkins,K., McCollum,A., Hutson,C., Davidson,W., Rao.A., Raman,□.and Li.Y.
EPI_ISL_15016110	Centers for Disease Contrai & Prevention (CDC), Division of High Consequence Pathogens and Pathology (DHCPP-PRB)	Centers for Disease Contrai & Prevention {CDC). Division of High Consequence Pathogens and Pathology (DHCPP-PRB)	Gigante,C.M., Kubin,G., Zhao,H., Batra,D., Hetrick,E.E., Howard,D.T., Kovar,L., Seabolt,M.H., Weigand,M.R., Burroughs,M., Lee,J., Wilkins,K., McCollum,A., Hutson,C., Davidson,W., Rao.A., White,S.L. and Li.Y.
EPI_ISL_15016111	Centers for Disease Contrai & Prevention (CDC), Division of High Consequence Pathogens and Pathology (DHCPP-PRB)	Centers for Disease Contrai & Prevention (CDC). Division of High Consequence Pathogens and Pathology (DHCPP-PRB)	Gigante,C.M., Thomas,L., Zhao,H., Batra,D., Hetrick,E.E., Howard,D.T., Kovar,L., Seabolt.M.H., Weigand,M.R., Burroughs,M., Lee,J., Wilkins,K., McCollum,A., Hutson,C., Davidson,W., Rao.A., Dunn,J. and Li.Y.

EPI_ISL_15016112	Centers for Disease Contrai & Prevention (CDC), Division of High Consequence Pathogens and Pathology (DHCPP-PRB)	Centers for Disease Contrai & Prevention (CDC). Division of High Consequence Pathogens and Pathology (DHCPP-PRB)	Gigante,C.M., Pettit,D., Zhao,H., Batra,D., Hetrick,E.E., Howard,D.T., Kovar,L., Seabolt,M.H., Weigand,M.R., Burroughs,M., Lee,J., Wilkins,K., McCollum,A., Hutson,C., Davidson,W., Rao.A., Deutsch-Feldman,M. and li,Y.
EPI_ISL_15016113	Centers for Disease Contrai & Prevention (CDC), Division of High Consequence Pathogens and Pathology (DHCPP-PRB)	Centers for Disease Contrai & Prevention {CDC). Division of High Consequence Pathogens and Pathology (DHCPP-PRB)	Gigante,C.M., Ghinai.l., Zhao.H., Batra,D.. Hetrick,E.E., Howard,D.T.. Kovar.L.. Seabolt.M.H., Weigand,M.R., Burroughs,M., Lee,J., Wilkins,K., McCollum.A., Hutson,C., Davidson,W., Rao.A., Kerins,J. and Li.Y
EPI_JSL_15016114, EPI_ISL_15016115	Centers for Disease Contrai & Prevention (CDC), Division of High Consequence Pathogens and Pathology (DHCPP-PRB)	Centers for Disease Contrai & Prevention (CDCl. Division of High Consequence Pathogens and Pathology (DHCPP-PRB)	Gigante,C.M., Thomas,L., Zhao,H., Batra,D., Hetrick,E.E., Howard,D.T., Kovar,L., Seabolt.M.H., Weigand,M.R., Burroughs,M., Lee,J., Wilkins,K., McCollum,A., Hutson,C., Davidson,W., Rao.A., Dunn,J. and Li,Y.
EPI_ISL_15016116	Centers for Disease Contrai & Prevention (CDC), Division of High Consequence Pathogens and Pathology (DHCPP-PRB)	Centers for Disease Contrai & Prevention {CDC). Division of High Consequence Pathogens and Pathology (DHCPP-PRB)	Gigante,C.M., Xia,D., Zhao,H., Batra,D., Hetrick,E.E., Howard,D.T., Kovar,L., Seabolt.M.H., Weigand,M.R., Burroughs,M., Lee,J., Wilkins,K., McCollum,A., Hutson,C., Davidson,W., Rao.A., Plipat.N. and Li.Y.
EPI_ISL_l5016117	Centers for Disease Contrai & Prevention (CDC), Division of High Consequence Pathogens and Pathology (DHCPP-PRB)	Centers for Disease Contrai & Prevention {CDC). Division of High Consequence Pathogens and Pathology (DHCPP-PRB)	Gigante,C.M., Epie,N., Zhao,H., Batra,D., Hetrick,E.E., Howard,D.T., Kovar,L., Seabolt,M.H., Weigand,M.R., Burroughs,M., Lee,J., Wilkins,K., McCollum,A., Hutson,C., Davidson,W., Rao.A., Perez,T. and Li,Y.
EPI ISL 15016118, EPI ISL 15016119, - - EP1_1sL_15ol61fo	Centers for Disease Contrai & Prevention (CDC), Division of High Consequence Pathogens and Pathology (DHCPP-PRB)	Centers for Disease Contrai & Prevention {CDC). Division of High Consequence Pathogens and Pathology (DHCPP-PRB)	Gigante,CM., Acheampong,E., Zhao,H., Batra,D., Hetrick,E.E., Howard,D.T., Kovar,L., Seabolt,M.H., Weigand,M.R., Burroughs,M., Lee,J., Wilkins,K., McCollum,A., Hutson,C, Davidson,W., Rao,A., McDermott.D. and Li,Y.
EPI_ISL_l5016121	Centers for Disease Contrai & Prevention (CDC), Division of High Consequence Pathogens and Pathology (DHCPP-PRB)	Centers for Disease Contrai & Prevention {CDC). Division of High Consequence Pathogens and Pathology (DHCPP-PRB)	Gigante.CM., Pettit,D., Zhao.H., Batra,D.. Hetrick,E.E., Howard.D.T.. Kovar.L.. Seabolt,M.H., Weigand,M.R., Burroughs,M., Lee.J., Wilkins,K., McCollum.A., Hutson,C, Davidson,W., Rao.A., Deutsch-Feldman,M. and Li.Y.
EPI_JSL_15016122, EPI_ISL_15016123	Centers for Disease Contrai & Prevention (CDC), Division of High Consequence Pathogens and Pathology (DHCPP-PRB)	Centers for Disease Contrai & Prevention (CDC). Division of High Consequence Pathogens and Pathology (DHCPP-PRB)	Gigante,C.M., Wang.X., Zhao,H., Batra,D., Hetrick,E.E., Howard,D.T., Kovar,L., Seabolt,M.H., Weigand,M.R., Burroughs,M., Lee,J., Wilkins,K., McCollum,A., Hutson,C., Davidson,W., Rao.A., Ostadkar,R. and Li,Y.
EPI_ISL_15016124	Centers for Disease Contrai & Prevention (CDC), Division of High Consequence Pathogens and Pathology (DHCPP-PRB)	Centers for Disease Contrai & Prevention {CDC). Division of High Consequence Pathogens and Pathology (DHCPP-PRB)	Gigante.CM., Haydel,D., Zhao,H., Batra,D., Hetrick,E.E.. Howard,D.T., Kovar,l., Seabolt,M.H., Weigand,M.R., Burroughs,M., Lee.J., Wilkins.K., McCollum,A., Hutson,C., Davidson,W., Rao.A., Salinas.A. and Li,Y.
EPI_JSL_15016125, EPI_ISL_15016126	Centers for Disease Contrai & Prevention (CDC), Division of High Consequence Pathogens and Pathology (DHCPP-PRB)	Centers for Disease Contrai & Prevention (CDC). Division of High Consequence Pathogens and Pathology (DHCPP-PRB)	Gigante.CM., Pavlick,J., Zhao,H., Batra,D., Hetrick,E.E., Howard,D.T., Kovar,L., Seabolt.M.H., Weigand,M.R., Burroughs,M., Lee,J., Wilkins,K., McCollum,A., Hutson,C., Davidson,W., Rao.A., Parrott,T. and Li,Y.
EPI_ISL_15016127, EPI_ISL_15016128	Centers for Disease Contrai & Prevention (CDC), Division of High Consequence Pathogens and Pathology (DHCPP-PRB)	Centers for Disease Contrai & Prevention {CDC). Division of High Consequence Pathogens and Pathology (DHCPP-PRB)	Gigante.CM., Pearson,C, Zhao,H., Batra,D., Hetrick,E.E., Howard,D.T., Kovar,L., Seabolt,M.H., Weigand,M.R., Burroughs,M., Lee,J., Wilkins,K., McCollum,A., Hutson,C., Davidson,W., Rao.A., Maloney,M. and Li,Y.
EPI_ISL_l5016129	Centers for Disease Contrai & Prevention (CDC), Division of High Consequence Pathogens and Pathology (DHCPP-PRB)	Centers for Disease Contrai & Prevention {CDC). Division of High Consequence Pathogens and Pathology (DHCPP-PRB)	Gigante.CM., Hauser,J.R., Zhao,H., Batra,D., Hetrick,E.E., Howard,D.T., Kovar,l., Seabolt,M.H., Weigand,M.R., Burroughs,M., Lee,J., Wilkins,K., McCollum,A., Hutson,C, Davidson,W., Rao,A., Mangla,A. and Li,Y.
EPI_ISL_15016130	Centers for Disease Contrai & Prevention (CDC), Division of High Consequence Pathogens and Pathology (DHCPP-PRB)	Centers for Disease Contrai & Prevention (CDC). Division of High Consequence Pathogens and Pathology (DHCPP-PRB)	Gigante.CM., Pearson,C, Zhao,H., Batra,O., Hetrick,E.E., Howard,O.T., Kovar,L., Seabolt,M.H., Weigand,M.R., Burroughs,M., Lee,J., Wilkins,K., McCollum,A., Hutson,C., Davidson,W., Rao.A., Maloney,M. and li,Y.
EPI_ISL_15016131, EPI_ISL_15016132, EPI ISL 15016133, EPI ISL 15016134, - - EPI_ISL_150l61f5	Centers for Disease Contrai & Prevention (CDC). Division of High Consequence Pathogens and Pathology (DHCPP-PRB)	Centers for Disease Contrai & Prevention {CDC). Division of High Consequence Pathogens and Pathology (DHCPP-PRB)	Gigante.CM., Ventura,J., Zhao,H., Batra,D., Hetrick.E.E., Howard,D.T., Kovar,l., Seabolt,M.H., Weigand.M.R., Burroughs,M., Lee,J.. Wilkins.K., McCollum,A., Hutson,C. Davidson,W.. Rao,A.. Nash.J. and Li,Y.
EPI_JSL_15022589, EPI_ISL_15022590	Indian Council of Medical Research-National Institute of Virology	Indian Council of Medical Research-National Institute of Virology	Pragya Yadav, Rima Sahay, Anita Aich Shete, Sreelekshmy Mohandas, Priya Abraham
EPI_ISL_15023203	Centers for Disease Contrai & Prevention (CDC), Division of High Consequence Pathogens and Pathology (DHCPP-PRB)	Centers for Disease Contrai & Prevention (CDC). Division of High Consequence Pathogens and Pathology (DHCPP-PRB)	Gigante.CM., Pavlick,J., Zhao,H., Batra,D., Hetrick,E.E., Howard,D.T., Kovar,l., Seabolt,M.H., Weigand,M.R., Burroughs,M., Lee,J., Wilkins,K., McCollum,A., Hutson,C., Davidson,W., Rao.A., Parrott,T. and Li,Y.
EPI_ISL_15055820	Sicilian Regional Laboratory - AOUP "P. Giaccone" - University of Palerme	Sicilian Regional Laboratory - AOUP "P. Giaccone" - University of Palerme	Fabio Tramuto, Carmelo Massimo Maida, Giulia Randazzo, Valeria Guzzetta, Walter Mazzucco, Giorgio Graziano, Vincenzo Restivo, Claudio Costantino, Francesco Vitale
EPI_JSL_15076130, EPI_ISL_15076131	Environmental, Agricultural, and Occupational Health, University of Nebraska Medical Center	Environmental, Agricultural, and Occupational Health, University of Nebraska Medical Center	Chapman,R.C, Bernhard,K., McCutchen,E.l., Fauver,J.R., O'Dell,J.X., Mannell,M., Wiley,M.R. and Cross,S.T.
EPI ISL 15076132, EPI ISL 15076133, EPI ISL 15076134, EPI ISL 15076135, EPI ISL 15076136, EPI ISL 15076137, EPI ISL 15076138, EPI ISL 15076139, EPI ISL 15076140, EPI ISL 15076141, EPI ISL 15076142, EPI ISL 15076143, EPI ISL 15076144, EPI ISL 15076145, EPI ISL 15076146, EPI ISL 15076147, EPI ISL 15076148, EPI ISL 15076149, EPI ISL 15076150, EPI ISL 15076151, EPCISL)5076152, EPI=ISL)5076153, EPÜ5L)5076154, EPCISL)5076155, EPI=ISL)5076156, EPCISL)5076157. EPI=ISL)5076158, EPÜSL)5076159. EPCISL)50761 60, EPI=ISL)5076161, EPCISL)5076162. EPI=ISL)5076163, EPÜSL)5076164, EPCISL)5076165 - - - - - - - - - - - -			
see above	Centre for Biological Threats, Highly Pathogenic Viruses, Robert Koch Institute	Centre for Biological Threats, Highly Pathogenic Viruses, Robert Koch Institute	Brinkmann,A., Kohl,C, Pape,K., Uddin,S., Schrick,L., Michel,]., Schaade,L. and Nitsche,A
EPI_ISL_l5076180. EPI_ISL_l5076181, EPI_ISL_l5076182, EPI_ISL_15076183, EPI_ISL_l5076184, EPI_ISL_l5076185. EPI_ISL_l5076186, EPI_ISL_l5076187. EPI_ISL_15076188, EPI_ISL_l5076189, EPI_ISL_l5076191. EPI_ISL_l5076192, EPI_ISL_l5076193. EPI_ISL_15076194. EPI_ISL_l5076195, EPI_ISL_15076196. EPI_ISL_15076197			
see above	Department of Genetics, University of North Carolina at Chapel Hill	Department of Genetics, University of North Carolina at Chapel Hill	Deanhardt,B., Miller,M. and wang,J.R.
EPI_ISL_l5083938. EPI_ISL_15083939, EPI ISL 15083940, EPI ISL 15083941, EPI-ISL-15083942, EPI-ISL-15083943, EPJ-ISL-15083944, EPI-ISL-15083945, EPI ISL 15083946, EPC_ISL 15083947	Centre for Biological Threats. Highly Pathogenic Viruses, Robert Koch Institute	Centre for Biological Threats. Highly Pathogenic Viruses, Robert Koch Institute	Brinkmann,A., Kohl,C.. Pape.K.. Schrick,l., Michel.J., Schaade.l. and Nitsche.A
EPI_ISL_15098376, EPI_ISL_15098377, EPI_ISL_l5098378, EPI_ISL_15098379, EPI_ISL_15098381. EPI_ISL_15098382, EPI_ISL_15098383, EPI_ISL_l5098384, EPI_ISL_15098385, EPI_ISL_15098386, EPI_ISL_15098387, EPI_ISL_15098388, EPI_ISL_l5098389, EPI_ISL_15098390, EPI_ISL_15098391. EPI_ISL_15098392, EPI_ISL_15098393, EPI_ISL_l5098394, EPI_ISL_15098395			
see above	CT Department of Public Health	CT Department of Public Health	Claire Pearson. Tu N. Nguyen, Kutluhan Incekara
EPI_ISL_15104903	Institute for Virology, Philipps-University Marburg	Institute for Virology, Philipps-University Marburg	Eickmann, M., lier, C, Kowalski, K., Kraft, F., Becker, S.
EPI ISL 15116266, EPI ISL 15116267, EPI ISL 15116268, EPI ISL 15116269, EPI ISL 15116270, EPI ISL 15116271, EPI ISL 15116272, EPI ISL 15116273, EPI ISL 15116274, EPI ISL 15116275, EPI ISL 15116276, EPI ISL 15116277, EPI ISL 15116278, EPI ISL 15116279, EPI ISL 15116280, EPI ISL 15116281, EPI ISL 15116282, EPI ISL 15116283, EPI ISL 15116284, EPI ISL 15116285, EPCISL)5116286, EPCISL)5116287, EPÜSL)5116288. EPCISL)5116289, EPI=ISL)5116290, EPCISL)5116291. EPI=ISL)5116292, EPÜSL)5116293. EPCISL)511629 4, EPI=ISL)5116295, EPCISL)5116296. EPI=ISL)5116297, EPÜSL)5116298. EPI=ISL)5116299 - - - - - - - - - - - -			
see above	Centre for Biological Threats, Highly Pathogenic Viruses, Robert Koch Institute	Centre for Biological Threats, Highly Pathogenic Viruses, Robert Koch Institute	Brinkmann,A., Kohl,C, Pape,K., Schrick,L., Michel.J., Schaade,L. and Nitsche,A
EPJ_ISL_l5116300. EPI_ISL_15116301. EPI ISL 15116302, EPI ISL 15116303, EPI=ISL)5116304, EPI=ISL)5116305, EPI_ISL_15116306, EPI_ISL_15116307	Centers for Disease Contrai & Prevention (CDC). Division of High Consequence Pathogens and Pathology (DHCPP-PRB)	Centers for Disease Contrai & Prevention {CDC). Division of High Consequence Pathogens and Pathology (DHCPP-PRB)	Gigante.CM.. Plumb.M., Ruprecht,A., Zhao.H., Wicker,V., Wilkins.K., Matheny,A., Khan.T.. Davidson,W., Sheth,M., Burgin.A.. Burroughs,M., Padilla.J., Lee.J.S., Batra.D.. Hetrick.E.E.. Howard.D.T., Gartin,J., Tate.l.. Hubsmith,S.J., Mendoza,R.M., Stanek,O., Gillani,S., Lee,M., Mangla,A., Blythe,D., SierraPatev,S., Carpenter-Azevedo,K., Huard,R.C., Gallagher,G., Hall,)., Ash,S., Kovar,L., Seabolt.M.H., Weigand,M.R., Damon,I., Satheshkumar,P.S., McCollum,A.M., Hutson,C.L., Wang.X. and Li,Y.
EPI_ISL_l5120448. EPI_ISL_15120449, EPJ_ISL_l5120450. EPI_JSL_15120451, EPI_ISL_15120452, EPI_ISL_l5120453. EPI_ISL_15120454, EPI_ISL_l5120455. EPI_ISL_15120456. EPI_ISL_15120457, EPI_ISL_l5120458. EPI_ISL_15120459, EPI_ISL_l5120461. EPI_ISL_l5120462, EPI_ISL_15120463, EPI_ISL_15120464. EPI_ISL_15120465, EPI_ISL_l5120466. EPI_ISL_l5120467, EPI_ISL_15120468, EPI ISL 15120469, EPI ISL 15120470, EPI ISL 15120473, EPI ISL 15120474, EPI ISL 15120475, EPI ISL 15120477, EPI ISL 15120478, EPI ISL 15120479, EPI ISL 15120480, EPI ISL 15120481. EPI ISL 15120482, EPI ISL 15120483, EPI ISL 15120484, EPI ISL 15120485, EPI ISL 15120486, EPI ISL 15120487, EPI ISL 15120489, EPI ISL 15120490, EPI ISL 15120492, EPI ISL 15120494, EPI=ISL)5120495, EPI=ISL)5120496, EPÜSL)5120497, EPI=ISL)5120498, EPI=ISL)5120499, EPI=ISL)5120500, EPI=ISL)5120501, EPÜSL)51 20502, EPI=ISL)5120503, EPI=ISL)5120504 -			
see above	Los Angeles County Public Health Laboratories	Los Angeles County Public Health Laboratories	P. Hemarajata et al.
EPI_ISL_15158314	Arbovirology and Entomology, Bernhard Nocht Institute for Tropical Medicine	Arbovirology and Entomology, Bernhard Nocht Institute for Tropical Medicine	Emmerich,P , Bialonski,A., Tomazatos,A. and Cadar,D.
EPI ISL 15158315, EPI ISL 15158316, EPI ISL 15158317, EPI ISL 15158318, EPI ISL 15158319, EPI ISL 15158320, EPI ISL 15158321, EPI ISL 15158322, EPI ISL 15158323, EPI ISL 15158324, EPI ISL 15158325, EPI ISL 15158326, EPI ISL 15158327, EPI ISL 15158328, EPI ISL 15158329, EPI ISL 15158330, EPI ISL 15158331, EPI ISL 15158332, EPI ISL 15158333, EPI ISL 15158335, EPI=ISL)5158336. EPI=ISL)5158337, EPÜSL)5158338. EPI=ISL=15158339, EPI=ISL)5158340, EPI=ISL)5158341. EPI=ISL)5158342, EPÜSL)5158343. EPI=ISL)5158344, EPI=ISL)5158345, EPI=ISL)5158346, EPI=ISL)5158347, EPÜSL)5158348. EPI=ISL)5158350, EPI=ISL)5158351, EPI=ISL)5158352. EPI=ISL)5158353. EPÜSL)5158354, EPI=ISL)5158355, EPI=ISL)5158356, EPI ISL 15158357, EPI ISL 15158358, EPI ISL 15158359, EPI ISL 15158360, EPI ISL 15158361. EPI ISL 15158362, EPI ISL 15158363, EPI ISL 15158364, EPI ISL 15158365, EPI ISL 15158366, EPI ISL 15158367, EPI ISL 15158368, EPI ISL 15158369, EPI ISL 15158370, EPI ISL 15158371, EPI ISL 15158374, EPI ISL 15158375, EPI ISL 15158376, EPI ISL 15158378, EPI ISL 15158379, EPI=ISL)5158380, EPI=ISL)5158381, EPÜSL)5158382, EPI=ISL)5158383, EPI=ISL)5158384, EPI=ISL)5158385, EPI=ISL)5158387, EPÜSL)5158388, EPI=ISL)5158389, EPI=ISL)5158390, EPI=ISL)5158391, EPI=ISL)5158392, EPÜSL)5158393, EPI=ISL)5158394, EPI=ISL)5158395, EPI=ISL)5158396. EPI=ISL)5158397, EPÜSL)5158398 - - - -			
see above	Molecular Biology, Microbiology, and Biochemistry, Southern Illinois University	Molecular Biology, Microbiology, and Biochemistry, Southern Illinois University	Gagnon,K.T.
EPI ISL 15158399, EPI ISL 15158400, EPJ ISL 15158401, EPI JSL 15158402, EPI ISL 15158403, EPI ISL 15158404, EPI ISL 15158405, EPI ISL 15158406, EPI ISL 15158407, EPI ISL 15158408, EPI ISL 15158409, EPI ISL 15158410, EPI ISL 15158411, EPI ISL 15158412, EPI ISL 15158413, EPI ISL 15158414. EPI ISL 15158415, EPI ISL 15158416, EPI ISL 15158417, EPI ISL 15158418, EP1=1sL)5158419, EPC1sL)5158420, EPÜSL)5158421. EPC1sL)5158422, EPC1sL)5158423, EP1=1sL)5158424. EPC1sL)5158425, EPÜSL)5158426, EPC1sL)5158427, EPC1sL)5158428, EP1=1sL)5158429, EPC1sL)5158430, EPÜSL)5158431, EPC1sL)5158432, EPC1sL)5158433, EP1=1sL)5158434, EPC1sL)5158435, EPÜSL)5158436, EPC1sL)5158437, EPC1sL)5158438, EPI ISL 15158439, EPI ISL 15158440, EPI ISL 15158441, EPI ISL 15158442, EPI ISL 15158443, EPI ISL 15158444, EPI ISL 15158445, EPI ISL 15158446, EPI ISL 15158447, EPI ISL 15158448, EPI ISL 15158449, EPI ISL 15158450, EPI ISL 15158451, EPI ISL 15158452, EPI ISL 15158453, EPI ISL 15158454, EPI ISL 15158455, EPI ISL 15158456, EPI ISL 15158457, EPI ISL 15158458, EPI=ISL)5158459, EPI=ISL)5158460, EPÜSL)5158461, EPI=ISL)5158462, EPI=ISL)5158463, EPI=ISL)5158464, EPI=ISL)5158465, EPÜSL)51 58466, EPCISL)5158467, EPI=ISL)5158468 -			
see above	Research and Evaluation, UKHSA	Research and Evaluation, UKHSA	Groves,N., Osman,K.L., Lewandowski,K.5., Carter,D.P., Pullan,S.T., Myers,R., Vipond,R. and Chand,M.
EPI_ISL_15165602, EPI_ISL_15165603, EPI_ISL_15165604, EPI_ISL_15165605, EPI_ISL_15165606, EPI_ISL_15165607, EPI_ISL_l5165608, EPI_ISL_15165610, EPI_ISL_l5165611, EPI_ISL_15165612, EPI_ISL_15165614, EPI_ISL_l5165615, EPI_ISL_15165616, EPI_ISL_l5165617, EPI_ISL_l5165618			
see above	Centra de Desenvolvimento Cientffico e Tecnol6gico (CDCT), Centra Estadual de Vigilância em Sal.lde	Centra de Desenvolvimento Cientffico e Tecnol6gico (CDCT). Centra Estadual de Vigilância em Saùde	Richard Steiner Salvato, Fernanda Marques Godinho, Regina Bones Barcellos, Patricia Sesterheim, Amanda Pellenz Ruivo, Viviane Horn de Melo, JUiio Augusto Schroder

	(CEVSJ da Secretaria Estadual da SaUde (SES-RS)	(CEVS) da Secretaria Estadual da SaUde (SES-RS)	
EPI ISL 15199623. EPI ISL 15199624, EPI I5l 15199627, EPI I5L 15199628, EPI ISL 15199629, EPI ISL 15199630. EPI I5L 15199632, EPI ISL 15199635, EPI ISL 15199636, EPI ISL 15199638, EPI ISL 15199639. EPI ISL 15199640, EPI ISL 15199643, EPI ISL 15199644, EPI ISL 15199645, EPI ISL 15199646. EPI ISL 15199647, EPI ISL 15199648, EPI ISL 15199649, EPI ISL 15199652, EPI-ISL-15199653, EPI-ISL-15199655, EPI-ISL-15199656, EPI-ISL-15199657, EPI-ISL-15199658, EPI-ISL-15199659, EPI-ISL-15199660, EPI-ISL-15199661, EPI-ISL-15199662, EPI-ISL-15199664, EPI-ISL-15199665, EPI-ISL-15199666, EPI-ISL-15199667, EPI-ISL-15199668, EPI-ISL-15199669, EPI-ISL-15199670, EPI-ISL-15199671, EPI-ISL-15199672, EPI-ISL-15199673, EPI-ISL-15199675, EPI-ISL-15199676, EPI-ISL-15199677, EPI-ISL-15199679, EPI-ISL-15199680, EPI-ISL-15199681, EPI-ISL-15199682, EPI-ISL-15199685, EPI-ISL-15199686, EPI-ISL-15199687, EPI-ISL-15199688, EPI-ISL-15199690, EPI-ISL-15199691, EPI-ISL-15199692, EPI-ISL-15199693, EPI-ISL-15199695, EPI-ISL-15199696, EPI-ISL-15199697, EPI-ISL-15199698, EPI-ISL-15199699, EPI-ISL-15199700, EPI-ISL-15199701, EPI-ISL-15199702, EPI-ISL-15199703, EPI-ISL-15199705, EPI-ISL-15199706, EPI-ISL-15199707, EPI-ISL-15199710, EPI-ISL-15199711, EPI-ISL-15199712, EPI-ISL-15199713, EPI-ISL-15199717, EPI-ISL-15199720, EPI-ISL-15199721, EPI-ISL-15199723, EPI-ISL-15199724, EPI-ISL-15199725, EPI-ISL-15199726, EPI-ISL-15199727, EPI-ISL-15199728, EPI-ISL-15199729, EPI-ISL-15199730, EPI-ISL-15199731, EPI-ISL-15199732, EPI-ISL-15199733, EPI-ISL-15199735, EPI-ISL-15199736, EPI-ISL-15199737, EPI-ISL-15199738, EPI-ISL-15199739, EPI-ISL-15199740, EPI-ISL-15199741, EPI-ISL-15199742, EPI-ISL-15199743, EPI-ISL-15199744, EPI-ISL-15199745, EPI-ISL-15199747, EPI-ISL-15199748, EPI-ISL-15199749, EPI-ISL-15199750, EPI-ISL-15199751, EPCISL)5199752, EPI=ISL)5199753, EPÜSL)5199754, EPCISL)5199755, EPI=ISL)5199757, EPCISL)5199758. EPI=ISL)5199759, EPÜSL)5199761, EPCISL)5199762, EPI=ISL)5199763, EPCISL)5199764. EPI=ISL)5199765, EPÜSL)5199766, EPCISL)5199767, EPI=ISL)5199768, EPCISL)5199769. EPI=ISL)5199773, EPÜSL)5199775, EPCISL)5199776, EPI=ISL)5199777, EPI ISL 15199778, EPI ISL 15199780, EPI ISL 15199781, EPI ISL 15199785, EPI ISL 15199787, EPI ISL 15199788, EPI ISL 15199789, EPI ISL 15199790, EPI ISL 15199791, EPI ISL 15199792, EPI ISL 15199793, EPI ISL 15199794, EPI ISL 15199795, EPI ISL 15199796, EPI ISL 15199797, EPI ISL 15199798, EPI ISL 15199799, EPI ISL 15199830, EPI ISL 15199856, EPI ISL 15199859, EPI=ISL)5199860, EPI=ISL)5199861, EPÜSL)5199862, EPI=ISL)5199863, EPI=ISL)5199864, EPI=ISL)5199866, EPI=ISL)5199868, EPÜSL)5199869, EPI=ISL)5199870, EPI=ISL)5199871, EPI=ISL)5200120 - - - - - - - - - - - - - - - - - -			
see above	Department of Infectious Diseases, National Institute of Health Doutor Ricardo Jorge, Portugal (INSA)	Department of Infectious Diseases, National Institute of Health Doutor Ricardo Jorge, Portugal (INSA)	Isidro,)., 8orges,V., Pinto,M., Sobral,D., Santos.)., Nunes,A., Mixao,v., Ferreira,R., Santos,□.,Duarte,S., Vieira,I., Borrego,M.J., Nuncio,S., Lopes de Carvalho,I., Pelerito,A., Cordeiro,R. and Gomes,J.P.
EPI_ISL_l5226669, EPI_ISL_l5226670, EPI_ISL_l5226671, EPI_ISL_l5226672, EPI_ISL_l5226673, EPI_ISL_l5226674, EPI_ISL_l5226675, EPI_ISL_l5226676, EPI_ISL_l5226677, EPI_ISL_l5226678, EPI_ISL_l5226679, EPI_ISL_l5226681, EPI_ISL_l5226682, EPI_ISL_l5226683, EPI_ISL_l5226684, EPI_ISL_l5226685, EPI_ISL_l5226686, EPI_ISL_l5226687, EPI_ISL_l5226688, EPI_ISL_l5226689, EPI_ISL_l5226690, EPI_ISL_l5226691, EPI_ISL_l5226692, EPI_ISL_l5226693, EPI_ISL_l5226694, EPI_ISL_l5226695			
see above	Los Angeles County Public Health Laboratories	Los Angeles County Public Health Laboratories	P. Hemarajata et al.
EPI_ISL_l5247221, EPI_ISL_l5247222, EPI_ISL_l5247223, EPI_ISL_l5247224, EPI-ISL-15247225, EPI-ISL-15247226, EPCis(.15241221, EPC1sL 1524122s	Medical University of Vienna Center for Virology	Medical University of Vienna Center for Virology	Jeremy V. Camp, Monika Redlberger-Fritz, Stephan W. Aberle
EPI_ISL_l5257658, EPI_ISL_l5257659, EPI_ISL_l5257660, EPI_ISL_l5257661, EPI_ISL_l5257662, EPI_ISL_l5257663, EPI_ISL_l5257664, EPI_ISL_l5257665, EPI_ISL_l5257666, EPI_ISL_l5257667, EPI_ISL_l5257668			
see above	Centre for 8iological Threats, Highly Pathogenic Viruses. Robert Koch Institute	Centre for Biological Threats, Highly Pathogenic Viruses, Robert Koch Institute	Brinkmann,A., Kohl,C., Pape,K., Schrick,L., Michel,)., Schaade,L. and Nitsche,A
EPI_ISL_l5257669	Public Health Agency of Canada, National Microbiology Laboratory	Public Health Agency of Canada, National Microbiology Laboratory	Duggan,A., Hole,D., Yadav,C., Knox,N., Haidl,E., Chapel,M., Tyler.A.□.,Domselaar,G.V., Graham,M., Audet,J., Fernando,L., Hagan,M., Safronetz,D., Leung,A., Peters,G., Go,A., Kaplen,B., Antonation,K., Laminman,V., Jolly,G., Croxen,M., DeoA, Dieu,P., Dong.X., Gill.K., Granger.D., Ferrato.C., Ikkurti,V., Kanji.J., Koleva,P., Li.V., lloyd,C., Lynch,T., Ma,R., Pabbaraju,K.. Rotich,S., Sergeant,H.. Skitsko.T.. Tipples,G., Thayer.J.. Shideler.S. and Wang.A.
EPI_ISL_l5257670, EPI_ISL_l5257671, EPI_ISL_l5257672, EPI_ISL_l5257673, EPI_ISL_l5257674, EPI_ISL_l5257675, EPI_ISL_l5257676, EPI_ISL_l5257678, EPI_ISL_l5257679, EPI_ISL_l5257680, EPI_ISL_l5257681, EPI_ISL_l5257682, EPI_ISL_l5257683, EPI_ISL_l5257684, EPI_ISL_l5257685, EPI_ISL_l5257686, EPI_ISL_l5257687			
see above	Viral and Rickettsial Disease Laboratory, California Department of Public Health	Viral and Rickettsial Disease Laboratory, California Department of Public Health	Probert,W., Espinosa,A., Kath,C., Haw,M., O'Neil,R., Bell,). and Hacker,J.
EPI_ISL_l5260057	High Containment Facility, Virology Division, Defence Research & Develeopment Establishment (DRDE)	Virology Division, Defence Research & Develeopment Establishment (DRDE)	Dr Sushil Kumar Sharma, Dr Paban Kumar Dash, Ram Govind Yadav, Ambuj Shrivastava, Rohit Menon, Dr Jyoti S Kurnar, Dr Shashi Sharma, Dr Suman Dhankher, Divya Kurnari, Dr Manmohan Parida
EPI_ISL_l5263355	Sicilian Regional Laboratory - AOUP "P. Giaccone" - University of Palermo	Sicilian Regional Laboratory - AOUP "P. Giaccone" - University of Palermo	Fabio Tramuto. Carmelo Massimo Maida, Giulia Randazzo, Valeria Guzzetta, Walter Mazzucco, Giorgio Graziano, Vincenzo Restivo, Claudio Costantino, Francesco Vitale
EPI ISL 15264003, EPI ISL 15266514, EPI ISL l5266518, EPCISL l5266615	Erasmus Medical Center Department of Virology	Erasmus Medical Center Department of Virology	Leonard Schuele, Bas Oude Munnink, Marjan Soter, Babette Weller, Babs Verstrepen, Richard Molenkamp, Janette Rahamat-Langendoen, Reina Sikkema, Marion Koopmans
EPI_ISL_l5266843	Erasmus Medical Center Department of Virology	Erasmus Medical Center Department of Virology	Leonard Schuele, Bas Oude Munnink, Marjan Soter, David Nieuwenhuijse, Babette Weller, Babs Verstrepen, Richard Molenkamp, Janette Rahamat-Langendoen, Reina Sikkema, Marion Koopmans
EPI ISL 15266988, EPI ISL 15267015, EPI-ISL-15267029, EPI-ISL-15267031, EPÜSL)5267800, EPCISL=l5268281, EPI_ISL_l5269049	Erasmus Medical Center Department of Virology	Erasmus Medical Center Department of Virology	Leonard Schuele, Bas Oude Munnink, Marjan Boter, Babette Weller, Babs Verstrepen, Richard Molenkamp, Janette Rahamat-Langendoen, Reina Sikkema, Marion Koopmans
EPI ISL 15269237, EPI ISL 15269238, EPI-ISL-15269375, EPI-ISL-15269384, EPÜSL)5269386, EPI=ISL)5269387, EPI ISL 15269388, EPI ISL 15269544, EPI ISL l5269595, EPCISL l5269598	Erasmus Medical Center Department of Viralogy	Erasmus Medical Center Department of Virology	Leonard Schuele, Bas Oude Munnink, Marjan Soter, David Nieuwenhuijse, Babette Weller. Babs Verstrepen, Richard Molenkamp, Janette Rahamat-Langendoen, Reina Sikkema, Marion Koopmans
EPI ISL 15269698, EPI ISL 15269699, EPÜSL)5269702, EPI=ISL)5269704, EPI_ISL_l5269708	Erasmus Medical (enter Department of Virology	Erasmus Medical (enter Department of Virology	Leonard Schuele, Bas Oude Munnink, Marjan Boter, Babette Weller, Babs Verstrepen, Richard Molenkamp, Janette Rahamat-Langendoen, Reina Sikkema, Marion Koopmans
EPI_ISL_l5283583. EPI_ISL_l5283585, EPI_ISL_l5283587, EPI_ISL_l5283589, EPI_ISL_l5283590, EPI_ISL_l5283592. EPI_ISL_l5283594, EPI_ISL_l5283596. EPI_ISL_l5283598. EPI_ISL_l5283600, EPI_ISL_l5283601. EPI_ISL_l5283603, EPI_ISL_l5283605. EPI_ISL_l5283607. EPI_ISL_l5283608, EPI_ISL_l5283610. EPI_ISL_l5283611, EPI_ISL_l5283612. EPI_ISL_l5283614, EPI_ISL_l5283616, EPI_ISL_l5283618, EPI_ISL_l5283620, EPI_ISL_l5283622			
see above	Centre for 8iological Threats, Highly Pathogenic Viruses. Robert Koch Institute	Centre for Biological Threats, Highly Pathogenic Viruses, Robert Koch Institute	Brinkmann,A., Kohl,C., Pape,K., Schrick,l., Michel,)., Schaade,L. and Nitsche,A
EPI_ISL_15292947	Center of Scientific Excellence for Influenza Viruses, National Research Centre {NRC)	Center of Scientific Excellence for Influenza Viruses, National Research Centre (NRC)	Roshdy,W.H., El-Shesheny,R., Moatasim,Y.M., Kamel,M., Shawky,S., Gomaa,M., Naguib,A., El Guindy,N., Fahim,M., Khalifa,M., Galal,R., Hassany,M., Mohsen,A., Ali.MA and Kandeel,A
EPI ISL 15292951, EPI ISL 15292953, EPÜSL)5292954, EPCISL=l5292960, EPI_ISL_l5292962, EPI_ISL_l5292969	Minnesota Department of Health	Minnesota Department of Health	Plurnb,M.R.
EPI_ISL_l5292973, EPI_ISL_l5292974, EPI_ISL_l5292975	USAFSAM, us Air Force	USAFSAM, us Air force	fries,A.
EPI_ISL_l5292976. EPI_ISL_l5292977, EPI_ISL_l5292978. EPI_ISL_l5292979, EPI_ISL_l5292980, EPI_ISL_l5292981. EPI_ISL_l5292982, EPI_ISL_l5292983. EPI_ISL_l5292984, EPI_ISL_l5292985, EPI_ISL_l5292986. EPI_ISL_l5292987, EPI_ISL_l5292988. EPI_ISL_l5292989. EPI_ISL_l5292990, EPI_ISL_l5292991. EPI_ISL_l5292992, EPI_ISL_l5292993. EPI_ISL_l5292994, EPI_ISL_l5292995, EPI_ISL_l5292996, EPI_ISL_l5292997, EPI_ISL_l5292998, EPI_ISL_l5292999, EPI_ISL_l5293000, EPI_ISL_l5293001, EPI_ISL_l5293002, EPI_ISL_l5293003, EPI_ISL_l5293004, EPI_ISL_l5293005, EPI_ISL_l5293006, EPI_ISL_l5293007, EPI_ISL_l5293008, EPI_ISL_l5293009			
see above	Institute of Health Carlos Ill, Bioinformatics Unit	Institute of Health Carlos Ill, Bioinformatics Unit	Cuesta,!.
EPI_ISL_l5293815	National Institute for Viral Disease Contrai and Prevention (IVDC), Chinese Center for Disease Contrai and Prevention , Beijing, China	National Institute for Viral Disease Contrai and Prevention (IVDC). Chinese Center for Disease Contrai and Prevention , Beijing, China	Wenjie Tan. Changcheng Wu, Ruhan A. Wenling Wang. Roujian Lu. Li Zhao, Baoying Huang. Fei Ye. Wenbo Xu
EPI_ISL_l5317143	Centers for Disease Contrai & Prevention (CDC), Division of High Consequence Pathogens and Pathology (DHCPP-PRB)	Centers for Disease Contrai & Prevention {CDC). Division of High Consequence Pathogens and Pathology (DHCPP-PRB)	Gigante.CM., Vang,K., Zhao,H., Batra,D., Hetrick,E.E., Howard,D.T., Kovar,L., Seabolt,M.H., Weigand,M.R., Burraughs,M., Lee,J., Wilkins,K., McCollum,A., Hutson,C., Davidson,W., Rao.A., Seely,K. and Li,Y.
EPI_ISL_l5317144	Centers for Disease Contrai & Prevention (CDC). Division of High Consequence Pathogens and Pathology (DHCPP-PRB)	Centers for Disease Contrai & Prevention {CDC). Division of High Consequence Pathogens and Pathology (DHCPP-PRB)	Gigante.CM., Segaloff.H.. Zhao,H., Batra.D., Hetrick.E.E., Howard,D.T., Kovar,l., Seabolt,M.H., Weigand.M.R.. Burraughs,M.. Lee,J.. Wilkins.K.. McCollum,A., Hutson,C. Davidson.W.. Rao.A.. florek.K. and li.Y.
EPI_ISL_l5317145	Centers for Disease Contrai & Prevention (CDC), Division of High Consequence Pathogens and Pathology (DHCPP-PRB)	Centers for Disease Contrai & Prevention {CDC). Division of High Consequence Pathogens and Pathology (DHCPP-PRB)	Gigante.CM., Pavlick,J., Zhao,H., Batra,D., Hetrick,E.E., Howard,D.T., Kovar,L., Seabolt,M.H., Weigand,M.R., Burroughs,M., Lee,J., Wilkins,K., McCollum,A., Hutson,C, Davidson,w., Rao.A., Parrott,T. and Li,Y.
EPI_I5L_l5317146	Centers for Oisease Contrai & Prevention (CDC), Division of High Consequence Pathogens and Pathology (DHCPP-PRB)	Centers for Disease Contrai & Prevention {CDC). Division of High Consequence Pathogens and Pathology (DHCPP-PRB)	Gigante.CM., Plumb,M., Ruprecht,A., Zhao,H., Wicker,V., Wilkins,K., Matheny,A., Khan,T., Davidson,W., Sheth,M., Burgin,A., Burroughs,M., Padilla,J., Lee,J.S., Batra,D., Hetrick,E.E., Howard,D.T., Garfin,J., Tate,L., Hubsmith,S.J., Mendoza,R.M., Stanek,D., Gillani,S., Lee,M., Mangla,A., Blythe,D., SierraPatev,S., Carpenter-Azevedo,K., Huard,R.C, Gallagher,G., Hall,J., Ash,S., Kovar,L., Seabolt,M.H., Weigand,M.R., Damon,I., Satheshkumar,P.S., McCollum,A.M., Hutson,CL., Wang.X. and Li.Y.
EPI_ISL_l5317147	Centers for Disease Contrai & Prevention (CDC), Division of High Consequence Pathogens and Pathology (DHCPP-PRB)	Centers for Disease Contrai & Prevention {CDC). Division of High Consequence Pathogens and Pathology (DHCPP-PRB)	Gigante,C.M., Epie,N., Zhao,H., Batra,O., Hetrick,E.E., Howard,O.T., Kovar,L., Seabolt,M.H., Weigand,M.R., Burroughs,M., Lee,J., Wilkins.K., McCollum,A., Hutson,C, Oavidson,W., Rao.A., Perez,T. and Li,Y.
EPI_ISL_l5317148	Centers for Oisease Contrai & Prevention (CDC), Division of High Consequence Pathogens and Pathology (DHCPP-PRB)	Centers for Disease Contrai & Prevention (CDC). Division of High Consequence Pathogens and Pathology (DHCPP-PRB)	Gigante.CM., Acheampong,E., Zhao,H., Batra,D., Hetrick,E.E., Howard,D.T., Kovar,L., Seabolt,M.H., Weigand,M.R., Burroughs,M., Lee,J., Wilkins,K., McCollum,A., Hutson,C, Davidson,W., Rao.A., McDermott,D. and li.Y.
EPI_ISL_l5317149	Centers for Disease Contrai & Prevention (CDC), Division of High Consequence Pathogens and Pathology (DHCPP-PRB)	Centers for Disease Contrai & Prevention {CDC). Division of High Consequence Pathogens and Pathology (DHCPP-PRB)	Gigante.CM.. Xia.□.. Zhao.H., Batra,O., Hetrick.E.E., Howard.0.T.. Kovar.L.. Seabolt.M.H.. Weigand.M.R.. Burroughs,M., Lee,J., Wilkins,K., McCollum.A.. Hutson,C, Davidson,W., Rao.A., Plipat.N. and Li,Y.
EPI_ISL_l5317150	Centers for Disease Contrai & Prevention (CDC), Division of High Consequence Pathogens and Pathology (DHCPP-PRB)	Centers for Disease Contrai & Prevention (CDC). Division of High Consequence Pathogens and Pathology (DHCPP-PRB)	Gigante.CM., Ruiz.V., Zhao,H., Batra,D., Hetrick,E.E., Howard,D.T., Kovar,L., Seabolt,M.H., Weigand,M.R., Burroughs,M., Lee,J., Wilkins,K., McCollum,A., Hutson,C., Davidson,W., Rao.A., Wang,). and Li.Y.
EPI_ISL_l5317151	Centers for Disease Contrai & Prevention (CDC). Division of High Consequence Pathogens and Pathology (DHCPP-PRB)	Centers for Disease Contrai & Prevention {CDC). Division of High Consequence Pathogens and Pathology (DHCPP-PRB)	Gigante.CM.. Francis,□.,Zhao,H.. Batra,D.. Hetrick,E.E., Howard.D.T., Kovar,L., Seabolt.M.H.. Weigand,M.R., Burroughs,M., Lee,J.. Wilkins.K.. McCollum,A.. Hutson,C, Davidson,W.. Rao.A.. Escobar,). and Li,Y.
EPI_ISL_l5317152	Centers for Disease Contrai & Prevention (CDC), Division of High Consequence Pathogens and Pathology (DHCPP-PRB)	Centers for Disease Contrai & Prevention (CDC). Division of High Consequence Pathogens and Pathology (DHCPP-PRB)	Gigante,CM., Johnson,$., Zhao,H., Batra,D., Hetrick,E.E., Howard,D.T., Kovar,L., Seabolt,M.H., Weigand,M.R., Burroughs,M., Lee,J., Wilkins,K., McCollum,A., Hutson,C, Davidson,w., Rao.A., Riner,D. and Li,Y.
EPI_ISL_l5317153	Centers for Oisease Contrai & Prevention (CDC), Division of High Consequence Pathogens and	Centers for Disease Contrai & Prevention (CDC). Division of High Consequence Pathogens and	Gigante.CM., Culbertson,M., Zhao,H., Batra,D., Hetrick,E.E., Howard,O.T., Kovar,L., Seabolt,M.H., Weigand,M.R., Burroughs,M., Lee,J., Wilkins,K., McCollum,A., Hutson,C, Davidson,W., Rao.A., Pope,B. and li.Y

	Pathology (DHCPP-PRB)	Pathology (DHCPP-PRB)	
EPI_ISL_15317154	Centers for Disease Control & Prevention (CDC), Division of High Consequence Pathogens and Pathology (DHCPP-PRB)	Centers for Disease Contrai & Prevention {CDC). Division of High Consequence Pathogens and Pathology (DHCPP-PRB)	Gigante,C.M.. Ventura,J.. Zhao,H., Batra,D.. Hetrick,E.E.. Howard,D.T., Kovar,L., Seabolt,M.H., Weigand,M.R., Burroughs,M.. Lee,J.. Wilkins,K.. McCollum,A.. Hutson,C.. Davidson,W.. Rao.A.. Nash,J. and Li,Y.
EPI_ISL_l5317155	Centers for Disease Contrai & Prevention (CDC), Division of High Consequence Pathogens and Pathology (DHCPP-PRB)	Centers for Disease Contrai & Prevention {CDC). Division of High Consequence Pathogens and Pathology (DHCPP-PRB)	Gigante,C.M., francis,D., Zhao,H., Batra,D., Hetrick,E.E., Howard,D.T., Kovar,L., Seabolt,M.H., Weigand,M.R., Burroughs,M., Lee,J., Wilkins,K., McCollum,A., Hutson,C., Davidson,W., Rao.A., Escobar,J. and Li,Y.
EPI_ISL_15317156	Centers for Disease Control & Prevention (CDC), Division of High Consequence Pathogens and Pathology (DHCPP-PRB)	Centers for Disease Control & Prevention {CDC). Division of High Consequence Pathogens and Pathology (DHCPP-PRB)	Gigante,C.M.. Ruiz,V., Zhao,H.. Batra,D.. Hetrick,E.E.. Howard,D.T.. Kovar,L.. Seabolt.M.H.. Weigand,M.R., Burroughs,M.. Lee,J., Wilkins,K.. McCollum,A.. Hutson,C., Davidson,W.. Rao.A.. Wang,J. and Li,Y.
EPI_ISL_15317157	Centers for Disease Contrai & Prevention (CDC), Division of High Consequence Pathogens and Pathology (DHCPP-PRB)	Centers for Disease Contrai & Prevention (CDC). Division of High Consequence Pathogens and Pathology (DHCPP-PRB)	Gigante,C.M., Pavlick,J., Zhao.H., Batra,D.. Hetrick,E.E., Howard.D.T.. Kovar.L., Seabolt,M.H., Weigand,M.R., Burroughs,M., Lee.J., Wilkins,K., McCollum.A., Hutson,C., Davidson,W., Rao,A., Parrott,T. and Li,Y.
EPJ ISL 15317158, EPI JSL 15317159, - - EPI_ISL_153l716-0	Centers for Disease Contrai & Prevention (CDC), Division of High Consequence Pathogens and Pathology (DHCPP-PRB)	Centers for Disease Control & Prevention (CDCl. Division of High Consequence Pathogens and Pathology (DHCPP-PRB)	Gigante,C.M., Ventura,J., Zhao,H., Batra,D., Hetrick,E.E., Howard,D.T., Kovar,L., Seabolt,M.H., Weigand,M.R., Burroughs,M., Lee,J., Wilkins,K., McCollum,A., Hutson,C., Davidson,W., Rao,A., Nash,). and Li,Y.
EPI_ISL_15321163, EPI_ISL_15321165, EPI_ISL_15321166, EPI_ISL_15321167, EPI_ISL_15321168, EPI_ISL_15321169, EPI_ISL_15321170, EPI_ISL_15321171, EPI_ISL_15321172, EPI_ISL_15321174, EPI_ISL_15321175			
see above	Rhode Island State Health Laboratory	Rhode Island State Health Laboratory	Kristin Carpenter-Azevedo, Sean Sierra-Patev, Richard C. Huard
EPI ISL 15325408, EPI I5L 15325409, EPI ISL 15325410, EPI JSL 15325411, EPI I5L 15325413, EPI ISL 15325414, EPI I5L 15325415, EPI ISL 15325416, EPI ISL 15325417, EPI ISL 15325418, EPI ISL 1S325419, EPI ISL 15325421, EPI ISL 15325422, EPI ISL 15325423, EPI ISL 15325424, EPI ISL 1S325425, EPI ISL 15325426, EPI ISL 15325428, EPI ISL 15325429, EPI ISL 15325430, EPCISL)5325431. EPI=ISL)5325432			
see above	Los Angeles County Public Health Laboratories	Los Angeles County Public Health Laboratories	P. Hemarajata et al.
EPI_ISL_15325433	Ouest Diagnostics Nichols Institute	Los Angeles County Public Health Laboratories	P. Hemarajata et al.
EPI_ISL_15332321	Centers for Disease Control & Prevention (CDC), Division of High Consequence Pathogens and Pathology (DHCPP-PRB)	Centers for Disease Control & Prevention (CDC). Division of High Consequence Pathogens and Pathology (DHCPP-PRB)	Gigante,C.M., Carlson,C., Zhao,H., Batra,D., Hetrick,E.E., Howard,D.T., Kovar,L. Seabolt,M.H., Knipe,K., Burroughs,$., Lee,)., Wilkins,K., McCollum,A., Hutson,C., Davidson,W., Rao.A., Southern,T. and Li,Y.
EPI_ISL_15332322	Centers for Disease Contrai & Prevention (CDC), Division of High Consequence Pathogens and Pathology (DHCPP-PRB)	Centers for Disease Control & Prevention {CDC). Division of High Consequence Pathogens and Pathology (DHCPP-PRB)	Gigante,C.M.. Murray,J., Zhao,H., Batra.D., Hetrick,E.E., Howard,D.T., Kovar,L., Seabolt,M.H., Knipe,K., Burroughs,S., Lee,J., Wilkins,K., McCollum,A., Hutson.C., Davidson.W., Rao.A., Atkinson,A. and Li,Y.
EPI_ISL_15332323	Centers for Disease Contrai & Prevention (CDC), Division of High Consequence Pathogens and Pathology (DHCPP-PRB)	Centers for Disease Control & Prevention {CDC). Division of High Consequence Pathogens and Pathology (DHCPP-PRB)	Gigante,C.M., Kubin,G., Zhao,H., Batra,D., Hetrick,E.E., Howard,D.T., Kovar,L., Seabolt,M.H., Knipe,K., Burroughs,S., Lee,J., Wilkins,K., McCollum,A., Hutson,C., Davidson,w., Rao.A., White,S.L. and Li,Y.
EPI_ISL_15332324	Centers for Disease Control & Prevention (CDC), Division of High Consequence Pathogens and Pathology (DHCPP-PRB)	Centers for Disease Control & Prevention {CDC). Division of High Consequence Pathogens and Pathology (DHCPP-PRB)	Gigante,C.M.. Xia,☐.,Zhao,H.. Batra,D.. Hetrick,E.E.. Howard,D.T.. Kovar,L.. Seabolt,M.H.. Knipe,K.. Burroughs,S.. Lee.)., Wilkins,K., McCollum,A.. Hutson,C., Davidson,W., Rao.A., Plipat.N. and Li,Y
EPI_ISL_l5332325	Centers for Disease Control & Prevention (CDC), Division of High Consequence Pathogens and Pathology (DHCPP-PRB)	Centers for Disease Contrai & Prevention {CDC). Division of High Consequence Pathogens and Pathology (DHCPP-PRB)	Gigante,C.M., Pettit,D., Zhao,H., Batra,D., Hetrick,E.E., Howard,D.T., Kovar,L., Seabolt,M.H., Knipe,K., Burroughs,S., Lee,J., Wilkins,K., McCollum,A., Hutson,C., Davidson,W., Rao,A., Deutsch-Feldman,M. and Li,Y.
EPI_ISL_15332326	Centers for Disease Control & Prevention (CDC), Division of High Consequence Pathogens and Pathology (DHCPP-PRB)	Centers for Disease Control & Prevention {CDC). Division of High Consequence Pathogens and Pathology (DHCPP-PRB)	Gigante,C.M., Buttery,E., Zhao,H., Batra,D., Hetrick,E.E., Howard,D.T., Kovar,L., Seabolt.M.H., Knipe,K., Burroughs,S.. Lee.J., Wilkins,K.. McCollum,A., Hutson,C., Davidson,W.. Rao.A.. Raman,☐.and Li.Y
EPI_ISL_15332327	Centers for Disease Contrai & Prevention (CDC). Division of High Consequence Pathogens and Pathology (DHCPP-PRB)	Centers for Disease Control & Prevention {CDC). Division of High Consequence Pathogens and Pathology (DHCPP-PRB)	Gigante.CM., Acheampong,E., Zhao,H., Batra,D., Hetrick,E.E., Howard,D.T.. Kovar,L.. Seabolt.M.H., Knipe,K., Burroughs.$., Lee,J., Wilkins,K., McCollum,A., Hutson,C., Davidson,W., Rao.A., McDermott.D. and Li,Y.
EPI_ISL_15332328	Centers for Disease Control & Prevention (CDC), Division of High Consequence Pathogens and Pathology (DHCPP-PRB)	Centers for Disease Control & Prevention {CDC). Division of High Consequence Pathogens and Pathology (DHCPP-PRB)	Gigante,C.M., Pettit,D., Zhao,H., Batra,D., Hetrick,E.E., Howard,D.T., Kovar,L., Seabolt,M.H., Knipe,K., Burroughs,$., Lee,J., Wilkins,K., McCollum,A., Hutson,C., Davidson,W., Rao,A., Deutsch-Feldman,M. and Li,Y.
EPI_ISL_15332329, EPI_ISL_15332330	Centers for Disease Contrai & Prevention (CDC), Division of High Consequence Pathogens and Pathology (DHCPP-PRB)	Centers for Disease Contrai & Prevention {CDC). Division of High Consequence Pathogens and Pathology (DHCPP-PRB)	Gigante,C.M., Myers.R.. Zhao,H., Batra.D., Hetrick.E.E.. Howard,D.T., Kovar,L., Seabolt,M.H., Knipe,K.. Burroughs,S.. Lee,J.. Wilkins.K.. McCollum,A., Hutson,C.. Davidson.W.. Rao,A., Blythe.D. and Li.Y.
EPI_ISL_15332331	Centers for Disease Contrai & Prevention (CDC), Division of High Consequence Pathogens and Pathology (DHCPP-PRB)	Centers for Disease Control & Prevention {CDC). Division of High Consequence Pathogens and Pathology (DHCPP-PRB)	Gigante,C.M., Pavlick,J., Zhao,H., Batra,D., Hetrick,E.E., Howard,D.T., Kovar,L., Seabolt,M.H., Knipe,K., Burroughs,S., Lee,J., Wilkins,K., McCollum,A., Hutson,C., Davidson,w., Rao,A., Parrott,T. and Li,Y.
EPI_JSL_15332332, EPI_ISL_15332333	Centers for Disease Contrai & Prevention (CDC), Division of High Consequence Pathogens and Pathology (DHCPP-PRB)	Centers for Disease Control & Prevention {CDC). Division of High Consequence Pathogens and Pathology (DHCPP-PRB)	Gigante,C.M., Lee,P., Zhao,H., Batra,D., Hetrick,E.E., Howard,D.T., Kovar,L., Seabolt,M.H., Knipe,K., Burroughs.S., Lee,J., Wilkins,K., McCollum,A., Hutson,C., Davidson,W., Rao.A., Stanek,D. and Li.Y
EPI_ISL_15332334	Centers for Disease Contrai & Prevention (CDC), Division of High Consequence Pathogens and Pathology (DHCPP-PRB)	Centers for Disease Control & Prevention (CDCl. Division of High Consequence Pathogens and Pathology (DHCPP-PRB)	Gigante,C.M., Hauser,J.R., Zhao,H., Batra,D., Hetrick,E.E., Howard,D.T., Kovar,L., Seabolt,M.H., Knipe,K., Burroughs,S., Lee,J., Wilkins,K., McCollum,A., Hutson,C., Davidson,W., Rao.A., Mangla,A. and Li,Y.
EPI_ISL_15332335	Centers for Disease Control & Prevention (CDC), Division of High Consequence Pathogens and Pathology (DHCPP-PRB)	Centers for Disease Control & Prevention (CDC). Division of High Consequence Pathogens and Pathology (DHCPP-PRB)	Gigante,C.M., Ventura,J., Zhao,H., Batra,D., Hetrick,E.E., Howard,D.T., Kovar,L., Seabolt,M.H., Knipe,K., Burroughs,S., Lee,J., Wilkins,K., McCollum,A., Hutson,C., Davidson,W., Rao.A., Nash,J. and li.Y
EPI_ISL_15332336, EPI_ISL_15332337	Environmental, Agricultural. and Occupational Health. University of Nebraska Medical Center	Environmental, Agricultural, and Occupational Health. University of Nebraska Medical Center	Tegomoh.B., Cross.S.T., Chapman,R.C., Bernhard,K., McCutchen,E.L., Fauver,J.R., Pratt,C.B., Warden,D.E., Iwen,P.C., Donahue,M. and Wiley,M.R.
EPI_ISL_15332338, EPI_ISL_15332339, EPI_ISL_15332340	Environmental, Agricultural, and Occupational Health, University of Nebraska Medical Center	Environmental, Agricultural, and Occupational Health, University of Nebraska Medical Center	Chapman,R.C., Bernhard,K., McCutchen,E.L., Fauver,J.R., O'Dell,J.X., Mannell,M., Wiley,M.R. and Cross,S.T.
EPI_ISL_15352249, EPI_ISL_15352251, EPJ_ISL_15352252. EPI_JSL_15352254. EPI_ISL_15352256, EPI_ISL_15352258, EPI_ISL_15352259, EPI_ISL_15352262. EPI_ISL_15352263. EPI_ISL_15352265, EPI_ISL_15352267, EPI_ISL_15352268, EPI_ISL_15352271. EPI_ISL_15352276. EPI_ISL_15352278, EPI_ISL_15352279, EPI_ISL_15352280, EPI_ISL_15352282. EPI_ISL_15352283, EPI_ISL_15352284, EPI_ISL_15352285, EPI_ISL_15352287, EPI_ISL_15352289, EPI_ISL_15352291, EPI_ISL_15352293, EPI_ISL_15352297, EPI_ISL_15352299, EPI_ISL_15352300, EPI_ISL_15352302, EPI_ISL_15352305, EPI_ISL_15352307, EPI_ISL_15352308, EPI_ISL_15352310, EPI_ISL_15352311, EPI_ISL_15352312, EPI_ISL_15352314			
see above	Centre for Biological Threats, Highly Pathogenic Viruses, Robert Koch Institute	Centre for Biological Threats, Highly Pathogenic Viruses, Robert Koch Institute	Brinkmann,A., Kohl,C., Pape,K., Schrick,L., Michel,)., Schaade,L. and Nitsche,A
EPI ISL 15367957, EPI ISL 15367958, EPI ISL 15367959, EPI ISL 15367960, EPI ISL 15367961. EPI ISL 15367962, EPI ISL 15367963, EPI ISL 15367964, EPI ISL 15367965, EPI ISL 15367966, EPI ISL 15367967, EPI ISL 15367968, EPI ISL 15367969, EPI ISL 15367970, EPI ISL 15367971, EPI ISL 15367972, EPI ISL 15367973, EPI ISL 15367974, EPI ISL 15367975, EPI ISL 15367976, EPI=ISL=15367977, EPI=ISL)5367978, EPÜSL)5367979, EPI=JSL)5367980, EPI=ISL)5367981, EPI=ISL=15367982, EPI=ISL)5367983, EPÜSL)5367984, EPI=ISL)5367985, EPI=ISL)5367986, EPI=ISL=15367987, EPI=ISL)5367988, EPÜSL)5367989, EPI=ISL)5367990, EPI=ISL)5367991, EPI=ISL=15367992, EPI=ISL)5367993, EPÜSL)5367994, EPI=ISL)5367995, EPI=ISL)5367996, EPI_ISL_15367997, EPI_I5L_15367998, EPJ_ISL_15367999, EPI_JSL_15368000, EPI_ISL_15368001, EPI_ISL_15368002, EPI_ISL_15368003, EPI_ISL_15368004, EPI_ISL_15368005, EPI_ISL_15368006, EPI_ISL_15368007, EPI_ISL_15368008, EPI_ISL_15368009, EPI_ISL_15368010, EPI_ISL_15368011			
see above	Laboratory Medicine, UW Virology	Laboratory Medicine. UW Virology	Sereewit.J.. Xie.H.. Roychoudhury,P. and Greninger.A.L.
EPI_ISL_15370026, EPI_ISL_15370027, EPJ_ISL_15370028, EPI_ISL_15370030, EPI_ISL_15370031, EPI_ISL_15370043, EPI_ISL_15370044, EPI_ISL_15370045, EPI_ISL_1537004 7, EPI_ISL_15370048, EPI_ISL_15370050, EPI_ISL_15370053, EPI_ISL_15370056, EPI_ISL_15370057, EPI_ISL_15370058, EPI_ISL_15370059, EPI_ISL_15370060, EPI_ISL_15370061			
see above	Laboratory for Diagnostics of zoonoses and WHO Centre. Institute of Microbiology and Immunology, Faculty of Medicine, University of Ljubljana	Laboratory for Diagnostics of zoonoses and WHO Centre, Institute of Microbiology and Immunology, Faculty of Medicine, University of Ljubljana	Zakotnik,S., Vlaj.D., Suljic,A., zorec,T.M., Korva,M., Poljak,M. and Avsic Zupanc,T.
EPI_ISL_15370065, EPI_ISL_15370066, EPI_ISL_15370068, EPI_ISL_15370069, EPI_ISL_15370070, EPI_ISL_15370071. EPI_ISL_15370072, EPI_ISL_15370073, EPI_ISL_15370074, EPI_ISL_15370075, EPI_ISL_15370076, EPI_ISL_15370077, EPI_ISL_15370078, EPI_ISL_15370079, EPI_ISL_15370080, EPI_ISL_15370081. EPI_ISL_15370082			
see above	Nigeria Centre for Disease Control, National Reference Laboratory	Chemical. Biological and Radiological Sciences. Defence Science and Technology Laboratory	Ndodo,N., Ashcroft.J., Lewandowski,K. Yinka-Ogunleye,A.. Chukwu.C.. Ahmad.A., King.☐., Akinpelu.A., Maluquer de Motes,C., Ribeca,P.. Summer,R.P., Rambaut,A., Chester,M., Maishman.T.. Babatunde,O., Mba,N., Babatunde,O.. Aruna,O., Pullan,S.T., Gannon,B., Brown,C., Ihekweazu,C., Adetifa,I. and Ulaeto,D.O.
EPI_ISL_15373792	Department of immunology and microbiology - Pasteur Institute in Ho Chi Minh city	Department of immunology and microbiology - Pasteur Institute in Ho Chi Minh city	Manh H. Dao, Nhung H. P. Vu, Hang T. T. Pham, Thang M. Cao, Thinh V. Nguyen, Quang D. Pham, Quang C. Luong, Trung V. Nguyen
EPI_ISL_15380492	Eastwood Medical City	Molecular Biology Laboratory, Research Institute for Tropical Medicine	Samantha Louise P. Bado, Niquitta B. Galap, Bea C. Mateo, Chelsea Mae M. Reyes, Amalea Dulcene Nicolasora, Miguel Francisco B. Abulencia, Francisco Gerardo M. Polotan on behalf of the Research Institute for Tropical Medicine
EPI_ISL_15384398, EPI_ISL_15384399, EPI_ISL_15384400, EPI_ISL_15384401, EPI_ISL_15384402, EPI_ISL_15384403, EPI_ISL_15384404, EPI_ISL_15384405, EPI_ISL_15384406, EPI_ISL_l5384407, EPI_ISL_15384408			
see above	Laboratory Medicine, UW Virology	Laboratory Medicine, UW Virology	Sereewit.J, Xie,H., Roychoudhury,P. and Greninger,A.L.
EPI_ISL_15384410	Sequencing/Bioinformatics, Delaware Public Health Lab	Sequencing/Bioinformatics, Delaware Public Health Lab	Bajwa,M.I. and Miller,H.
EPI ISL 15384418, EPI ISL 15384419, EPI ISL 15384420, EPI ISL 15384421, EPI ISL 15384422, EPI ISL 15384423, EPI ISL 15384424, EPI ISL 15384425, EPI ISL 15384426, EPI ISL 15384427, EPI ISL 15384428, EPI ISL 15384429, EPI ISL 15384430, EPI ISL 15384431, EPI ISL 15384432, EPI ISL 15384433, EPI ISL 15384435, EPI ISL 15384436, EPI ISL 15384437, EPI ISL 15384438, EPI=ISL)5384439			
see above	Centre for Biological Threats, Highly Pathogenic Viruses, Robert Koch Institute	Centre for Biological Threats, Highly Pathogenic Viruses, Robert Koch Institute	Brinkmann,A., Kohl,C., Pape,K., Schrick,L., Michel,)., Schaade,L. and Nitsche,A.

EPI ISL 15390495, EPI ISL 15390496, EPI=ISL=I5390497, EPI=JSL=I5390498, EPI ISL 15390499, EPI ISL 15390500, - - EPI_ISL_I539050-I	Centre for Biological Threats, Highly Pathogenic Viruses, Robert Koch Institute	Centre for Biological Threats, Highly Pathogenic Viruses, Robert Koch Institute	Brinkmann,A., Kohl,C, Pape,K., Uddin,S., Schrick,L., Michel,)., Schaade,L. and Nitsche,A.
EPI_ISL_I5390502	Division of High-Consequence Pathogens and Pathology (DHCPP-PRB), CDC	Division of High-Consequence Pathogens and Pathology {DHCPP-PRB), CDC	Gigante,C.M., Pavlick,J., Zhao,H., Batra,D., Hetrick,E.E., Howard,D.T., Kovar,L., Seabolt,M.H., Weigand,M.R., Burroughs,M., Lee,J., Wilkins,K., McCollum,A., Hutson,C, Davidson,W., Rao.A., Parrott,T. and Li,Y.
EPI_ISL_15412318, EPI_ISL_15412320, EPI_ISL_15412322, EPI_ISL_15412324, EPI_ISL_15412326, EPI_ISL_I5412328, EPI_ISL_15412330, EPI_ISL_15412331, EPI_ISL_15412333, EPI_ISL_15412335, EPI_ISL_15412336, EPI_ISL_15412338			
see above	CT Department of Public Health	CT Department of Public Health	Claire Pearson, Tu N. Nguyen, Kutluhan Incekara, Neranjan V. Perera
EPI_ISL_I5415308	Hospital Clfnica Bfblica	Inciensa, Instituto de Investigaci6n y Enserïanza en Nutrici6n y Salud	Francisco Quarte, Ana Isela Ruiz-Gonzalez, Hillary Serrano, Diana Cantillo, Claudio Soto-Garita, Gustavo Vega, Estela Cordera, Adriana Godinez & Melany Calderon
EPI ISL 15417986, EPI ISL 15417987, EPI ISL 15417989, EPI ISL 15417990, EPI ISL 15417991. EPI ISL 15417992, EPI ISL 15417993, EPI ISL 15417994, EPI ISL 15417995, EPI ISL 15417996, EPI ISL 15417997, EPI ISL 15417998, EPI ISL 15417999, EPI ISL 15418000, EPI ISL 15418001. EPI ISL 15418002, EPI ISL 15418003, EPI ISL 15418004, EPI ISL 15418005, EPI ISL 15418006, EPI=ISL)5418007, EPI=ISL)5418008, EPÜSL)5418009, EPI=JSL)5418010, EPI=ISL)5418011, EPI=ISL)5418012			
see above	Los Angeles County Public Health Laboratories	Los Angeles County Public Health Laboratories	P. Hemarajata et al.
EPI_ISL_I5419131	Instituto de Infectologia Emilio Ribas	Instituto Adolfo Lutz Strategic Laboratory	Claudio Tavares Sacchi, Karoline Rodrigues Campos, Ariadne Ferreira Amarante, Marion Benedito Nascimento Santos, Adriano Abbud, Adriana Bugno
EPI_ISL_I5419132	Centra de Saude de Sao Roque Dr Jose Carvalho Brito	Instituto Adolfo Lutz Strategic Laboratory	Claudio Tavares Sacchi. Karoline Rodrigues Campos, Ariadne Ferreira Amarante, Marion Benedito Nascimento Santos, Adriano Abbud, Adriana Bugno
EPI_ISL_I5419133	UBS II COHAB Presidente Prudente	Instituto Adolfo Lutz Strategic Laboratory	Claudio Tavares Sacchi, Karoline Rodrigues Campos, Ariadne Ferreira Amarante, Marion Benedito Nascimento Santos, Adriano Abbud, Adriana Bugno
EPI_ISL_I5419134	CTA Centra de Testagem e Aconselhamento de Caieiras	Instituto Adolfo Lutz Strategic Laboratory	Claudio Tavares Sacchi, Karoline Rodrigues Campos, Ariadne Ferreira Amarante, Marion Benedito Nascimento Santos, Adriano Abbud, Adriana Bugna
EPI_ISL_I5419135	SAE OST AIDS Cidade Outra	Instituto Adolfo Lutz Strategic Laboratory	Claudio Tavares Sacchi, Karoline Rodrigues Campos, Ariadne Ferreira Amarante, Marion Benedito Nascimento Santos, Adriano Abbud, Adriana Bugno
EPI_ISL_I5419136	UBS J Nordeste	Instituto Adolfo Lutz Strategic Laboratory	Claudio Tavares Sacchi, Karoline Rodrigues Campos, Ariadne Ferreira Amarante, Marion Benedito Nascimento Santos, Adriano Abbud, Adriana Bugna
EPI_ISL_15419137	CTA Centro de Testagem e Aconselhamento Favo de Mel	Instituto Adolfo Lutz Strategic Laboratory	Claudio Tavares Sacchi, Karoline Rodrigues Campos, Ariadne Ferreira Amarante, Marion Benedito Nascimento Santos, Adriano Abbud, Adriana Bugno
EPI_ISL_I5419138	Vigilancia Epidemiologica e Controle de Vetores de Pirassununga	Instituto Adolfo Lutz Strategic Laboratory	Claudio Tavares Sacchi, Karoline Rodrigues Campos, Ariadne Ferreira Amarante, Marion Benedito Nascimento Santos, Adriano Abbud, Adriana Bugno
EPI_ISL_I5419139	Policlinica Maria Dirce	Instituto Adolfo Lutz Strategic Laboratory	Claudio Tavares Sacchi, Karoline Rodrigues Campos, Ariadne Ferreira Amarante, Marion Benedito Nascimento Santos, Adriano Abbud, Adriana Bugno
EPI_ISL_I5419140	Secretaria Municipal de Saude de Batatais SP	Instituto Adolfo Lutz Strategic Laboratory	Claudio Tavares Sacchi, Karoline Rodrigues Campos, Ariadne Ferreira Amarante, Marion Benedito Nascimento Santos, Adriano Abbud, Adriana Bugno
EPI_ISL_I5419141	UBS J Nordeste	Instituto Adolfo Lutz Strategic Laboratory	Claudio Tavares Sacchi, Karoline Rodrigues Campos, Ariadne Ferreira Amarante, Marion Benedito Nascimento Santos, Adriano Abbud, Adriana Bugna
EPI_ISL_I5419142	Instituto de Infectologia Emilio Ribas	Instituto Adolfo Lutz Strategic Laboratory	Claudio Tavares Sacchi, Karoline Rodrigues Campos, Ariadne Ferreira Amarante, Marion Benedito Nascimento Santos, Adriano Abbud, Adriana Bugno
EPI_ISL_I5419143	Santa Casa de Barretos	Instituto Adolfo Lutz Strategic Laboratory	Claudio Tavares Sacchi, Karoline Rodrigues Campos, Ariadne Ferreira Amarante, Marion Benedito Nascimento Santos, Adriano Abbud, Adriana Bugno
EPI_ISL_I5419144	Hospital Vera Cruz	Instituto Adolfo Lutz Strategic Laboratory	Claudio Tavares Sacchi, Karoline Rodrigues Campos, Ariadne Ferreira Amarante, Marion Benedito Nascimento Santos, Adriano Abbud, Adriana Bugno
EPI_ISL_I5419145	NotreDame Intermedica Saude	Instituto Adolfo Lutz Strategic Laboratory	Claudio Tavares Sacchi, Karoline Rodrigues Campos, Ariadne Ferreira Amarante, Marion Benedito Nascimento Santos, Adriano Abbud, Adriana Bugna
EPI_ISL_I5419146	Hospital e Maternidade Santa Maria Cruz Azul	Instituto Adolfo Lutz Strategic Laboratory	Claudio Tavares Sacchi, Karoline Rodrigues Campos, Ariadne Ferreira Amarante, Marion Benedito Nascimento Santos, Adriano Abbud, Adriana Bugna
EPI_ISL_I5419147	Pronto Socorro Central de Diadema	Instituto Adolfo Lutz Strategic Laboratory	Claudio Tavares Sacchi, Karoline Rodrigues Campos, Ariadne Ferreira Amarante, Marion Benedito Nascimento Santos, Adriano Abbud, Adriana Bugno
EPI_ISL_I5419148	NotreDame Intermedica Saude Santo Andre	Instituto Adolfo Lutz Strategic Laboratory	Claudio Tavares Sacchi, Karoline Rodrigues Campos, Ariadne Ferreira Amarante, Marion Benedito Nascimento Santos, Adriano Abbud, Adriana Bugno
EPI_ISL_I5419149	UBS Dom Angelico	Instituto Adolfo Lutz Strategic Laboratory	Claudio Tavares Sacchi, Karoline Rodrigues Campos, Ariadne Ferreira Amarante, Marion Benedito Nascimento Santos, Adriano Abbud, Adriana Bugno
EPI_ISL_I5419150	Secao de Centra de Diagnostico SECEDI	Instituto Adolfo Lutz Strategic Laboratory	Claudio Tavares Sacchi, Karoline Rodrigues Campos, Ariadne Ferreira Amarante, Marion Benedito Nascimento Santos, Adriano Abbud, Adriana Bugna
EPI_ISL_I5419151	UPA 24H Brotas	Instituto Adolfo Lutz Strategic Laboratory	Claudio Tavares Sacchi, Karoline Rodrigues Campos, Ariadne Ferreira Amarante, Marion Benedito Nascimento Santos, Adriano Abbud, Adriana Bugno
EPI_ISL_I5419152	Hospital Nossa Senhora de Lourdes	Instituto Adolfo Lutz Strategic Laboratory	Claudio Tavares Sacchi, Karoline Rodrigues Campos, Ariadne Ferreira Amarante, Marion Benedito Nascimento Santos, Adriano Abbud, Adriana Bugno
EPI_ISL_I5419153	AMA Paraisopolis	Instituto Adolfo Lutz Strategic Laboratory	Claudio Tavares Sacchi, Karoline Rodrigues Campos, Ariadne Ferreira Amarante, Marion Benedito Nascimento Santos. Adriano Abbud. Adriana Bugno
EPI_ISL_I5419154	Pronto Atendimento Infantil e Central de Quimioterapia de Sao Jose do Rio Preto	Instituto Adolfo Lutz Strategic Laboratory	Claudio Tavares Sacchi, Karoline Rodrigues Campos, Ariadne Ferreira Amarante, Marion Benedito Nascimento Santos, Adriano Abbud, Adriana Bugno
EPI_ISL_I5419155	Santa Casa de Atibaia Pro Saude	Instituto Adolfo Lutz Strategic Laboratory	Claudio Tavares Sacchi, Karoline Rodrigues Campos, Ariadne Ferreira Amarante, Marion Benedito Nascimento Santos. Adriano Abbud. Adriana Bugno
EPI_ISL_I5419156	UPA Vila Mariana	Instituto Adolfo Lutz Strategic Laboratory	Claudio Tavares Sacchi, Karoline Rodrigues Campos, Ariadne Ferreira Amarante, Marion Benedito Nascimento Santos, Adriano Abbud, Adriana Bugno
EPI_ISL_I5419157	Secretaria de Saude de Mogi das Cruzes	Instituto Adolfo Lutz Strategic Laboratory	Claudio Tavares Sacchi, Karoline Rodrigues Campos, Ariadne Ferreira Amarante, Marion Benedito Nascimento Santos, Adriano Abbud, Adriana Bugna
EPI_ISL_I5419158	Unidade Mista de saude Mariano Gayoso Castelo Branco	Instituto Adolfo Lutz Strategic Laboratory	Claudio Tavares Sacchi, Karoline Rodrigues Campos, Ariadne Ferreira Amarante, Marion Benedito Nascimento Santos, Adriano Abbud, Adriana Bugno
EPI_ISL_I5419159	Hospital Vivalle	Instituto Adolfo Lutz Strategic Laboratory	Claudio Tavares Sacchi, Karoline Rodrigues Campos, Ariadne Ferreira Amarante, Marion Benedito Nascimento Santos, Adriano Abbud, Adriana Bugna
EPI_ISL_I5419160	UPA Nova Hortolandia Manoel Geogino Lopes	Instituto Adolfo Lutz Strategic Laboratory	Claudio Tavares Sacchi, Karoline Rodrigues Campos, Ariadne Ferreira Amarante, Marion Benedito Nascimento Santos, Adriano Abbud, Adriana Bugno
EPI_ISL_I5419161	Centra de Saude I Albertina Affonso Jaboticabal	Instituto Adolfo Lutz Strategic Laboratory	Claudio Tavares Sacchi. Karoline Rodrigues Campos, Ariadne Ferreira Amarante, Marion Benedito Nascimento Santos, Adriano Abbud, Adriana Bugno
EPI_ISL_I5419162	SAE DST AIDS M Boi Mirim Servico de Atencao Especializada	Instituto Adolfo Lutz Strategic Laboratory	Claudio Tavares Sacchi, Karoline Rodrigues Campos, Ariadne Ferreira Amarante, Marion Benedito Nascimento Santos. Adriano Abbud. Adriana Bugno
EPI_ISL_I5419163	Instituto de Infectologia Emilio Ribas	Instituto Adolfo Lutz Strategic Laboratory	Claudio Tavares Sacchi, Karoline Rodrigues Campos, Ariadne Ferreira Amarante, Marion Benedito Nascimento Santos, Adriano Abbud, Adriana Bugno
EPI_ISL_I5433343, EPI_ISL_I5433344, EPI ISL 15433345, EPI ISL 15433346, EPI ISL I5433347, EPC_ISL I5433348	Laboratory Medicine, uw Virology	Laboratory Medicine. uw Virology	Sereewit.J., Xie,H.. Roychoudhury.P. and Greninger,A.L.
EPI_ISL_I5455851	Centers for Disease Contrai & Prevention (CDC), Division of High Consequence Pathogens and Pathology (DHCPP-PRB)	Centers for Disease Contrai & Prevention {CDC). Division of High Consequence Pathogens and Pathology (DHCPP-PRB)	Gigante.CM., Francis,D., Zhao,H., Batra,D., Hetrick,E.E., Howard,D.T., Kovar,L., Morrison,S.S. Seabolt,M.H., Weigand,M.R., Knipe,K. Sheth,M., Burgin,A., Burroughs,M., Lee,J., Wilkins,K., McCollum,A., Hutson,C, Davidson,W., Rao.A., Escobar,). and Li,Y.
EPI_ISL_I5455852, EPI_ISL_I5455853	Centers for Disease Contrai & Prevention (CDC). Division of High Consequence Pathogens and Pathology (DHCPP-PRB)	Centers for Disease Contrai & Prevention {CDC). Division of High Consequence Pathogens and Pathology (DHCPP-PRB)	Gigante,C.M., Ventura,J.. Zhao.H., Batra,D.. Hetrick,E.E.. Howard.D.T.. Kovar,L., Morrison,S.S., Seabolt.M.H.. Weigand,M.R., Knipe,K.. Sheth,M.. Burgin,A., Burroughs.M., Lee,J., Wilkins,K.. McCollum,A., Hutson.C., Davidson,W.. Rao.A.. Nash,J. and Li,Y.
EPI_ISL_I5455854	Centers for Disease Contrai & Prevention (CDC), Division of High Consequence Pathogens and Pathology (DHCPP-PRB)	Centers for Disease Contrai & Prevention {CDC). Division of High Consequence Pathogens and Pathology (DHCPP-PRB)	Gigante.CM., Maloney,M., Zhao,H., Batra,D., Hetrick,E.E., Howard,D.T., Kovar,L., Morrison,S.S. Seabolt,M.H., Weigand,M.R., Knipe,K. Sheth,M., Burgin,A., Burroughs,M., Lee,J., Wilkins,K., McCollum,A., Hutson,C, Davidson,w., Rao.A., Pearson,C and Li,Y.
EPI ISL 15455855, EPI ISL 15455856, - - EPI_ISL_I545585-7	Centers for Disease Contrai & Prevention (CDC), Division of High Consequence Pathogens and Pathology (DHCPP-PRB)	Centers for Disease Contrai & Prevention {CDC). Division of High Consequence Pathogens and Pathology (DHCPP-PRB)	Gigante.CM., Lee,P., Zhao,H., Batra.D., Hetrick,E.E., Howard,D.T., Kovar,L., Morrison.S.S., Seabolt,M.H, Weigand,M.R., Knipe,K., Sheth,M., Burgin,A., Burroughs,M., Lee,J., Wilkins,K., McCollum,A., Hutson,C, Davidson,W., Rao.A., Stanek,D. and Li,Y.
EPI_ISL_I5455858	Centers for Disease Contrai & Prevention (CDC), Division of High Consequence Pathogens and Pathology (DHCPP-PRB)	Centers for Disease Contrai & Prevention {CDC). Division of High Consequence Pathogens and Pathology (DHCPP-PRB)	Gigante.CM., Johnson,S., Zhao,H., Batra,D., Hetrick,E.E., Howard,D.T., Kovar,L., Morrison,S.S. Seabolt,M.H., Weigand,M.R., Knipe,K. Sheth,M., Burgin,A., Burroughs,M., Lee,J., Wilkins,K., McCollum,A., Hutson,C, Davidson,W., Rao.A., Riner,D. and Li.Y.
EPI_ISL_I5455859	Centers for Disease Contrai & Prevention (CDC), Division of High Consequence Pathogens and Pathology (DHCPP-PRB)	Centers for Disease Contrai & Prevention {CDC). Division of High Consequence Pathogens and Pathology (DHCPP-PRB)	Gigante.CM., Ostadkar,R., Zhao,H., Batra,D., Hetrick,E.E., Howard,D.T., Kovar,L., Morrison,S.S, Seabolt.M.H., Weigand,M.R., Knipe,K., Sheth,M., Burgin,A., Burroughs,M., Lee,J., Wilkins,K., McCollum,A., Hutson,C, Davidson,W., Rao.A., Wang.X. and Li.Y.
EPI_ISL_I5455860	Centers for Disease Contrai & Prevention (CDC), Division of High Consequence Pathogens and Pathology (DHCPP-PRB)	Centers for Disease Contrai & Prevention {CDC). Division of High Consequence Pathogens and Pathology (DHCPP-PRB)	Gigante,C.M., Pettit,D., Zhao,H., Batra,D., Hetrick,E.E., Howard,D.T., Kovar,L., Morrison,S.S, Seabolt,M.H., Weigand,M.R., Knipe,K., Sheth,M., Burgin,A., Burroughs.M., Lee,J., Wilkins,K., McCollum,A., Hutson.C., Davidson.W., Rao.A., Deutsch-Feldman,M. and Li,Y.
EPI_ISL_I5455861	Centers for Disease Contrai & Prevention (CDC), Division of High Consequence Pathogens and Pathology (DHCPP-PRB)	Centers for Disease Contrai & Prevention {CDC). Division of High Consequence Pathogens and Pathology (DHCPP-PRB)	Gigante,C.M., Pettit.D., Zhao,H., Batra,O., Hetrick,E.E., Howard,D.T., Kovar,L., Morrison,S.S., Seabolt.M.H., Weigand,M.R., Knipe,K., Sheth,M., Burgin,A., Burroughs,M., Lee,J., Wilkins,K., McCollum,A., Hutson,C., Oavidson,W., Rao.A., Oeutsch-Feldman,M. and Li,Y
EPI_JSL_I5455862, EPI_ISL_I5455863	Centers for Disease Contrai & Prevention (CDC), Division of High Consequence Pathogens and Pathology (DHCPP-PRB)	Centers for Disease Contrai & Prevention {CDC). Division of High Consequence Pathogens and Pathology (DHCPP-PRB)	Gigante.CM., Acheampong,E., Zhao,H., Batra,D., Hetrick,E.E., Howard,O.T., Kovar,L., Morrison.S.S., Seabolt,M.H., Weigand,M.R., Knipe,K., Sheth,M., Burgin,A., Burroughs,M., Lee,J., Wilkins,K., McCollum.A., Hutson,C, Davidson,W., Rao.A., McDermott.D. and li.Y.
EPI ISL 15455864, EPI ISL 15455865, - - EPI_ISL_I545586-6	Centers for Disease Contrai & Prevention (CDC), Division of High Consequence Pathogens and Pathology (DHCPP-PRB)	Centers for Disease Contrai & Prevention {CDC). Division of High Consequence Pathogens and Pathology (DHCPP-PRB)	Gigante.CM., Ruiz.V., Zhao,H., Batra,D., Hetrick,E.E., Howard,D.T., Kovar,L., Morrison,S.S., Seabolt,M.H, Weigand,M.R., Knipe,K., Sheth,M., Burgin,A., Burroughs,M., Lee,J., Wilkins,K., McCollum,A., Hutson,C, Oavidson,W., Rao.A., Wang,J. and Li,Y.
EPI_ISL_I5455867	Centers for Disease Contrai & Prevention (CDC),	Centers for Disease Contrai & Prevention {CDC).	Gigante,CM.. Lee,B.. Zhao,H., Batra,D.. Hetrick,E.E., Howard,D.T., Kovar,L., Morrison,S.S., Seabolt.M.H., Weigand,M.R., Knipe,K., Sheth,M.. Burgin,A., Burroughs,M., Lee,J.. Wilkins,K., McCollum,A., Hutson,C.. Davidson.W.. Rao.A..

	Division of High Consequence Pathogens and Pathology (DHCPP-PRB)	Division of High Consequence Pathogens and Pathology (DHCPP-PRB)	Salehi,E. and Li,Y.
EPI_ISL_I5455868	Centers for Disease Contrai & Prevention (CDC), Division of Hi9h Consequence Pathogens and Pathology (DHCPP-PRB)	Centers for Disease Contrai & Prevention {CDC). Division of High Consequence Pathogens and Pathology (DHCPP-PRB)	Gigante,C.M., Berns,A., Zhao,H., Batra,D., Hetrick,E.E., Howard,D.T., Kovar,L., Morrison,5.5, Seabolt,M.H., Weigand,M.R., Knipe,K., Sheth,M, Burgin,A., Burraughs,M., Lee,J., Wilkins,K., McCollum,A., Hutson,C., Davidson,W., Rao.A., Carpenter-Azevedo,K. and Li,Y.
EPI_ISL_I5455869, EPI_ISL_I5455870	Centers for Disease Contrai & Prevention (CDC), Division of High Consequence Pathogens and Pathology (DHCPP-PRB)	Centers for Disease Contrai & Prevention {CDC). Division of High Consequence Pathogens and Pathology (DHCPP-PRB)	Gigante.CM., Kubin,G., Zhao,H., Batra,D., Hetrick,E.E., Howard,D.T., Kovar,L., Morrison.S.S. Seabolt,M.H., Weigand,M.R., Knipe,K. Sheth,M., Burgin,A., Burraughs,M., Lee,J., Wilkins,K., McCollum,A., Hutson,C, Davidson,W., Rao.A., White,S.L. and Li,Y.
EPI_ISL_I5455871, EPI_JSL_I5455872, EPI_ISL_I5455873, EPI_ISL_I5455874	Centers for Disease Contrai & Prevention (CDC), Division of High Consequence Pathogens and Pathology (DHCPP-PRB)	Centers for Disease Contrai & Prevention {CDC). Division of High Consequence Pathogens and Pathology (DHCPP-PRB)	Brinkmann,A., Kohl,C., Pape,K., Schrick,L., Michel,J., Schaade,L. and Nitsche,A.
EPI ISL 15455B75, EPI ISL 15455876, EPI ISL 15455B77, EPI ISL 1545587B, EPI ISL 15455879, EPI ISL 15455880, EPI ISL 15455881, EPI ISL 15455882, EPI ISL 15455883, EPI ISL 15455884, EPI ISL 15455885, EPI ISL 15455886, EPI ISL 15455887, EPI ISL 15455888, EPI ISL 15455889, EPI ISL 15455890, EPI ISL 15455891, EPI ISL 15455892, EPI ISL 15455893, EPI ISL 15455894, EPI=ISL)5455895, EPI=ISL)5455896, EPÜSL)5455897, EPI=JSL)5455898, EPI=ISL)5455899, EPI=ISL)5455900, EPI=ISL)5455901, EPÜSL)54 55902, EPI=ISL)5455903, EPI=ISL)5455904, EPI=ISL)5455905, EPI=ISL)5455906, EPÜSL)5455907			
see above	Centre for Biological Threats, Highly Pathogenic Viruses. Robert Koch Institute	Centre for Biological Threats, Highly Pathogenic Viruses, Robert Koch Institute	Brinkmann,A., Kohl,C., Pape,K., Schrick,L., Michel,]., Schaade,L. and Nitsche,A
EPI_ISL_I5455914, EPI_JSL_I5455916, EPI_ISL_15455917	Direccion de Investigacion en Salud Publica, Instituto Nacional de Salud	Direccion de Investigacion en Salud Publica, Instituto Nacional de Salud	Laiton-Donato,K., Alvarez-Diaz,D.A., Franco-Munoz,C., Ruiz-Moreno,H.A., Rojas-Estevez,P., Rosales,A., Martinez,D., Flores.A., Prieto,F., Walteros,D., Gomez,S. and Mercado-Reyes,M.
EPJ_ISL_I5458903, EPI_ISL_15458904, EPI_ISL_I5458905, EPI_ISL_I5458906	Nebraska Public Health Laboratory (NPHL)	Environmental, Agricultural, and Occupational Health, University of Nebraska Medical Center	Chapman,R.C.. Bernhard,K., McCutchen,E.L.. Fauver,J.R.. O'Dell.J.X., Mannell,M., Wiley,M.R. and Cross,S.T.
EPJ_ISL_I5458907, EPI_JSL_15500242, EPI_ISL_15500243	Laboratory Medicine, UW Virology	Laboratory Medicine, UW Virology	Sereewit,J., Xie,H., Roychoudhury,P. and Greninger,A.L.
EPI ISL 15500244, EPI ISL 15500245, EPI ISL 15500246, EPI ISL 15500247, EPI ISL 15500248, EPI ISL 15500249, EPI ISL 15500250, EPI ISL 15500251, EPI ISL 15500252, EPI ISL 15500253, EPI ISL 15500254, EPI ISL 15500255, EPI ISL 15500256, EPI ISL 15500257, EPI ISL 15500258, EPI ISL 15500259, EPI ISL 15500260, EPI ISL 15500261, EPI ISL 15500262, EPI ISL 15500263, EPI=ISL)5500264			
see above	Centre for Biological Threats, Highly Pathogenic Viruses, Robert Koch Institute	Centre for Biological Threats, Highly Pathogenic Viruses, Robert Koch Institute	Brinkmann,A., Kohl,C., Pape,K., Schrick,L., Michel,]., Schaade,L. and Nitsche,A
EPI ISL 15502320, EPI ISL 15502321, EPJ ISL 15502322, EPI JSL 15502324, EPI ISL 15502325, EPI ISL 15502326, EPI ISL 15502327, EPI ISL 15502328, EPI ISL 15502329, EPI ISL 15502330, EPI ISL 15502331, EPI ISL 15502332, EPI ISL 15502333, EPI ISL 15502334, EPI ISL 15502335, EPI ISL 15502336, EPI ISL 15502337, EPI ISL 15502338, EPI ISL 15502339, EPI ISL 15502340, EPCISL)5502341. EPI=ISL)5502342, EPÜSL)5502343			
see above	Los Angeles County Public Health Laboratories	Los Angeles County Public Health Laboratories	P. Hemarajata et al.
EPI_ISL_I5528148	Department of Clinical Sciences, Institute of Tropica Medicine	Department of Clinical Sciences, Institute ofTropica Medicine	Berens-Riha,N., De Block,T., Rutgers,J., Van Gestel,L., Hens,M., Kenyon,C , Soentjens,P., Van Griensven,J., Brosius,I , Arien,K., Van Esbroeck,M., Rezende,A.M., Vercauteren,K. and Liesenborghs,L.
EPI_ISL_I5528149, EPI_JSL_15528150, EPI_ISL_I5528151	Department of Clinical Sciences, Institute of Trapica Medicine	Department of Clinical Sciences, Institute ofTrapica Medicine	Berens-Riha,N., De Block,T., Rutgers,J., Van Gestel,L., Hens,M., Kenyon,C., Soentjens,P., Van Griensven,J., Brasius,I., Arien,K., Van Esbraeck,M., Rezende,A.M. and Vercauteren,K.
EPJ_ISL_I5587960, EPI_ISL_15587961, EPI ISL 15587962, EPI JSL 15587963, EPI=ISL=15587964, EPI=ISL)5587965, EPI ISL 15587966, EPI 15L 15587967, EPI JSL I5587968, EPC_ISL I5587969	Utah Public Health Laboratory	Utah Public Health Laboratory	lverson.T.. Oakeson,K.F., Young,E.L., Wagner,]. and Hergert,J.
EPI_ISL_I5593715	Divisi6n Diagn6stico Molecular Hospital México	Divisi6n Diagn6stico Molecular Hospital México	Juan Carlos Villalobos Ugalde, Vanessa Villalobos Alfaro, Carlos Ramirez Chavarria
EPI_ISL_15593717	LESP State of Mexico	Instituto de Diagnostico y Referencia Epidemiologicos (INDRE)	Abril Rodrfguez-Maldonado; Claudia Wong-Arâmbula; Felipe Arguijo-Perez; Helios Cârdenas-Hernândez; Carmen Castra-Méndez; Lidia Garda-Torres; Ruth Madera-Sandoval; América Mandujano-Martfnez; Nancy Martfnez-Velâzquez; Mireya Mederos-Michel; Angélica Pedraza-Meléndez; Joaqufn Ouiroz-Mercado; Daniel Regalado-Santiago; Silvia Rivero-Arredondo; Erika Sierra-Atanacio; Fernando Gonzâlez-Domfnguez; Lucia Hernândez-Rivas, Irma L6pez-Martfnez; Ernesto Ramfrez-Gonzâlez; Maribel Gonzâlez-Villa
EPI_ISL_I5593718	LESP Mexico City	Instituto de Diagnostico y Referencia Epidemiologicos (INDRE)	Abril Rodrfguez-Maldonado; Claudia Wong-Arâmbula; Felipe Arguijo-Perez; Helios Cârdenas-Hernândez; Carmen Castro-Méndez; Lidia Garda-Torres; Ruth Madera-Sandoval; América Mandujano-Martfnez; Nancy Martfnez-Velâzquez; Mireya Mederos-Michel; Angélica Pedraza-Meléndez; Joaqufn Ouiraz-Mercado; Daniel Regalado-Santiago; Silvia Rivera-Arredondo; Erika Sierra-Atanacio; Fernando Gonzâlez-Domfnguez; Lucia Hernândez-Rivas, Irma L6pez-Martfnez; Ernesto Ramfrez-Gonzâlez: Maribel Gonzâlez-Villa
EPI_ISL_15593719	LESP Puebla	Instituto de Diagnostico y Referencia Epidemiologicos (INDRE)	Abril Rodrfguez-Maldonado; Claudia Wong-Arâmbula; Felipe Arguijo-Perez; Helios Cârdenas-Hernândez; Carmen Castra-Méndez; Lidia Garda-Torres; Ruth Madera-Sandoval; América Mandujano-Martfnez; Nancy Martfnez-Velâzquez; Mireya Mederos-Michel; Angélica Pedraza-Meléndez; Joaqufn Quiroz-Mercado; Daniel Regalado-Santiago; Silvia Rivero-Arredondo; Erika Sierra-Atanacio; Fernando Gonzélez-Domfnguez; Lucia Hernéndez-Rivas, Irma L6pez-Martfnez; Ernesto Ramfrez-Gonzâlez; Maribel Gonzâlez-Villa
EPI_ISL_I5593720	LESPTamaulipas	Instituto de Diagnostico y Referencia Epidemiologicos (INDRE)	Abril Rodriguez-Maldonado; Claudia Wong-Arâmbula; Felipe Arguijo-Perez; Helios Cérdenas-Hernandez; Carmen Castro-Méndez; Lidia Garda-Torres; Ruth Madera-Sandoval; América Mandujano-Martinez; Nancy Martfnez-Velazquez; Mireya Mederos-Michel; Angélica Pedraza-Meléndez; Joaqufn Quiroz-Mercado; Daniel Regalado-Santiago: Silvia Rivera-Arredondo; Erika Sierra-Atanacio: Fernando Gonzâlez-Domfnguez; Lucia Hernândez-Rivas, Irma L6pez-Martfnez: Ernesto Ramfrez-Gonzâlez; Maribel Gonzâlez-Villa
EPI_ISL_I5593721	LESP Baja California	Instituto de Diagnostico y Referencia Epidemiologicos (INDRE)	Abril Rodrfguez-Maldonado; Claudia Wong-Arâmbula; Felipe Arguijo-Perez; Helios Cârdenas-Hernândez; Carmen Castro-Méndez; Lidia Garda-Torres; Ruth Madera-Sandoval; América Mandujano-Martfnez: Nancy Martfnez-Velâzquez; Mireya Mederos-Michel; Angélica Pedraza-Meléndez; Joaqufn Ouiroz-Mercado; Daniel Regalado-Santiago: Silvia Rivero-Arredondo; Erika Sierra-Atanacio; Fernando Gonzâlez-Domfnguez; Lucia Hernândez-Rivas, Irma L6pez-Martinez: Ernesto Ramfrez-Gonzâlez: Maribel Gonzâlez-Villa
EPI_ISL_15593722	LESP Nuevo Lean	Instituto de Oiagnostico y Referencia Epidemiologicos (INDRE)	Abril Rodrfguez-Maldonado; Claudia Wong-Arâmbula; Felipe Arguijo-Perez; Helios Cârdenas-Hernândez: Carmen Castro-Méndez: Lidia Garda-Torres; Ruth Madera-Sandoval; América Mandujano-Martfnez; Nancy Martfnez-Velâzquez; Mireya Mederos-Michel: Angélica Pedraza-Meléndez; Joaqufn Quiroz-Mercado; Daniel Regalado-Santiago; Silvia Rivera-Arredondo; Erika Sierra-Atanacio: Fernando Gonzâlez-Domfnguez; Lucia Hernândez-Rivas, Irma L6pez-Martfnez: Ernesto Ramlrez-Gonzâlez; Maribel Gonzâlez-Villa
EPI_ISL_I5597039	Los Angeles County Public Health Laboratories	Los Angeles County Public Health Laboratories	P. Hemarajata et al.
EPJ ISL 15597040, EPI JSL 15597041, EPJ-ISL-15597042, EPI-ISL-15597043, EPÜSL)5597044. EPI=ISL)5597045, EPI_ISL_I5597046	UCtA Clinical Micro Lab	Los Angeles County Public Health Laboratories	P. Hemarajata et al.
EPJ ISL 15597047. EPI JSL 15597048, EPJ-ISL-15597049, EPI-ISL-15597050, EPI=ISL)5597051, EPI=ISL)5597052, EPJ ISL 15597054, EPI JSL 15597055, - - EPI_ISL_I559705-6	Ouest Diagnostics Nichais Institute	Los Angeles County Public Health Laboratories	P. Hemarajata et al.
EPJ ISL 15597058, EPI ISL 15597059, EPÜSL)5597060, EPI=ISL)5597061, EPI_JSL_I5597062, EPI_ISL_15597063	Los Angeles County Public Health Laboratories	Los Angeles County Public Health Laboratories	P. Hemarajata et al.
EPI_ISL_I5607969. EPI_ISL_15607970, EPJ_ISL_15607971. EPI_JSL_I5607972, EPI_ISL_15607973, EPI_ISL_15607974. EPI_ISL_15607975, EPI_ISL_15607976. EPI_ISL_I5607977. EPI_ISL_15607978, EPI_ISL_I5607979. EPI_ISL_15607980, EPI_ISL_15607981. EPI_ISL_I5607982, EPI_ISL_15607983, EPI_ISL_I 5607984. EPI_ISL_15607985, EPI_ISL_15607986. EPI_ISL_I5607987, EPI_ISL_15607988, EPI ISL 15607989, EPI ISL 15607990, EPJ ISL 15607991, EPI JSL 15608021, EPI ISL 15608022, EPI ISL 15608023, EPI ISL 15608024, EPI ISL 15608025, EPI ISL 15608026, EPI ISL 15608027, EPI ISL 15608028, EPI ISL 15608029, EPI ISL 15608030, EPI ISL 15608031, EPI ISL 15608032, EPI ISL 15608033, EPI ISL 15608034, EPI ISL 15608035, EPI ISL 15608036, EPI ISL 15608037, EPI=ISL=15608038, EPI=ISL)5608039, EPÜSL)5608040, EPI=JSL)5608041, EPI=ISL)5608042, EPI=ISL=15608043, EPI=ISL)5608044, EPÜSL)5608045, EPI=ISL)5608046, EPI=ISL)5608047, EPI=ISL=15608048, EPI=ISL)5608049, EPÜSL)5608050, EPI=ISL)5608051, EPI=ISL)5608052, EPI=ISL=15608053, EPI=ISL)5608054, EPÜSL)5608055, EPI=ISL)5608056, EPI=ISL)5608057, EPI_ISL_I5608058, EPI_ISL_I5608059			
see above	Laboratory Medicine, UW Virology	Laboratory Medicine. UW Virology	Sereewit.J., Xie.H. Roychoudhury.P. and Greninger.A.L.
EPI_ISL_15608908, EPI_ISL_15608909, EPI_JSL_I5608910, EPI_ISL_15608911	Southern Nevada Public Health Laboratory	Southern Nevada Public Health Laboratory	Michael Picker
EPJ_ISL_15617330. EPI_ISL_15617332, EPI_ISL_I5617333	Sequencing/Bioinformatics. Delaware Public Health Lab	Sequencing/Bioinformatics, Delaware Public Health Lab	Bajwa.M.I. and Miller,H.
EPI_ISL_15617335	Centers for Disease Contrai & Prevention (CDC), Division of High Consequence Pathogens and Pathology (DHCPP-PRB)	Centers for Disease Contrai & Prevention {CDC). Division of High Consequence Pathogens and Pathology (DHCPP-PRB)	Gigante,C.M., Vang,K., Zhao,H., Batra,D., Hetrick,E.E.. Howard,D.T., Kovar,L., Seabolt.M.H , Morrison,5.5., Desch,M., Knipe,K., Weigand,M.R., Sheth,M , Burgin,A., Burroughs,M., Lee,J., Wilkins,K., McCollum,A., Hutson,C., Davidson,W., Rao.A., Seely,K. and Li,Y.
EPI_ISL_15617336	Centers for Disease Contrai & Prevention (CDC), Division of High Consequence Pathogens and Pathology (DHCPP-PRB)	Centers for Disease Contrai & Prevention {CDC). Division of High Consequence Pathogens and Pathology (DHCPP-PRB)	Gigante,C.M , Francis,D., Zhao,H , Batra,D., Hetrick,E.E., Howard,D.T., Kovar,L. Seabolt.M.H , Morrison,S.S., Desch.M., Knipe.K., Weigand,M.R., Sheth,M., Burgin,A., Burraughs,M , Lee,J , Wilkins,K., McCollum,A., Hutson.C , Davidson.W., Rao.A.. Escobar,J. and Li,Y.
EPI_ISL_I5617337	Centers for Disease Contrai & Prevention (CDC), Division of High Consequence Pathogens and Pathology (DHCPP-PRB)	Centers for Disease Contrai & Prevention {CDC). Division of High Consequence Pathogens and Pathology (DHCPP-PRB)	Gigante,C.M , Ventura,J , Zhao,H , Batra,D., Hetrick,E.E., Howard,D.T., Kovar,L., Seabolt.M.H , Morrison,S.S., Desch,M., Knipe,K., Weigand,M.R. Sheth,M , Burgin,A., Burroughs,M., Lee,J , Wilkins,K., McCollum,A., Hutson,C., Davidson,W., Rao.A., Nash,J. and Li,Y.
EPI_ISL_15617338	Centers for Disease Contrai & Prevention (CDC), Division of High Consequence Pathogens and Pathology (DHCPP-PRB)	Centers for Disease Contrai & Prevention {CDC). Division of High Consequence Pathogens and Pathology (DHCPP-PRB)	Gigante,C.M., Lee,P., Zhao,H., Batra,D., Hetrick,E.E., Howard.D.T., Kovar,L., Seabolt,M.H., Morrison,S.S., Desch,M., Knipe,K., Weigand,M.R., Sheth,M., Burgin,A.. Burraughs,M., Lee,J., Wilkins,K., McCollum,A., Hutson,C., Davidson,W., Rao.A., Stanek,D. and Li,Y.
EPI_ISL_15617339	Centers for Disease Contrai & Prevention (CDC), Division of High Consequence Pathogens and Pathology (DHCPP-PRB)	Centers for Disease Contrai & Prevention {CDC). Division of High Consequence Pathogens and Pathology (DHCPP-PRB)	Gigante,C.M., Pavlick,J., Zhao,H., Batra,D., Hetrick,E.E., Howard,D.T., Kovar,L., Seabolt.M.H, Morrison,5.5., Desch,M., Knipe,K., Weigand,M.R, Sheth,M., Burgin,A., Burroughs,M., Lee,J., Wilkins,K., McCollum,A., Hutson,C., Davidson,W., Rao.A., Parrott,T. and Li,Y.

EPI_ISL_I5617340, EPI_ISL_I5617341	Centers for Disease Contrai & Prevention (CDC), Division of High Consequence Pathogens and Pathology (DHCPP-PRB)	Centers for Disease Contrai & Prevention (CDC). Division of High Consequence Pathogens and Pathology (DHCPP-PRB)	Gigante,C.M., Fisher,S., Zhao,H., Batra,D., Hetrick,E.E., Howard,D.T., Kovar,L., Seabolt,M.H., Morrison,5.5., Desch,M., Knipe,K., Weigand,M.R., Sheth,M., Burgin,A., Burroughs,M., Lee,J., Wilkins,K., McCollum,A., Hutson,C., Davidson,W., Rao.A.. Siebert,M. and Li,Y.
EPI_ISL_I5617342, EPI_ISL_I5617343, EPI_ISL_I5617344, EPI_ISL_I5617345	Centers for Disease Contrai & Prevention (CDC), Division of High Consequence Pathogens and Pathology (DHCPP-PRB)	Centers for Disease Contrai & Prevention {CDC). Division of High Consequence Pathogens and Pathology (DHCPP-PRB)	Gigante,C.M.. Ruiz.V., Zhao,H.. Batra,D., Hetrick,E.E., Howard,O.T., Kovar,L., Seabolt,M.H., Morrison,5.5., Desch,M., Knipe,K., Weigand,M.R., Sheth,M., Burgin,A., Burroughs.M., Lee,J., Wilkins,K., McCollum,A.. Hutson.C., Davidson,W., Rao.A., Wang,]. and U,Y.
EPJ ISL 15617346, EPI JSL 15617347, - - EPI_ISL_I56I734-8	Centers for Disease Contrai & Prevention (CDC), Division of High Consequence Pathogens and Pathology (DHCPP-PRB)	Centers for Disease Contrai & Prevention (CDCl. Division of High Consequence Pathogens and Pathology (DHCPP-PRB)	Gigante,C.M., Cogswell,K., Zhao,H., Batra,D., Hetrick,E.E., Howard,D.T., Kovar,L., Seabolt.M.H., Morrison,S.S., Desch,M., Knipe,K., Weigand,M.R., Sheth,M., Burgin,A., Burroughs,M., Lee,J., Wilkins,K., McCollum,A., Hutson,C., Davidson,W., Rao.A., Grenz.L. and U,Y.
EPI_I5L_I5617349, EPI_ISL_I5617350	Centers for Disease Contrai & Prevention (CDC), Division of High Consequence Pathogens and Pathology (DHCPP-PRB)	Centers for Disease Contrai & Prevention {CDC). Division of High Consequence Pathogens and Pathology (DHCPP-PRB)	Gigante,C.M.. Epie,N.. Zhao,H., Batra,D.. Hetrick,E.E.. Howard,D.T.. Kovar,L.. Seabolt,M.H.. Morrison,S.S.. Desch,M., Knipe,K.. Weigand,M.R.. Sheth,M.. Burgin,A.. Burroughs,M., Lee,J.. Wilkins,K.. McCollum,A., Hutson,C., Davidson,W., Rao.A., Perez,T. and Li,Y.
EPI_ISL_I5617351, EPI_ISL_I5617352	Centers for Disease Contrai & Prevention (CDC), Division of High Consequence Pathogens and Pathology (DHCPP-PRB)	Centers for Disease Contrai & Prevention (CDC). Division of High Consequence Pathogens and Pathology (DHCPP-PRB)	Gigante,C.M., Thomas,L., Zhao,H., Batra,D., Hetrick,E.E., Howard,D.T., Kovar,L., Seabolt,M.H., Morrison,5.5., Desch,M., Knipe,K., Weigand,M.R., Sheth,M., Burgin,A., Burroughs,M., Lee,J., Wilkins,K., McCollum,A., Hutson,C., Davidson,W., Rao.A.. Ounn,J. and Li,Y.
EPI_ISL_I5617353	Centers for Disease Contrai & Prevention (CDC), Division of High Consequence Pathogens and Pathology (DHCPP-PRB)	Centers for Disease Contrai & Prevention {CDC). Division of High Consequence Pathogens and Pathology (DHCPP-PRB)	Gigante,C.M.. Kubin,G.. Zhao,H., Batra,D.. Hetrick,E.E.. Howard,D.T.. Kovar,L., Seabolt,M.H.. Morrison,S.S.. Oesch,M., Knipe,K., Weigand,M.R., Sheth,M., Burgin,A.. Burroughs,M., Lee,J.. Wilkins,K., McCollum,A., Hutson,C., Davidson,W.. Rao.A., White,S.L. and U,Y.
EPI_ISL_I5617354, EPI_ISL_I5617355, EPI ISL 15617356, EPI ISL 15617357, - - EPI_ISL_I56I735-8	Centers for Disease Contrai & Prevention (CDC), Division of High Consequence Pathogens and Pathology (DHCPP-PRB)	Centers for Disease Contrai & Prevention {CDC). Division of High Consequence Pathogens and Pathology (DHCPP-PRB)	Gigante,C.M., Goldoft,M., Zhao.H., Batra,D.. Hetrick,E.E., Howard.DT.. Kovar,L.. Seabolt,M.H , Morrison,5.5.. Desch,M., Knipe,K.. Weigand,M.R. Sheth.M., Burgin,A., Burroughs,M., Lee.J., Wilkins.K., McCollum,A., Hutson,C., Davidson,W., Rao,A.. Holshue,M. and Li,Y.
EPI ISL 15641541, EPI ISL 15641542, EPJ ISL 15641543, EPI JSL 15641544, EPI ISL 15641545, EPI ISL 15641546, EPI ISL 15641547, EPI ISL 15641548, EPI ISL 15641549, EPI ISL 15641550, EPI ISL 15641551, EPI ISL 15641552, EPI ISL 15641553, EPI ISL 15641554, EPI ISL 15641555, EPI ISL 15641556, EPI ISL 15641557, EPI ISL 15641559, EPI ISL 15641560, EPI ISL 15641561, EPI-ISL-15641562, EPI-ISL-15641563, EPI-ISL-15641564, EPI-ISL-15641565, EPI-ISL-15641566, EPI-ISL-15641567, EPI-ISL-15641568, EPI-ISL-15641569, EPI-ISL-15641570, EPI-ISL-15641571, EPI-ISL-15641572, EPI-ISL-15641573, EPI-ISL-15641574, EPI-ISL-15641576, EPI-ISL-15641577, EPI-ISL-15641578, EPI-ISL-15641579, EPI-ISL-15641580, EPI-ISL-15641581, EPI-ISL-15641582, EPI-ISL-15641583, EPI-ISL-15641584, EPI-ISL-15641585, EPI-ISL-15641586, EPI-ISL-15641587, EPI-ISL-15641588, EPI-ISL-15641589, EPI-ISL-15641590, EPI-ISL-15641591, EPI-ISL-15641592, EPI-ISL-15641593, EPI-ISL-15641594, EPI-ISL-15641595, EPI-ISL-15641596, EPI-ISL-15641597, EPI-ISL-15641598, EPI-ISL-15641599, EPI-ISL-15641600, EPI-ISL-15641601, EPI-ISL-15641602, EPI=ISL)5641603, EPI=ISL)5641604, EPÜSL)5641605, EPI=I5L)5641606, EPI=ISL)5641607, EPI=ISL)5641608, EPI=ISL)5641609			
see above	Public Health Laboratory, Public Health Service Amsterdam, The Netherlands	Department of Medical Microbiology & Infection prevention, Amsterdam University Medical Centers location AMC	Matthijs Welkers,]elle Koopsen, Robin van Houdt, Marcel Jonges, Sebastien Matamoros, Sjoerd Rebers, Fokla Zorgdrager, Sylvia Bruisten, Akke Cornelissen, Janke Schinkel, Ewout Fanay, Roisin Bavalia, Menno de Jang and Mariken van der Lubben on behalf of the Amsterdam Regional Genomic epidemiology and Outbreak Surveillance (ARGOS) consortium
EPI_ISL_I5651836, EPI_ISL_I5651837, EPI_ISL_I5651839, EPI_ISL_I5651841, EPI_ISL_I5651842, EPI_ISL_I5651844. EPI_ISL_I5651846, EPI_ISL_I5651847, EPI_ISL_I5651849, EPI_ISL_I5651850, EPI_ISL_I5651852. EPI_ISL_I5651854, EPI_ISL_I5651856, EPI_ISL_I5651857, EPI_ISL_I5651859, EPI_ISL_I5651861			
see above	Centre for Biological Threats, Highly Pathogenic Viruses. Robert Koch Institute	Centre for Biological Threats, Highly Pathogenic Viruses, Robert Koch Institute	Brinkmann,A., Kohl,C., Pape,K., Schrick,L., Michel,J., Schaade,L. and Nitsche,A.
EPI_ISL_I5655944	Erasmus Medical Center Department of Virology	Erasmus Medical Center Oepartment of Virology	Leonard Schuele, Bas Oude Munnink, Marjan Soter, Babette Weller, Babs Verstrepen, Richard Molenkamp. Janette Rahamat-Langendoen, Reina Sikkema, Marion Koopmans
EPI_ISL_I5684645	Laboratory of Virology, University Hospitals of Geneva	Laboratory of Virology, University Hospitals of Geneva	Laubscher,F., Marques-Melancia,S., Cordey,S., Schibler,M.,Kaiser,L. and Renzoni,A.
EPI_ISL_I5701569	Institute for Medical Virology, University Hospital, Goethe University Frankfurt, Germany	Institute of Virology, Charité-Universiti"itsmedizin Berlin, Humboldt-Universiti"it zu Berlin. Germany	Denisa Bojkova, Julia Schneider, Victor M. Carman, Jindrich Cinatl jr.
EPI_ISL_I5702015, EPI_ISL_I5702711	Erasmus Medical Center Department of Virology	Erasmus Medical Center Oepartment of Virology	Leonard Schuele, Bas Oude Munnink, Marjan Soter, Babette Weller, Babs Verstrepen, Richard Molenkamp, Janette Rahamat-Langendoen, Reina Sikkema, Marion Koopmans
EPI_ISL_I5704688	Institute for Medical Virology, University Hospital, Goethe University Frankfurt, Germany	Institute of Virology, Charité-Universiti"itsmedizin Berlin, Humboldt-Universiti"it zu Berlin. Germany	Denisa Bojkova, Julia Schneider, Victor M. Carman, Jindrich Cinatl jr.
EPI_ISL_I5704845	Erasmus Medical Center Department of Virology	Erasmus Medical Center Oepartment of Virology	Leonard Schuele, Bas Oude Munnink, Marjan Soter, Babette Weller, Babs Verstrepen, Richard Molenkamp, Janette Rahamat-Langendoen, Reina Sikkema, Marion Koopmans
EPI ISL 15704861, EPI ISL 15705166, EPÜSL)5705315, EPI=ISL)5705498, EPI ISL 15705640, EPI ISL 15705771, EPI-ISL-15705945, EPI-ISL-15706105, ePC1s(_15706238, EPC1sL I5706380	Institute for Medical Virology, University Hospital, Goethe University Frankfurt, Germany	Institute of Virology, Charité-Universiti"itsmedizin Berlin, Humboldt-Universiti"it zu Berlin. Germany	Denisa Bojkova, Julia Schneider, Victor M. Carman, Jindrich Cinatl jr.
EPI_ISL_I5709387	Divisi6n de Microbiologfa, Hospital Nacional de Nii'ios Dr. Carlos Sâenz Herrera	Inciensa, Instituto de Investigaci6n y Ensei'ianza en Nutrici6n y Salud	Francisco Ouarte, Ana Isela Ruiz-Gonzalez, Hillary Serrano, Diana Cantillo, Claudio Soto-Garita, Gustavo Vega, Estela Cordera, Adriana Godinez, Melany Calderon & Cristian Pérez-Corrales
EPI_ISL_I5712853. EPI_ISL_I5712860, EPI_ISL_I5714234, EPI_ISL_I5714244, EPI_ISL_I5714245, EPI_ISL_I5714286. EPI_ISL_I5714287, EPI_ISL_I5714288. EPI_ISL_I5714299. EPI_ISL_I5714539, EPI_ISL_I5714920. EPI_ISL_I5714989, EPI_ISL_I5714990, EPI_ISL_I5715289. EPI_ISL_I5715292, EPI_ISL_I5715305. EPI_ISL_I5718514, EPI_ISL_I5718920			
see above	Erasmus Medical Center Department of Virology	Erasmus Medical Center Oepartment of Virology	Leonard Schuele, Bas Oude Munnink, Marjan Soter, Babette Weller, Babs Verstrepen, Richard Molenkamp, Janette Rahamat-Langendoen, Reina Sikkema, Marion Koopmans
EPI_ISL_I5722680, EPI_ISL_I5722681, EPI_ISL_I5722682, EPI_ISL_I5722683, EPI_ISL_I5722684, EPI_ISL_I5722685, EPI_ISL_I5722686, EPI_ISL_I5722687, EPI_ISL_I5722688, EPI_ISL_I5722689, EPI_ISL_I5722690			
see above	Rush University Medical Center	RIPHL at Rush University Medical Center	Stefan Green, Kevin Kunstman, Hannah Barbian, Felix Araujo Perez, Edith Perez, Sofiya Bobrovska, Alyse Kittner, Cecilia Chau, Giancarlo Balangue, Lok Yiu Ashley Wu, Mary Hayden, Joyce Houlihan, Diane Springer, Nicholas Moore
EPI_ISL_I5747900	WHO National Influenza Centre Russian Federation	WHO National Influenza Centre Russian Federation	Andrey Komissarov, Artem Fadeev, Nikita Yolshin, Evgeny Venev, Kseniya Komissarova, Daria Danilenko, Dmitry Lioznov
EPI_ISL_I5763810. EPI_ISL_I5763811, EPI_ISL_I5763812, EPI_ISL_I5763813, EPI_ISL_I5763814, EPI_ISL_I5763815. EPI_ISL_I5763816, EPI_ISL_I5763817. EPI_ISL_I5763818, EPI_ISL_I5763819, EPI_ISL_I5763820. EPI_ISL_I5763821, EPI_ISL_I5763822, EPI_ISL_I5763823, EPI_ISL_I5763824, EPI_ISL_I5763825. EPI_ISL_I5763826, EPI_ISL_I5763827. EPI_ISL_I5763828, EPI_ISL_I5763829, EPI_ISL_I5763830, EPI_ISL_I5763831. EPI_ISL_I5763832			
see above	National Virus Reference laboratory	National Virus Reference Laboratory	Gabriel Gonzalez, Michael Carr, Brian Keogan, Jose Maria Urtasun Elizari. Jonathan Dean, Daniel Hare, Cillian F De Gascun
EPI_ISL_I5802695. EPI_ISL_I5802696, EPI_ISL_I5802697. EPI_ISL_I5802698, EPI_ISL_I5802700, EPI_ISL_I5802703. EPI_ISL_I5802704, EPI_ISL_I5802705. EPI_ISL_I5802706, EPI_ISL_I5802707, EPI_ISL_I5802708			
see above	Direccion de Investigacion en Salud Publica, Instituto Nacional de Salud	Direccion de Investigacion en Salud Publica, Instituto Nacional de Salud	Laiton-Donato,K.D., Franco,C.E., Alvarez-Diaz.DA, Ruiz-Moreno,H.A., Prada,D.A., Martinez,D. and Mercado-Reyes,M.M.
EPI_ISL_I5802709	Animal Health, Istituto Zooprofilattico Sperimentale del Mezzogiorno	Animal Health, Istituto Zooprofilattico Sperimentale del Mezzogiorno	Viscardi.M.. Cozzolino.L.. De Martinis,(.. Cardillo,l. and fusco.G.
EPJ ISL 15802710, EPI JSL 15802711, EPI-ISL-15802712, EPI-ISL-15802713, EPI=ISL)5802714. EPI=ISL)5802715, EPI_I5L_I5802718, EPI_ISL_I5802721	Direccion de Investigacion en Salud Publica, Instituto Nacional de Salud	Direccion de Investigacion en Salud Publica, Instituto Nacional de Salud	Laiton-Donato,K.D., Franco,C.E., Alvarez-Diaz.DA, Ruiz-Moreno,H.A., Prada,D.A.. Martinez,D. and Mercado-Reyes,M.M.
EPI_ISL_I5802722. EPI_ISL_I5802723, EPJ_ISL_I5802724. EPI_JSL_I5802725. EPI_ISL_I5802726, EPI_ISL_I5802727. EPI_ISL_I5802728, EPI_ISL_I5802729. EPI_ISL_I5802730. EPI_ISL_I5802731, EPI_ISL_I5802732. EPI_ISL_I5802733, EPI_ISL_I5802734. EPI_ISL_I5802735. EPI_ISL_I5802736, EPI_ISL_I5802737. EPI_ISL_I5802738, EPI_ISL_I5802739. EPI_ISL_I5802740, EPI_ISL_I5802741, EPI_ISL_I5802742, EPI_ISL_I5802743			
see above	Centre for Biological Threats, Highly Pathogenic Viruses, Robert Koch Institute	Centre for Biological Threats, Highly Pathogenic Viruses, Robert Koch Institute	Brinkmann,A., Kohl,C., Pape,K., Schrick,L., Michel,J., Schaade,l. and Nitsche,A
EPI_ISL_15819624, EPI_ISL_15819625, EPI_ISL_15819626, EPI_ISL_15819627, EPI_ISL_15819628, EPI_ISL_15819629, EPI_ISL_15819630, EPI_ISL_15819631, EPI_ISL_15819634, EPI_ISL_15819635, EPI_ISL_15819636, EPI_ISL_15819637, EPI_ISL_15819638			
see above	California Department of Public Health	California Department of Public Health	Viral and Rickettsial Disease Laboratory
EPI_ISL_I5831211	Divisi6n Diagn6stico Molecular Hospital México	Divisi6n Diagn6stico Molecular Hospital México	Juan Carlos Villalobos Ugalde. Vanessa Villalobos Alfaro. Carlos Ramirez Chavarrfa
EPI_ISL_I5831212	Divisi6n Diagn6stico Molecular Hospital México	Divisi6n Diagn6stico Molecular Hospital México	Juan Carlos Villalobos Ugalde, Vanessa Villalobos Alfaro, Soffa Villalobos Abarca
EPI_ISL_I5855044, EPI_ISL_I5855046, EPI_ISL_I5855048, EPI_ISL_I5855050, EPI_ISL_I5855052, EPI_ISL_I5855054, EPI_ISL_I5855056, EPI_ISL_I5855058, EPI_ISL_I5855061, EPI_ISL_I5855063, EPI_ISL_I5855065, EPI_ISL_I5855067, EPI_ISL_I5855069			
see above	Centre for Biological Threats, Highly Pathogenic Viruses, Robert Koch Institute	Centre for Biological Threats, Highly Pathogenic Viruses, Robert Koch Institute	Brinkmann,A., Kohl,C., Pape,K., Schrick,l., Michel,]., Schaade,L. and Nitsche,A
EPI ISL 15889123, EPI ISL 15889124, EPI ISL 15889125, EPI ISL 15889127, EPI ISL 15889129, EPI ISL 15889130, EPI ISL 15889131, EPI ISL 15889132, EPI ISL 15889134, EPI ISL 15889135, EPI ISL 15889136, EPI ISL 15889138, EPI ISL 15889147, EPI ISL 15889148, EPI ISL 15889149, EPI ISL 15889151, EPI ISL 15889152, EPI ISL 15889155, EPI ISL 15889156, EPI ISL 15889158, EP(ISL)5889159. EPCtsL)5889160, EPÜSL)5889161. EP(JSL)5889163, EPI=ISL)5889164, EP(ISL)5889167. EPI=ISL)5889169			
see above	Public Health and Environmental Laboratories, New Jersey Department of Health	Public Health and Enviranmental Laboratories, New Jersey Department of Health	Palmateer.N.C.
EPI_ISL_I5889180	Centers for Disease Contrai & Prevention (CDC), Division of High Consequence Pathogens and Pathology (DHCPP-PRB)	Centers for Disease Contrai & Prevention {CDC). Division of High Consequence Pathogens and Pathology (DHCPP-PRB)	Gigante,(., Ingram.A., Zhao,H.. Batra.D., Hetrick.E., Howard,D., Kovar,L.. Seabolt.M.. Morrison.S , Desch,M., Knipe,K.. Weigand,M . Sheth.M.. Burgin.A.. Burroughs,M., Lee.J.. Wilkins.K.. McCollum.A., Hutson,C.. Davidson,W.. Rao.A.. Geeter,E. and U,Y.
EPI_ISL_I5889181, EPI_ISL_I5889182	Centers for Disease Contrai & Prevention (CDC), Division of High Consequence Pathogens and Pathology (DHCPP-PRB)	Centers for Disease Contrai & Prevention (CDC). Division of High Consequence Pathogens and Pathology (DHCPP-PRB)	Gigante,C., Ventura,J., Zhao,H., Batra,D., Hetrick,E., Howard,□.,Kovar,L., Seabolt.M., Morrison,S, Oesch,M., Knipe,K., Weigand,M, Sheth,M., Burgin,A., Burroughs,M., Lee,J., Wilkins,K., McCollum,A., Hutson,C., Davidson,W., Rao.A., Nash,J. and Li,Y.
EPI_ISL_I5889183	Centers for Disease Contrai & Prevention (CDC). Division of High Consequence Pathogens and Pathology (DHCPP-PRB)	Centers for Disease Contrai & Prevention {CDC). Division of High Consequence Pathogens and Pathology (DHCPP-PRB)	Gigante,C., Moreno-Gorrin,C., Zhao,H.. Batra,D., Hetrick,E.. Howard.D., Kovar,l., Seabolt,M., Morrison,S., Desch,M.. Knipe,K., Weigand,M, Sheth,M., Burgin,A.. Burroughs,M., Lee,J.. Wilkins,K., McCollum,A.. Hutson,C.. Davidson,W., Rao,A., Miller,H. and Li,Y.
EPI_JSL_I5889184, EPI_ISL_I5889185	Centers for Disease Contrai & Prevention (CDC), Division of High Consequence Pathogens and Pathology (DHCPP-PRB)	Centers for Disease Contrai & Prevention (CDC). Division of High Consequence Pathogens and Pathology (DHCPP-PRB)	Gigante,C., Lee,P., Zhao,H., Batra,D., Hetrick,E., Howard,□., Kovar,l., Seabolt.M., Morrison,S., Desch,M., Knipe,K., Weigand,M, Sheth,M., Burgin,A., Burroughs,M., Lee,J., Wilkins,K., McCollum,A., Hutson,C., oavidson,w., Rao,A., Stanek,D. and Li,Y.
EPI_ISL_15889186	Centers for Disease Contrai & Prevention (CDC),	Centers for Disease Contrai & Prevention (CDC).	Gigante,C., Salinas.A., Zhao,H., Batra,D., Hetrick,E., Howard,□.,Kovar,L., Seabolt.M., Morrison,S., Desch,M., Knipe,K., Weigand,M., Sheth,M., Burgin,A., Burroughs,M., Lee,J., Wilkins,K., McCollum,A., Hutson,C., Davidson,W., Rao.A.,

	Division of High Consequence Pathogens and Pathology (DHCPP·PRB)	Division of High Consequence Pathogens and Pathology (DHCPP·PRB)	Haydel,D. and Li,Y.
EPI_ISL_I5889187, EPI_ISL_I5889188	Centers for Disease Contrai & Prevention (CDC), Division of High Consequence Pathogens and Pathology (DHCPP-PRB)	Centers for Disease Contrai & Prevention {CDC). Division of High Consequence Pathogens and Pathology (DHCPP-PRB)	Gigante,C., Myers,R., Zhao,H., Batra,D., Hetrick,E., Howard,D., Kovar,L., Seabolt,M., Morrison,S., Desch,M., Knipe,K., Weigand,M , Sheth,M., Burgin,A., Burraughs,M., Lee,J., Wilkins,K., McCollum,A., Hutson,C., Davidson,W., Rao.A., Blythe,D. and Li,Y.
EPI_I5L_I5889189	Centers for Disease Contrai & Prevention (CDC), Division of High Consequence Pathogens and Pathology (DHCPP-PRB)	Centers for Disease Contrai & Prevention {CDC). Division of High Consequence Pathogens and Pathology (DHCPP-PRB)	Gigante,C. Cleavinger,K., Zhao,H., Batra,D., Hetrick,E., Howard,O., Kovar,L., Seabolt.M., Morrison,S., Desch,M., Knipe,K., Weigand,M , Sheth,M., Burgin,A., Burroughs,M., Lee,J., Wilkins,K., McCollum,A., Hutson,C., Davidson,W., Rao.A., Sinn,M. and Li,Y.
EPI_ISL_I5889190, EPI_ISL_I5889191, EPI_ISL_I5889192	Centers for Disease Contrai & Prevention (CDC), Division of High Consequence Pathogens and Pathology (DHCPP-PRB)	Centers for Disease Contrai & Prevention {CDC). Division of High Consequence Pathogens and Pathology (DHCPP-PRB)	Gigante,C., Kubin,G., Zhao,H., Batra,D., Hetrick,E., Howard,D., Kovar,L., Seabolt,M., Morrison,S., Desch,M., Knipe,K., Weigand,M., Sheth,M., Burgin,A., Burraughs,M., Lee,J., Wilkins,K., McCollum,A., Hutson,C., Davidson,W., Rao.A., White.$. and Li,Y.
EPI_ISL_I5889193, EPI_ISL_I5889194	Centers for Disease Contrai & Prevention (CDC), Division of High Consequence Pathogens and Pathology (DHCPP-PRB)	Centers for Disease Contrai & Prevention {CDC). Division of High Consequence Pathogens and Pathology (DHCPP-PRB)	Gigante,C., Griffin-Thomas,L., Zhao,H., Batra,0., Hetrick,E., Howard,D., Kovar,L., Seabolt,M., Morrison,S., Desch,M., Knipe,K., Weigand,M, Sheth,M., Burgin,A., Burroughs,M., Lee,J., Wilkins,K., McCollum,A., Hutson,C., Davidson,W., Rao.A., Crain,). and Li,Y.
EPI_ISL_I5889195	Centers for Disease Contrai & Prevention (CDC), Division of High Consequence Pathogens and Pathology (DHCPP·PRB)	Centers for Disease Contrai & Prevention {CDC). Division of High Consequence Pathogens and Pathology (DHCPP·PRB)	Gigante,C.. Achillies,C.. Zhao,H., Batra,D., Hetrick,E., Howard,D., Kovar,L., Seabolt,M.. Morrison,5., Desch,M., Knipe,K., Weigand,M , Sheth,M.. Burgin,A., Burroughs,M., Lee,J., Wilkins,K., McCollum.A., Hutson,C., Davidson,W., Rao.A., Blevins,L. and Li,Y.
EPI ISL 15896301, EPI ISL 15896302, EP1-=_1sL-=_15896303, EPi"_:JsL-=.15896305	Los Angeles County Public Health Laboratories	Los Angeles County Public Health Laboratories	P. Hemarajata et al.
EPI ISL 15896306, EPI ISL 15896307, EPI ISL 15896308, EPI ISL 15896309, EPI I5l 15896310, EPI ISL 15896311, EPI I5L 15896312, EPI ISL 15896313, EPI I5L 15896314, EPI ISL 15896315, EPI I5L 15896316, EPI I5L 15896317, EPI ISL 1S896318, EPI I5L 15896319, EPI ISL 1S896320, EPI I5L 15896321, EPI I5L 15896322, EPI ISL 1S896323, EPI I5L 15896324, EPI ISL 15896325, EPCISL)5896326, EPI=ISL)5896327			
see above	Kaiser Permanente China Hills Regional Reference Laboratories	Los Angeles County Public Health Laboratories	P. Hemarajata et al.
EPI_ISL_1S896329. EPI_ISL_I5896330, EPI_ISL_I5896331, EPI_ISL_I5896332, EPI_ISL_I5896334, EPI_ISL_I5896335. EPI_ISL_I5896336, EPI_ISL_I5896337, EPI_ISL_I5896338, EPI_ISL_I5896339, EPI_ISL_I5896340. EPI_ISL_I5896341, EPI_ISL_I5896342, EP1_15L_I5896343, EPI_ISL_I5896344, EPI_ISL_I5896345. EPI_ISL_I5896346, EPI_ISL_I5896347, EPI_I5L_I5896348, EPI_ISL_I5896349, EPI_ISL_I5896350, EPI_ISL_15896351, EPI_ISL_15896352, EPI_ISL_15896353			
see above	Los Angeles County Public Health Laboratories	Los Angeles County Public Health Laboratories	P. Hemarajata et al.
EPI_ISL_1S896817. EPI_ISL_I5896818, EPI_ISL_I5897122, EPI_ISL_I5897123, EPI_ISL_I5897124, EPI_ISL_I5897493. EPI_ISL_I5897494, EPI_ISL_I5897569, EPI_ISL_I5897570, EPI_ISL_I5897573, EPI_ISL_I5897587. EPI_I5L_I5897588, EPI_ISL_I5897589, EP1_15L_I5897590, EPI_ISL_I5897591			
see above	Landspitali Oepartment of Clinical Micrabiology	Landspitali Oepartment of Clinical Micrabiology	Freyja Valsdottir, Zarko Urasevic, Brynja Armannsdottir, Karl Gustaf Kristinsson
EPI ISL 15912046, EPI ISL 15912047, EPI ISL 15912048, EPI ISL 15912049, EPI ISL 15912050, EPI ISL 15912051, EPI ISL 15912052, EPI ISL 15912053, EPI ISL 15912054, EPI ISL 15912055, EPI ISL 15912056, EPI ISL 15912057, EPI ISL 15912058, EPI ISL 15912059, EPI ISL 15912060, EPI ISL 15912061, EPI ISL 15912062, EPI ISL 15912063, EPI ISL 15912064, EPI ISL 15912066, EPCISL)5912067. EPI=ISL)5912068, EPÜSL)5912069, EPCI5L)5912070, EPI=ISL)5912071, EPCISL)5912072. EPI=I5L)5912073, EPÜSL)5912074, EPCI5L)5912075, EPI=ISL)5912076, EPCISL)5912077. EPI=I5L)5912078, EPÜSL)5912079, EPCI5L)5912080, EPI=ISL)5912081, EPCISL)5912082. EPI=I5L)5912083, EPÜSL)5912084, EPCI5L)5912085, EPI=ISL)5912086, EPI ISL 15912087, EPI ISL 15912088, EPI ISL 15912089, EPI ISL 15912090, EPI ISL 15912091. EPI ISL 15912092, EPI ISL 15912093, EPI ISL 15912094, EPI ISL 15912095, EPI ISL 15912096, EPI ISL 15912097, EPI ISL 15912098, EPI ISL 15912099, EPI ISL 15912100, EPI ISL 15912101. EPI ISL 15912102, EPI ISL 15912103, EPI ISL 15912104, EPI ISL 15912105, EPI ISL 15912106, EPI=ISL)5912107, EP1=15L)5912108, EPÜSL)5912109, EP1=15L)5912110, EPI=ISL)5912111, EPI=ISL)5912112, EP1=15L)5912113, EPÜSL)5912114, EP1=15L)5912115, EPI=ISL)5912116, EPI=ISL)5912117, EP1=15L)5912118, EPÜSL)5912119, EPI=ISL)5912120, EPI=ISL)5912121, EPI=ISL)5912123, EPI=ISL)5912124, EPÜSL)5912125, EPI=ISL)5912126, EPI=ISL)5912127, EPI ISL 15912129, EPI ISL 15912130, EPI ISL 15912131. EPI ISL 15912132, EPI ISL 15912133, EPI ISL 15912134, EPI ISL 15912135, EPI ISL 15912137, EPI ISL 15912138, EPI ISL 15912140, EPI ISL 15912142, EPI ISL 15912143, EPI ISL 15912145, EPI ISL 15912146, EPI ISL 15912147, EPI ISL 15912148, EPI ISL 15912150, EPI ISL 15912151, EPI ISL 15912153, EPI ISL 15912154, EPI-ISL-15912156, EPI-ISL-15912157, EPI-ISL-15912159, EPI-ISL-15912160, EPI-ISL-15912164, EPI-ISL-15912165, EPI-ISL-15912166, EPI-ISL-15912167, EPI-ISL-15912168, EPI-ISL-15912169, EPI-ISL-15912170, EPI-ISL-15912171, EPI-ISL-15912174, EPI-ISL-15912175, EPI-ISL-15912176, EPI-ISL-15912177, EPI-ISL-15912181, EPI-ISL-15912182, EPI-ISL-15912183, EPI-ISL-15912184, EPCISL)5912185. EPI=ISL)5912187, EPÜSL)5912188, EPCISL)5912189, EPI=ISL)5912190, EPCISL)5912191. EPI=ISL)5912196, EPÜSL)59121 99, EPCISL)5912200, EPI=ISL)5912203, EPCISL)5912205. EPI=ISL)5912208, EPÜSL)5912209, EPCISL)5912211			
see above	DPH, Massachusetts State Public Health Laboratory	DPH, Massachusetts State Public Health Laboratory	Ooucette,M., Gagne,L. and Smole,S.C.
EPI ISL 15912317, EPI ISL 15912318, EPI ISL 15912319, EPI ISL 15912320, EPI ISL 15912321, EPI ISL 15912322, EPI ISL 15912323, EPI ISL 15912324, EPI ISL 15912325, EPI ISL 15912326, EPI ISL 15912327, EPI ISL 15912328, EPI ISL 15912329, EPI ISL 15912330, EPI ISL 15912331, EPI ISL 15912332, EPI ISL 15912333, EPI ISL 15912334, EPI ISL 15912335, EPI ISL 15912336, EPCISL)5912337. EPI=ISL)5912338, EPÜSL)5912339, EPCISL)5912340, EPI=ISL)5912341, EPCISL)5912342. EPI=ISL)5912343, EPÜSL)59123 44			
see above	Indian Council of Medical Research-National Institute of Viralogy, Micrabial Containment Complex	Indian Council of Medical Research-National Institute of Viralogy, Microbial Containment Complex	Pragya O. Yadav
EPI_I5L_I5912916	The Micrabiology laboratory, AUSL Romagna, Piesestina, Cesena, FC	Dipartimento di Medicina Specialistica Diagnostica e Sperimentale, University of Balogna Italy	Alessandra Scagliarini, Vittorio Sambri, MAria Elena Turba, Fabio Gentilini
EPI_I5L_I5942296	Sexually Transmitted Diseases (STDs) outpatient service of Dermatology Unit, Fondazione IRCCS Ca' Granda Ospedale Maggiore Policlinico of Milan	Bioinformatic lab, Scientific Institute IRCCS E. Medea	Diego Forni, Rachele Cagliani. Manuela Sironi, Chiara Moltrasio. Luigia Venegoni. Eleonora Quattri, Angelo Marzano
EPI ISL 15942637, EPI ISL 15942638, EPCisL-=_15942885, EPC1sL-=_1s942886	Sexually Transmitted Diseases (STDs) outpatient service of Dermatology Unit, Fondazione IRCCS Ca' Granda Ospedale Maggiore Policlinico of Milan	Bioinformatic Lab, Scientific Institute IRCCS E. Medea	Diego Forni, Rachele Cagliani, Manuela Sironi, Chiara Moltrasio, Luigia Venegoni, Eleonora Quattri, Angelo Marzano
EPI_ISL_I5950021. EPI_ISL_I5950022, EPI_ISL_I5950023, EPI_ISL_I5950024, EPI_ISL_I5950025, EPI_ISL_I5950026, EPI_ISL_I5950027, EPI_ISL_I5950029, EPI_ISL_I5950030, EPI_ISL_I5950031. EPI_ISL_I5950032			
see above	Rhode Island State Health Laboratory	Rhode Island State Health Laboratory	Kristin Carpenter-Azevedo, Sean Sierra-Patev. Richard C. Huard
EPI_ISL_I5955332, EPI_ISL_I5955333, EPI ISL 15955334, EPI ISL 15955335, eP1-=_1sL-=_1s955336, EPCisL-=_15955331	Kaiser Permanente China Hills Regional Reference Laboratories	Los Angeles County Public Health Laboratories	P. Hemarajata et al.
EPI_ISL_I5955338, EPI_ISL_I5955339	Los Angeles County Public Health Laboratories	Los Angeles County Public Health Laboratories	P. Hemarajata et al.
EPI_ISL_I5955340	Laboratory Corporation of America	Los Angeles County Public Health Laboratories	P. Hemarajata et al.
EPI_ISL_I5955341. EPI_ISL_I5955342, EPI_ISL_I5955343. EPI_ISL_I5955344, EPI_ISL_I5955345, EPI_ISL_I5955346. EPI_ISL_I5955347, EPI_ISL_I5955348. EPI_ISL_I5955349, EPI_ISL_I5955350, EPI_ISL_I5955351. EPI_ISL_I5955352, EPI_ISL_I5955353. EPI_ISL_I5955354. EPI_ISL_I5955355, EPI_ISL_I5955356. EPI_ISL_I5955357			
see above	Kaiser Permanente China Hills Regional Reference Laboratories	Los Angeles County Public Health Laboratories	P. Hemarajata et al.
EPI_ISL_I5965179	Institute of Microbiology, Universidad San Francisco de Quito	Institute of Microbiology, Universidad San Francisco de Quito	Belén Prado-Vivar, Mateo Carvajal. Sully Mtirquez. Erika B. Muiioz. Rommel Guevara, Maritza Paez, Estefania Rivadeneira, Evelyn Stinchez Espinoza. Josefina Coloma. Ver6nica Barragtin, Patricio Rojas-Silva, Gabriel Trueba. Michelle Grunauer, Pal.JI Ctirdenas
EPI_ISL_I6283050. EPI_ISL_I6283051, EPI_ISL_I6283052. EPI_ISL_I6283053. EPI_ISL_I6283054, EPI_ISL_I6283055. EPI_ISL_I6283056, EPI_ISL_I6283057. EPI_ISL_I6283058. EPI_ISL_I6283059, EPI_ISL_I6283060. EPI_ISL_I6283061, EPI_ISL_I6283062. EPI_ISL_I6283063. EPI_ISL_I6283064, EPI_ISL_I6283065. EPI_ISL_I6283066, EPI_ISL_I6283067. EPI_ISL_I6283068, EPI_ISL_I6283069, EPI_ISL_I6283070, EPI_ISL_I6283071, EPI_ISL_I6283072, EPI_ISL_I6283073, EPI_ISL_I6283074, EPI_ISL_I6283075, EPI_ISL_I6283076, EPI_ISL_I6283077, EPI_ISL_I6283078			
see above	National Reference Laboratory, Nigeria Centre for Disease Contrai	National Reference Laboratory, Nigeria Centre for Disease Contrai	Ndodo Nnaemeka, Olusola Anuoluwapo Akanbi, Chimaobi Chukwu, Oyeronke Ayansola, Adama Ahmad, Adesola Yinka-Ogunleye, Akinpelu Afolabi, Olajumoke Babatunde, Chikwe Ihekweazu, Ifedayo Adetifa, Matthew Mauldin, Crystal Gigante, Dhwani Batra, Michael Weigand, Dakota Howard, Mili Sheth, Matthew Seabolt, Yu Li
EPI_ISL_I6283079	Nigeria Centre for Disease Contrai, National Reference Laboratory	Nigeria Centre for Disease Contrai, National Reference Laboratory	Ndodo Nnaemeka, Olusola Anuoluwapo Akanbi, Chimaobi Chukwu, Oyeronke Ayansola, Adama Ahmad, Adesola Yinka-Ogunleye, Akinpelu Afolabi, Olajumoke Babatunde, Chikwe Ihekweazu, Ifedayo Adetifa, Matthew Mauldin, Crystal Gigante, Dhwani Batra, Michael Weigand, Dakota Howard, Mili Sheth, Matthew Seabolt, Yu Li
EPI_ISL_I8539019	Queensland Health Forensic and Scientific Services	Queensland Health Forensic and Scientific Services	Stewart.A., Schlebusch,S., Vlack,S., McMahon,J., Sullivan,M., Pyke,A. and Hajkowicz,K.
EPI_ISL_18812961, EPI_ISL_18812962	Centre for Biological Threats • Highly Pathogenic Viruses, Robert Koch Institute	Centre for Biological Threats • Highly Pathogenic Viruses, Robert Koch Institute	Brinkmann,A., Kohl,C., Pape,K., Schrick,L., Michel,)., Schaade,L. and Nitsche,A
EPI ISL 18812963, EPI ISL 18812964, EPÜSL)8812965. EPCISL=I8812966, EPI_ISL_I8812967	Centre for Biological Threats • Highly Pathogenic Viruses. Robert Koch Institute	Centre for Biological Threats • Highly Pathogenic Viruses. Robert Koch Institute	Brinkmann,A., Kohl,C., Pape,K., Bourquain,D., Thurmer,A., Michel,J., Schaade,L. and Nitsche,A.
