# Supplementary figures and images for: Structural insights into tecovirimat antiviral activity and poxvirus resistance
Source: Nat Microbiol. 2025 Feb 12;10(3):734–48. doi: 10.1038/s41564-025-01936-6 (PMC11879855; doi:10.1038/s41564-025-01936-6)

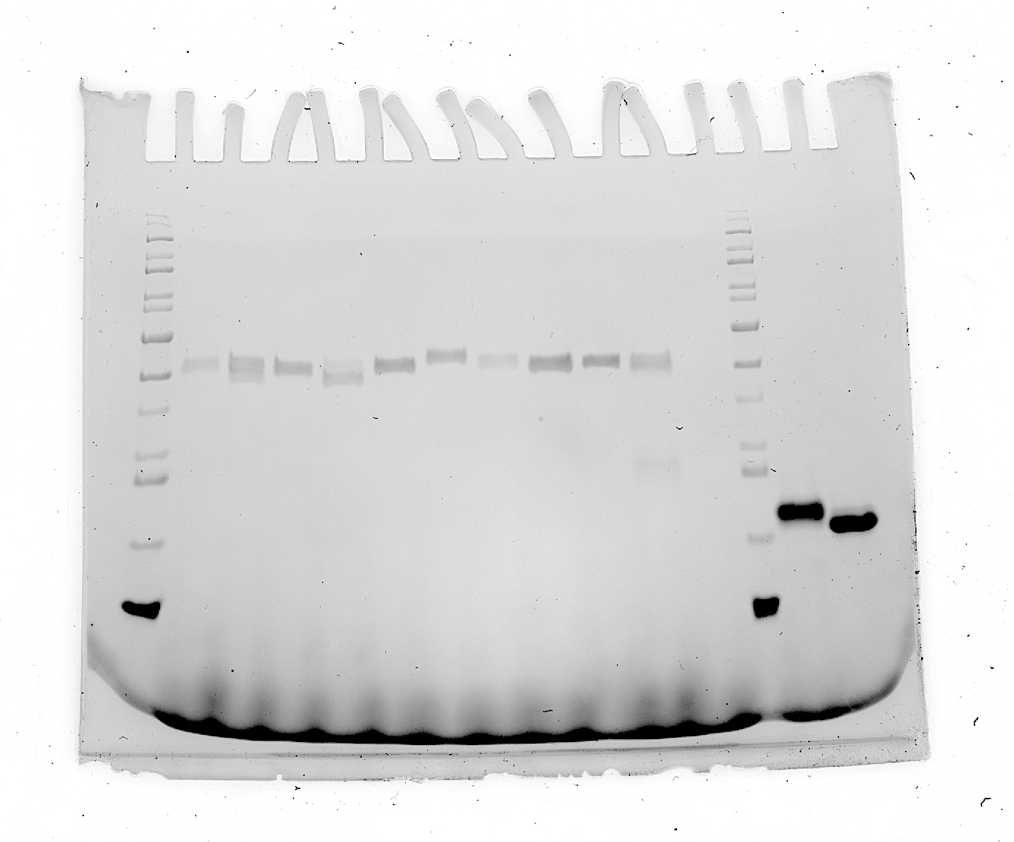

Supplement: Supplementary file 11 — Unprocessed SDS–PAGE. [file 41564_2025_1936_MOESM11_ESM.tif]
